# Sulfonamide-Controlled Pd(II)-Catalyzed [5+2] Rollover Annulation to Access (Hetero)pyrido-Fused 1‑Benzazepines

**DOI:** 10.1021/acs.orglett.6c02463

**Published:** 2026-07-27

**Authors:** Alejandro Suárez-Lustres, Jesús A. Varela, Carlos Saá

**Affiliations:** Centro Singular de Investigación en Química Biolóxica e Materiais Moleculares (CiQUS), Departamento de Química Orgánica, 16780 Universidade de Santiago de Compostela, 15782 Santiago de Compostela, Spain

## Abstract

A novel palladium-catalyzed rollover annulation is developed for the synthesis of pyrido-fused 1-benzazepines from *N*-sulfonyl-*N*-phenyl-2-aminopyridines and alkynes. Operating via a formal [5+2] cycloaddition with sequential aryl and heteroaryl C–H activations, the reaction relies on a key sulfonamide directing group to control periselectivity over competing [3+2] indole formation. The resulting benzazepines provide direct access to complex seven-membered N-heterocycles and serve as versatile intermediates for divergent skeletal editing into ring-expanded sultams and ring-contracted pyrroles.

## Graphical abstract

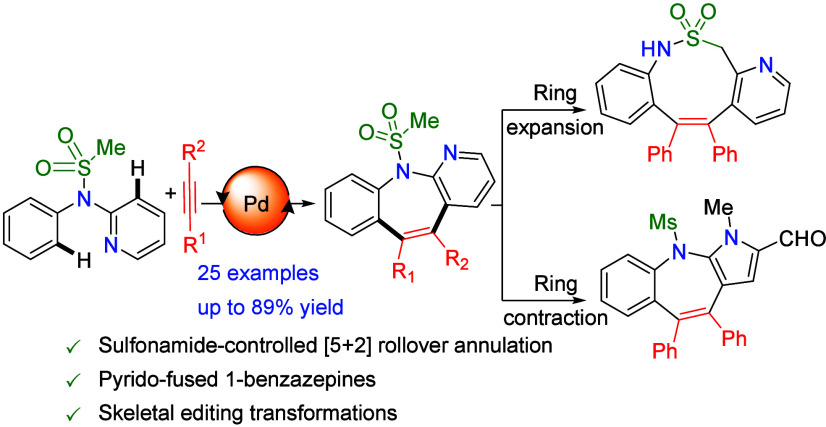

Medium-sized N-heterocycles, particularly benzo-fused seven-membered azaheterocycles, represent privileged structural motifs frequently encountered in biologically active molecules and pharmaceutical agents.^1^ Among them, pyridine-fused 1-benzazepines have attracted a considerable amount of attention because of their broad spectrum of biological activities, including HDAC6 inhibition^2^ and BRD4 modulation^3^ (Scheme 1A).

**Scheme 1:**
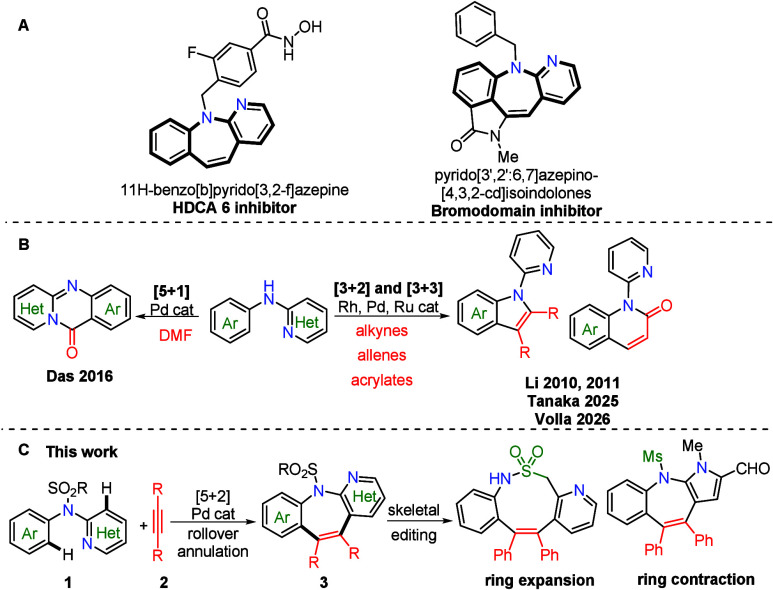
(A) Biologically Active Pyrido-Fused Benzazepines, (B) Metal-Catalyzed Oxidative Annulations of *N*-Phenyl-2-aminopyridine, and (C) Pd­(II)-Catalyzed [5+2] Rollover Annulation of *N*-Sulfonyl-*N*-phenyl-2-aminopyridine to Pyrido-Fused 1-Benzazepines

Despite the synthetic and medicinal relevance of these scaffolds, efficient methods for the construction of pyrido-fused benzazepines remain limited. Existing approaches often require prefunctionalized substrates, multistep cyclizations, or elaborate catalytic systems.^4^ Therefore, catalytic annulations based on C–H functionalization represent an attractive alternative for the rapid assembly of these heterocyclic frameworks. In particular, oxidative annulation reactions involving unsaturated coupling partners have made it possible to access medium-sized heterocyclic architectures efficiently.^5^ In this context, aminopyridines have proven to be especially versatile substrates because of their unique coordinative and electronic flexibility. Previous studies by Li, Tanaka, and Volla demonstrated that 2-aminopyridines may operate as three-atom synthons in Rh-, Pd-, and Ru-catalyzed annulations with alkynes, allenes, and acrylates, enabling formal [3+2] and [3+3] cyclizations.^6^ Likewise, Das and co-workers disclosed Pd-catalyzed annulation processes exploiting the intrinsic reactivity of aminopyridine derivatives toward heterocycle construction.^7^ In these systems, the free amino N–H bond plays a decisive role in directing reactivity and governing the formation of five- and six-membered heterocycles (Scheme 1B).

We envisaged that a nitrogen substituent could profoundly alter the coordination environment around palladium. This would enable a switch in the conventional annulation manifold and promote a [5+2] rollover annulation pathway through sequential C–H activation.^8^ Herein, we report a sulfonamide-controlled Pd-catalyzed periselective [5+2] rollover annulation for the efficient synthesis of pyrido-fused benzazepines from readily accessible aminopyridine derivatives and alkynes (Scheme 1C).^9^ Combined experimental and computational studies support a sequential rollover C–H activation pathway, revealing that the sulfonamide moiety plays a decisive role in governing annulation selectivity. The resulting pyrido-fused benzazepines serve as versatile synthetic intermediates that enable divergent skeletal editing into both ring-expanded sultams and ring-contracted pyrrole derivatives.

The mechanistic influence of the nitrogen substituent on annulation selectivity was initially examined using diphenylacetylene **2a** as a model coupling partner and AgOAc as an oxidant (Table 1).^9^ Under these conditions, the tertiary amine substrate (Z = Me) afforded desired 1-benzazepine **3** in only 10% yield together with indole **4** (12%), arising from a competing formal [3+2] annulation pathway (entry 1). The *N*-acetyl derivative (Z = Ac), previously employed in Pd-catalyzed C–H functionalization reactions,^10^ completely inhibited formation of the benzazepine scaffold (entry 2), indicating that simple electronic deactivation alone is insufficient to promote productive rollover reactivity.

**Table 1:**
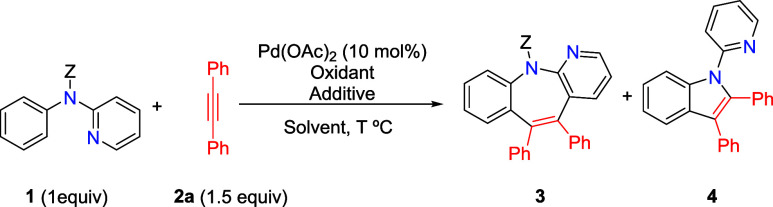
Optimization of the Reaction Conditions^a^,^b^

entry	Z	oxidant (equiv)	solvent	*T* (°C)	additive (equiv)	product (%)^c^
1	Me	AgOAc (2)	DMF	120	PivOH (5)	**3** (10), **4** (12)
2	Ac	AgOAc (3)	DCE	100	–	SM
3	Ms	AgOAc (2)	DMF	120	–	**3aa** (28)
4	Ms	Cu(OAc)_2_·H_2_O (2)	DMF	120	–	**3aa** (45)
5	Ms	Cu(OAc)_2_·H_2_O (2)	MeCN	105	PivOH (5)	**3aa** (67)
6	Ms	Cu(OAc)_2_·H_2_O (0.1)/O_2_ (1 atm)	MeCN	105	PivOH (5)	**3aa** (87) [78]
7	Me	Cu(OAc)_2_·H_2_O (0.1)/O_2_ (1 atm)	MeCN	105	PivOH (5)	**3** (6), **4** (33)
8	Ac	Cu(OAc)_2_·H_2_O (0.1)/O_2_ (1 atm)	MeCN	105	PivOH (5)	**4** (20)

a Typical conditions: **1** (0.2 mmol, 1 equiv), **2a** (0.3 mmol, 1.5 equiv), and Pd­(OAc)_2_ (10 mol %) in a solvent (2.0 mL) under an air atmosphere, unless otherwise stated.

b Reaction time of 72 h.

c Determined by ^1^H NMR analysis vs 1,3,5-trimethoxybenzene. Isolated yields are given in brackets.

We next turned our attention to sulfonamide-substituted aminopyridines, privileged pharmacophores in medicinal chemistry.^11^ Remarkably, all sulfonamide substrates completely suppressed formation of indole **4**, selectively promoting the formal [5+2] annulation pathway. Among the derivatives examined, methanesulfonamide substrate **1a** provided benzazepine **3aa** in 28% yield (entry 3).^12^ Replacement of AgOAc with Cu­(OAc)_2_·H_2_O increased the efficiency of the annulation process, and further improvement was achieved using MeCN as the solvent in the presence of PivOH, consistent with the beneficial role of carboxylate-assisted C–H activation pathways (entries 4 and 5).^13^ Importantly, use of catalytic Cu­(OAc)_2_·H_2_O under an O_2_ atmosphere^14^ further enhanced the efficiency of the transformation, affording benzazepine **3aa** in 78% isolated yield (entry 6).^15^ The structure of **3aa** was unambiguously confirmed by X-ray diffraction analysis.^16^ Control experiments under the optimized conditions further underscored the decisive role of the nitrogen substituent in governing annulation periselectivity. Whereas sulfonamide derivatives selectively underwent formal [5+2] rollover annulation, both tertiary amine (Z = Me) and acetamide (Z = Ac) substrates preferentially delivered indole **4** through competing formal [3+2] cyclization pathways (entries 7 and 8, respectively).

With the optimized conditions established, we next investigated the generality of the [5+2] rollover annulation process (Scheme 2). Symmetrical diaryl alkynes bearing either electron-donating groups or electron-withdrawing substituents efficiently participated in the transformation, furnishing the corresponding pyrido-fused benzazepines **3ab–3ai** in good to excellent yields. Notably, electron-deficient alkynes consistently exhibited higher reactivity, suggesting that enhanced alkyne electrophilicity facilitates the key migratory insertion events involved in the rollover annulation sequence. Importantly, halogen substituents were fully compatible with the catalytic conditions, as illustrated by brominated product **3ae**, thereby enabling further downstream functionalization.

**Scheme 2:**
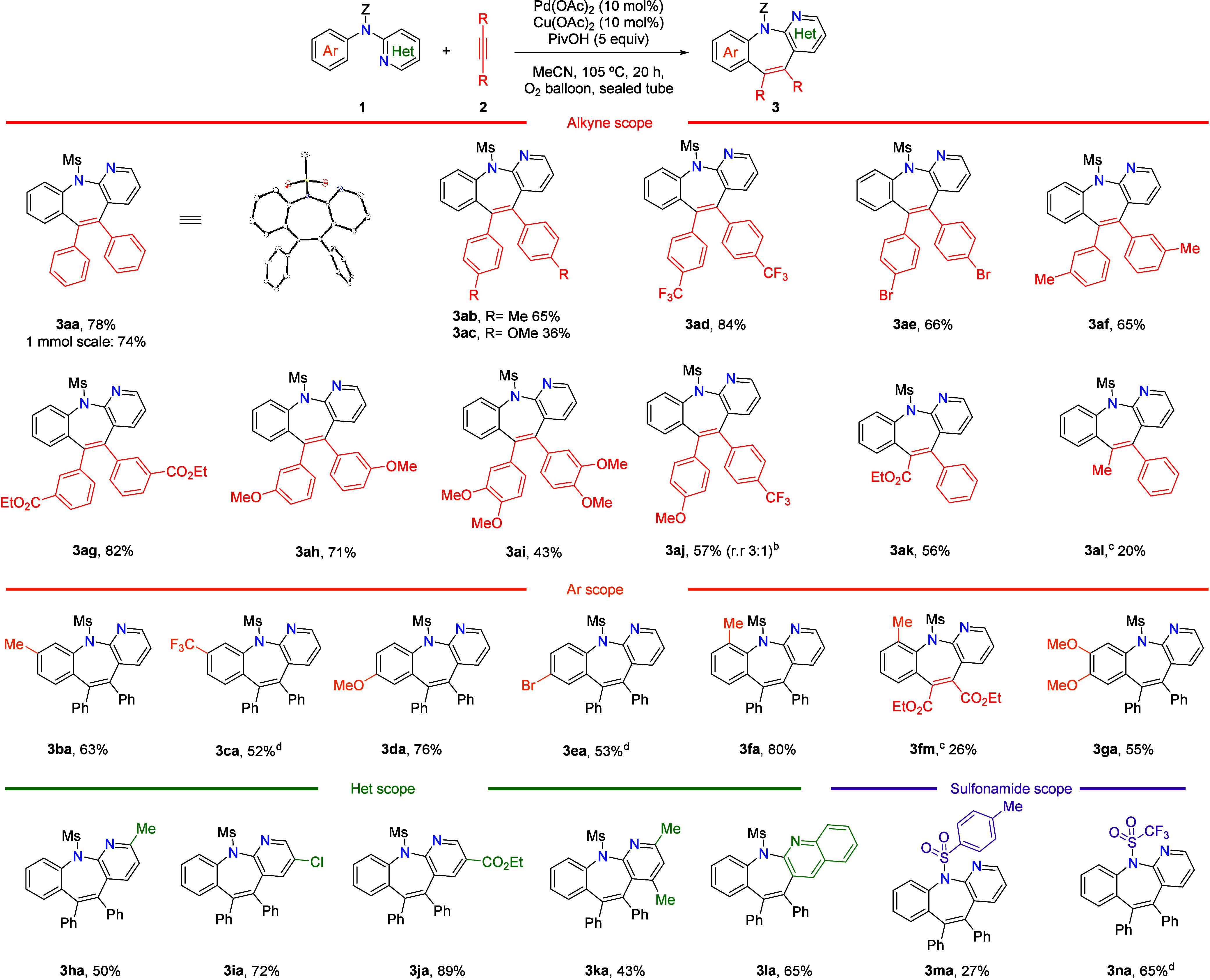
Scope of the Reaction^a^

a Reaction conditions: **1** (0.2 mmol, 1 equiv), **2** (0.3 mmol, 1.5 equiv), MeCN (0.1 M), 105 °C, 20 h, saturated with O_2_. Isolated yields are shown.
b Regioisomeric ratios were determined from isolated products.
c A total of 2.0 equiv of alkyne was used.
d Reaction time of 72 h. The ORTEP drawing of **3aa** shows ellipsoids at the 50% contour probability level.

Unsymmetrical diaryl alkynes bearing electronically differentiated aryl substituents also proved to be competent coupling partners. For example, substrate **2j** furnished benzazepine **3aj** as a mixture of regioisomers, indicating relatively modest regiocontrol during alkyne insertion. By contrast, conjugated alkynes such as ethyl 3-phenylpropiolate (**2k**) and 1-phenyl-1-propyne (**2l**) underwent highly regioselective annulation to afford products **3ak** and **3al**, respectively, in moderate to good yields. The enhanced regioselectivity observed for polarized alkynes likely reflects preferential migratory insertion into electronically differentiated Pd–C bonds during the rollover sequence.^17^

In contrast, dialkyl alkynes were unreactive under the optimized conditions for the [5+2] rollover annulation.^17^ This pronounced substrate dependence sharply contrasts with previously reported Pd-catalyzed formal [3+2] annulations of *N*-phenyl-2-aminopyridines, in which alkyl-substituted alkynes readily undergo indole-forming cyclization.^6c^ These observations strongly suggest that the present rollover annulation proceeds through a mechanistically distinct insertion manifold with substantially different electronic requirements.

We next examined the influence of the aminopyridine substitution pattern on the annulation efficiency. Both electron-rich and electron-deficient aryl substituents at the *meta* and *para* positions were readily accommodated, delivering benzazepines **3ba–3da** in consistently good yields. Halogenated substrates likewise remained compatible under the catalytic conditions, while sterically congested *ortho*-substituted and disubstituted systems also participated efficiently in the annulation process. Gratifyingly, the annulation process also tolerated more electronically activated alkynes such as diethyl acetylenedicarboxylate, although product **3fm** was obtained in only moderate yield.

Notably, substitution on the pyridine ring itself was well tolerated, providing products **3ha–3ja** in good yields and indicating the substantial flexibility of the rollover activation manifold toward electronic perturbation of the azaheterocycle. More sterically demanding substrates, including quinoline-derived systems, also underwent productive annulation to furnish products **3ka** and **3la**, demonstrating that the sequential C–H activation pathway can accommodate extended π-conjugated azaarenes.

Finally, variation of the sulfonamide substituent revealed a pronounced protecting group effect on annulation efficiency. Although tosyl- and triflyl-protected substrates afforded desired products **3ma** and **3na**, respectively, the methanesulfonamide derivative consistently provided superior reactivity and selectivity. This observation further supports the critical mechanistic role of the sulfonamide group in modulating the catalytic reaction manifold and enabling an efficient rollover annulation.

To gain mechanistic insight into the origin of the observed periselectivity, a series of experimental and computational studies were undertaken.^18^ DFT calculations were performed for the reaction of substrate **1a** with alkyne **2a** using Pd­(OAc)_2_ as the catalyst in the presence of AcOH in MeCN (Scheme 3). The initial C–H activation step followed by alkyne coordination and migratory insertion into the Pd–C bond generates intermediate **VI**, a six-membered palladacycle, that is substantially stabilized by transient coordination of the sulfonamide moiety. This stabilization proved to be critical for productive rollover annulation.^19^

**Scheme 3:**
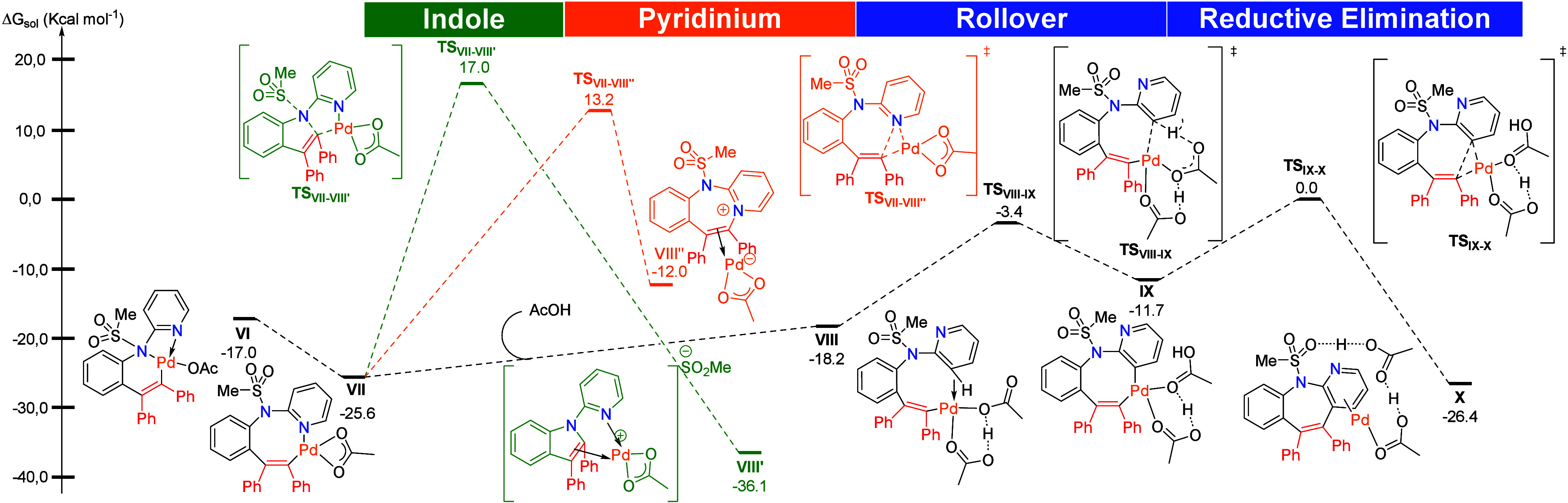
Free Energy Profile for the Pd-Catalyzed Reaction of *N*-Methanesulfonyl-*N*-phenyl-2-aminopyridine with **2a**

A key mechanistic feature emerges upon reorganization of the acetate ligand from monodentate X-type to bidentate (L,X)-coordination mode, releasing the sulfonamide donor and generating eight-membered intermediate **VII**. This species constitutes the selectivity-determining branching point of the catalytic cycle. From **VII**, acetate-assisted rollover C–H activation proceeds through **TS**_**VIII–IX**_ with a Δ*G* ^⧧^ of 22.2 kcal/mol, enabling the formation of palladacycle **IX** through sequential ligand decoordination/recoordination characteristic of rollover cyclometalation. Subsequent reductive elimination easily furnishes benzazepine **3aa** and the Pd(0) catalyst.

Importantly, the competing pathways leading to formal [3+2] annulation and nonrollover [5+2] cyclization products are both kinetically disfavored relative to the rollover pathway (**TS**_**VII–VIII′**_ and **TS**_**VII–VIII″**_ vs **TS**_**IX‑X**_). The origin of this periselectivity can be traced to the strong electron-withdrawing nature of the sulfonamide substituent, which destabilizes the ionic character required for indole-forming reductive elimination while simultaneously favoring the neutral rollover annulation manifold. In contrast, replacing the sulfonamide group (N = SO_2_R) with an electron-donating methyl substituent (N = Me) dramatically alters the reaction profile. In this case, reductive elimination leading to indole formation becomes significantly more favorable owing to stabilization of the corresponding ionic transition state.^20^ These computational results are fully consistent with the experimental observations, in which tertiary amine substrates preferentially undergo formal [3+2] annulation, whereas sulfonamide derivatives selectively furnish pyrido-fused benzazepines through the rollover [5+2] pathway.

The synthetic versatility of pyrido-fused benzazepine **3aa** was next explored through a series of derivatization studies (Scheme 4). Reductive deamination was successfully accomplished using LiAlH_4_ in THF under reflux conditions, affording pyridoazepine **5** in 55% yield (Scheme 4, eq 1).^21^ Interestingly, attempts to access the same product via base-mediated cleavage of the sulfonamide led instead to unexpected ring-expanded sulfonamide **6** in an excellent 84% yield (Scheme 4, eq 2).^22^ The structure of sultam **6** was unambiguously confirmed by X-ray diffraction analysis.

**Scheme 4:**
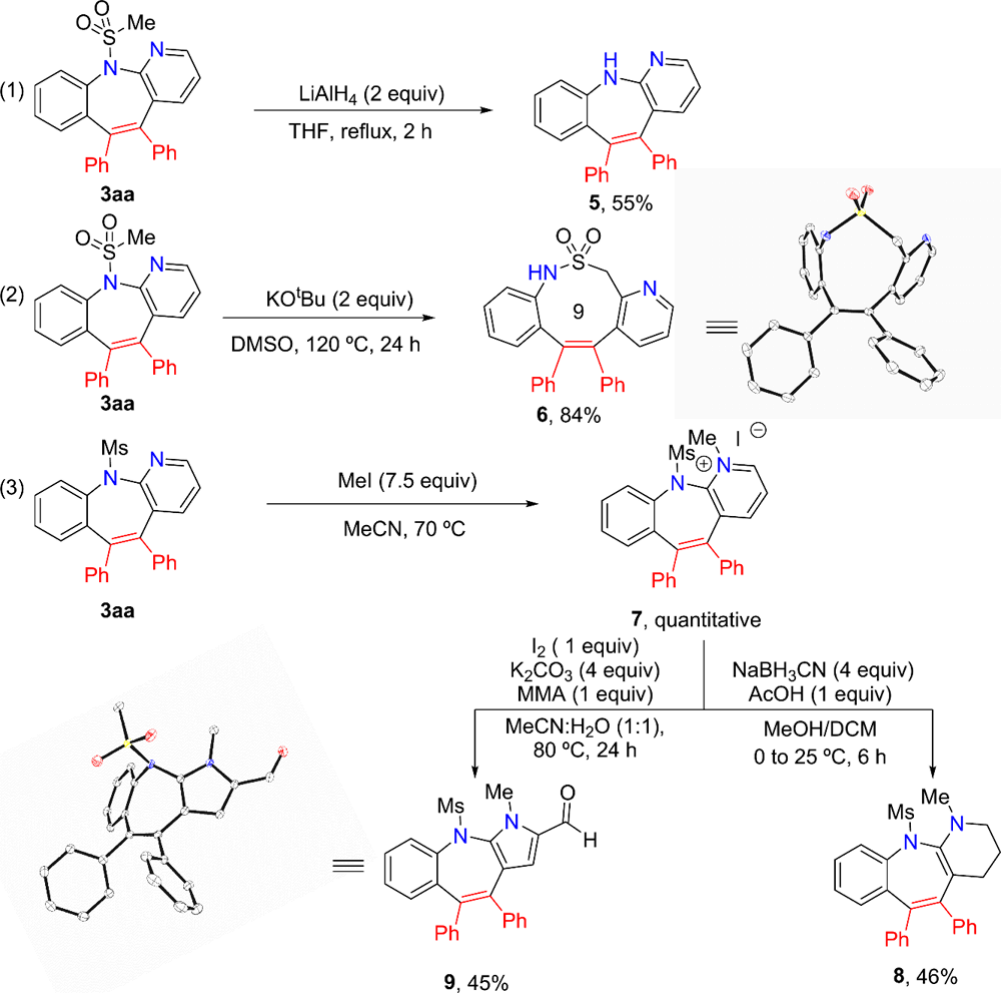
Derivatizations of Pyrido-Fused Benzazepine **3aa**

Further functionalization of **3aa** was achieved through alkylation with methyl iodide, which furnished pyridinium salt **7**. Subsequent reduction with NaBH_3_CN in a MeOH/DCM mixture provided tetrahydro derivative **8**, in a moderate 46% yield over two steps (Scheme 4, eq 3).^23^

Pleasingly, we achieved a skeletal editing transformation involving ring contraction of electron-deficient pyridinium intermediate **7**. Treatment under I_2_-mediated conditions in the presence of water triggered a pyridinium ring-opening/cyclization sequence, ultimately delivering electron-rich pyrrolo-1-benzazepine **9** ^16^ in a reasonable 45% yield (Scheme 4, eq 3).^24^

In conclusion, a versatile palladium-catalyzed rollover annulation strategy that enables the efficient synthesis of benzo­[*b*]­pyrido­[3,2-*f*]­azepines from *N*-sulfonyl-*N*-phenyl-2-aminopyridines and alkynes has been developed. This transformation proceeds via a formal [5+2] cycloaddition involving sequential C–H activation at both aryl and heteroaryl positions. Mechanistic studies support a key role of the sulfonamide directing group in governing periselectivity, favoring the rollover annulation pathway over competing [3+2] cycloaddition leading to indole formation.

The resulting pyrido-fused benzazepines serve as versatile scaffolds, readily amenable to structural diversification into both ring-expanded nine-membered sultams and ring-contracted pyrrole frameworks, highlighting their utility in skeletal editing. Overall, this work establishes a mechanistically distinct C–H activation manifold for the construction of seven-membered N-heterocycles and expands the scope of palladium-catalyzed annulation chemistry.

## Supplementary Material



## Data Availability

The data underlying this study are available in the published article and its Supporting Information.

## Notes

General experimental procedures, NMR spectra, computational details, and Cartesian coordinates for all calculated species (PDF)
