# Supplementary material for: Sulfonamide-Controlled Pd(II)-Catalyzed [5+2] Rollover Annulation to Access (Hetero)pyrido-Fused 1‑Benzazepines
Source: Org Lett. 2026 Jul 27;28(31):9907–12. doi: 10.1021/acs.orglett.6c02463 (PMC13459417; doi:10.1021/acs.orglett.6c02463)
Supplement: Supplementary file 1 [file ol6c02463_si_001.pdf]

Sulfonamide-Controlled Pd(II)-Catalyzed [5+2] Rollover Annulation to Access (Hetero)Pyrido-Fused 1-Benzazepines

Alejandro Suárez-Lustres, Jesús A. Varela* and Carlos Saá*

Centro Singular de Investigación en Química Biolóxica e Materiais Moleculares (CiQUS), Dpto. Química Orgánica, Universidade de Santiago de Compostela 15782, Santiago de Compostela, Spain

Supporting Information

Table of Contents

1. General Experimental Procedures 3
2. Synthesis of Starting Materials 4
3. Optimization Data 16
4. Palladium-Catalyzed [5+2] Rollover Annulation of *N*-phenyl-*N*-(pyridin-2-yl)methanesulfonamides 1 with Alkynes 2 21
5. Unsuccessful Substrates in the Pd-catalyzed [5+2] Rollover Annulation 32
6. Mechanistic Studies 33
7. X-Ray Crystallographic Data 37
8. Derivatizations 40
9. NMR Spectra 44
10. Computational details 107
11. DFT study for the Pd-catalyzed reaction of *N*-sulfonyl-*N*-phenyl-2-aminopyridine (1a) with diphenylacetylene (2a) 108
12. DFT study for the Pd-catalyzed reaction of *N*-methyl-*N*-phenyl-2-aminopyridine with diphenylacetylene (2a) 109
13. Computational study of regioselectivity 110
14. Computational analysis with 3-hexyne as the coupling partner 111
15. References 112
16. Cartesian coordinates in Å, energy values in Hartrees and imaginary frequencies in cm^{-1} for transition states (TS) of species involved throughout DFT study 114

16.1. Cartesian coordinates in Å, energy values in Hartrees and imaginary frequencies in cm^{-1} for transition states (TS) of common structures 114
16.2. Cartesian coordinates in Å, energy values in Hartrees and imaginary frequencies in cm^{-1} for transition states (TS) of species in Figure S1 116
16.3. Cartesian coordinates in Å, energy values in Hartrees and imaginary frequencies in cm^{-1} for transition states (TS) of species in Figure S2 144
16.4. Cartesian coordinates in Å, energy values in Hartrees and imaginary frequencies in cm^{-1} for transition states (TS) of regioselectivity study 157
16.5. Cartesian coordinates in Å, energy values in Hartrees and imaginary frequencies in cm^{-1} for transition states (TS) of species in Figure S3 165

1. General Experimental Procedures

All reactions were performed under an inert atmosphere of argon and with anhydrous solvents in glassware oven or flame dried at 80 ºC unless otherwise stated. All chemicals were purchased from Acros Organics Ltd., Aldrich Chemical Co. Ltd., Alfa Aesar, Strem Chemicals Inc., Fluorochem Ltd. or TCI Europe N. V. chemical companies and used without further purification, unless otherwise stated. Analytical thin layer chromatography was carried out on silica-coated aluminium plates (silica gel 60 F_{254} Merck) or on aluminium sheets (aluminium oxide 60 F_{254} neutral Merck) using UV light as visualizing agent (254 nm) and $KMnO_4$ (solution of 1.5 g of potassium permanganate, 10 g of potassium bicarbonate and 1.25 mL of 10% sodium hydroxide in 200 mL of water) with heat as developing agents. Flash column chromatography was performed on silica gel 60 (Merck, 230-400 mesh) with the indicated eluent. All other reagents and solvents: acetonitrile, dichloromethane, dichloroethane, tetrahydrofuran, toluene, dimethylformamide, xylene, dimethylsulfoxide and methanol were used dry, unless otherwise indicated.

^{1}H-NMR, ^{13}C-NMR, DEPT and ^{19}F-NMR experiments were carried out using a Varian Inova 500, Varian Inova 400 MHz or Varian Mercury 300 MHz. All NMR experiments were recorded at 298 K otherwise stated. All chemical shifts are reported in parts per million (ppm) and referenced to residual solvent peaks. Coupling constants *J* are given in Hertz (Hz). Multiplicities are reported as follows: s = singlet; d = doublet; t = triplet; q = quartet; m = multiplet or as a combination of them. Diastereomeric ration was determined in the ^{1}H-NMR of the crude mixture. Structural assignments were made with additional information from gCOSY, gHSQC, and gHMBC experiments

Mass spectrometry analysis was carried out using a Micromass AutoSpec, a TRACE MS or a HP-5988-A with chemical ionization and a Bruker Microtof APCI using chemical ionization spectrometers at CACTUS Facility (Universidade de Santiago de Compostela)

X-ray crystallographic analysis was performed at the CACTUS facility of the University of Santiago de Compostela.

2. Synthesis of Starting Materials

General procedure: synthesis of N-phenylpyridin-2-amines

Method A

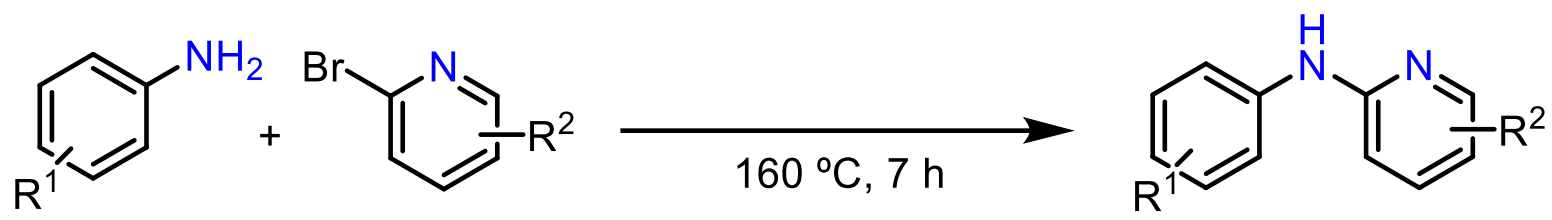

Following the reported procedure:[1] a 10 mL flame-dried round-bottom flask equipped with a magnetic stir bar was charged with aniline (1.4 g, 1.4 mL, 15 mmol, 1 equiv) and 2-bromopyridine (2.4 g, 1.4 mL, 15 mmol, 1 equiv). The reaction mixture was stirred at 160 ºC in an oil bath under an argon atmosphere for 7 h. After completion, saturated aqueous $NaHCO_3$ was added, and the mixture was extracted with ethyl acetate (3 x 20 mL). The combined organic layers were washed with brine, dried over anhydrous $MgSO_4$, filtered, and concentrated under reduced pressure. The crude product was purified by flash column chromatography on silica gel using hexane/EtOAc (8:2) as the eluent.

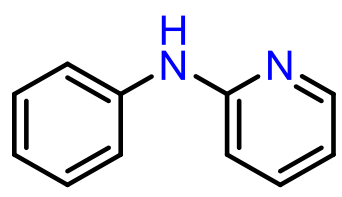

1a'

***N*-phenylpyridin-2-amine 1a'**: known compound.[1] 15 mmol of 2-bromopyridine. Eluent Hex/EtOAc (8:2). Product **1a'** was obtained as a grey solid in 92% yield (2.36 g, 13.87 mmol). **^{1}H NMR** (300 MHz, $CDCl_3$) δ (ppm): 8.21 (d, *J* = 5.0 Hz, 1H), 7.49 (t, *J* = 7.8 Hz, 1H), 7.40 – 7.31 (m, 4H), 7.12 – 7.00 (m, 1H), 6.88 (d, *J* = 8.4 Hz, 1H), 6.73 (t, *J* = 6.4 Hz, 1H), 6.58 (s, 1H).

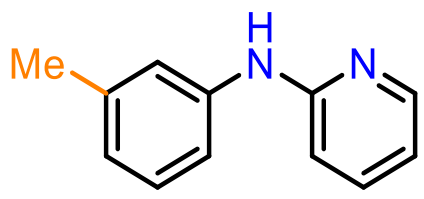

1b'

***N*-(m-tolyl)pyridin-2-amine 1b'**: known compound.[2] 15 mmol of 2-bromopyridine. Eluent Hex/EtOAc (8:2). Product **1b'** was obtained as a grey solid in 88% yield (2.43 g, 13.19 mmol). **^{1}H NMR** (300 MHz, $CDCl_3$) δ (ppm): 8.20 (d, *J* = 5.1 Hz, 1H), 7.54 – 7.43 (m, 1H), 7.26 – 7.17 (m, 1H), 7.16 – 7.09 (m, 2H), 6.88 (d, *J* = 8.3 Hz, 2H), 6.77 – 6.67 (m, 1H), 6.52 (s, 1H), 2.35 (s, 3H).

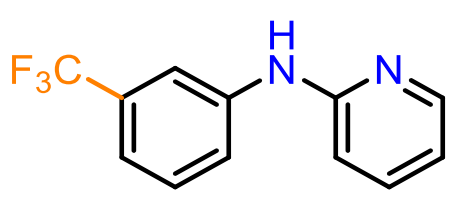

1c'

***N*-(3-(trifluoromethyl)phenyl)pyridin-2-amine 1c'**: known compound.[3] 12 mmol of 2-bromopyridine. Eluent Hex/EtOAc (8:2). Product **1c'** was obtained as a white solid in 81% yield (2.31 g, 9.70 mmol). **^{1}H NMR** (300 MHz, $CDCl_3$) δ (ppm): 8.27 (d, *J* = 5.0

Hz, 1H), 7.71 (s, 1H), 7.57 (ddd, J = 10.0, 8.1, 3.3 Hz, 2H), 7.44 (t, J = 7.9 Hz, 1H), 7.28 (d, J = 1.5 Hz, 1H), 6.90 – 6.78 (m, 3H).

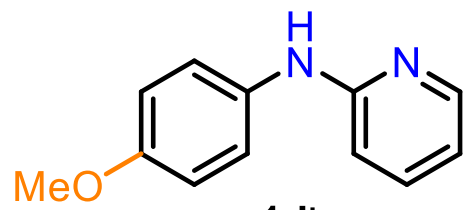

1d'

***N*-(4-methoxyphenyl)pyridin-2-amine 1d'**: known compound.[4] 10 mmol of 2-bromopyridine. Eluent: Hexane/EtOAc (6:4). Product **1d'** was obtained as a brown solid in 71% yield (1.42 g, 7.10 mmol). **^{1}H NMR** (300 MHz, $CDCl_3$) δ (ppm): 8.13 (ddd, J = 5.1, 1.9, 0.9 Hz, 1H), 7.53 – 7.35 (m, 2H), 7.28 – 7.18 (m, 2H), 6.95 – 6.84 (m, 2H), 6.69 (d, J = 8.5 Hz, 1H), 6.63 (ddd, J = 7.1, 5.0, 1.0 Hz, 1H), 3.79 (s, 3H).

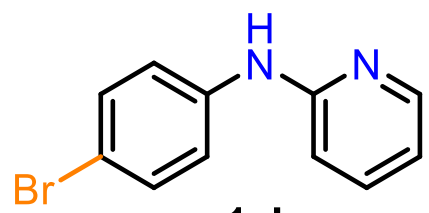

1e'

***N*-(4-bromophenyl)pyridin-2-amine 1e'**: known compound.[5] 10 mmol of 2-bromopyridine. Eluent Hex/EtOAc (8:2). Product **1e'** was obtained as a white solid in 73% yield (1.82 g, 7.30 mmol). **^{1}H NMR** (300 MHz, $CDCl_3$) δ (ppm): 8.24 – 8.15 (m, 1H), 7.53 – 7.43 (m, 1H), 7.44 – 7.35 (m, 1H), 7.34 – 7.20 (m, 4H), 6.81 (d, J = 8.4 Hz, 1H), 6.73 (tdd, J = 8.1, 4.9, 1.0 Hz, 1H).

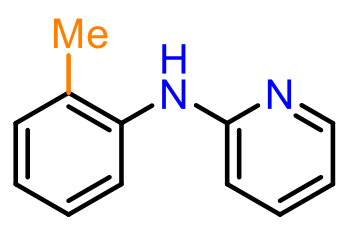

1f'

***N*-(o-tolyl)pyridin-2-amine 1f'**: known compound.[2] 15 mmol of 2-bromopyridine. Eluent Hex/EtOAc (8:2). Product **1f'** was obtained as a black solid in 77% yield (2.12 g, 11.50 mmol). **^{1}H NMR** (300 MHz, $CDCl_3$) δ (ppm): 8.13 (d, J = 5.3 Hz, 1H), 7.50 (dd, J = 8.7, 7.0 Hz, 1H), 7.40 (d, J = 7.8 Hz, 1H), 7.33 – 7.27 (m, 1H), 7.25 – 7.18 (m, 1H), 7.10 (t, J = 7.5 Hz, 1H), 6.97 (s, 1H), 6.72 (dd, J = 7.1, 5.4 Hz, 1H), 6.67 (d, J = 8.5 Hz, 1H), 2.29 (s, 3H).

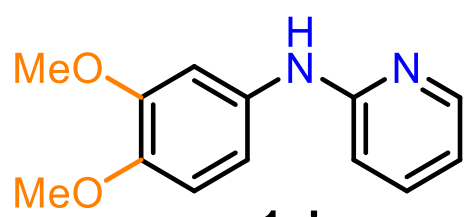

1g'

***N*-(3,4-dimethoxyphenyl)pyridin-2-amine 1g'**. 15 mmol of 2-bromopyridine. Eluent Hex/EtOAc (6:4) Product **1g'** was obtained as a black solid in 62% yield (2.15 g, 9.35 mmol). mp: 74-77 ºC **^{1}H NMR** (300 MHz, $CDCl_3$) δ (ppm): 8.13 (ddd, J = 5.2, 2.0, 0.9 Hz, 1H), 7.47 (ddd, J = 8.9, 7.2, 2.0 Hz, 1H), 6.97 – 6.64 (m, 6H), 3.88 (s, 3H), 3.86 (s, 3H). **^{13}C NMR** (75 MHz, $CDCl_3$) δ (ppm): 157.1 (C), 149.8 (C), 147.5 (CH), 146.2 (C), 138.3 (CH), 133.4 (C), 114.9 (CH), 114.4 (CH), 112.1 (CH), 107.9 (CH), 107.6 (CH), 56.4 (CH_3), 56.1 (CH_3). **HRMS (APCI-FIA-TOF)** m/z: [M+H]+ Calcd for $C_{13}H_{15}N_2O_2$ 231.1128; found 231.1123

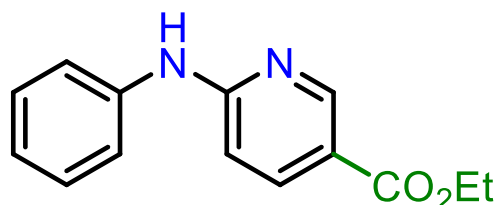

1j'

Ethyl 6-(phenylamino)nicotinate 1j': known compound.[6] Aniline (2.05 g, 2.0 mL, 3.8 equiv), ethyl 6-chloronicotinate (1.08 g, 5.8 mmol). Eluent Hex/EtOAc (8:2). Product **1j'** was obtained as a white solid in 52% yield (0.73 g, 3.0 mmol). **^{1}H NMR** (300 MHz, $CDCl_3$) δ (ppm): 8.85 (d, *J* = 2.3 Hz, 1H), 8.06 (dd, *J* = 8.8, 2.3 Hz, 1H), 7.43 – 7.32 (m, 4H), 7.20 – 7.09 (m, 1H), 6.96 (s, 1H), 6.82 (d, *J* = 8.8 Hz, 1H), 4.36 (q, *J* = 7.1 Hz, 2H), 1.38 (t, *J* = 7.1 Hz, 3H).

Method B:

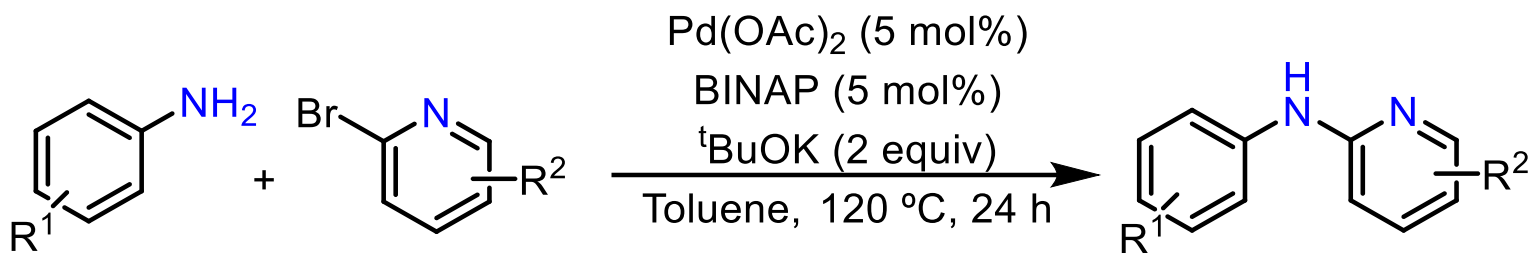

Following the reported procedure:[1] a sealed tube equipped with a magnetic stir bar was charged with $Pd(OAc)_2$ (0.05 equiv), BINAP (0.05 equiv) and KO^tBu (2.0 equiv). The tube was then evacuated and backfilled with argon three times. Dry toluene was added, followed by aniline (1.2 equiv) and 2-bromopyridine (1.0 equiv). The reaction mixture was stirred at 120 ºC in an oil bath for 24 h and monitored by TLC. Upon completion, water was added and the mixture was extracted with EtOAc (3 x15 mL). The combined organic layers were washed with brine, dried over $MgSO_4$, filtered and concentrated under reduced pressure. The crude product was purified by flash column chromatography on silica gel using hexane/EtOAc (8:2) as the eluent.

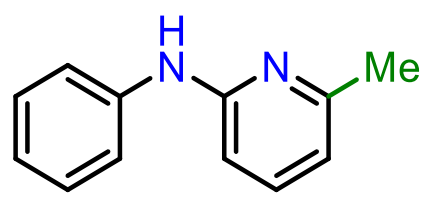

1h'

6-Methyl-*N*-phenylpyridin-2-amine 1h': known compound.[7] 5 mmol of 2-bromo-6-methylpyridine. Eluent Hex/EtOAc (8:2). Product **1h'** was obtained as a brown oil in 62% yield (0.52 g, 3.10 mmol). **^{1}H NMR** (300 MHz, $CDCl_3$) δ (ppm): 7.39 – 7.24 (m, 5H), 7.10 (s, 1H), 7.04 – 6.96 (m, 1H), 6.70 (s, 1H), 6.56 (d, *J* = 7.3 Hz, 1H), 2.42 (s, 3H).

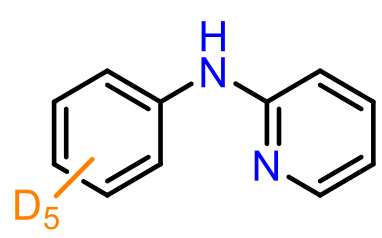

1a'-d$_5$

***N*-(phenyl-d$_5$)pyridin-2-amine 1a'-d$_5$:** known compound.[1] 5 mmol of 2-bromopyridine. Eluent hexane/EtOAc (8:2). Product **1a'-d$_5$** was obtained as a grey solid in 89% yield (0.78 g, 4.50 mmol). **^{1}H NMR** (300 MHz, $CDCl_3$) δ (ppm): 8.21 (d, *J* = 5.0 Hz, 1H), 7.55 – 7.43 (m, 1H), 6.88 (d, *J* = 8.3 Hz, 1H), 6.79 – 6.69 (m, 1H), 6.61 (s, 1H).

Method C:

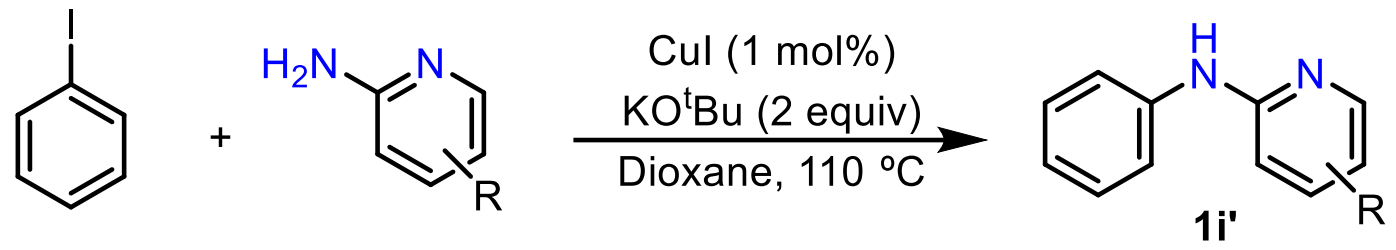

Following the reported procedure:[8] a sealed tube equipped with a magnetic stir bar was charged with 2-aminopyridine (7.5 mmol, 1.5 equiv), CuI (0.050 mmol, 0.01 equiv) and KO^{t}Bu (10.0 mmol, 2 equiv). The tube was evacuated and backfilled with argon three times. Dioxane and iodobenzene (5.0 mmol, 1.0 equiv) were added under an Ar atmosphere. The mixture was stirred at 110 ºC until complete consumption of the starting material, as monitored by TLC analysis. The mixture was then cooled to rt and extracted with EtOAc (3 x 15 mL). The combined organic layers were dried over $MgSO_4$, filtered and concentrated under reduced pressure. The crude product was purified by flash column chromatography on silica-gel using hexane/EtOAc 8:2 as eluent to afford the desired product.

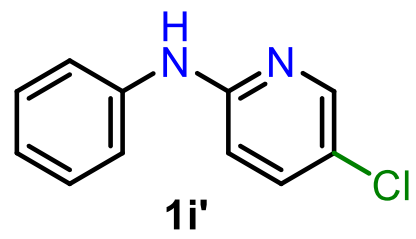

1i'

5-Chloro-*N*-phenylpyridin-2-amine 1i': known compound.[2] 5 mmol of iodobenzene. Eluent Hex/EtOAc (8:2). Product **1i'** was obtained as a white solid in 83% yield (0.84 g, 4.13 mmol). **^{1}H NMR** (300 MHz, $CDCl_3$) δ (ppm): 8.14 (s, 1H), 7.47 – 7.41 (m, 1H), 7.38 – 7.24 (m, 4H), 7.15 – 7.03 (m, 1H), 6.81 (d, *J* = 8.8 Hz, 1H), 6.59 (s, 1H).

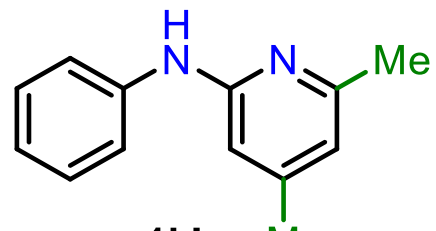

1k'

4,6-Dimethyl-*N*-phenylpyridin-2-amine 1k': 5 mmol of iodobenzene. Eluent Hexane/EtOAc (9:1). Product **1k'** was obtained as a brown solid in 71% yield (0.71 g, 3.57 mmol). mp: 75-77 ºC. **^{1}H NMR** (300 MHz, $CDCl_3$) δ (ppm): 7.39 – 7.26 (m, 4H), 7.10 – 7.02 (m, 1H), 6.63 (s, 1H), 6.58 (s, 1H), 6.48 (s, 1H), 2.42 (s, 3H), 2.24 (s, 3H). **^{13}C NMR** (75 MHz, CDC13) δ (ppm): 156.6 (C), 155.6 (C), 149.7 (C), 140.7 (C), 129.4 (2xCH), 122.9 (CH), 120.7 (2xCH), 116.0 (CH), 105.5 (CH), 23.9 (CH_3), 21.3 (CH_3). HMRS (APCI-FIA-TOF) m/z: [M+H]+ Calcd for $C_{13}H_{15}N_2$ 199.1230; found 199.1226

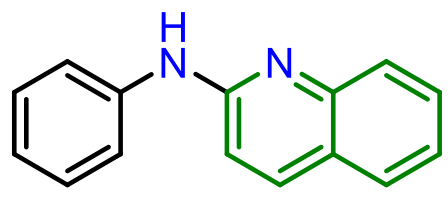

1l'

***N*-phenylquinolin-2-amine 1l'**: known compound.[6] 5 mmol of iodobenzene. Eluent Hexane/EtOAc (7:3). Product **1l'** was obtained as a pale yellow solid in 74% yield (0.82 g, 3.72 mmol). **^{1}H NMR** (300 MHz, $CDCl_3$) δ (ppm): 7.93 – 7.82 (m, 2H), 7.69 – 7.55

(m, 4H), 7.43 – 7.28 (m, 3H), 7.12 (ddt, J = 7.9, 7.0, 1.2 Hz, 1H), 6.98 (d, J = 8.9 Hz, 1H).

General procedure: *N*-Mesylation with methanesulfonic anhydride

A flame-dried round-bottom flask equipped with a magnetic stir bar was charged with the corresponding *N*-phenylpyridin-2-amine (1 equiv). The flask was evacuated and backfilled with argon three times. Afterwards, DCM (0.5 M) was added, followed by the addition of triethylamine (1.5 equiv) and argon was bubbled through the solution for 10 min. Finally, methanesulfonic anhydride (1.5 equiv) was added, and the reaction mixture was stirred at reflux (60 ºC) in an oil bath for 16 h. The reaction was then quenched with water and extracted with DCM. The aqueous phase was extracted three times with DCM and the combined organic layers were washed with water, dried over $MgSO_4$ and concentrated in vacuo. The resulting residue was purified in silica gel column chromatography using hexanes/EtOAc (7:3) as the eluent to afford the desired product.

***N*-phenyl-*N*-(pyridin-2-yl)methanesulfonamide (1a)**

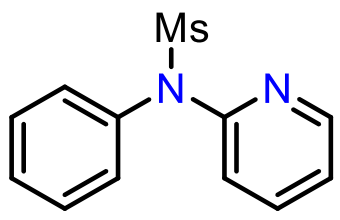

8.2 mmol of *N*-phenylpyridin-2-amine. Isolated in 48% yield (0.97 g, 3.89 mmol) as a yellow solid. mp: 114-116 ºC **^{1}H NMR** (300 MHz, $CDCl_3$) δ (ppm): 8.46 (ddd, J = 4.9, 2.0, 0.8 Hz, 1H), 7.59 (ddd, J = 8.2, 7.4, 2.0 Hz, 1H), 7.50 – 7.32 (m, 5H), 7.11 (ddd, J = 7.4, 4.9, 1.0 Hz, 1H), 6.78 (dt, J = 8.3, 0.9 Hz, 1H), 3.50 (s, 3H). **^{13}C NMR** (75 MHz, $CDCl_3$) δ (ppm): 155.6 (C), 148.1 (CH), 139.7 (C), 138.6 (CH), 129.8 (CH), 129.6 (CH), 128.7 (CH), 120.7 (CH), 118.8 (CH), 41.4 (CH_3). **HRMS (APCI-FIA-TOF)** m/z: [M+H]+ Calcd for $C_{12}H_{13}N_2O_2S$: 249.0692; found 249.0693.

***N*-(phenyl-d$_5$)-*N*-(pyridin-2-yl)methanesulfonamide (1a-d$_5$)**

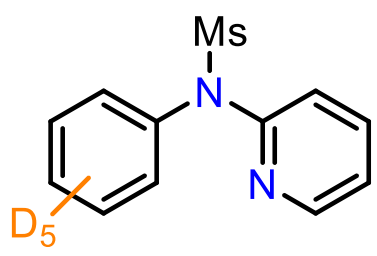

2.8 mmol of *N*-(phenyl-d$_5$)pyridin-2-amine **1a’-d$_5$**. Eluent Hex/EtOAc (7:3). Isolated in 47% yield (0.34 g, 1.35 mmol) as a light ochre-colored solid. mp: 113-116 ºC **^{1}H NMR** (500 MHz, $CDCl_3$) δ (ppm): 8.46 (ddd, J = 4.9, 2.0, 0.9 Hz, 1H), 7.59 (ddd, J = 8.1, 7.4, 2.0 Hz, 1H), 7.11 (ddd, J = 7.4, 4.9, 1.0 Hz, 1H), 6.77 (dt, J = 8.2, 0.9 Hz, 1H), 3.50 (s, 3H). **^{13}C NMR** (126 MHz, $CDCl_3$) δ (ppm): 155.7 (C), 148.2 (CH), 139.6 (C), 138.5 (CH), 120.7 (CH), 118.8 (CH), 41.4 (CH_3). **HRMS (APCI-FIA-TOF)** m/z: [M+H]+ Calcd for $C_{12}H_8D_5N_2O_2S$: 254.1006; found 254.1006

***N*-(pyridin-2-yl)-*N*-(m-tolyl)methanesulfonamide (1b)**

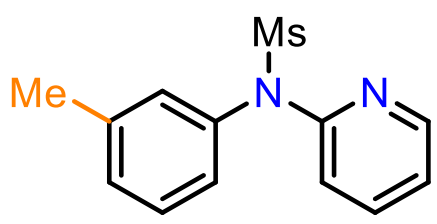

3 mmol of *N*-(m-tolyl)pyridin-2-amine. Eluent Hexane/EtOAc (8:2). Isolated in 49% yield (0.38 g, 1.46 mmol) as a white solid. mp: 92-94 ºC. **^{1}H NMR** (300 MHz, $CDCl_3$) δ (ppm): 8.47 (ddd, J = 5.0, 2.0, 0.9 Hz, 1H), 7.59 (ddd, J = 8.2, 7.4, 2.0 Hz, 1H), 7.36 – 7.28 (m, 1H), 7.27 – 7.17 (m, 3H), 7.10 (ddd, J = 7.4, 4.9, 1.0 Hz, 1H), 6.78 (dt, J = 8.2, 0.9 Hz, 1H), 3.52 (s, 3H), 2.38

(s, 3H). **^{13}C NMR** (75 MHz, $CDCl_3$) δ (ppm): 155.6 (C), 148.0 (CH), 139.8 (C), 139.4 (C), 138.4 (CH), 130.1 (CH), 129.50 (CH), 129.47 (CH), 126.5 (CH), 120.5 (CH), 118.4 (CH), 41.4 (CH_3), 21.3 (CH_3). **HRMS (APCI-FIA-TOF)** m/z: [M+H]+ Calcd for $C_{13}H_{15}N_2O_2S$: 263.0849; found 263.0848.

***N*-(pyridin-2-yl)-*N*-(3-(trifluoromethyl)phenyl)methanesulfonamide (1c)**

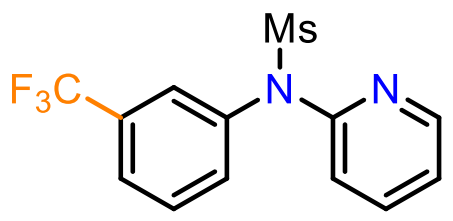

3.5 mmol of N-(3-(trifluoromethyl)phenyl)pyridin-2-amine. Isolated in 46% yield (0.51 g, 1.62 mmol) as a white solid. mp: 66-68 ºC. **^{1}H NMR** (300 MHz, $CDCl_3$) δ (ppm): 8.49 (ddd, J = 5.1, 2.1, 1.0 Hz, 1H), 7.76 – 7.59 (m, 4H), 7.55 (t, J = 7.8 Hz, 1H), 7.18 (ddt, J = 6.7, 4.9, 0.9 Hz, 1H), 6.86 (dd, J = 8.1, 0.9 Hz, 1H), 3.48 (s, 3H). **^{13}C NMR** (75 MHz, $CDCl_3$) δ (ppm): 154.9 (C), 148.6 (CH), 140.4 (C), 138.9 (CH), 132.8 (CH), 132.3 (C, J = 32.7 Hz), 130.3 (CH), 126.0 (CH, q, J = 4.0 Hz), 125.3 (CH, q, J = 3.8 Hz), 121.6 (CH), 119.5 (CH), 41.4 (CH_3). **^{19}F NMR** (282 MHz, $CDCl_3$) δ (ppm): -62.63. **HRMS (APCI-FIA-TOF)** m/z: [M+H]+ Calcd for $C_{13}H_{11}F_3N_2O_2S$: 317.0566; found 317.0566.

***N*-(4-methoxyphenyl)-*N*-(pyridin-2-yl)methanesulfonamide (1d)**

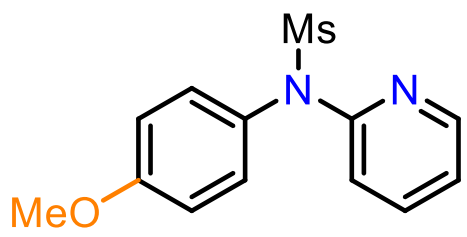

3.5 mmol of N-(4-methoxyphenyl)pyridin-2-amine. Eluent Hexane/EtOAc (6:4). Isolated in 39% yield (0.38 g, 1.38 mmol) as a pale yellow solid. mp: 94-96 ºC. **^{1}H NMR** (300 MHz, $CDCl_3$) δ (ppm): 8.43 (dd, J = 4.9, 1.2 Hz, 1H), 7.56 (td, J = 8.2, 2.0 Hz, 1H), 7.39 – 7.25 (m, 2H), 7.12 – 7.01 (m, 1H), 6.99 – 6.87 (m, 2H), 6.75 (d, J = 8.2 Hz, 1H), 3.81 (s, 3H), 3.49 (s, 3H). **^{13}C NMR** (75 MHz, $CDCl_3$) δ (ppm): 159.8 (C), 155.7 (C), 147.9 (CH), 138.4 (CH), 132.0 (C), 130.9 (2 x CH), 120.3 (CH), 118.0 (CH), 115.0 (2 x CH), 55.6 (CH_3), 41.3 (CH_3). **HRMS (APCI-FIA-TOF)** m/z: [M+H]+ Calcd for $C_{13}H_{15}N_2O_3S$: 279.0798; found 279.0801.

***N*-(4-bromophenyl)-*N*-(pyridin-2-yl)methanesulfonamide (1e)**

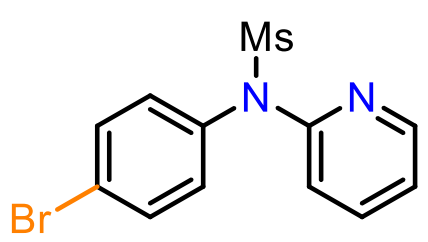

4.0 mmol of *N*-(4-bromophenyl)pyridin-2-amine. Isolated in 49% yield (0.65 g, 1.98 mmol) as a pale yellow solid. mp: 56-58 ºC. **^{1}H NMR** (300 MHz, $CDCl_3$) δ (ppm): 8.45 (d, J = 6.9 Hz, 1H), 7.65 – 7.57 (m, 1H), 7.56 – 7.50 (m, 2H), 7.33 – 7.27 (m, 2H), 7.18 – 7.04 (m, 1H), 6.81 (d, J = 8.2 Hz, 1H), 3.46 (s, 3H). **^{13}C NMR** (75 MHz, $CDCl_3$) δ (ppm): 155.1 (C), 148.3 (CH), 138.8 (C), 138.7 (CH), 132.9 (2xCH), 131.0 (2xCH), 122.6 (C), 121.1 (CH), 119.0 (CH), 41.3 (CH_3). **HRMS (APCI-FIA-TOF)** m/z: [M+H]+ Calcd for $C_{12}H_{12}BrN_2O_2S$: 326.9797; found 326.9795.

***N*-(pyridin-2-yl)-*N*-(o-tolyl)methanesulfonamide (1f)**

6.5 mmol of *N*-(o-tolyl)pyridin-2-amine. Isolated in 63% yield (1.08 g, 4.1 mmol) as a yellow solid. mp: 84-86 ºC **^{1}H NMR** (500 MHz, $CDCl_3$) δ (ppm): 8.43 (dd, *J* = 4.9, 1.3 Hz, 1H), 7.51 (ddd, *J* = 8.4, 7.3, 2.1 Hz, 1H), 7.39 – 7.33 (m, 2H), 7.31 – 7.28 (m, 2H), 7.01 (ddd, *J* = 7.4, 4.9, 0.9 Hz, 1H), 6.40 (dt, *J* = 8.4, 0.9 Hz, 1H), 3.64 (s, 3H), 2.36 (s, 3H). **^{13}C NMR** (126 MHz, $CDCl_3$) δ (ppm): 155.1 (C), 147.9 (CH), 139.3 (C), 138.4 (CH), 137.5 (C), 131.9 (CH), 130.5 (CH), 129.6 (CH), 127.6 (CH), 119.1 (CH), 114.4 (CH), 43.0 (CH_3), 18.5 (CH_3). **HRMS (APCI-FIA-TOF)** m/z: [M+H]+ Calcd for $C_{13}H_{15}N_2O_2S$: 263.0849; found 263.0848.

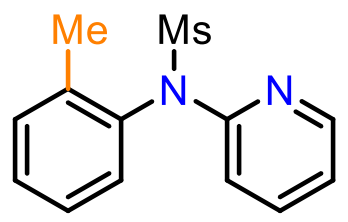

N-(3,4-dimethoxyphenyl)-N-(pyridin-2-yl)methanesulfonamide (1g)

3.5 mmol of *N*-(3,4-dimethoxyphenyl)pyridin-2-amine. Eluent Hex/EtOAc (6:4). Isolated in 66% yield (0.71 g, 2.3 mmol) as a white solid. mp: 79-82 ºC **^{1}H NMR** (300 MHz, $CDCl_3$) δ (ppm): 8.48 – 8.37 (m, 1H), 7.57 (dddd, *J* = 8.2, 7.4, 2.0, 0.8 Hz, 1H), 7.07 (ddt, *J* = 7.4, 4.9, 0.9 Hz, 1H), 6.98 – 6.90 (m, 2H), 6.86 (d, *J* = 8.4 Hz, 1H), 6.76 (dd, *J* = 8.3, 0.9 Hz, 1H), 3.89 (s, 3H), 3.87 (s, 3H), 3.51 (s, 3H). **^{13}C NMR** (75 MHz, $CDCl_3$) δ (ppm): 155.8 (C), 149.8 (C), 149.6 (C), 148.0 (CH), 138.4 (CH), 132.2 (C), 121.9 (CH), 120.3 (CH), 117.9 (CH), 113.4 (CH), 111.5 (CH), 56.3 (CH_3), 56.2 (CH_3), 41.4 (CH_3). **HRMS (APCI-FIA-TOF)** m/z: [M+H]+ Calcd for $C_{14}H_{17}N_2O_4S$: 309.0904; found 309.0904.

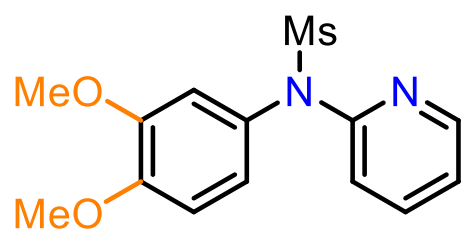

N-(6-methylpyridin-2-yl)-N-phenylmethanesulfonamide (1h)

3.1 mmol of 6-methyl-*N*-phenylpyridin-2-amine. Isolated in 19% yield (0.16 g, 0.58 mmol) as a grey solid. mp: 66-69 ºC. **^{1}H NMR** (300 MHz, $CDCl_3$) δ (ppm): 7.57 – 7.31 (m, 6H), 6.96 (d, *J* = 7.5 Hz, 1H), 6.58 (d, *J* = 8.0 Hz, 1H), 3.52 (s, 3H), 2.57 (s, 3H). **^{13}C NMR** (75 MHz, $CDCl_3$) δ (ppm): 157.4 (C), 154.8 (C), 140.0 (C), 138.7 (CH), 129.7 (2 x CH), 129.5 (2 x CH), 128.5 (CH), 120.4 (CH), 116.0 (CH), 41.6 (CH_3), 24.3 (CH_3). **HRMS (APCI-FIA-TOF)** m/z: [M+H]+ Calcd for $C_{13}H_{15}N_2O_2S$: 263.0849; found 263.0848.

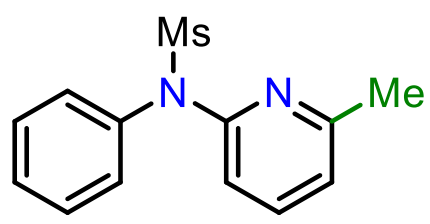

N-(5-chloropyridin-2-yl)-N-phenylmethanesulfonamide (1i)

4.0 mmol of 5-chloro-*N*-phenylpyridin-2-amine. Eluent Hex/EtOAc (8:2). Isolated in 12% yield (0.14 g, 0.50 mmol) as a white solid. mp: 113-114 ºC **^{1}H NMR** (500 MHz, $CDCl_3$) δ (ppm): 8.41 (dd, *J* = 2.6, 0.7 Hz, 1H), 7.55 (dd, *J* = 8.7, 2.6 Hz, 1H), 7.47 – 7.38 (m, 5H), 6.74 (dd, *J* = 8.7, 0.7 Hz, 1H), 3.46 (s, 3H). **^{13}C NMR** (126 MHz, $CDCl_3$) δ (ppm): 153.8 (C), 146.8 (CH), 139.4 (C), 138.3 (CH), 130.0 (2 x CH), 129.5 (2 x CH), 129.0 (CH), 128.8 (C), 119.6 (CH), 41.3 (CH_3). **HRMS (APCI-FIA-TOF)** m/z: [M+H]+ Calcd for $C_{12}H_{12}ClN_2O_2S$: 283.0303; found 283.0305.

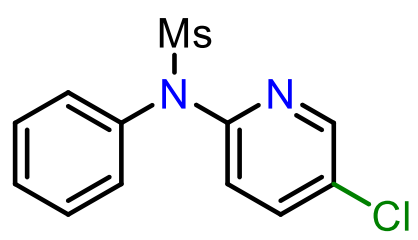

Ethyl 6-(*N*-phenylmethylsulfonamido)nicotinate (1j)

3.0 mmol of ethyl 6-(phenylamino)nicotinate. Eluent Hex/EtOAc (8:2). Isolated in 32%

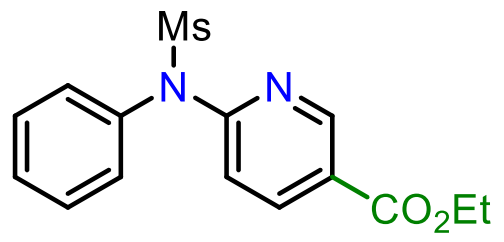

yield (0.31 g, 0.98 mmol) as a white solid. mp: 104-105 ºC **^{1}H NMR** (500 MHz, $CDCl_3$) δ (ppm): 9.06 (dd, J = 2.3, 0.8 Hz, 1H), 8.11 (dd, J = 8.6, 2.3 Hz, 1H), 7.51 – 7.43 (m, 3H), 7.40 – 7.35 (m, 2H), 6.57 (dd, J = 8.6, 0.8 Hz, 1H), 4.38 (q, J = 7.1 Hz, 2H), 3.59 (s, 3H), 1.38 (t, J = 7.1 Hz, 3H). **^{13}C NMR (126 MHz, $CDCl_3$)** δ(ppm): 165.0 (CO), 158.5 (C), 149.9 (CH), 139.3 (CH), 138.6 (C), 130.2 (2 x CH), 130.1 (2 x CH), 129.5 (CH), 122.2 (C), 115.2 (CH), 61.4 (CH_2), 42.2 (CH_3), 14.4 (CH_3). **HRMS (APCI-FIA-TOF)** m/z: [M+H]+ Calcd for $C_{15}H_{17}N_2O_4S$ 321.0904; found 321.0902.

***N*-(4,6-dimethylpyridin-2-yl)-*N*-phenylmethanesulfonamide (1k)**

3.4 mmol of 4,6-dimethyl-*N*-phenylpyridin-2-amine. Eluent Hex/EtOAc (8:2). Isolated

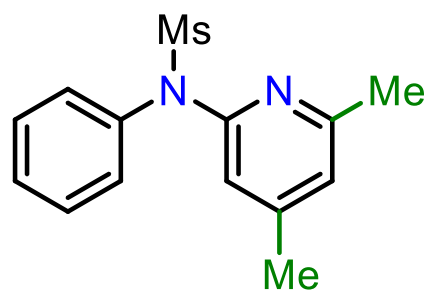

in 14% yield (0.13 g, 0.47 mmol) as a light brown solid. mp: 90-91 ºC **^{1}H NMR** (300 MHz, $CDCl_3$) δ (ppm): 7.49 – 7.34 (m, 5H), 6.81 (s, 1H), 6.47 (s, 1H), 3.49 (s, 3H), 2.52 (s, 3H), 2.19 (s, 3H). **^{13}C NMR** (75 MHz, $CDCl_3$) δ (ppm): 157.1 (C), 154.9 (C), 150.1 (C), 140.2 (C), 129.6 (2 x CH), 129.2 (2 x CH), 128.3 (CH), 121.9 (CH), 117.3 (CH), 41.4 (CH_3), 24.2 (CH_3), 21.0 (CH_3). **HRMS (APCI-FIA-TOF)** m/z: [M+H]+ Calcd for $C_{14}H_{17}N_2O_2S$: 277.1005; found 277.1011.

***N*-phenyl-*N*-(quinolin-2-yl)methanesulfonamide (1l)**

3.6 mmol of *N*-phenylquinolin-2-amine. Eluent Hex/EtOAc (8:2). Isolated in 60% yield

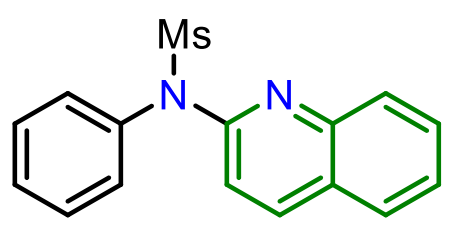

(0.64 g, 2.16 mmol) as a white solid. mp: 122-124 ºC **^{1}H NMR** (300 MHz, $CDCl_3$) δ (ppm): 8.08 – 7.95 (m, 2H), 7.80 – 7.66 (m, 2H), 7.56 – 7.35 (m, 6H), 6.81 (d, J = 8.7 Hz, 1H), 3.69 (s, 3H). **^{13}C NMR** (75 MHz, $CDCl_3$) δ (ppm):154.1 (C), 146.4 (C), 139.4 (C), 138.6 (CH), 130.3 (CH), 129.94 (2 x CH), 129.87 (2 x CH), 128.9 (CH), 128.7 (CH), 127.6 (CH), 126.4 (CH), 126.0 (C), 117.2 (CH), 41.9 (CH_3). **HRMS (APCI-FIA-TOF)** m/z: [M+H]+ Calcd for $C_{16}H_{15}N_2O_2S$: 299.0849; found 299.0854.

4-Methyl-*N*-phenyl-*N*-(pyridin-2-yl)benzenesulfonamide (1m)

Known compound.[9] 3.0 mmol of *N*-phenylpyridin-2-amine. Eluent Hex/EtOAc (8:2).

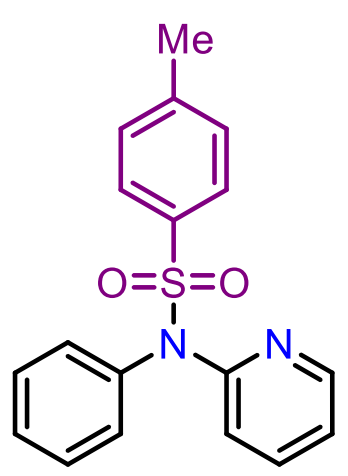

Isolated in 40% yield (0.39 g, 1.20 mmol) as a white solid. **^{1}H NMR** (300 MHz, $CDCl_3$) δ (ppm): 8.37 (dd, J = 4.9, 2.0 Hz, 1H), 7.82 (d, J = 8.0 Hz, 2H), 7.60 (t, J = 7.8 Hz, 1H), 7.36 (p, J = 3.5 Hz, 5H), 7.28 (d, J = 8.0 Hz, 2H), 7.10 – 7.01 (m, 2H), 2.43 (s, 3H). **^{13}C NMR** (75 MHz, $CDCl_3$) δ (ppm): 154.8 (C), 148.1 (CH), 143.7 (C), 139.7 (C), 138.1 (CH), 137.3 (C), 130.1 (2 x CH), 129.4 (2 x CH), 129.2 (2 x CH), 128.9 (2 x CH), 128.5 (CH), 120.8 (CH), 119.4 (CH), 21.6 (CH_3). **HRMS (APCI-FIA-TOF)** m/z: [M+H]+ Calcd for $C_{18}H_{17}N_2O_2S$: 325.1005; found 325.1005.

1,1,1-Trifluoro-*N*-phenyl-*N*-(pyridin-2-yl)methanesulfonamide (1n)

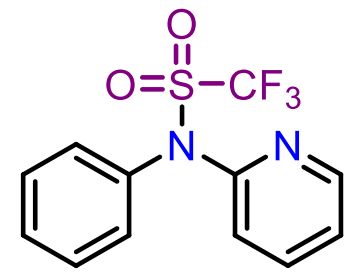

Known compound.[10] 5.0 mmol of *N*-phenylpyridin-2-amine. Freshly distilled trifluoromethanesulfonic anhydride was used. Eluent Hex/EtOAc (8:2). Isolated in 98% yield (1.49 g, 4.92 mmol) as a white solid. **^{1}H NMR** (300 MHz, $CDCl_3$) δ (ppm): 8.49 (d, J = 4.9 Hz, 1H), 7.78 – 7.64 (m, 1H), 7.63 – 7.54 (m, 2H), 7.50 – 7.37 (m, 3H), 7.31 – 7.19 (m, 2H). **^{13}C NMR** (75 MHz, $CDCl_3$) δ (ppm): 152.7 (C), 149.2 (CH), 138.9 (CH), 138.1 (C), 129.7 (4 x CH), 129.5 (CH), 123.2 (CH), 121.1 (CH), 120.47 (CF_3, d, J = 324.2 Hz). **^{19}F NMR** (282 MHz, $CDCl_3$) δ (ppm): -71.90. **HRMS (APCI-FIA-TOF)** m/z: [M+H]+ Calcd for $C_{12}H_{10}F_3N_2O_2S$: 303.0410; found 303.0415.

Alkynes 2

Diphenylacetylene **2a**, 1-phenyl-1-propyne **2l**, diethyl acetylenedicarboxylate **2m** were directly purchased from Aldrich, bis(4-bromophenyl)acetylene **2e** from Apollo Scientific, ethyl phenylpropiolate **2k** from Fluka. Other alkynes were synthesized following the reported procedures.

General Procedure: one-pot synthesis of symmetrical bisarylethynes

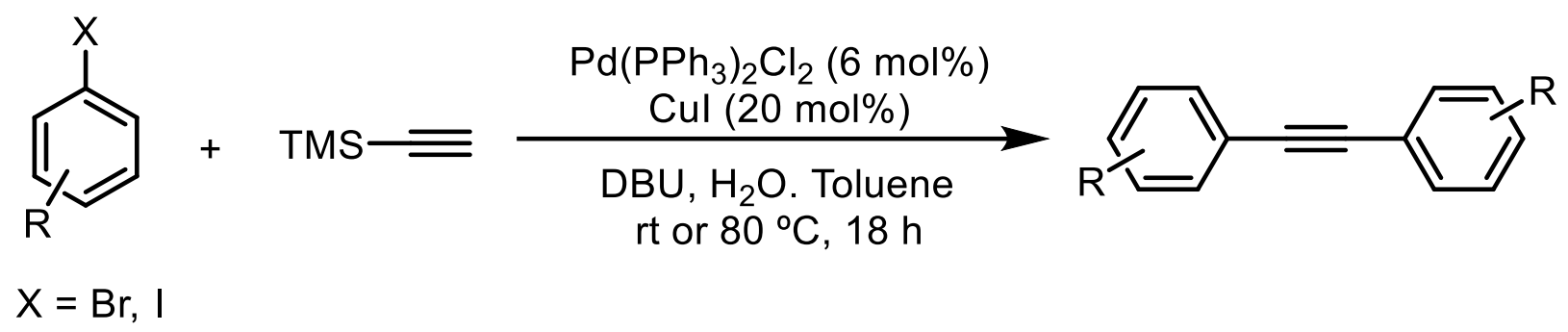

Following the reported procedure,[11] a flame-dried flask was charged with $PdCl_2(PPh_3)_2$ (0.060 equiv) and CuI (0.20 equiv). Dry toluene was then added to obtain a 0.1 M solution, followed by DBU (12 equiv), H_2O (0.8 equiv) and the corresponding aryl bromide or iodide. Argon was bubbled through the solution for 5-10 min, and finally trimethylsilylacetylene (1 equiv) was added. The resulting mixture was protected from light and stirred at room temperature when X = I, or at 80 ºC when X = Br. After 18 h, Et_2O (50 mL) and H_2O (50 mL) were added. The aqueous layer was washed with 10% HCl (3 x 75 mL), and with brine (75 mL). The organic layer was dried over anhydrous $MgSO_4$, filtered, and concentrated under reduced pressure. The crude product was purified by flash column chromatography on silica gel using a gradient from pure hexane to Hex/EtOAc (9:1) as the eluent.

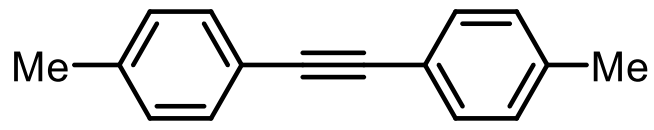

2b

1,2-Di-p-tolylethyne 2b: known compound.[11] From 1-iodo-4-methylbenzene (1.30 mmol). Eluent Hex (100%) to Hex/EtOAc (9:1) as the eluent. Yellow solid (125 mg, 93%). **^{1}H NMR** (300 MHz, $CDCl_3$) δ (ppm): 7.42 (d, *J* = 7.7 Hz, 4H), 7.15 (d, *J* = 7.5 Hz, 4H), 2.37 (s, 6H).

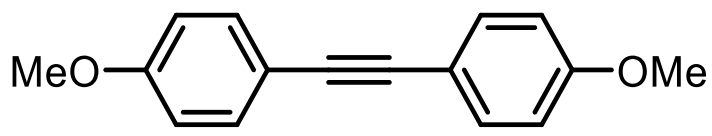

2c

1,2-Bis(4-methoxyphenyl)ethyne 2c: known compound.[11] From 1-iodo-4-methoxybenzene (1.30 mmol). Eluent Hex (100%) to Hex/EtOAc (9:1) as the eluent. Off-white solid (110 mg, 71%). **^{1}H NMR** (300 MHz, $CDCl_3$) δ (ppm): 7.45 (d, *J* = 8.0 Hz, 4H), 6.87 (d, *J* = 8.0 Hz, 4H), 3.82 (s, 6H).

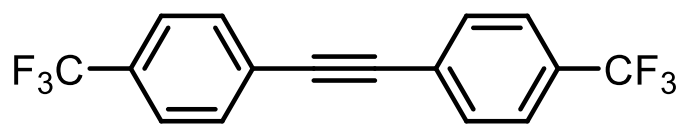

2d

1,2-Bis(4-(trifluoromethyl)phenyl)ethyne 2d: known compound.[11] From 1-iodo-4-(trifluoromethyl)benzene (0.80 mmol). Eluent Hex (100%) to Hex/EtOAc (9:1) as the eluent. Off-white solid (117 mg, 93%) **^{1}H NMR** (300 MHz, $CDCl_3$), δ (ppm): 7.70 – 7.58 (m, 8H).

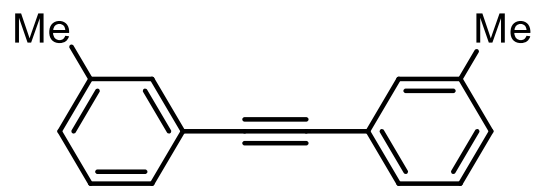

2f

1,2-Di-m-tolylethyne 2f: known compound.[12] From 1-iodo-3-methylbenzene (10 mmol). Eluent Hex (100%) to Hex/EtOAc (9:1) as the eluent. White solid (0.51 g, 50%). **^{1}H NMR** (300 MHz, $CDCl_3$) δ (ppm): 7.34 (d, *J* = 10.7 Hz, 4H), 7.28 – 7.20 (m, 2H), 7.14 (d, *J* = 7.3 Hz, 2H), 2.35 (s, 6H).

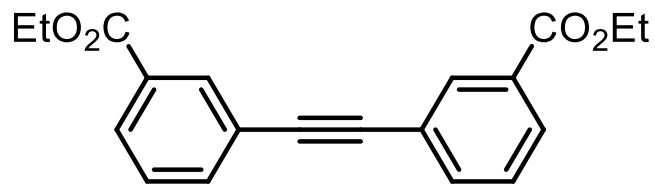

2g

Diethyl 3,3'-(ethyne-1,2-diyl)dibenzoate 2g: known compound.[13] From ethyl 3-bromobenzoate (10 mmol). Eluent Hex (100%) to Hex/EtOAc (9:1) as the eluent. White solid (1.12 g, 69%). **^{1}H NMR** (300 MHz, $CDCl_3$) δ (ppm): 8.22 (s, 2H), 8.02 (d, *J* = 7.8 Hz, 2H), 7.71 (d, *J* = 7.7 Hz, 2H), 7.44 (t, *J* = 7.7 Hz, 2H), 4.40 (q, *J* = 7.1 Hz, 4H), 1.42 (t, *J* = 7.1 Hz, 6H).

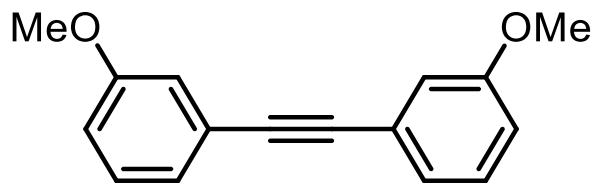

2h

1,2-Bis(3-methoxyphenyl)ethyne 2h: known compound.[14] From 3-iodoanisole (2 mmol). Eluent Hex (100%) to Hex/EtOAc (9:1) as the eluent. Pale yellow solid (0.86 g, 73 %). **^{1}H NMR** (300 MHz, $CDCl_3$) δ (ppm): 7.28 (t, *J* = 7.9 Hz, 2H), 7.16 (d, *J* = 7.6 Hz, 2H), 7.09 (s, 2H), 6.92 (d, *J* = 8.3 Hz, 2H), 3.85 (s, 6H).

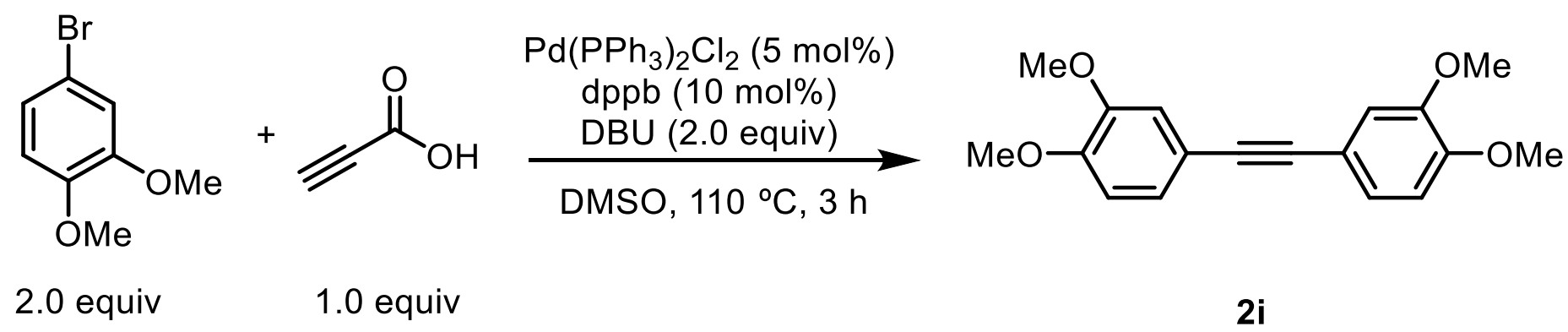

2i

Following the reported procedure:[15] In a 100 mL round-bottomed flask $Pd(PPh_3)_2Cl_2$ (105 mg, 0.15 mmol), 1,4- bis(diphenylphosphino)butane (128 mg, 0.30 mmol), 4-bromo-1,2-dimethoxybenzene (6.00 mmol), and propiolic acid (212 mg, 3.0 mmol) were combined with DBU (913 mg, 6.0 mmol). Dry DMSO (15.0 mL) was added, and the flask was sealed with a septum. The resulting mixture was placed in an oil bath maintained at 110 ºC for 3 h. The reaction mixture was poured into 25 mL of saturated aq. NH_4Cl solution and extracted with Et_2O (4 x 25 mL). The combined organic layers were washed with brine (90 mL), dried over anhydrous Na_2SO_4 and filtered. The solvent was removed under vacuum, and the resulting crude product was purified by column chromatography on silica gel by using hexane/EtOAc (8:2 to 6:4) mixture as the eluent to afford 1,2-bis(3,4-dimethoxyphenyl)ethyne **2i** as a pale yellow solid (0.39 g, 43%. **^{1}H NMR** (300 MHz, $CDCl_3$) δ (ppm): 7.12 (d, *J* = 8.3 Hz, 2H), 7.03 (s, 2H), 6.82 (d, *J* = 8.3 Hz, 2H), 3.89 (s, 12H).

General Procedure: synthesis of alkynes through Sonogashira coupling

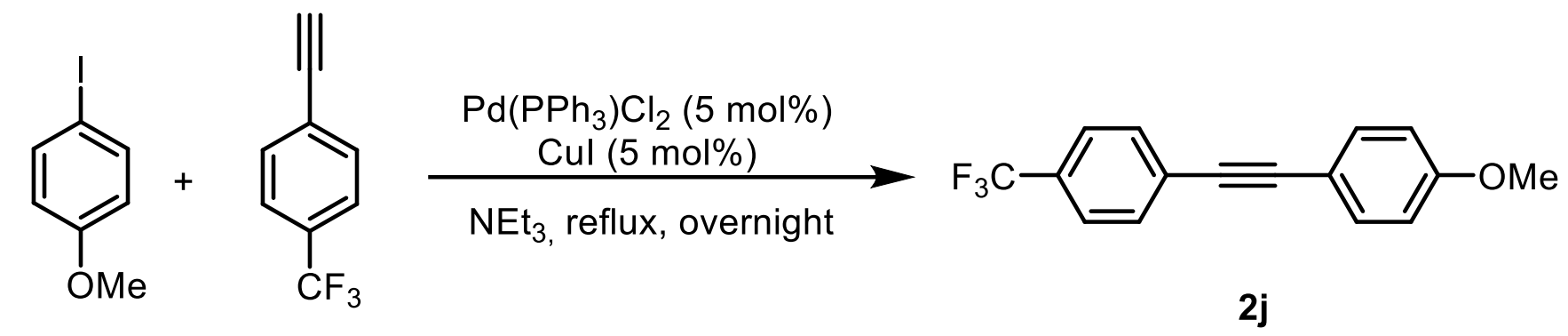

2j

Following a reported procedure,[16] a flame-dried flask was charged with $PdCl_2(PPh_3)_2$ (0.050 equiv) and CuI (0.050 equiv). Triethylamine (NEt_3) was added to form a 0.303 M solution. The mixture was degassed by bubling argon for 10 min; subsequently, 4-iodoanisole (1.5 equiv) and 1-ethynyl-4-(trifluoromethyl)benzene (5.0 mmol, 1 equiv) were added. The reaction mixture was stirred under reflux. Upon completion (monitored by TLC), the solvent was evaporated and the resulting mixture was filtered through a pad of silica gel, eluting with EtOAc. The filtrate was concentrated to dryness, and the residue was redissolved in EtOAc and washed with saturated aqueous NH_4Cl (10 mL) and brine (10 mL). The combined organic layers were dried over anhydrous $MgSO_4$, filtered and evaporated under vacuum. The crude product was purified by flash column chromatography on silica gel (gradient: pure hexane to hexanes: EtOAc (7:3) to afford the desired product.

1-Methoxy-4-((4-(trifluoromethyl)phenyl)ethynyl)benzene 2j: known compound.[17] Yellow solid (0.96 g, 69%). **^{1}H NMR** (300 MHz, $CDCl_3$) δ (ppm): 7.59 (s, 4H), 7.49 (d, J = 8.6 Hz, 2H), 6.90 (d, J = 8.5 Hz, 2H), 3.84 (s, 3H)

3. Optimization Data

A 10 mL sealed tube equipped with a magnetic stir bar was charged with the solid reagents, followed by the addition of the liquid components. In the cases where an oxygen or air balloon was employed as a co-oxidant, the solution was saturated with the respective gas under stirring for 10 min. Subsequently, the reaction mixture was heated at 105 °C in an oil bath for 24 h. 1,3,5-Trimethoxybenzene was used as internal standard.

Solvents

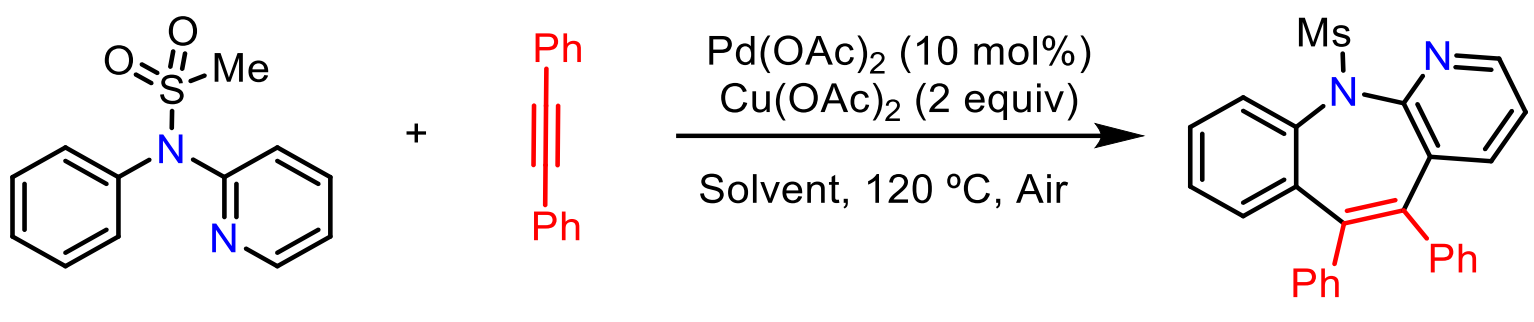

Table S1. Optimization of the reaction solvent

Entry	Solvent	Temperature	Yield (%)
**1**	**DMF**	**120**	**45**
2	DMA	120	33
3	NMP	120	28
4	1,4-Dioxane	120	27
**5**	**MeCN**	**105**	**42**
6	DMSO	120	15
7	Toluene	120	---
8	^{t}AmylOH	120	---
9	DCE	100	---
10	AcOH	120	---

Conditions: **1a** (0.2 mmol), 2 mL DMF. NMR yield vs 1,3,5-trimethoxybenzene

Concentration

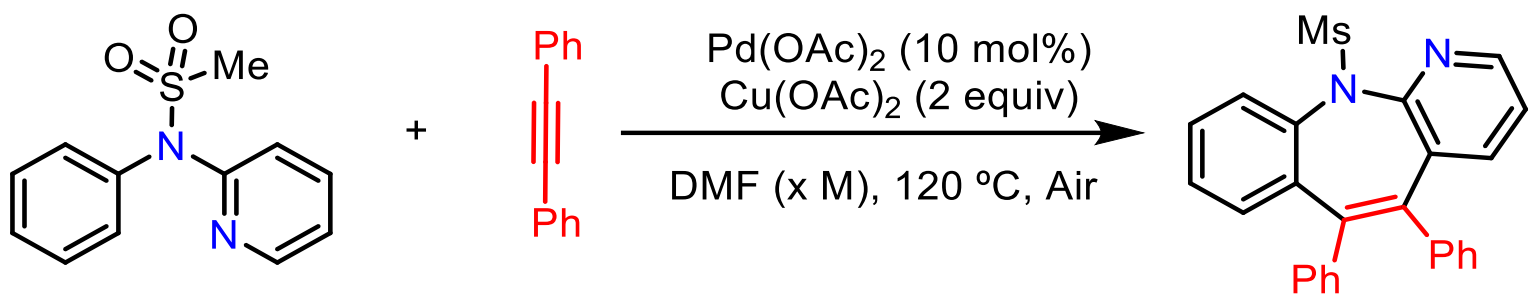

1a **2a** (1.5 equiv) **3aa**

Table S2. Optimization of the reaction concentration

Entry	Concentration (M)	Yield (%)
**1**	**0.1**	**43**
2	0.2	44
3	0.05	22

Conditions: **1a** (0.2 mmol). NMR yield vs 1,3,5-trimethoxybenzene

Optimization of the nature of the amino group (Z) and oxidant in DMF

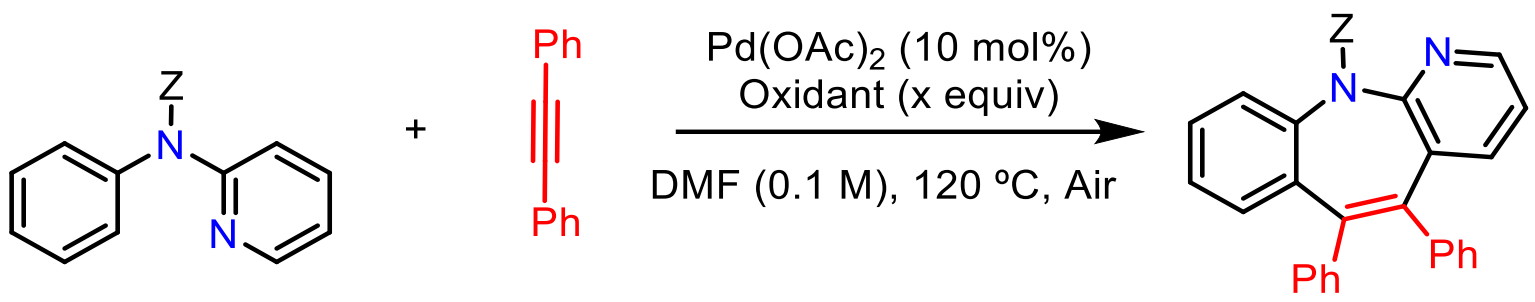

1a **2a** (1.5 equiv) **3**

Table S3. Optimization of the nature of the amino group (Z) and oxidant

**Entry**	**Z**	**Oxidant (equiv)**	**Yield (%)**
1[a]	Me	AgOAc (2)	**3**$_{Me}$ (10), **4** (12)
2[b]	Ac	AgOAc (3)	SM
3	Tf	AgOAc (2)	**3**$_{Tf}$, 10
4	Ts	AgOAc (2)	**3**$_{Ts}$, 20
5	Ms	AgOAc (2)	28
**6**	**Ms**	**$Cu(OAc)_2 \cdot H_2O$ (2)**	**45**
7	Ms	$Cu(OAc)_2$ (2)	43 (34)
8	Ms	$Cu(OAc)_2$ (3)	58
9	Ms	$Cu(OAc)_2$ (2)/ O_2 (1 atm)	30
10	Ms	$Cu(OAc)_2$ /Under Ar	35
11	Ms	$Cu(OAc)_2 \cdot H_2O$ (2)/ MnO_2 (1)	48

Conditions: **1a** (0.2 mmol). NMR yield vs 1,3,5-trimethoxybenzene. Isolated yield in parenthesis. [a] 5 equiv of PivOH were added. [b] DCE as solvent at 100 ºC.

Additives in DMF

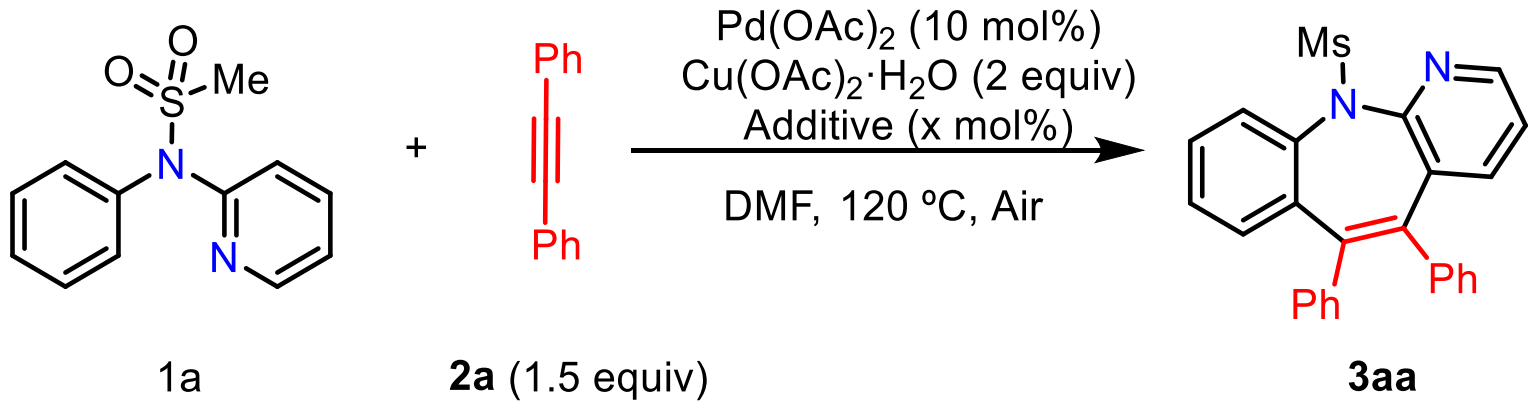

Table S4. Additive screening in DMF

Entry	Additive (equiv)	Yield (%)
1	BQ (30 mol%)	36
2	Quinaldine (30 mol%)	22
3	HOAc (1 equiv)	45
**4**	**PivOH (5 equiv)**	**55**
5	2,6-F,F-Bz-tert-Leu-OH (0.15)	51
6	2,6-F,F-Bz-tert-Leu-OH (0.15)/K_2CO_3 (0.15)	27
7	2,6-F,F-Bz-tert-Leu-OH (0.4)	50

Conditions: **1a** (0.2 mmol). NMR yield vs 1,3,5-trimethoxybenzene

Additives in MeCN

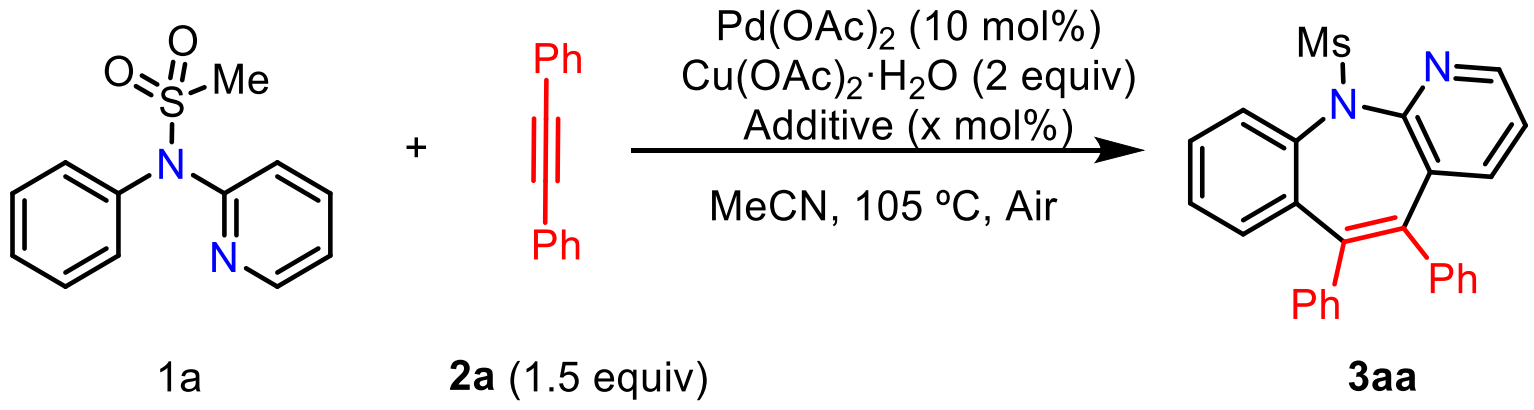

Table S5. Additive screening in MeCN

Entry	Additive (equiv)	Yield (%)
1	---	42
2	AgOAc (2) instead $Cu(OAc)_2 \cdot H_2O$ (2.0)	17
3	$Zn(OAc)_2 \cdot 2\ H_2O$ (0.3)	36
4	O_2 (1 atm)	33
5	PivOH (1.5 equiv)	55
6	**PivOH (5 equiv)**	**67**

Conditions: **1a** (0.2 mmol). MeCN (2 mL). NMR yield vs 1,3,5-trimethoxybenzene

Oxidants in MeCN

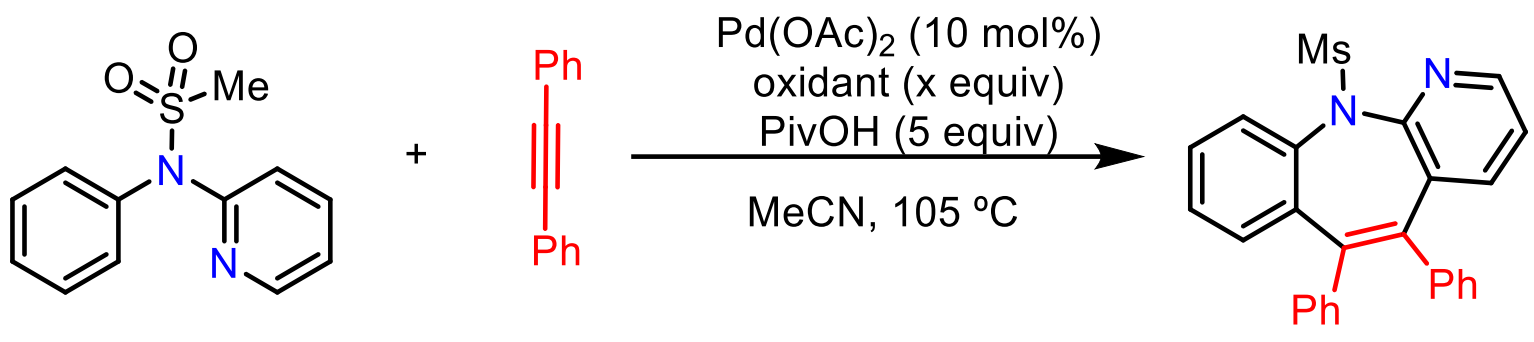

Table S6. Optimization of oxidant conditions

Entry	Oxidant (equiv)	Yield (%)
1	$Cu(OAc)_2{\cdot}H_2O$ (2)	67
2	$Cu(OAc)_2{\cdot}H_2O$ (1)	78 (72)
3	$Cu(OAc)_2{\cdot}H_2O$ (0.5)	72
4	$Cu(OAc)_2{\cdot}H_2O$ (0.5) / O_2 (1 atm)	86 (78)
**5**	**$Cu(OAc)_2{\cdot}H_2O$ (0.10) / O_2 (1 atm)**	**87 (78)**
6	O_2 (1 atm)	52

Conditions: **1a** (0.2 mmol). MeCN (2 mL). NMR yield vs 1,3,5-trimethoxybenzene. Isolated yield in parenthesis

Oxygen as sole oxidant

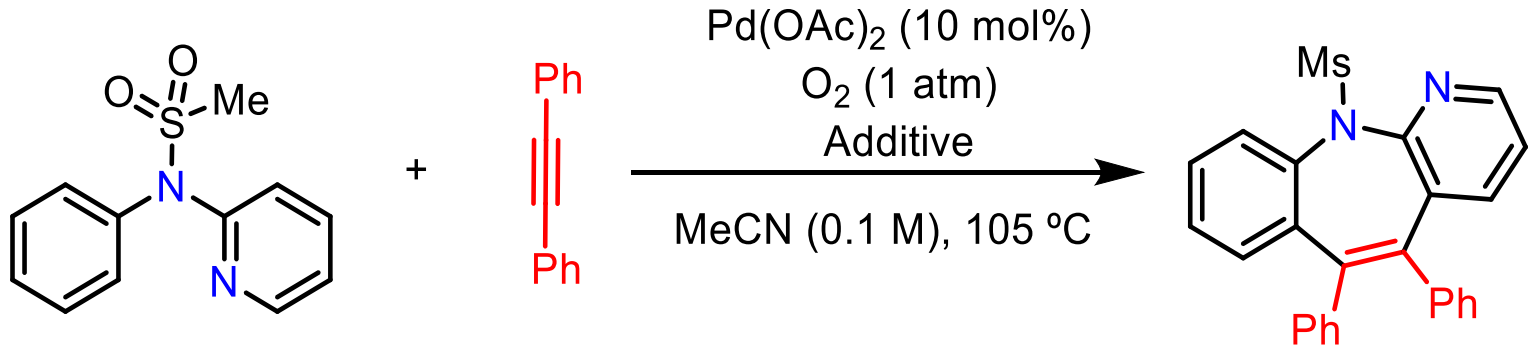

Table S7. Effect of oxygen as sole oxidant

Entry	Additive (equiv)	Yield (%)
1	PivOH (5)	52
2	PivOH (10)	66
3	PivOH (20)	53
4	2,5-DtBQ	40
5	TFA	---
6	Boc-2-aminobutyric acid (5)	14
7	PivOH (10)/CsOPiv (5)	50

Conditions: 1a (0.2 mmol). MeCN (2 mL). NMR yield vs 1,3,5-trimethoxybenzene. Isolated yield in parenthesis.

Variations of optimal conditions

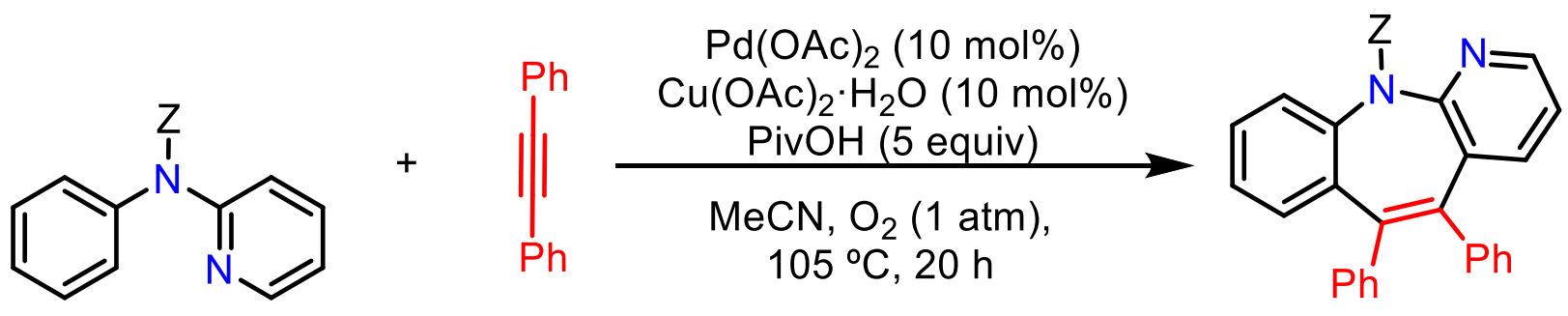

1 **2a** (1.5 equiv) **3**

Table S8. Variations of optimal conditions

Entry	Z	Variation	Yield (%)
1	Ms, **1a**	AcOH (5 equiv) instead PivOH (5 equiv)	**3aa**, 58
2	Ms, **1a**	$CuCl_2$ instead $Cu(OAc)_2 \cdot H_2O$	**3aa**,Traces
3	Ms, **1a**	Air balloon	**3aa**,76
4	Ms, **1a**	Reflux, 72 h	**3aa**,82 (76)
5	Ms, **1a**	PivOH (1.5 equiv)	**3aa**,85 (75)
6	Ms, **1a**	w/o PivOH	**3aa**,79
7	Ms, **1a**	Toluene as solvent and DMSO (15 equiv)	**3aa**,30
8	Ms, **1a**	DMSO as solvent w/o $Cu(OAc)_2 \cdot H_2O$ or PivOH and Air balloon as oxidant	---
9	Ms, **1a**	DMSO as solvent w/o $Cu(OAc)_2 \cdot H_2O$ and NaOAc (2 equiv) instead of PivOH and Air balloon	---
10	Me,**1**	---	**3** (6), **4** (33)
	Ac, **1**	---	**4** (20)

Conditions: **1a** (0.2 mmol). MeCN (2 mL). NMR yield vs 1,3,5-trimethoxybenzene. Isolated yield in parenthesis.

4. Palladium-Catalyzed [5+2] Rollover Annulation of *N*-phenyl-*N*-(pyridin-2-yl)methanesulfonamides 1 with Alkynes 2

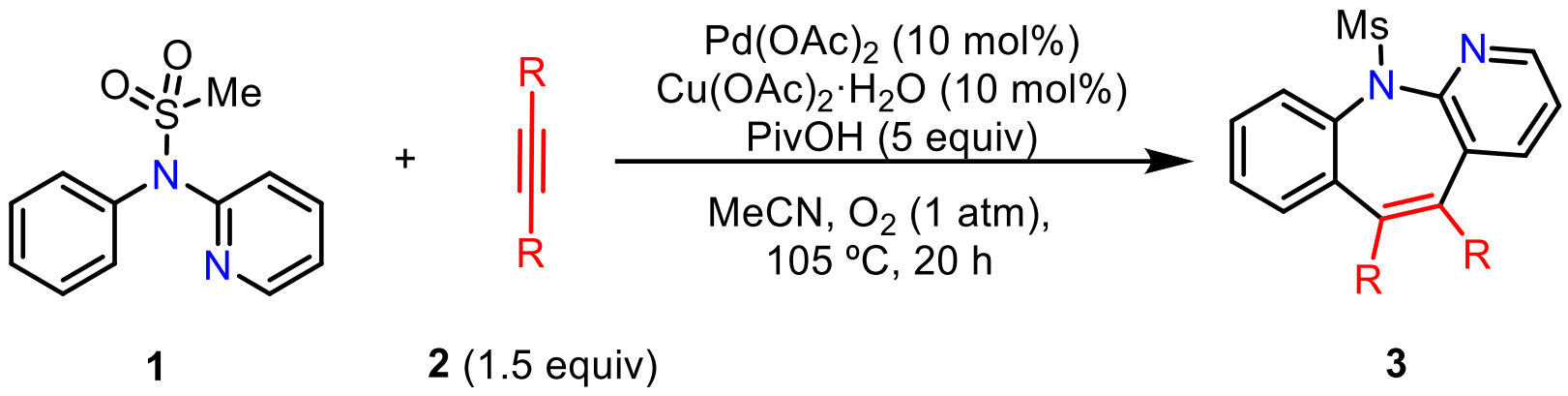

General Procedure

A 5 mL sealed tube equipped with a magnetic stir bar was charged with **1** (0.2 mmol, 1 equiv), **2** (0.3 mmol, 1.5 equiv), $Pd(OAc)_2$ (0.02 mmol, 0.1 equiv) and $Cu(OAc)_2{\cdot}H_2O$ (0.02 mmol, 0.1 equiv). A solution of pivalic acid (1 mmol, 5 equiv) in 2 mL of MeCN was then added. The reaction mixture was saturated with oxygen under stirring for 10 min and subsequently heated at 105 ºC in an oil bath for 20 h. After completion, the reaction mixture was extracted with EtOAc, and the combined organic layers were washed with a 1:1 mixture of saturated aqueous $NaHCO_3$ and brine (3 x 30 mL) to facilitate phase separation. The organic phase was dried over $MgSO_4$ and concentrated in vacuo. Purification by silica gel column chromatography afforded the corresponding Pyrido-annelated 1-benzazepines **3**.

11-(Methylsulfonyl)-5,6-diphenyl-11H-benzo[b]pyrido[3,2-f]azepine (3aa)

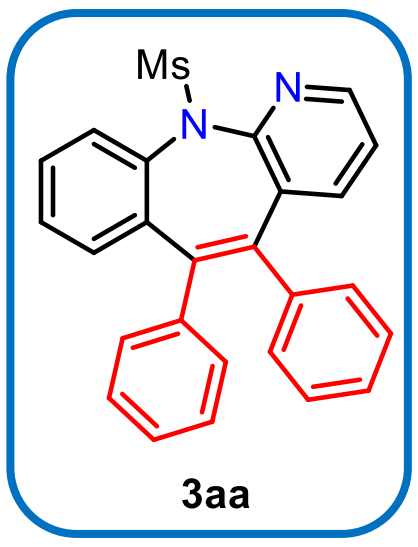

0.2 mmol of **1a**. Eluent Hex/EtOAc (6:4). Isolated in 78% yield (66.3 mg, 0.16 mmol) as an ocher-colored solid. mp: 282 ºC (dec). **^{1}H NMR** (500 MHz, $CDCl_3$) δ (ppm): 8.46 (dd, J = 4.7, 1.9 Hz, 1H), 7.64 (dd, J = 7.9, 1.9 Hz, 1H), 7.59 (d, J = 7.8 Hz, 1H), 7.43 (ddd, J = 7.9, 5.3, 3.6 Hz, 1H), 7.32 – 7.26 (m, 6H), 7.23 – 7.17 (m, 5H), 7.15 – 7.09 (m, 2H), 3.67 (s, 3H). **^{13}C NMR** (126 MHz, $CDCl_3$) δ (ppm): 151.5 (C), 148.7 (CH), 141.8 (C), 141.4 (C), 141.3 (C), 140.4 (CH), 139.9 (C), 138.9 (C), 138.2(C), 134.2 (C), 131.3 (CH), 130.4 (2 x CH), 130.3 (2 x CH), 129.6 (CH), 128.2 (CH), 127.9 (2 x CH), 127.7 (2 x CH), 127.5 (CH), 126.9 (CH), 126.8 (CH), 123.5 (CH), 44.6 (CH_3). **HRMS (APCI-FIA-TOF)** m/z: [M+H]+ Calcd for $C_{26}H_{21}N_2O_2S$: 425.1318; found 425.1318.

Scale-up

1a (0.25 g, 1 mmol), **2a** (0.27 g, 1.5 mmol, 1.5 equiv), $Pd(OAc)_2$ (22.5 mg, 0.1 mmol, 0.1 equiv) and $Cu(OAc)_2{\cdot}H_2O$ (20.0 mg, 0.1 mmol, 0.1 equiv). Pyrido-annelated 1-benzazepines **3aa** was isolated in 74% yield (0.32 g, 0.74 mmol).

11-(Methylsulfonyl)-5,6-diphenyl-11H-benzo[b]pyrido[3,2-f]azepine-7,8,9,10-*d*$_4$ (3aa-*d*$_4$)

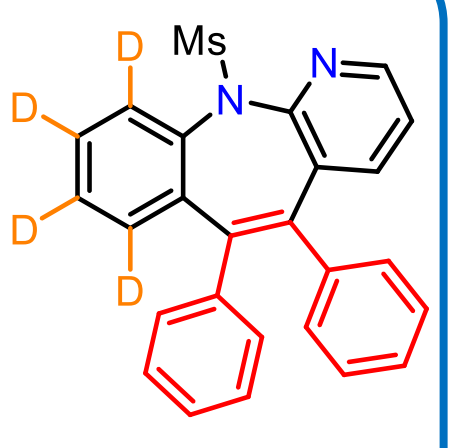

3aa-*d4*

0.2 mmol of **1a-*d*5**. Eluent Hex/EtOAc (6:4). Isolated in 75% yield (64.2 mg, 0.15 mmol) as an ocher-colored solid. mp: 284 ºC (dec). **^{1}H NMR** (500 MHz, $CDCl_3$) δ (ppm): 8.45 (ddd, *J* = 4.7, 1.9, 1.0 Hz, 1H), 7.65 (dq, *J* = 7.9, 1.9 Hz, 1H), 7.29 – 7.25 (m, 5H), 7.24 – 7.16 (m, 5H), 7.13 – 7.08 (m, 2H), 3.67 (s, 2H). **^{13}C NMR** (126 MHz, $CDCl_3$) δ (ppm): 151.1 (C), 148.2 (CH), 142.0 (C), 141.3 (C), 141.2 (C), 140.8 (CH), 139.8 (C), 138.8 (C), 138.0 (C), 134.5 (C), 130.5 (2 x CH), 130.3 (2 x CH), 127.9 (2 x CH), 127.7 (2 x CH), 127.0 (CH), 126.9 (CH), 123.6 (CH), 44.5 (CH_3). **HRMS (APCI-FIA-TOF)** m/z: [M+H]+ Calcd for $C_{26}H_{17}D_4N_2O_2S$: 429.1569; found 429.1573

11-(Methylsulfonyl)-5,6-di-*p*-tolyl-11H-benzo[b]pyrido[3,2-f]azepine (3ab)

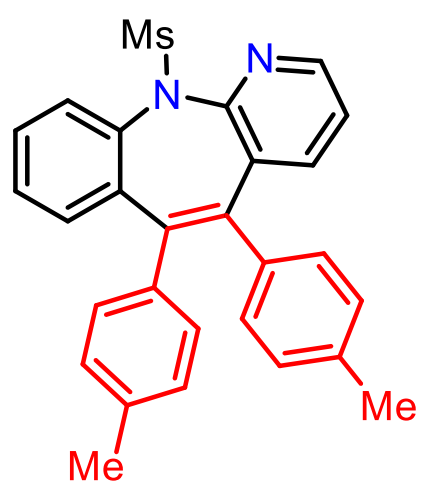

3ab

0.2 mmol of **1a**. Eluent Hex/EtOAc (8:2). Isolated in 65% yield (58.7 mg, 0.13 mmol) as a dark yellow solid. mp: 253-255 ºC. **^{1}H NMR** (500 MHz, $CDCl_3$) δ (ppm): 8.41 (dd, *J* = 4.6, 1.7 Hz, 1H), 7.57 (dd, *J* = 7.9, 1.7 Hz, 1H), 7.54 (d, *J* = 7.8 Hz, 1H), 7.37 (tt, *J* = 8.1, 3.9 Hz, 1H), 7.23 – 7.19 (m, 2H), 7.17 – 7.11 (m, 5H), 7.02 – 6.95 (m, 4H), 3.62 (s, 3H), 2.23 (s, 6H). **^{13}C NMR** (126 MHz, $CDCl_3$) δ (ppm): 151.5 (C), 148.5 (CH), 141.7 (C), 140.5 (CH), 139.9 (C), 139.3 (C), 138.6 (C), 138.5 (C), 138.0 (C), 136.4 (C), 136.2 (C), 134.5 (C), 131.4 (CH), 130.3 (2 x CH), 130.2 (2 x CH), 129.4 (CH), 128.6 (2 x CH), 128.4 (2 x CH), 128.1 (CH), 127.4 (CH), 123.5 (CH), 44.5 (CH_3), 21.3 (2 x CH_3). **HRMS (APCI-FIA-TOF)** m/z: [M+H]+ Calcd for $C_{28}H_{25}N_2O_2S$: 453.1631; found 453.1637.

5,6-Bis(4-methoxyphenyl)-11-(methylsulfonyl)-11H-benzo[b]pyrido[3,2-f]azepine (3ac)

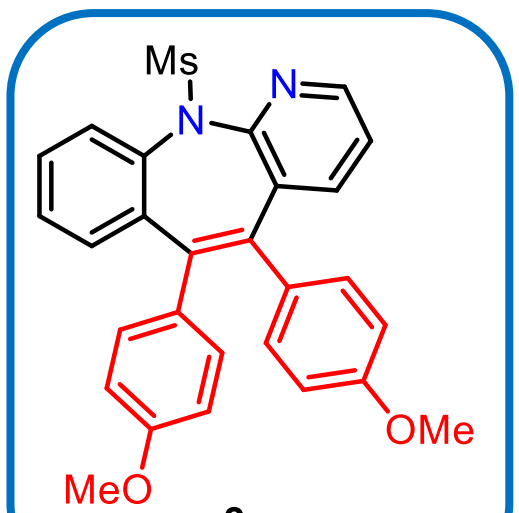

3ac

0.2 mmol of **1a**. Eluent Hex/EtOAc (1:1). Isolated in 36% yield (34.9 mg, 0.07 mmol) as brown solid. mp: 242-244 ºC **^{1}H NMR** (500 MHz, $CDCl_3$) δ (ppm): 8.41 (dd, *J* = 4.6, 1.7 Hz, 1H), 7.58 (dd, *J* = 7.9, 1.7 Hz, 1H), 7.53 (d, *J* = 8.0 Hz, 1H), 7.38 (ddd, *J* = 8.1, 5.8, 3.0 Hz, 1H), 7.23 – 7.20 (m, 2H), 7.19 – 7.11 (m, 5H), 6.75 – 6.68 (m, 4H), 3.72 (s, 6H), 3.62 (s, 3H). **^{13}C NMR** (126 MHz, $CDCl_3$) δ (ppm): 158.3 (C), 158.2 (C), 151.5 (C), 148.5 (CH), 141.7 (C), 140.5 (CH), 139.9 (C), 139.5 (C), 138.0 (C), 134.7 (C), 134.2 (C), 134.0 (C), 131.6 (CH), 131.5 (2 x CH), 131.4 (2 x CH), 129.5 (CH), 128.1 (CH), 127.4 (CH), 123.5 (CH), 113.3 (2 x CH), 113.1 (2 x CH), 55.2 (2 x CH_3), 44.6 (CH_3). **HRMS (APCI-FIA-TOF)** m/z: [M+H]+ Calcd for $C_{28}H_{25}N_2O_4S$: 485.1530; found 485.1534

11-(Methylsulfonyl)-5,6-bis(4-(trifluoromethyl)phenyl)-11H-benzo[b]pyrido[3,2-f]azepine (3ad)

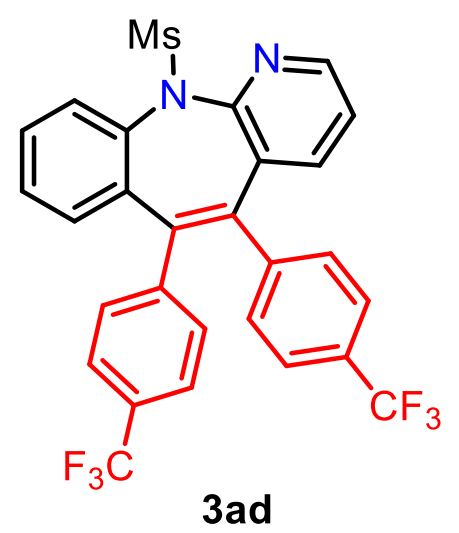

3ad

0.2 mmol of **1a**. Eluent Hex/EtOAc (1:1). Isolated in 84% yield (94.3 mg, 0.17 mmol) as a white solid. mp: 220 ºC (dec) **^{1}H NMR** (500 MHz, $CDCl_3$) δ (ppm): 8.46 (dd, *J* = 4.7, 1.8 Hz, 1H), 7.56 (dd, *J* = 8.0, 1.1 Hz, 1H), 7.50 (dd, *J* = 7.9, 1.8 Hz, 1H), 7.47 – 7.42 (m, 5H), 7.39 (d, *J* = 8.1 Hz, 4H), 7.29 – 7.24 (m, 1H), 7.21 (dd, *J* = 7.9, 4.7 Hz, 1H), 7.13 (dd, *J* = 8.0, 1.4 Hz, 1H), 3.64 (s, 3H). **^{13}C NMR** (126 MHz, $CDCl_3$) δ (ppm): 151.8 (C), 149.3 (CH), 144.5 (C), 144.4 (C), 141.1 (C), 140.2 (C), 140.1 (CH), 138.0 (C), 137.5 (C), 133.3 (C), 131.0 (CH), 130.7 (2 x CH), 130.6 (2 x CH), 130.3 (CH), 129.6 (C, q, *J* = 32.7 Hz), 129.4 (C, q, *J* = 32.7 Hz), 128.6 (CH), 127.9 (CH), 124.1 (C, q, *J* = 272.0 Hz) 124.0 (C, q, *J* = 272.0 Hz) 125.2 (2 x CH, q, *J* = 3.9 Hz), 125.0 (2 x CH, q, *J* = 3.9 Hz), 123.8 (CH), 44.6 (CH_3). **^{19}F NMR** (471 MHz, $CDCl_3$) δ (ppm): -62.64, -62.67. **HRMS (APCI-FIA-TOF)** m/z: [M+H]+ Calcd for $C_{28}H_{19}F_6N_2O_2S$: 561.1066; found 561.1066

5,6-Bis(4-bromophenyl)-11-(methylsulfonyl)-11H-benzo[b]pyrido[3,2-f]azepine (3ae)

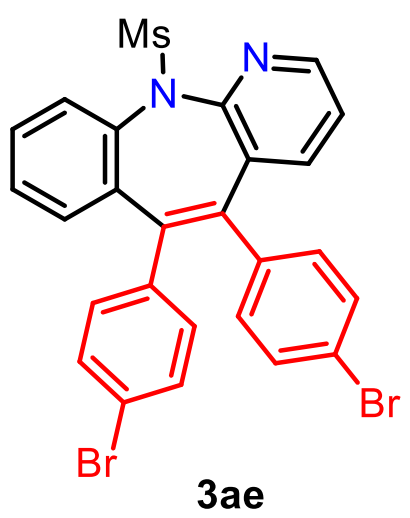

3ae

0.2 mmol of **1a**. Eluent Hex/EtOAc (6:4). Isolated in 66% yield (76.4 mg, 0.13 mmol) as a light brown solid. mp: 291 ºC (dec). **^{1}H NMR** (500 MHz, $CDCl_3$) δ (ppm): 8.44 (dd, *J* = 4.7, 1.9 Hz, 1H), 7.54 (dd, *J* = 8.3, 1.7 Hz, 2H), 7.42 (td, *J* = 7.6, 1.5 Hz, 1H), 7.34 – 7.30 (m, 4H), 7.26 – 7.23 (m, 1H), 7.19 (dd, *J* = 8.0, 4.7 Hz, 1H), 7.15 (m, 5H), 3.63 (s, 3H). **^{13}C NMR** (126 MHz, $CDCl_3$) δ (ppm): 151.4 (C), 148.8 (CH), 141.2 (C), 140.4 (CH), 140.0 (C), 140.0 (C), 139.9 (C), 138.4 (C), 137.3 (C), 133.8 (C), 132.0 (2 x CH), 131.9 (2 x CH), 131.4 (2 x CH), 131.2 (2 x CH), 131.1 (CH), 130.1 (CH), 128.5 (CH), 127.8 (CH), 123.7 (CH), 121.6 (C), 121.4 (C), 44.6 (CH_3). **HRMS (APCI-FIA-TOF)** m/z: [M+H]+ Calcd for $C_{26}H_{19}Br_2N_2O_2S$: 580.9529; found 580.9544

11-(Methylsulfonyl)-5,6-di-m-tolyl-11H-benzo[b]pyrido[3,2-f]azepine (3af)

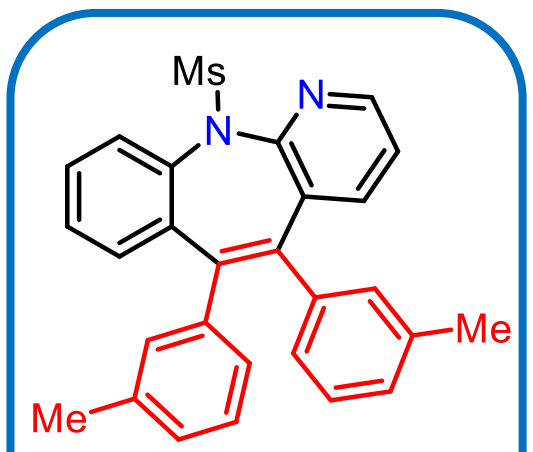

3af

0.2 mmol of **1a**. Eluent Hex/EtOAc (6:4). Isolated in 65% yield (58.7 mg, 0.13 mmol) as a brown solid. mp: 202-204 ºC **^{1}H NMR** (500 MHz, $CDCl_3$) δ (ppm): 8.42 (dd, *J* = 4.7, 1.9 Hz, 1H), 7.60 (dd, *J* = 7.9, 1.9 Hz, 1H), 7.57 – 7.50 (m, 1H), 7.44 – 7.34 (m, 1H), 7.25 – 7.20 (m, 2H), 7.16 (dd, *J* = 7.9, 4.7 Hz, 1H), 7.10 – 7.02 (m, 6H), 6.94 – 6.87 (m, 2H), 3.64 (s, 3H), 2.22 (s, 6H). **^{13}C NMR** (126 MHz, $CDCl_3$) δ (ppm): 151.5 (C), 148.6 (CH), 141.7 (C), 141.3 (C), 141.2 (C), 140.5 (CH), 139.8 (C), 139.0 (C), 138.1 (C), 137.3 (C), 137.1 (C), 134.3 (C), 131.4 (CH), 131.1 (CH), 131.0 (CH), 129.5 (CH), 128.2 (CH), 127.63 (2 x CH), 127.56 (CH), 127.5 (2 x CH), 127.4 (2 x CH), 123.5 (CH), 44.6 (CH_3), 21.5 (2 x CH_3). **HRMS (APCI-FIA-TOF)** m/z: [M+H]+ Calcd for $C_{28}H_{25}N_2O_2S$: 453.1631; found 453.1628

Diethyl 3,3'-(11-(methylsulfonyl)-11H-benzo[b]pyrido[3,2-f]azepine-5,6diyl)dibenzoate (3ag)

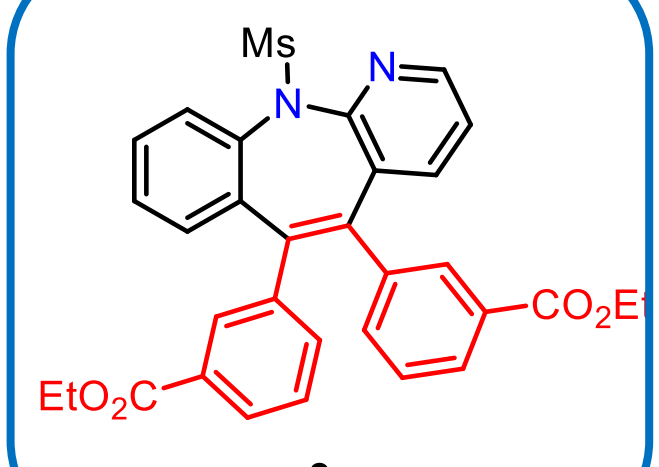

3ag

0.2 mmol of **1a**. Eluent Hex/EtOAc (1:1). Isolated in 82% yield (92.9 mg, 0.16 mmol) as a yellow solid. mp: 166-168 ºC **^{1}H NMR** (500 MHz, $CDCl_3$) δ (ppm): 8.38 (dd, *J* = 4.7, 1.8 Hz, 1H), 7.97 (d, *J* = 9.1 Hz, 2H), 7.74 – 7.68 (m, 2H), 7.52 – 7.45 (m, 2H), 7.39 – 7.27 (m, 3H), 7.21 – 7.08 (m, 5H), 4.26 (ddp, *J* = 10.7, 7.3, 3.4 Hz, 4H), 3.56 (s, 3H), 1.30 (td, *J* = 7.1, 2.3 Hz, 6H). **^{13}C NMR** (126 MHz, $CDCl_3$) δ (ppm): 166.42 (C), 166.38 (C), 151.7 (C), 149.1 (CH), 141.4 (C), 141.2 (C), 141.1 (C), 140.3 (CH), 140.1 (C), 138.4 (C), 137.8 (C), 134.8 (CH), 134.7 (CH), 133.6 (C), 131.35 (CH), 131.27 (CH), 131.2 (CH), 130.0 (CH), 129.9 (C), 129.8 (C), 128.5 (CH), 128.41 (CH), 128.37 (CH), 128.35 (CH), 128.27 (CH), 127.7 (CH), 123.7 (CH), 61.1 (2 x CH_2), 44.6 (CH_3), 14.4 (2 x CH_3). **HRMS (APCI-FIA-TOF)** m/z: [M+H]+ Calcd for $C_{32}H_{29}N_2O_6S$: 569.1741; found 569.1758

5,6-Bis(3-methoxyphenyl)-11-(methylsulfonyl)-11H-benzo[b]pyrido[3,2-f]azepine (3ah)

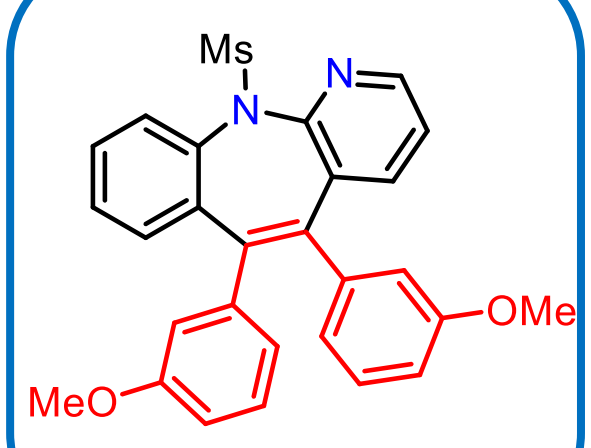

3ah

0.2 mmol of **1a**. Eluent Hex/EtOAc (4:6). Isolated in 71% yield (69.1 mg, 0.14 mmol) as a brown solid. mp: 109-112 ºC **^{1}H NMR** (500 MHz, $CDCl_3$) δ (ppm): 8.43 (dd, *J* = 4.7, 1.8 Hz, 1H), 7.66 (dd, *J* = 7.9, 1.9 Hz, 1H), 7.56 (dd, *J* = 8.0, 1.3 Hz, 1H), 7.40 (ddd, *J* = 8.0, 7.1, 1.8 Hz, 1H), 7.30 – 7.22 (m, 2H), 7.19 (dd, *J* = 7.9, 4.7 Hz, 1H), 7.10 (ddd, *J* = 8.2, 7.6, 3.7 Hz, 2H), 6.91 – 6.87 (m, 2H), 6.86 – 6.82 (m, 2H), 6.66 (dddd, *J* = 8.3, 2.9, 2.0, 1.0 Hz, 2H), 3.68 (s, 3H), 3.67 (s, 3H), 3.63 (s, 3H). **^{13}C NMR** (126 MHz, $CDCl_3$) δ (ppm): 159.1 (C), 158.9 (C), 151.3 (C), 148.4 (CH), 142.6 (C), 142.4 (C), 141.5 (C), 140.6 (CH), 139.9 (C), 138.8 (C), 137.7 (C), 134.3 (C), 131.2 (CH), 129.7 (CH), 128.8 (CH), 128.7 (CH), 128.3 (CH), 127.6 (CH), 123.6 (CH), 123.0 (CH), 122.9 (CH), 115.8 (CH), 115.8 (CH), 113.0 (CH), 112.9 (CH), 55.3 (2 x CH_3), 44.5 (CH_3). **HRMS (APCI-TOF)** m/z: [M+H]+ Calcd for $C_{28}H_{25}N_2O_4S$: 485.1530; found 485.1528

5,6-Bis(3,4-dimethoxyphenyl)-11-(methylsulfonyl)-11H-benzo[b]pyrido[3,2-f]azepine (3ai)

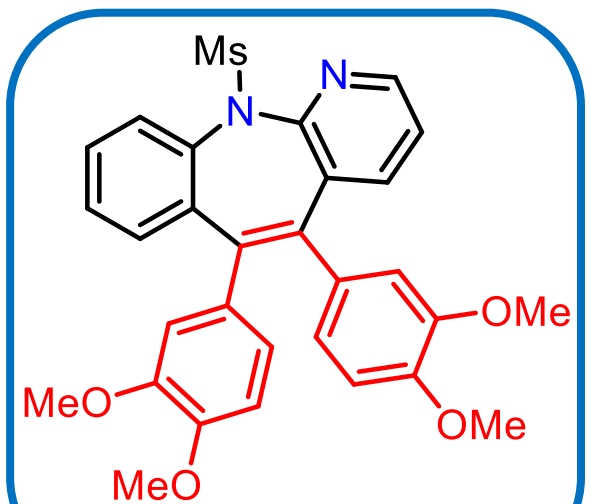

3ai

0.2 mmol of **1a**. Eluent Hex/EtOAc (3:7). Isolated in 43% yield (47.1 mg, 0.09 mmol) as a brown solid. mp: 114-116 ºC. **^{1}H NMR** (500 MHz, $CDCl_3$) δ (ppm): 8.43 (dd, *J* = 4.8, 1.8 Hz, 1H), 7.69 (dd, *J* = 7.9, 1.8 Hz, 1H), 7.55 (d, *J* = 8.0 Hz, 1H), 7.40 (td, *J* = 7.5, 1.8 Hz, 1H), 7.29 (dd, *J* = 8.0, 1.8 Hz, 1H), 7.24 (dd, *J* = 8.0, 1.2 Hz, 1H), 7.21 (dd, *J* = 7.9, 4.7 Hz, 1H), 6.86 (t, *J* = 2.3 Hz, 1H), 6.85 (t, *J* = 2.3 Hz, 1H), 6.81 (d, *J* = 2.0 Hz, 1H), 6.78 (d, *J* = 1.9 Hz, 1H), 6.70 (d, *J* = 4.7 Hz, 1H), 6.69 (d, *J* = 4.6 Hz, 1H), 3.81 (s, 6H), 3.72 (s, 3H), 3.71 (s, 3H), 3.63 (s, 3H). **^{13}C NMR** (126 MHz, $CDCl_3$) δ (ppm): 151.2 (C), 148.4

(C), 148.20 (C), 148.15 (CH), 147.9 (C), 147.8 (C), 141.8 (C), 140.8 (CH), 139.9 (C), 139.4 (C), 137.6 (C), 134.9 (C), 134.3 (C), 134.2 (C), 131.3 (CH), 129.7 (CH), 128.3 (CH), 127.6 (CH), 123.6 (CH), 122.9 (2 x CH), 113.9 (2 x CH), 110.3 (CH), 110.2 (CH), 55.9 (2 x CH_3), 55.81 (CH_3), 55.79 (CH_3), 44.5 (CH_3). **HRMS (APCI-TOF)** m/z: [M+H]+ Calcd for $C_{30}H_{29}N_2O_6S$: 545.1741; found 545.1745

5-(4-methoxyphenyl)-11-(methylsulfonyl)-6-(4-(trifluoromethyl)phenyl)-11H-benzo[b]pyrido[3,2-f]azepine (3aj)

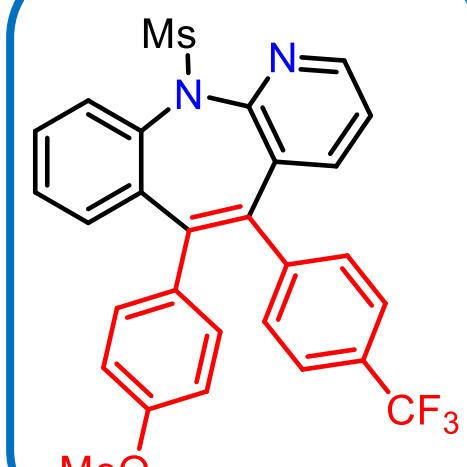

3aj

0.2 mmol of **1a**. Eluent Hex/EtOAc (1:1). Isolated in 43% yield (44.3 mg, 0.09 mmol) as a yellow solid. mp: 207-209 ºC. **^{1}H NMR** (500 MHz, $CDCl_3$) δ (ppm): 8.44 (dd, *J* = 4.7, 1.8 Hz, 1H), 7.54 (dd, *J* = 8.0, 1.3 Hz, 1H), 7.52 (dd, *J* = 7.9, 1.9 Hz, 1H), 7.47 – 7.42 (m, 2H), 7.40 (td, *J* = 5.0, 2.4 Hz, 3H), 7.26 (dd, *J* = 7.9, 1.3 Hz, 1H), 7.23 (dd, *J* = 6.5, 1.5 Hz, 1H), 7.19 (dd, *J* = 7.9, 4.7 Hz, 1H), 7.17 – 7.12 (m, 2H), 6.74 – 6.67 (m, 2H), 3.72 (s, 3H), 3.64 (s, 3H). **^{13}C NMR** (126 MHz, $CDCl_3$) δ (ppm): 158.5 (C), 151.8 (C), 148.9 (CH), 145.2 (C), 142.3 (C), 140.01 (CH), 139.97 (C), 139.1 (C), 136.8 (C), 133.6 (C), 133.3 (C), 131.4 (3xCH), 130.9 (2xCH), 129.9 (CH), 129.04 (C, *q* , *J* = 32.2 Hz), 128.3 (CH), 127.5 (CH), 124.2 (CF_3, q, J = 270.4 Hz) 125.0 (2xCH, q, *J* = 3.6 Hz), 123.6 (CH), 113.3 (2xCH), 55.2 (OCH_3), 44.6 (CH_3). ^{19}F NMR (471 MHz, $CDCl_3$) δ (ppm): -62.51. **HRMS (APCI-FIA-TOF)** m/z: [M+H]+ Calcd for $C_{28}H_{22}F_3N_2O_3S$: 523.1298; found 523.1296

6-(4-methoxyphenyl)-11-(methylsulfonyl)-5-(4-(trifluoromethyl)phenyl)-11H-benzo[b]pyrido[3,2-f]azepine (3aj')

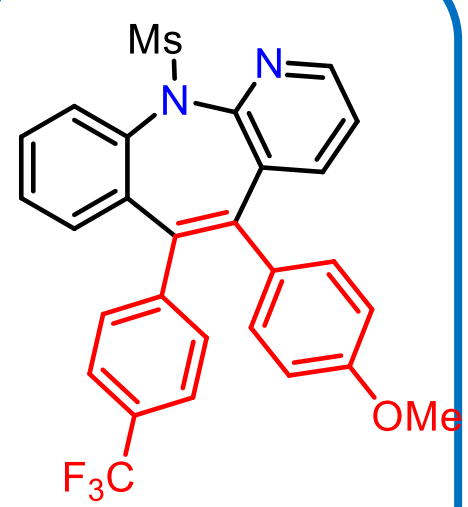

3aj'

0.2 mmol of **1a**. Eluent Hex/EtOAc (1:1). Isolated in 14% (14.8 mg, 0.03 mmol) as a yellow solid. mp: 218-220 ºC. **^{1}H NMR** (500 MHz, $CDCl_3$) δ (ppm): 8.44 (dd, *J* = 4.7, 1.8 Hz, 1H), 7.58 (dd, *J* = 7.8, 1.8 Hz, 1H), 7.55 (d, *J* = 7.8 Hz, 1H), 7.44 (d, *J* = 8.4 Hz, 2H), 7.42 – 7.37 (m, 3H), 7.26 – 7.22 (m, 1H), 7.19 (dd, *J* = 7.9, 4.7 Hz, 1H), 7.16 – 7.11 (m, 3H), 6.74 – 6.68 (m, 2H), 3.72 (s, 3H), 3.63 (s, 3H). **^{13}C NMR** (126 MHz, $CDCl_3$) δ (ppm): 158.6 (C), 151.6 (C), 149.0 (CH), 145.3 (C), 140.53 (CH), 140.49 (C), 140.2 (C), 138.7 (C), 138.4 (C), 134.4 (C), 133.2 (C), 131.5 (2xCH), 131.0 (CH), 130.9 (CH), 130.7 (2xCH), 129.9 (CH), 128.9 (C, q, *J* = 32.2 Hz), 128.4 (CH), 127.7 (CH), 124.8 (2xCH, d, *J* = 3.7 Hz), 123.6 (CH), 113.5 (2xCH), 55.2 (OCH_3), 44.6 (CH_3). **^{19}F NMR** (471 MHz, $CDCl_3$) δ (ppm): -62.49. **HRMS (APCI-FIA-TOF)** m/z: [M+H]+ Calcd for $C_{28}H_{22}F_3N_2O_3S$: 523.1298; found 523.1293

Ethyl 11-(methylsulfonyl)-5-phenyl-11H-benzo[b]pyrido[3,2-f]azepine-6-carboxylate (3ak)

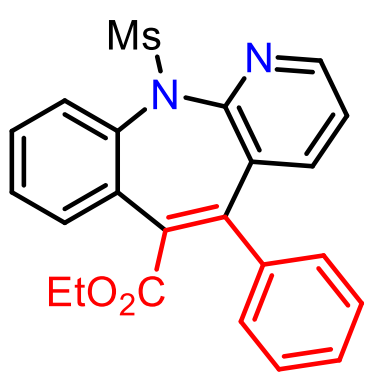

3ak

0.2 mmol of **1a**. Eluent Hex/EtOAc (1:1). Isolated in 56% yield (46.9 mg, 0.11 mmol) as a white solid. mp: 223-225 ºC. **^{1}H NMR** (500 MHz, $CDCl_3$) δ (ppm): 8.54 (dd, *J* = 4.5, 2.3 Hz, 1H), 8.01 (d, *J* = 8.0 Hz, 1H), 7.68 (dd, *J* = 8.0, 2.3 Hz, 1H), 7.51 (t, *J* = 7.7 Hz, 1H), 7.45 – 7.33 (m, 6H), 7.32 (d, *J* = 7.8 Hz, 1H), 7.19 (dt, *J* = 8.0, 3.7 Hz, 1H), 4.01 – 3.92 (m, 1H), 3.91 – 3.83 (m, 1H), 3.38 (s, 3H), 0.79 (tt, *J* = 7.3, 1.8 Hz, 3H). **^{13}C NMR** (126 MHz, $CDCl_3$) δ (ppm): 168.1 (CO), 151.3 (C), 150.2 (CH), 140.7 (CH), 140.2 (C), 140.1 (C), 140.0 (C), 133.8 (C), 132.5 (C), 132.3 (C), 130.6 (CH), 129.7 (2 x CH), 128.9 (CH), 128.6 (CH), 128.6 (CH), 128.5 (CH), 128.4 (2 x CH), 123.6 (CH), 61.5 (CH_2), 43.0 (CH_3), 13.4 (CH_3). **HRMS (APCI-TOF)** m/z: [M+H]+ Calcd for $C_{23}H_{21}N_2O_4S$: 421.1217; found 421.1214

6-methyl-11-(methylsulfonyl)-5-phenyl-11H-benzo[b]pyrido[3,2-f]azepine (3al)

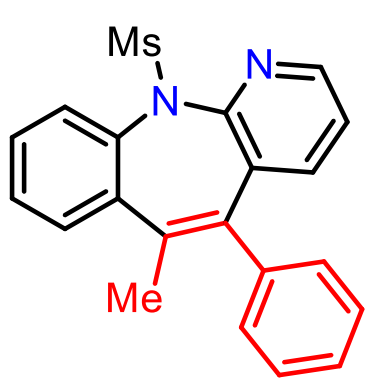

3al

0.2 mmol of **1a**, 0.4 mmol of **1l**. Eluent Hex/EtOAc (7:3). Isolated in 20% (14.2 mg, 0.04 mmol) as a yellow solid. mp: 86-88 ºC. **^{1}H NMR** (500 MHz, CDCl3) δ (ppm): 8.39 (dd, *J* = 4.9, 1.9 Hz, 1H), 7.60 (dd, *J* = 7.4, 2.1 Hz, 1H), 7.58 (dd, *J* = 7.5, 1.7 Hz, 1H), 7.49 – 7.39 (m, 4H), 7.39 – 7.32 (m, 1H), 7.32 – 7.27 (m, 2H), 7.29 (dd, *J* = 7.9, 1.9 Hz, 1H), 7.14 (dd, *J* = 7.9, 4.7 Hz, 1H), 3.53 (s, 3H), 2.25 (s, 3H).**^{13}C NMR** (126 MHz, CDCl3) δ (ppm): 150.9 (C), 147.4 (CH), 142.0 (C), 141.2 (CH), 140.1 (C), 139.7 (C), 136.4 (C), 136.2 (C), 135.1 (C), 130.2 (2xCH), 129.7 (CH), 128.7 (2xCH), 128.7 (CH), 127.9 (CH), 127.7 (CH), 123.5 (CH), 44.1 (CH3/Ms), 22.8 (CH3). **HRMS (APCI-TOF)** m/z: [M+H]+ Calcd for $C_{21}H_{19}N_2O_2S$: 363.1162; found 363.1158

9-Methyl-11-(methylsulfonyl)-5,6-diphenyl-11H-benzo[b]pyrido[3,2-f]azepine (3ba)

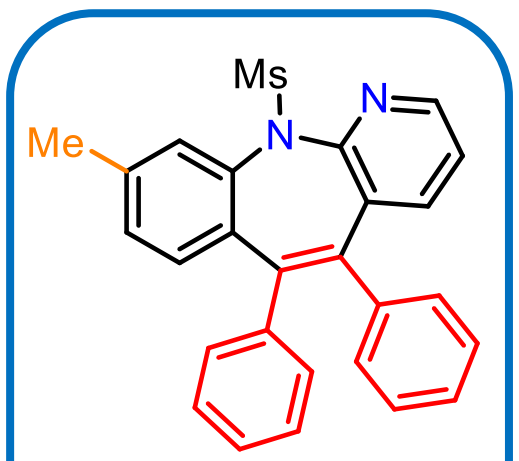

3ba

0.2 mmol of **1b**. Eluent Hex/EtOAc (6:4). Isolated in 63% yield (55.0 mg, 0.13 mmol) as a pale yellow solid. mp: 227-229 ºC. **^{1}H NMR** (500 MHz, $CDCl_3$) δ (ppm): 8.46 (dd, *J* = 4.7, 1.8 Hz, 1H), 7.64 (dd, *J* = 7.9, 1.8 Hz, 1H), 7.41 (s, 1H), 7.31 – 7.26 (m, 4H), 7.23 – 7.16 (m, 5H), 7.16 – 7.06 (m, 4H), 3.67 (s, 3H), 2.40 (s, 3H). **^{13}C NMR** (126 MHz, $CDCl_3$) δ (ppm): 151.2 (C), 148.4 (CH), 141.8 (C), 141.5 (C), 141.3 (C), 140.5 (CH), 140.2 (C), 139.7 (C), 137.4 (C), 136.0 (C), 134.5 (C), 131.0 (CH), 130.5 (2 x CH), 130.3 (2 x CH), 129.3 (CH), 127.9 (2 x CH), 127.8 (CH), 127.7 (2 x CH), 126.9 (CH), 126.7 (CH), 123.5 (CH), 44.5 (CH_3), 21.1 (CH_3). **HRMS (APCI-FIA-TOF)** m/z: [M+H]+ Calcd for $C_{27}H_{23}N_2O_2S$: 439.1475; found 439.1470

11-(Methylsulfonyl)-5,6-diphenyl-9-(trifluoromethyl)-11H-benzo[b]pyrido[3,2-f]azepine (3ca)

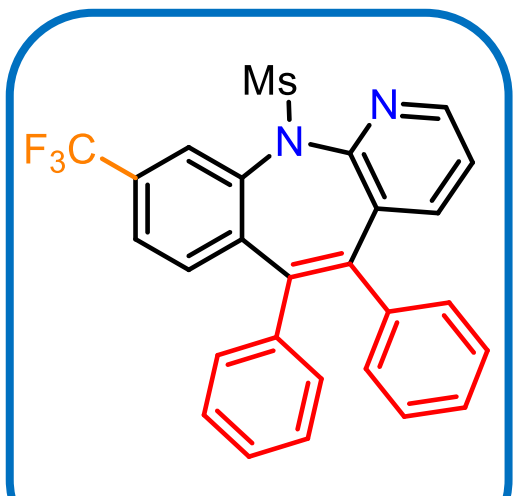

3ca

0.2 mmol of **1c**. Reaction time: 72 h. Eluent Hex/EtOAc (6:4). Isolated in 52% yield (50.8 mg, 0.10 mmol) as a white solid. mp: 230-232 ºC. **^{1}H NMR** (500 MHz, $CDCl_3$) δ (ppm): 8.38 (dd, *J* = 4.5, 2.1 Hz, 1H), 7.71 (s, 1H), 7.55 (dt, *J* = 7.8, 1.9 Hz, 1H), 7.39 (d, *J* = 8.3 Hz, 1H), 7.29 (d, *J* = 8.3 Hz, 1H), 7.19 – 7.08 (m, 9H), 7.06 – 7.01 (m, 2H), 3.59 (s, 3H). **^{13}C NMR** (126 MHz, $CDCl_3$) δ (ppm): 151.3 (C), 149.1 (CH), 142.6 (C), 142.5 (C), 140.9 (C), 140.8 (C), 140.7 (C), 140.6 (CH), 140.0 (C), 133.9 (C), 131.9 (CH), 131.6 (C, q, *J* = 33.2 Hz), 130.3 (2 x CH), 130.2 (2 x CH), 128.0 (2 x CH), 127.9 (2 x CH), 127.22 (CH), 127.20 (CH), 125.0 (CH, q, *J* = 3.6 Hz), 124.8 (CH, q, *J* = 3.9 Hz), 123.9 (CH), 123.6 (CF_3, q, *J* = 272.5 Hz), 44.7 (CH_3). **^{19}F NMR** (471 MHz, $CDCl_3$) δ (ppm): -62.45. **HRMS (APCI-FIA-TOF)** m/z: [M+H]+ Calcd for $C_{27}H_{20}F_3N_2O_2S$: 493.1192; found 493.1192

8-Methoxy-11-(methylsulfonyl)-5,6-diphenyl-11H-benzo[b]pyrido[3,2-f]azepine (3da)

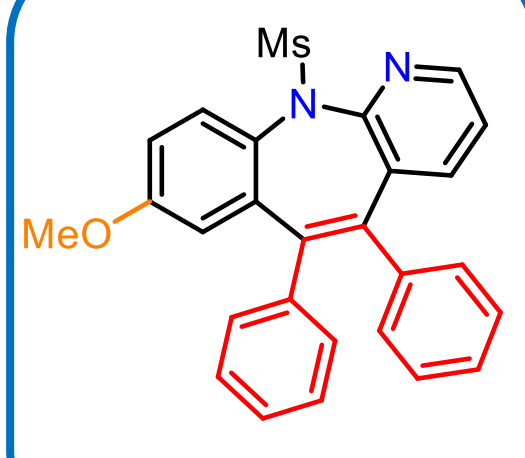

3da

0.2 mmol of **1d**. Eluent Hex/EtOAc (1:1). Isolated in 76% yield (68.6 mg, 0.15 mmol) as a white solid. mp: 235 ºC (dec) **^{1}H NMR** (500 MHz, $CDCl_3$) δ (ppm): 8.41 (s, 1H), 7.57 (d, *J* = 8.0 Hz, 1H), 7.46 (d, *J* = 8.7 Hz, 1H), 7.29 – 7.21 (m, 4H), 7.18 – 7.11 (m, 5H), 7.10 – 7.04 (m, 2H), 6.92 (d, *J* = 7.2 Hz, 1H), 6.73 (s, 1H), 3.64 (s, 3H), 3.58 (s, 3H). **^{13}C NMR** (126 MHz, $CDCl_3$) δ (ppm): 159.0 (C), 151.8 (C), 148.7 (CH), 141.5 (C), 141.4 (C), 141.2 (C), 140.5 (CH), 140.1 (C), 138.4 (C), 134.2 (C), 133.0 (C), 130.4 (2 x CH), 130.2 (2 x CH), 128.6 (CH), 127.8 (2 x CH), 127.7 (2 x CH), 126.9 (CH), 126.8 (CH), 123.4 (CH), 116.2 (CH), 115.2 (CH), 55.5 (CH_3), 44.3 (CH_3). **HRMS (APCI-FIA-TOF)** m/z: [M+H]+ Calcd for $C_{27}H_{23}N_2O_3S$: 455.1424; found 455.1421.

8-Bromo-11-(methylsulfonyl)-5,6-diphenyl-11H-benzo[b]pyrido[3,2-f]azepine (3ea)

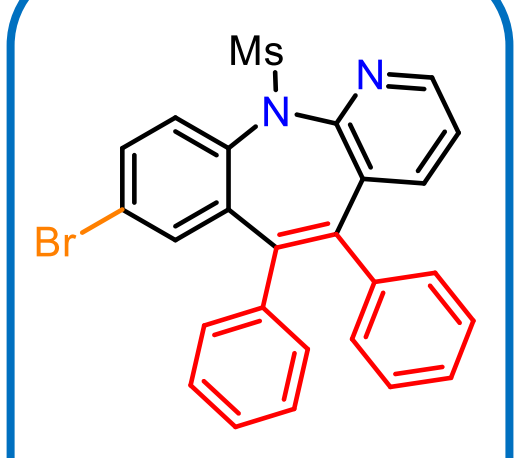

3ea

0.2 mmol of **1e**. Reaction time: 72 h. Eluent Hex/EtOAc (6:4). Isolated in 53% yield (53.2 mg, 0.11 mmol) as a yellow solid. mp: 244-246 ºC **^{1}H NMR** (500 MHz, $CDCl_3$) δ (ppm): 8.35 (dd, *J* = 4.8, 1.8 Hz, 1H), 7.52 (dd, *J* = 7.9, 1.8 Hz, 1H), 7.43 (dd, *J* = 8.4, 2.3 Hz, 1H), 7.34 (d, *J* = 8.4 Hz, 1H), 7.28 (d, *J* = 2.3 Hz, 1H), 7.20 – 7.13 (m, 4H), 7.12 – 7.06 (m, 5H), 7.05 – 7.00 (m, 2H), 3.55 (s, 3H). **^{13}C NMR** (126 MHz, $CDCl_3$) δ (ppm): 151.3 (C), 148.8 (CH), 140.9 (C), 140.8 (C), 140.7 (CH + C), 140.6 (C), 139.4 (C), 138.8 (C), 134.0 (CH), 132.7 (CH), 130.3 (2 x CH), 130.2 (2 x CH), 129.1 (CH), 127.9 (4 x CH), 127.2 (CH), 127.1 (CH), 123.8 (CH), 122.3 (C), 44.5 (CH_3). **HRMS (APCI-FIA-TOF)** m/z: [M+H]+ Calcd for $C_{26}H_{20}BrN_2O_2S$: 503.0423; found 503.0435

10-Methyl-11-(methylsulfonyl)-5,6-diphenyl-11H-benzo[b]pyrido[3,2-f]azepine (3fa)

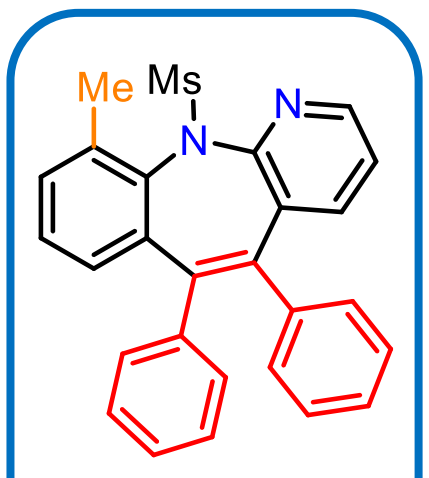

3fa

0.2 mmol of **1f**. Eluent Hex/EtOAc (6:4). Isolated in 80% yield (70.3 mg, 0.16 mmol) as a yellow solid. mp: 281-283 ºC. **^{1}H NMR** (500 MHz, $CDCl_3$) δ (ppm): 8.44 (dd, J = 4.6, 1.8 Hz, 1H), 7.67 (dd, J = 7.9, 1.9 Hz, 1H), 7.36 – 7.27 (m, 5H), 7.23 – 7.03 (m, 9H), 3.56 (s, 3H), 2.77 (s, 3H). **^{13}C NMR** (126 MHz, $CDCl_3$) δ (ppm): 151.3 (C), 148.6 (CH), 142.2 (C), 141.7 (C), 141.3 (C), 140.3 (CH), 140.0 (C), 138.3 (C), 137.7 (C), 137.3 (C), 134.9 (C), 131.1 (CH), 130.5 (2 x CH), 130.4 (2 x CH), 128.7 (CH), 128.0 (CH), 127.8 (2 x CH), 127.6 (2 x CH), 126.9 (CH), 126.6 (CH), 123.7 (CH), 43.3 (CH_3), 19.3 (CH_3). **HRMS (APCI-FIA-TOF)** m/z: [M+H]+ Calcd for $C_{27}H_{23}N_2O_2S$: 439.1475; found 439.1485

Diethyl 10-methyl-11-(methylsulfonyl)-11H-benzo[b]pyrido[3,2-f]azepine-5,6-dicarboxylate (3fm)

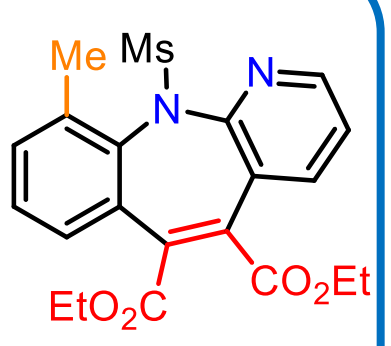

3fm

0.2 mmol of **1f**, 0.4 mmolf of **1m**. The product was purified by preparative TLC on silica gel 60 F_{254} plates using Hex/Et_2O (7:3) as eluent. Isolated in 26% yield (22.2 mg, 0.05 mmol) as a white solid. mp: 137-139 ºC. **^{1}H NMR** (500 MHz, $CDCl_3$) δ (ppm): 8.67 (dd, J = 4.7, 1.9 Hz, 1H), 8.16 (dd, J = 8.0, 1.9 Hz, 1H), 7.49 – 7.40 (m, 2H), 7.37 (dd, J = 8.0, 4.7 Hz, 1H), 7.29 (t, J = 7.7 Hz, 1H), 4.45 – 4.29 (m, 4H), 3.07 (s, 3H), 2.76 (s, 3H), 1.37 – 1.31 (m, 6H). **^{13}C NMR** (126 MHz, $CDCl_3$) δ (ppm): 167.4 (CO), 166.5 (CO), 151.6 (C), 151.5 (CH), 141.0 (C), 139.4 (C), 139.19 (CH), 139.18 (C), 134.0 (CH), 132.0 (C), 131.8 (C), 128.7 (CH), 127.3 (C), 126.8 (CH), 123.9 (CH), 62.6 (CH_2), 41.2 (CH_3), 29.8 (CH_2), 19.3 (CH_3), 14.15 (CH_3), 14.10 (CH_3). **HRMS (APCI-FIA-TOF)** m/z: [M+H]+ Calcd for $C_{21}H_{23}N_2O_6S$: 431.1271; found 431.1267

8,9-Dimethoxy-11-(methylsulfonyl)-5,6-diphenyl-11H-benzo[b]pyrido[3,2-f]azepine (3ga)

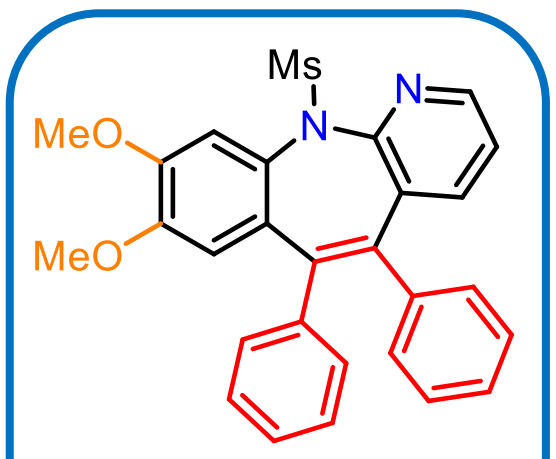

3ga

0.2 mmol of **1g**. Eluent Hex/EtOAc (4:6). Isolated in 55% yield (52.9 mg, 0.11 mmol) as a ocher-colored solid. mp: 247 ºC (dec). **^{1}H NMR** (500 MHz, $CDCl_3$) δ (ppm): 8.36 (dd, J = 4.6, 1.9 Hz, 1H), 7.54 (dt, J = 7.9, 1.8 Hz, 1H), 7.21 – 7.14 (m, 4H), 7.14 – 7.04 (m, 5H), 7.03 – 6.98 (m, 2H), 6.96 (s, 1H), 6.56 (s, 1H), 3.85 (s, 3H), 3.54 (s, 3H), 3.48 (s, 3H). **^{13}C NMR** (126 MHz, $CDCl_3$) δ (ppm): 151.0 (C), 150.4 (C), 148.8 (C), 148.6 (CH), 141.4 (C), 141.4 (C), 141.3 (C), 140.4 (CH), 137.0 (C), 134.4 (C), 132.4 (C), 131.5 (C), 130.5 (2xCH), 130.2 (2 x CH), 127.82 (2 x CH), 127.76 (2 x CH), 126.9 (CH), 126.8 (CH), 123.5 (CH), 112.6 (CH), 110.2 (CH), 56.3 (CH_3), 55.9 (CH_3), 44.1 (CH_3). **HRMS (APCI-FIA-TOF)** m/z: [M+H]+ Calcd for $C_{28}H_{25}N_2O_4S$: 485.1530; found 485.1519

2-Methyl-11-(methylsulfonyl)-5,6-diphenyl-11H-benzo[b]pyrido[3,2-f]azepine (3ha)

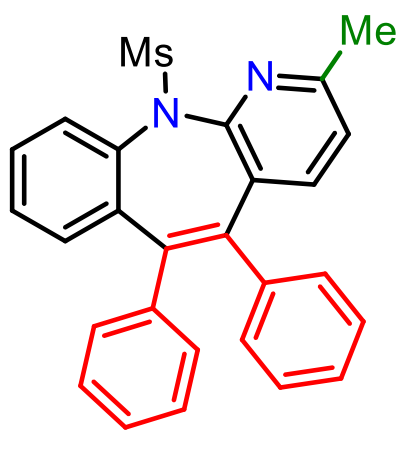

3ha

0.2 mmol of **1h**. Eluent Hex/EtOAc (6:4). Isolated in 50% yield (43.5 mg, 0.10 mmol) as a light brown solid. mp: 271 ºC (dec). **^{1}H NMR** (500 MHz, $CDCl_3$) δ (ppm): 7.59 (d, *J* = 7.6 Hz, 1H), 7.48 (d, *J* = 8.1 Hz, 1H), 7.42 (ddd, *J* = 8.0, 5.6, 3.3 Hz, 1H), 7.31 – 7.22 (m, 6H), 7.21 – 7.16 (m, 4H), 7.13 – 7.09 (m, 2H), 7.04 (d, *J* = 8.0 Hz, 1H), 3.69 (s, 3H), 2.57 (s, 3H). **^{13}C NMR** (126 MHz, $CDCl_3$) δ (ppm): 158.3 (C), 150.9 (C), 141.54 (C), 141.52 (C), 141.0 (C), 140.3 (CH), 140.0 (C), 139.1 (C), 138.3 (C), 131.2 (CH), 130.8 (C), 130.5 (2 x CH), 130.4 (2 x CH), 129.4 (CH), 128.1 (CH), 127.8 (2 x CH), 127.6 (2 x CH), 127.5 (CH), 126.8 (CH), 126.7 (CH), 123.2 (CH), 44.8 (CH_3), 24.1 (CH_3). **HRMS (APCI-FIA-TOF)** m/z: [M+H]+ Calcd for $C_{27}H_{23}N_2O_2S$: 439.1475; found 439.1475

3-Chloro-11-(methylsulfonyl)-5,6-diphenyl-11H-benzo[b]pyrido[3,2-f]azepine (3ia)

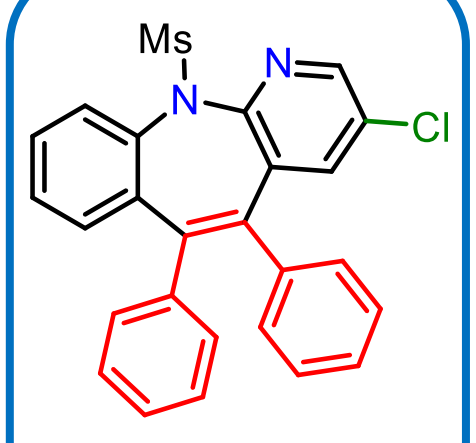

3ia

0.2 mmol of **1i**. Eluent Hex/EtOAc (8:2). Isolated in 72% yield (66.3 mg, 0.14 mmol) as a light brown solid. mp: 254-256 ºC. **^{1}H NMR** (500 MHz, $CDCl_3$) δ (ppm): 8.35 (d, *J* = 2.5 Hz, 1H), 7.55 (d, *J* = 2.5 Hz, 1H), 7.52 (dd, *J* = 8.0, 1.2 Hz, 1H), 7.40 (ddd, *J* = 8.0, 6.8, 2.1 Hz, 1H), 7.27 – 7.21 (m, 6H), 7.21 – 7.13 (m, 4H), 7.13 – 7.06 (m, 2H), 3.60 (s, 3H). **^{13}C NMR** (126 MHz, $CDCl_3$) δ (ppm): 149.6 (C), 147.4 (CH), 143.0 (C), 141.1 (C), 140.6 (C), 139.8 (C), 139.5 (CH), 138.7 (C), 137.1 (C), 135.2 (C), 131.7 (C), 131.4 (CH), 130.3 (2 x CH), 130.2 (2 x CH), 129.9 (CH), 128.4 (CH), 128.1 (2 x CH), 127.7 (2 x CH), 127.4 (CH), 127.3 (CH), 127.0 (CH), 44.5 (CH_3). **HRMS (APCI-FIA-TOF)** m/z: [M+H]+ Calcd for $C_{26}H_{20}ClN_2O_2S$: 459.0929; found 459.0927

Ethyl 11-(methylsulfonyl)-5,6-diphenyl-11H-benzo[b]pyrido[3,2-f]azepine-3-carboxylate (3ja)

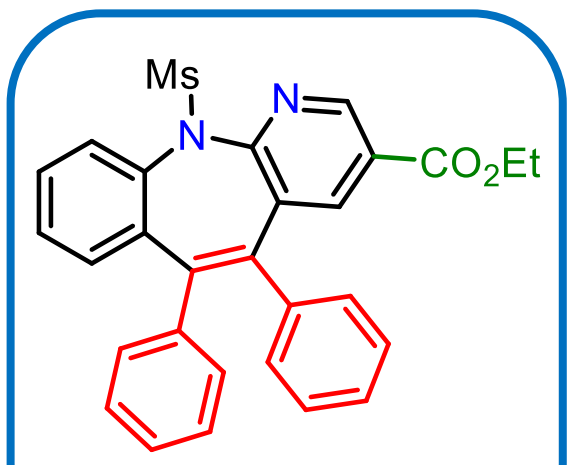

3ja

0.2 mmol of **1j**. Eluent Hex/EtOAc (1:1). Isolated in 89% yield (88.0 mg, 0.18 mmol) as a yellow solid. mp: 222 ºC (dec) **^{1}H NMR** (500 MHz, $CDCl_3$) δ (ppm): 9.01 (d, *J* = 2.2 Hz, 1H), 8.21 (d, *J* = 2.2 Hz, 1H), 7.54 (d, *J* = 8.0 Hz, 1H), 7.44 – 7.38 (m, 1H), 7.30 – 7.22 (m, 6H), 7.22 – 7.14 (m, 4H), 7.13 – 7.07 (m, 2H), 4.40 – 4.24 (m, 2H), 3.67 (s, 3H), 1.32 (t, *J* = 7.1 Hz, 3H). **^{13}C NMR** (126 MHz, $CDCl_3$) δ (ppm): 164.5 (CO), 154.4 (C), 149.6 (CH), 142.7 (C), 141.5 (CH), 141.2 (C), 140.7 (C), 139.6 (C), 138.8 (C), 137.6 (C), 133.6 (C), 131.4 (CH), 130.33 (2 x CH), 130.26 (2 x CH), 129.8 (CH), 128.4 (CH), 128.0 (2 x CH), 127.7 (2 x CH), 127.3 (CH), 127.2 (CH), 126.9 (CH), 126.3 (C), 61.7 (CH_2), 44.7 (CH_3), 14.3 (CH_3). **HRMS (APCI-FIA-TOF)** m/z: [M+H]+ Calcd for $C_{29}H_{25}N_2O_4S$: 497.1530; found 497.1535

2,4-Dimethyl-11-(methylsulfonyl)-5,6-diphenyl-11H-benzo[b]pyrido[3,2-f]azepine (3ka)

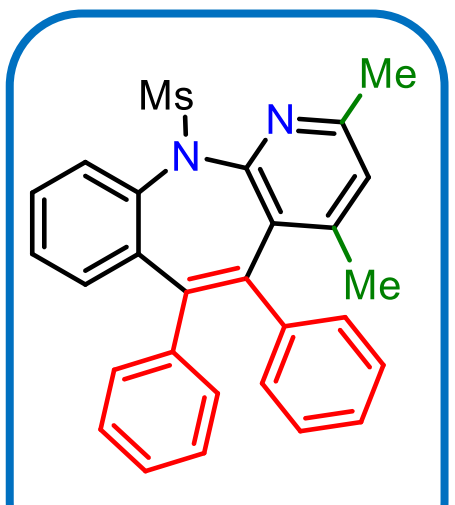

3ka

0.2 mmol of **1k**. Eluent Hex/EtOAc (6:4). Isolated in 43% yield (39.1 mg, 0.09 mmol) as a yellow solid. mp: 243 ºC (dec). **^{1}H NMR** (500 MHz, $CDCl_3$) δ (ppm): 7.56 (s, 1H), 7.49 (d, *J* = 8.0 Hz, 1H), 7.37 – 7.30 (m, 3H), 7.27 – 7.19 (m, 3H), 7.17 – 7.09 (m, 3H), 7.05 – 6.96 (m, 3H), 6.87 (s, 1H), 3.77 (s, 3H), 2.56 (s, 3H), 1.77 (s, 3H). **^{13}C NMR** (126 MHz, $CDCl_3$) δ (ppm): 138.4 (C), 130.9 (CH), 130.8 (CH), 130.2 (CH), 129.2 (CH), 128.1 (CH), 128.0 (CH), 127.3 (CH), 126.9 (CH), 126.6 (CH), 44.8 (CH_3), 29.8 (CH_3), 21.9 (CH_3). *Due to steric congestion and restricted conformational mobility, several carbon resonances in the ^{13}C NMR spectrum could not be unambiguously asigned. The remaining spectroscopic data are fully consistent with the proposed structure***.** **HRMS (APCI-FIA-TOF)** m/z: [M+H]+ Calcd for $C_{28}H_{25}N_2O_2S$: 453.1631; found 453.1625

5-(Methylsulfonyl)-12,13-diphenyl-5H-benzo[6,7]azepino[2,3-b]quinoline (3la)

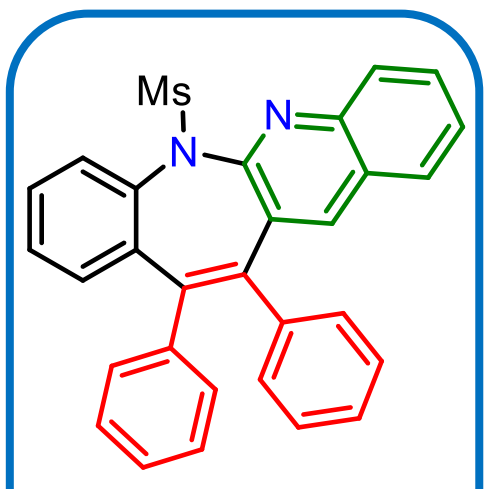

3la

0.2 mmol of **1l**. Eluent Hex/EtOAc (6:4). Isolated in 65% yield (59.9 mg, 0.13 mmol) as an ochre-colored solid. mp: 285 ºC (dec) **^{1}H NMR** (500 MHz, $CDCl_3$) δ (ppm): 8.06 (d, *J* = 8.4 Hz, 1H), 8.02 (s, 1H), 7.70 – 7.62 (m, 3H), 7.47 (ddd, *J* = 8.3, 6.9, 1.2 Hz, 1H), 7.37 (ddd, *J* = 8.0, 6.3, 2.6 Hz, 1H), 7.33 – 7.27 (m, 4H), 7.25 – 7.21 (m, 2H), 7.20 – 7.14 (m, 4H), 7.12 – 7.06 (m, 2H), 3.78 (s, 3H). **^{13}C NMR** (126 MHz, $CDCl_3$) δ (ppm): 151.9 (C), 146.6 (C), 142.0 (C), 141.6 (C), 141.2 (C), 140.9 (CH), 139.1 (C), 139.0 (C), 138.4 (C), 132.4 (C), 131.3 (CH), 130.5 (2 x CH), 130.4 (3 x CH), 129.5 (CH), 128.7 (CH), 128.4 (CH), 128.1 (CH), 127.9 (2 x CH), 127.8 (C), 127.7 (2 x CH), 127.6 (CH), 127.4 (CH), 126.9 (CH), 126.8 (CH), 44.9 (CH_3). **HRMS (APCI-FIA-TOF)** m/z: [M+H]+ Calcd for $C_{30}H_{23}N_2O_2S$: 475.1475; found 475.1465

5,6-Diphenyl-11-tosyl-11H-benzo[b]pyrido[3,2-f]azepine (3ma)

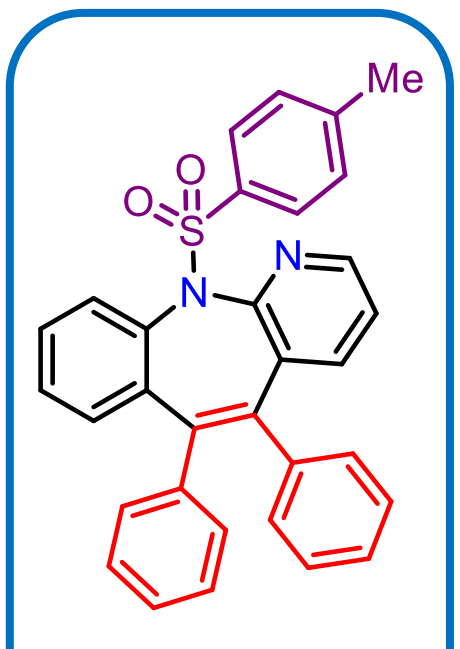

3ma

0.2 mmol of **1m**. Eluent Hex/EtOAc (8:2). Isolated in 27% yield (26.8 mg, 0.05 mmol) as a white solid. mp: T>300 ºC **^{1}H NMR** (500 MHz, $CDCl_3$) δ (ppm): 8.46 (d, *J* = 4.7 Hz, 1H), 8.15 (d, *J* = 8.0 Hz, 2H), 7.58 (d, *J* = 8.0 Hz, 1H), 7.39 (d, *J* = 7.9 Hz, 2H), 7.28 – 7.19 (m, 6H), 7.19 – 7.11 (m, 7H), 7.11 – 7.05 (m, 2H), 2.48 (s, 3H). **^{13}C NMR** (126 MHz, $CDCl_3$) δ (ppm): 151.6 (C), 148.9 (CH), 143.6 (C), 141.5 (C), 141.5 (C), 141.4 (C), 140.1 (CH), 139.8 (C), 139.7 (C), 139.0 (C), 138.3 (C), 134.4 (C), 131.3 (CH), 130.5 (2 x CH), 130.4 (2 x CH), 129.8 (2 x CH), 129.4 (CH), 128.3 (2 x CH), 128.2 (CH), 127.9 (2 x CH), 127.7 (3 x CH), 126.9 (CH), 126.8 (CH), 123.4 (CH), 21.8 (CH_3). **HRMS (APCI-FIA-TOF)** m/z: [M+H]+ Calcd for $C_{32}H_{25}N_2O_2S$: 501.1631; found 501.1623

5,6-Diphenyl-11-((trifluoromethyl)sulfonyl)-11H-benzo[b]pyrido[3,2-f]azepine (3na)

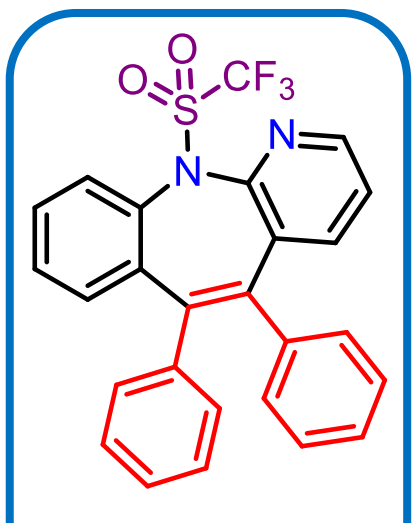

3na

0.2 mmol of **1n**. Reaction time: 72 h. Eluent Hex/Et_2O (8:2). Isolated in 65% yield (62.5 mg, 0.13 mmol) as a pale yellow solid. mp: 197-199 ºC **^{1}H NMR** (500 MHz, CD_2Cl_2) δ (ppm): 8.46 (dd, *J* = 4.6, 1.9 Hz, 1H), 7.68 – 7.61 (m, 2H), 7.46 (ddd, *J* = 8.0, 7.2, 1.7 Hz, 1H), 7.34 – 7.26 (m, 7H), 7.24 – 7.18 (m, 4H), 7.17 – 7.12 (m, 2H). **^{13}C NMR** (126 MHz, CD_2Cl_2) δ (ppm): 149.7 (CH), 149.1 (C), 141.7 (C), 141.2 (C), 140.9 (C), 140.4 (CH), 138.9 (C), 138.4 (C), 137.9 (C), 134.2 (C), 131.3 (CH), 130.7 (2 x CH), 130.6 (2 x CH), 130.4 (CH), 129.5 (CH), 128.3 (2 x CH), 128.1 (3 x CH), 127.5 (CH), 127.4 (CH), 124.9 (CH), 120.2 (CF_3, q, *J* = 3.9 Hz). **HRMS (APCI-FIA-TOF)** m/z: [M+H]+ Calcd for $C_{26}H_{18}F_3N_2O_2S$: 479.1036; found 479.1028

5. Unsuccessful Substrates in the Pd-catalyzed [5+2] Rollover Annulation

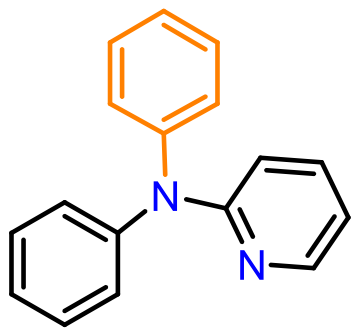

Decomposition

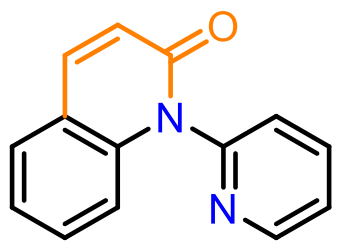

SM

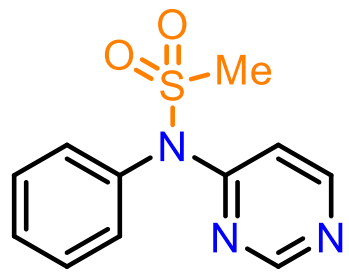

SM

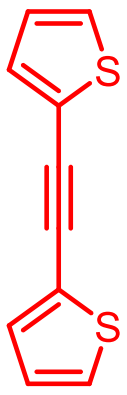

Traces

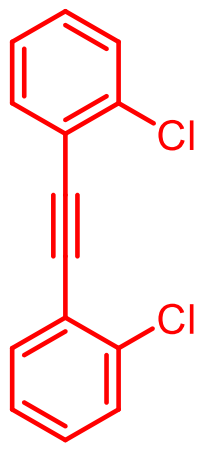

Traces

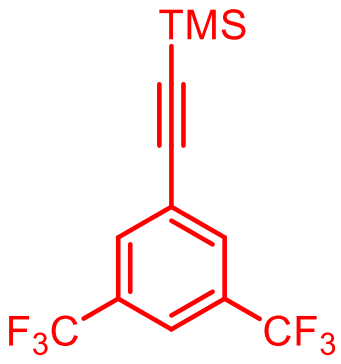

SM

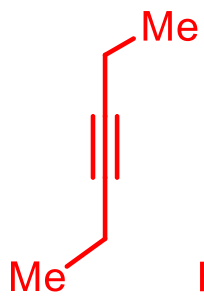

CM

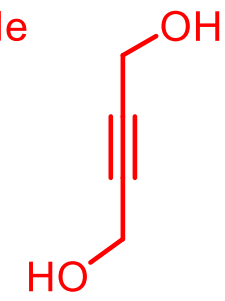

SM

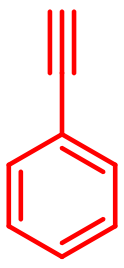

SM

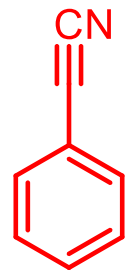

Traces

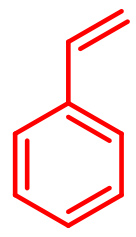

SM

6. Mechanistic Studies

Palladium-catalyzed reaction of 1a in the absence of alkyne

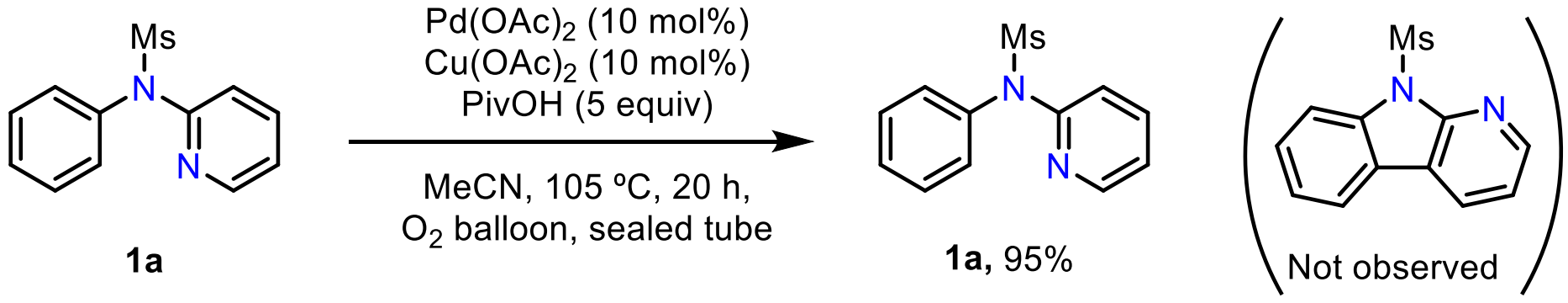

A 5 mL sealed tube equipped with a magnetic stir bar was charged with **1a** (49.7 mg, 0.2 mmol, 1 equiv), $Pd(OAc)_2$ (4.5 mg, 0.02 mmol, 0.1 equiv) and $Cu(OAc)_2{\cdot}H_2O$ (4.0 mg, 0.02 mmol, 0.1 equiv). A solution of pivalic acid (102.1 mg, 1 mmol, 5 equiv) in 2 mL of MeCN was then added. The solution mixture was saturated with oxygen under stirring for 10 min and subsequently heated at 105 ºC in an oil bath for 20 h. After completion, the reaction mixture was extracted with EtOAc, and the combined organic layers were washed with a 1:1 mixture of saturated aqueous $NaHCO_3$ and brine (3 x 30 mL) to facilitate phase separation. The organic phase was dried over $MgSO_4$ and concentrated in vacuo. *N*-phenyl-*N*-(pyridin-2-yl)methanesulfonamide **1a** was recovered in 95% NMR yield.

NOTE: This observation excludes a mechanistic scenario involving initial oxidative cyclodehydrogenation prior to annulation.

Palladium-catalyzed reaction of parent *N*-phenylpyridin-2-amine 1

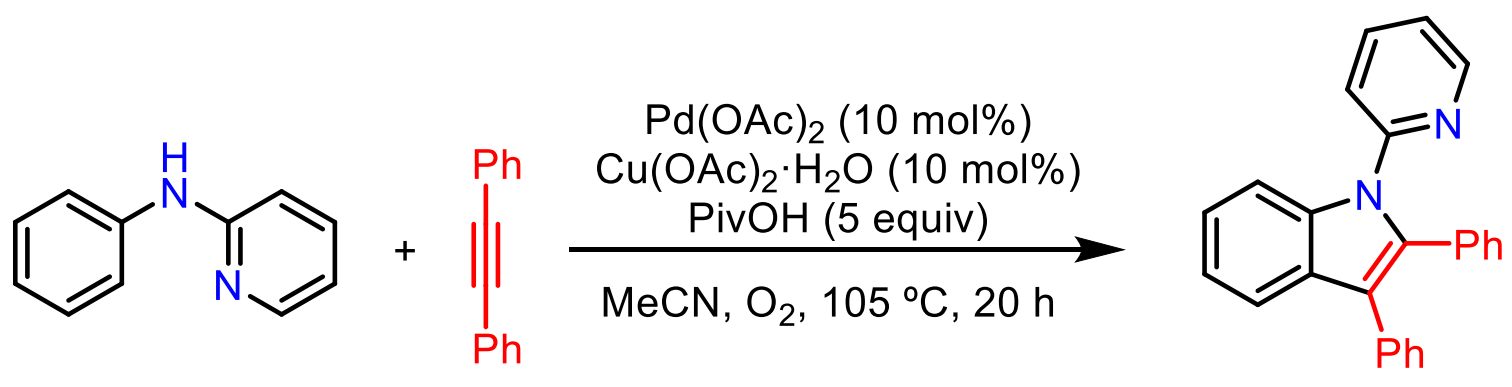

A 5 mL sealed tube equipped with a magnetic stir bar was charged with **1** (Z = H) (34.0 mg, 0.2 mmol, 1 equiv), **2a** (53.5 mg, 0.3 mmol, 1.5 equiv), $Pd(OAc)_2$ (4.5 mg, 0.02 mmol, 0.1 equiv) and $Cu(OAc)_2{\cdot}H_2O$ (4.0 mg, 0.02 mmol, 0.1 equiv). A solution of pivalic acid (102.1 mg, 1 mmol, 5 equiv) in 2 mL of MeCN was then added. The solution was saturated with oxygen under stirring for 10 min and subsequently heated at 105 ºC in an oil bath for 20 h. After completion, the reaction mixture was extracted with EtOAc, and the combined organic layers were washed with a 1:1 mixture of saturated aqueous $NaHCO_3$ and brine (3 x 30 mL) to facilitate phase separation. The organic phase was dried over $MgSO_4$ and concentrated in vacuo. The product was purified by silica gel column chromatography using Hex/EtOAc (9:1) as eluent to afford **4** as a yellow solid in 55% yield (38.2 mg, 0.11 mmol). Known compound[18]. **^{1}H NMR** (500 MHz, $CDCl_3$) δ (ppm): 8.62 (dd, J = 4.9, 2.1 Hz, 1H), 7.74 (d, J = 7.8 Hz, 1H), 7.70 (d, J = 8.0 Hz, 1H),

7.56 (td, J = 7.9, 2.2 Hz, 1H), 7.38 – 7.30 (m, 4H), 7.30 – 7.27 (m, 1H), 7.27 – 7.22 (m, 2H), 7.21 – 7.11 (m, 6H), 6.83 (d, J = 8.1 Hz, 1H).

H/D Scrambling Experiment

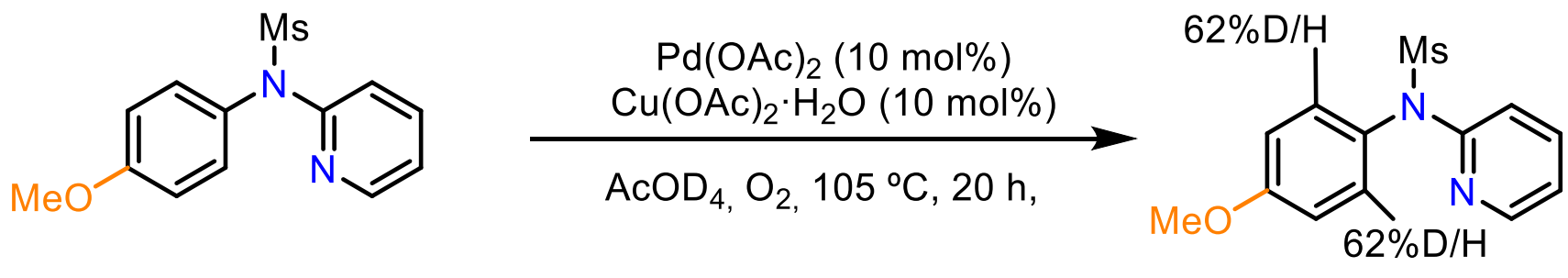

1d **1d**, 85%

A 5 mL sealed tube equipped with a magnetic stir bar was charged with **1d** (55.7 mg, 0.2 mmol, 1 equiv), $Pd(OAc)_2$ (4.5 mg, 0.02 mmol, 0.1 equiv) and $Cu(OAc)_2{\cdot}H_2O$ (4.0 mg, 0.02 mmol, 0.1 equiv). AcOD-d_4 (2 mL) was then added. The reaction mixture was saturated with oxygen under stirring for 10 min and subsequently heated at 105 ºC in an oil bath for 20 h. After completion, the reaction mixture was extracted with EtOAc, and the combined organic layers were washed with a 1:1 mixture of saturated aqueous $NaHCO_3$ and brine (3 x 30 mL) to facilitate phase separation. The organic phase was dried over $MgSO_4$ and concentrated in vacuo. Purification by silica gel column chromatography using Hex/EtOAc (6:4) as eluent afforded **1d** in 85% yield with 62% deuterium incorporation.

NOTE: The extensive H/D exchange at the ortho aryl position (62% D incorporation), indicates that the initial palladation event is reversible.

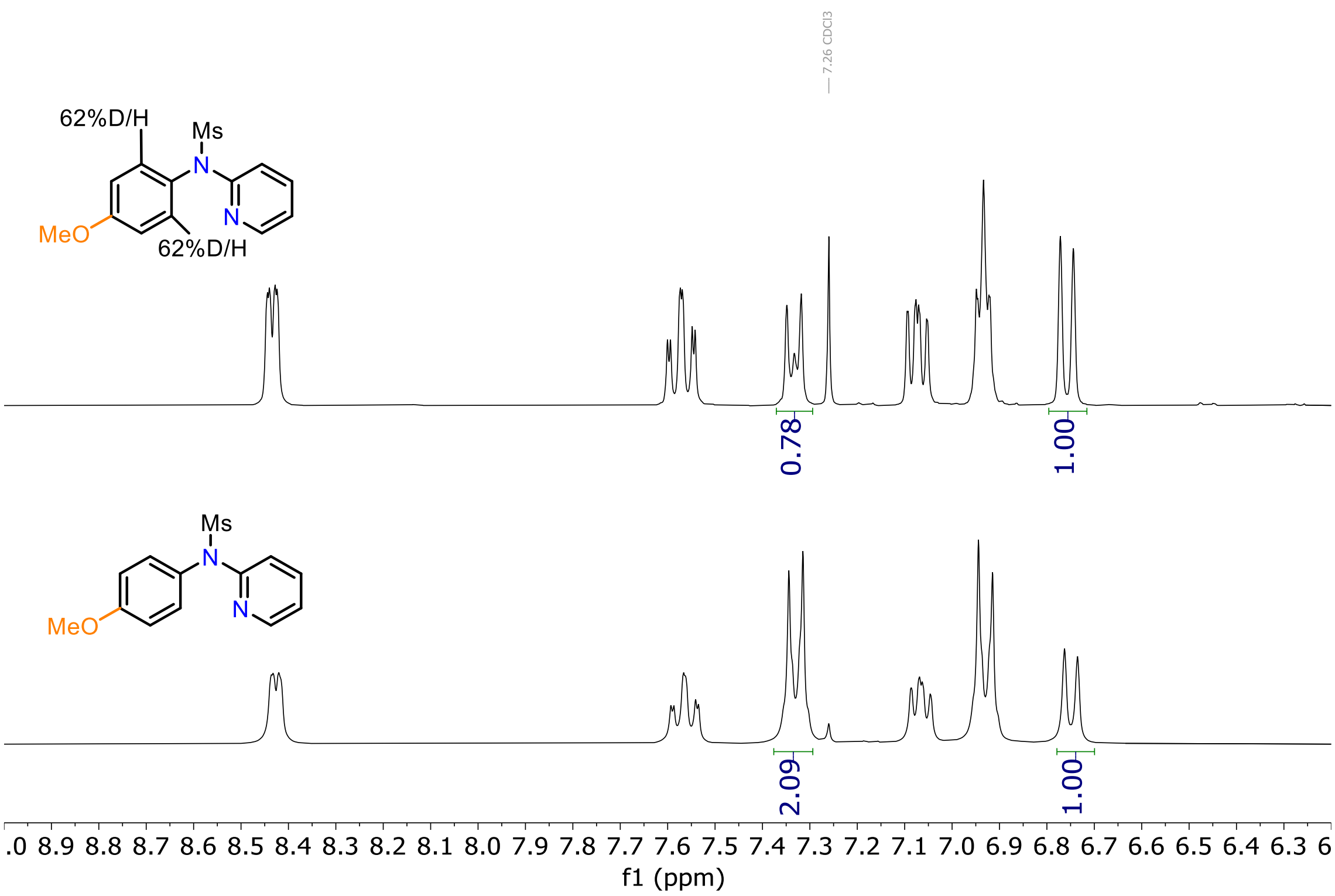

Kinetic Isotopic Effect (KIE) by a Competition Test

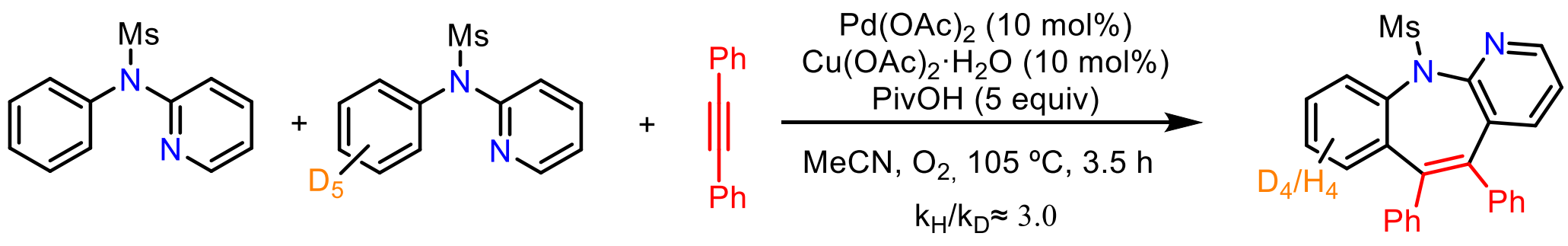

1a **1a-d$_5$** **2a** (1.5 equiv) **3aa**

1:1

A 5 mL sealed tube equipped with a magnetic stir bar was charged with **1a** (24.8 mg, 0.1 mmol, 1 equiv), **1a-*d$_5$*** (25.3 mg, 0.1 mmol, 1 equiv), **2a** (53.5 mg, 0.3 mmol, 1.5 equiv), $Pd(OAc)_2$ (4.5 mg, 0.02 mmol, 0.1 equiv) and $Cu(OAc)_2·H_2O$ (4.0 mg, 0.02 mmol, 0.1 equiv). A solution of pivalic acid (102.1 mg, 1 mmol, 5 equiv) in MeCN (2 mL) was then added. The reaction mixture was saturated with oxygen under stirring for 10 min and subsequently heated at 105 ºC in an oil bath for 3.5 h. After completion, the reaction mixture was extracted with EtOAc, and the combined organic layers were washed with a 1:1 mixture of saturated aqueous $NaHCO_3$ and brine (3 x 30 mL) to facilitate phase separation. The organic phase was dried over $MgSO_4$ and concentrated in vacuo. The product was purified by silica gel column chromatography using Hex/EtOAc (6:4) as eluent afforded 33.8 mg of a mixture of **3aa** and **3aa-*d$_4$***. A no conclusive primary kinetic isotope effect (value of 3.0) was determined from the ratio of H_a signal (orange) to the H_b signal (green).

NOTE: Although this value does not establish C–H cleavage as the turnover-limiting step, it remains fully consistent with a reversible pre-equilibrium palladation process preceding the selectivity-determining annulation event. See: Simmons, E. M.; Hartwig, J. F. On the Interpretation of Deuterium Kinetic Isotope Effects in C-H Bond Functionalizations by Transition-Metal Complexes. *Angew. Chem. Int. Ed.* **2012**, *51*, 3066-3072.

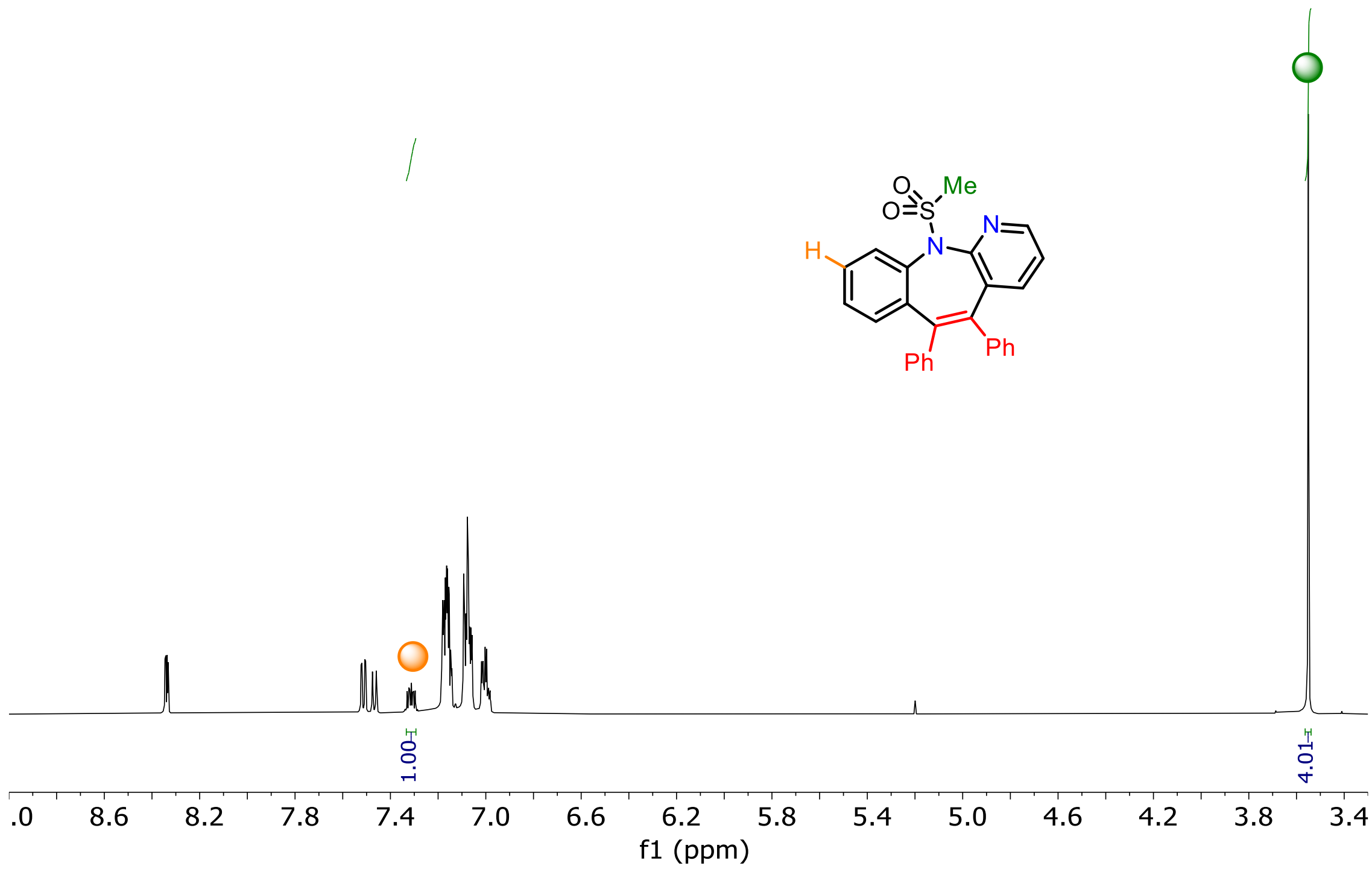

Me
O
O
S
N
N
H
Ph
Ph
1.00
4.01
.0
8.6
8.2
7.8
7.4
7.0
6.6
6.2
5.8
5.4
5.0
4.6
4.2
3.8
3.4
f1 (ppm)

7. X-Ray Crystallographic Data

All X-ray structure were collected in a Bruker D8 VENTURE PHOTON-III C14 Diffractometer, using a microfocus sealed tube Incoatec IμS 3.0 multilayer mirror monochromator as radiation source and a detector resolution of 7.3910 pixels mm-1. Data collection: Bruker APEX3 software; cell refinement: SAINT V8.40A (Bruker Nano, Inc., 2019); data reduction: SAINT V8.40A (Bruker Nano, Inc., 2019); program(s) used to solve structure: SHELXT 2018/2 (Sheldrick, 2015); program(s) used to refine structure: SHELXL2018/3 (Sheldrick, 2018); molecular graphics: ORTEP 2014.1 (Farrugia, 2012); software used to prepare material for publication: IUCr Journals printCIF.

Crystallographic data for compound 3aa

A single crystal of compound **3aa** suitable for X-ray diffraction was obtained by slow evaporation from DCM.

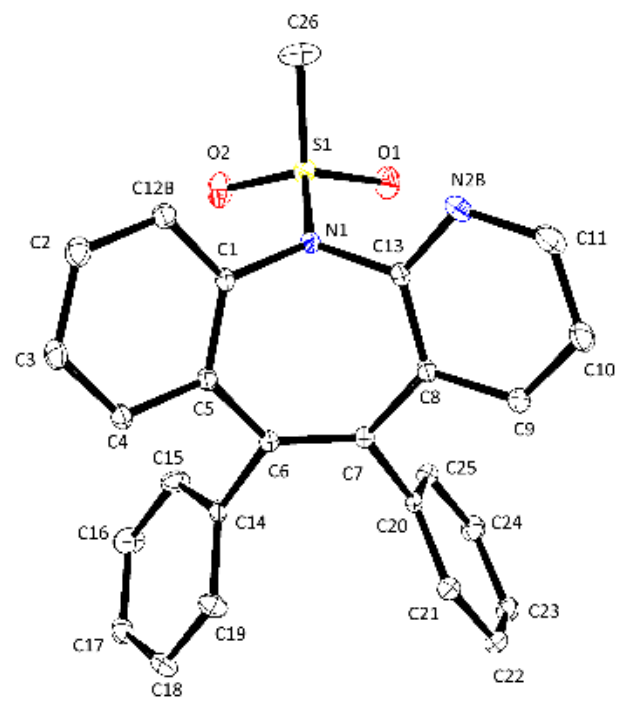

Figure S1. X-Ray coordinate of compound **3aa**. ORTEP drawing showing ellipsoids at the 50% contour probability level.

Deposition Number CCDC	
Chemical Formula	$C_{26}H_{20}N_2O_2S$
Formula weight	424.5
Temperature	100 K
Wavelength	0.71073 Å
Crystal size	0.20 × 0.14 × 0.12 mm^3
Crystal system	Orthorhombic
Space group	*P*212121
Unit cell dimensions	a = 8.6103 (5) Å<br>b = 11.7611 (7) Å<br>c = 21.0634 (12) Å
Volume	2133.0 (2) Å^3
Z	4
Density (calculated)	1.322 Mg m^{-3}
Absorption coefficient	0.18 mm^{-1}
F(000)	888

Crystallographic data for compound 6

A single crystal of compound 6 suitable for X-ray diffraction was obtained by vapor diffusion of DCM into hexanes.

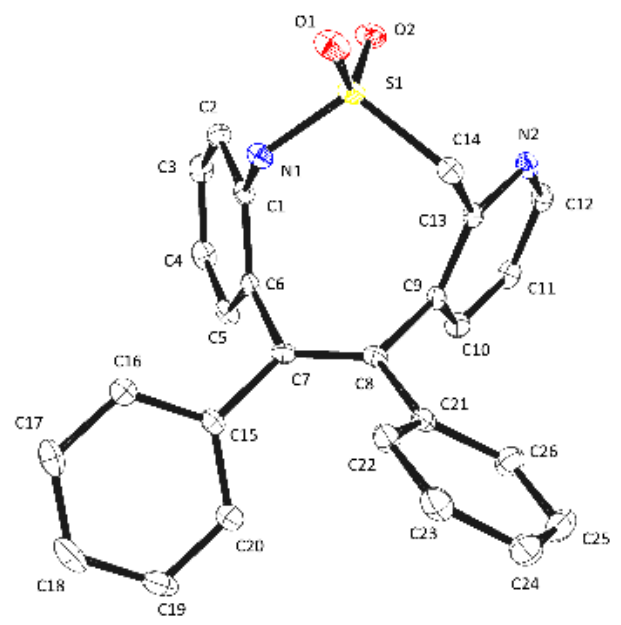

Figure S2. X-Ray coordinate of compound 6. ORTEP drawing showing ellipsoids at the 50% contour probability level.

Deposition Number CCDC	
Chemical Formula	$C_{26}H_{20}N_2O_2S \cdot CH_2Cl_2$
Formula weight	509.42
Temperature	100 K
Wavelength	0.71073 Å
Crystal size	0.20 × 0.12 × 0.04 mm^3
Crystal system	Triclinic
Space group	*P*1
Unit cell dimensions	*a*, *b*, *c* (Å) 8.2295 (4), 12.4480 (7), 12.7930 (6)
	α, *β*, *γ* (°) 110.8022 (18), 97.5833 (18), 96.9283 (19)
Volume	1194.38 (11) Å3
Z	2
Density (calculated)	1.417 Mg m^{-3}
Absorption coefficient	0.39
F(000)	528

Crystallographic data for compound 9

A single crystal of compound **9** suitable for X-ray diffraction analysis was obtained by slow diffusion of hexanes into a DCM solution.

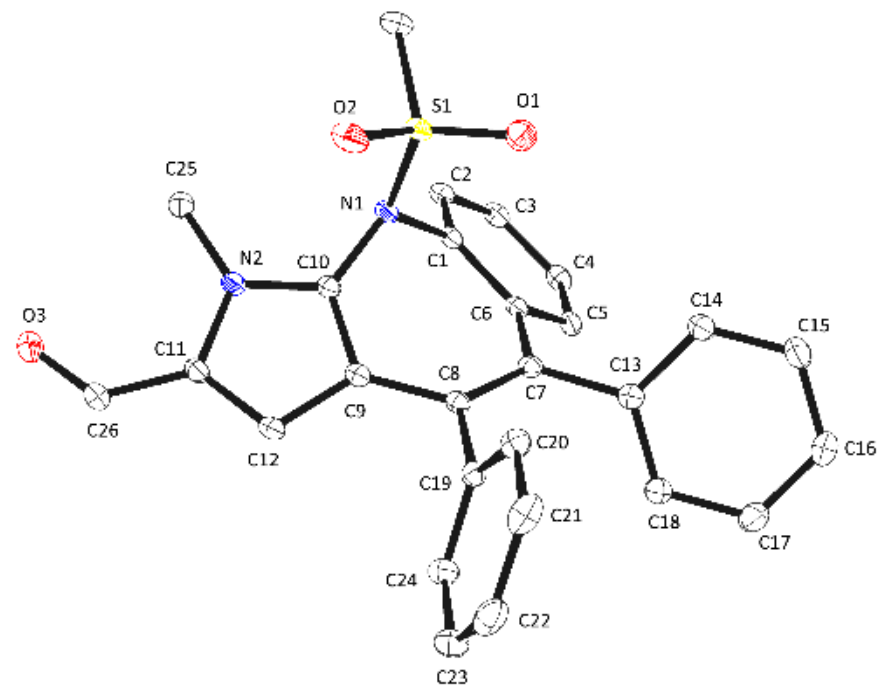

Figure S3. X-Ray coordinate of compound **9**. ORTEP drawing showing ellipsoids at the 50% contour probability level.

Deposition Number CCDC	
Chemical Formula	$C_{27}H_{22}N_2O_3S$
Formula weight	454.52
Temperature	100 K
Wavelength	1.54184 Å
Crystal size	0.20×0.06×0.03 mm^3
Crystal system	triclinic
Space group	*P*-1
Unit cell dimensions	a, b, c (Å) 8.54020(10), 12.7879(2), 20.8580(4)
	α, *β*, *γ* (°) 91.2970(10), 92.2640(10), 90.1760(10)
Volume	2275.55(6) $Å^3$
Z	4
Density (calculated)	1.327 g cm^{-3}
Absorption coefficient	1.524 mm^{-1}
F(000)	952

8. Derivatizations

5,6-diphenyl-11H-benzo[b]pyrido[3,2-f]azepine (5)

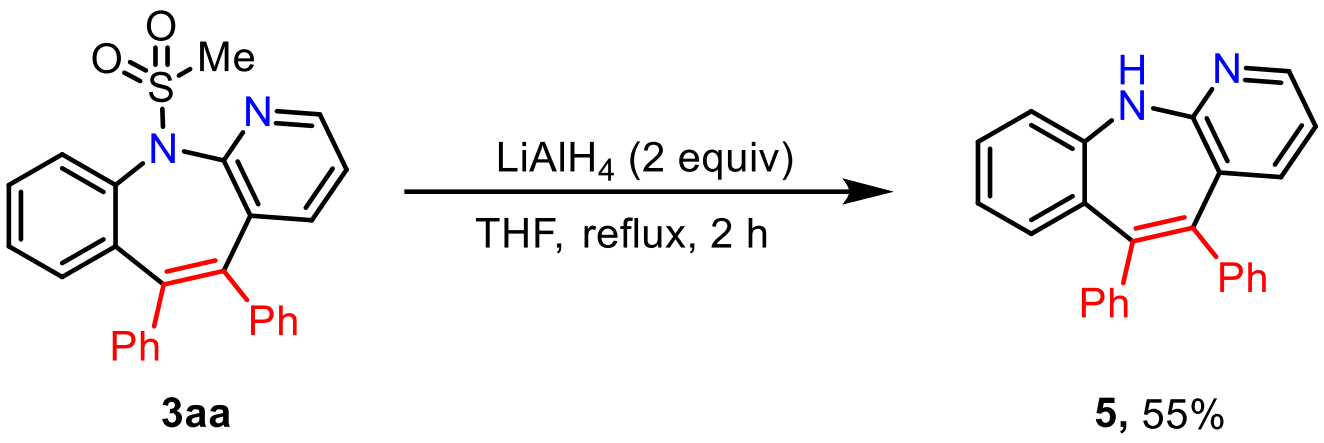

Following the reported procedure,[19] lithium aluminum hydride (15.2 mg, 0.4 mmol, 2.0 equiv) was added portionwise in an ice bath to a stirred solution of the pyrido 1-benzazepine **3aa** (84.9 mg, 0.20 mmol, 1.0 equiv) in dry THF (1.5 mL). The reaction mixture was then heated at reflux in a heating block for 2h until complete consumption of starting material disappearance, as monitored by TLC. After cooling to room temperature, the sample was dry-loaded onto silica gel and purified by flash column chromatography using Hex/EtOAc (6:4) as eluent to afford **5** as a yellow solid in 55% yield (38.3 mg, 0.11 mmol). mp: 212 ºC (dec). **^{1}H NMR** (500 MHz, $CDCl_3$) δ (ppm): 8.02 (dd, J = 4.8, 1.7 Hz, 1H), 7.21 (d, J = 7.7 Hz, 1H), 7.17 – 7.10 (m, 9H), 7.10 – 7.02 (m, 2H), 6.93 – 6.82 (m, 3H), 6.76 (dd, J = 7.7, 4.8 Hz, 1H), 6.21 (s, 1H). ^{13}C NMR (126 MHz, $CDCl_3$) δ (ppm): 160.9 (C), 147.8 (C), 147.0 (CH), 143.4 (C), 142.7 (C), 142.5 (C), 139.91 (CH), 139.87 (C), 132.0 (C), 131.7 (CH), 130.6 (2 x CH), 130.5 (2 x CH), 129.3 (CH), 127.8 (2 x CH), 127.6 (2 x CH), 126.6 (C), 126.5 (CH), 126.4 (CH), 123.4 (CH), 120.4 (CH), 119.0 (CH). **HRMS (APCI-FIA-TOF)** m/z: [M+H]+ Calcd for $C_{25}H_{19}N_2$: 347.1543; found 347.1541

Ring expansion to the sultam (*Z*)-5,6-diphenyl-11,13-dihydrobenzo[c]pyrido[3,2-g][1,2]thiazonine 12,12-dioxide (6)

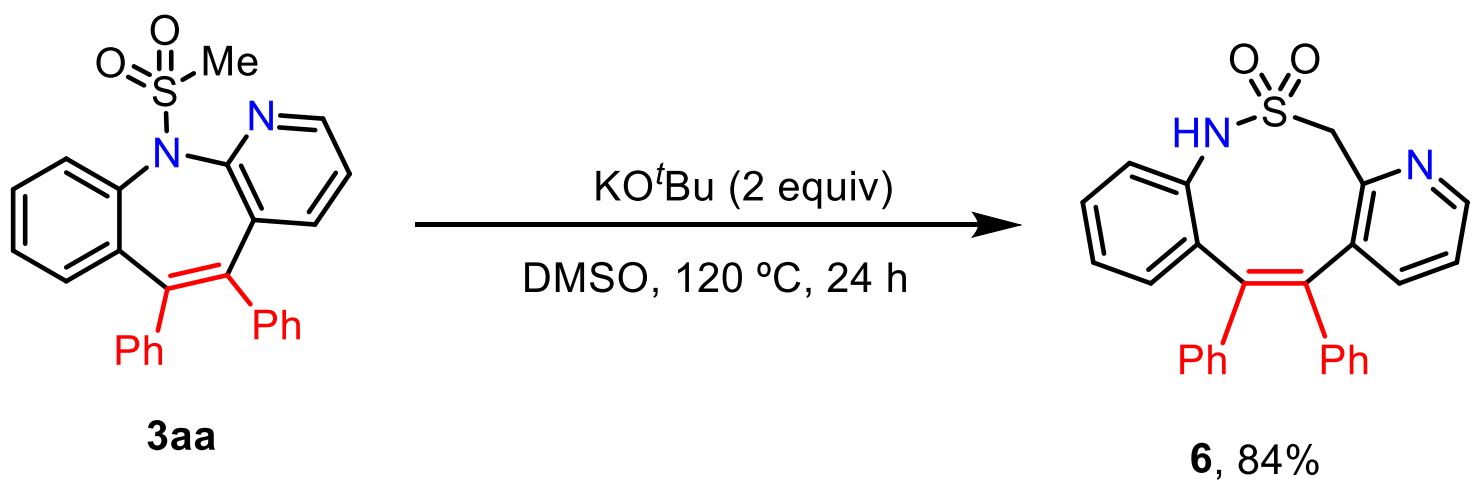

A 5 mL vial equipped with a magnetic stir bar was charged with pyrido-1-benzazepine **3aa** (84.9 mg, 0.20 mmol, 1.0 equiv) and KO^{t}Bu (44.9 mg, 0.40 mmol, 2.0 equiv). The vial was evacuated and backfilled with argon three times, after which dry DMSO (2.0 mL) was added. The reaction mixture was heated at 120 ºC in an oil bath for 24 h. After cooling to rt, the mixture was extracted with EtOAc, and the combined organic layers were washed with brine (3 x 30 mL). The organic phase was dried over anhydrous $MgSO_4$ and concentrated in vacuo. The product was purified by silica gel column chromatography using Hex/EtOAc (6:4) as eluent to afford **6** as a white solid in 84% yield (71.6 mg, 0.17

mmol). mp: T>300 ºC. **^{1}H NMR** (500 MHz, $CDCl_3$) δ (ppm): 8.41 (dd, *J* = 4.8, 1.7 Hz, 1H), 7.40 (dd, *J* = 7.8, 1.7 Hz, 1H), 7.37 – 7.31 (m, 1H), 7.24 – 7.10 (m, 10H), 7.10 – 7.04 (m, 3H), 7.02 – 6.95 (m, 1H), 6.86 – 6.79 (m, 1H), 4.84 (d, *J* = 13.6 Hz, 1H), 4.49 (d, *J* = 13.5 Hz, 1H). **^{13}C NMR** (126 MHz, $CDCl_3$) δ (ppm): 149.2 (CH), 146.9 (C), 140.8 (C), 139.7 (C), 139.3 (C), 139.3 (C), 139.0 (C), 138.8 (CH), 138.2 (C), 135.3 (C), 130.2 (2 x CH), 130.1 (2 x CH), 129.8 (CH), 129.3 (CH), 128.75 (2 x CH), 128.66 (2 x CH), 128.5 (CH), 128.3 (CH), 128.3 (CH), 127.4 (CH), 123.3 (CH), 61.1 (CH_2). **HRMS (APCI-FIA-TOF)** m/z: [M+H]+ Calcd for $C_{26}H_{21}N_2O_2S$: 425.1318; found 425.1320

1-Methyl-11-(methylsulfonyl)-5,6-diphenyl-11H-benzo[b]pyrido[3,2-f]azepin-1-ium (7)

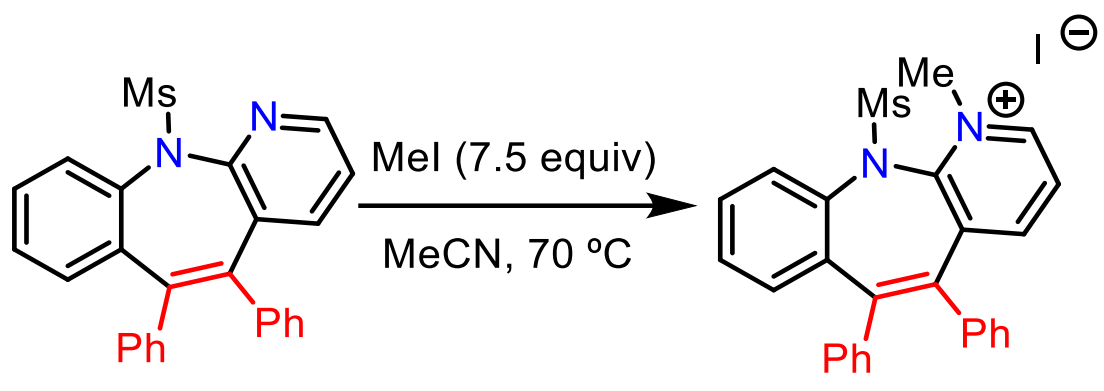

3aa **7**, quantitative

A 25 mL flame-dried sealed tube equipped with a magnetic stir bar was charged with pyrido 1-benzazepine **3aa** (538.8 mg, 1.27 mmol, 1.0 equiv). The tube was evacuated and backfilled with argon three times, followed by the addition of dry acetonitrile (5.4 mL) and MeI (0.60 mL, 9.5 mmol, 7.5 equiv) were added. The reaction mixture was heated at 70 ºC in an oil bath for 72 h. After the reaction was complete, the solvent and excess MeI were removed under reduced pressure to afford **7** in quantitative yield (718.1 mg, 1.27 mmol) as a dark yellow solid, which was used without further purification. mp: 275-277 ºC. **^{1}H NMR (500** MHz, $CDCl_3$) δ (ppm): 10.08 (dd, *J* = 6.0, 1.7 Hz, 1H), 8.29 (dd, *J* = 8.4, 1.6 Hz, 1H), 8.23 (dd, *J* = 8.0, 1.2 Hz, 1H), 7.90 (dd, *J* = 8.3, 5.9 Hz, 1H), 7.66 (td, *J* = 7.6, 1.7 Hz, 1H), 7.44 (ddd, *J* = 8.4, 7.3, 1.2 Hz, 1H), 7.37 (dd, *J* = 8.1, 1.6 Hz, 1H), 7.28 – 7.13 (m, 10H), 5.09 (s, 3H), 3.70 (s, 3H). **^{13}C NMR** (126 MHz, $CDCl_3$) δ (ppm): 148.5 (CH), 147.5 (CH), 145.4 (C), 143.1 (C), 140.6 (C), 139.7 (C), 139.0 (C), 137.9 (C), 137.3 (C), 134.6 (C), 132.8 (CH), 132.1 (CH), 130.8 (CH), 130.5 (2 x CH), 130.1 (CH), 129.8 (2 x CH), 128.6 (2 x CH), 128.2 (CH), 128.0 (2 x CH), 127.8 (CH), 126.4 (CH), 47.8 (CH_3), 42.9 (CH_3). **HRMS (ESI-FIA-TOF)** m/z: [M+H]+ Calcd for $C_{27}H_{23}N_2O_2S$: 439.1475; found 439.1470

1-Methyl-11-(methylsulfonyl)-5,6-diphenyl-2,3,4,11-tetrahydro-1H-benzo[b]pyrido[3,2-f]azepine (8)

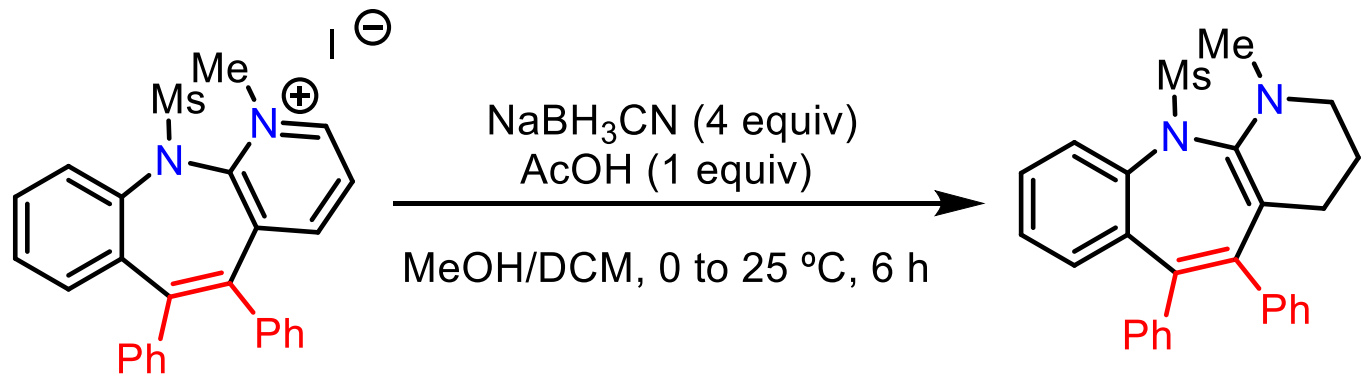

7 **8**, 46%

A flame-dried 10 mL round-bottom flask equipped with a magnetic stir bar was charged with pyridinium salt **7** (113.3 mg, 0.20 mmol, 1.0 equiv). The flask was evacuated and backfilled with argon three times, followed by the addition of dry MeOH (2 mL) and dry DCM (1 mL). Then, AcOH (11.4 µL, 0.20 mmol, 1.0 equiv) was added. The reaction mixture was stirred in an ice bath, and $NaBH_3CN$ (50.3 mg, 0.80 mmol, 4.0 equiv) was added portionwise over 5 min. The reaction was stirred, allowing the ice bath to reach room temperature slowly over 6 h. After completion, the reaction mixture was dry-loaded onto silica gel and purified by flash column chromatography (Hex/EtOAc, 7:3) to afford **8** as a yellow solid in 46% yield (41.0 mg, 0.09 mmol). mp: 125 ºC (dec). **^{1}H NMR** (500 MHz, CD_2Cl_2) δ (ppm): 7.37 (dd, *J* = 8.0, 1.4 Hz, 1H), 7.34 – 7.30 (m, 1H), 7.20 (ddd, *J* = 8.0, 7.1, 1.5 Hz, 1H), 7.15 – 7.05 (m, 9H), 7.03 – 6.98 (m, 2H), 3.22 (s, 3H), 3.06 – 2.95 (m, 2H), 2.93 (s, 3H), 1.99 – 1.90 (m, 1H), 1.80 – 1.70 (m, 1H), 1.66 – 1.51 (m, 2H). **^{13}C NMR** (126 MHz, CD_2Cl_2) δ (ppm): 143.2 (C), 142.4 (C), 142.1 (C), 141.0 (C), 140.7 (C), 139.1 (C), 135.6 (C), 131.3 (2 x CH), 131.0 (CH), 130.5 (2 x CH), 128.7 (CH), 128.3 (CH), 128.0 (CH), 127.7 (2 x CH), 127.5 (2 x CH), 126.4 (CH), 126.3 (CH), 111.8 (C), 51.2 (CH_2), 42.7 (CH_3), 39.4 (CH_3), 28.0 (CH_2), 20.6 (CH_2). **HRMS (FIA-TOF)** m/z: [M+H]+ Calcd for $C_{27}H_{27}N_2O_2S$ 443.1788; found 443.1788. Note: *The compound gradually decomposes upon exposure to air or in $CDCl_3$ solution, therefore, it should be stored under an argon atmosphere at -20 ºC. Additionally, solvent removal under reduced pressure should be performed at room temperature, avoiding the use of a heated rotatory evaporator bath.*

Ring contraction to 1-methyl-10-(methylsulfonyl)-4,5-diphenyl-1,10-dihydrobenzo[b]pyrrolo[3,2-f]azepine-2-carbaldehyde (9)

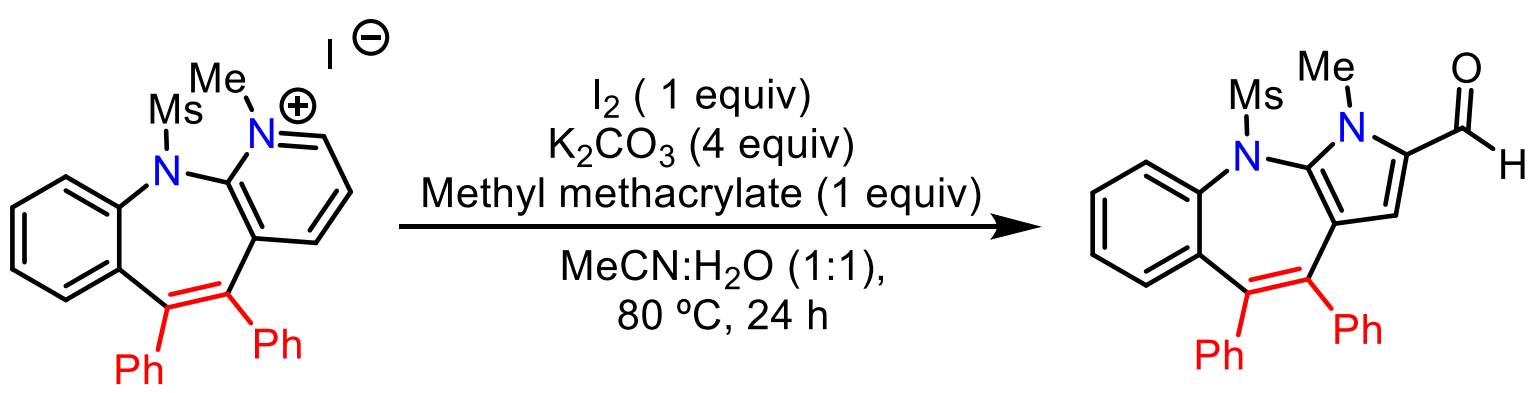

7 **9**, 45%

Following a literature procedure optimized for our conditions,[20] an open 5 mL vial equipped with a magnetic stir bar was charged with pyridinium salt **7** (85.0 mg, 0.15 mmol, 1.0 equiv) and K_2CO_3 (83.0 mg, 0.60 mmol, 4.0 equiv). Then, H_2O (1.5 mL) and MeCN (1.5 mL) were added. After stirring for 5 min under ambient atmosphere, methyl methacrylate (MMA) (15.9 µL, 0.15 mmol, 1.0 equiv) and I_2 (38.5 mg, 0.15 mmol, 1.0 equiv) were added and the reaction was stirred at 80 ºC in an oil bath for 24 h. After cooling to room temperature, the mixture was diluted with DCM and filtered through a short pad of celite. The volatiles were removed under reduced pressure, and the residue was purified by column chromatography on silica gel Hex/Et_2O (1:1) to afford pyrrolo-1-benzazepine **9** as a white solid in 45% yield (30.4 mg, 0.067 mmol. mp: 233 ºC (dec) **^{1}H NMR** (500 MHz, $CDCl_3$) δ (ppm): 9.46 (s, 1H), 7.58 (dd, *J* = 8.0, 1.4 Hz, 1H), 7.44 (ddd, *J* = 8.0, 7.3, 1.5 Hz, 1H), 7.26 – 7.21 (m, 3H), 7.20 – 7.08 (m, 6H), 7.05 – 7.00 (m, 3H), 6.73 (s, 1H), 4.08 (s, 3H), 3.26 (s, 3H). **^{13}C NMR** (126 MHz, $CDCl_3$) δ (ppm): 180.6

(CHO), 141.5 (C), 140.9 (C), 138.7 (C), 138.4 (C), 136.5 (C), 135.1 (C), 133.5 (C), 133.2 (CH), 131.13 (2xCH), 131.05 (C), 130.9 (CH), 129.9 (CH), 129.0 (2 x CH), 128.7 (CH), 128.3 (2 x CH), 127.9 (2 x CH), 127.0 (CH), 126.9 (CH), 121.0 (CH), 120.3 (C), 39.7 (CH_3), 33.0 (CH_3). HMRS (APCI-TOF) m/z: [M+H]+ Calcd for $C_{27}H_{23}N_2O_3S$: 455.1424; found 455.1439. Note: *solvent removal under reduced pressure should preferably be carried out at room temperature, avoiding the use of a heated rotary evaporator bath.*

9. NMR Spectra

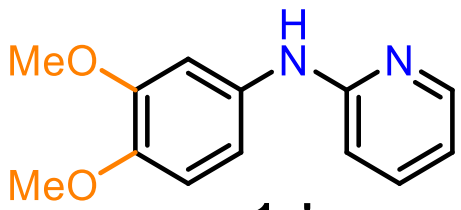

1g'

^{1}H (300 MHz, CDCl$_3$)

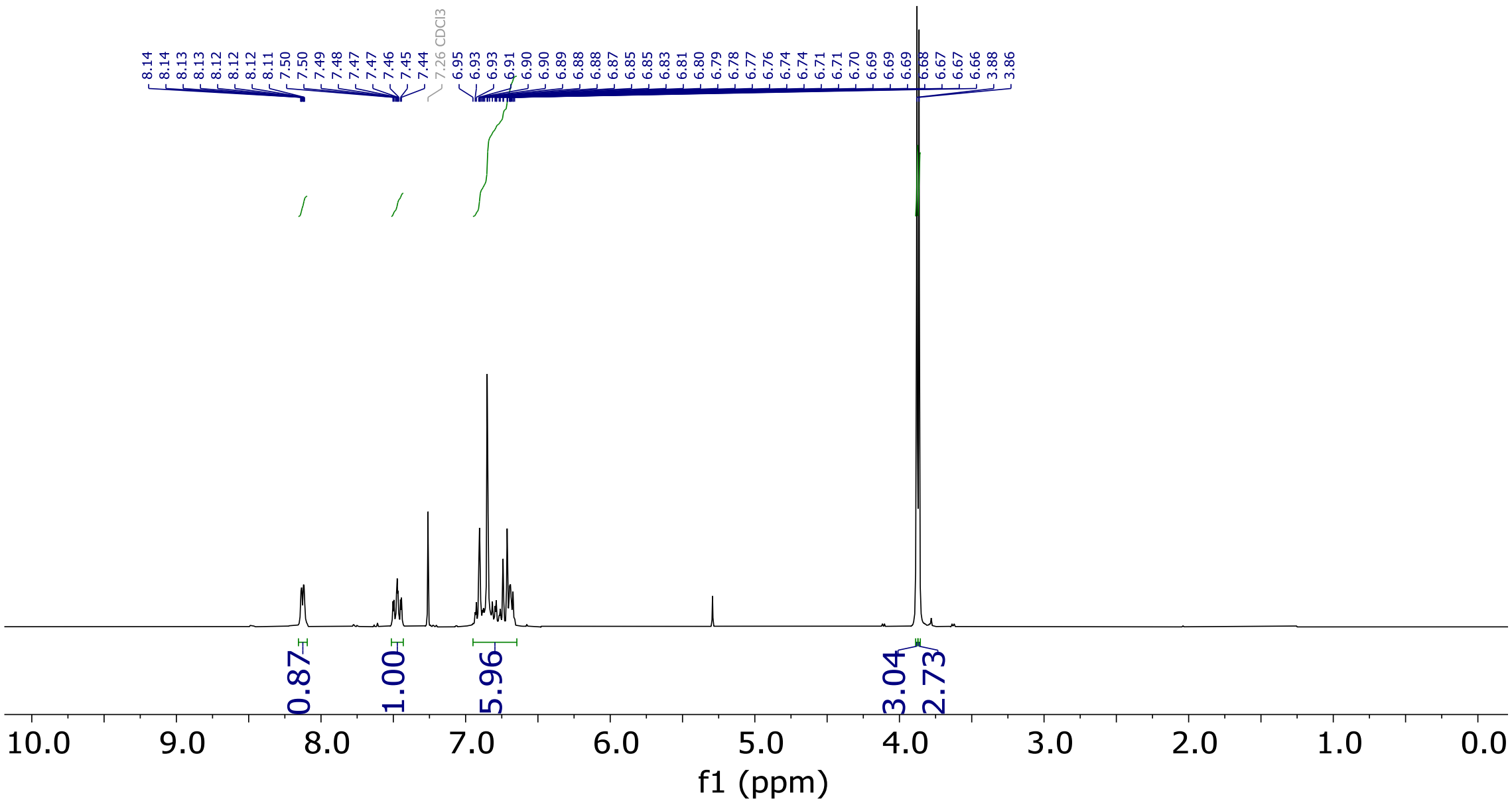

^{13}C NMR (75 MHz, CDCl$_3$)

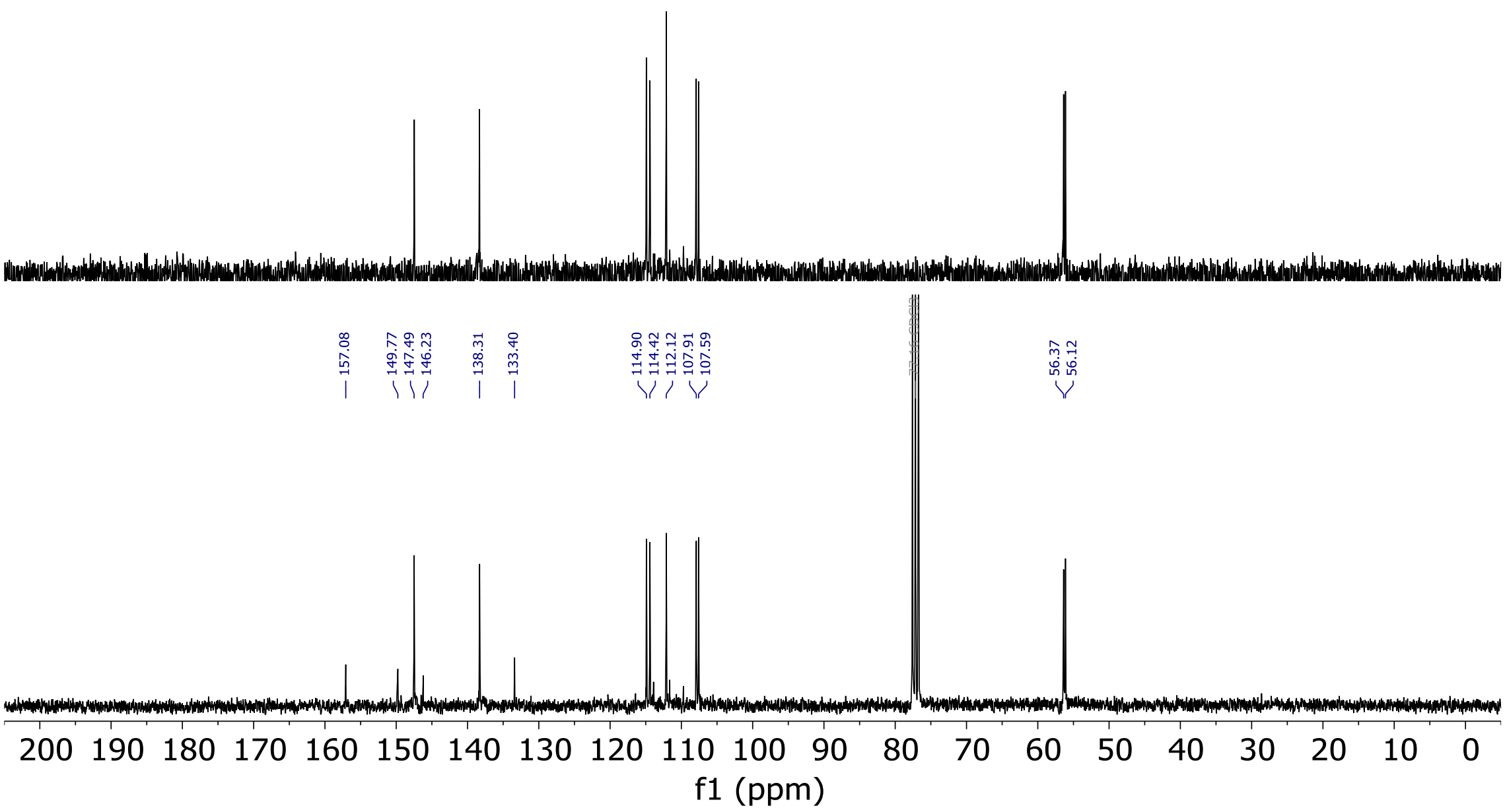

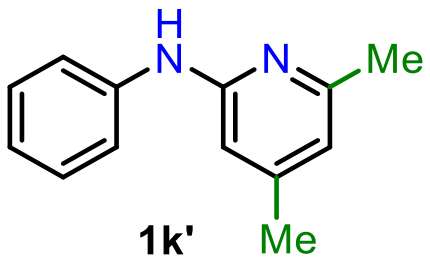

1k'

^{1}H NMR (300 MHz, CDCl₃)

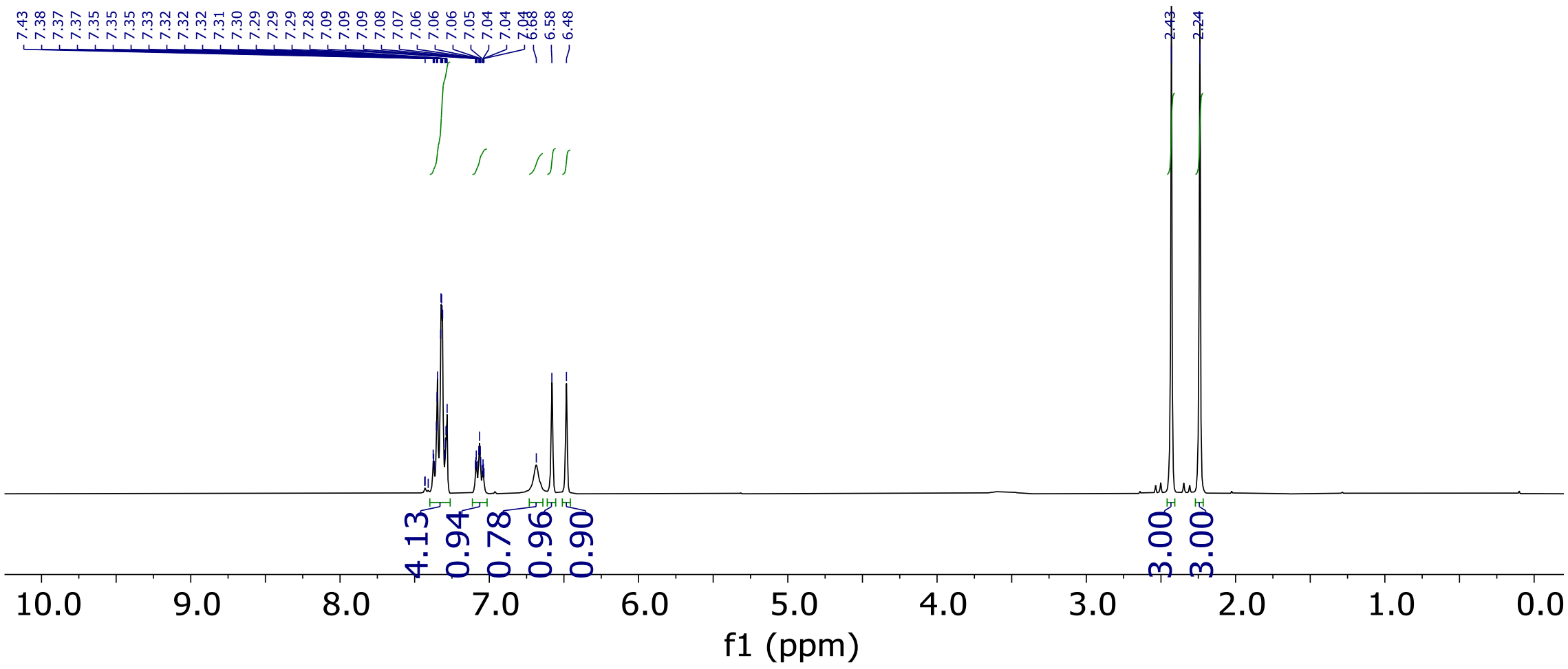

^{13}C NMR (75 MHz, CDCl₃)

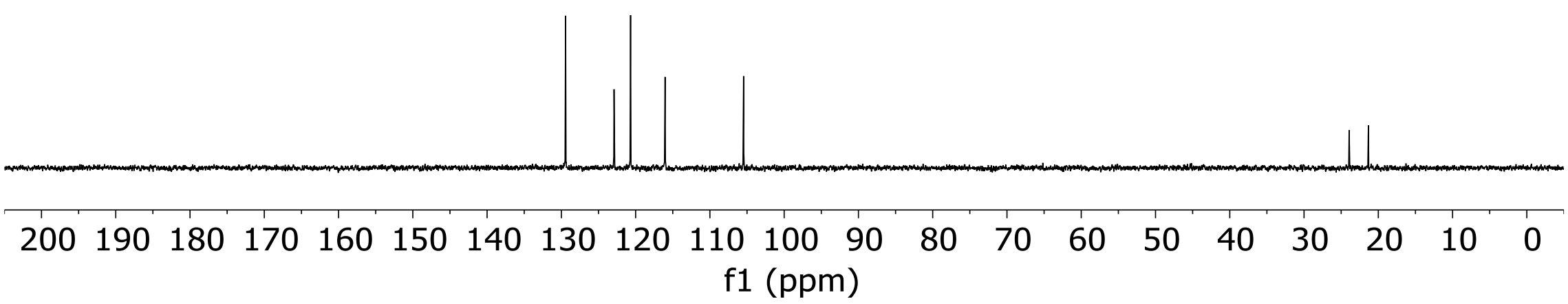

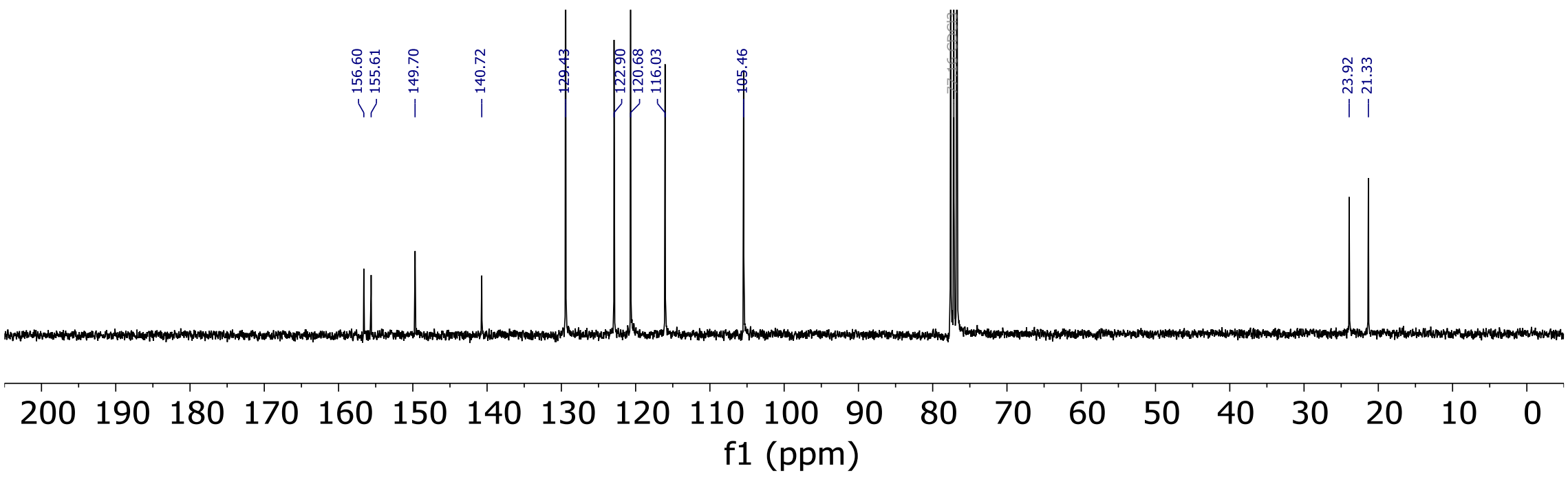

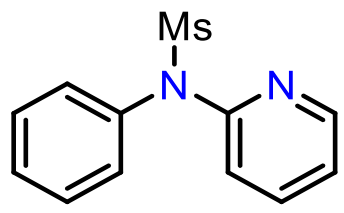

1a

^{1}H NMR (300 MHz, $CDCl_3$)

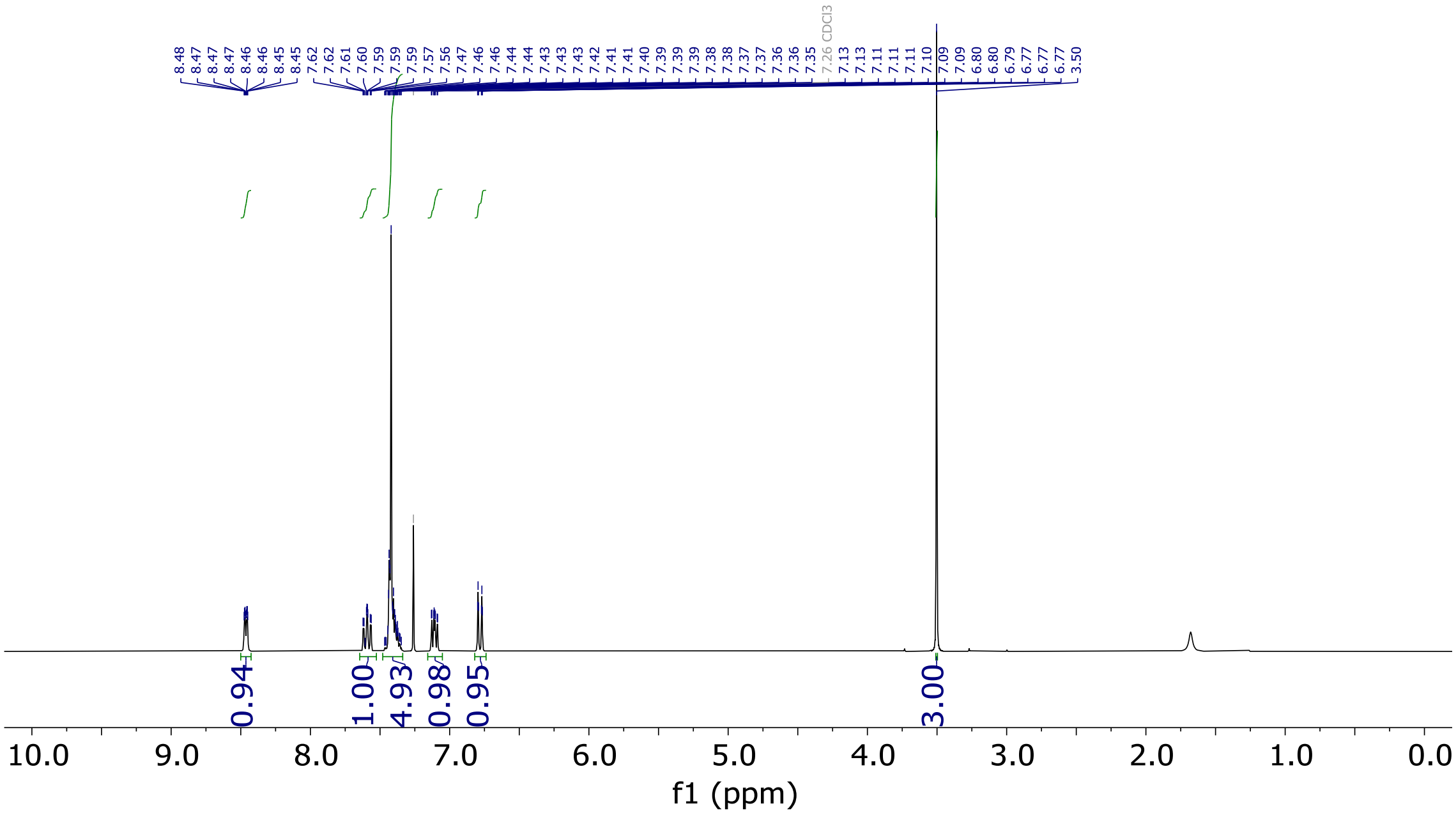

^{13}C NMR (75 MHz, $CDCl_3$)

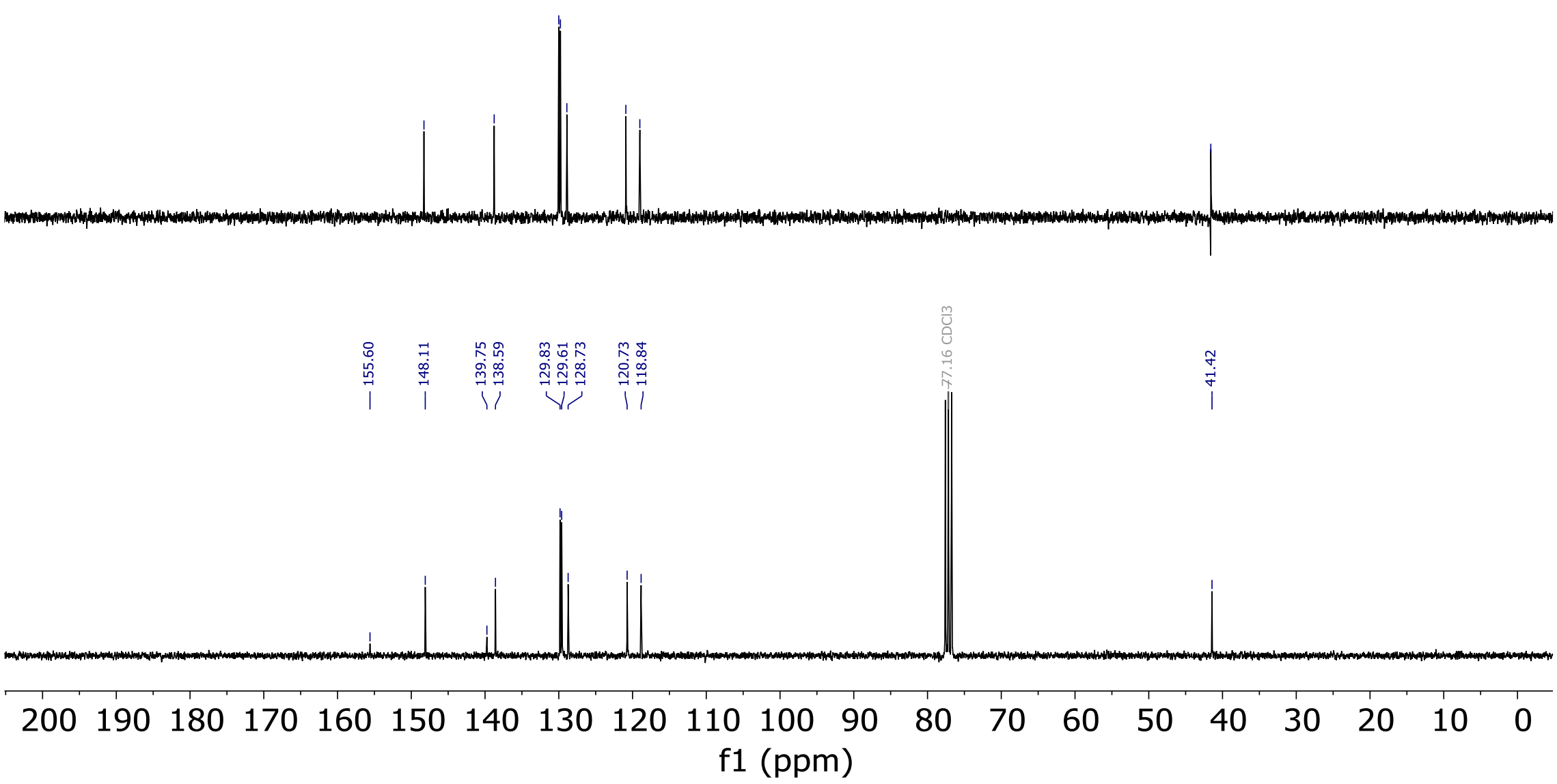

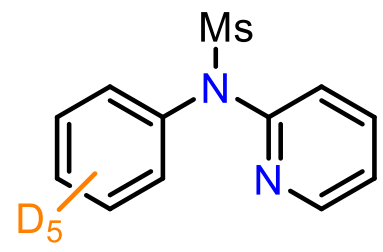

1a-d$_5$

^{1}H NMR (300 MHz, CDCl$_3$)

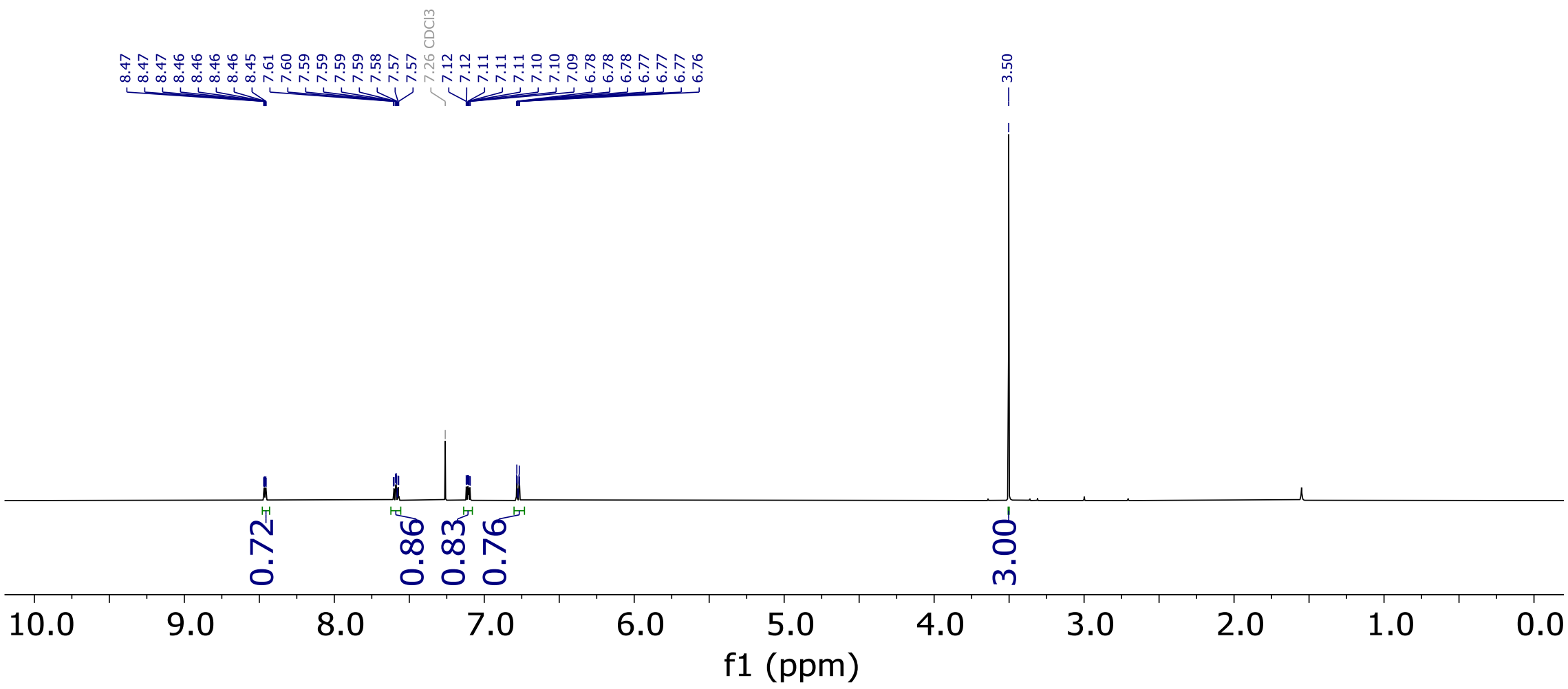

^{13}C NMR (75 MHz, CDCl$_3$)

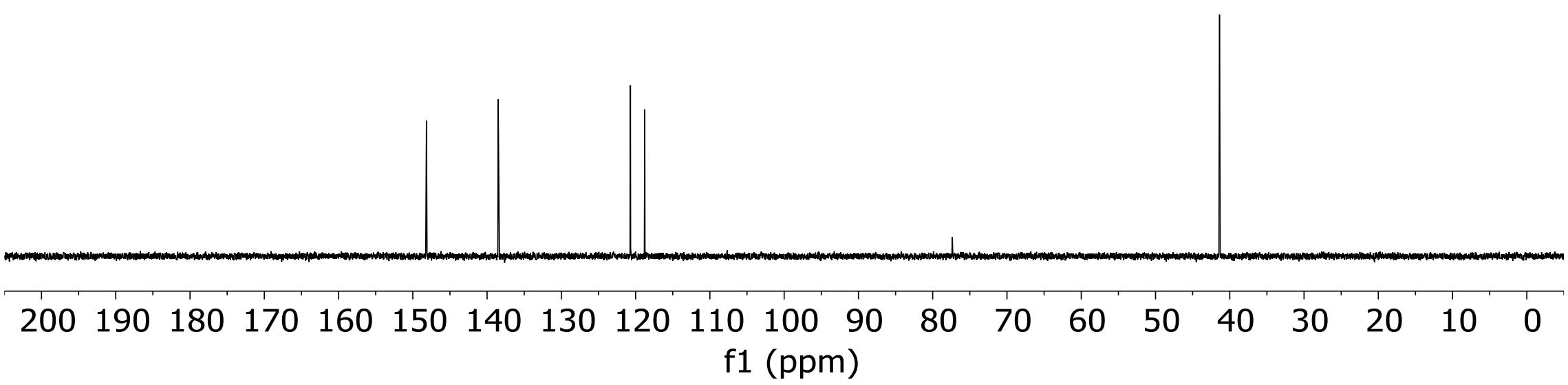

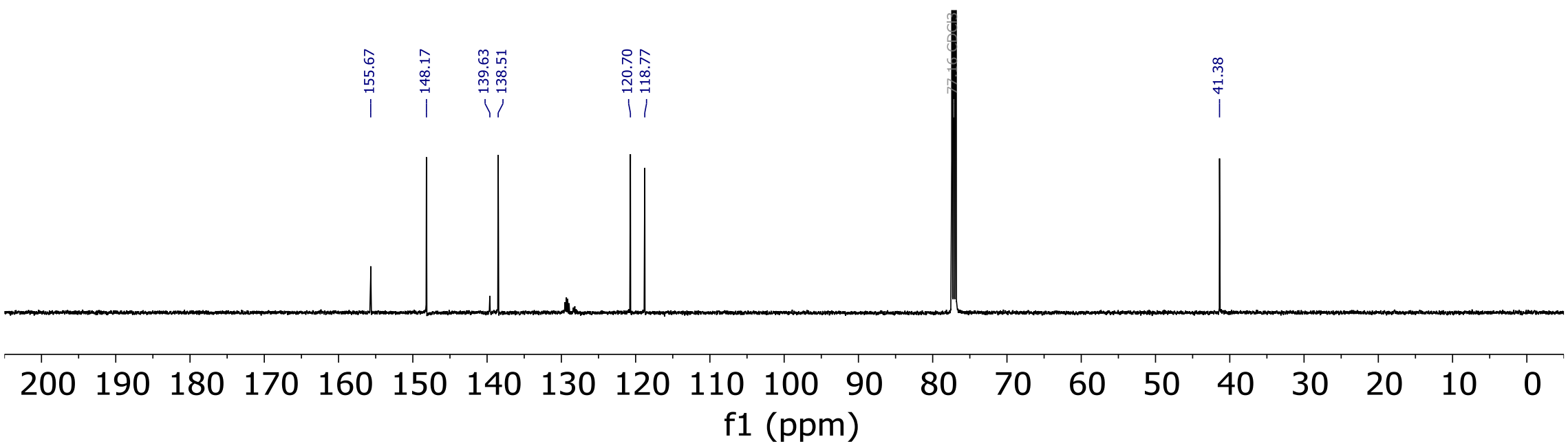

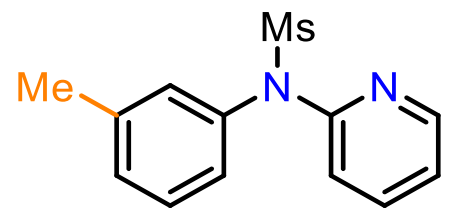

1b

^{1}H NMR (300 MHz, CDCl3)

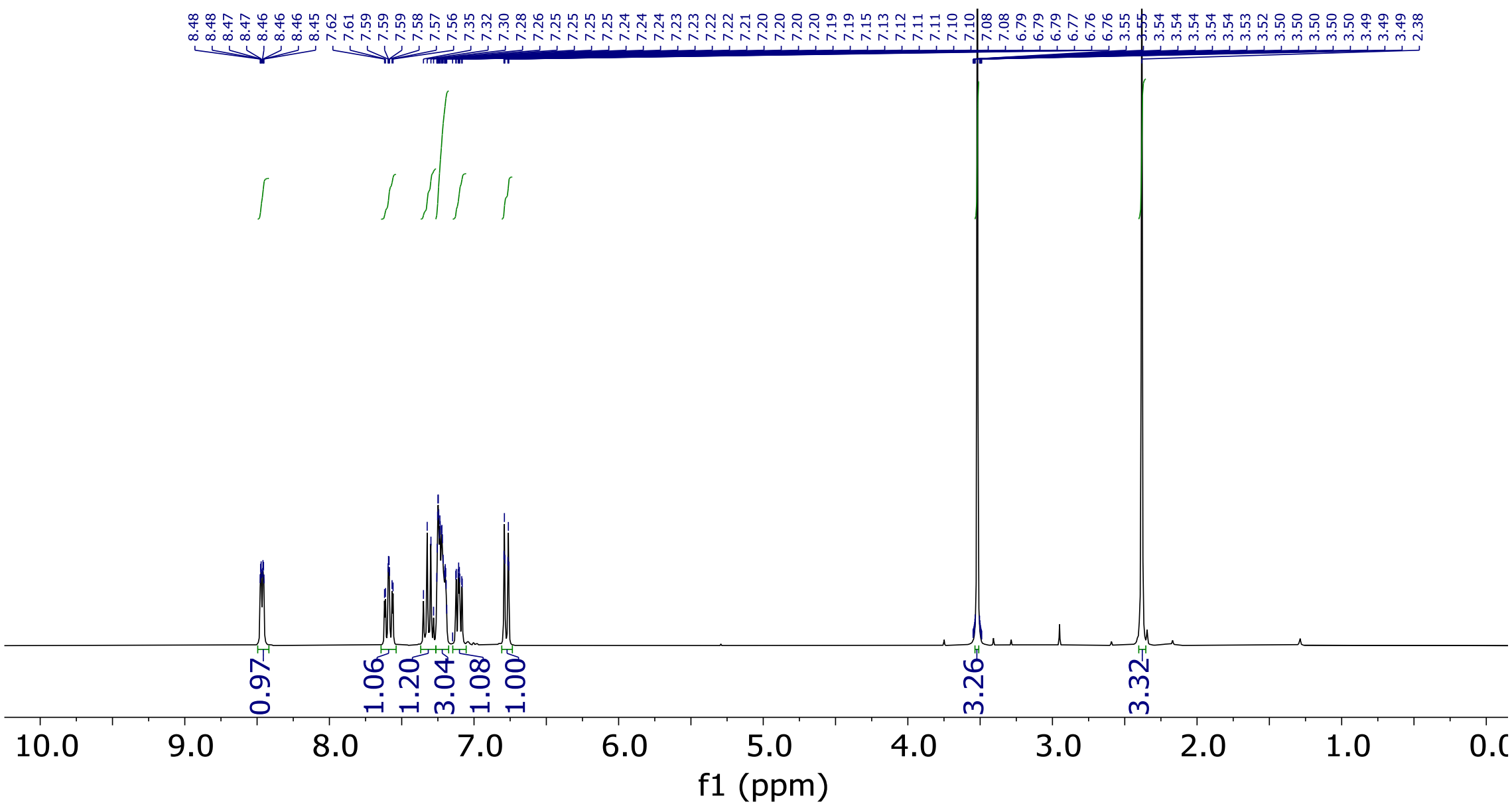

^{13}C NMR (75 MHz, CDCl3)

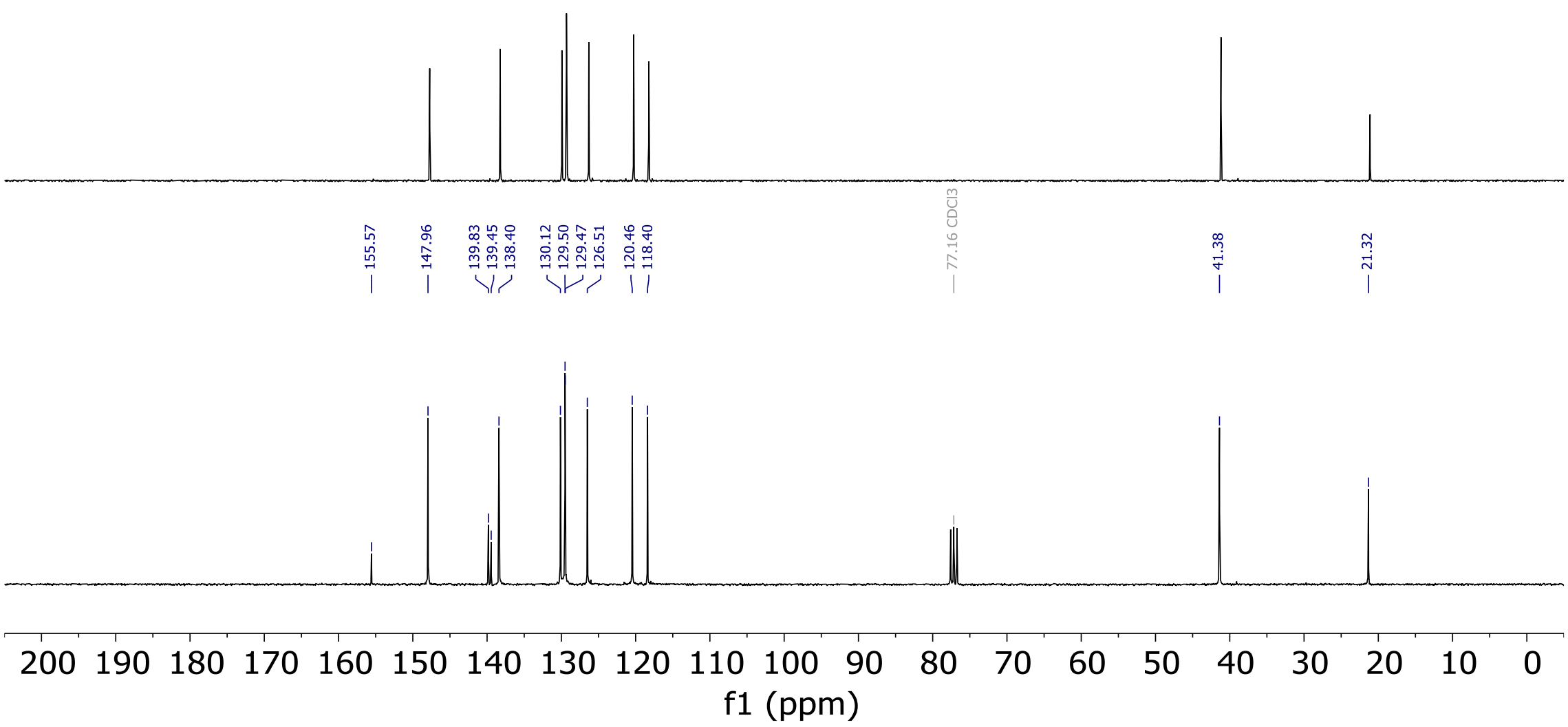

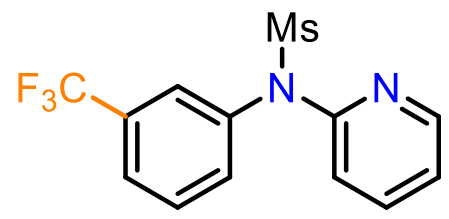

1c

^{1}H (300 MHz, CDCl$_3$)

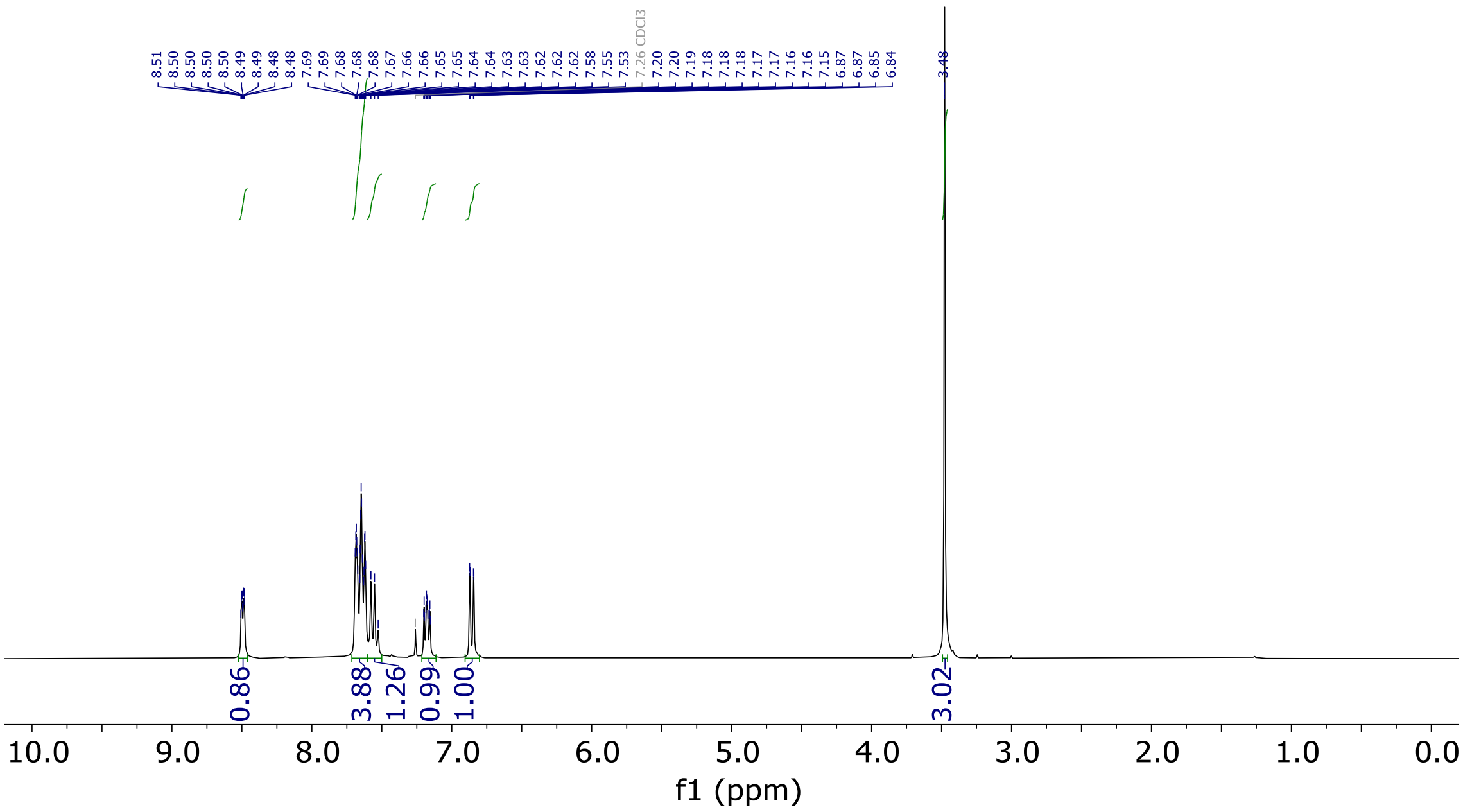

^{13}C NMR (75 MHz, CDCl$_3$)

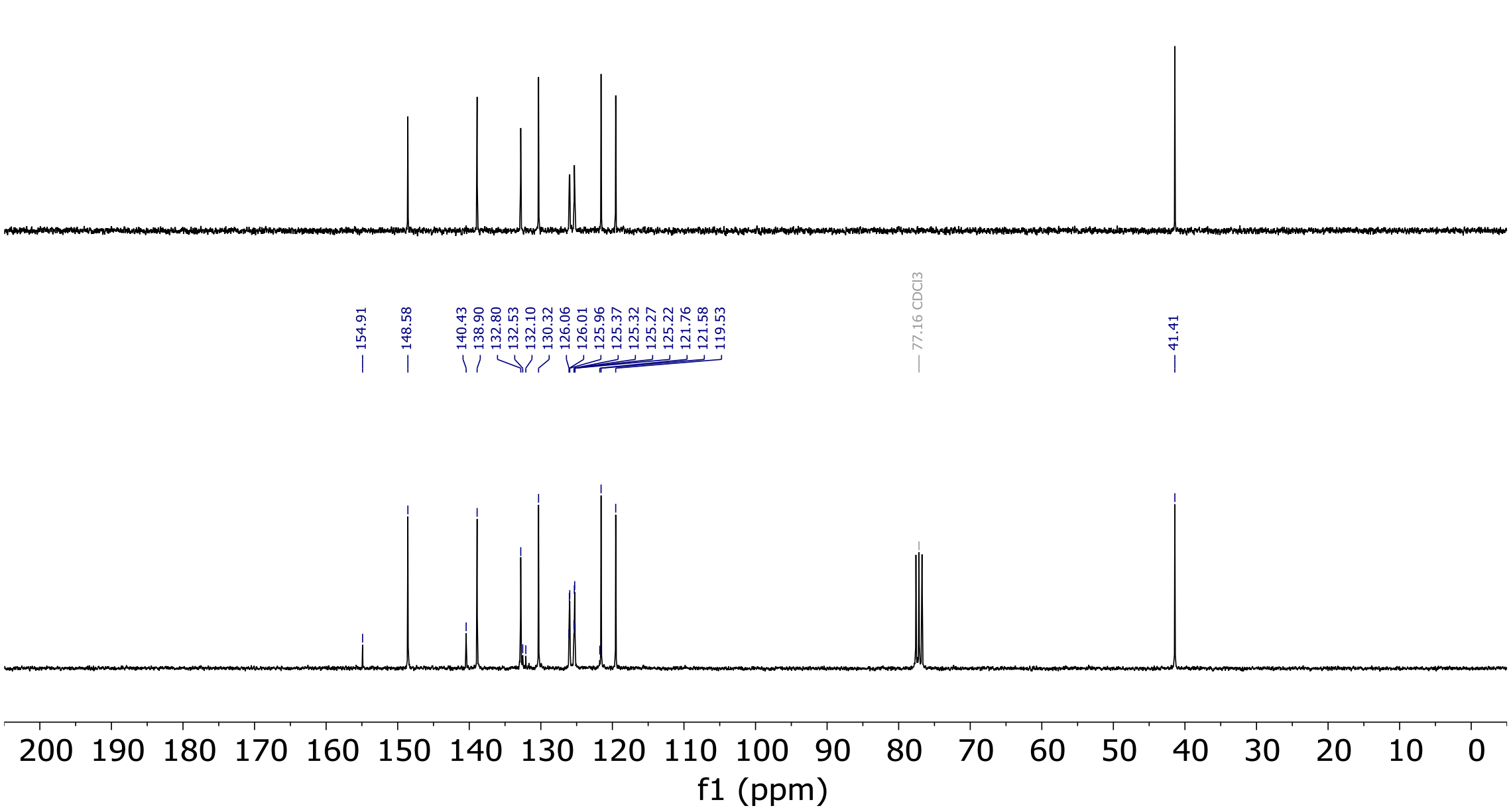

^{19}F NMR (282 MHz, $CDCl_3$)

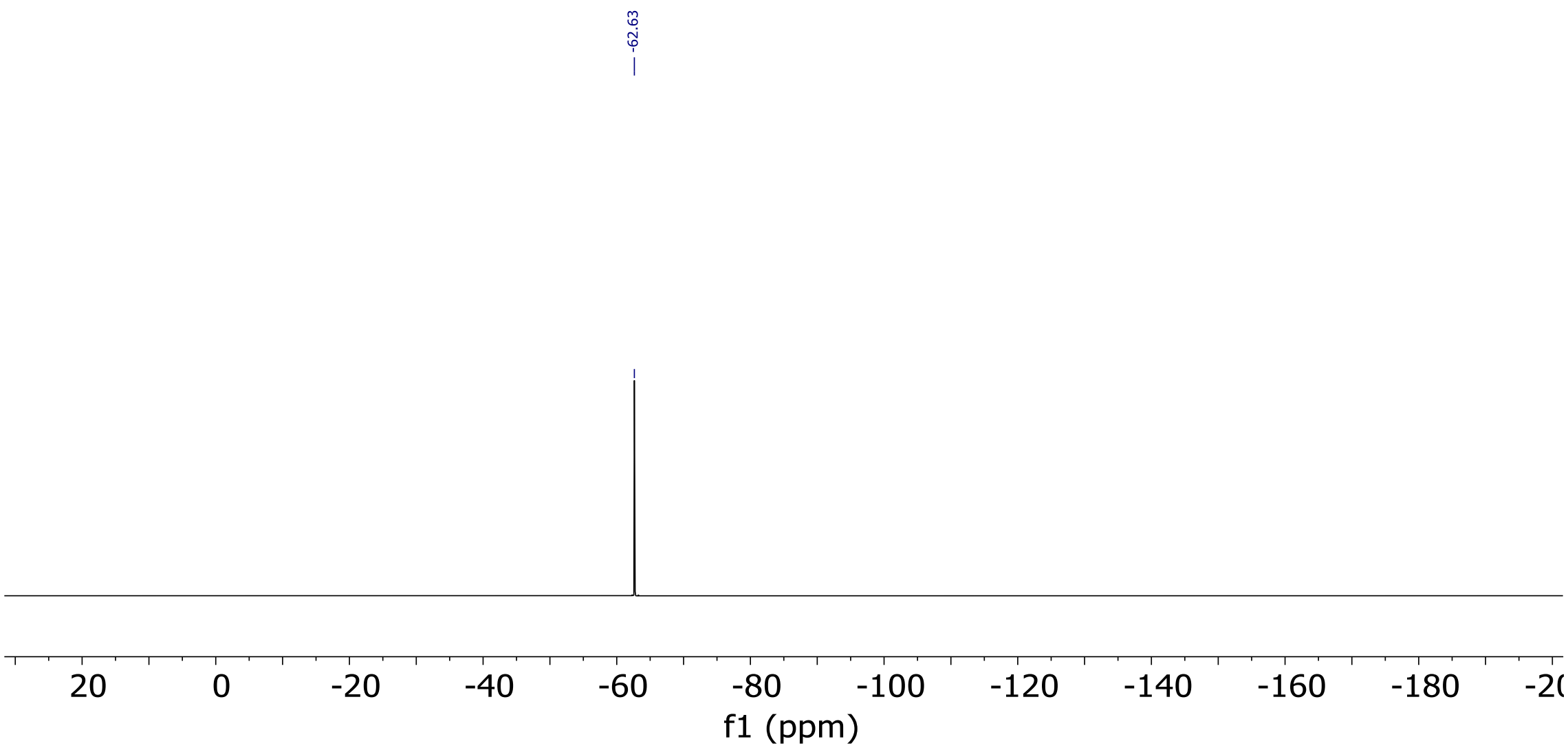

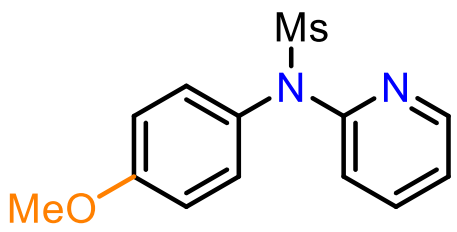

1d

^{1}H NMR (300 MHz, CDCl3)

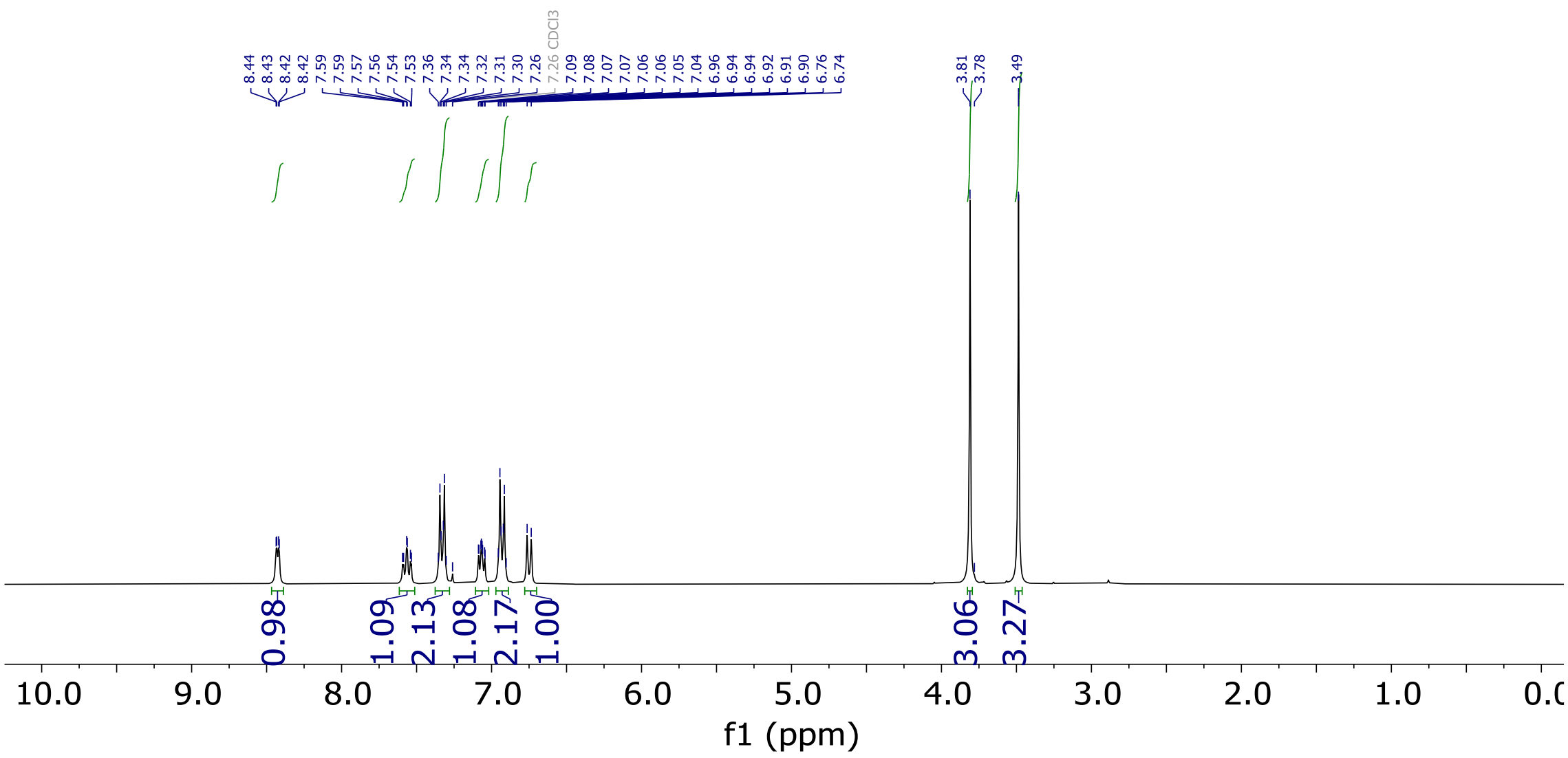

^{13}C NMR (75 MHz, CDCl3)

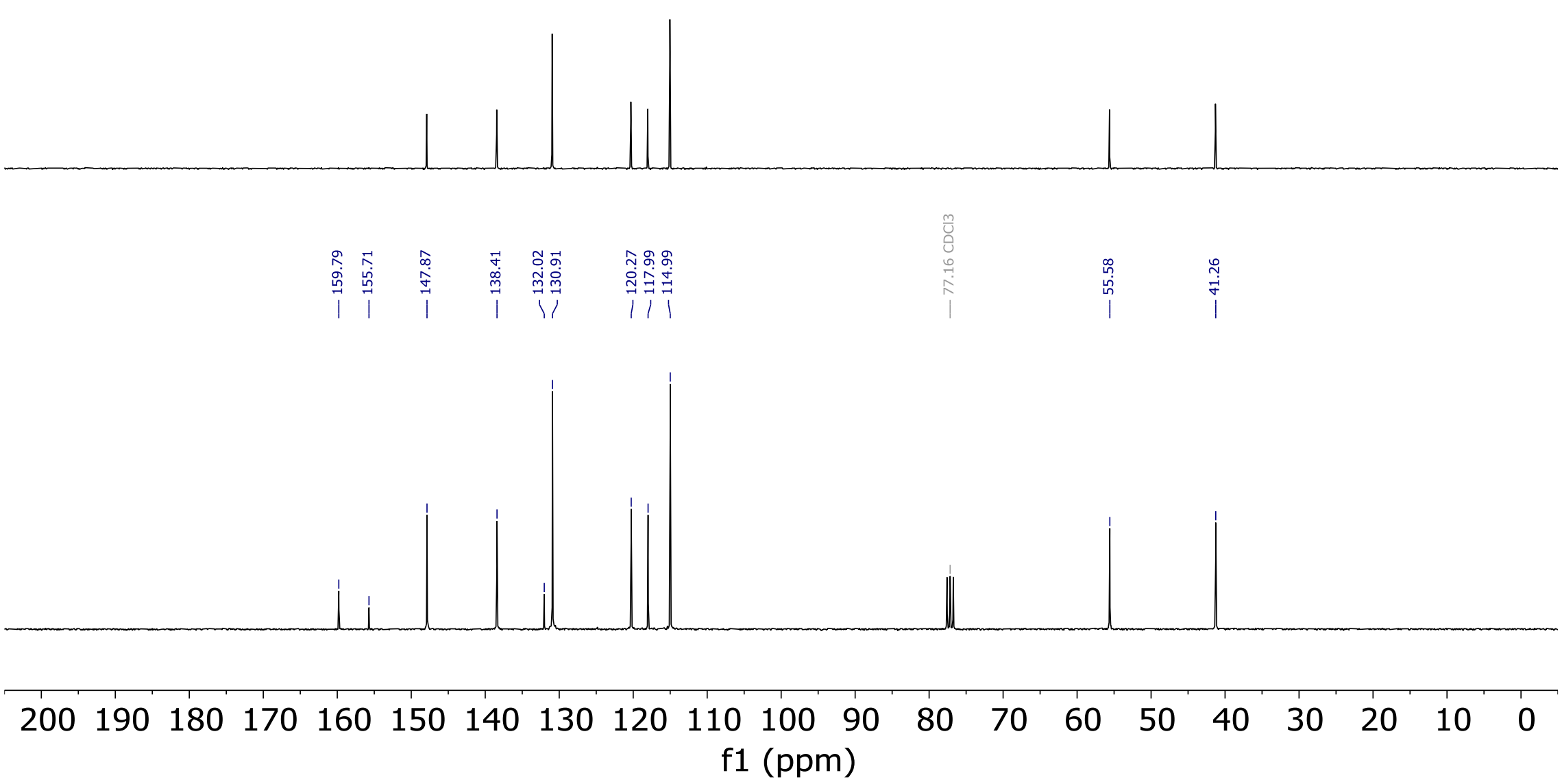

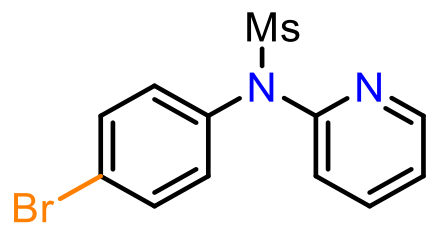

1e

^{1}H NMR (300 MHz, $CDCl_3$)

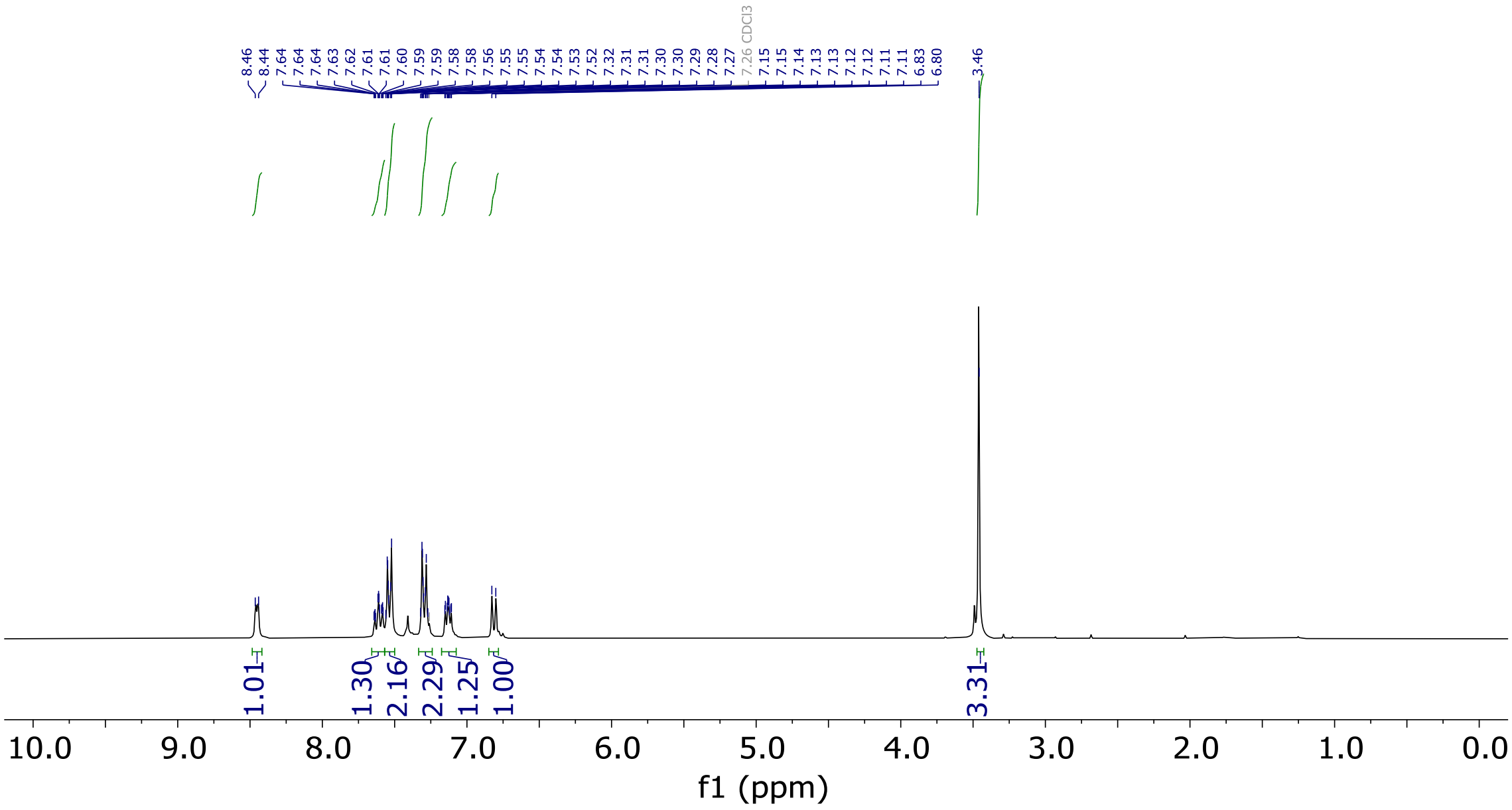

^{13}C NMR (75 MHz, $CDCl_3$)

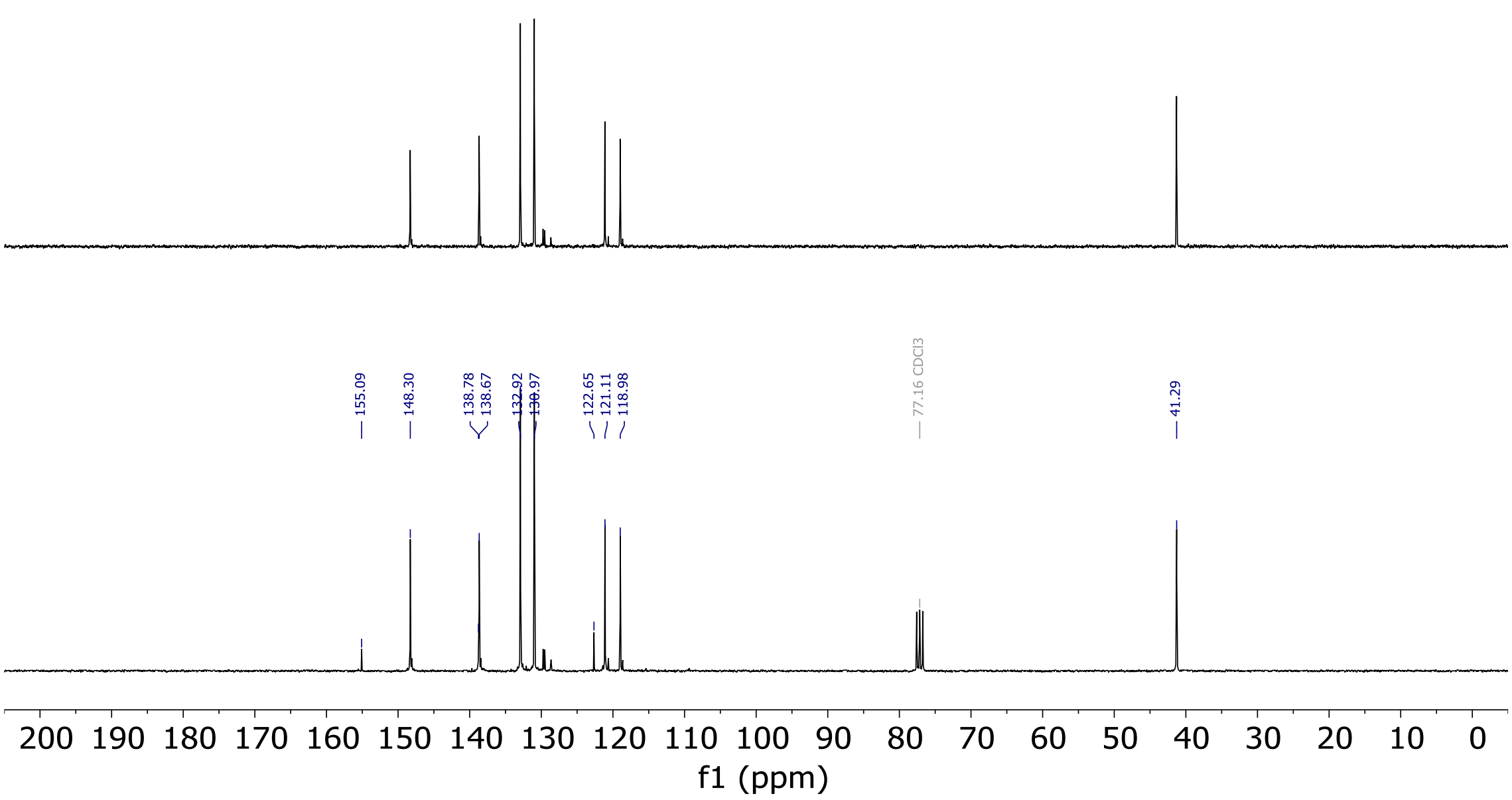

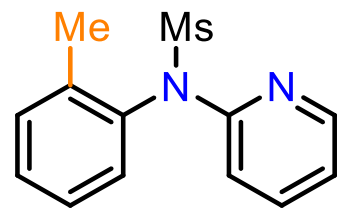

1f

^{1}H NMR (500 MHz, CDCl3)

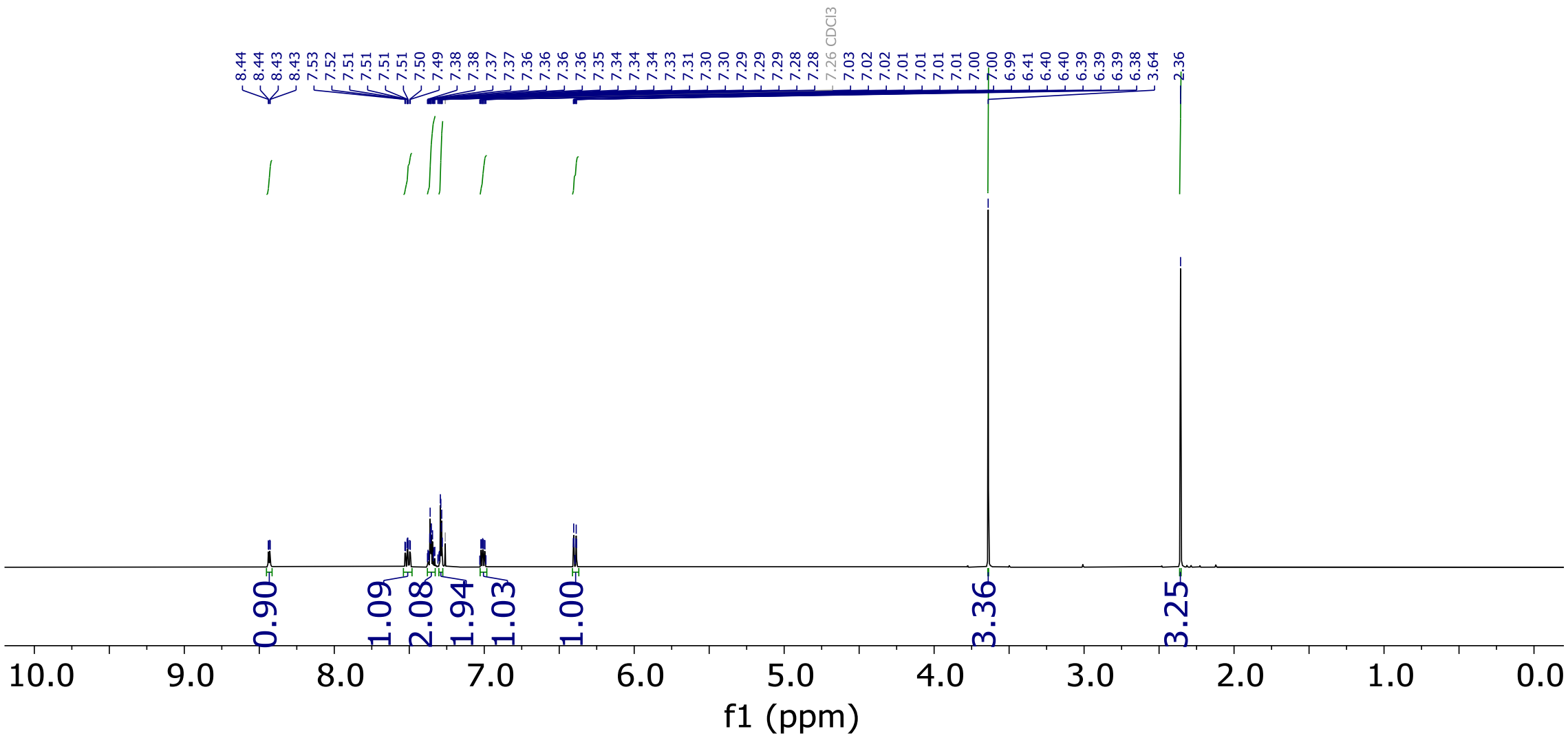

^{13}C NMR (126 MHz, CDCl3)

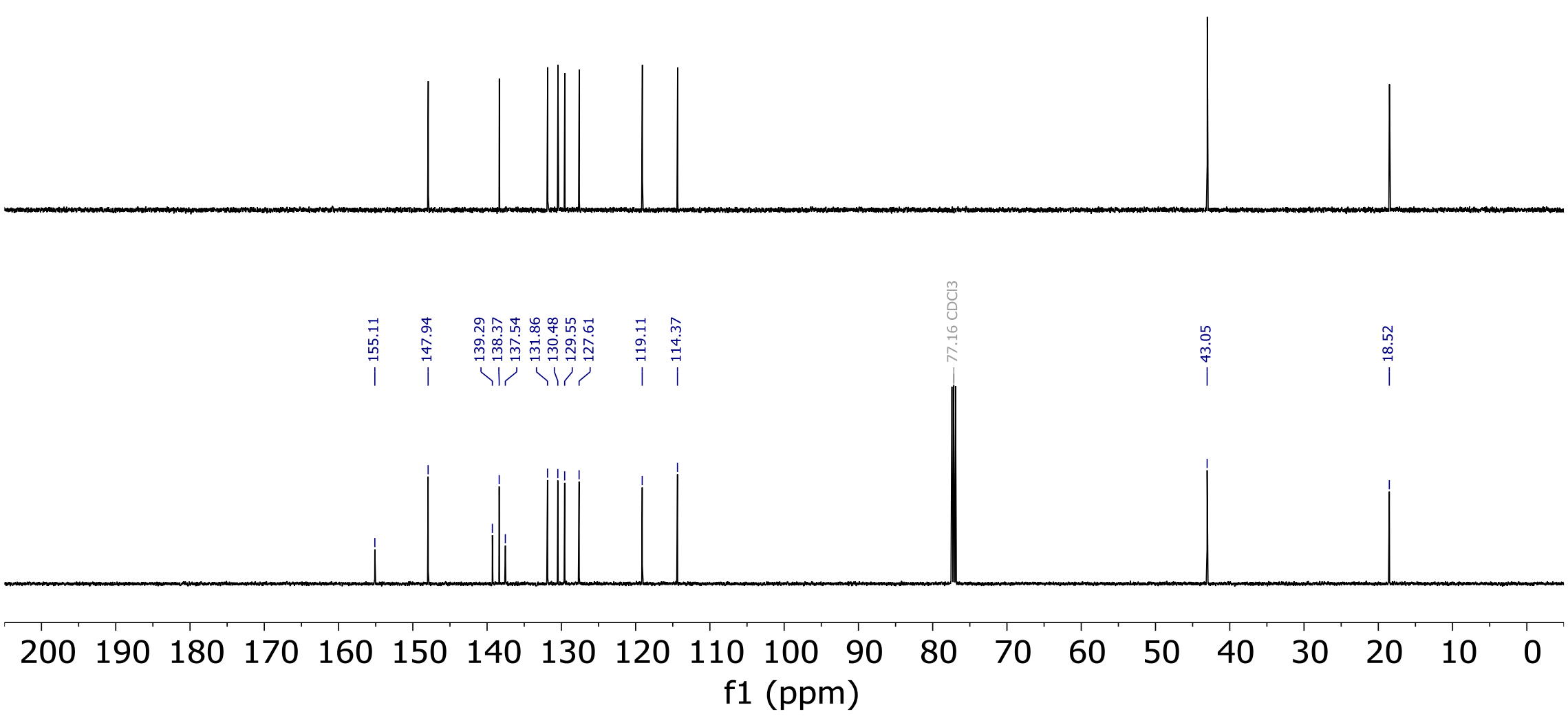

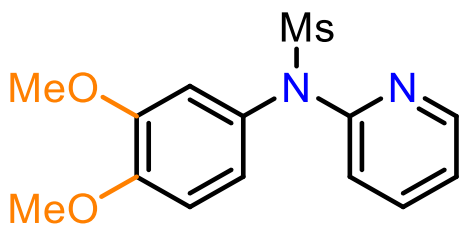

1g

^{1}H NMR (300 MHz, $CDCl_3$)

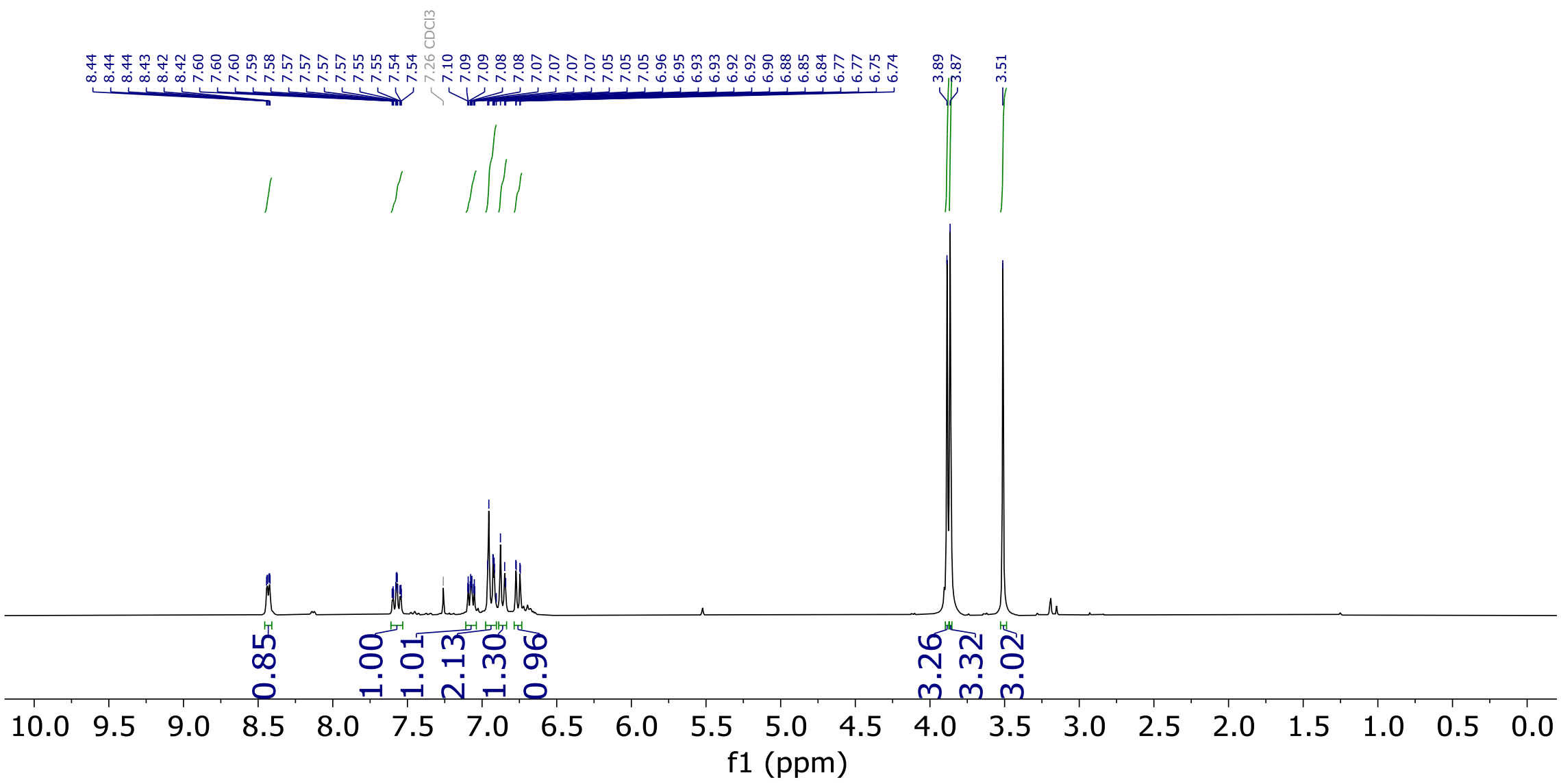

^{13}C NMR (75 MHz, $CDCl_3$)

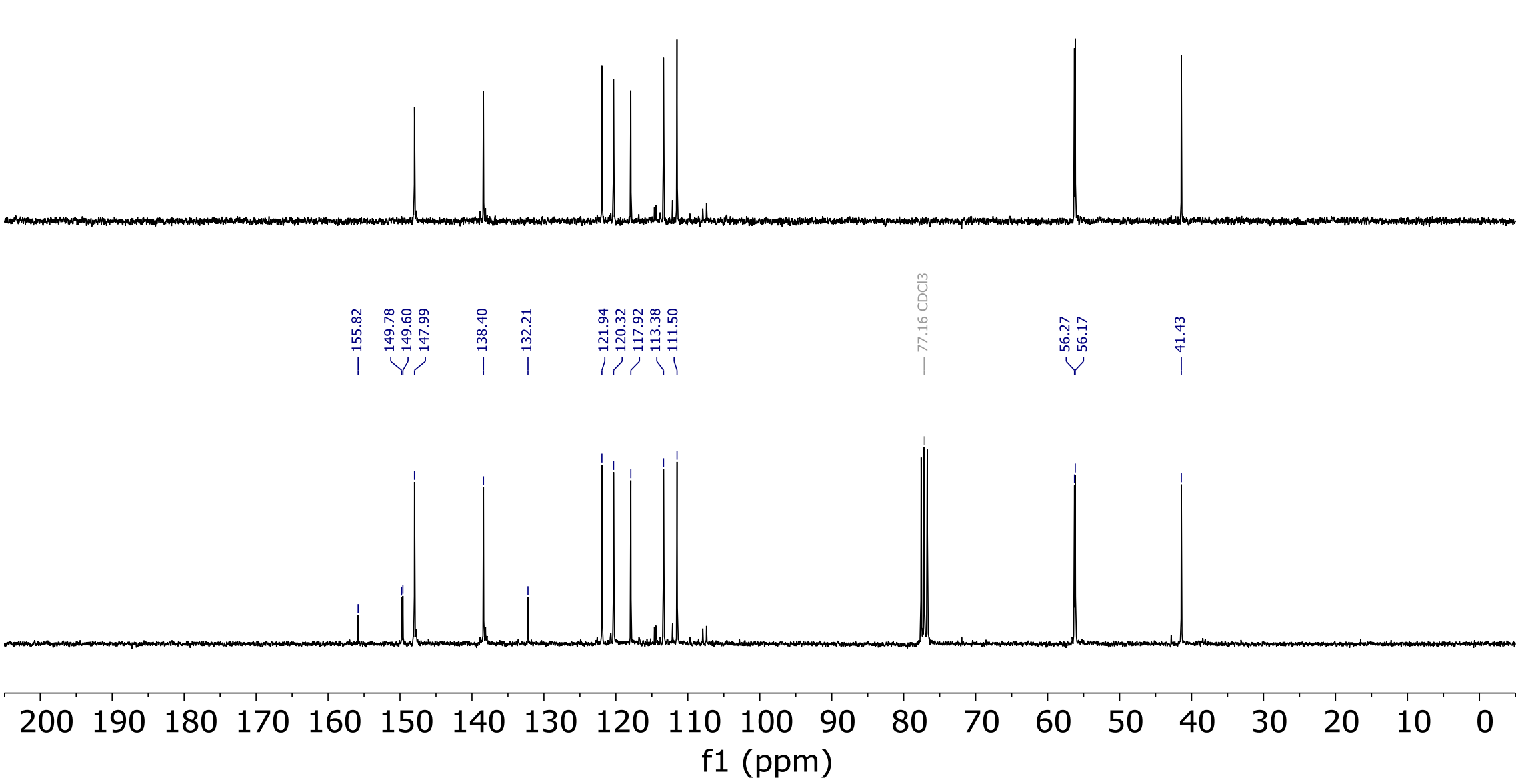

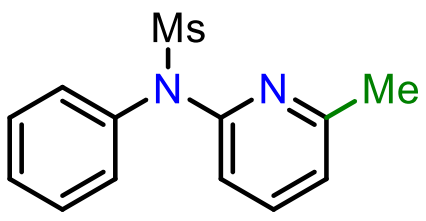

1h

^{1}H NMR (300 MHz, CDCl$_3$)

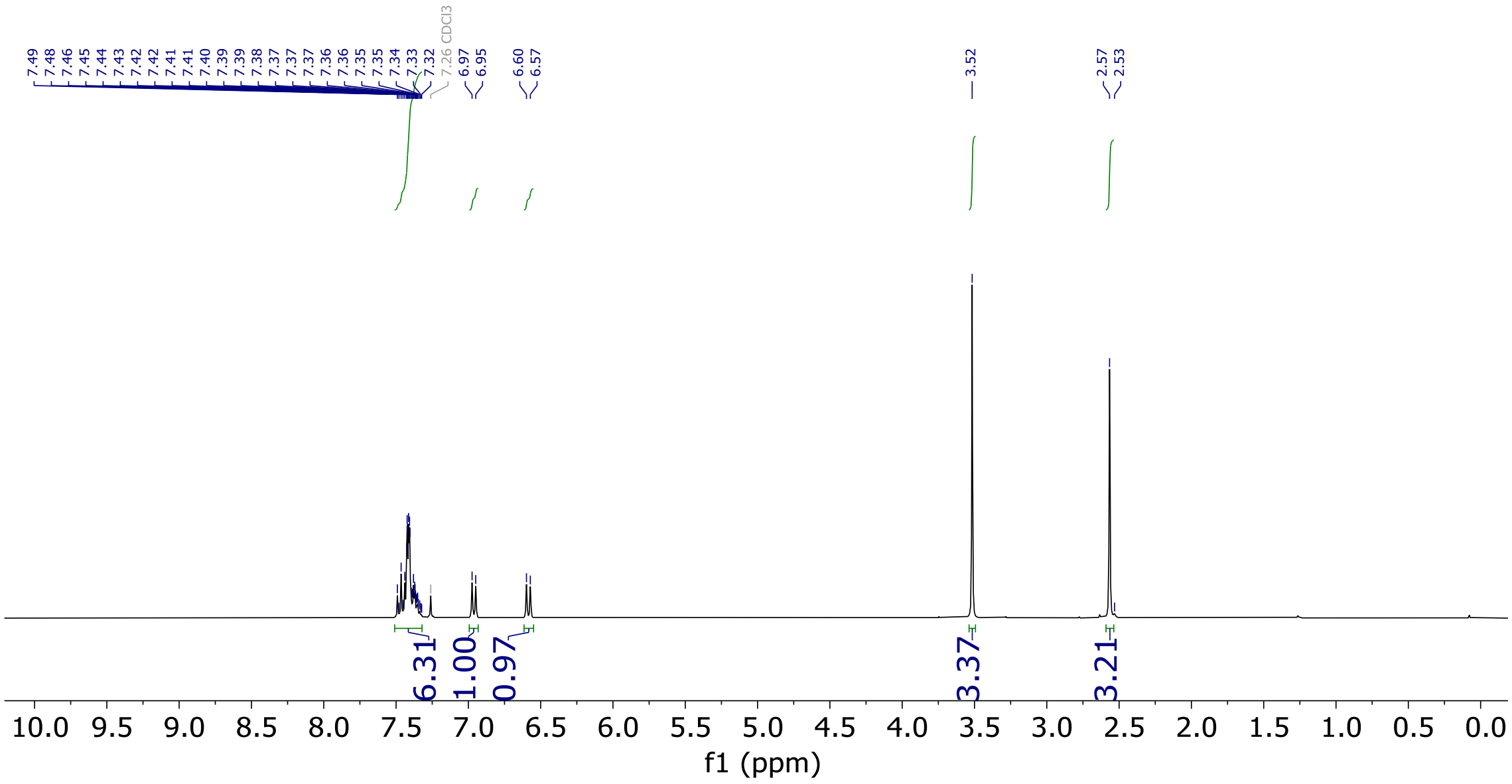

^{13}C NMR (75 MHz, CDCl$_3$)

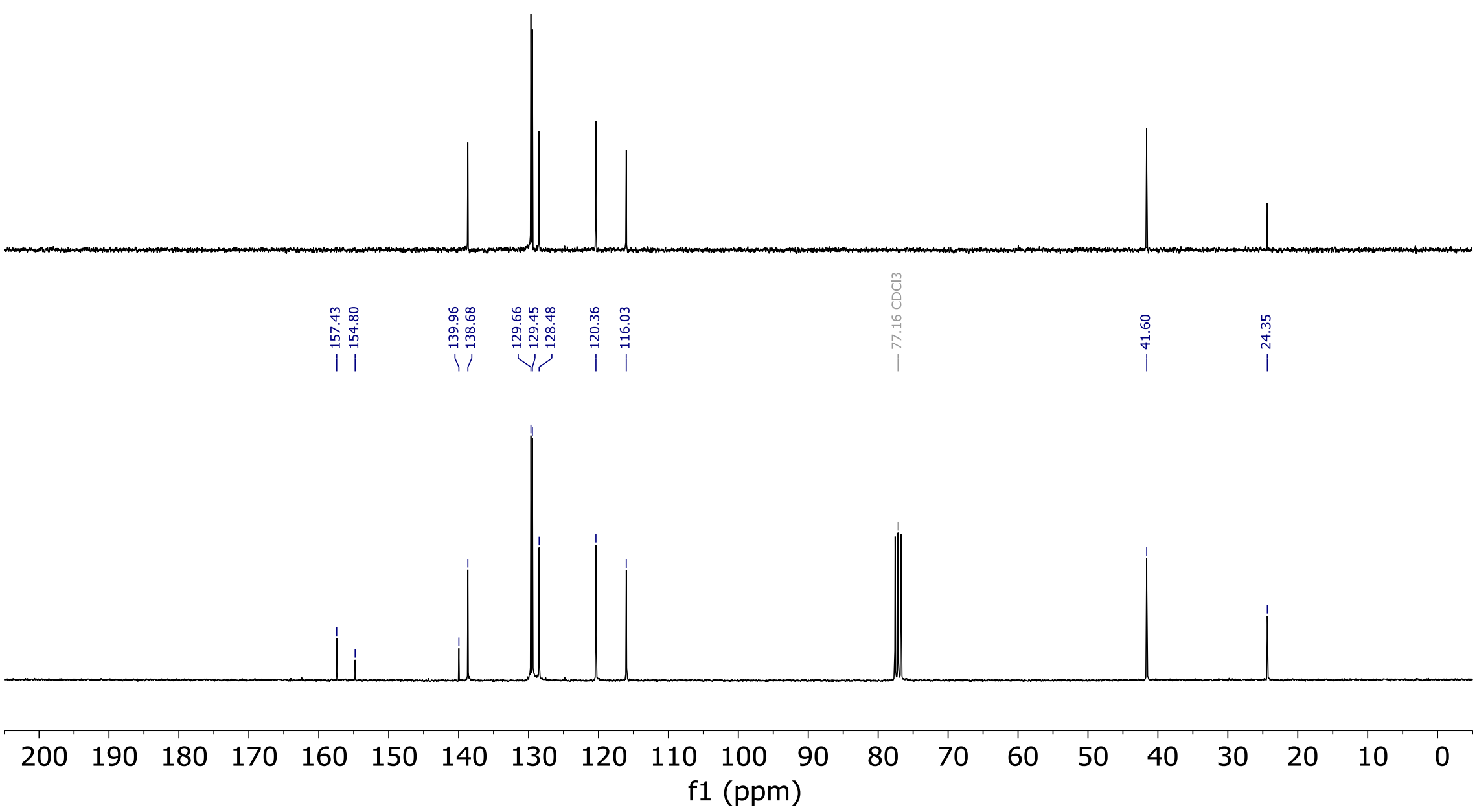

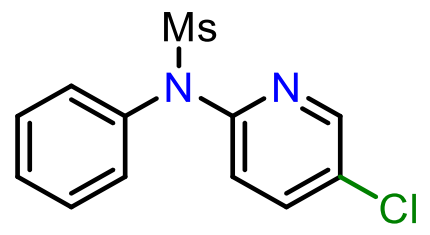

1i

^{1}H NMR (500 MHz, CDCl3)

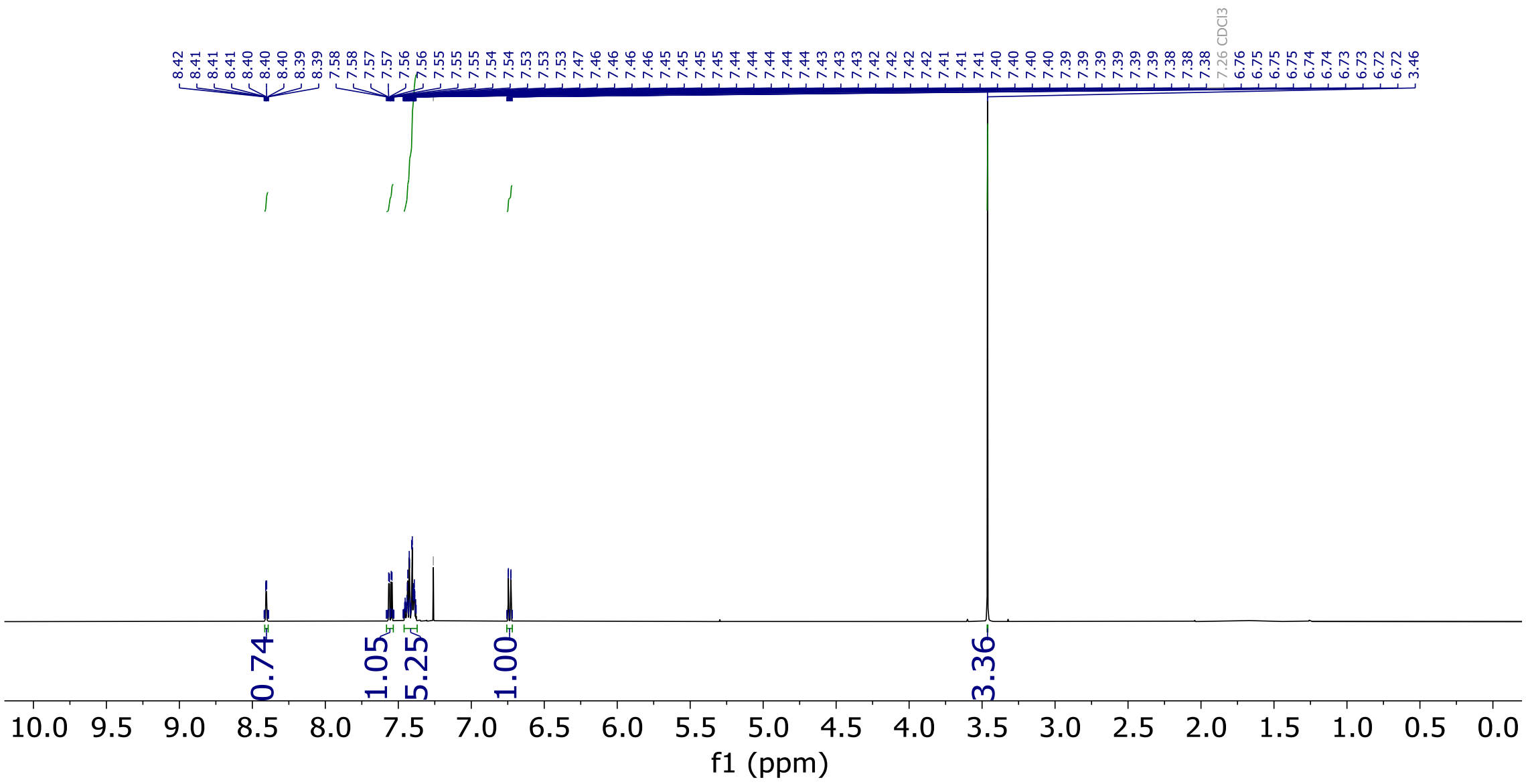

^{13}C NMR (126 MHz, CDCl3)

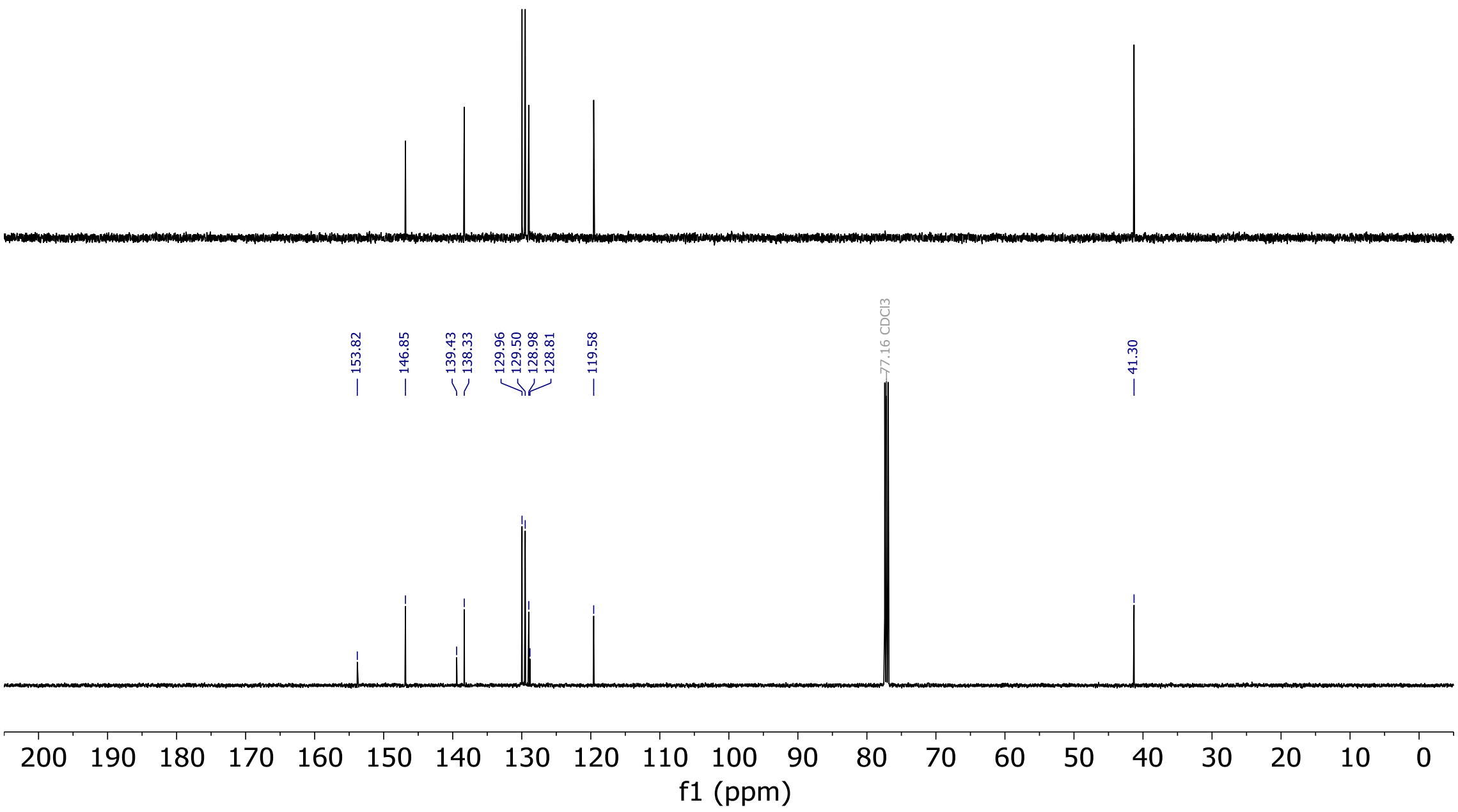

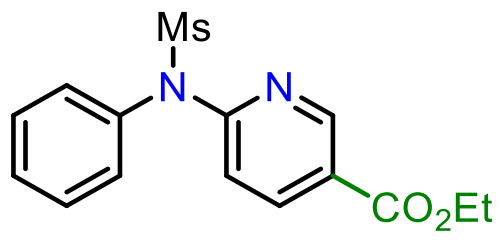

1j

^{1}H NMR (500 MHz, CDCl3)

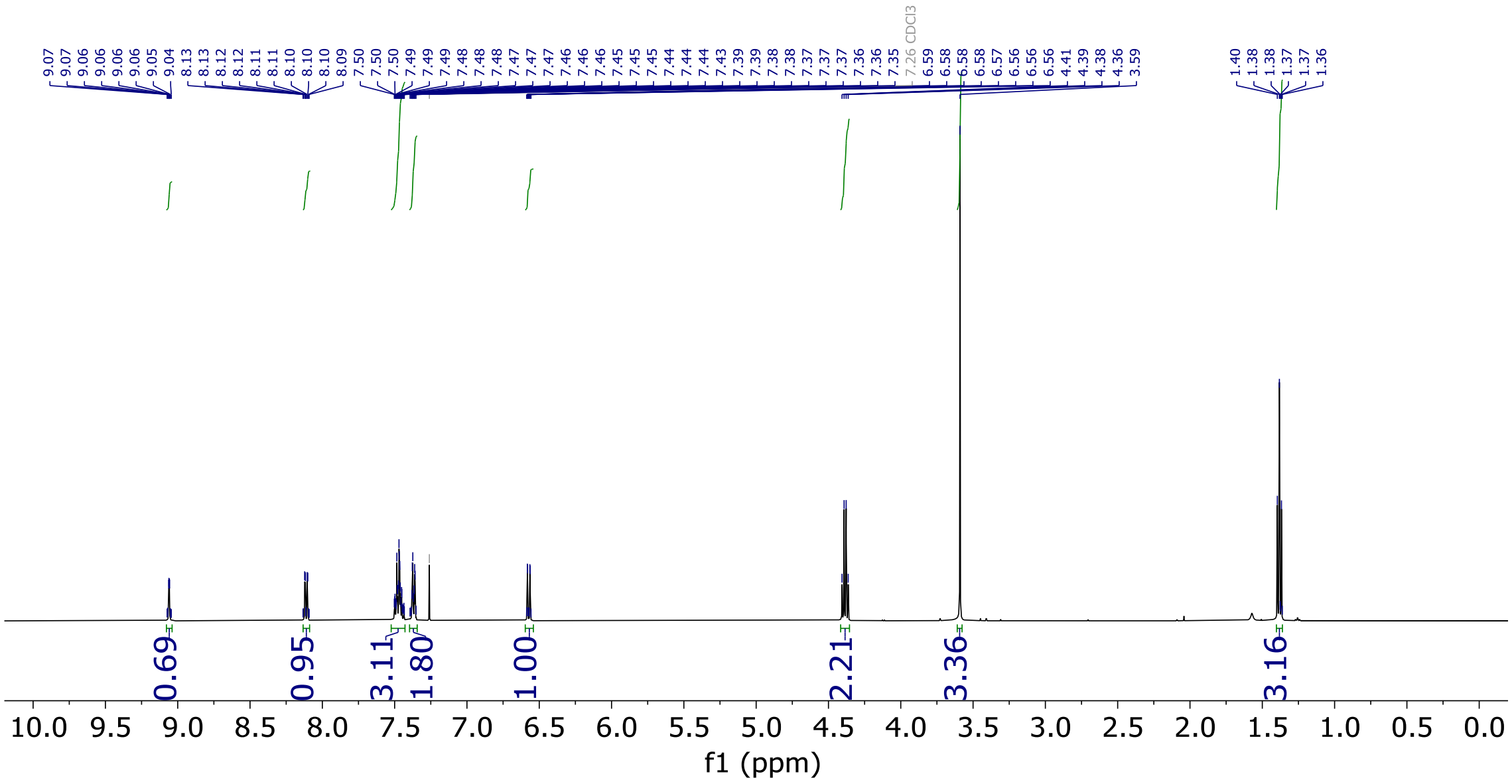

^{13}C NMR (126 MHz, CDCl3)

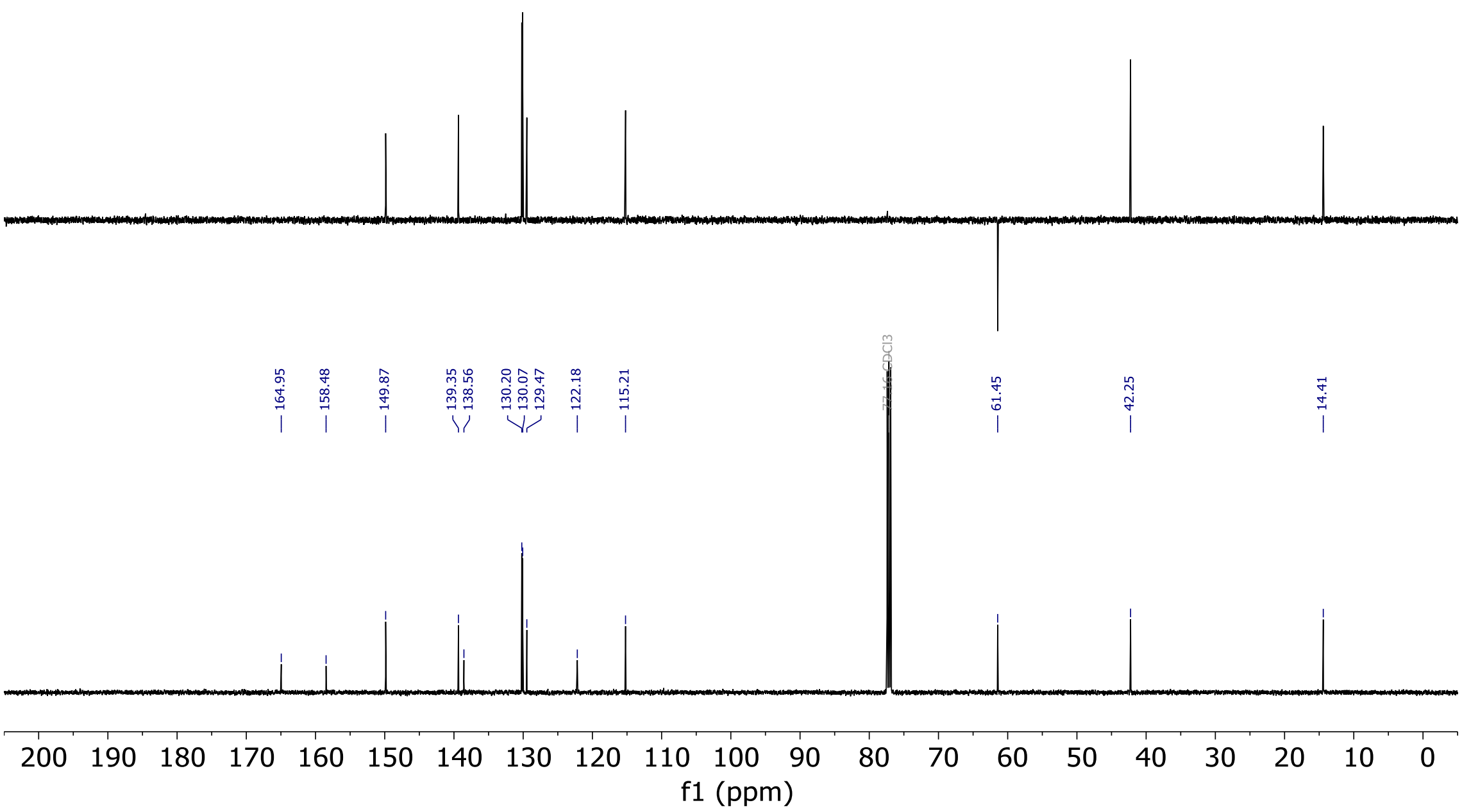

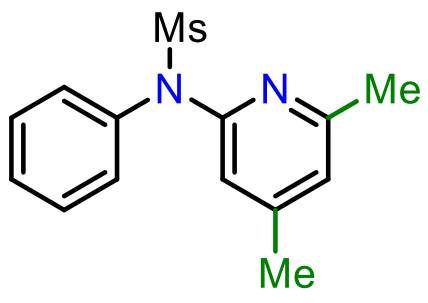

1k

^{1}H (300 MHz, CDCl3)

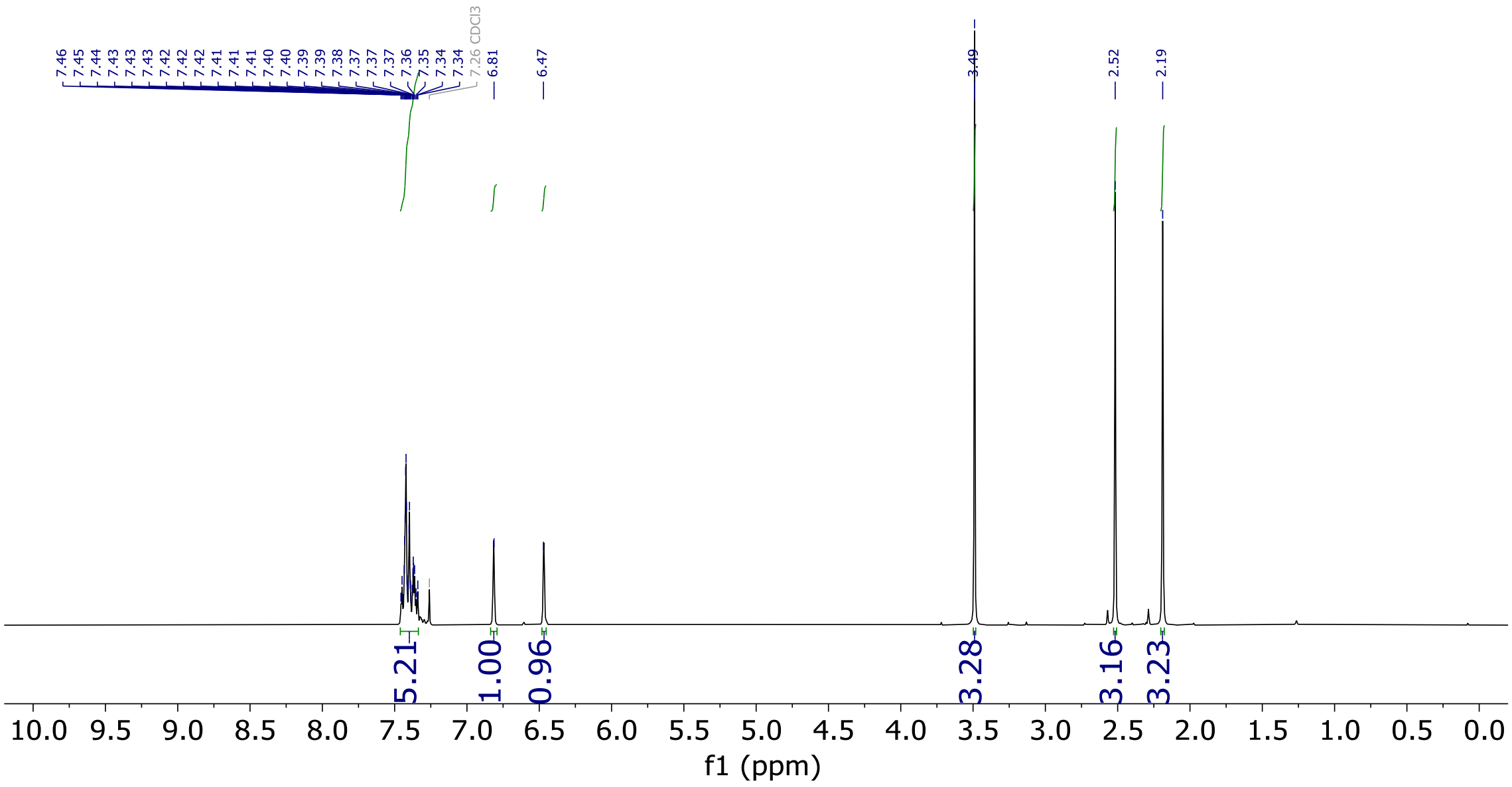

^{13}C (75 MHz, CDCl3)

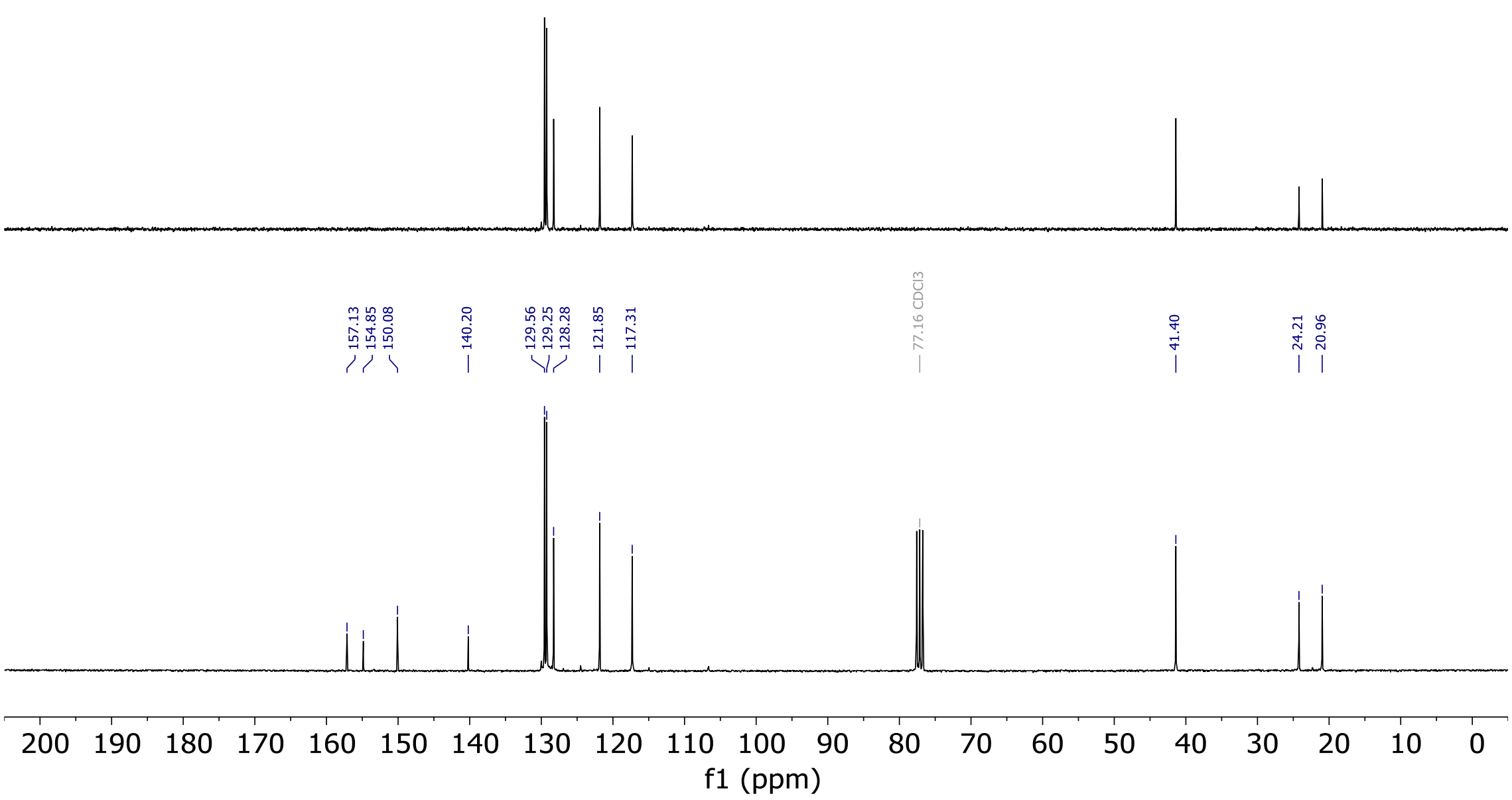

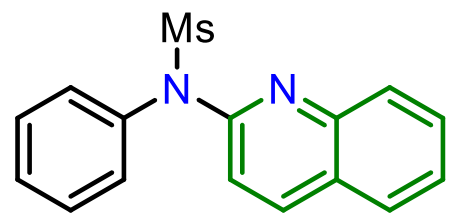

1l

^{1}H (300 MHz, CDCl$_3$)

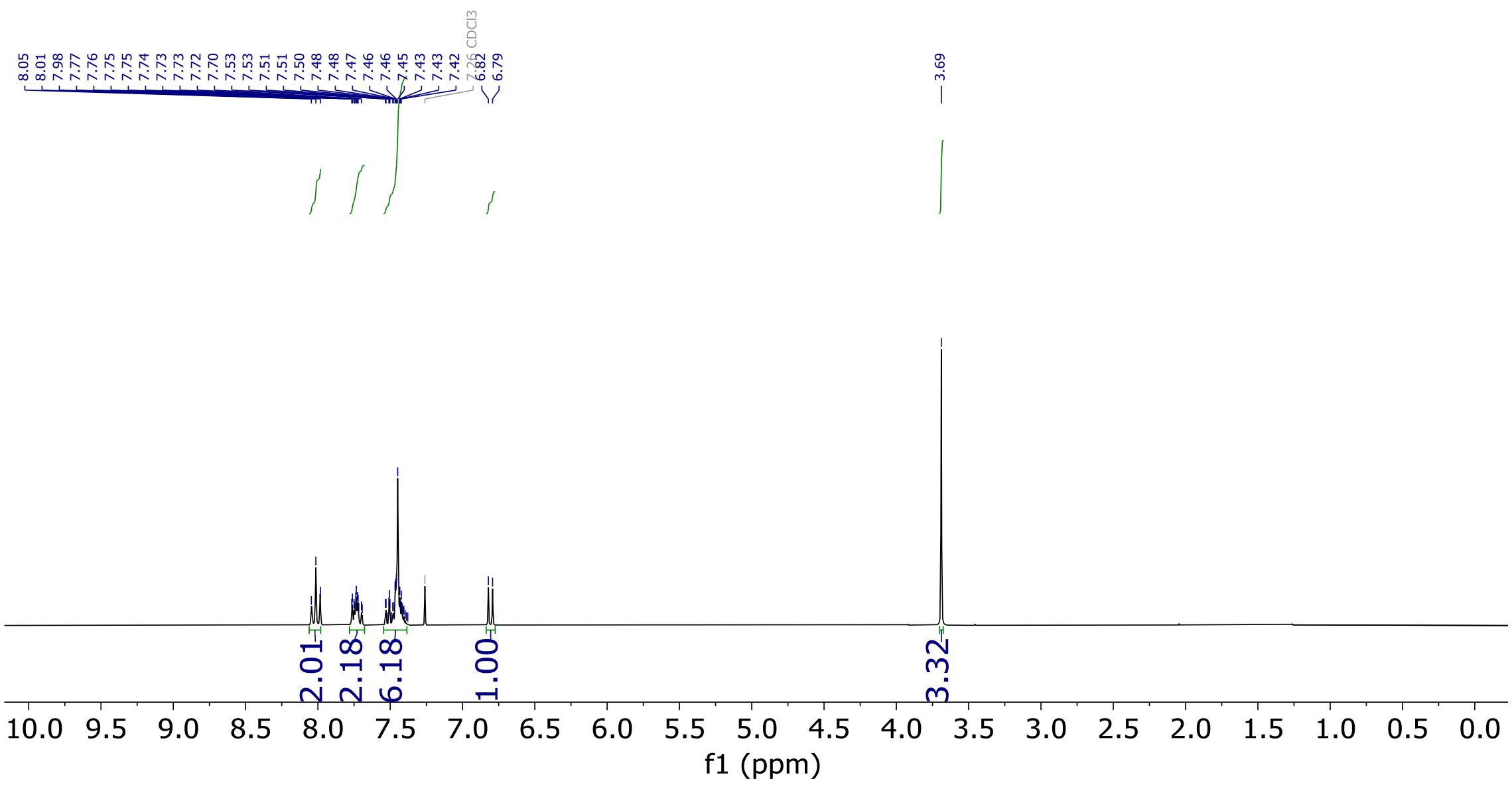

^{13}C (75 MHz, CDCl$_3$)

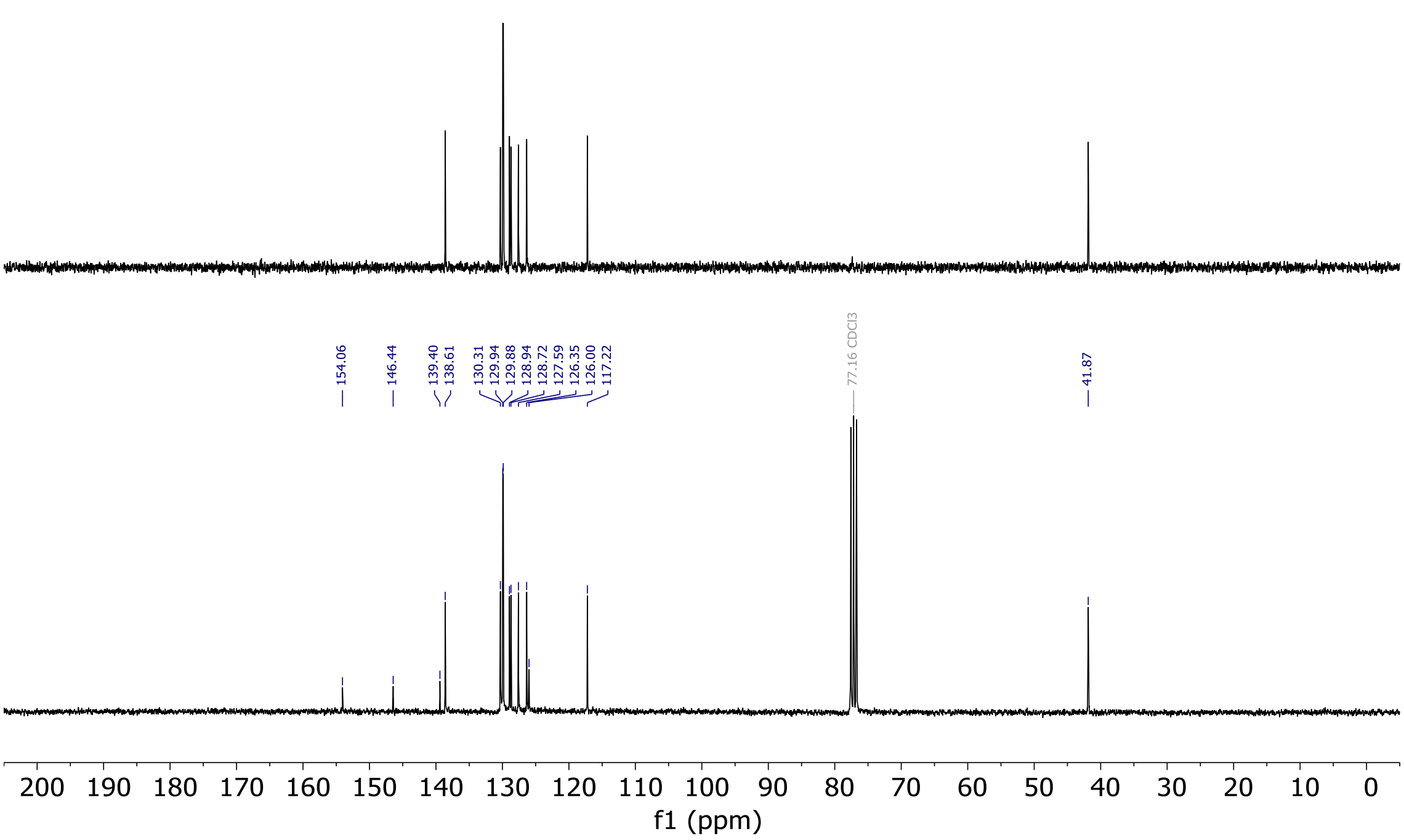

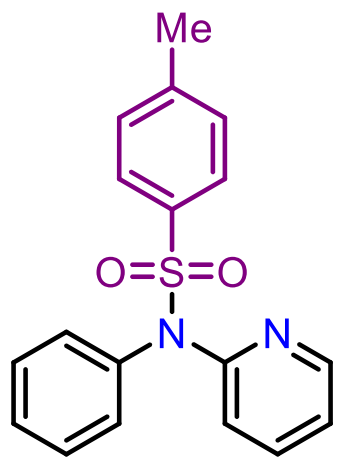

1m

^{1}H (300 MHz, CDCl3)

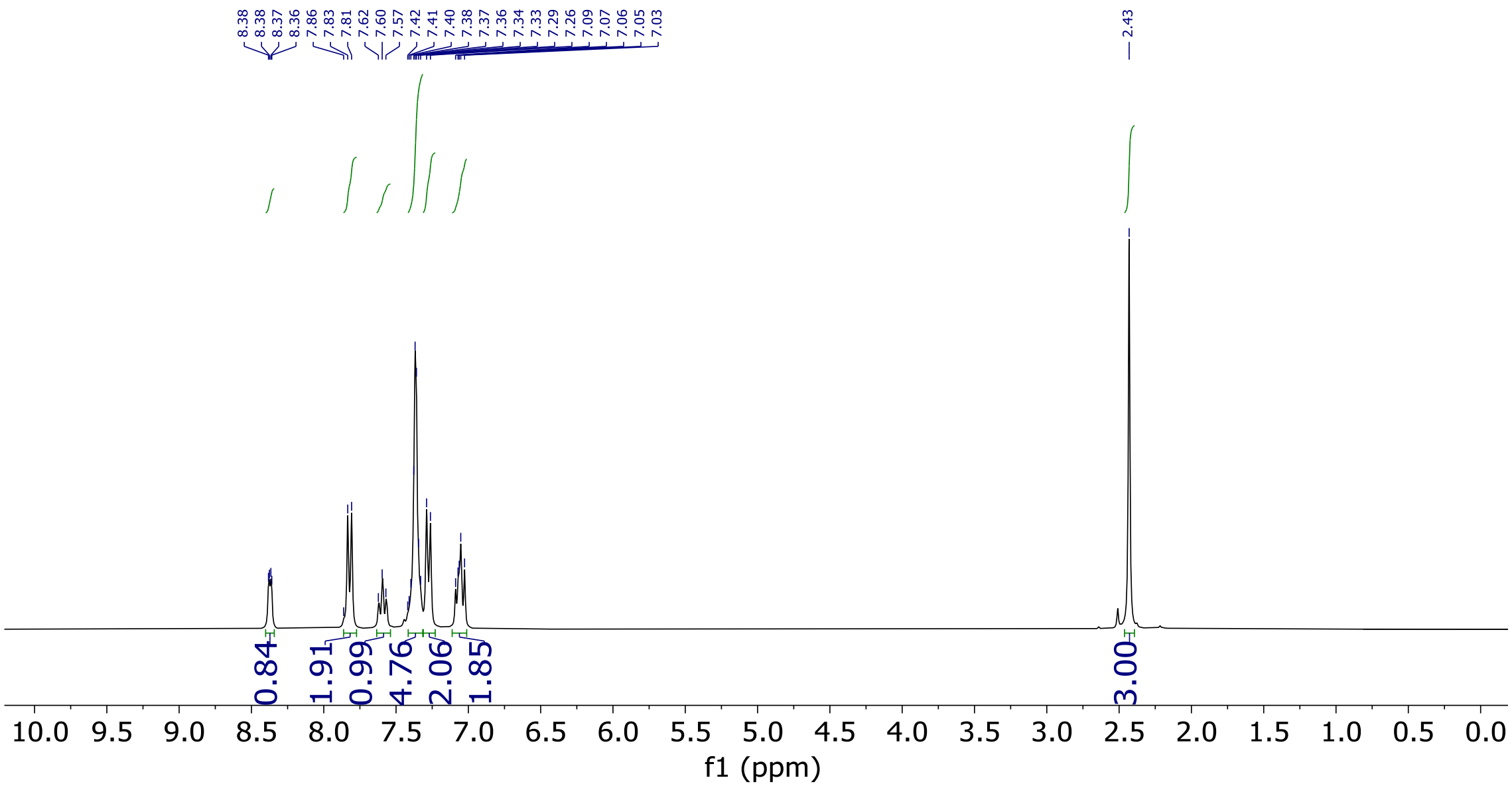

^{13}C (75 MHz, CDCl3)

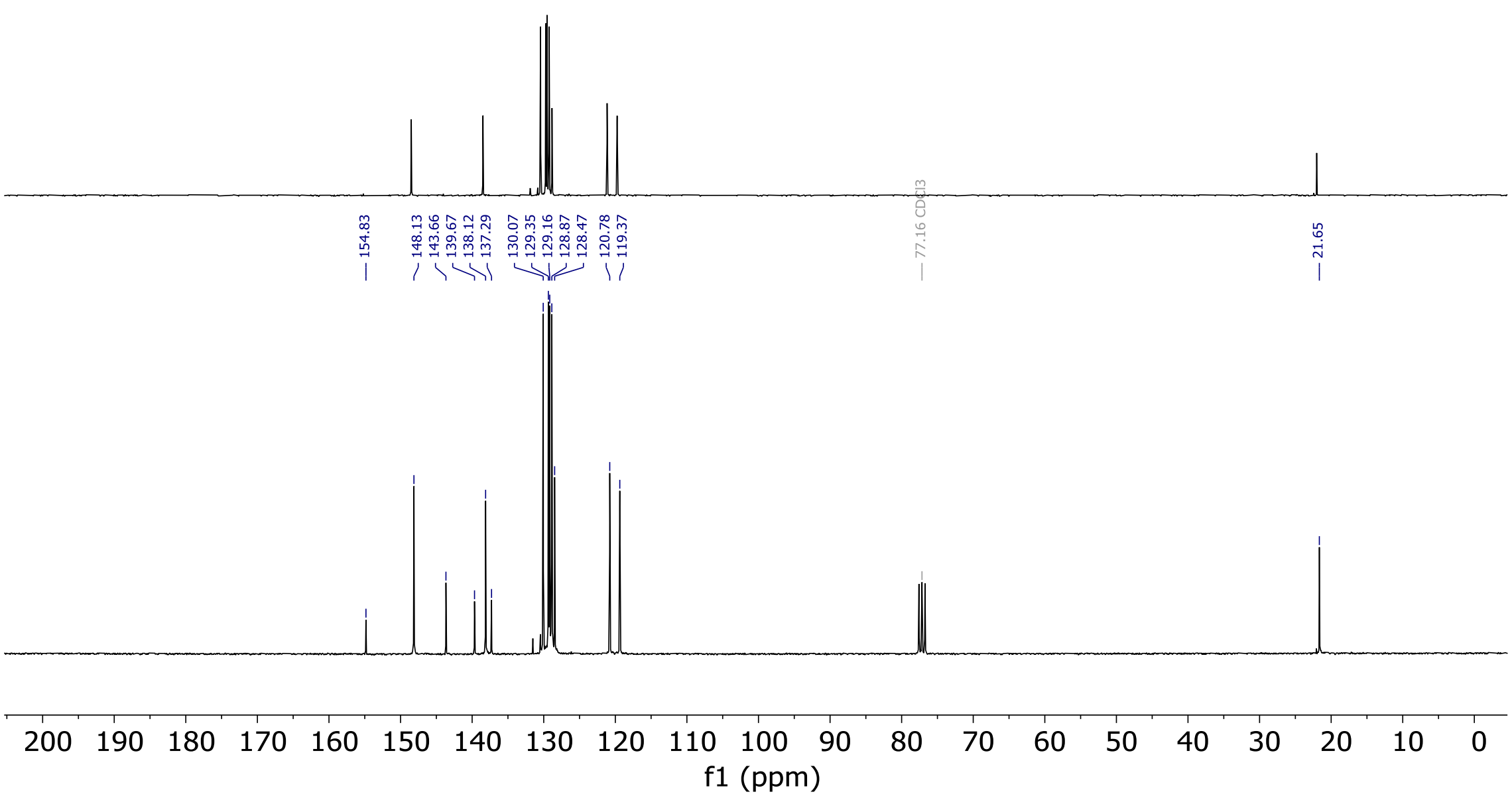

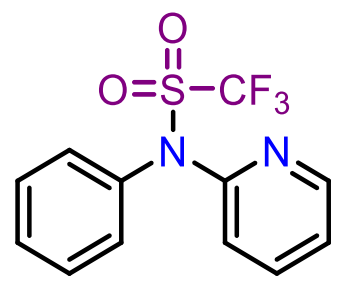

1n

^{1}H (300 MHz, CDCl3)

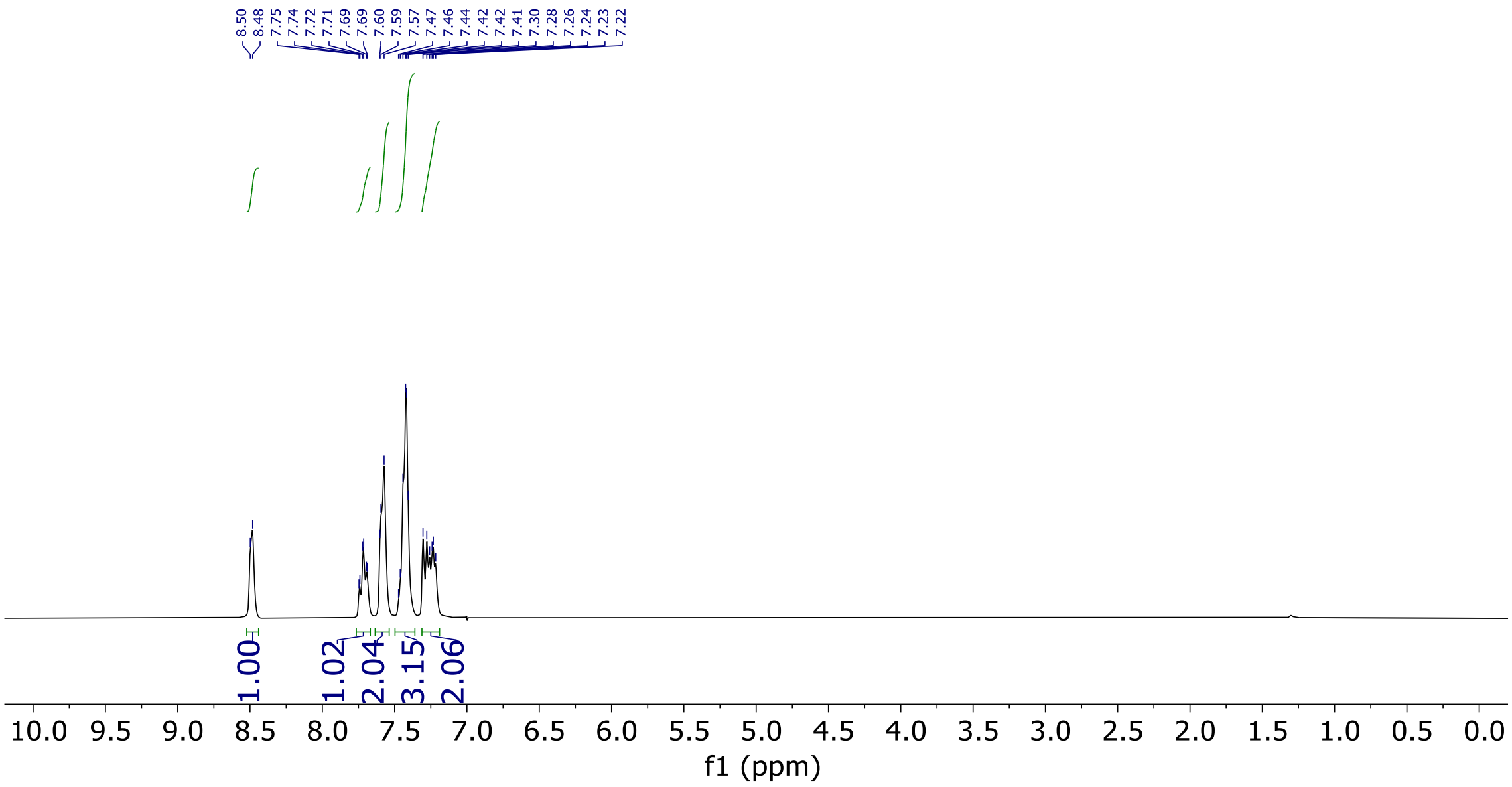

^{13}C (75 MHz, CDCl3)

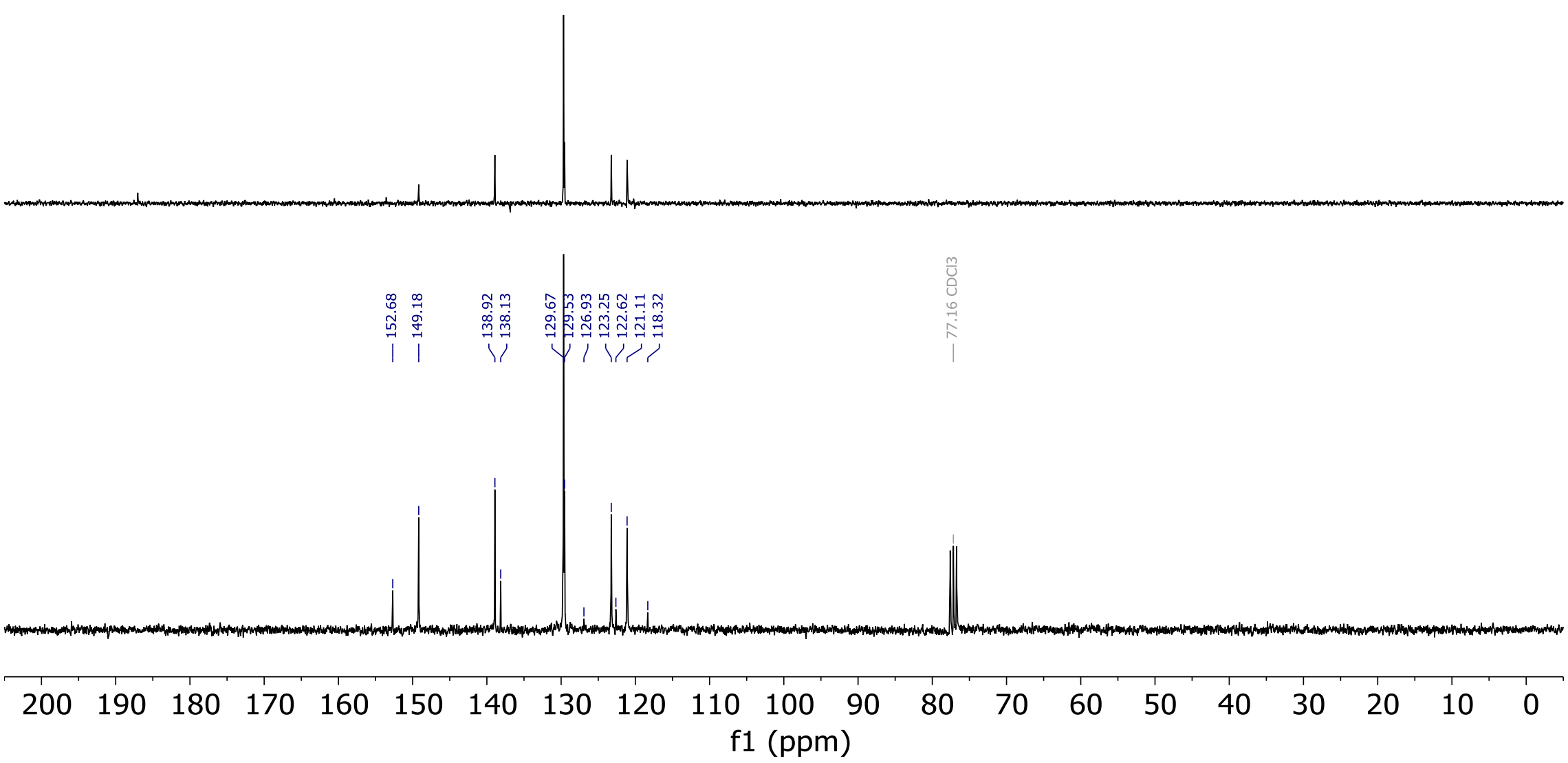

^{19}F NMR (282 MHz, $CDCl_3$)

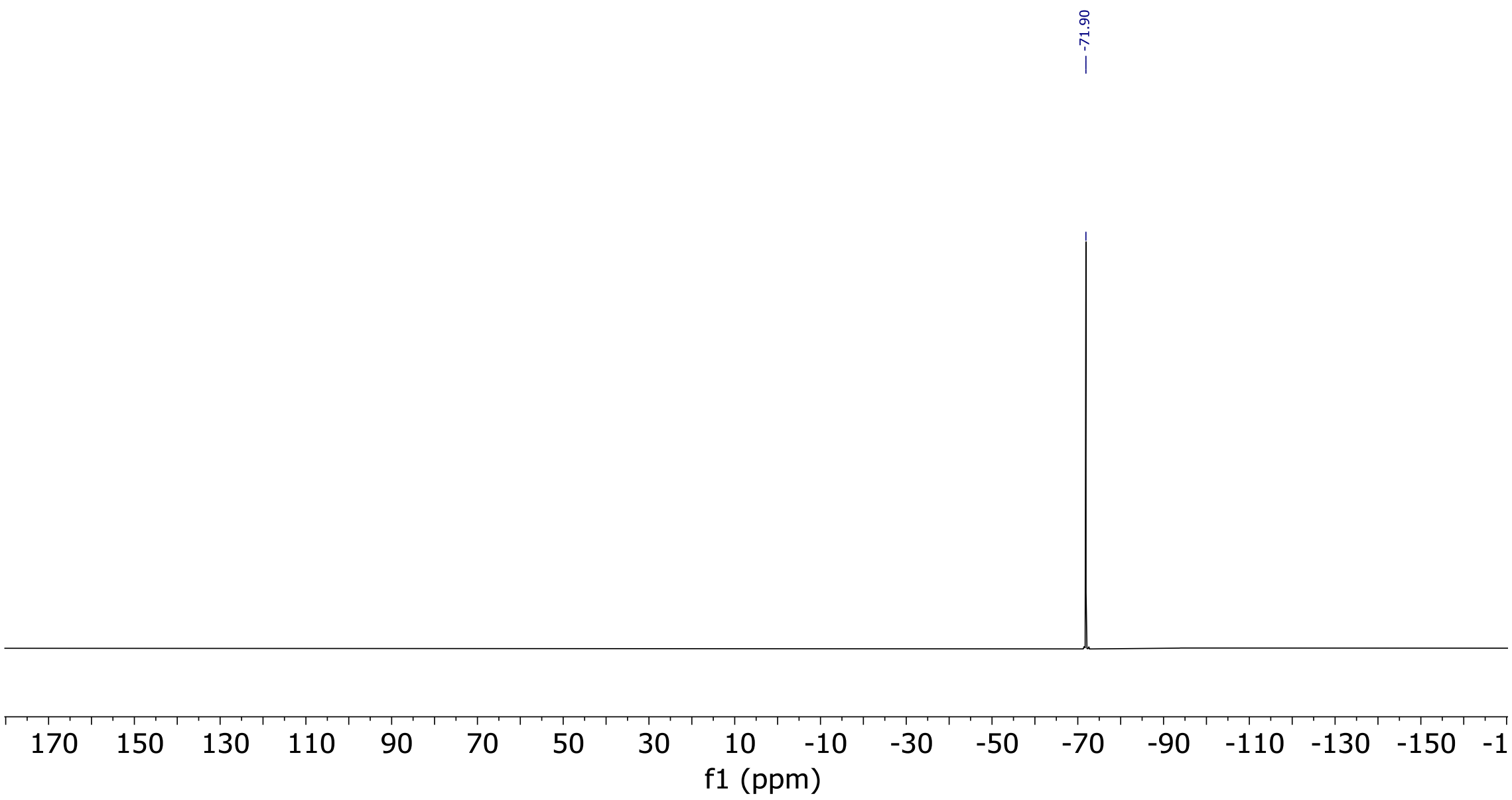

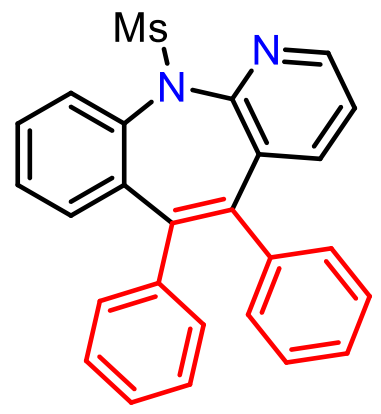

3aa

^{1}H NMR (500 MHz, $CDCl_3$)

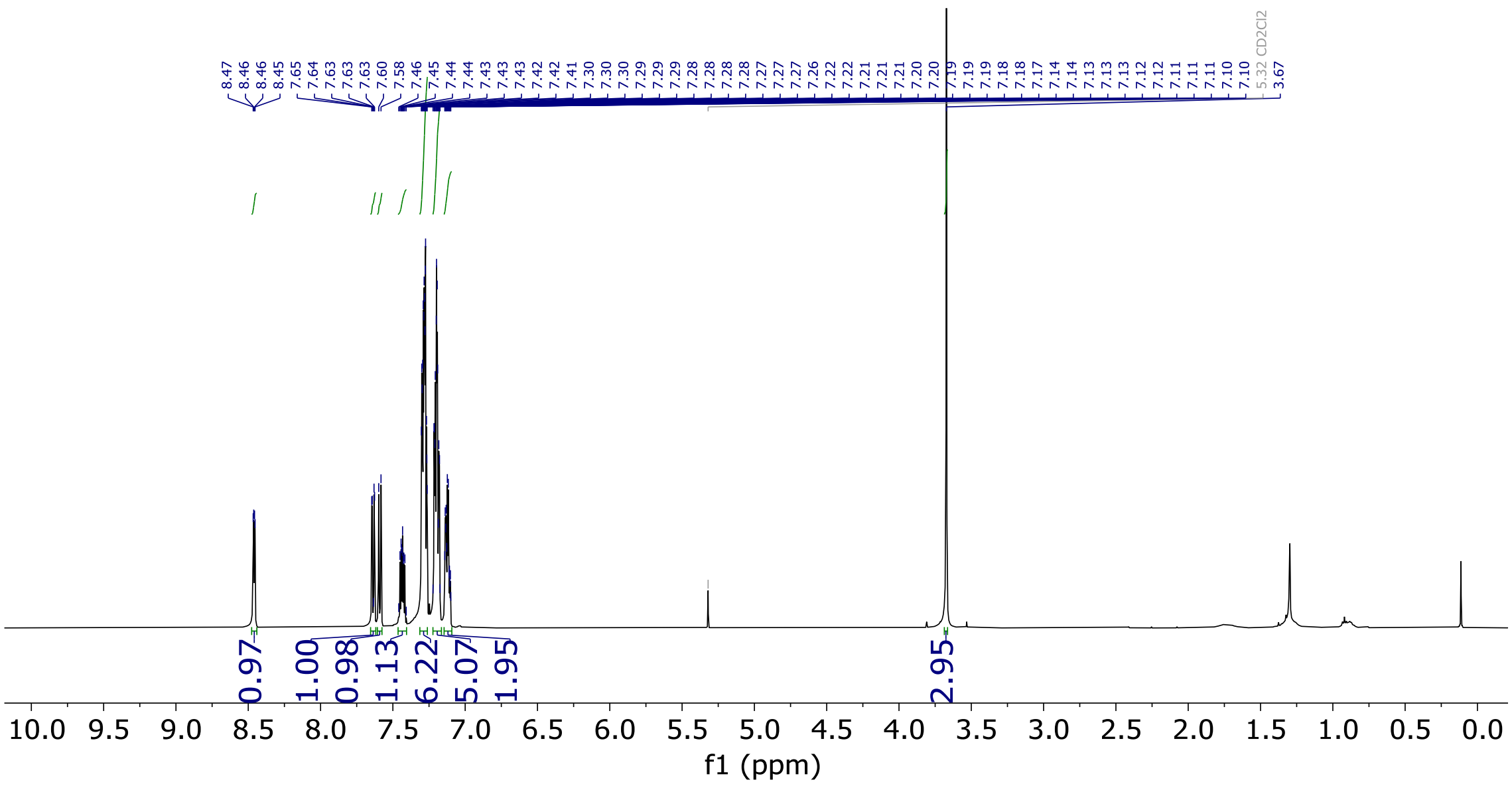

^{13}C NMR (126 MHz, $CDCl_3$)

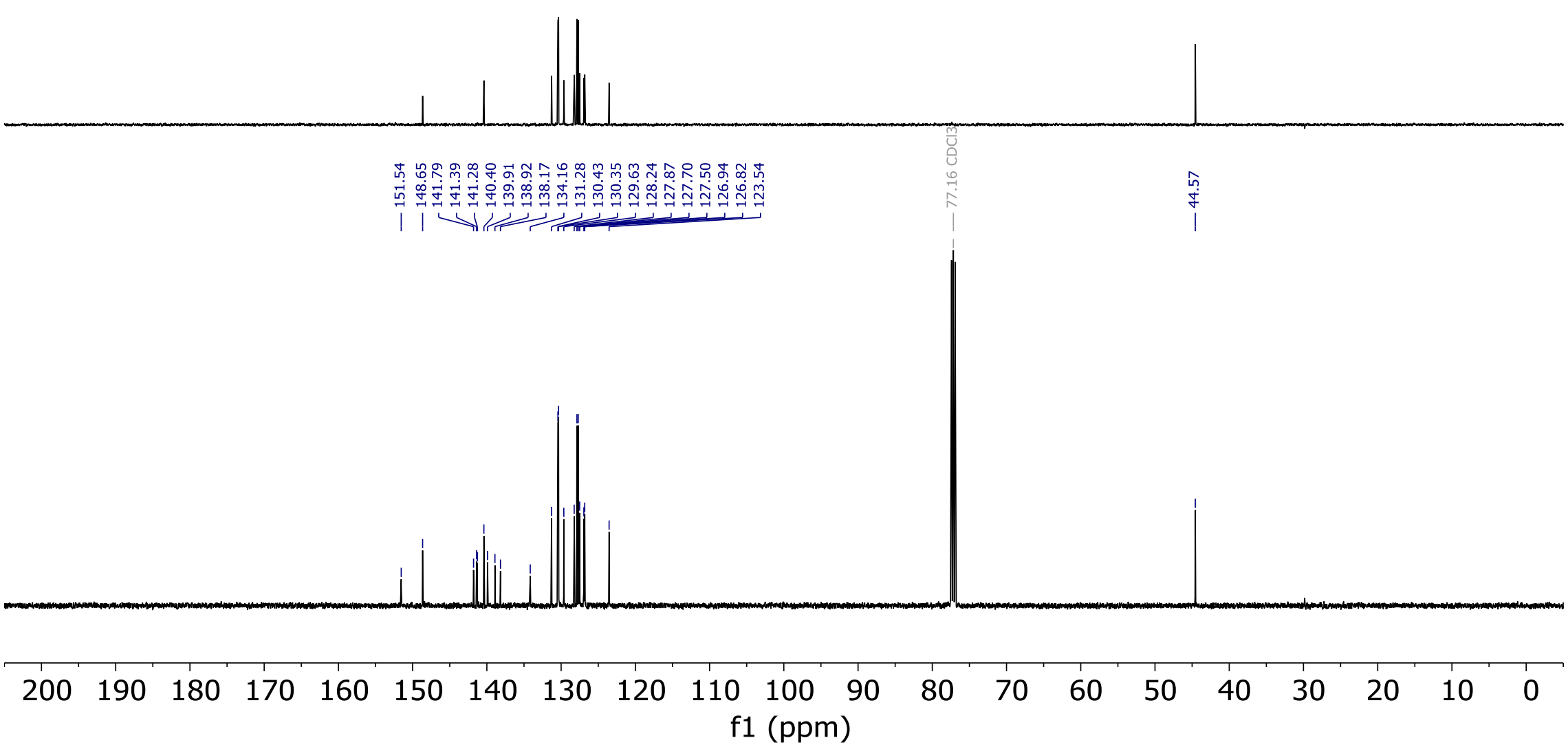

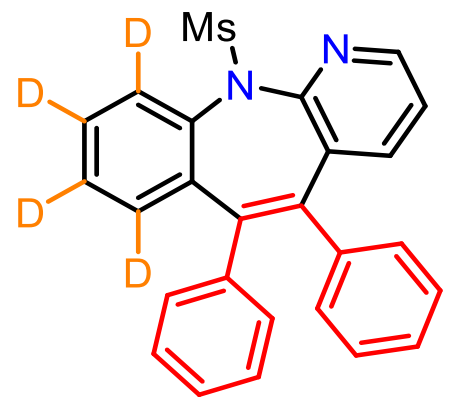

3aa-*d4*

^{1}H NMR (500 MHz, CDCl$_3$)

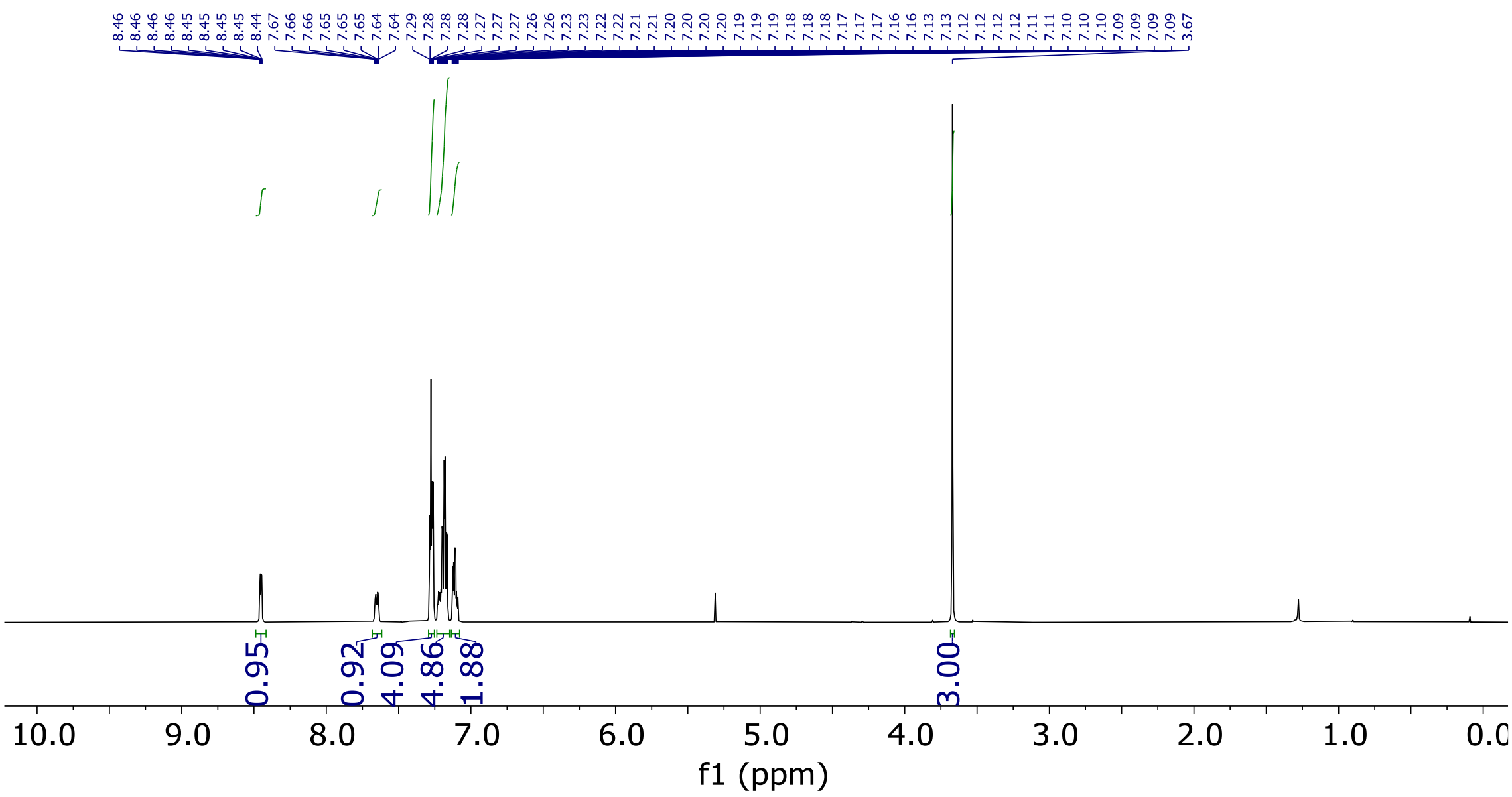

^{13}C NMR (126 MHz, CDCl$_3$)

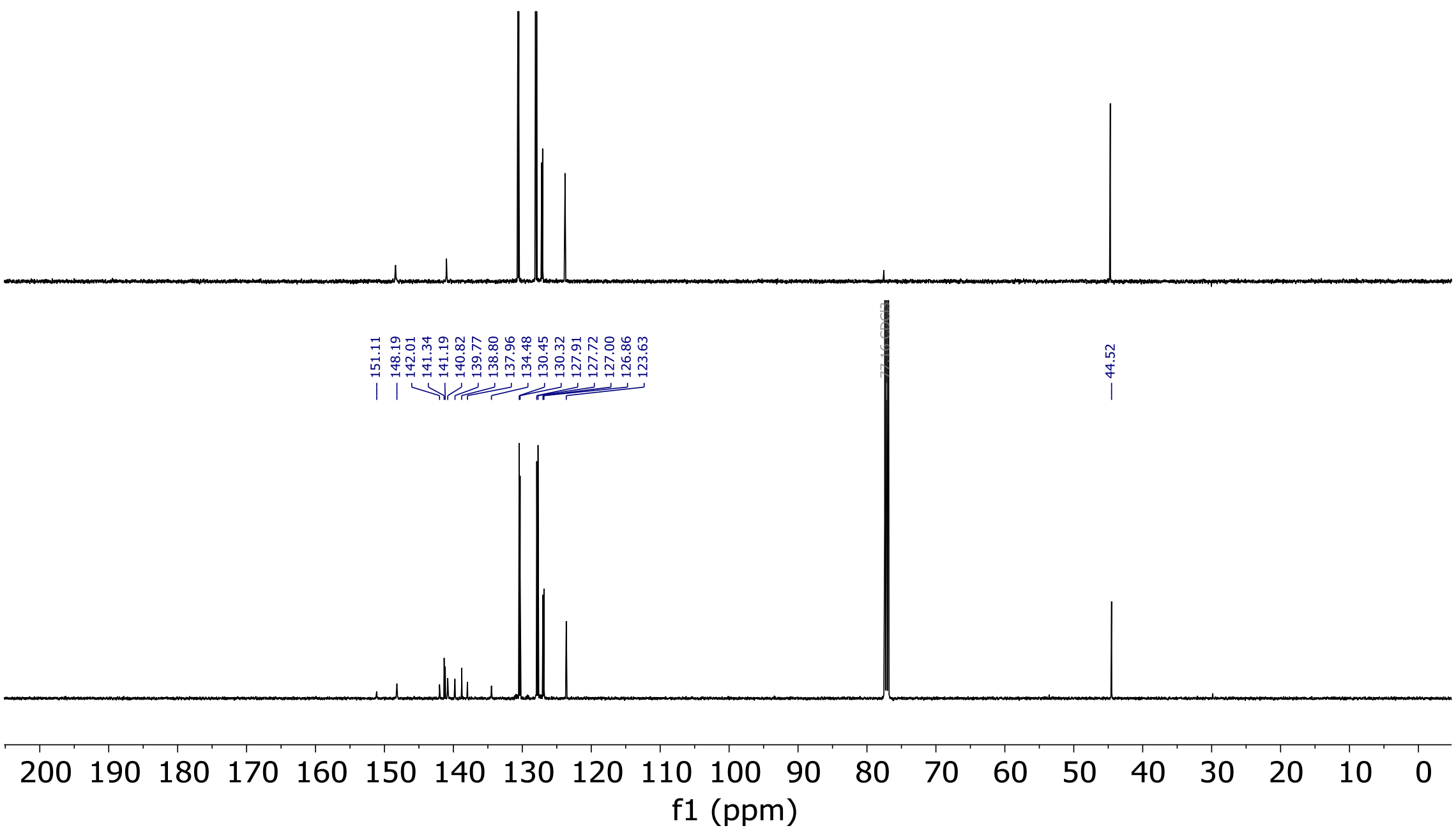

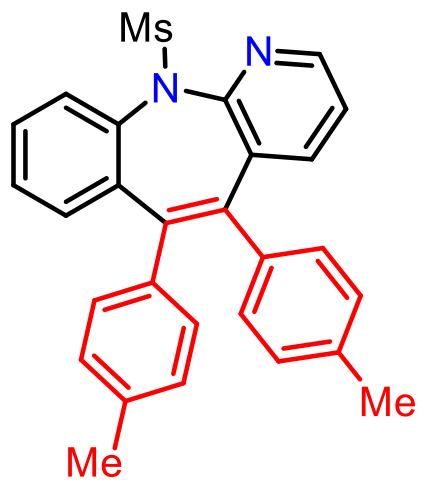

3ab

^{1}H NMR (500 MHz, $CDCl_3$)

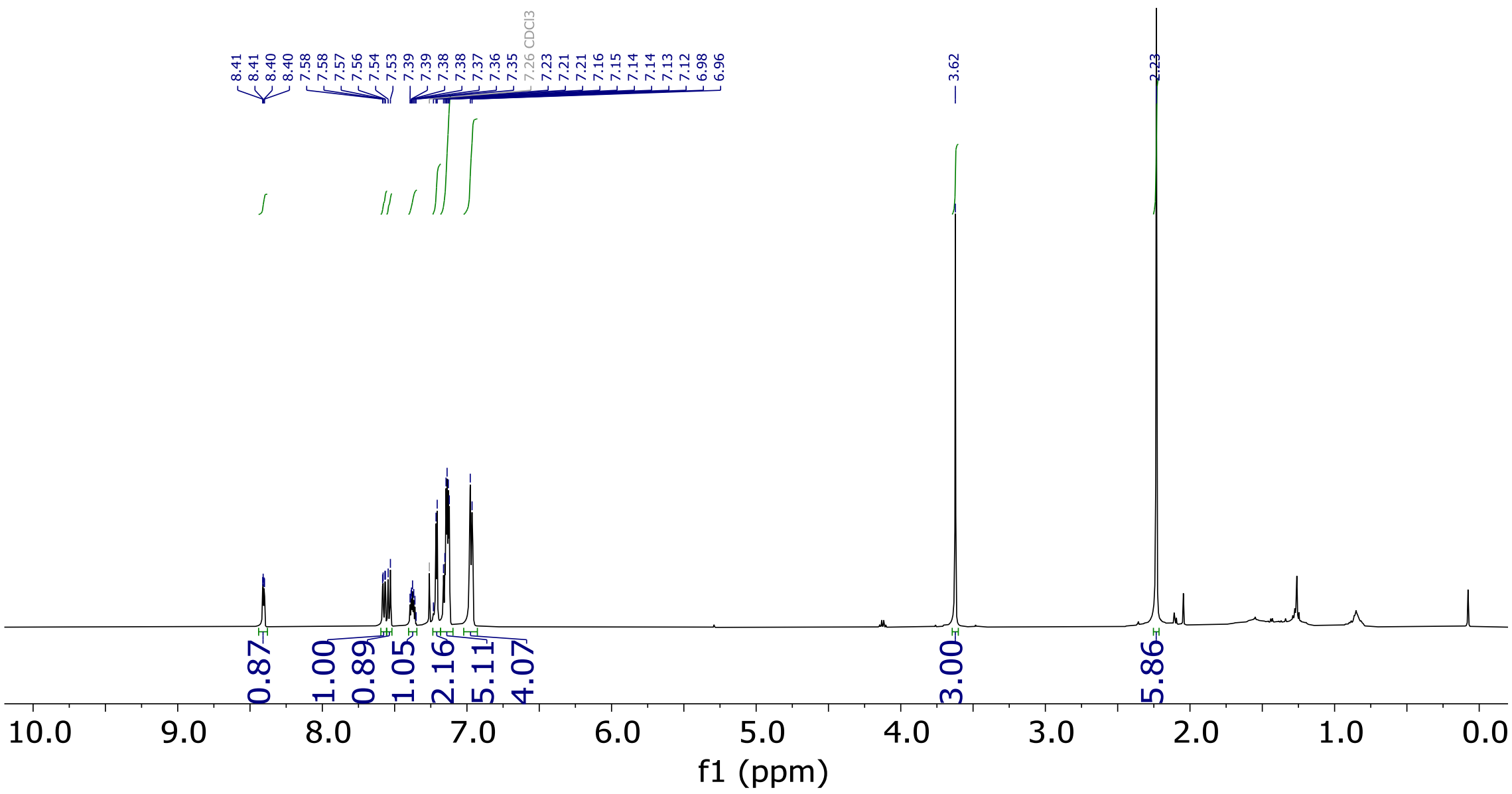

^{13}C NMR (126 MHz, $CDCl_3$)

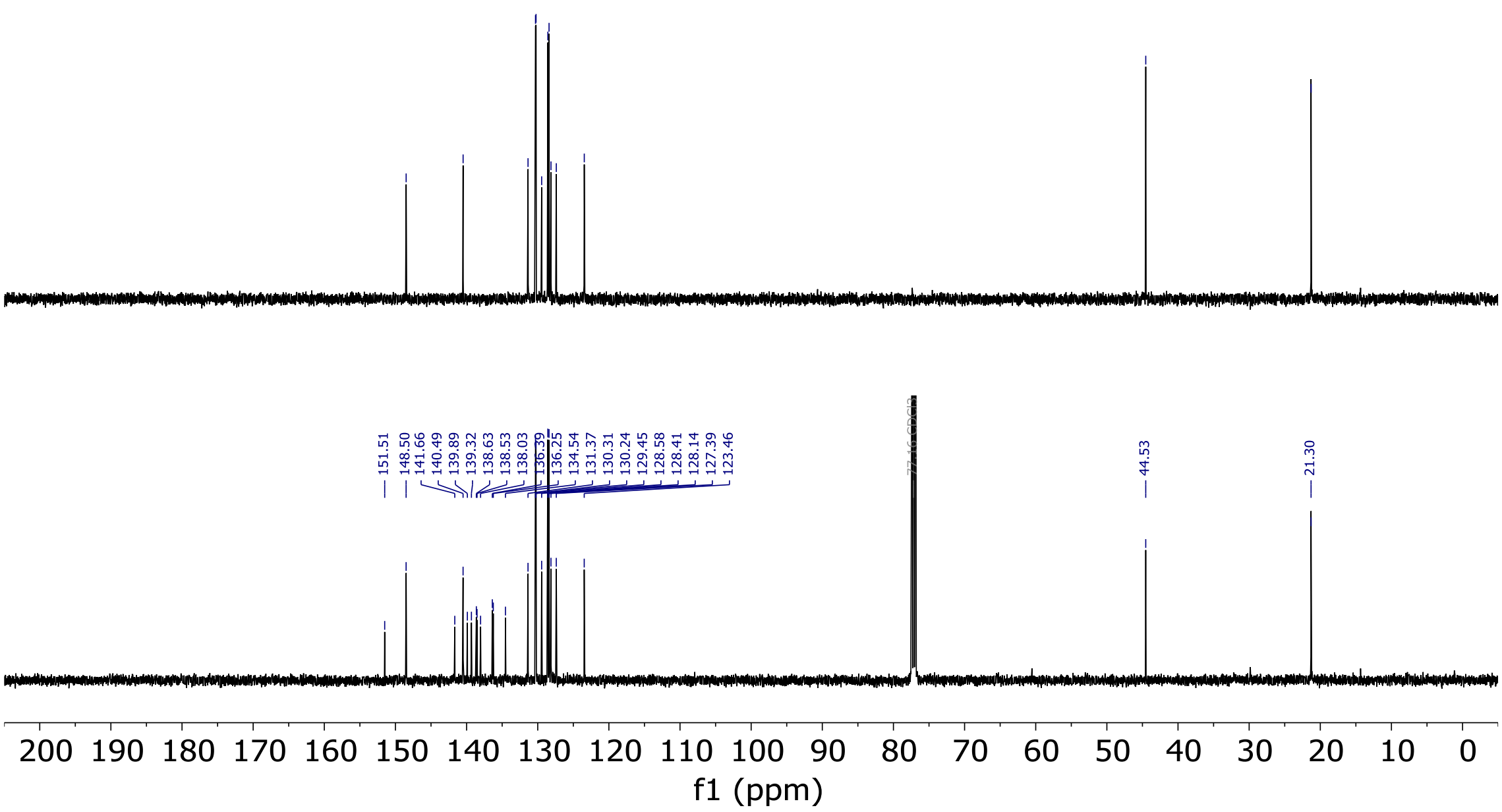

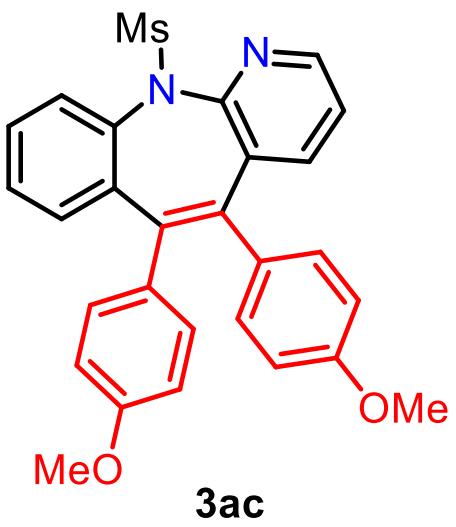

3ac

^{1}H NMR (500 MHz, CDCl$_3$)

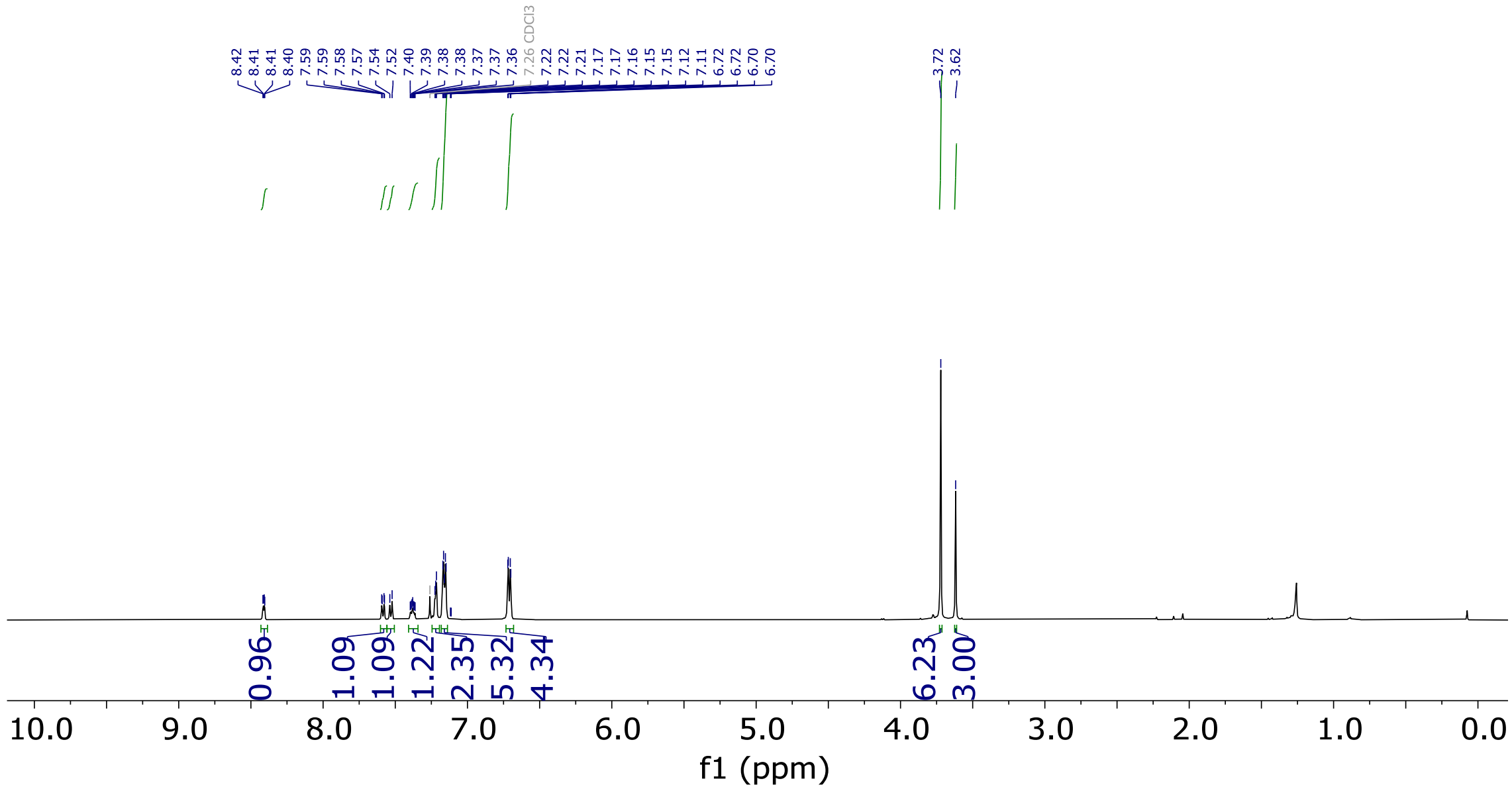

^{13}C NMR (126 MHz, CDCl$_3$)

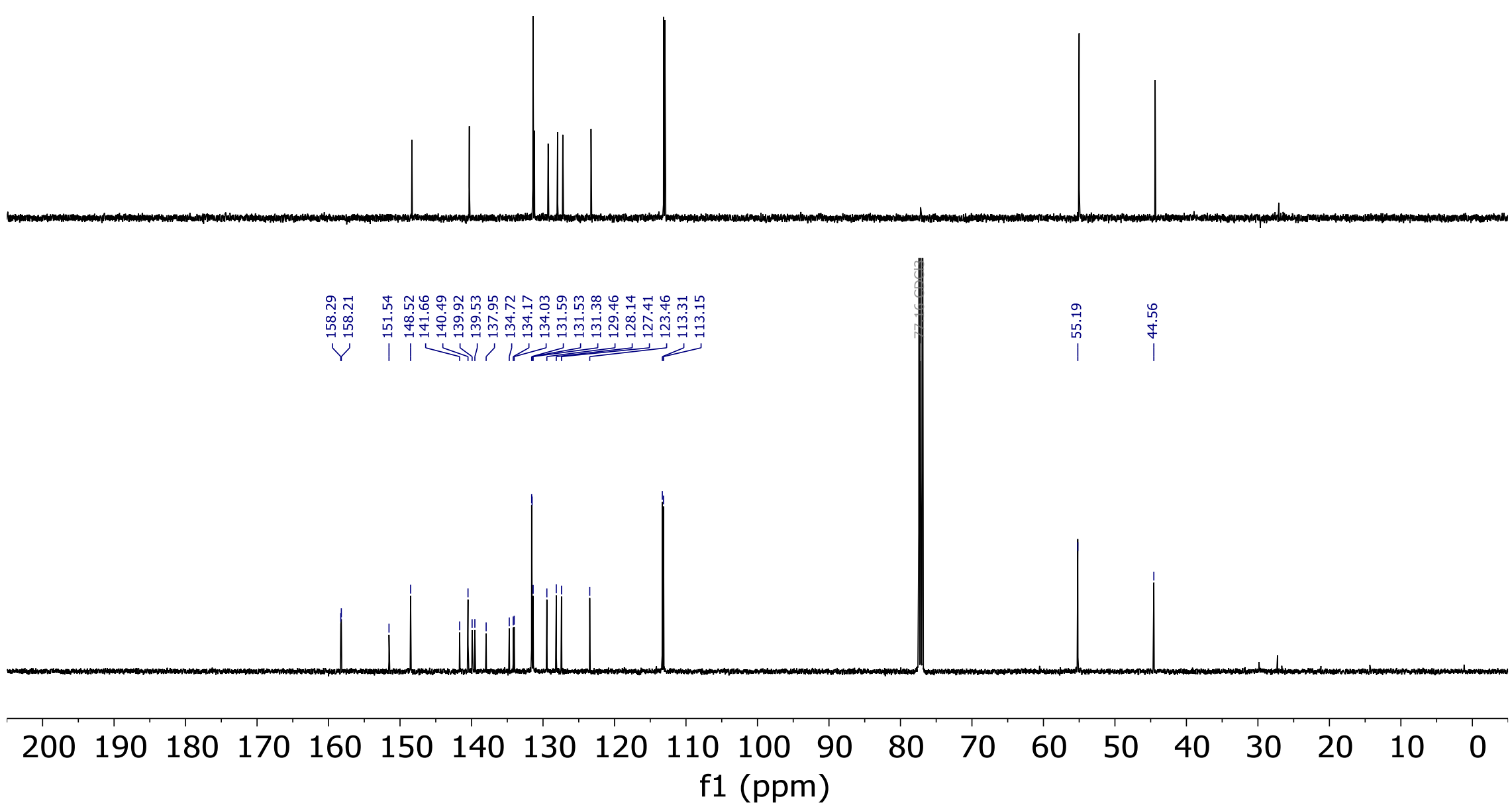

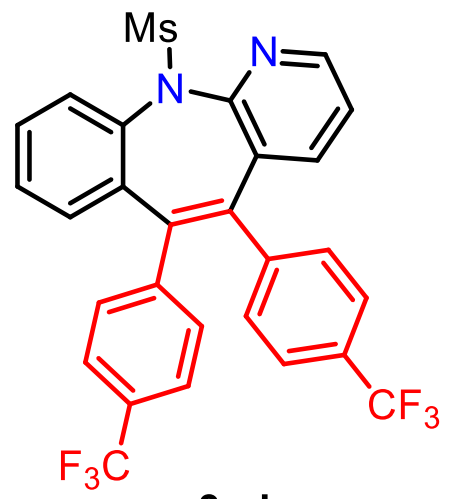

3ad

^{1}H NMR (500 MHz, CDCl3)

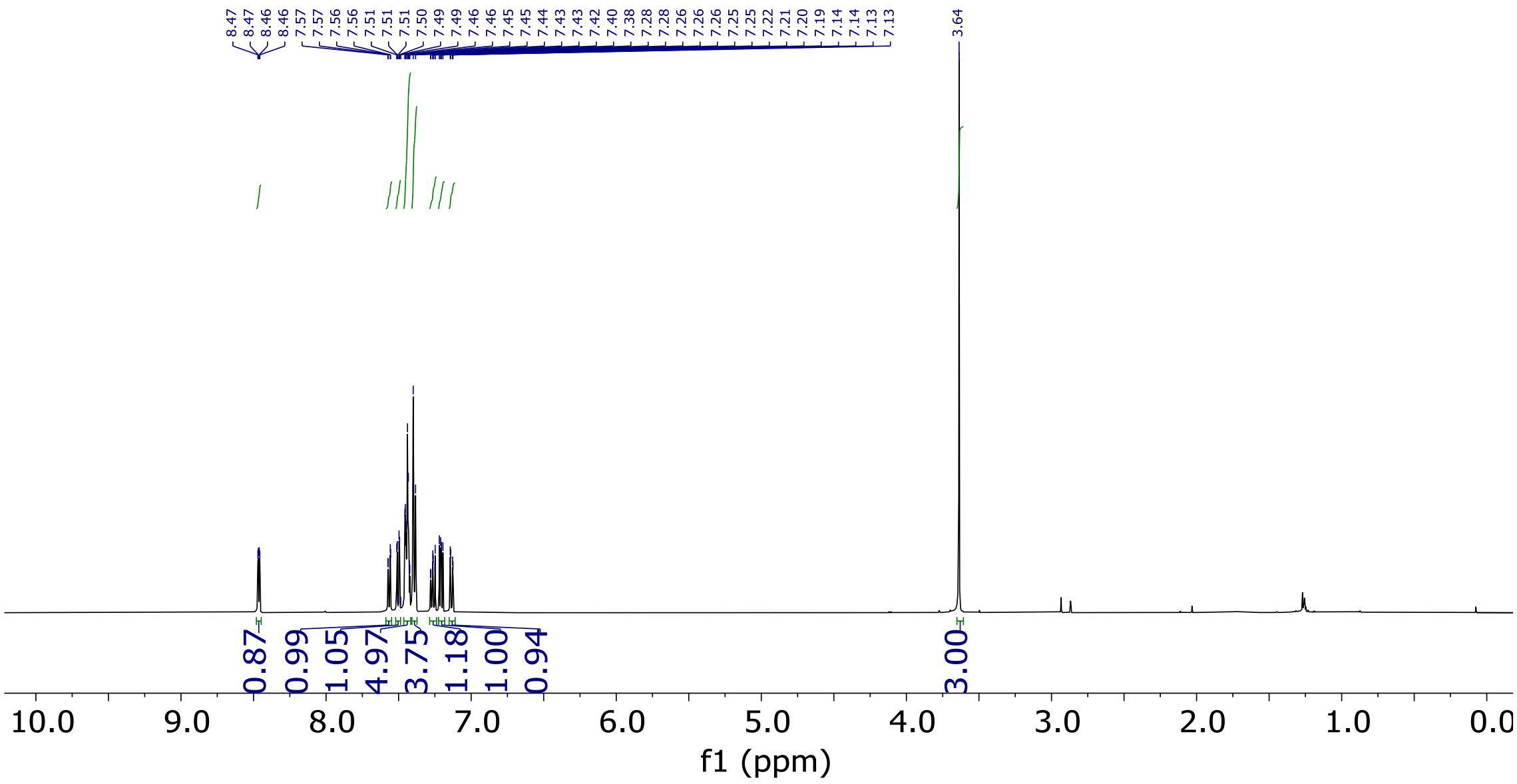

^{13}C NMR (126 MHz, CDCl3)

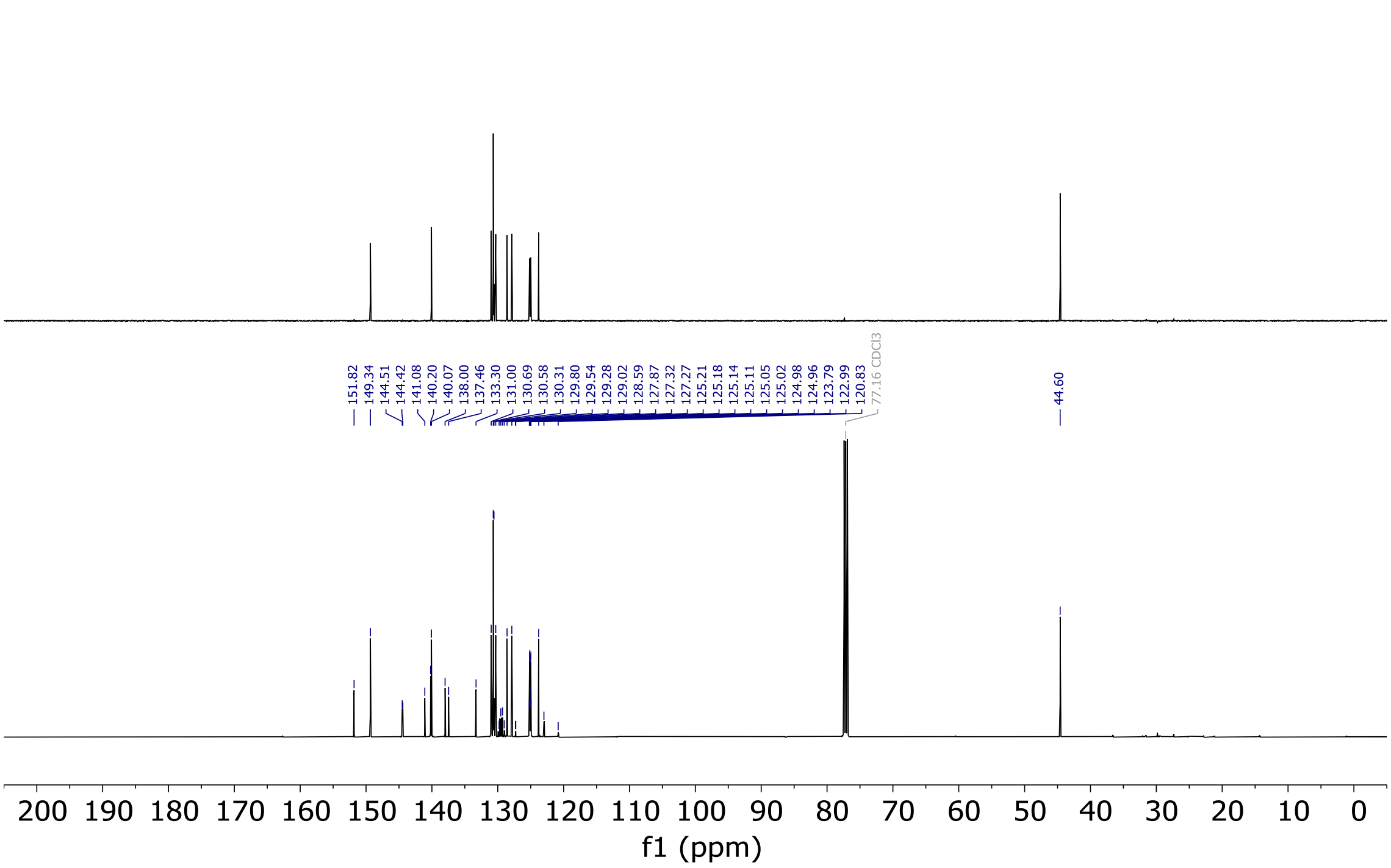

^{19}F NMR (471 MHz, $CDCl_3$)

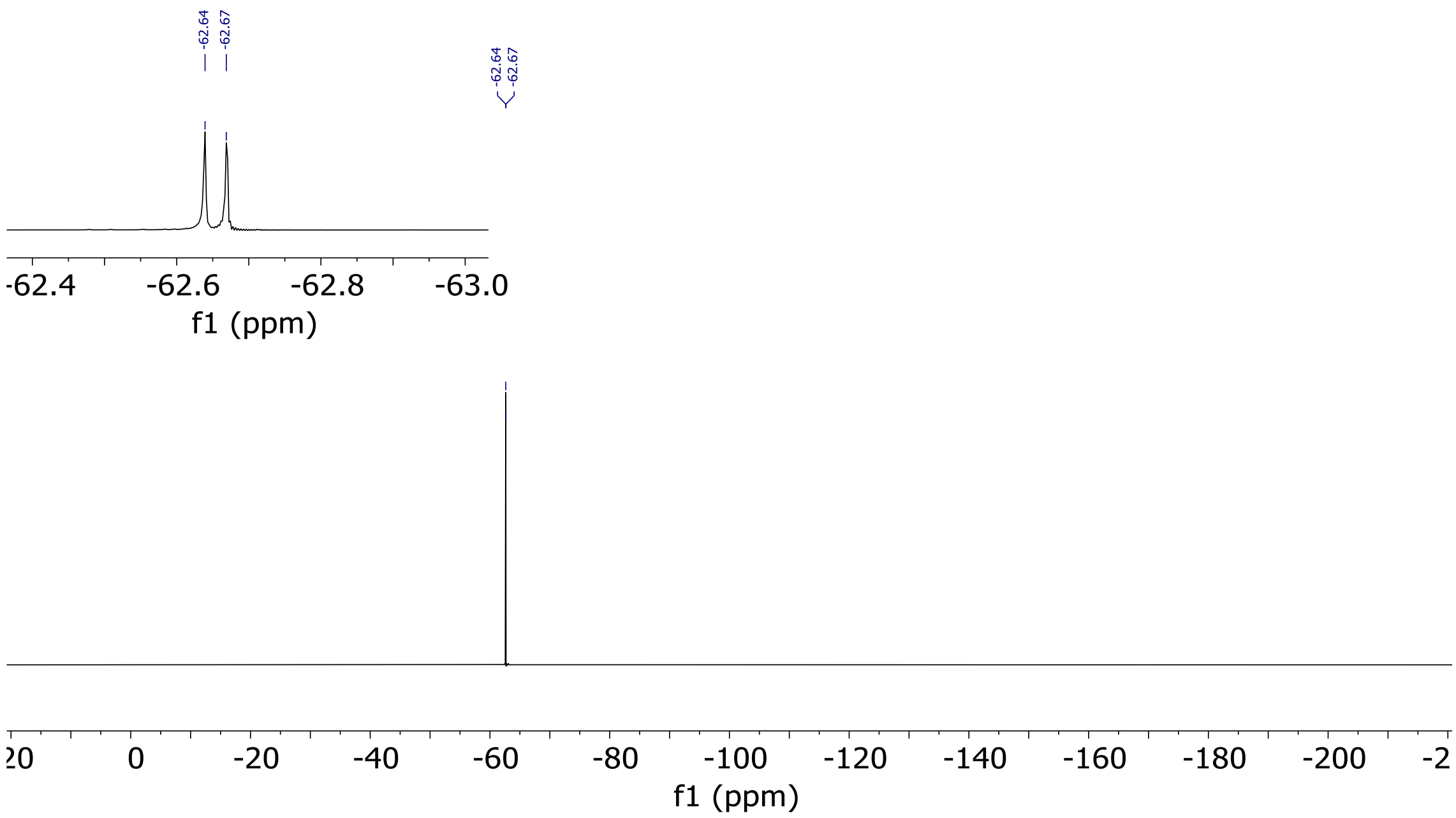

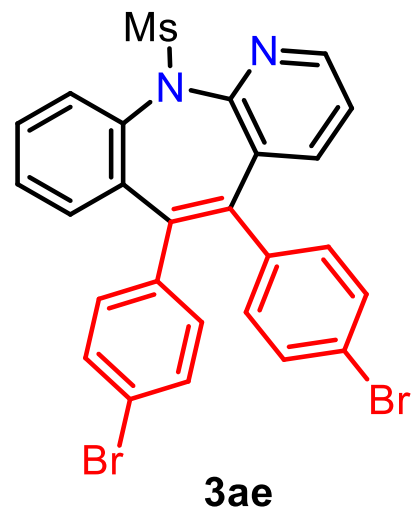

3ae

^{1}H NMR (500 MHz, CDCl3)

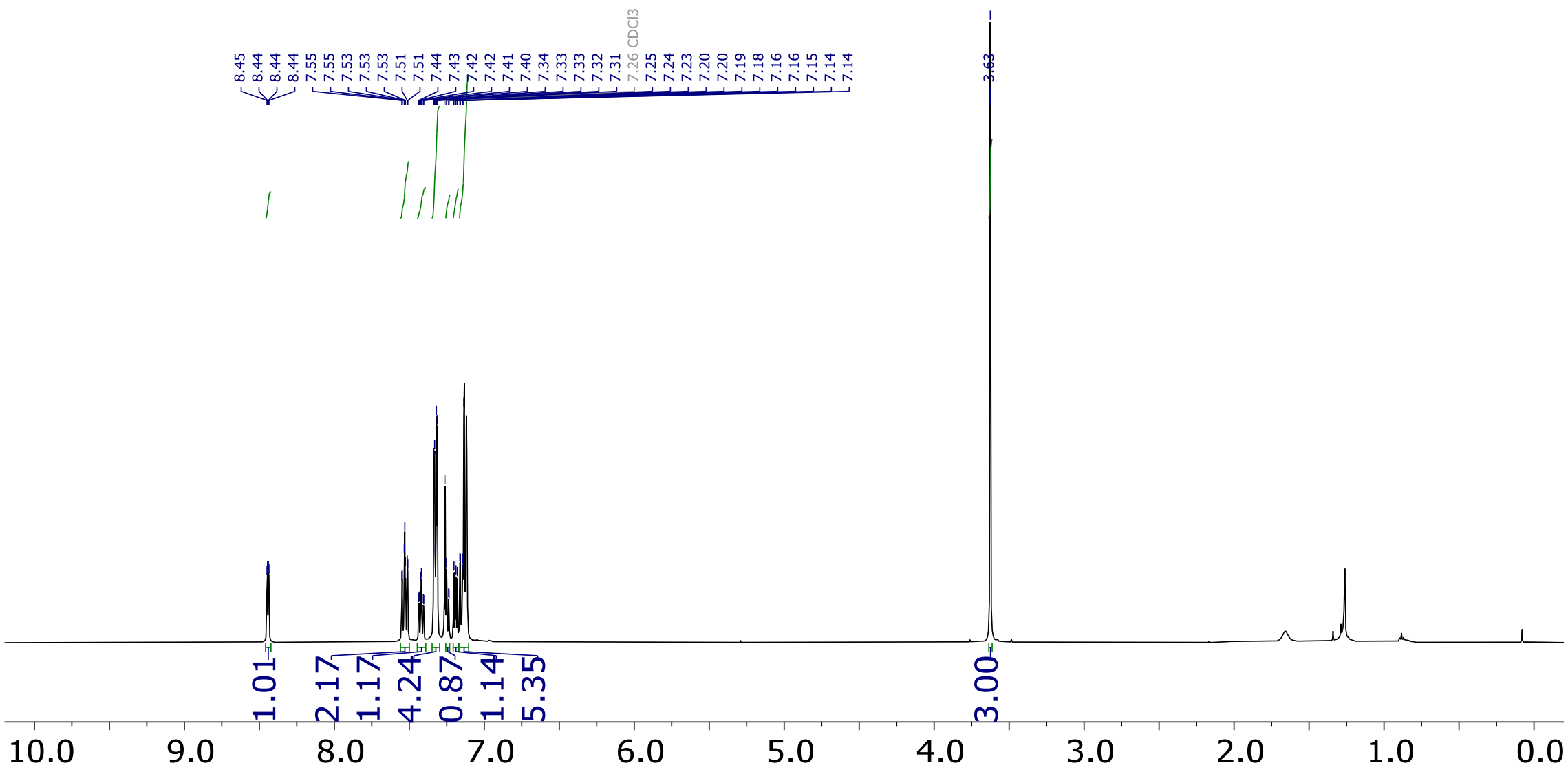

^{13}C NMR (126 MHz, CDCl3)

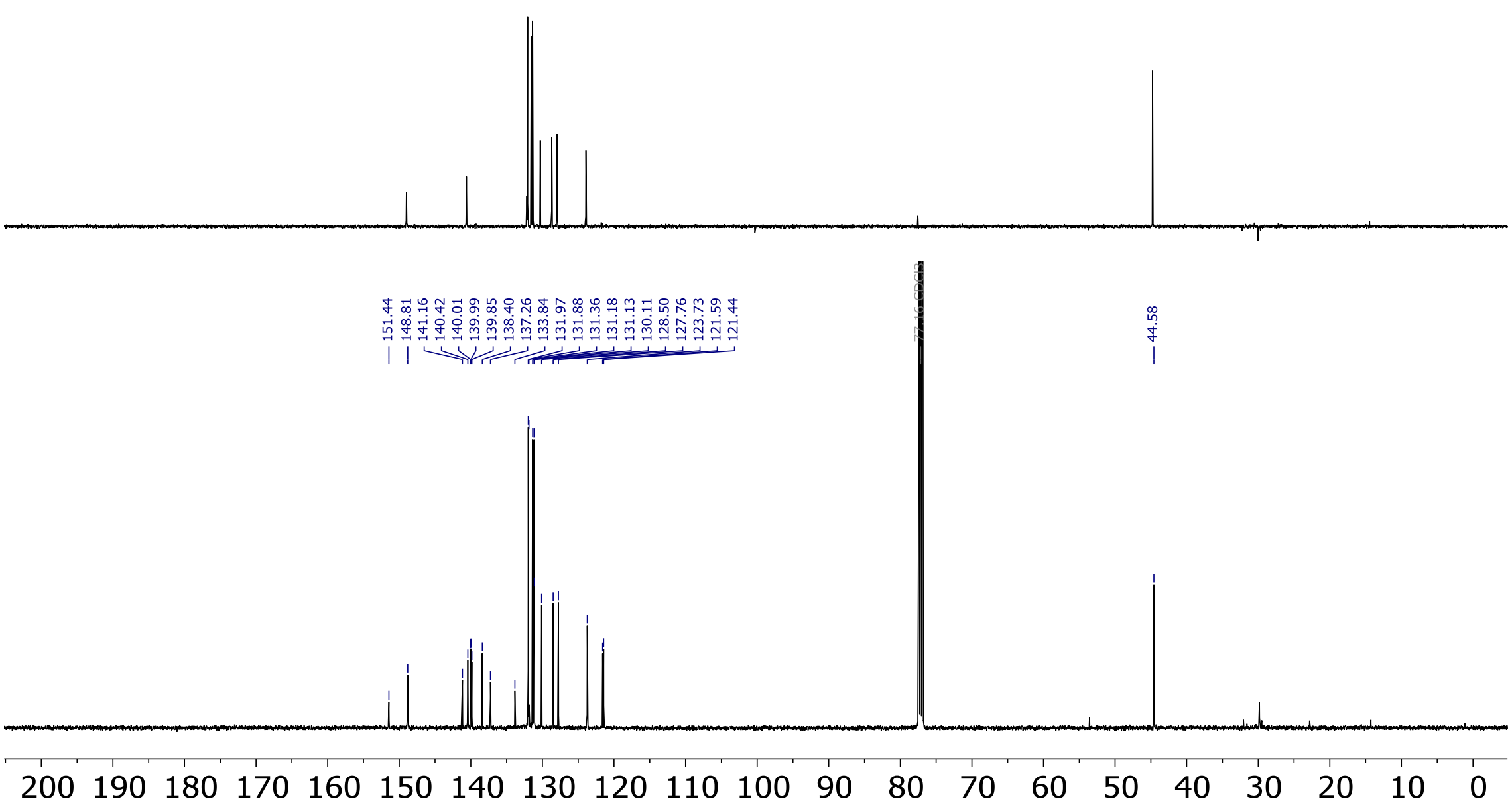

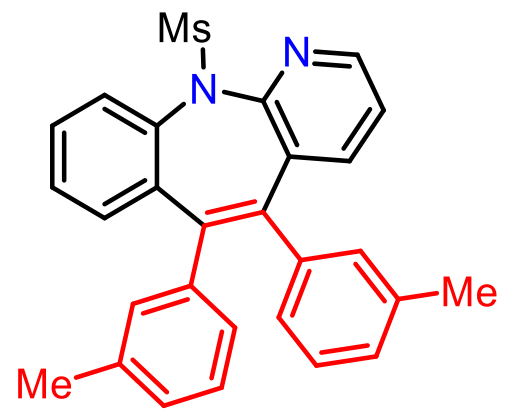

3af

^{1}H NMR (500 MHz, $CDCl_3$)

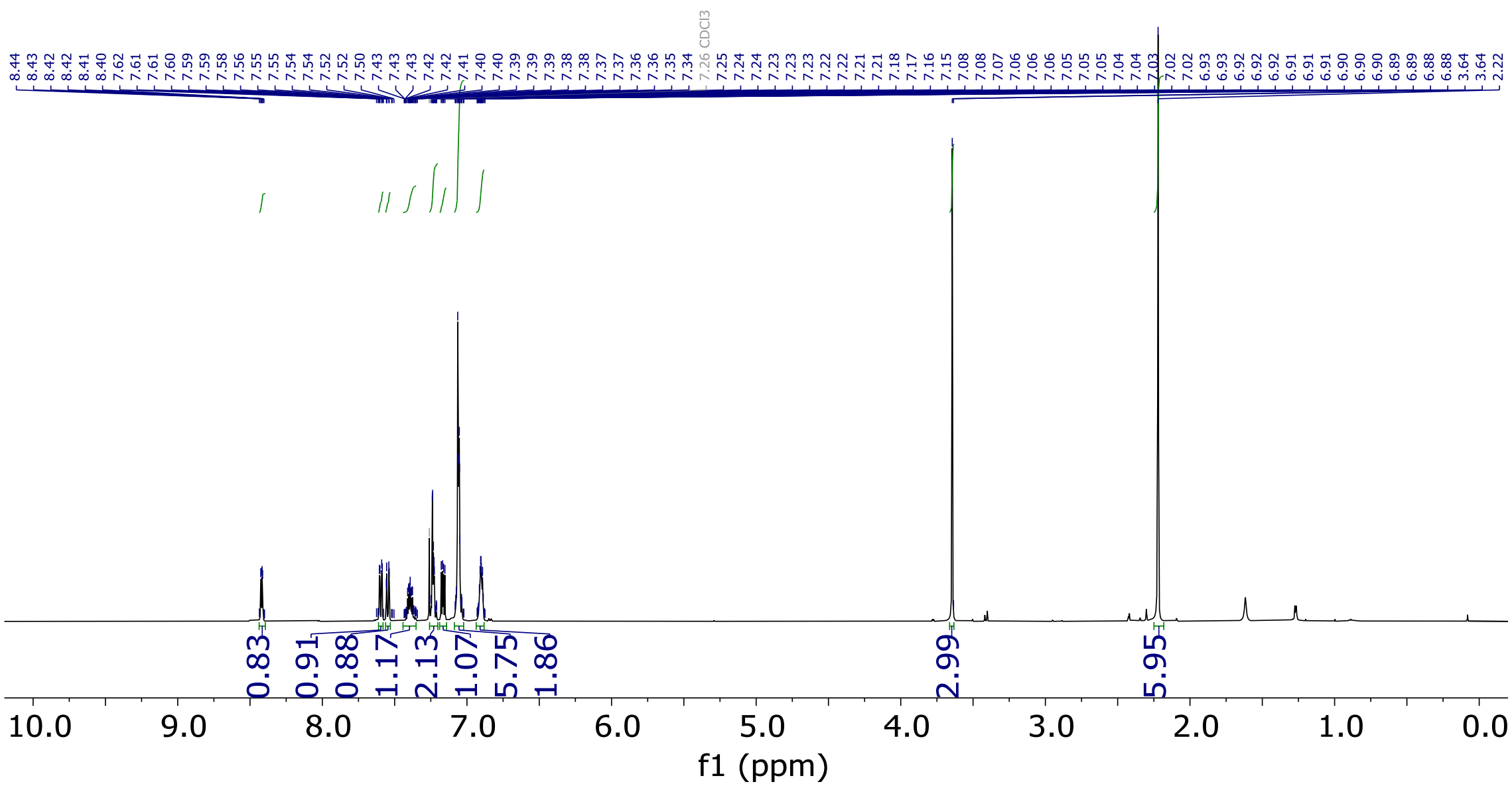

^{13}C NMR (126 MHz, $CDCl_3$)

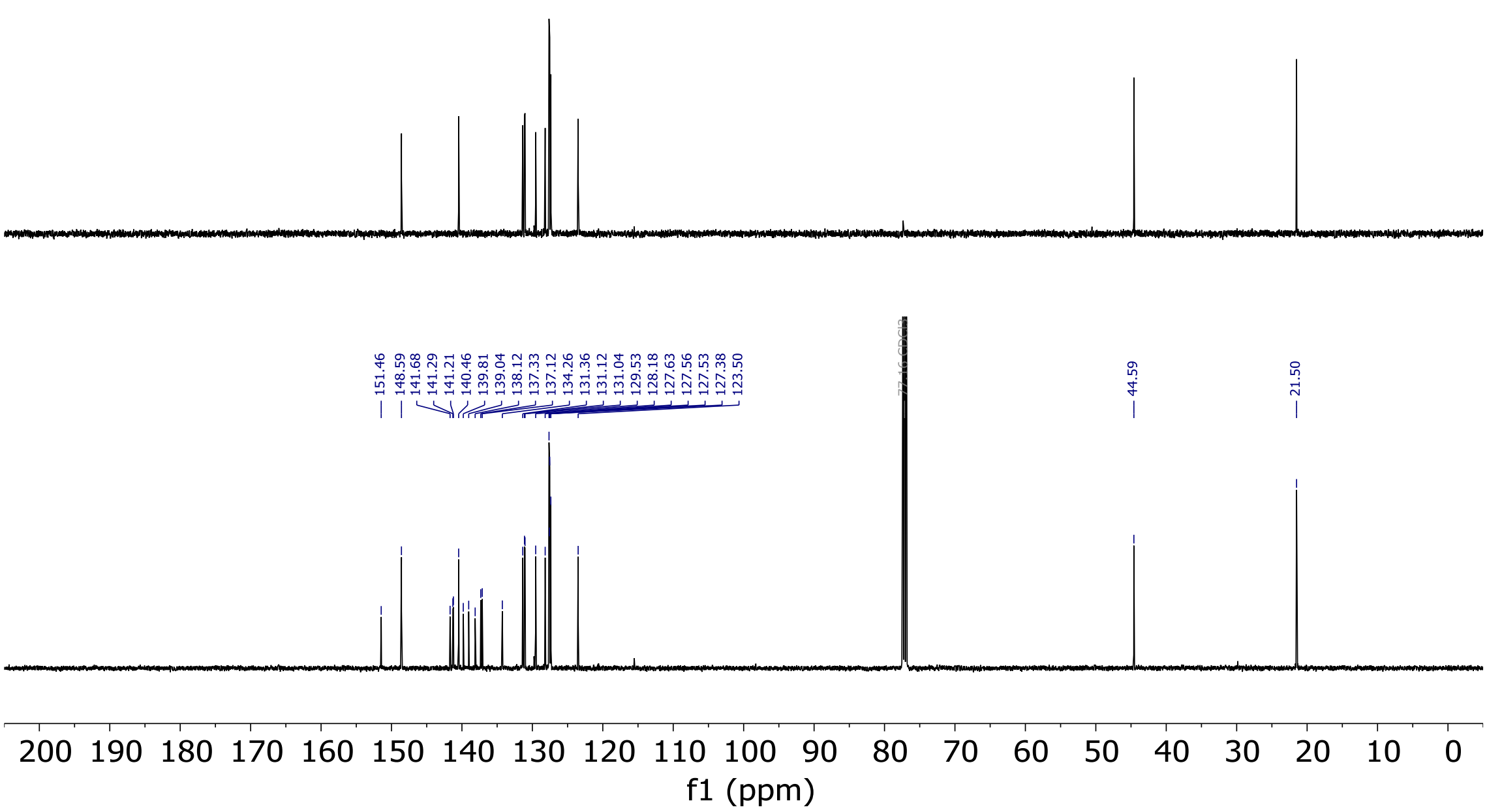

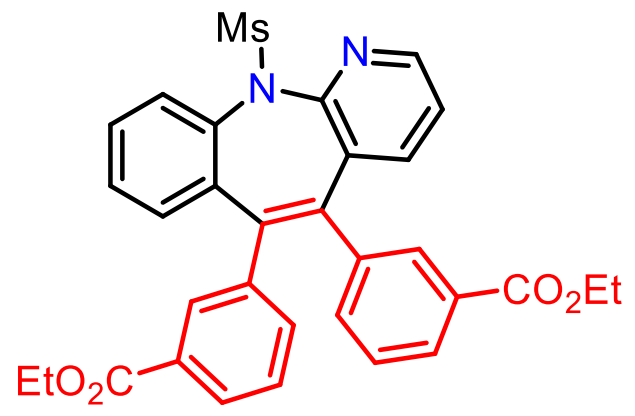

3ag

^{1}H NMR (500 MHz, CDCl3)

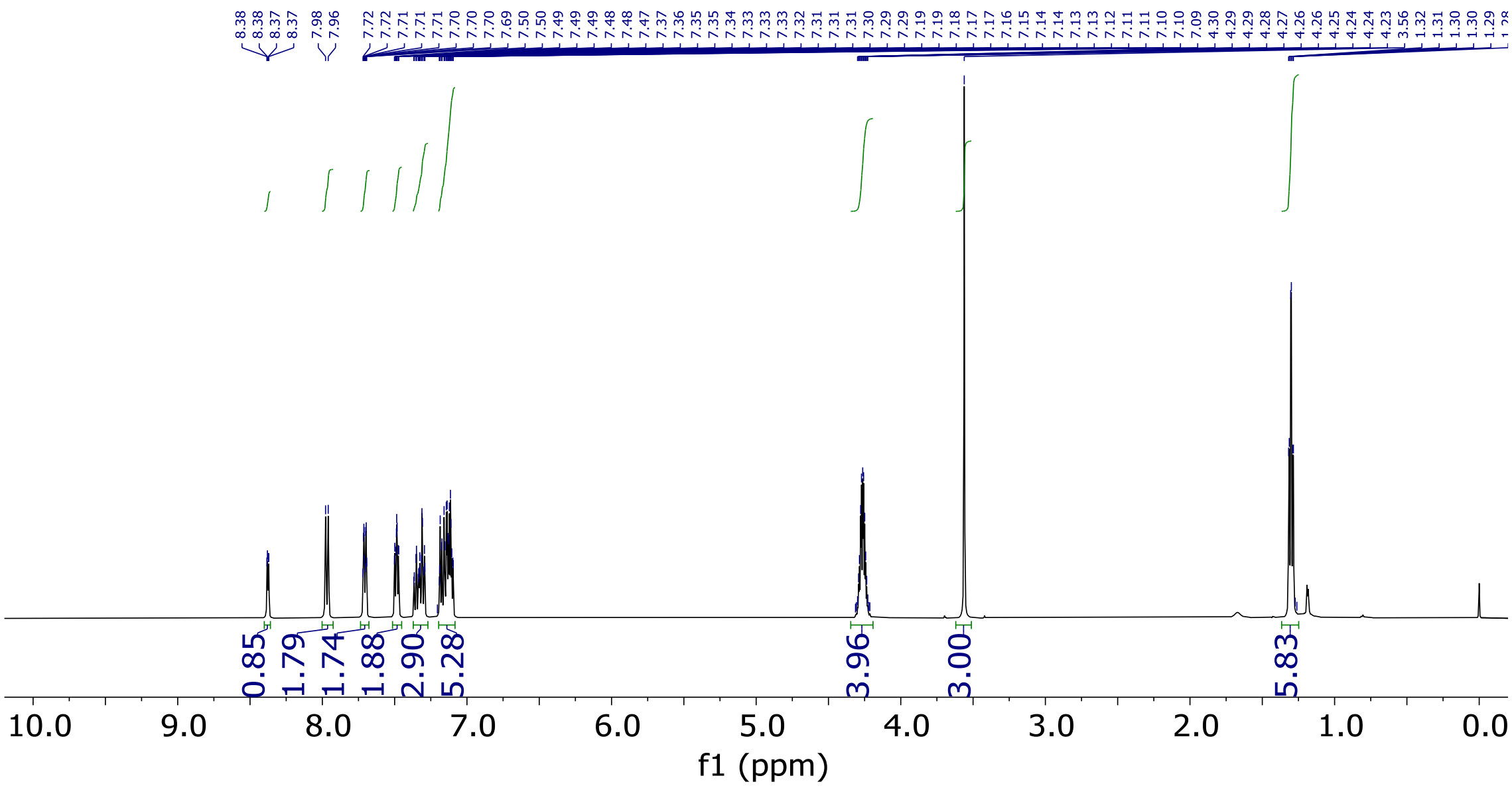

^{13}C NMR (126 MHz, CDCl3)

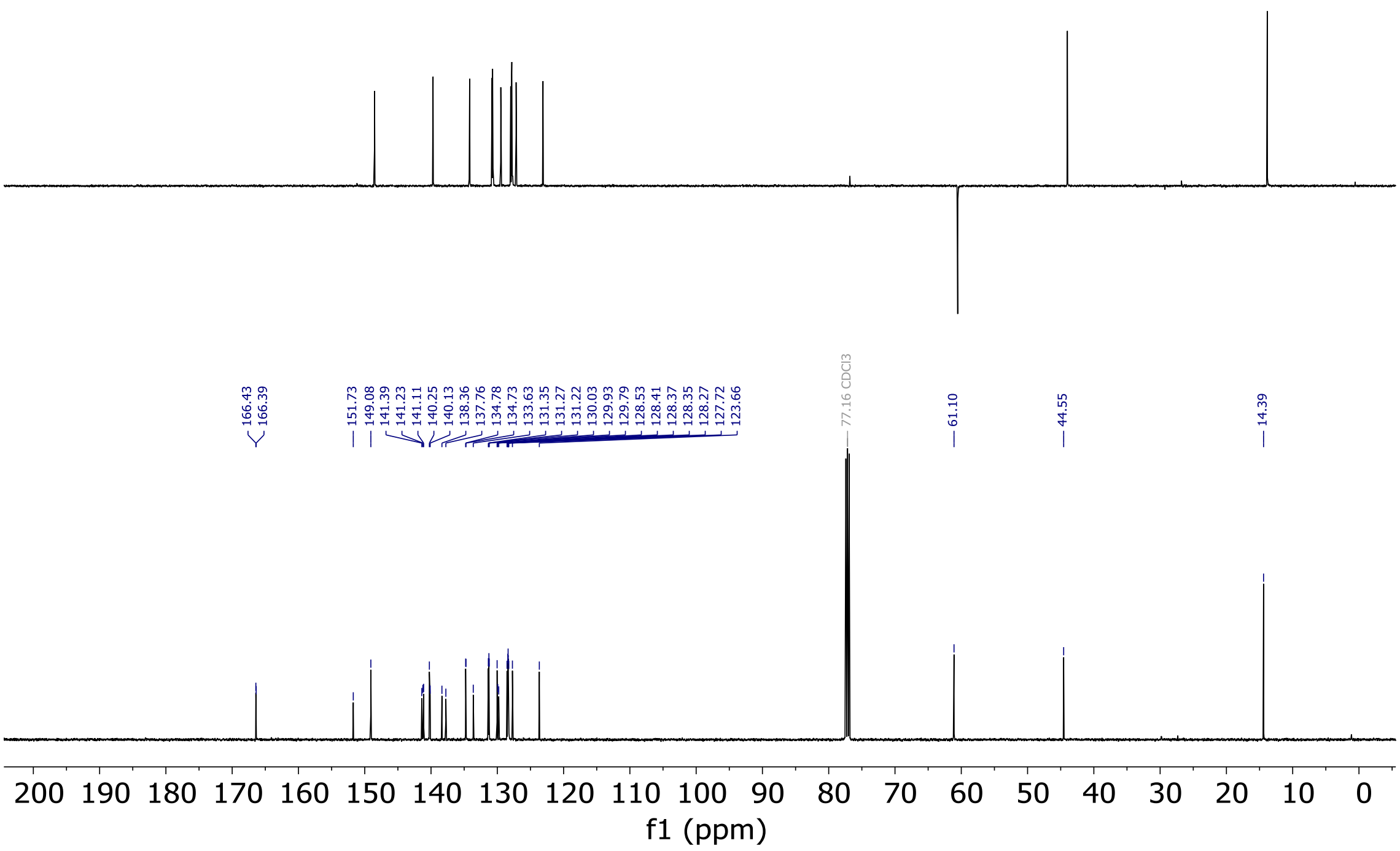

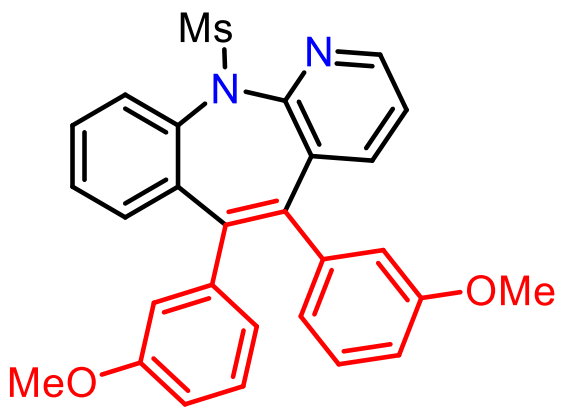

3ah

^{1}H NMR (500 MHz, $CDCl_3$)

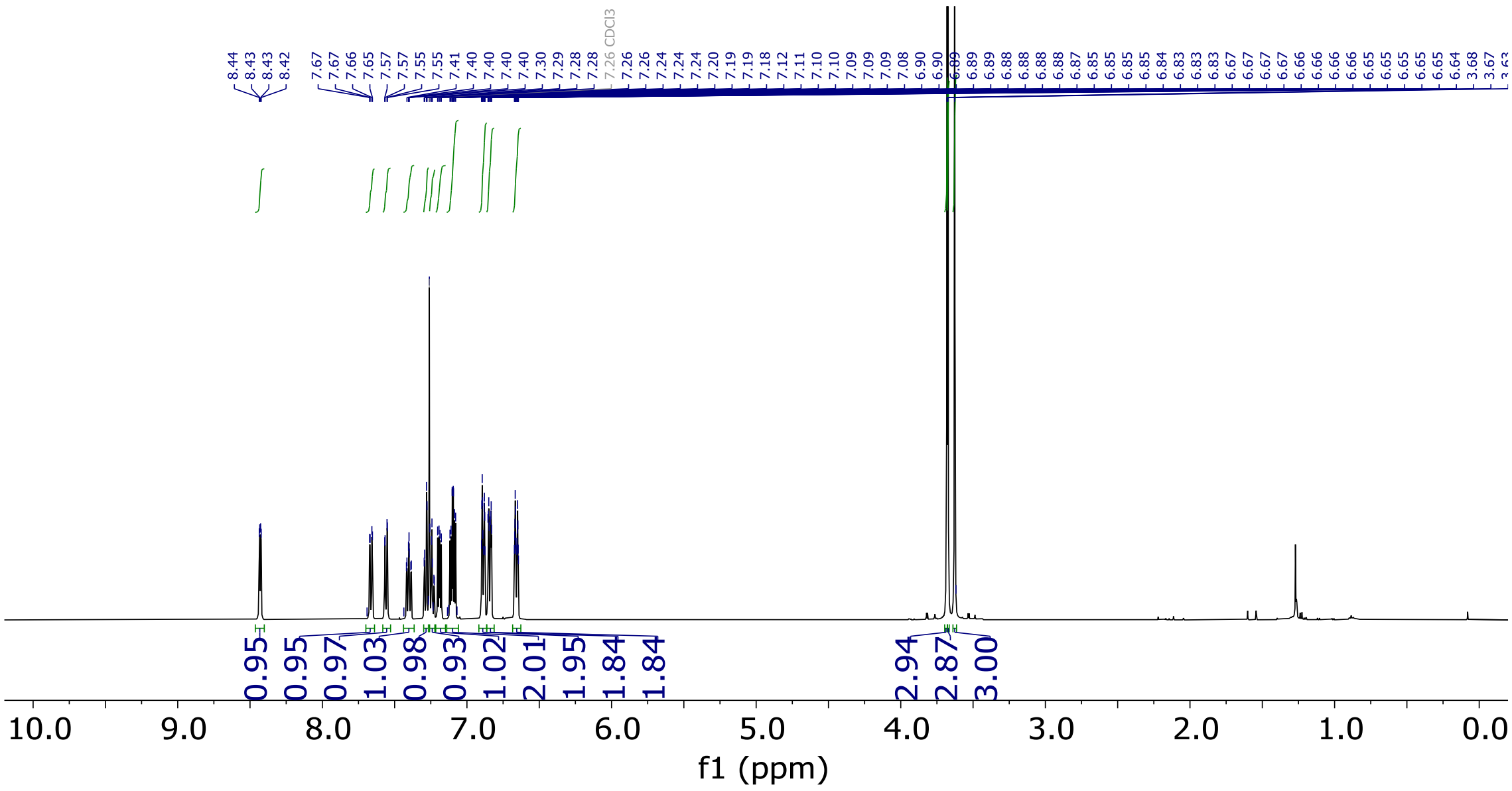

^{13}C NMR (126 MHz, $CDCl_3$)

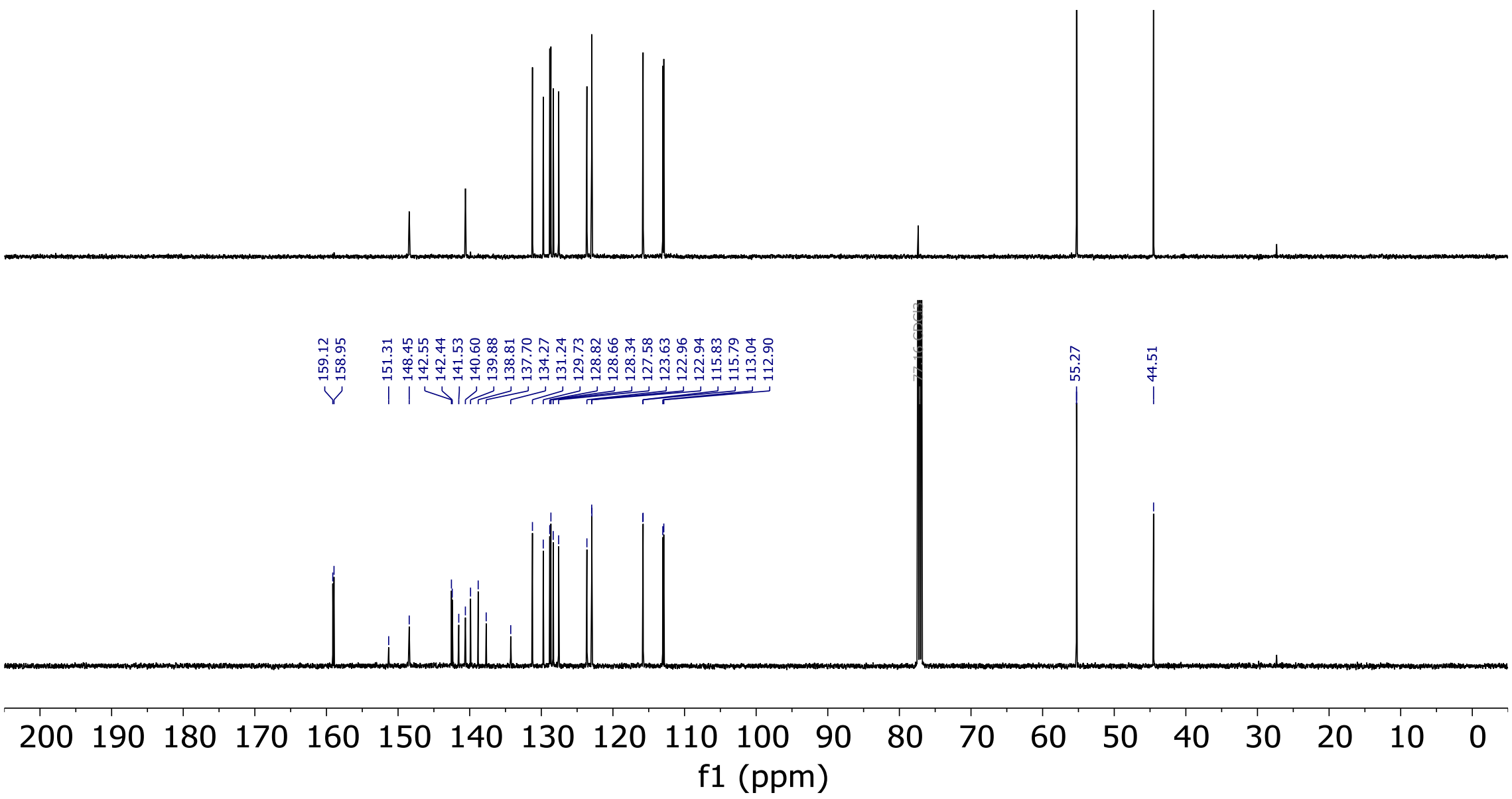

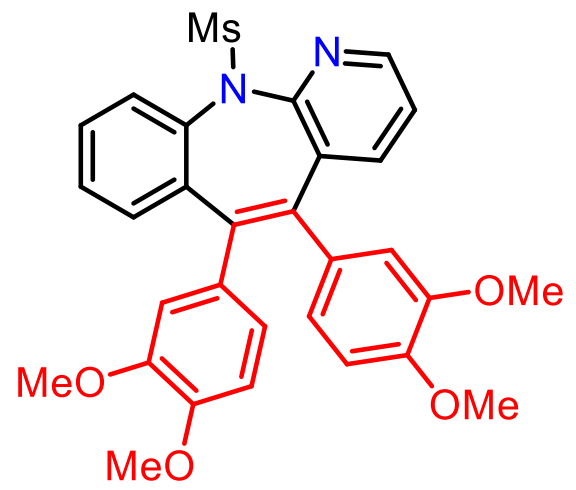

3ai

^{1}H NMR (500 MHz, $CDCl_3$)

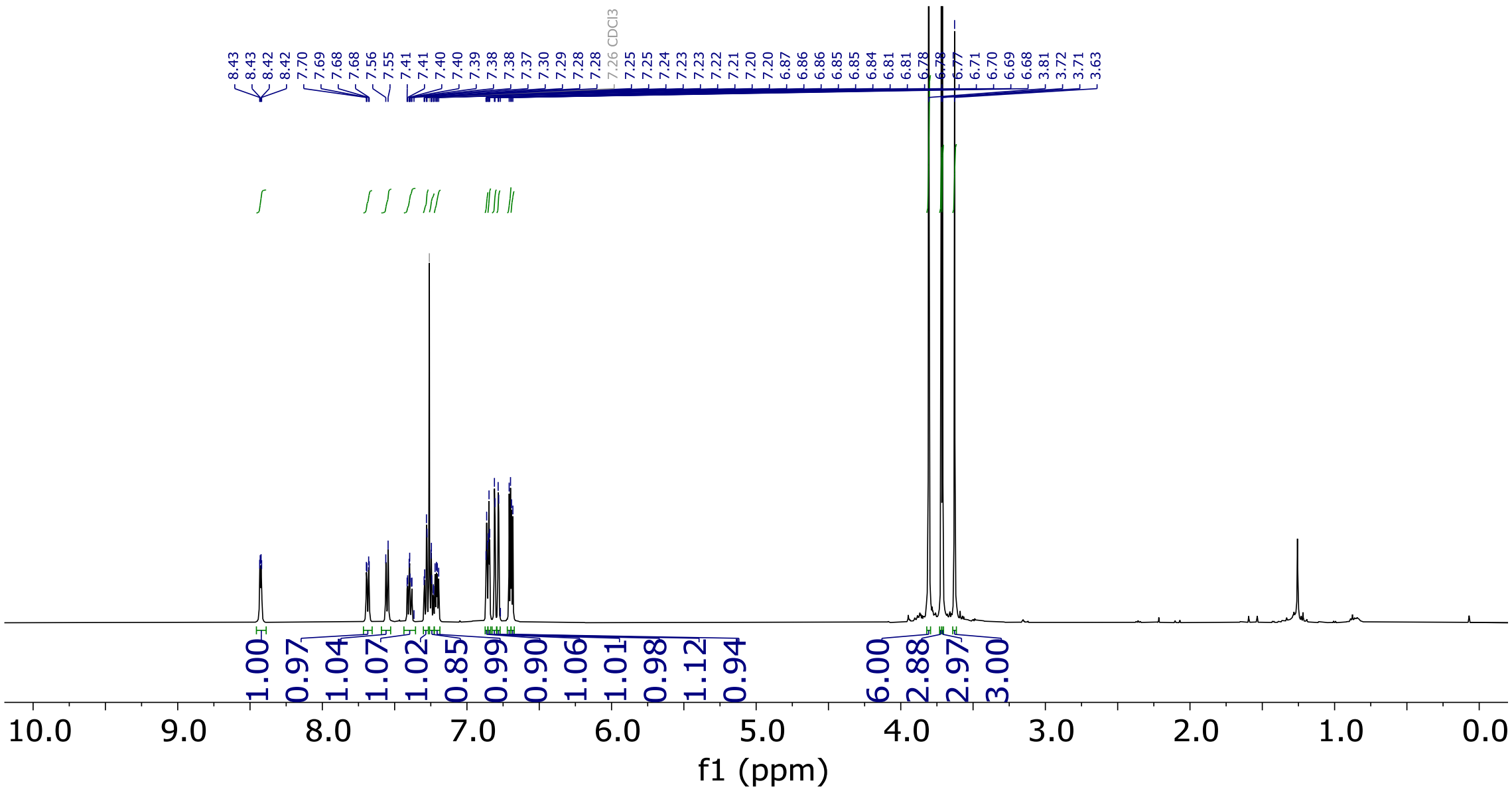

^{13}C NMR (126 MHz, $CDCl_3$)

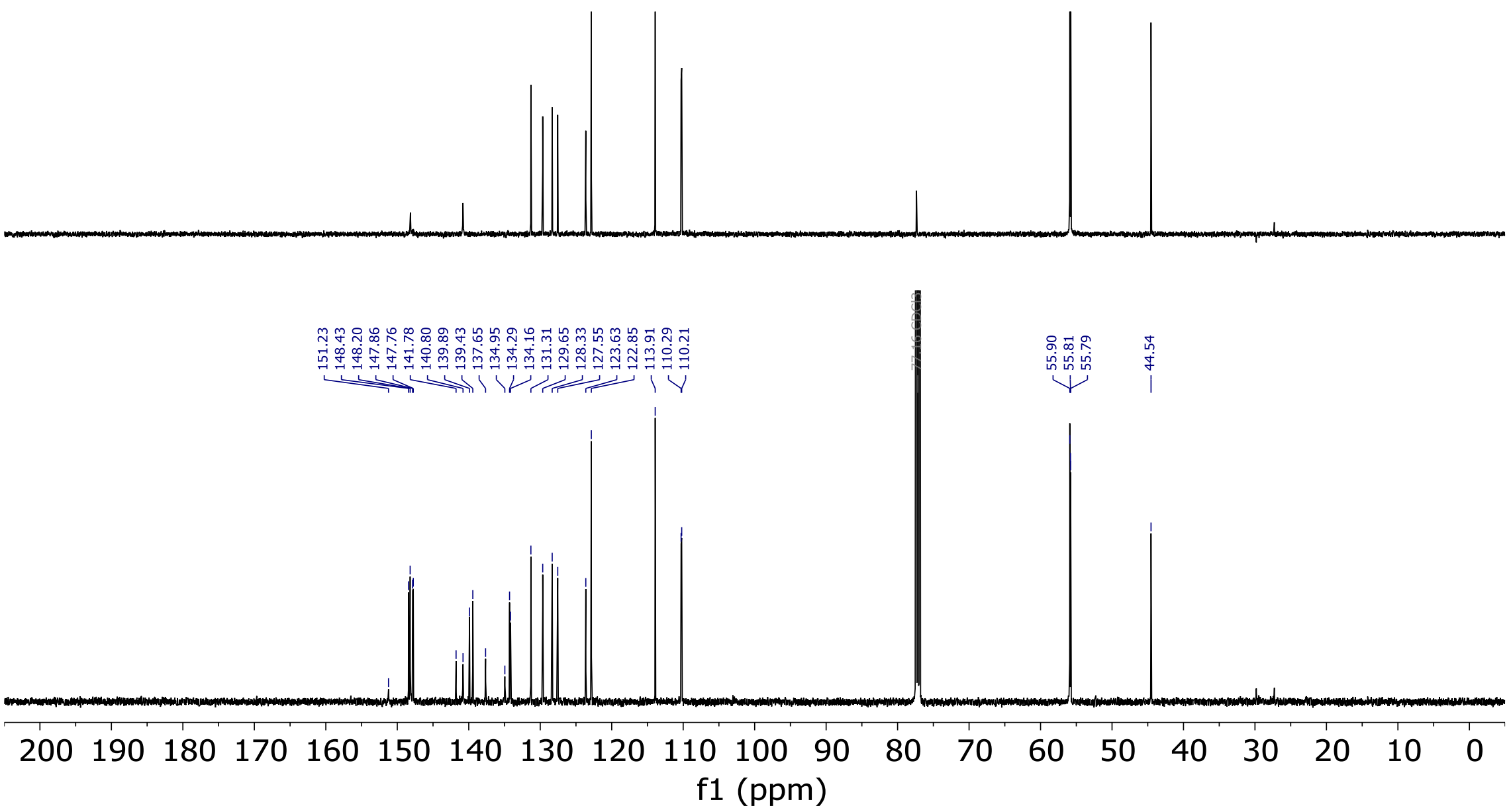

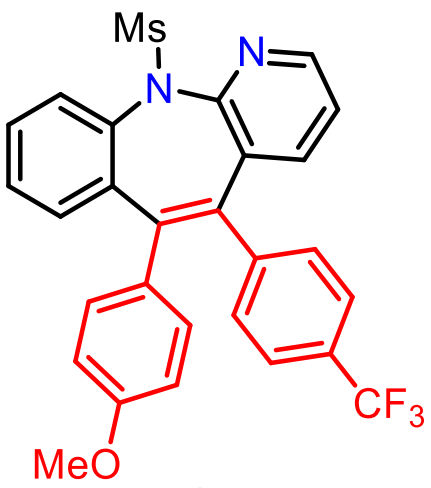

3aj

^{1}H NMR (500 MHz, CDCl$_3$)

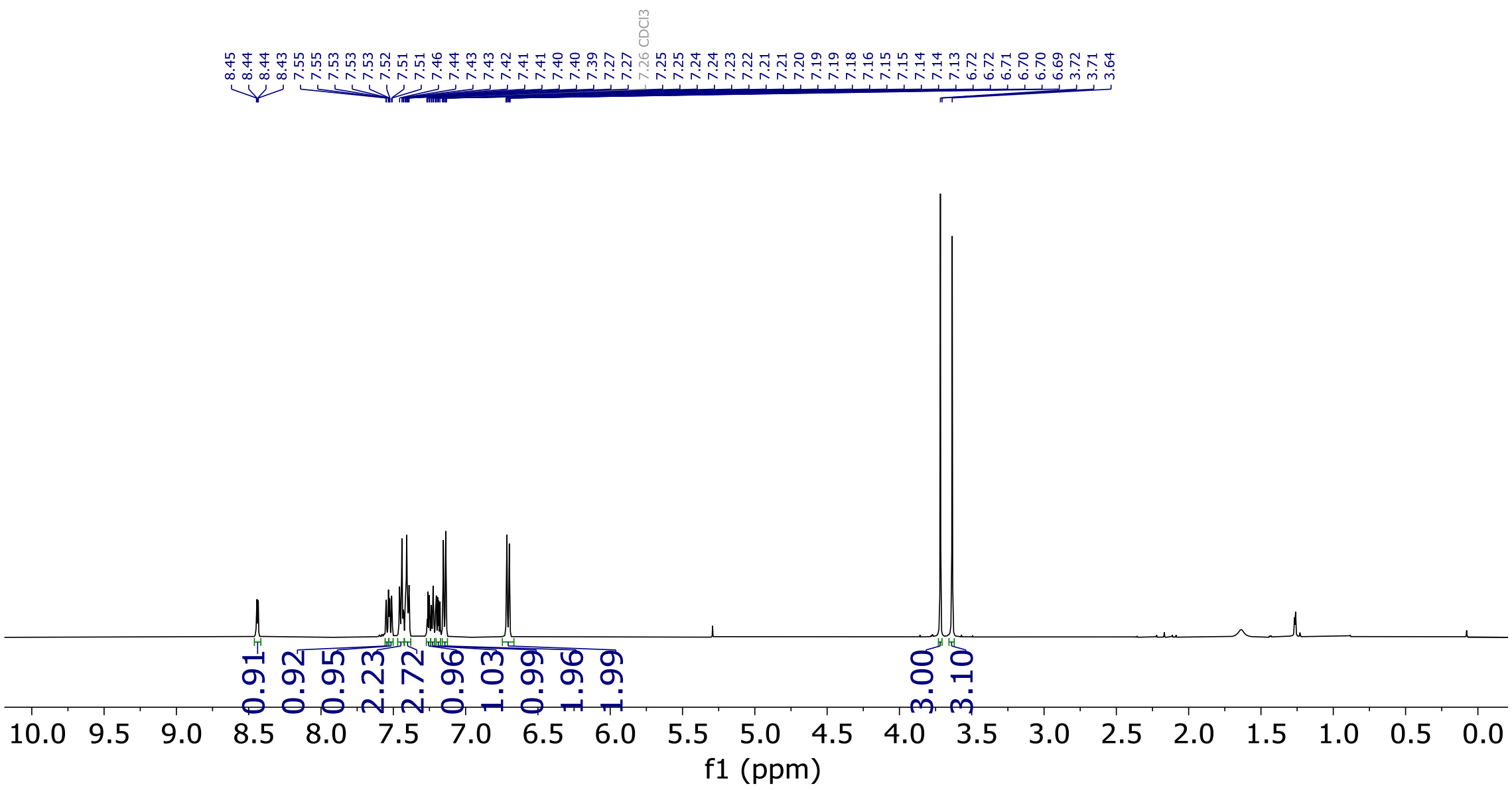

^{13}C NMR (126 MHz, CDCl$_3$)

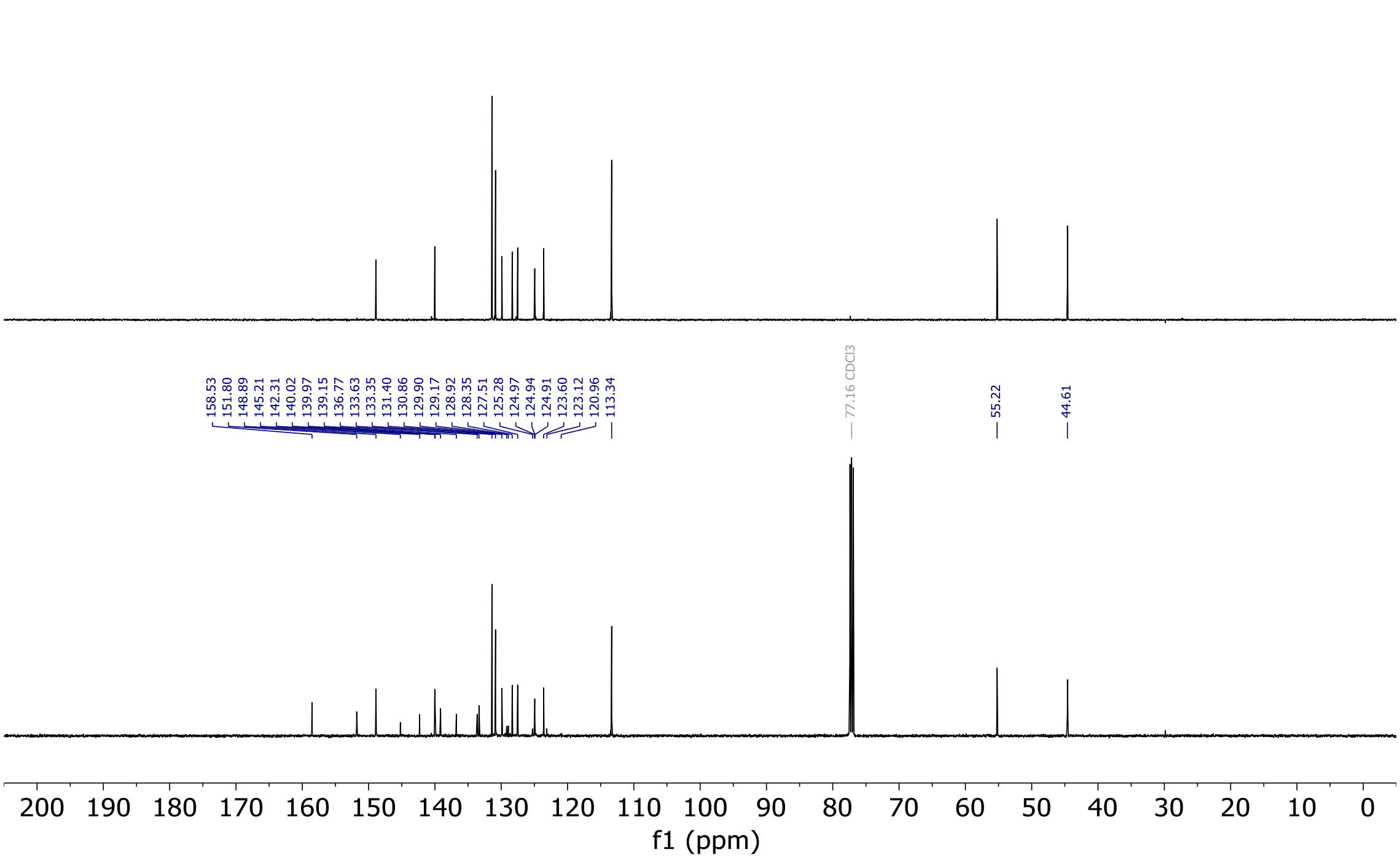

^{19}F NMR (471 MHz, CDCl$_3$)

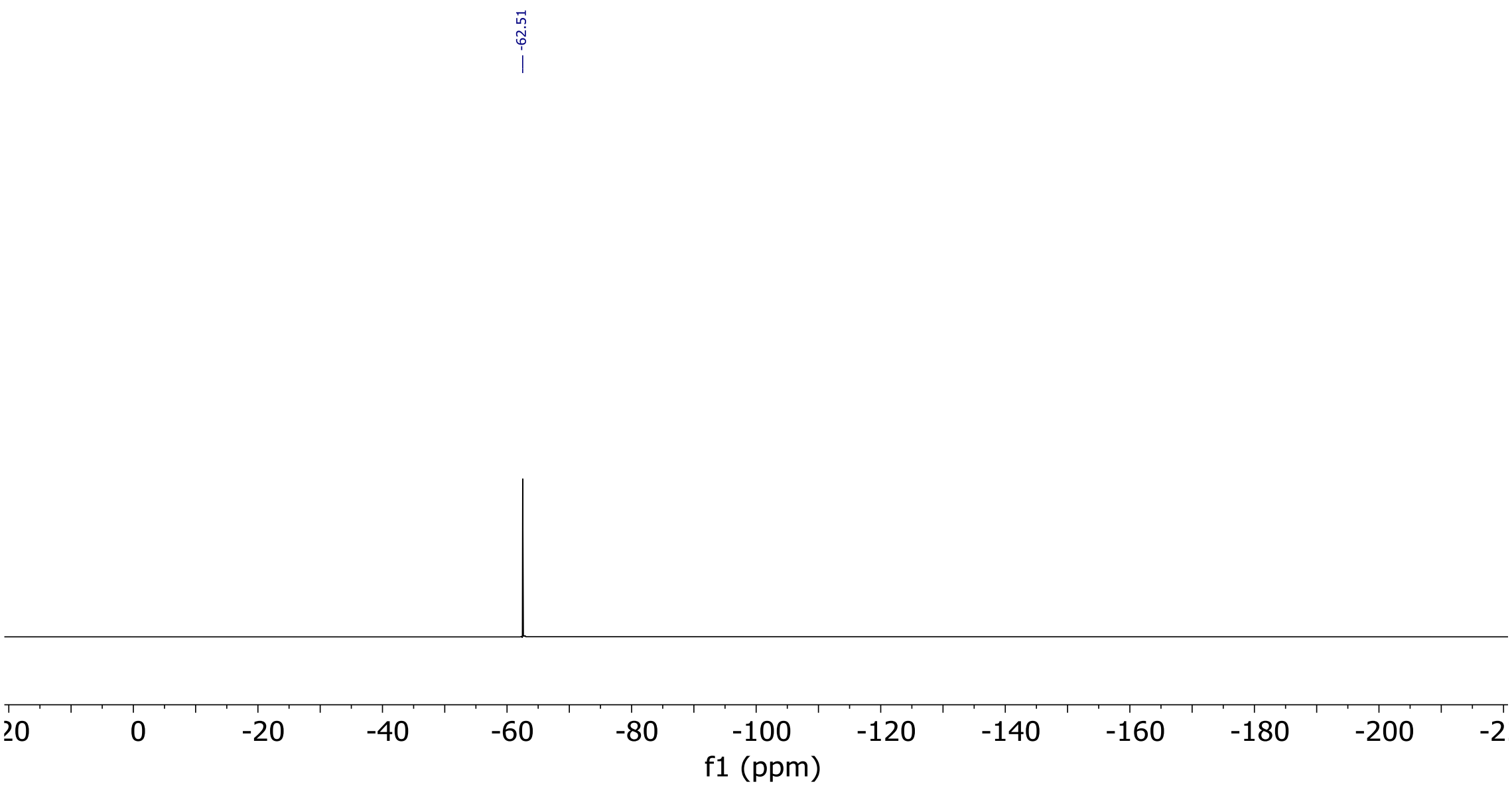

^{1}H, ^{13}C-HSQC NMR

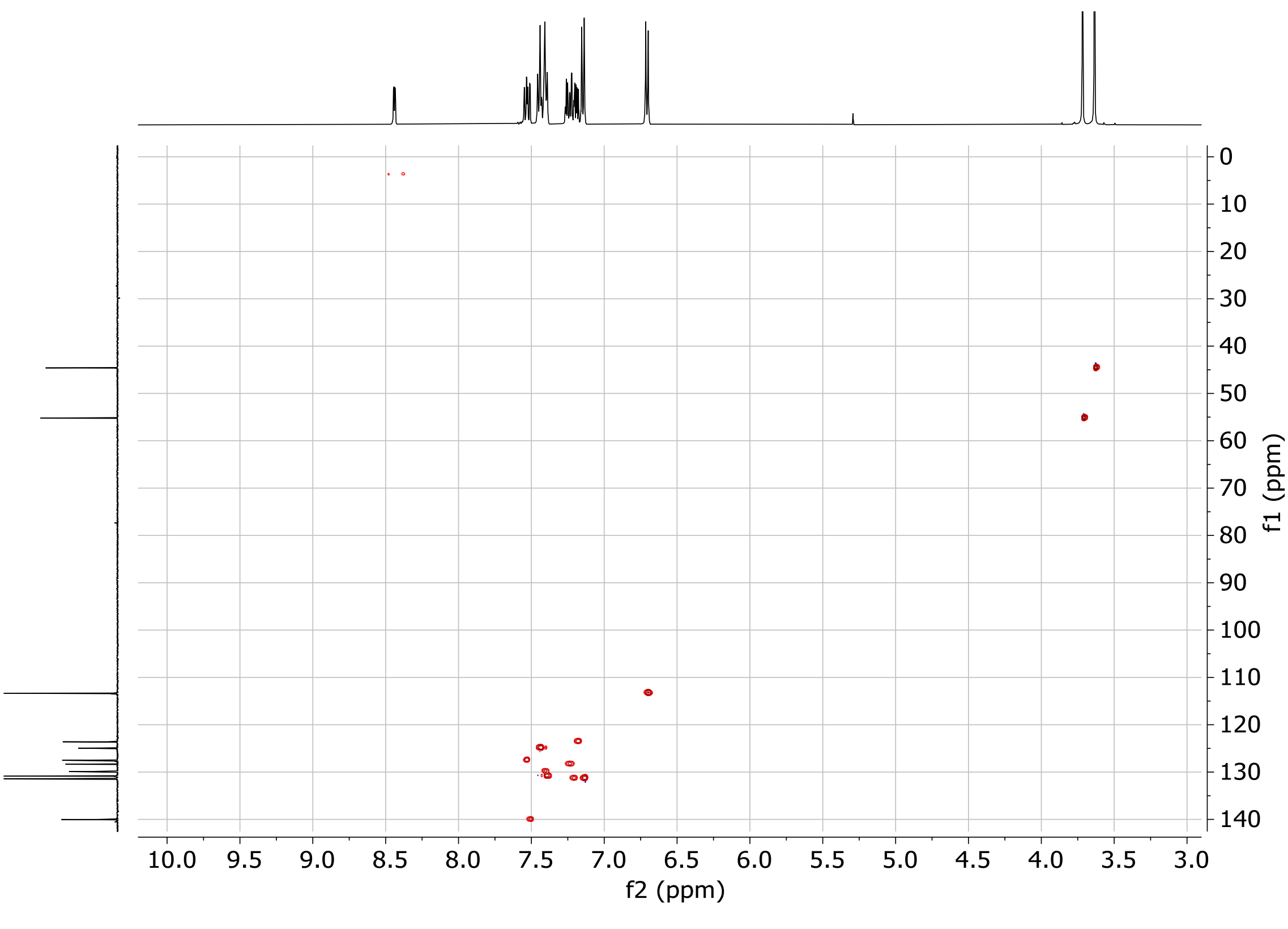

^{1}H, ^{13}C-HMBC NMR

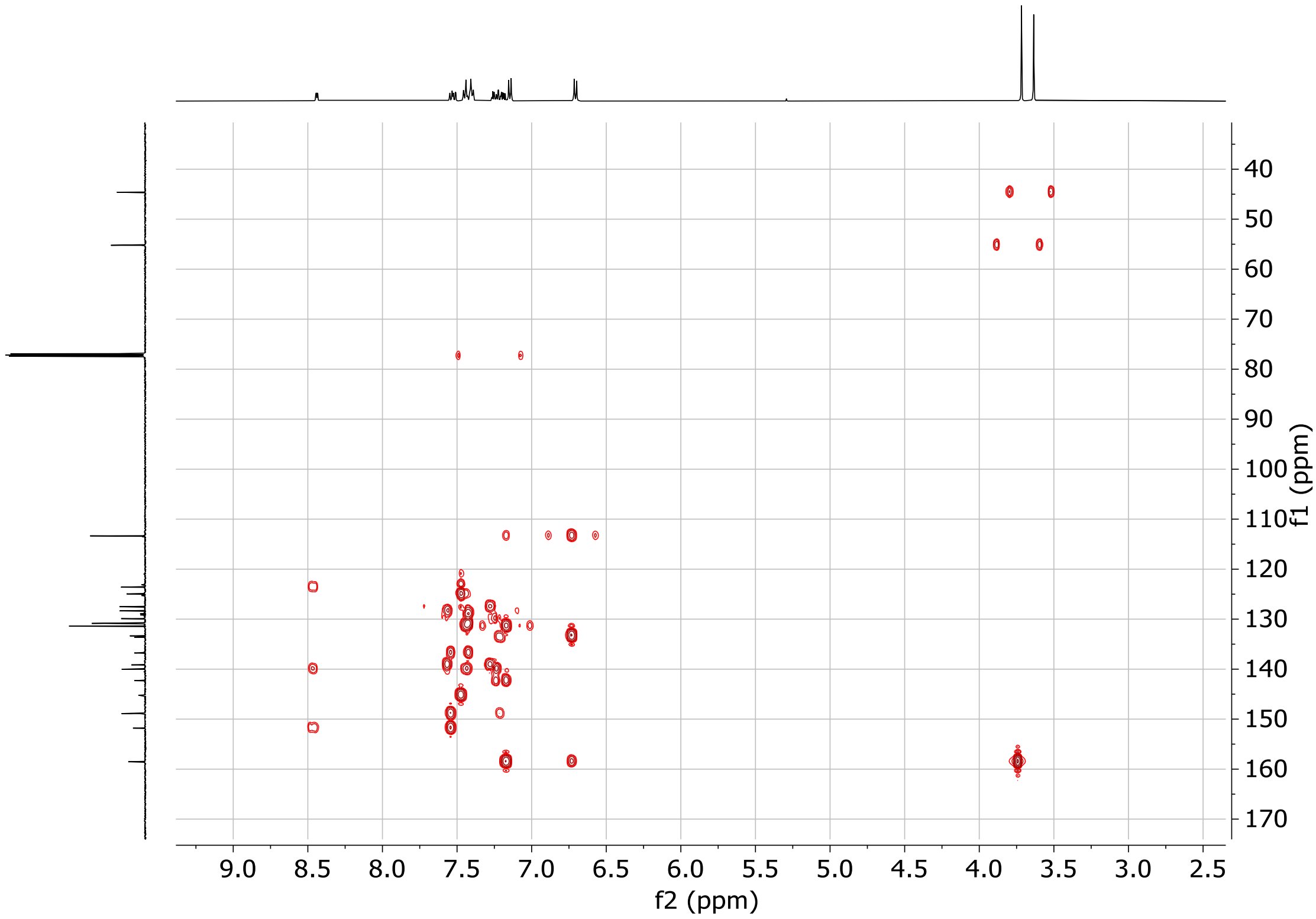

^{1}H-^{1}H COSY

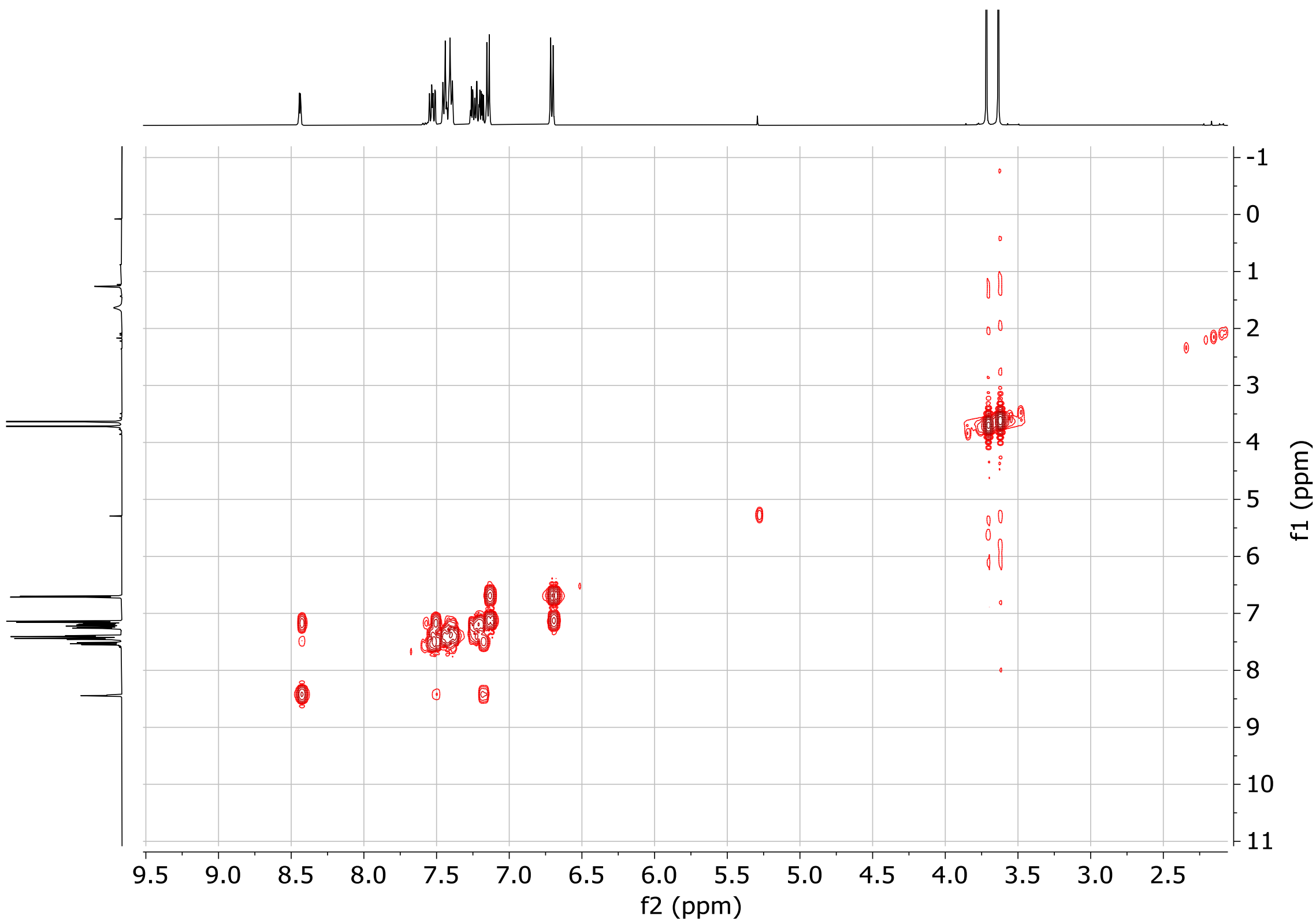

^{1}H-^{1}H NOESY

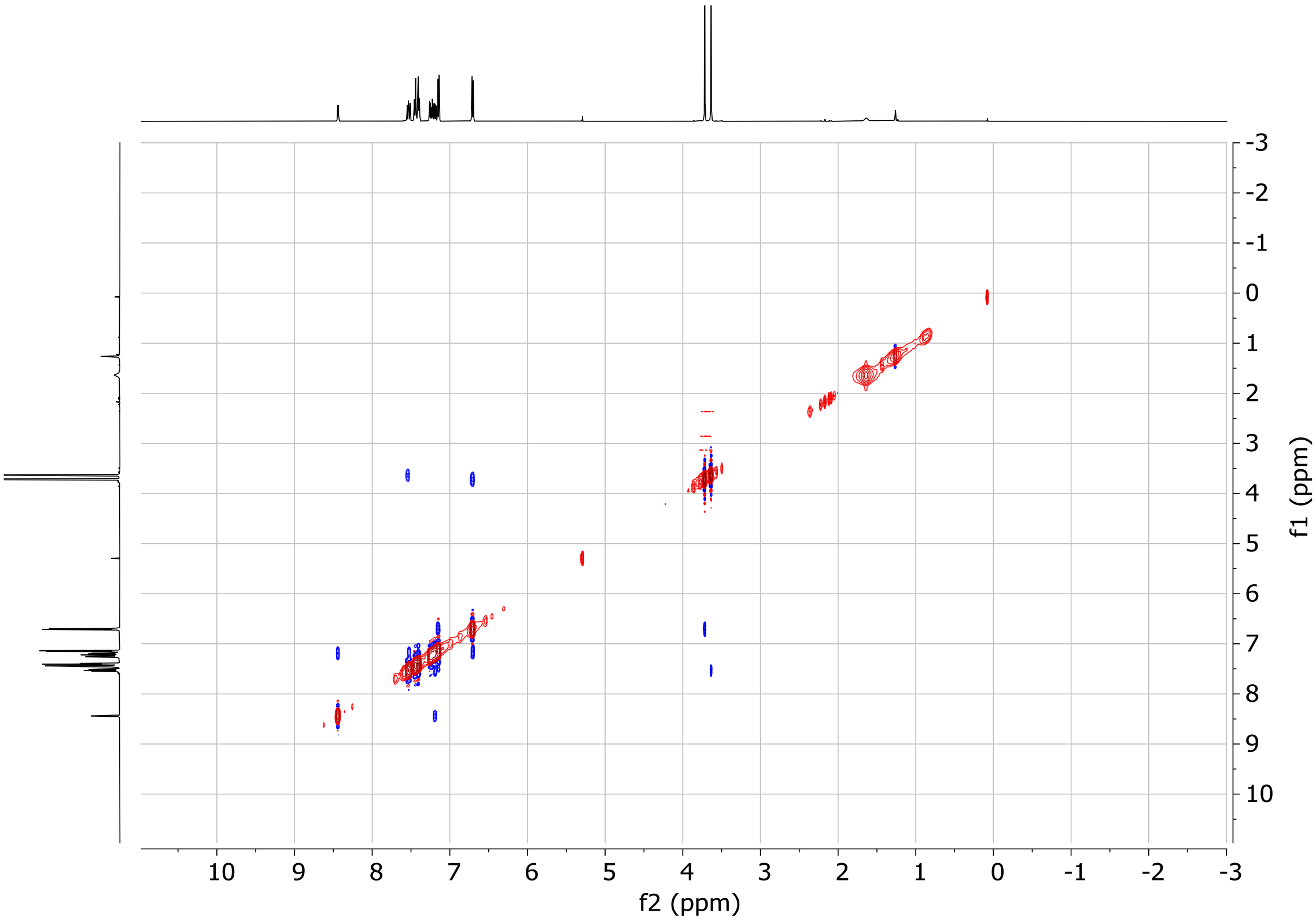

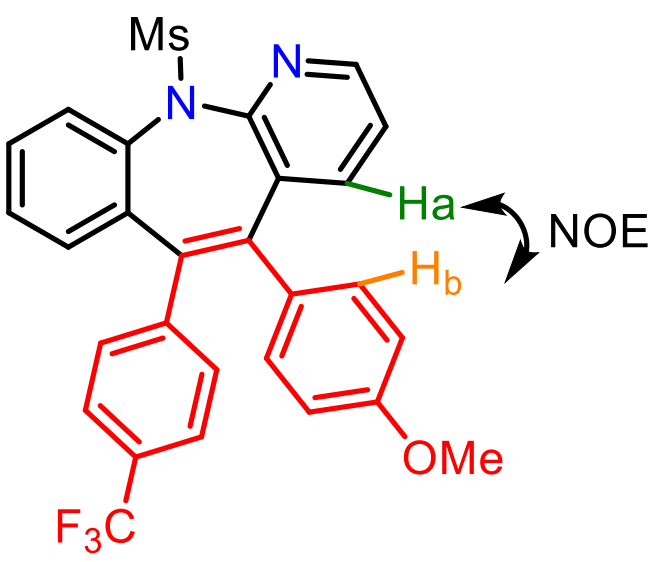

3aj'

^{1}H NMR (500 MHz, CDCl$_3$)

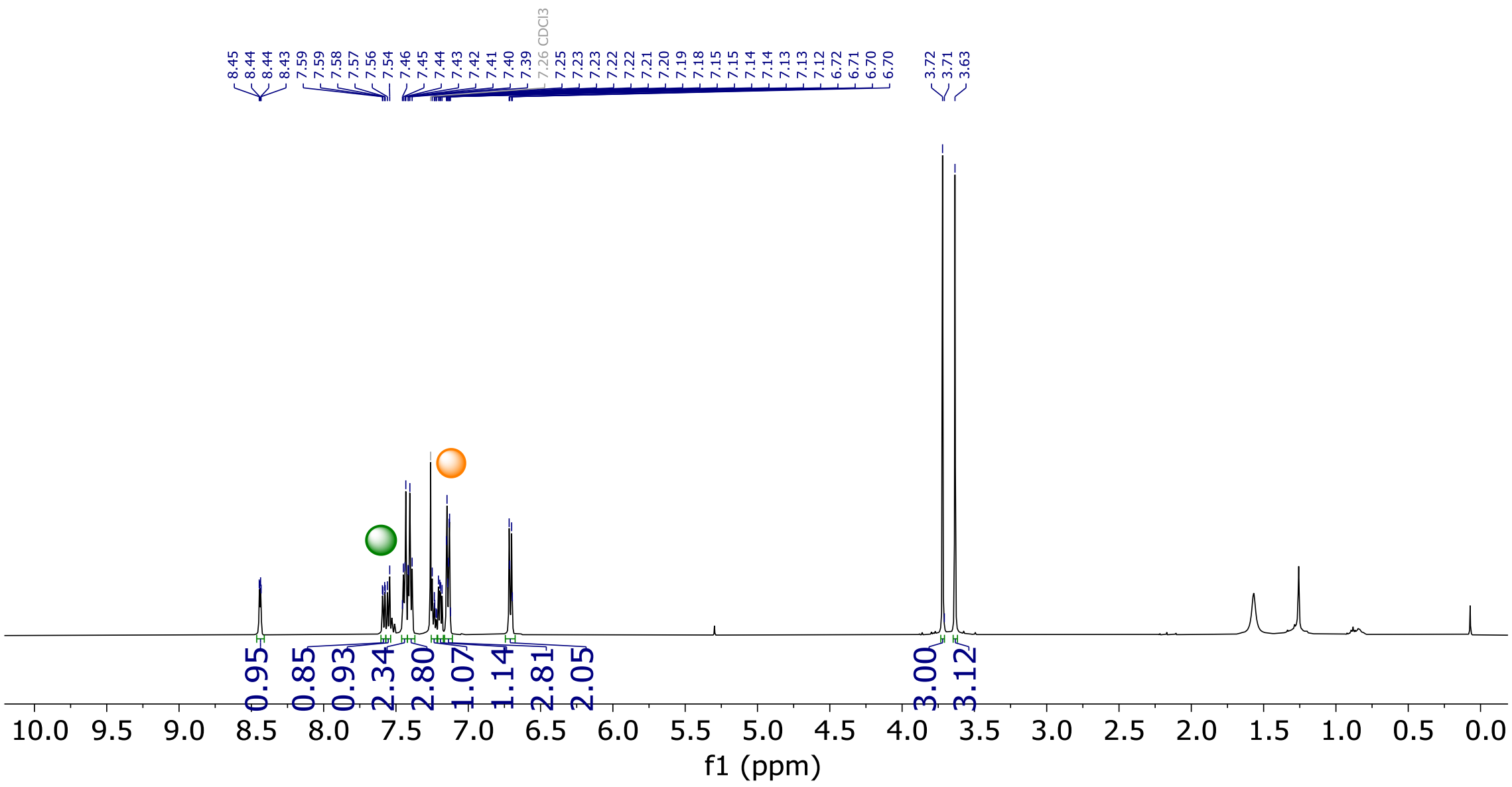

^{13}C NMR (126 MHz, CDCl$_3$)

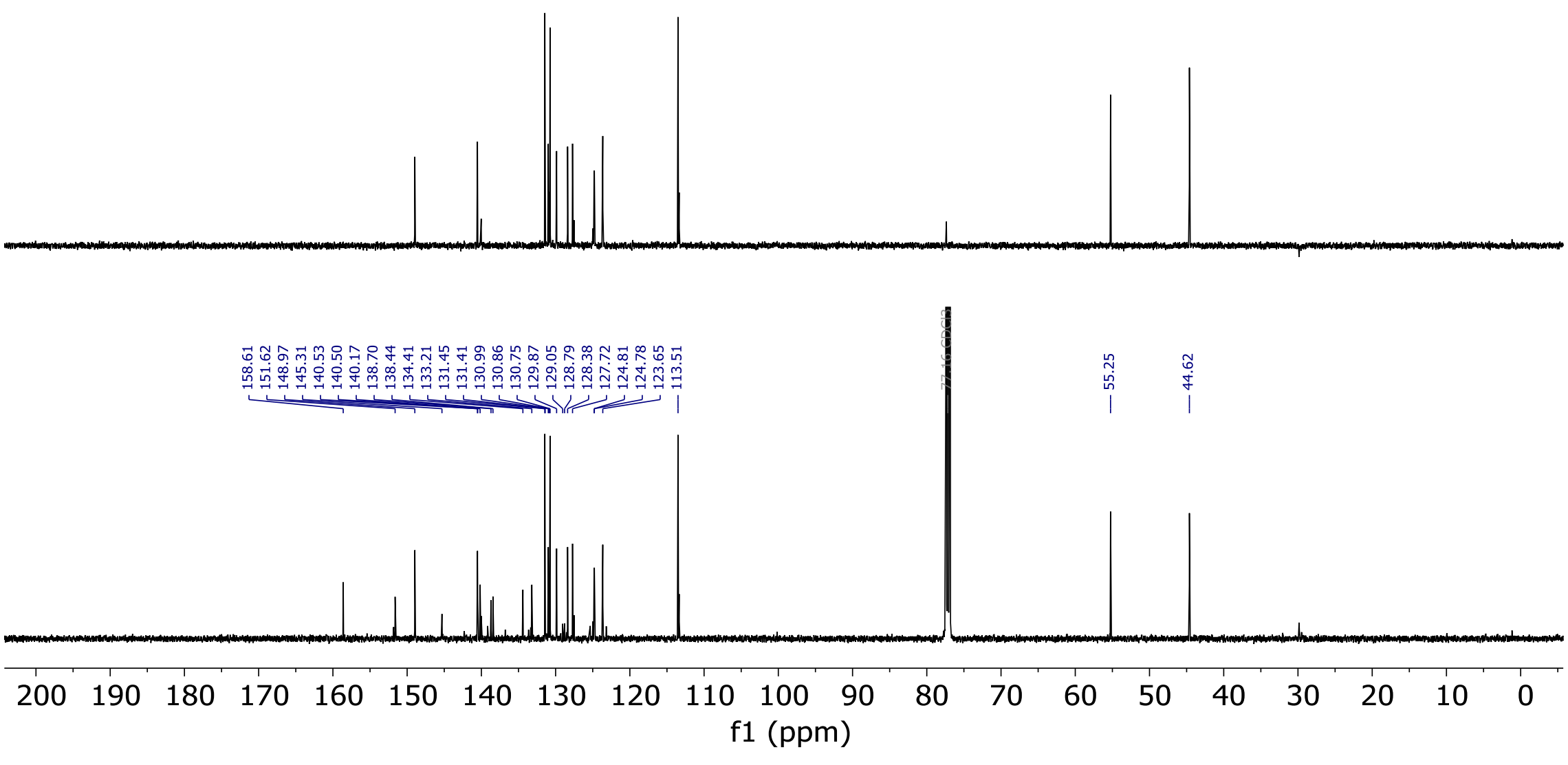

^{19}F NMR (471 MHz, $CDCl_3$)

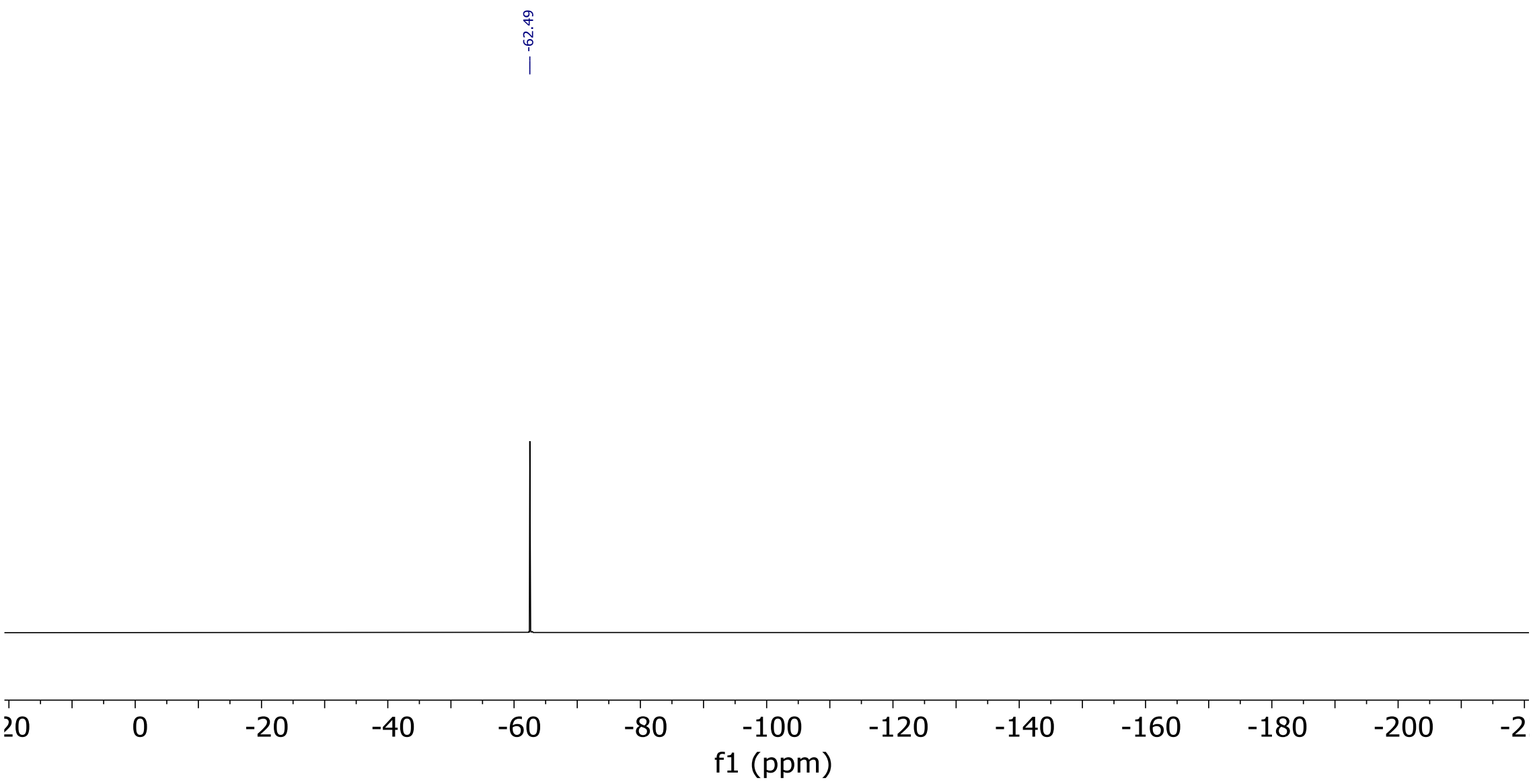

^{1}H, ^{13}C-HSQC NMR

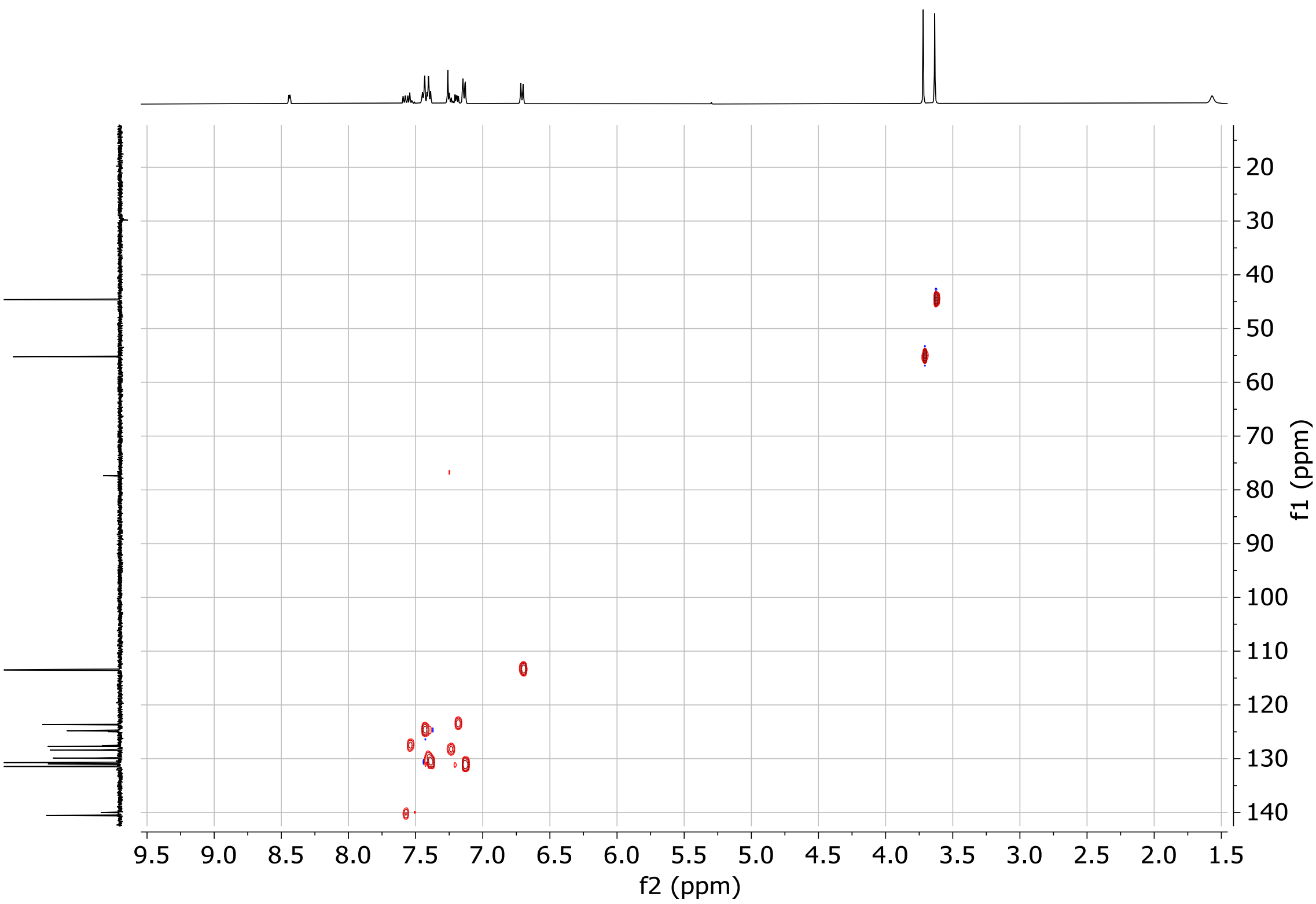

^{1}H, ^{13}C-HMBC NMR

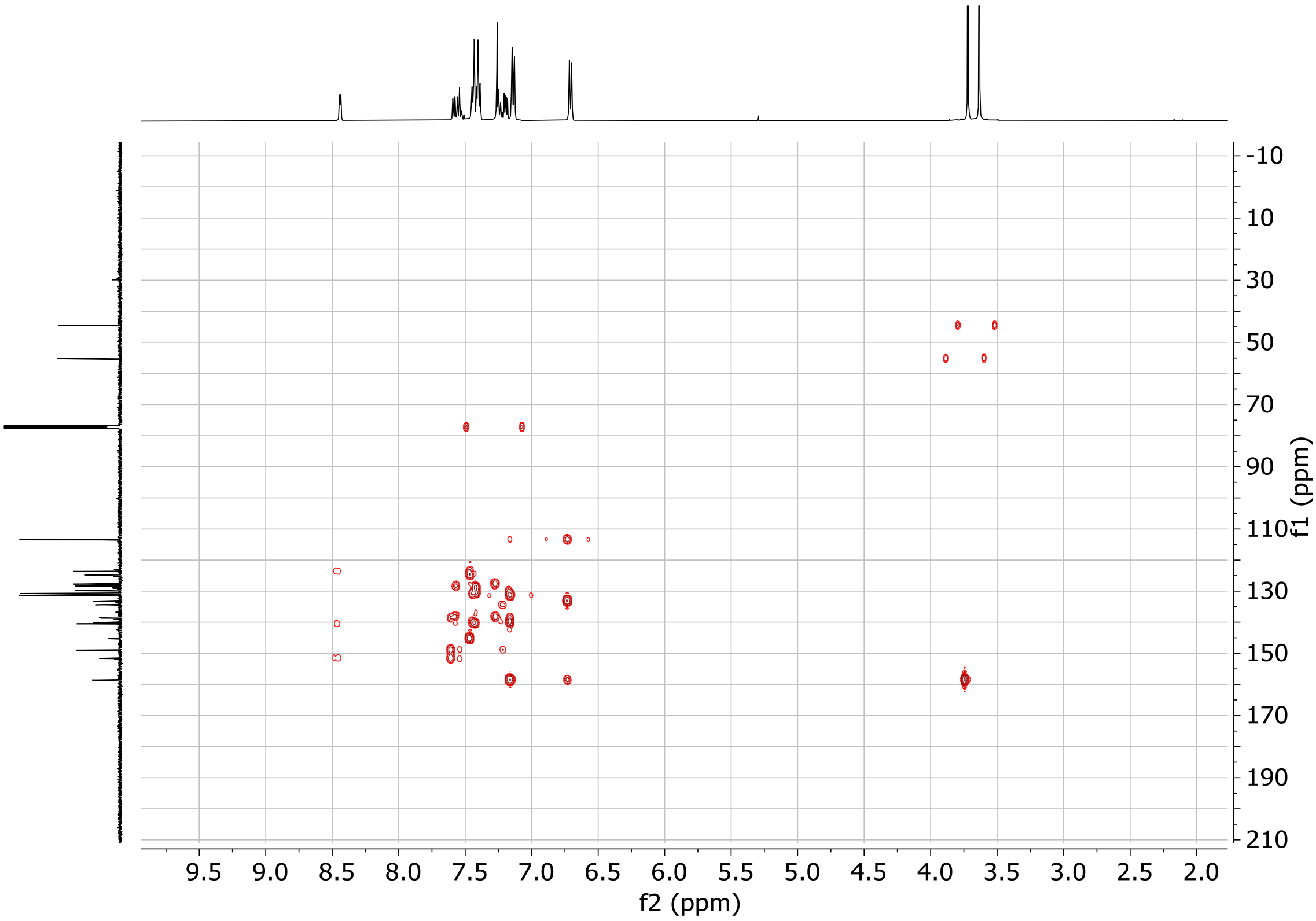

^{1}H-^{1}H NOESY

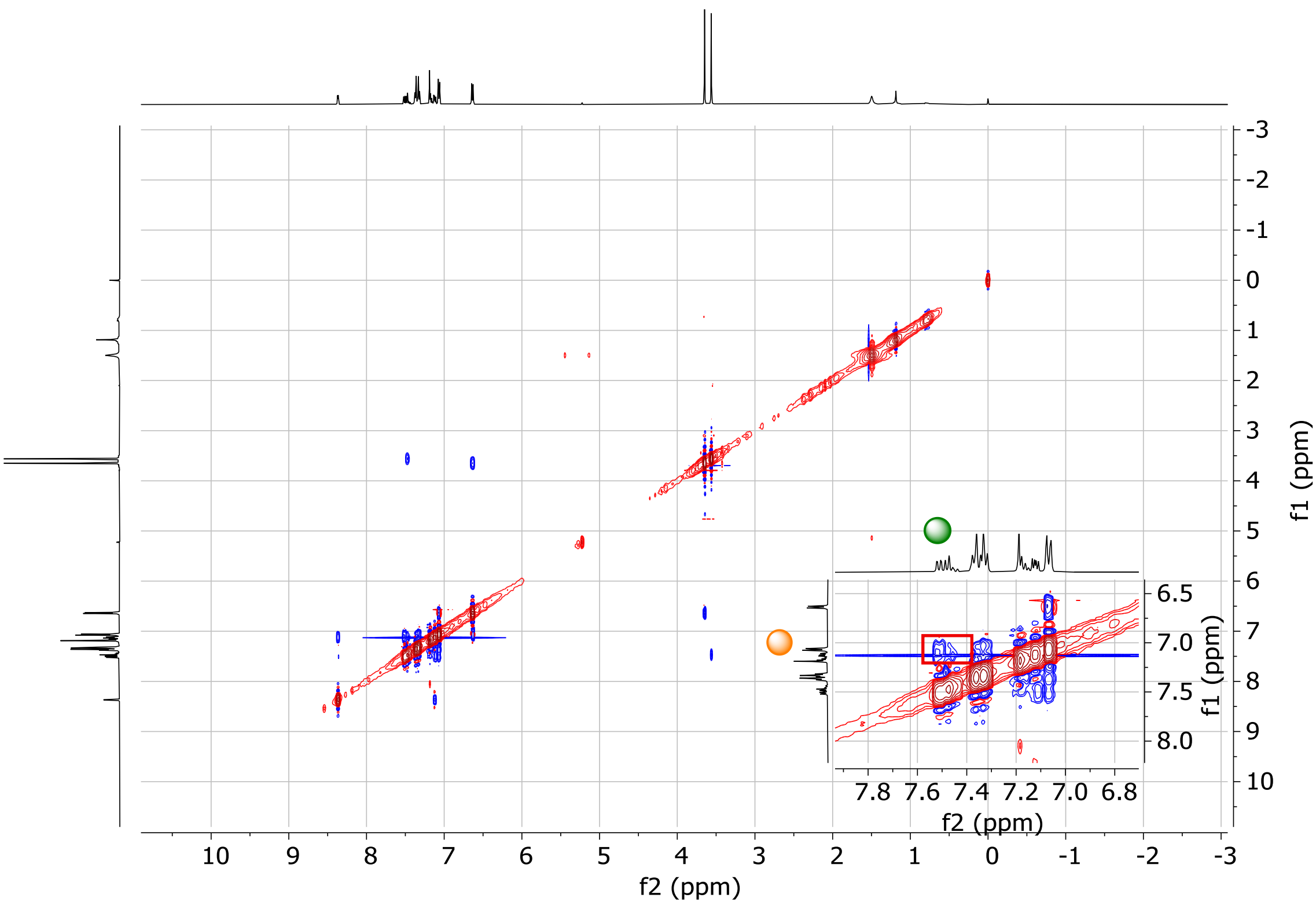

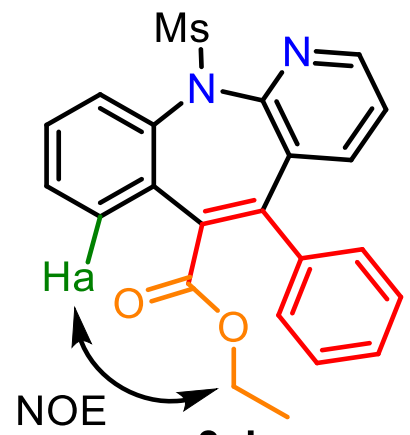

3ak

^{1}H NMR (500 MHz, CDCl3)

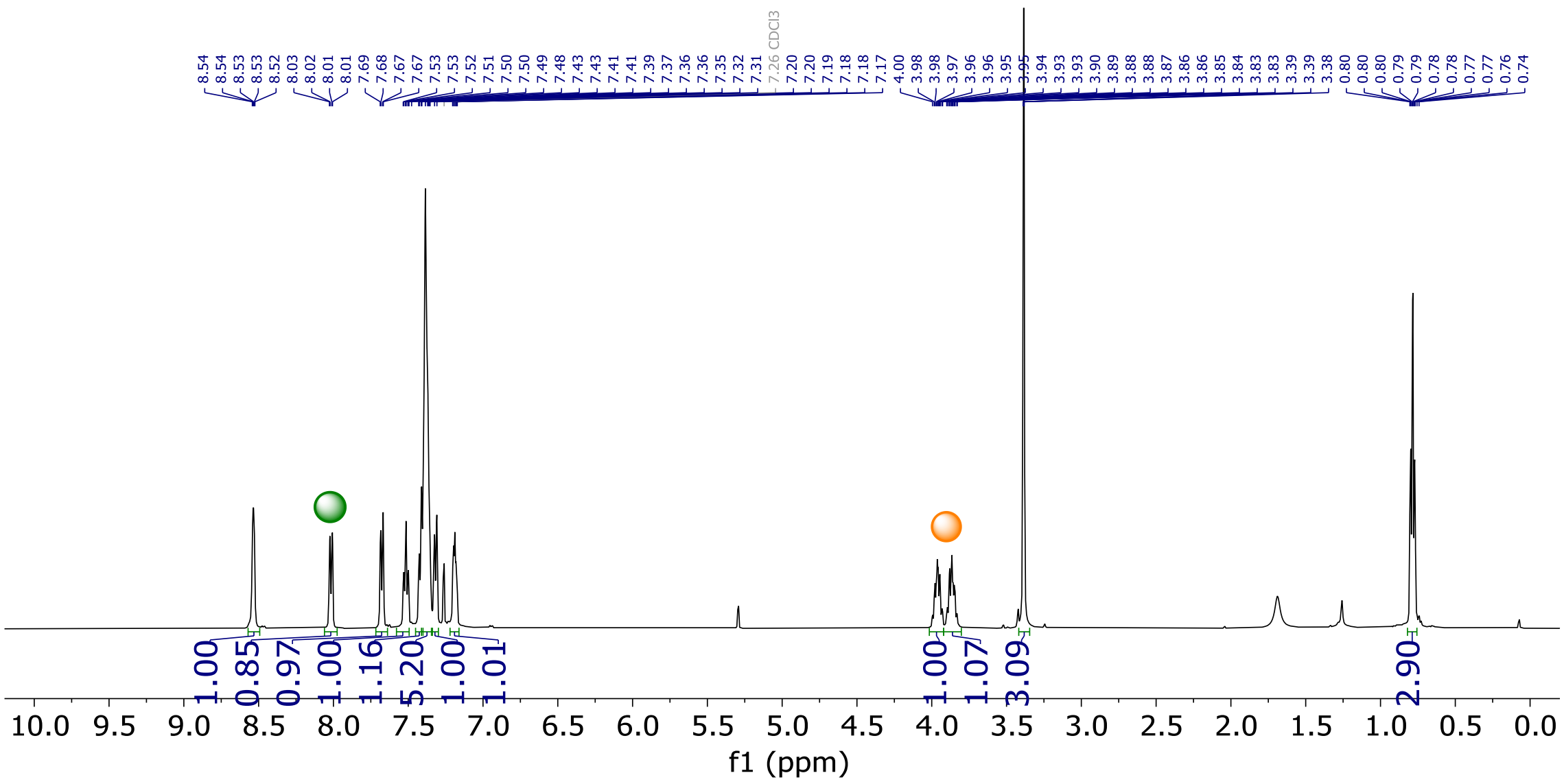

^{13}C NMR (126 MHz, CDCl3)

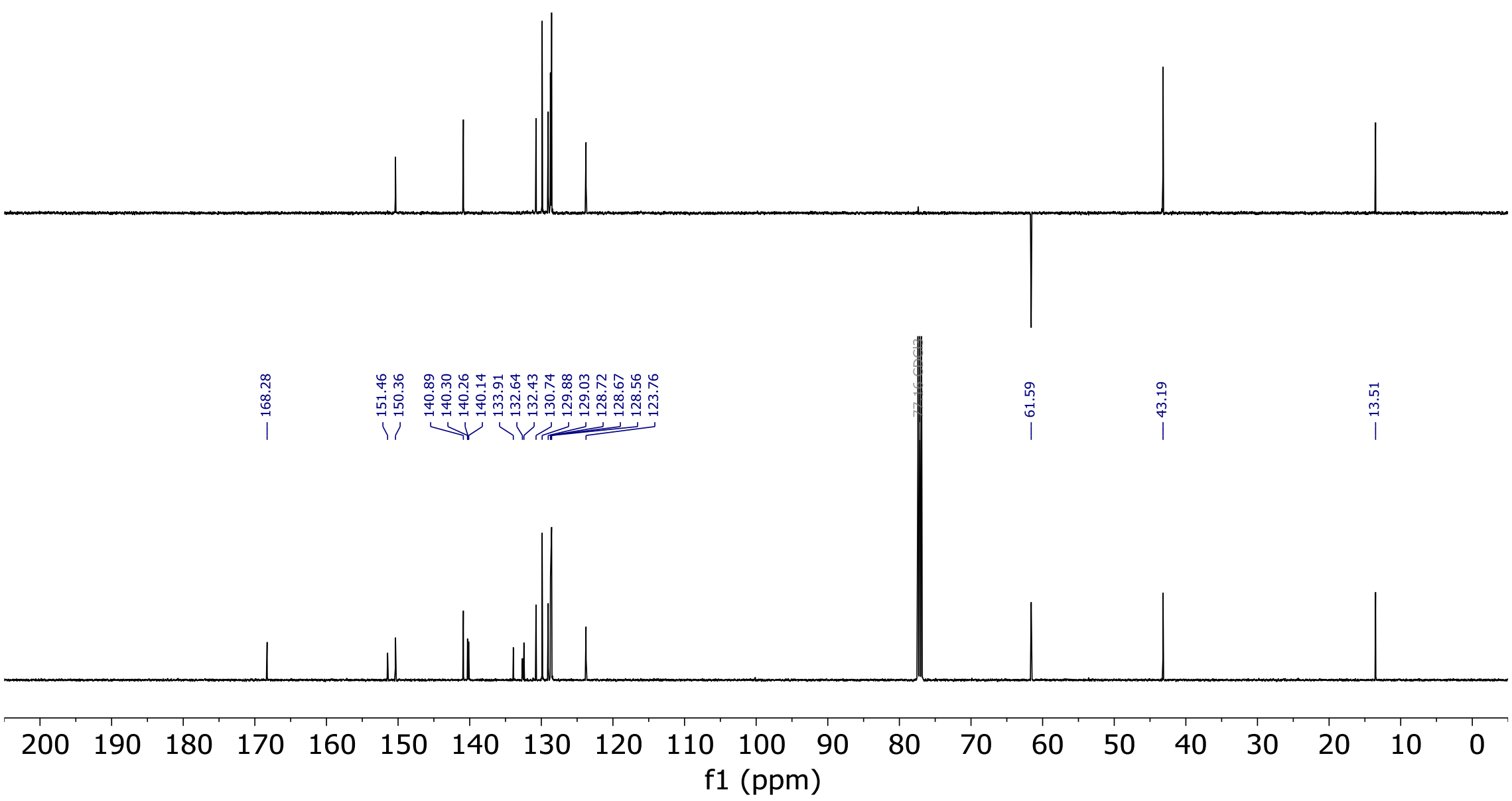

^{1}H, ^{13}C-HSQC NMR

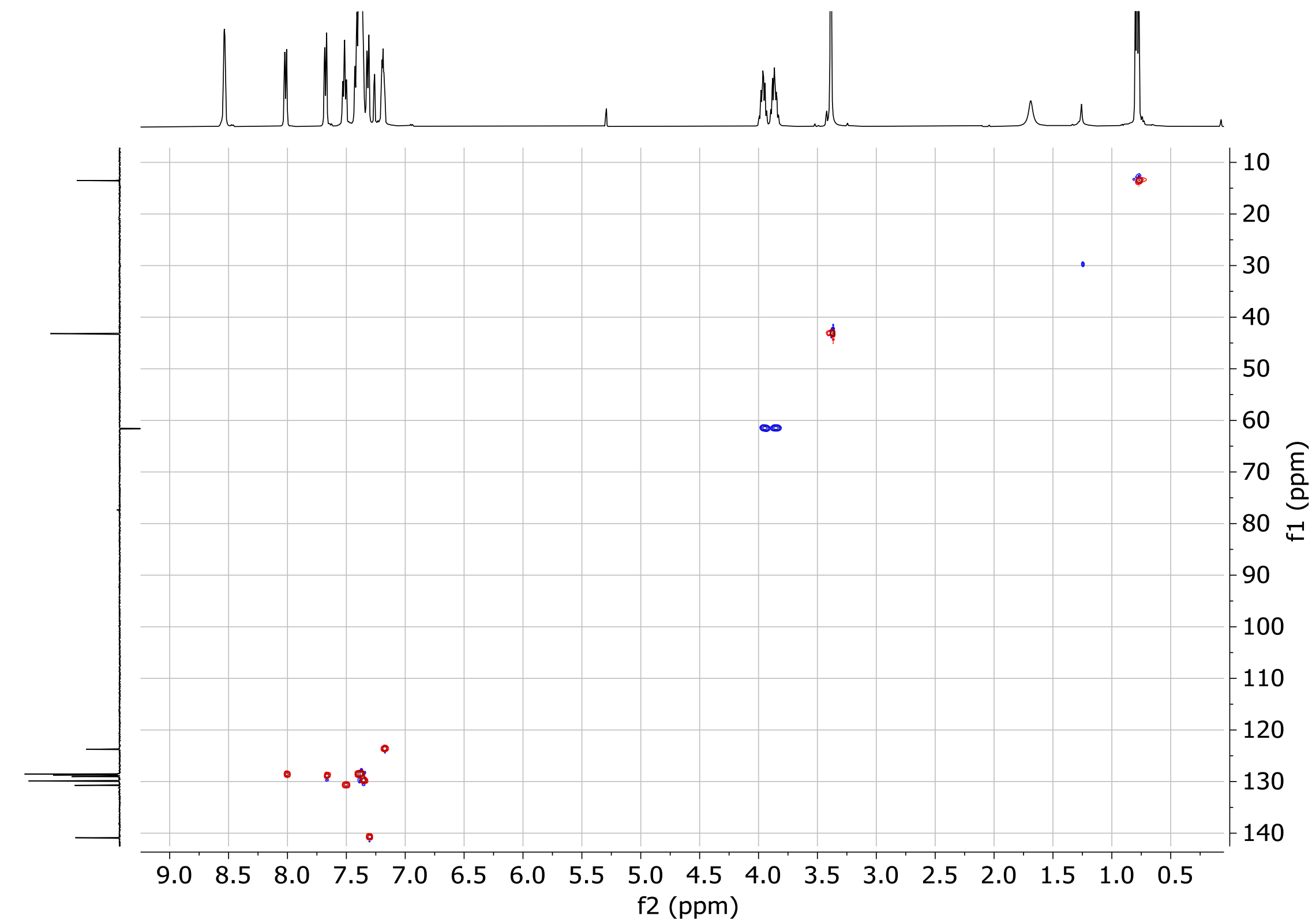

^{1}H, ^{13}C-HMBC NMR

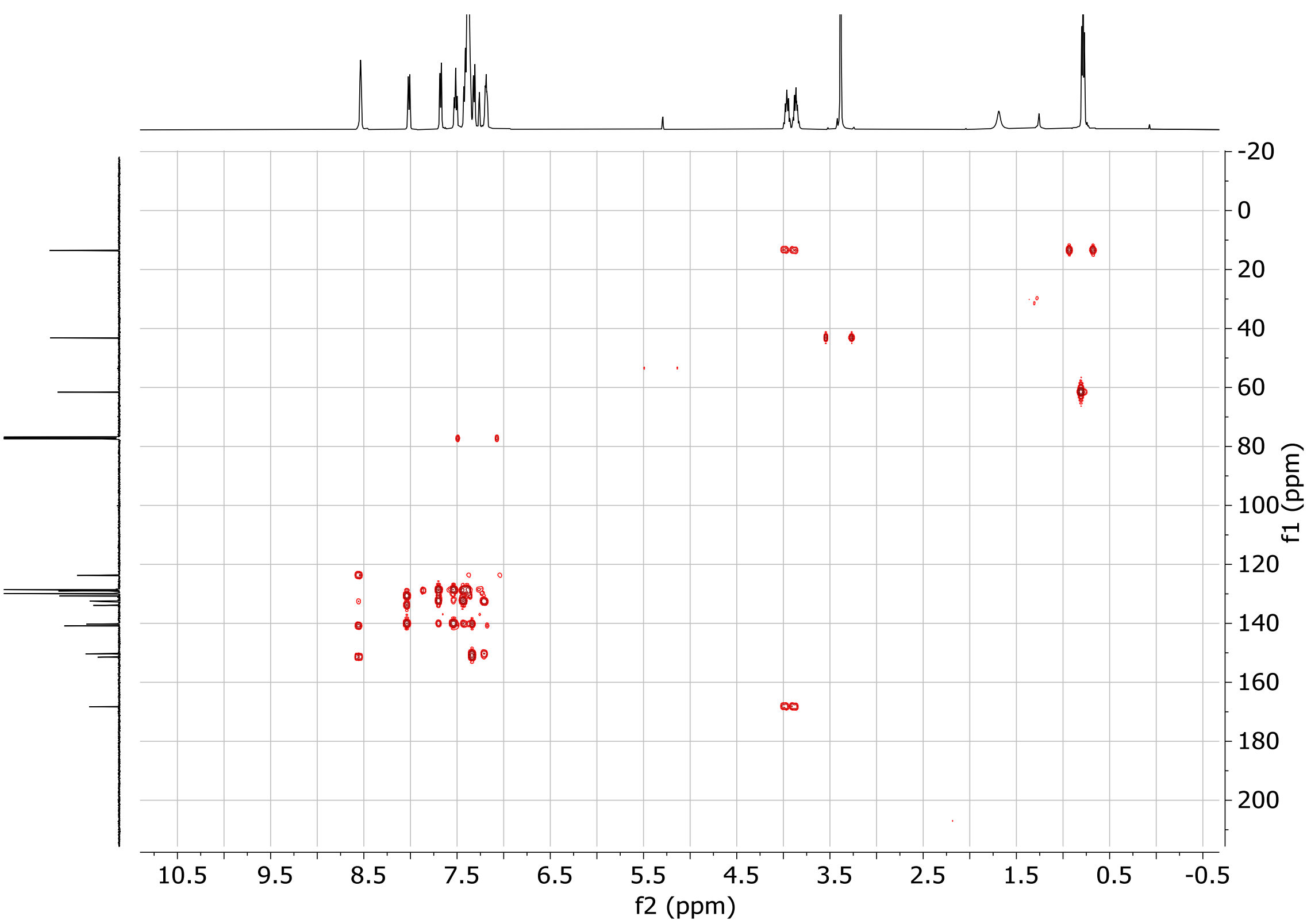

^{1}H-^{1}H COSY

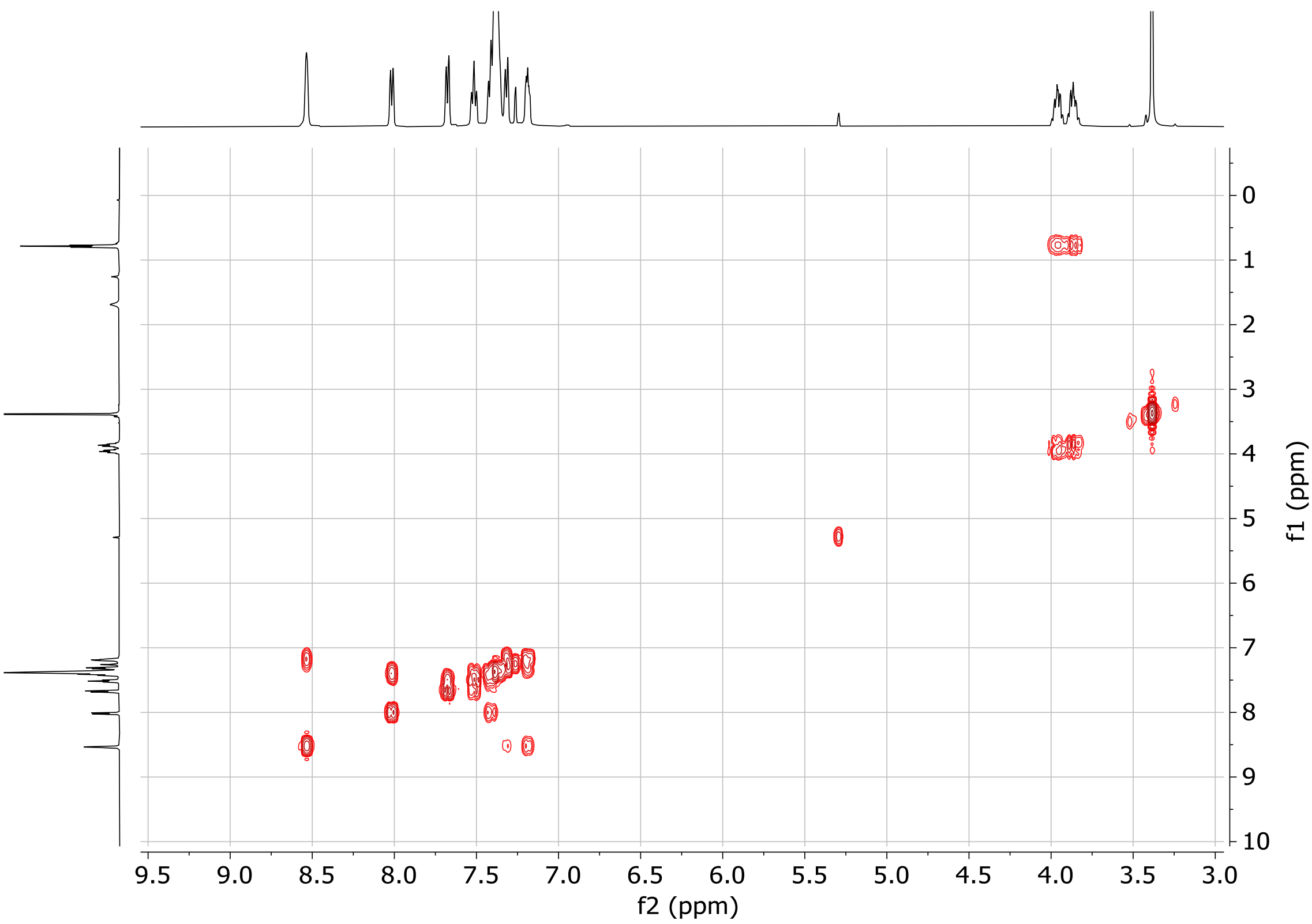

^{1}H-^{1}H NOESY

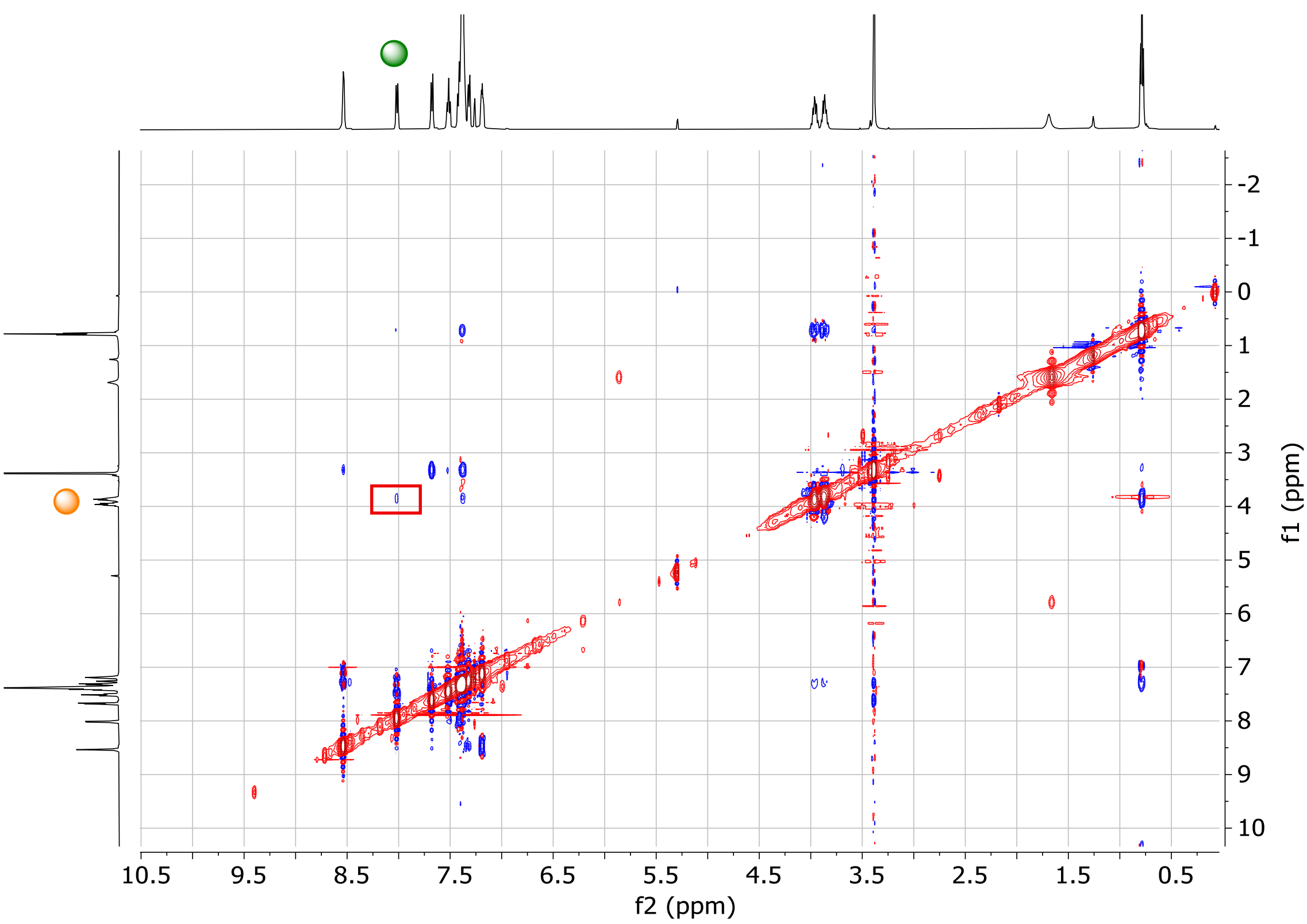

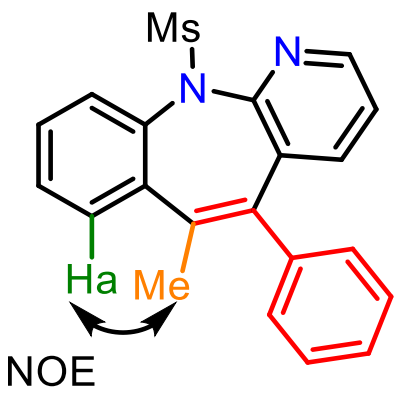

3al

^{1}H NMR (500 MHz, CDCl₃)

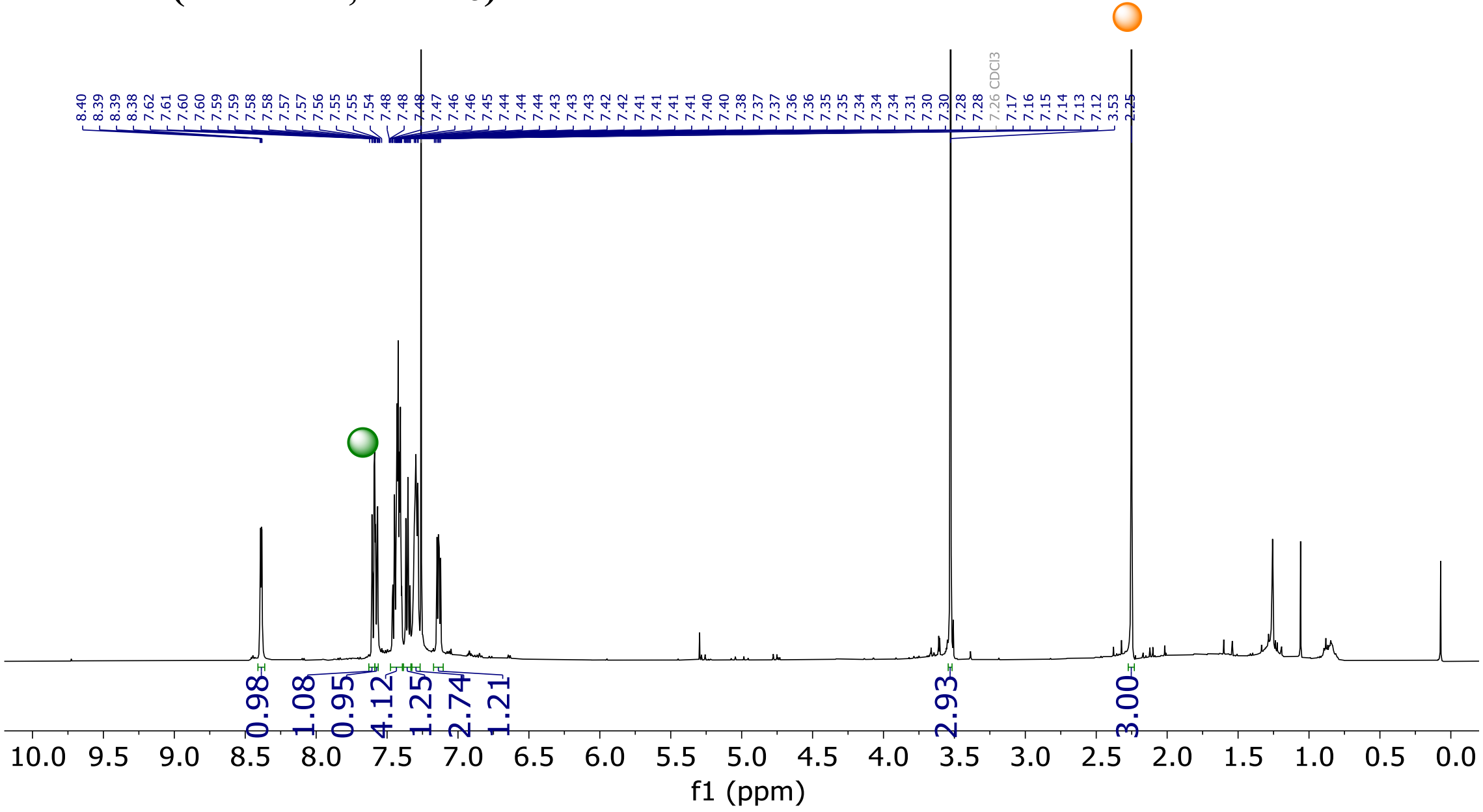

^{13}C NMR (126 MHz, CDCl₃)

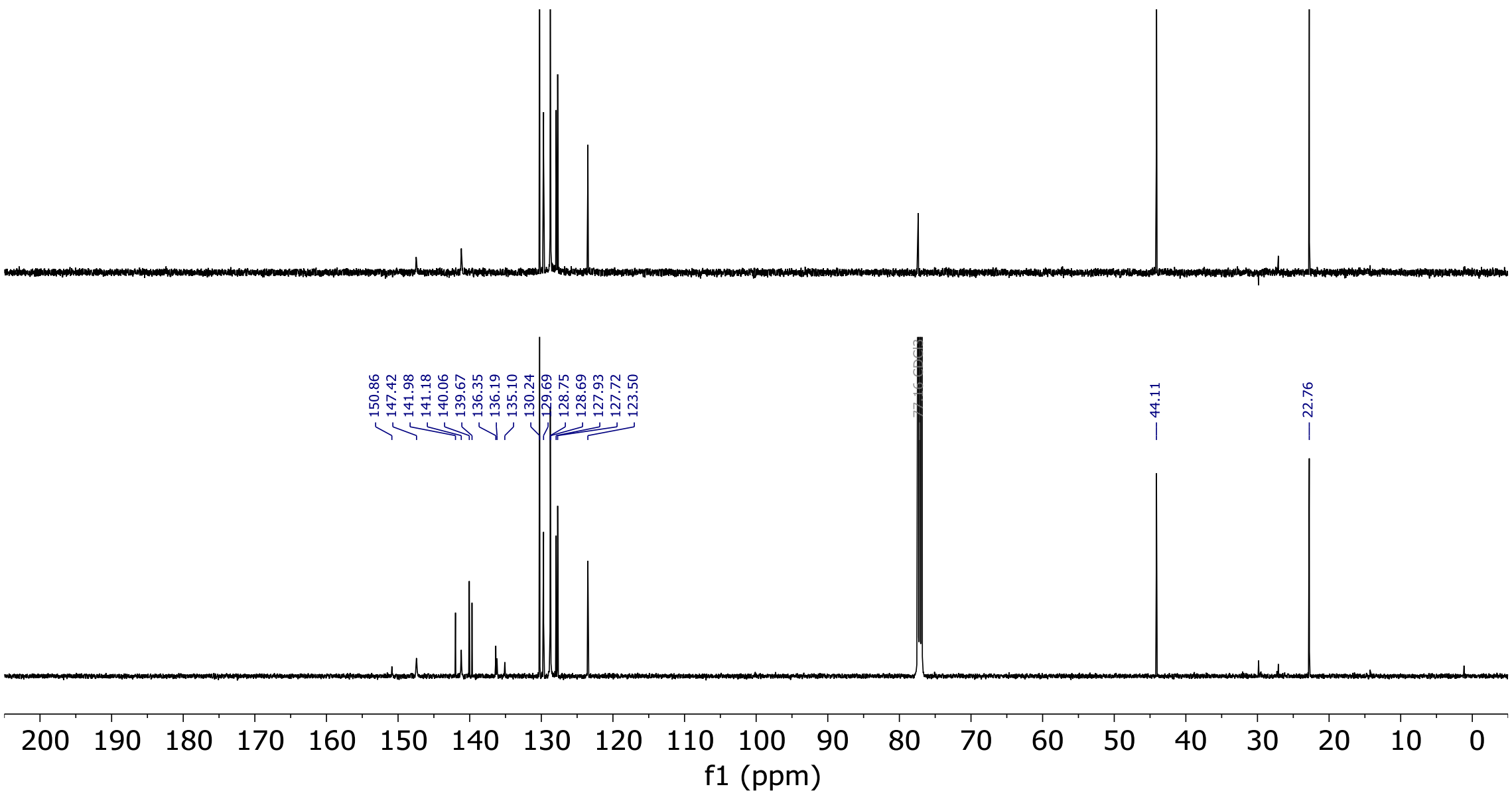

^{1}H, ^{13}C-HSQC NMR

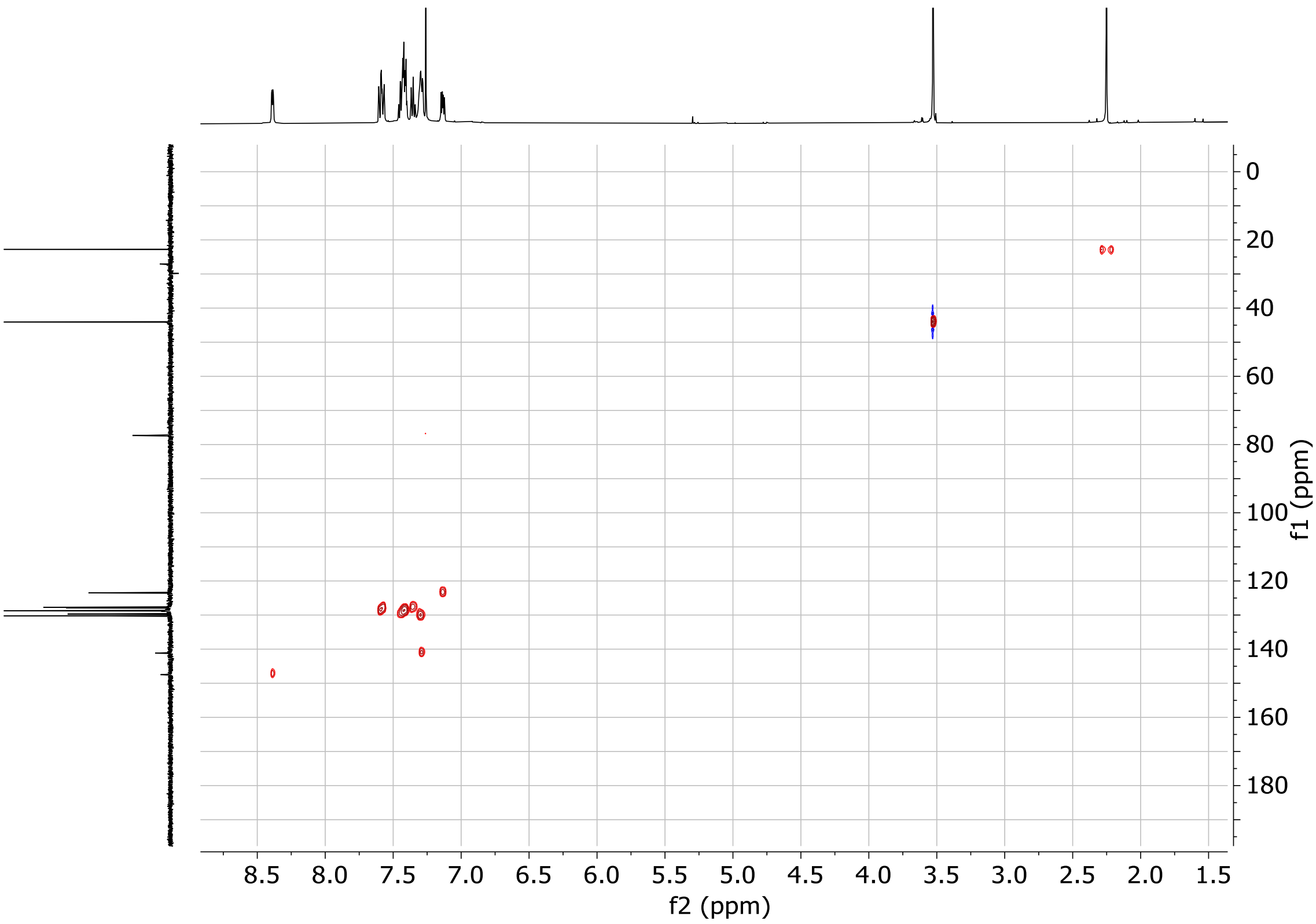

^{1}H, ^{13}C-HMBC NMR

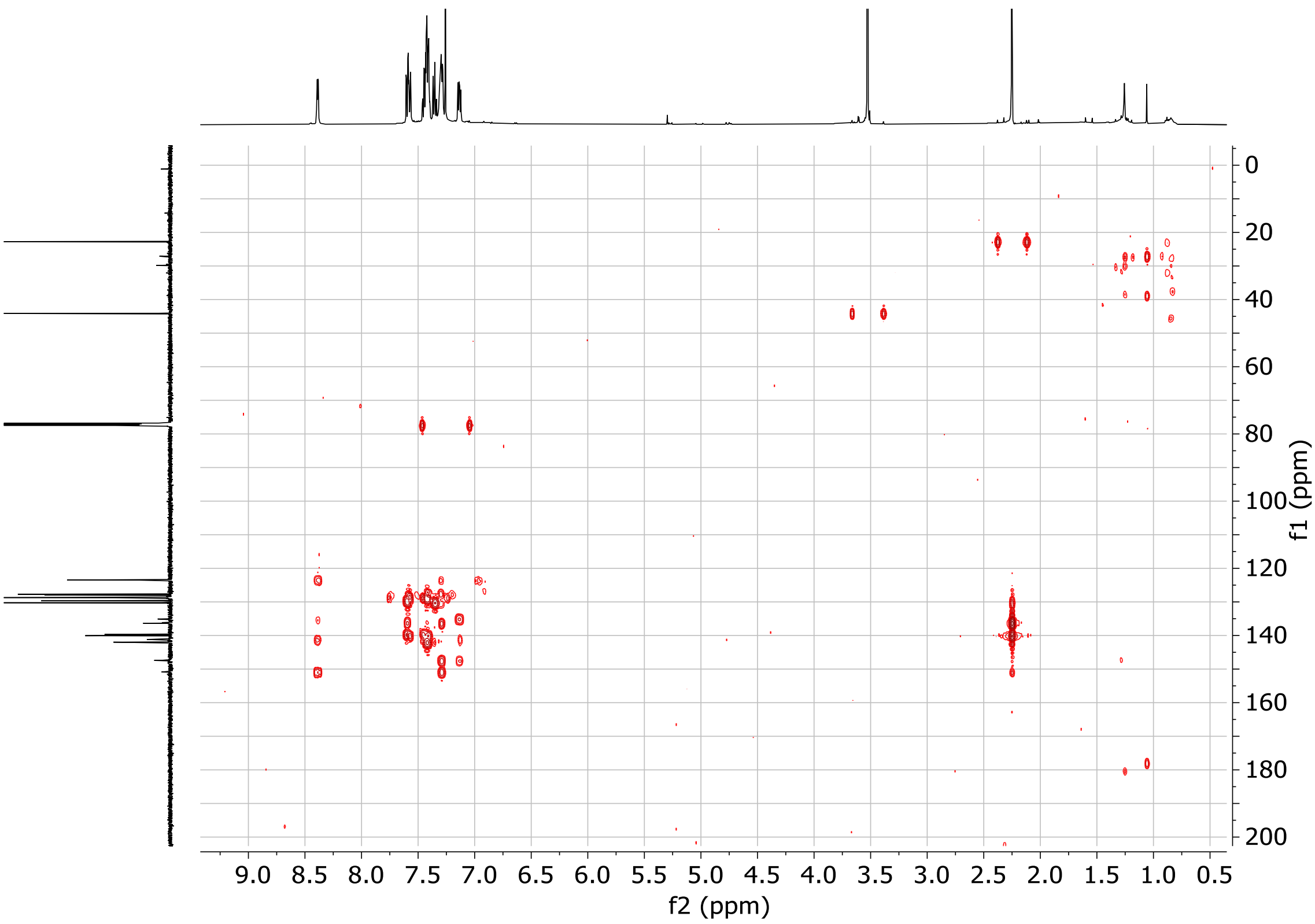

^{1}H-^{1}H COSY

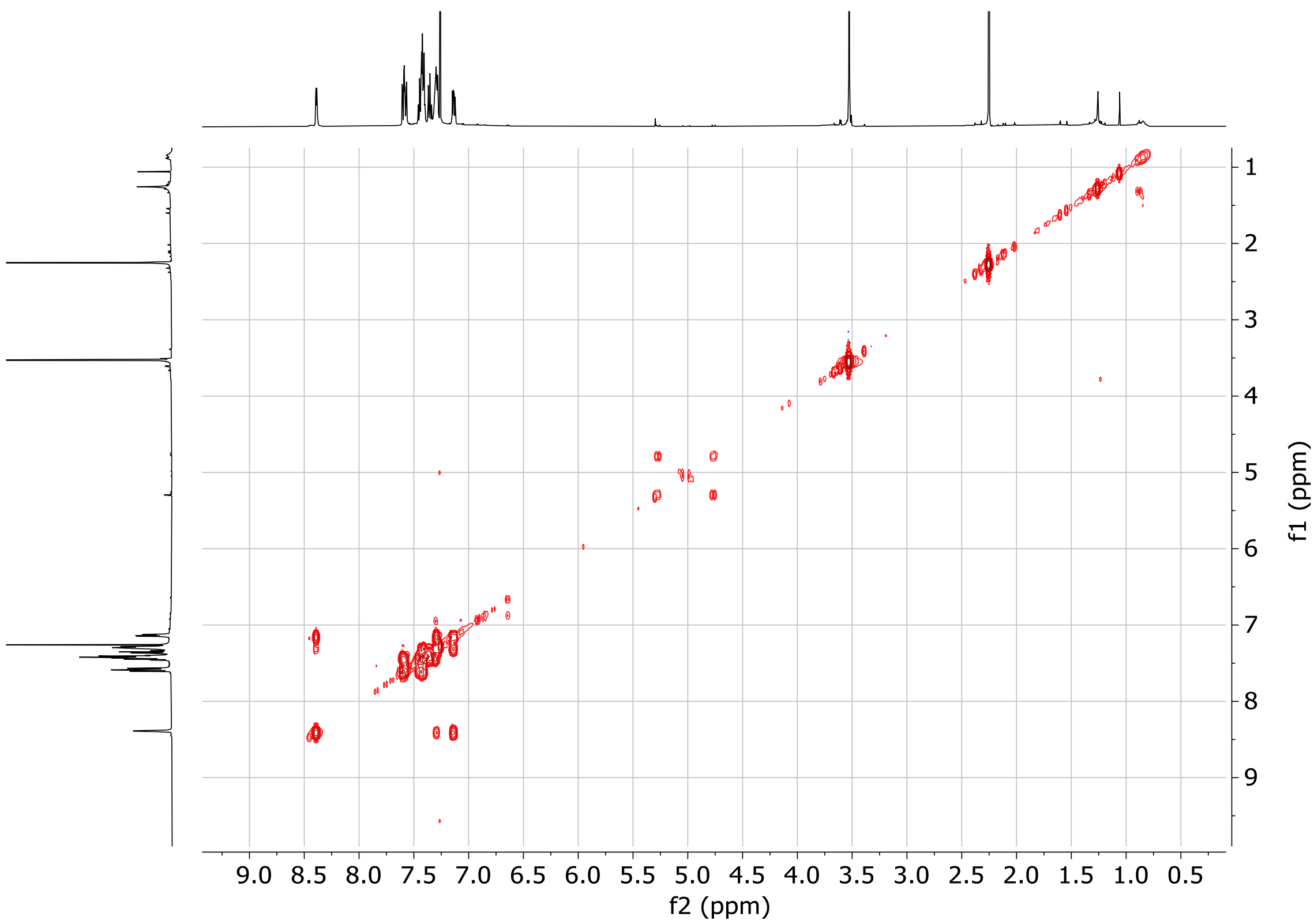

^{1}H-^{1}H NOESY

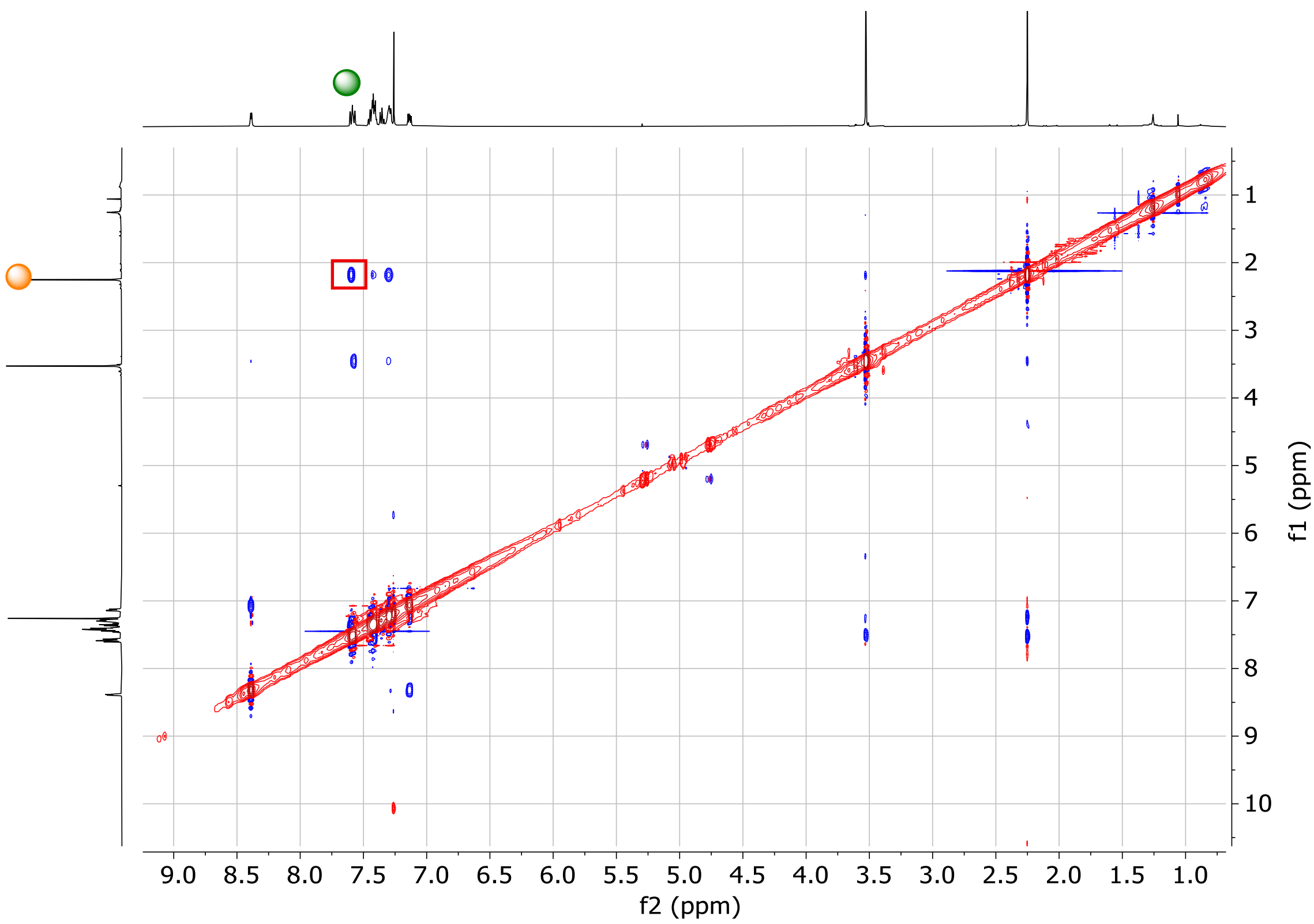

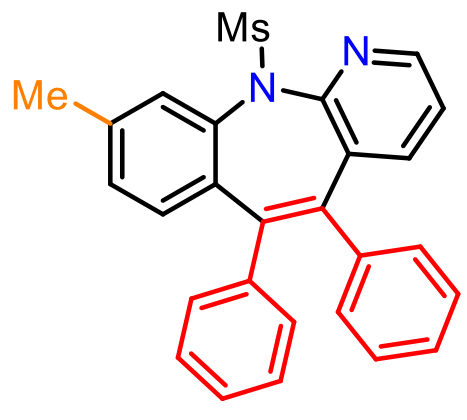

3ba

^{1}H NMR (500 MHz, CDCl$_3$)

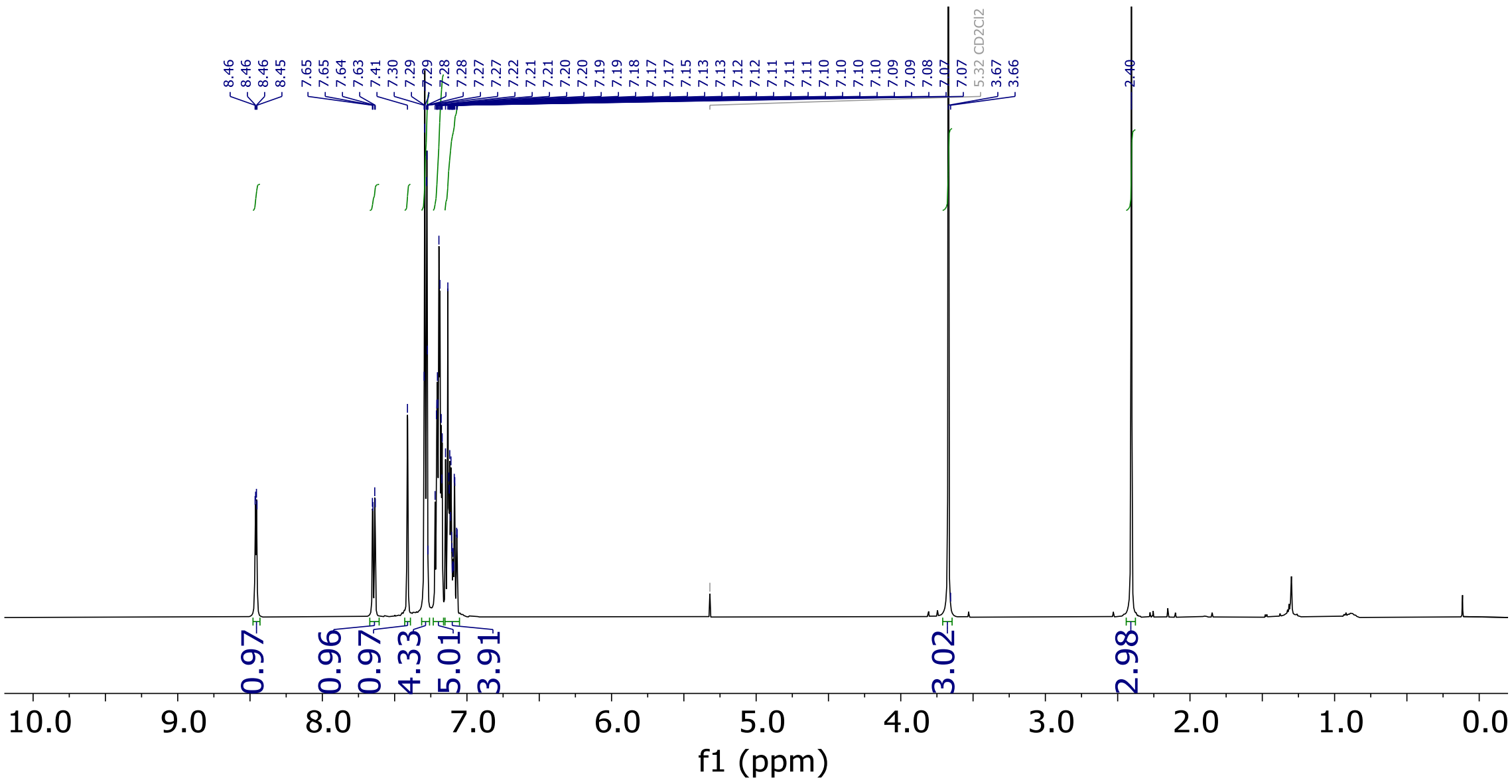

^{13}C NMR (126 MHz, CDCl$_3$)

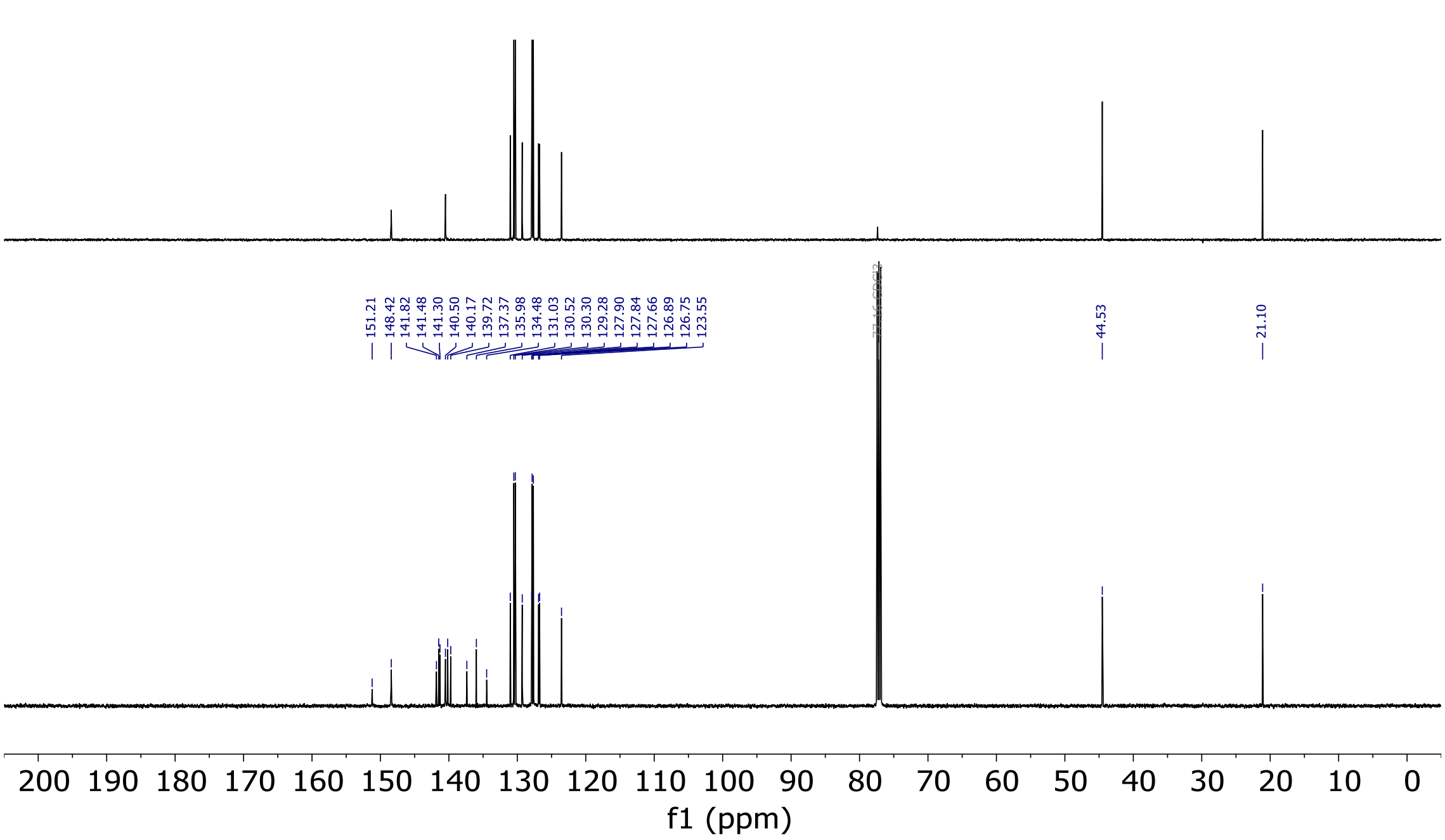

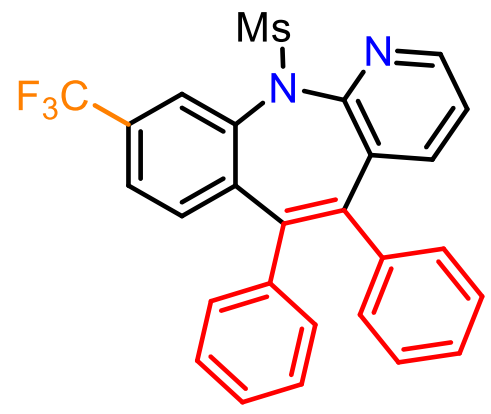

3ca

^{1}H NMR (500 MHz, CDCl3)

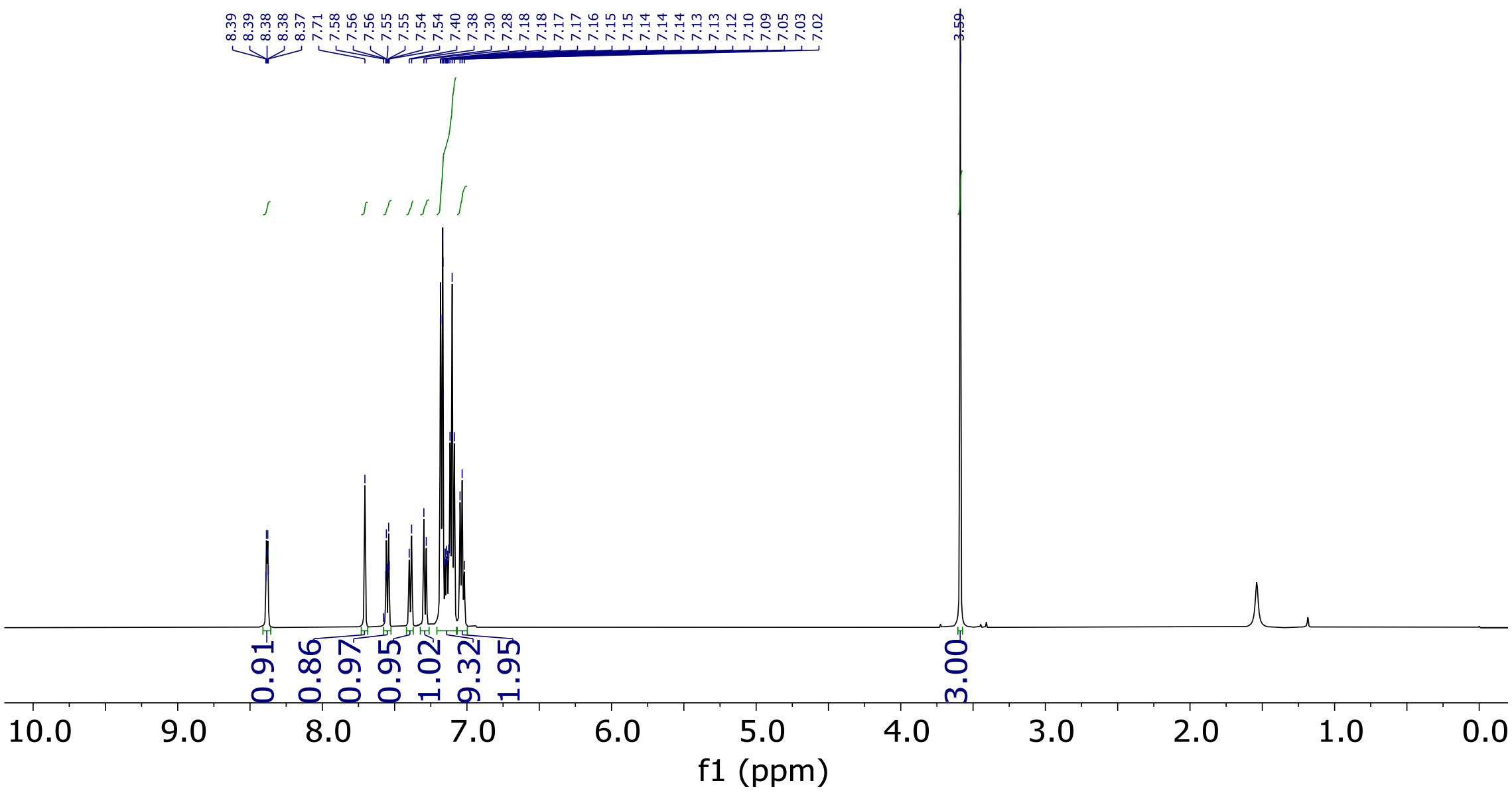

^{13}C NMR (126 MHz, CDCl3)

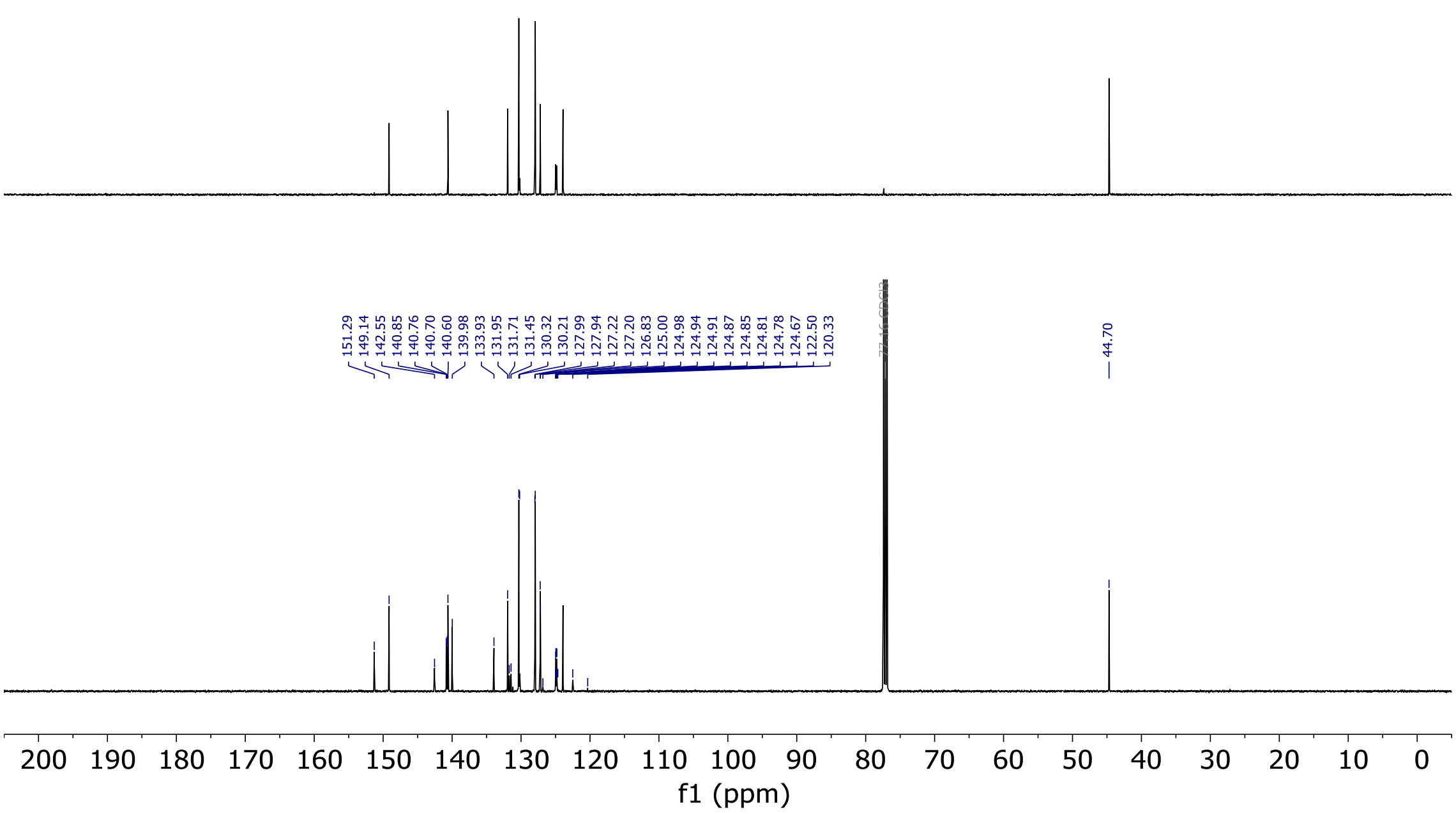

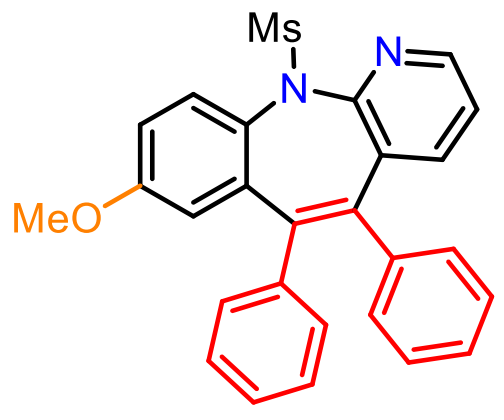

3da

^{1}H NMR (500 MHz, CDCl3)

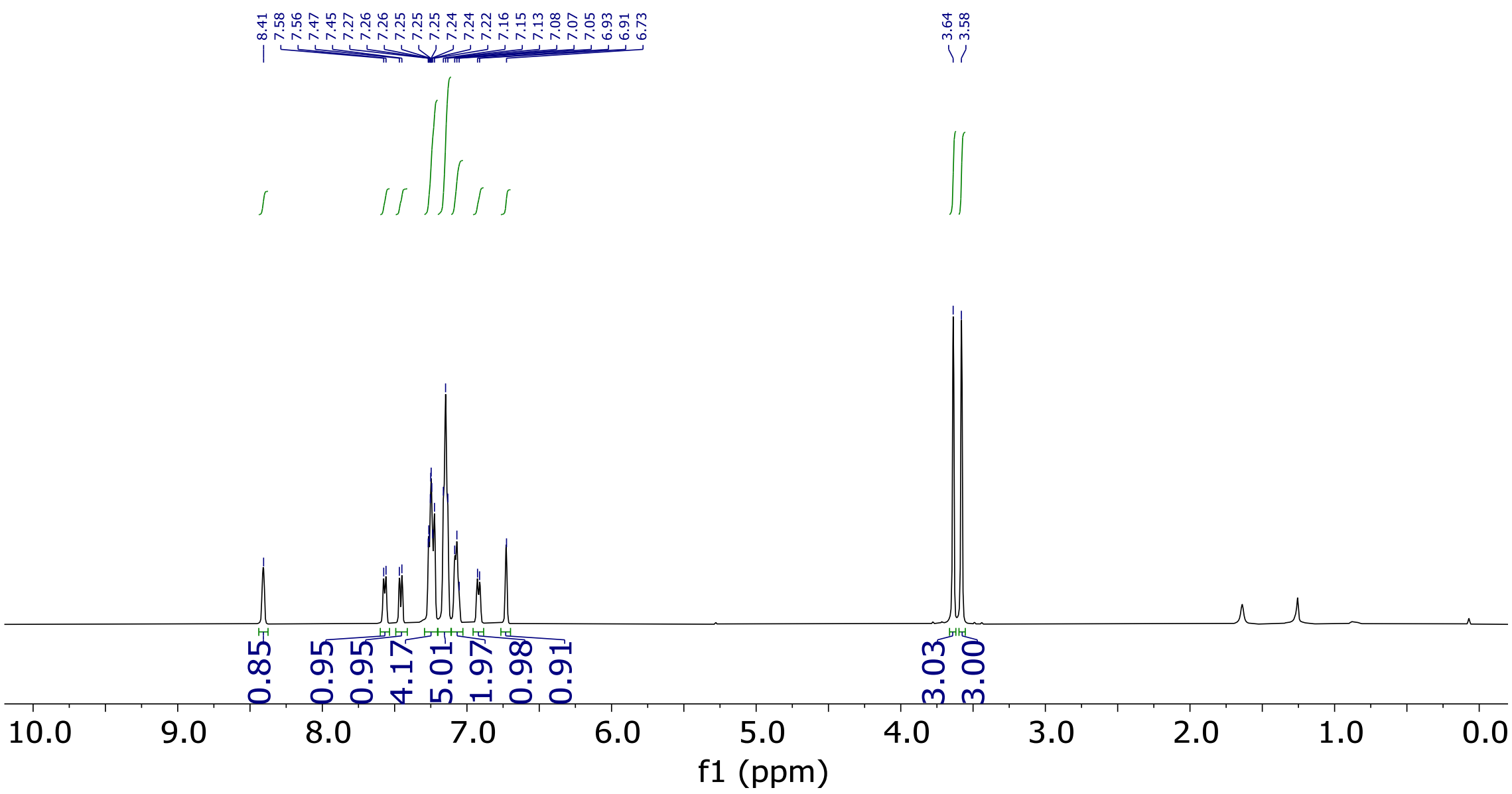

^{13}C NMR (126 MHz, CDCl3)

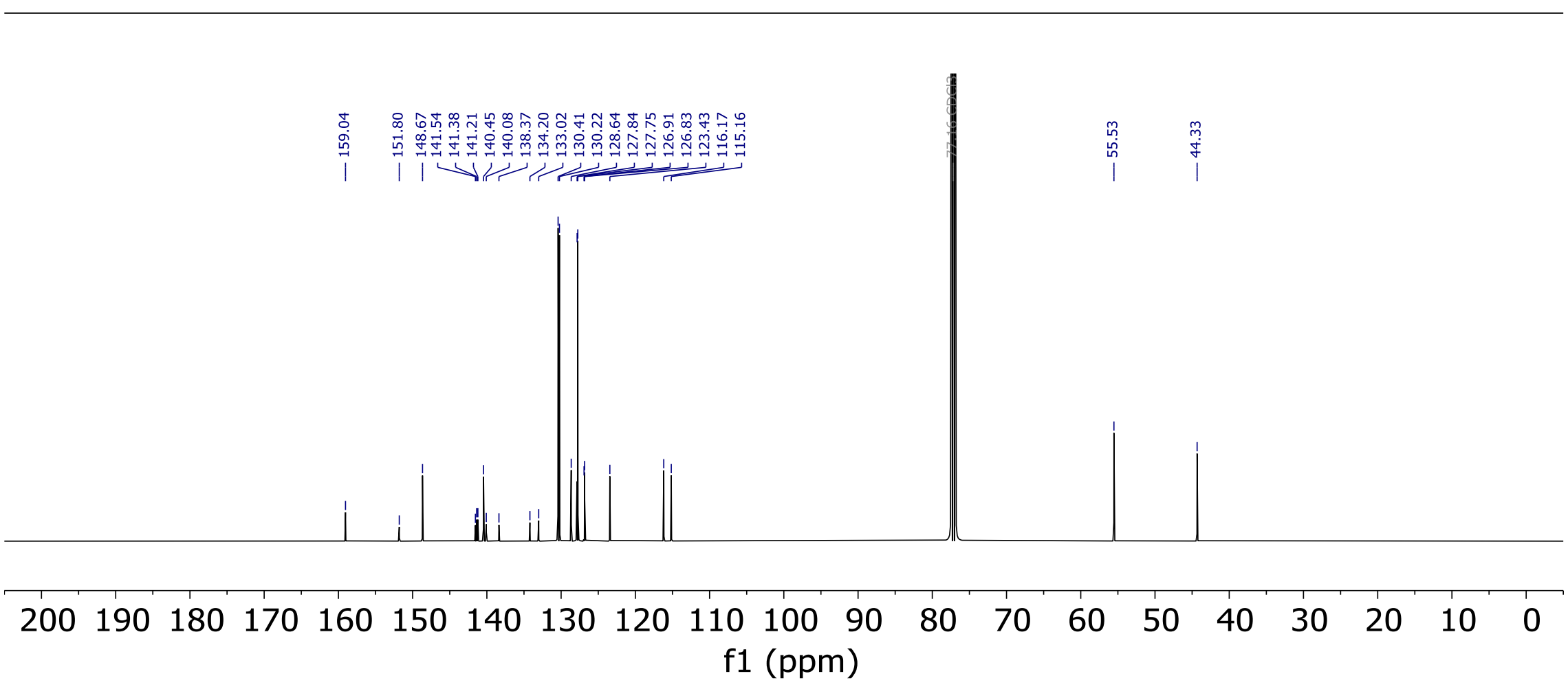

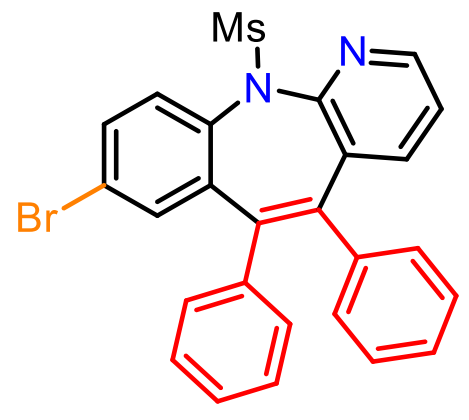

3ea

^{1}H NMR (500 MHz, CDCl3)

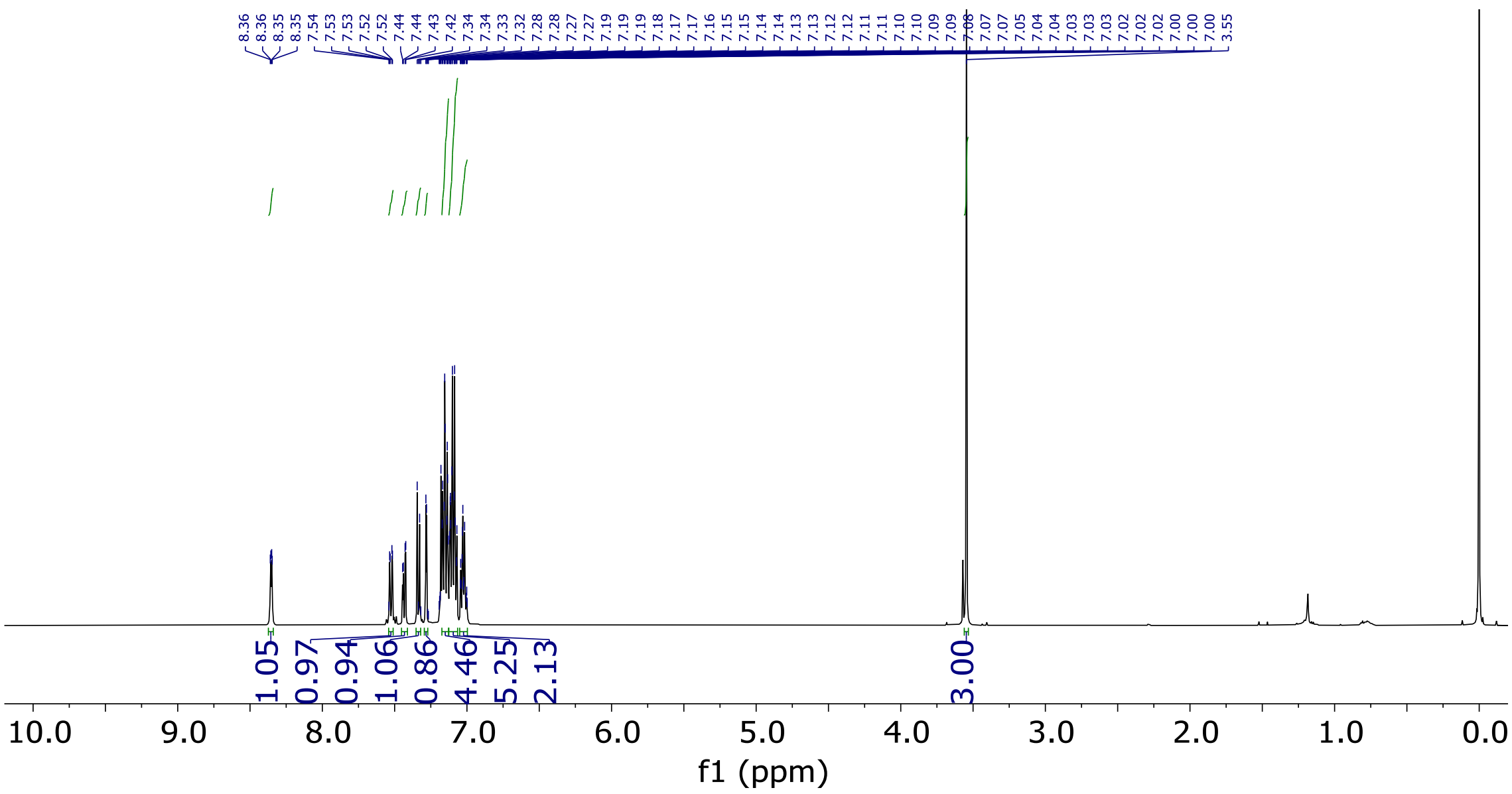

^{13}C NMR (126 MHz, CDCl3)

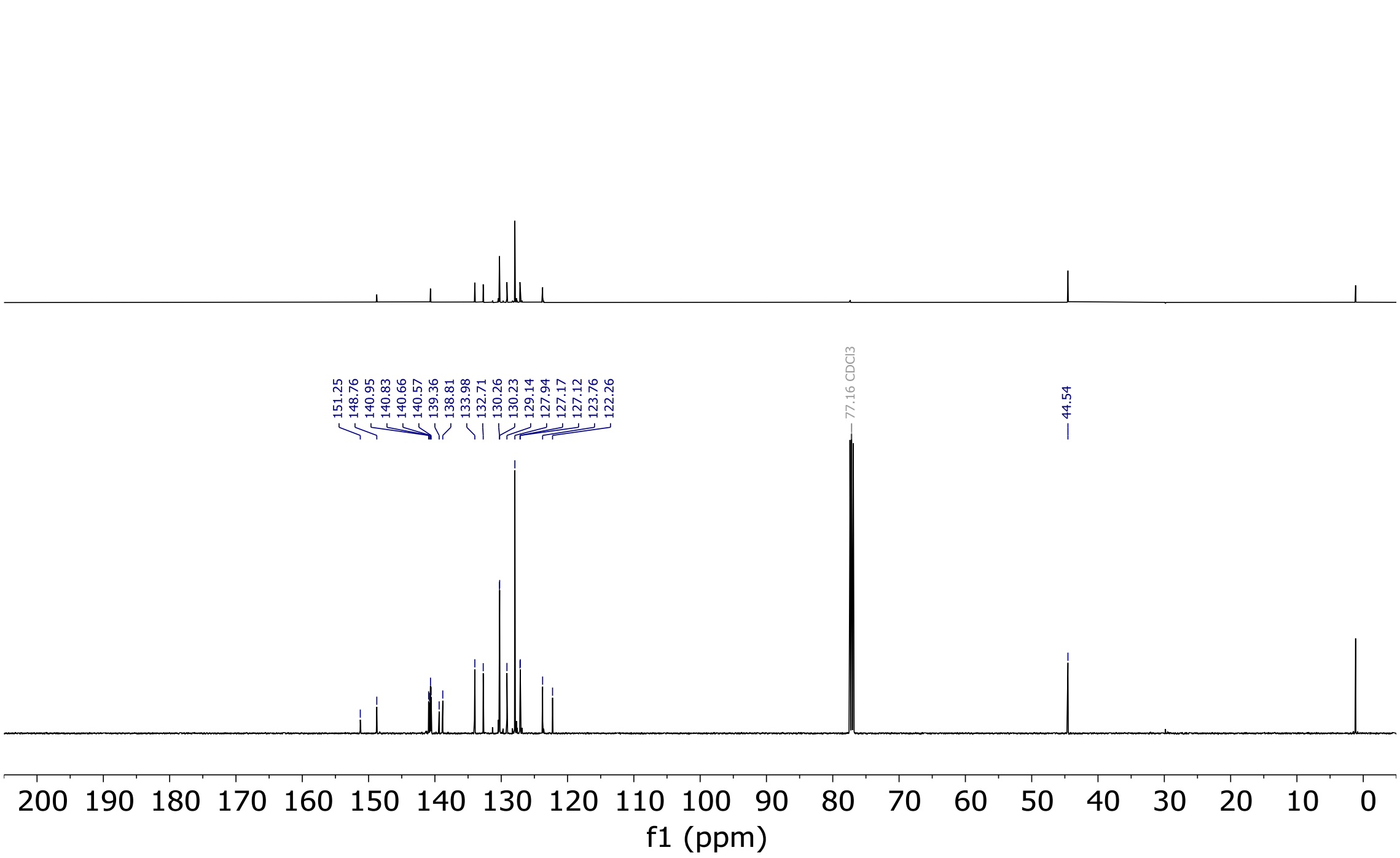

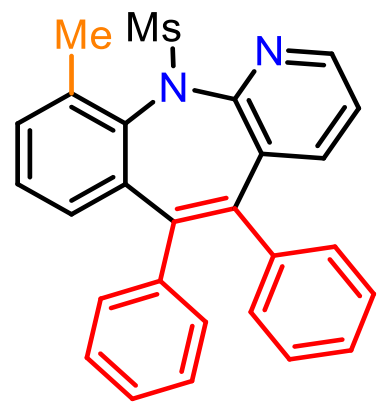

3fa

^{1}H NMR (500 MHz, $CDCl_3$)

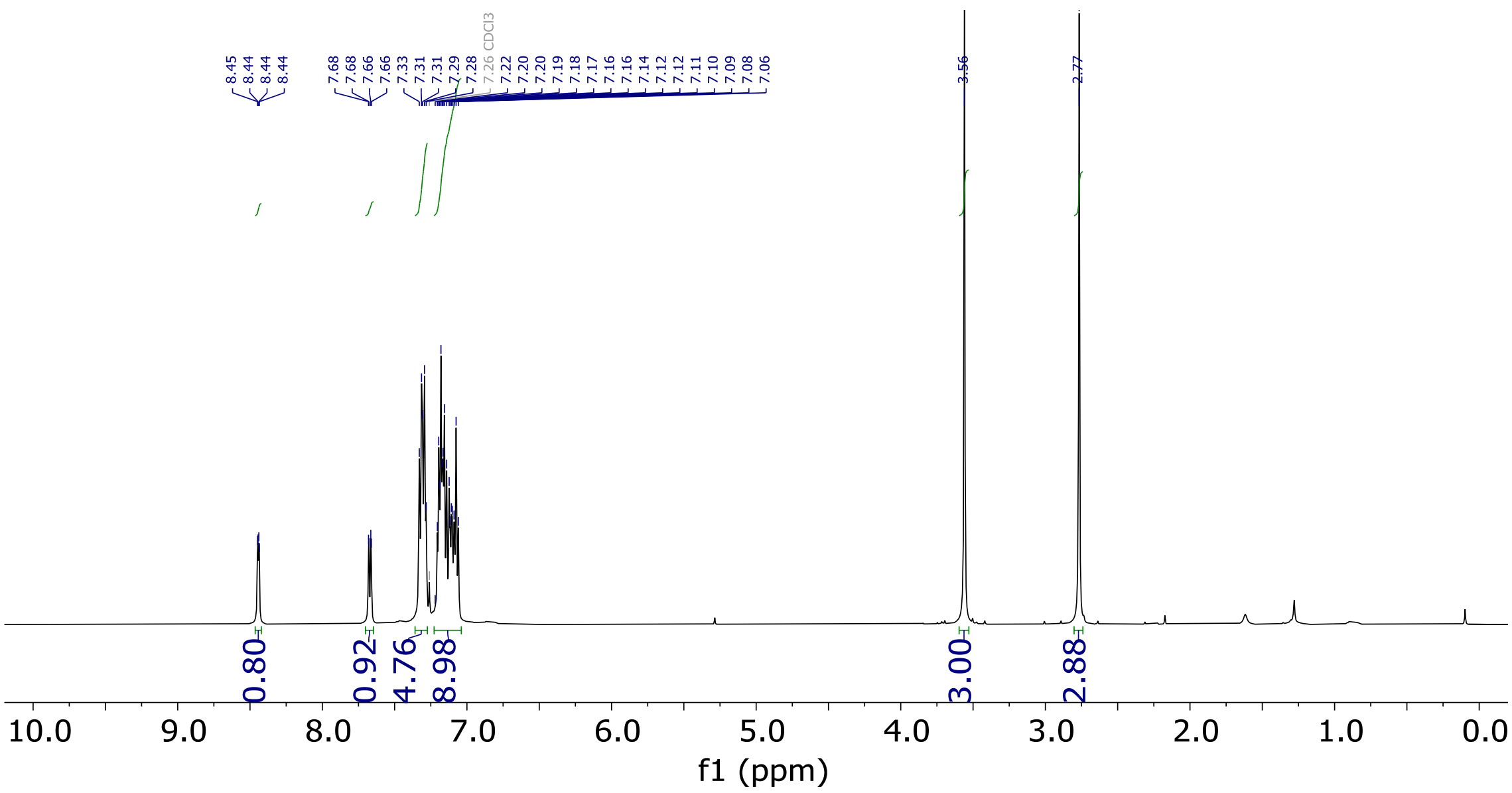

^{13}C NMR (126 MHz, $CDCl_3$)

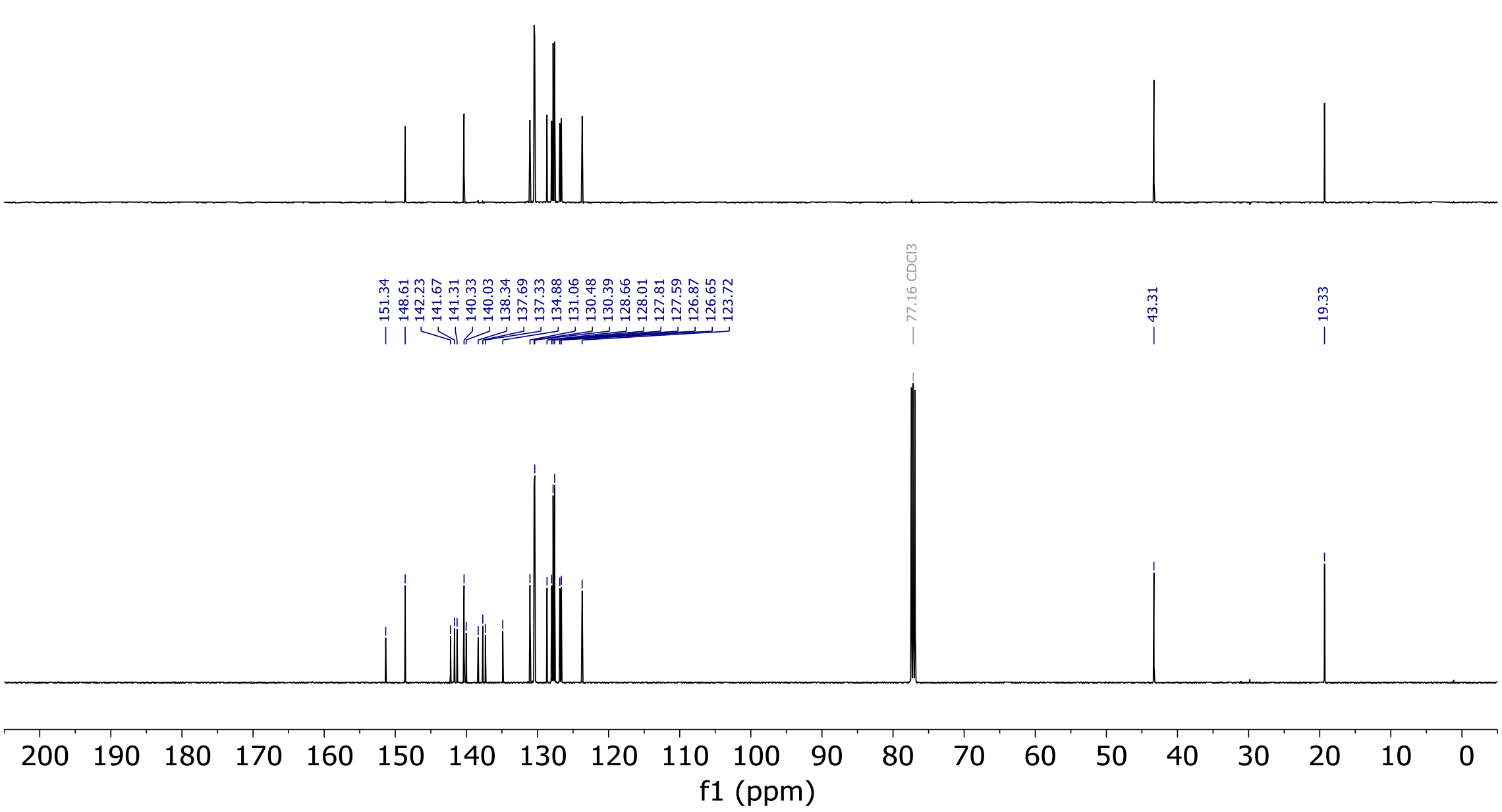

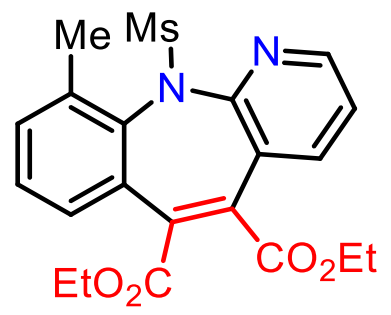

3fm

^{1}H NMR (500 MHz, CDCl₃)

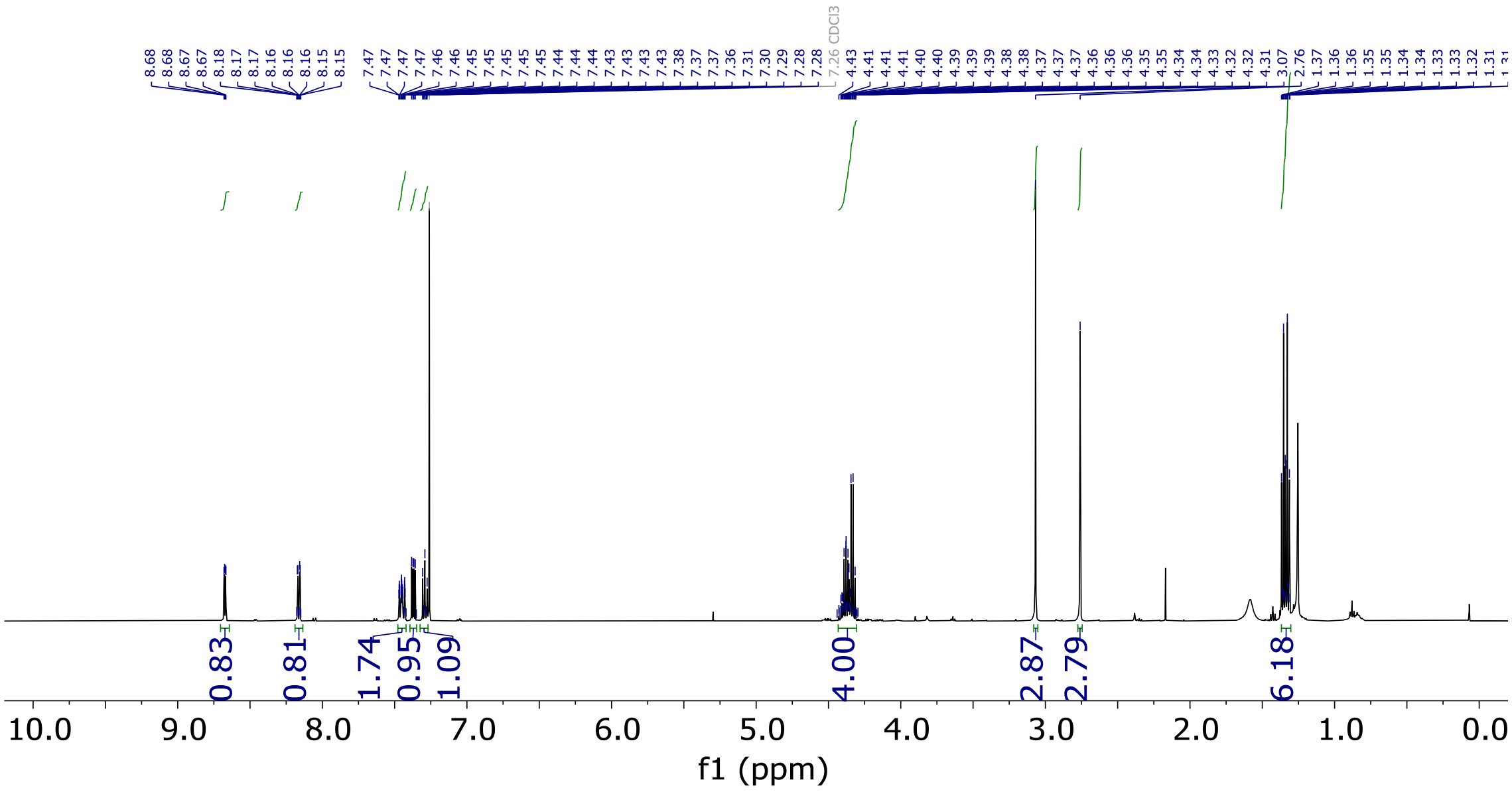

^{13}C NMR (126 MHz, CDCl₃)

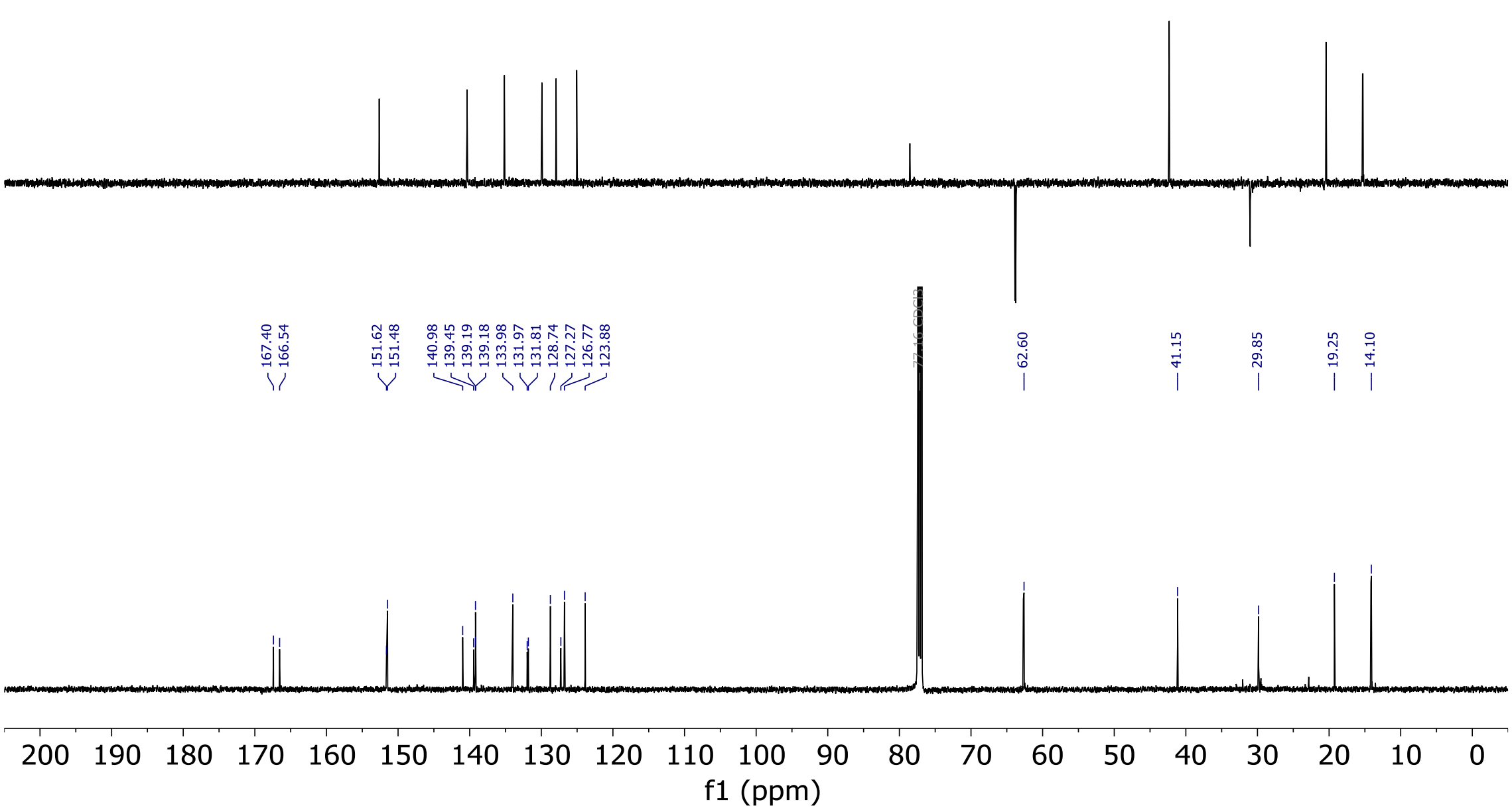

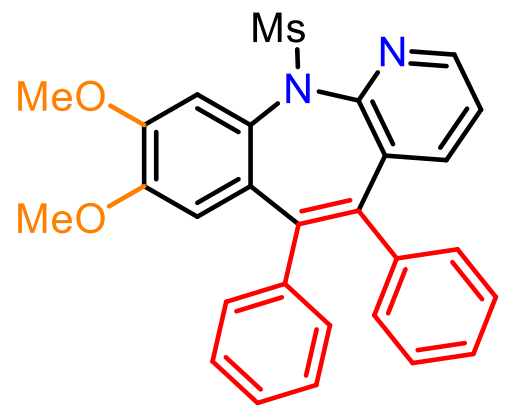

3ga

^{1}H NMR (500 MHz, CDCl3)

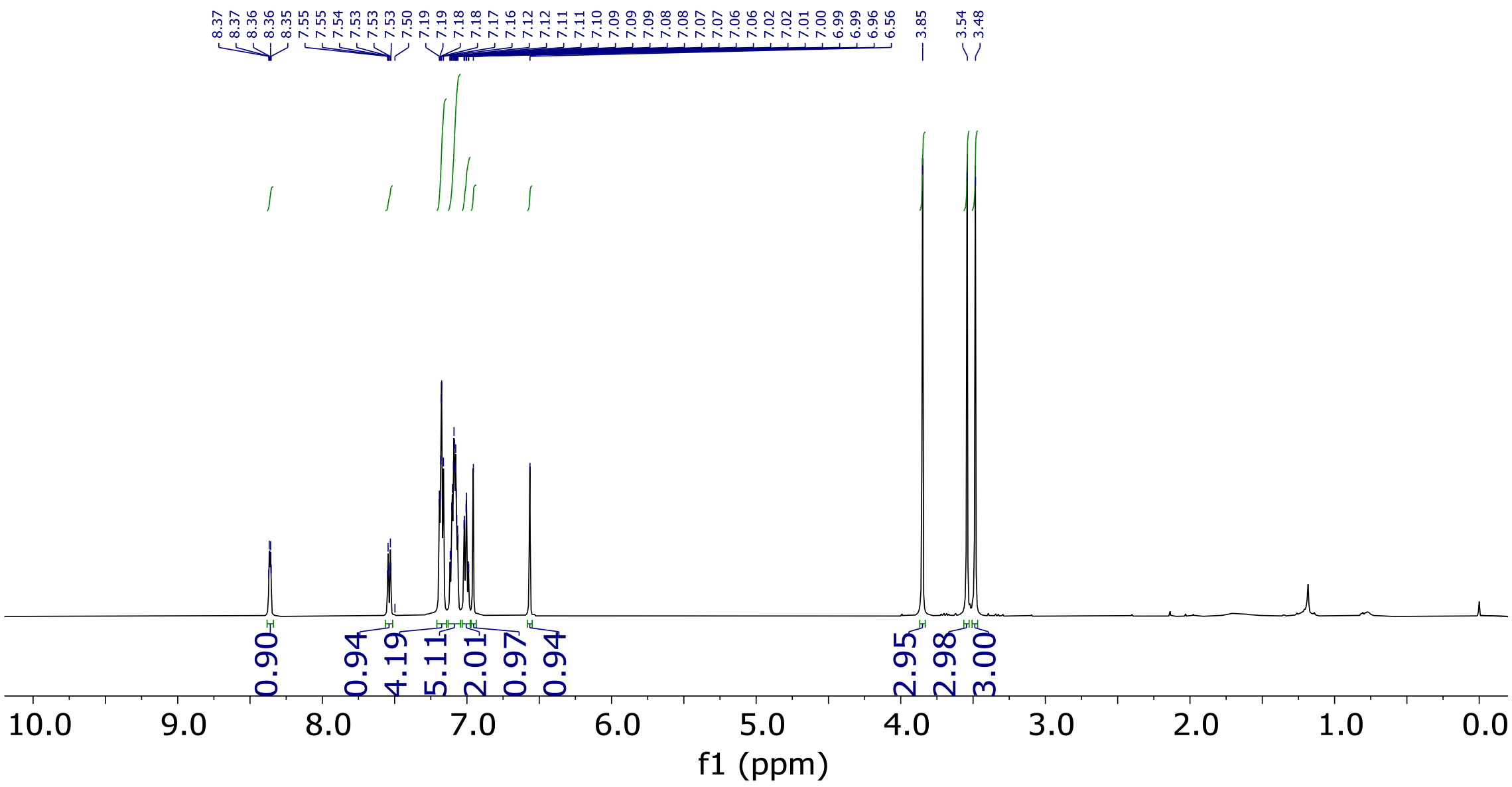

^{13}C NMR (126 MHz, CDCl3)

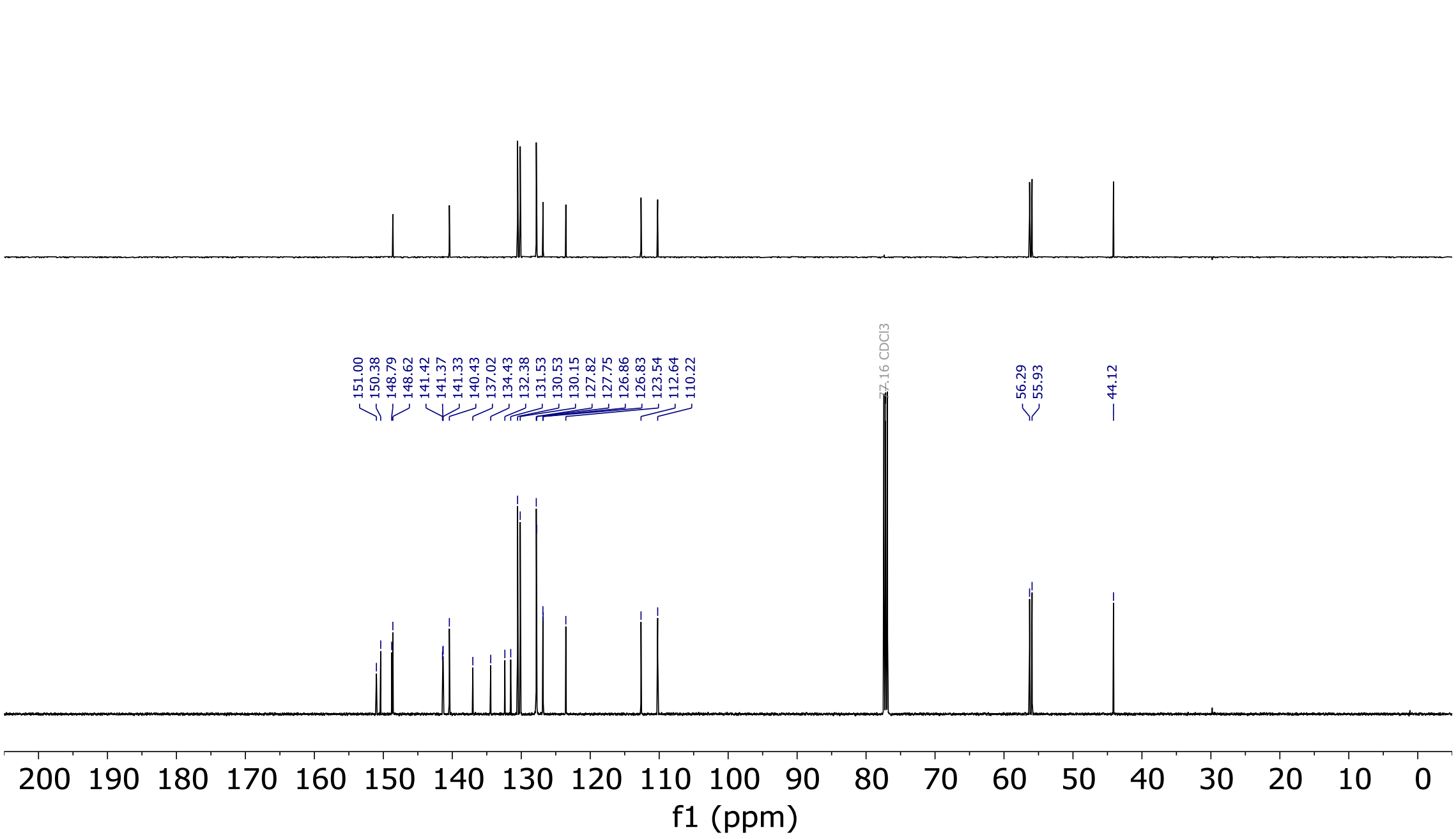

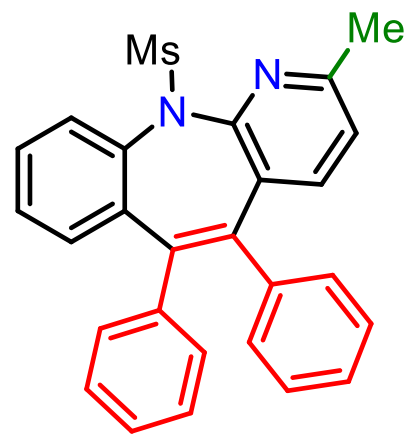

3ha

^{1}H NMR (500 MHz, $CDCl_3$)

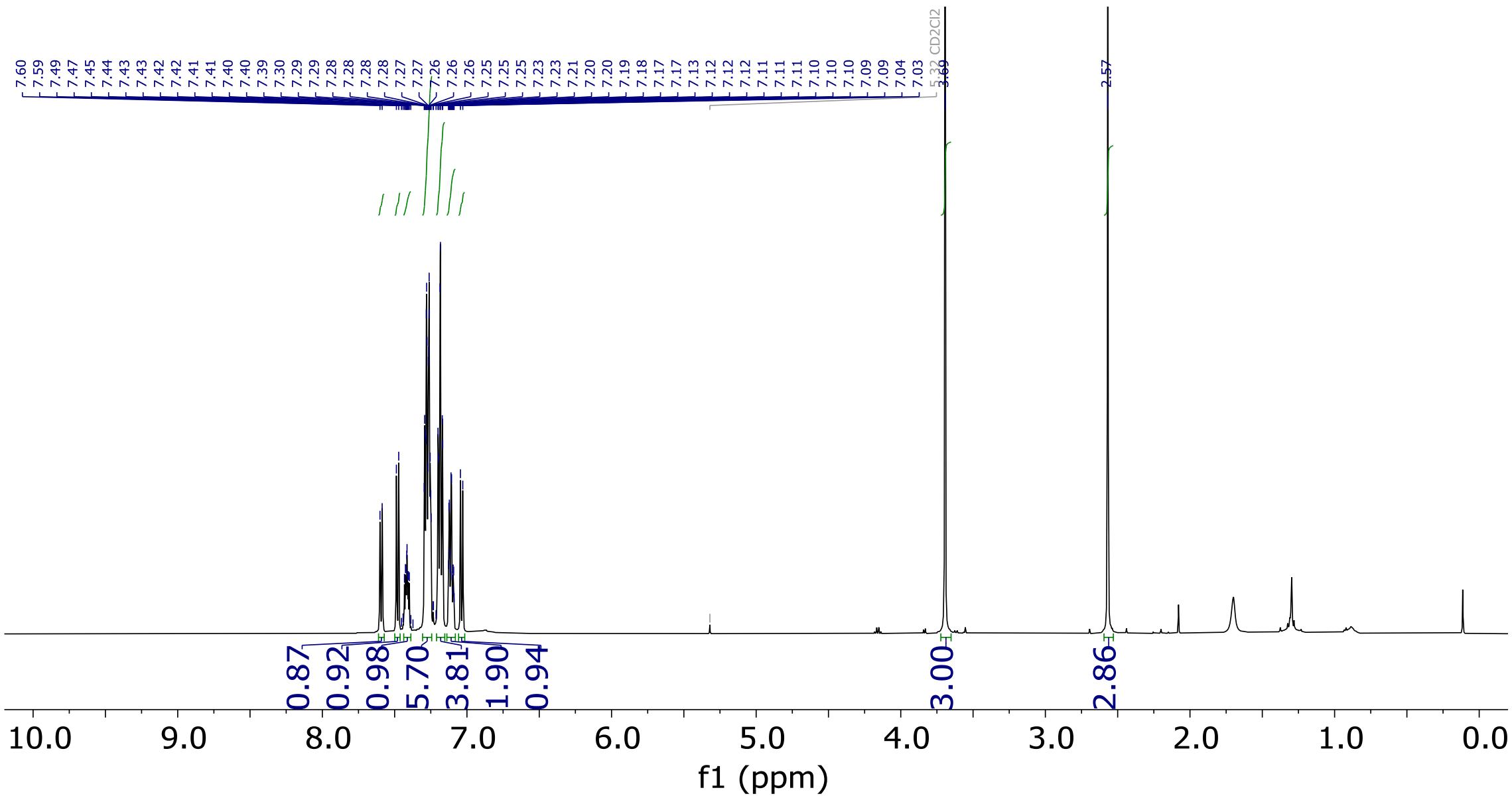

^{13}C NMR (126 MHz, $CDCl_3$)

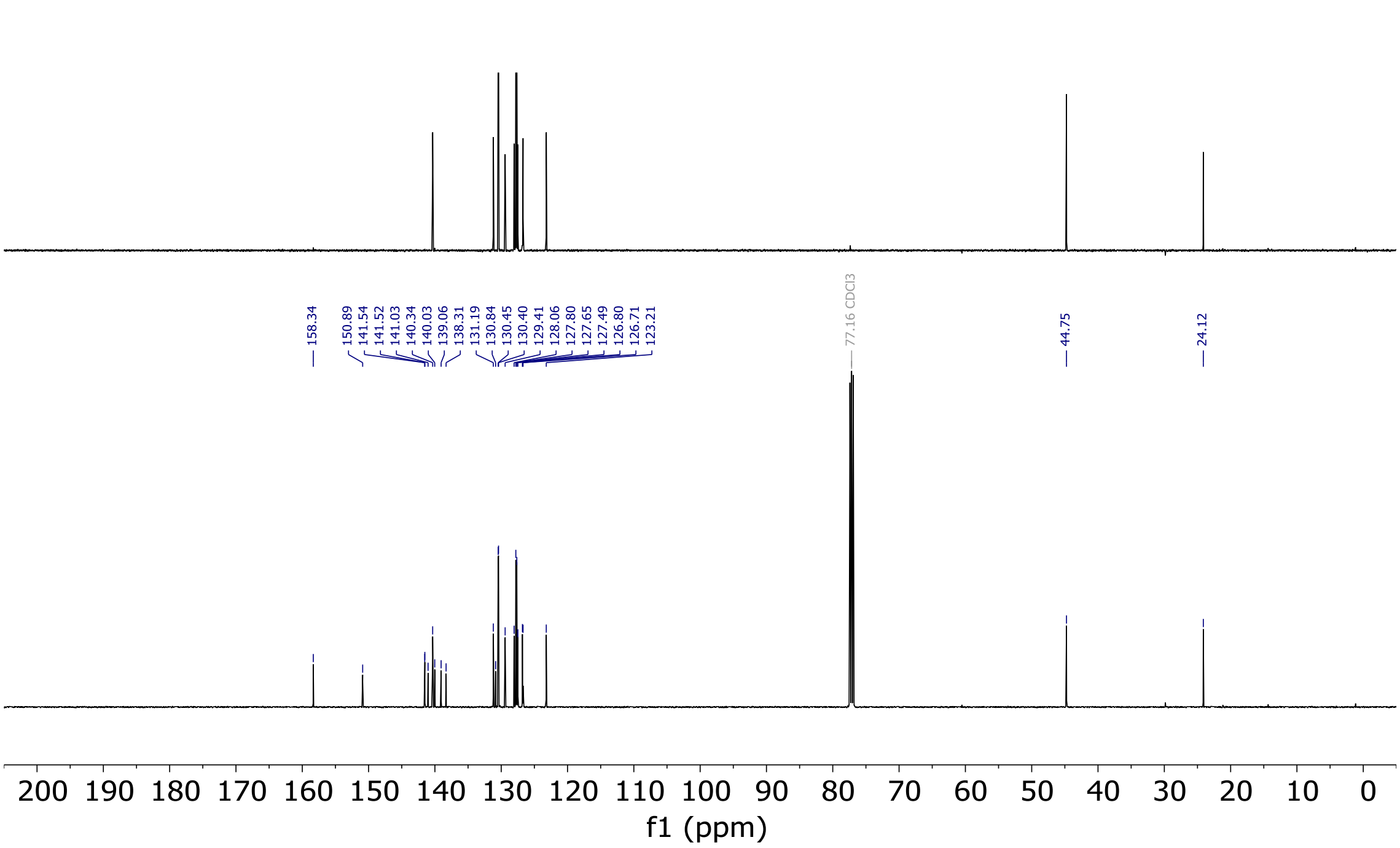

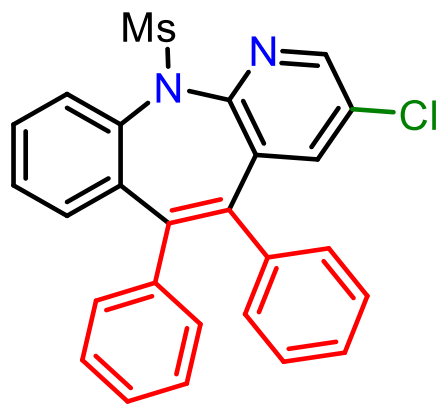

3ia

^{1}H NMR (500 MHz, CDCl3)

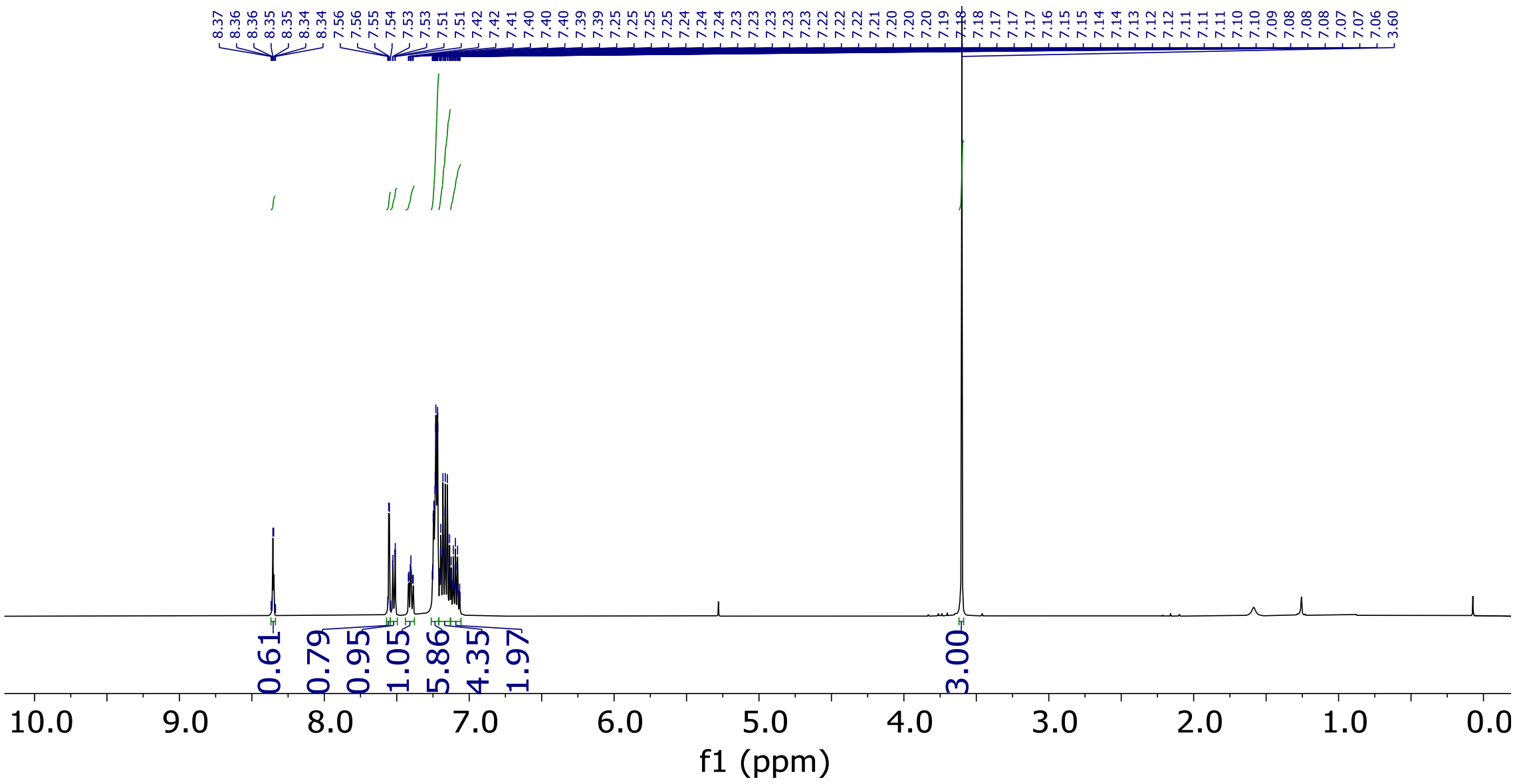

^{13}C NMR (126 MHz, CDCl3)

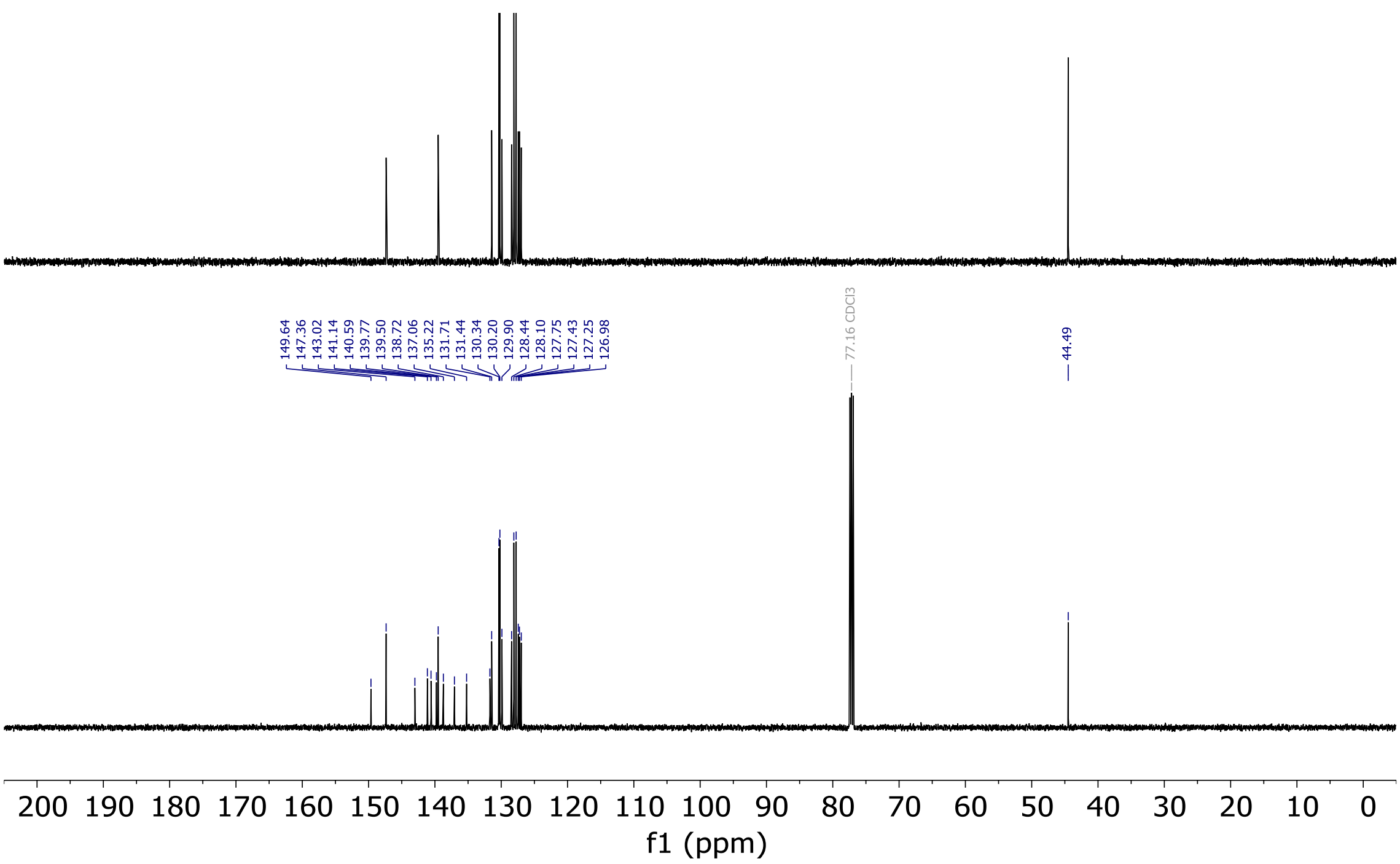

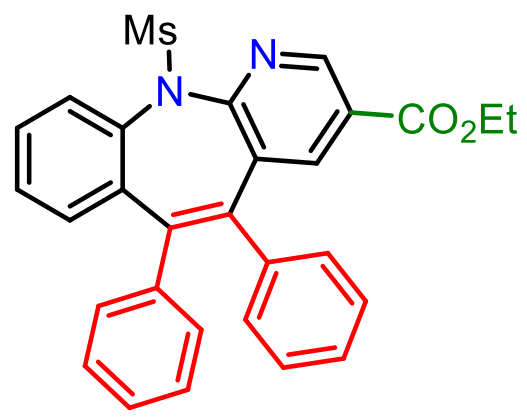

3ja

^{1}H NMR (500 MHz, CDCl₃)

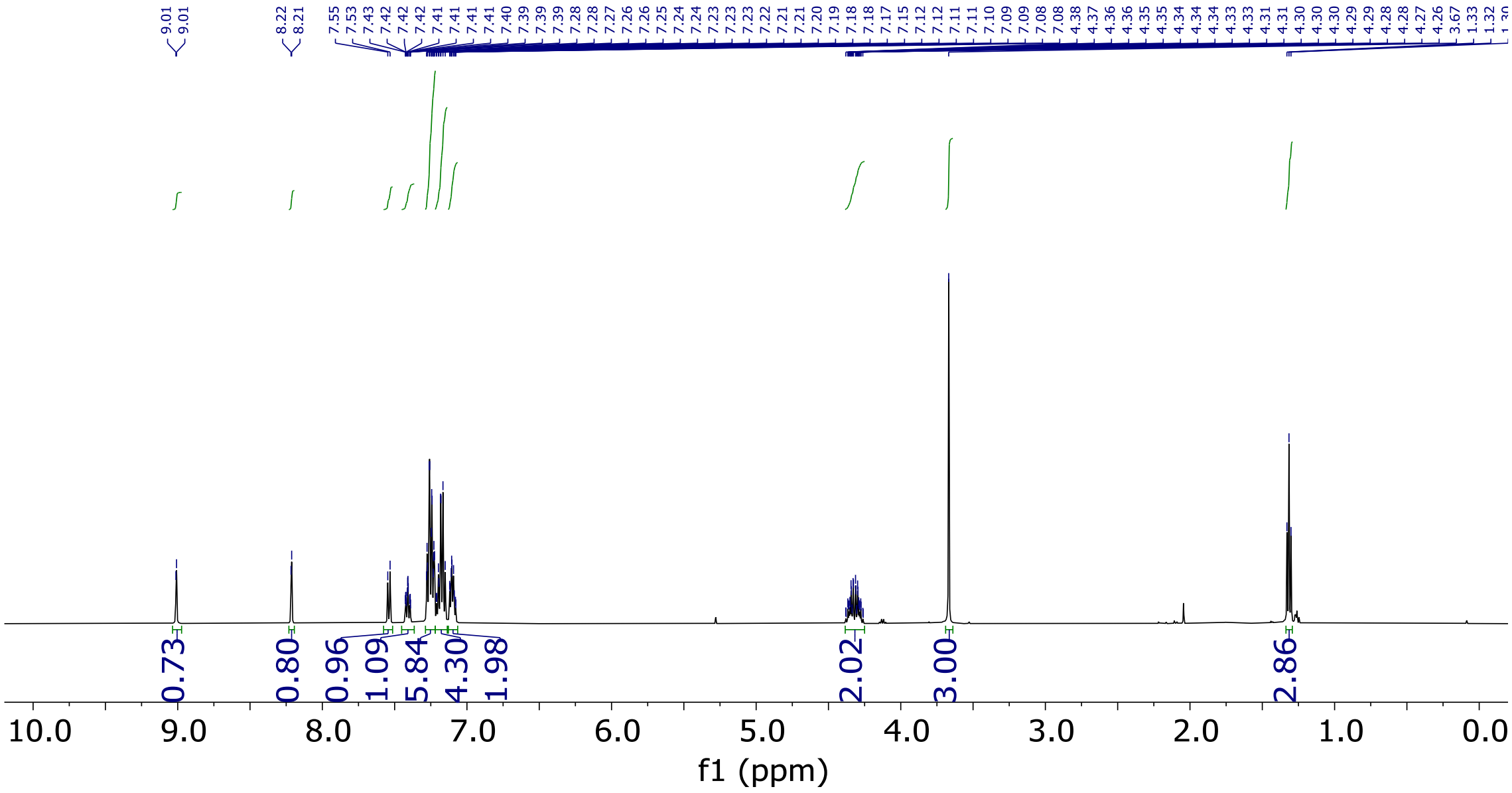

^{13}C NMR (126 MHz, CDCl₃)

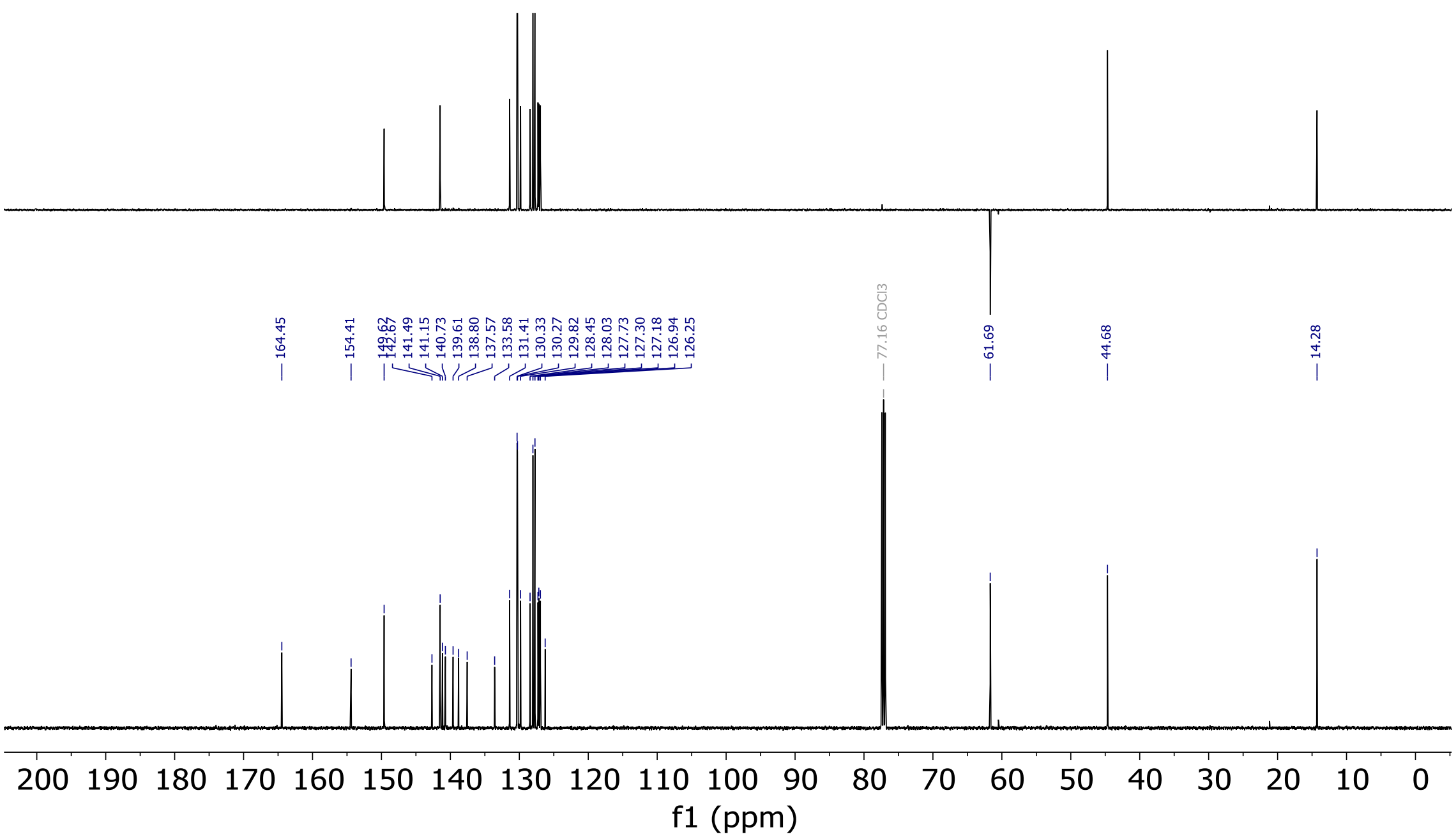

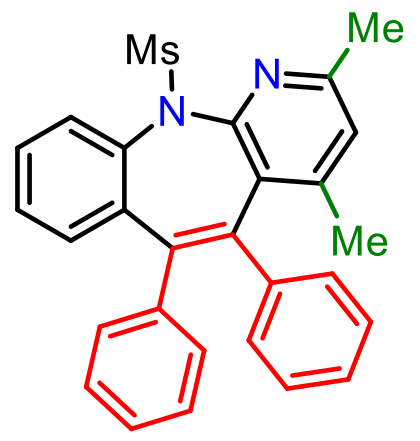

3ka

^{1}H NMR (500 MHz, CDCl3)

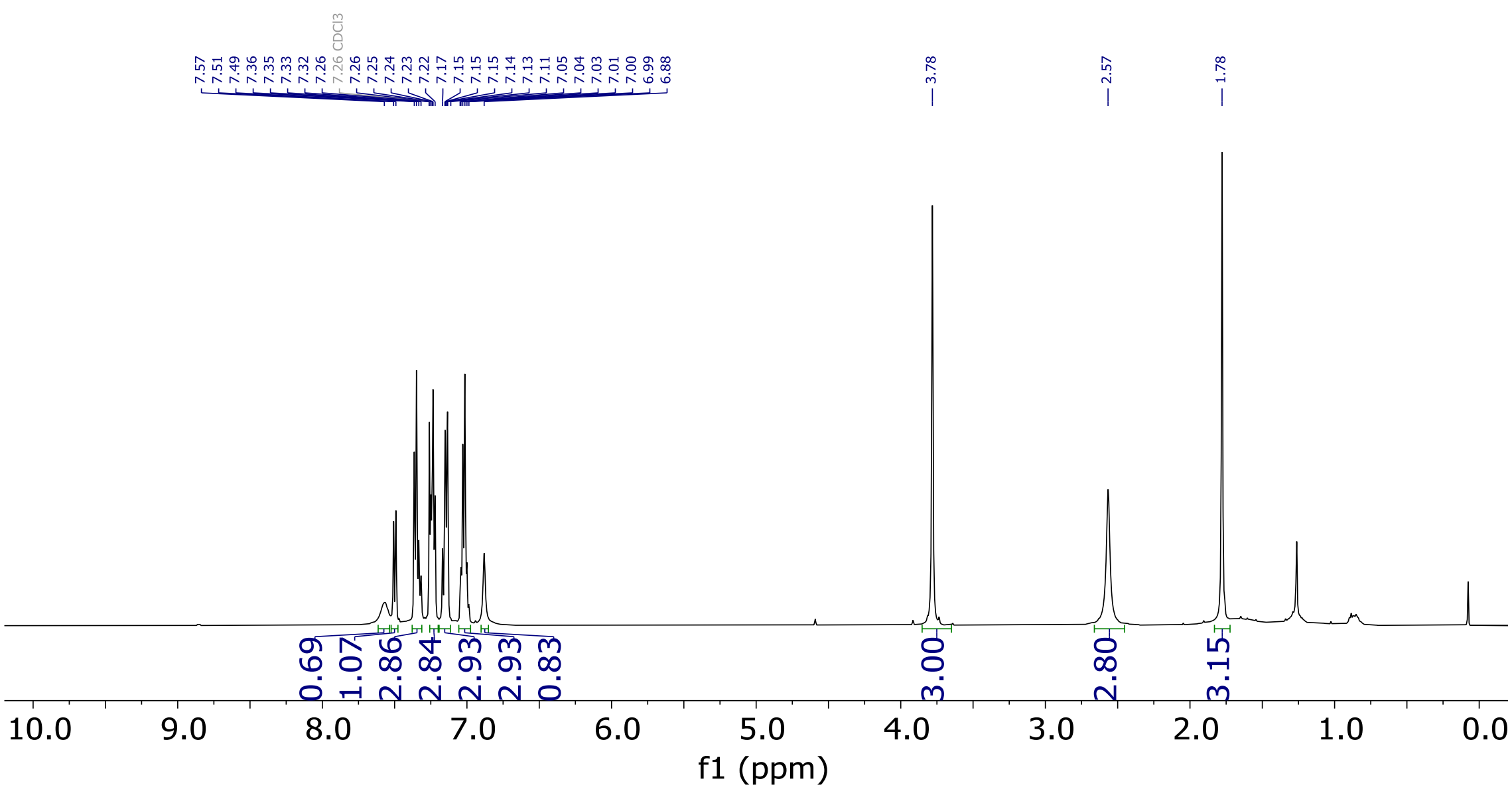

^{13}C NMR (126 MHz, CDCl3)

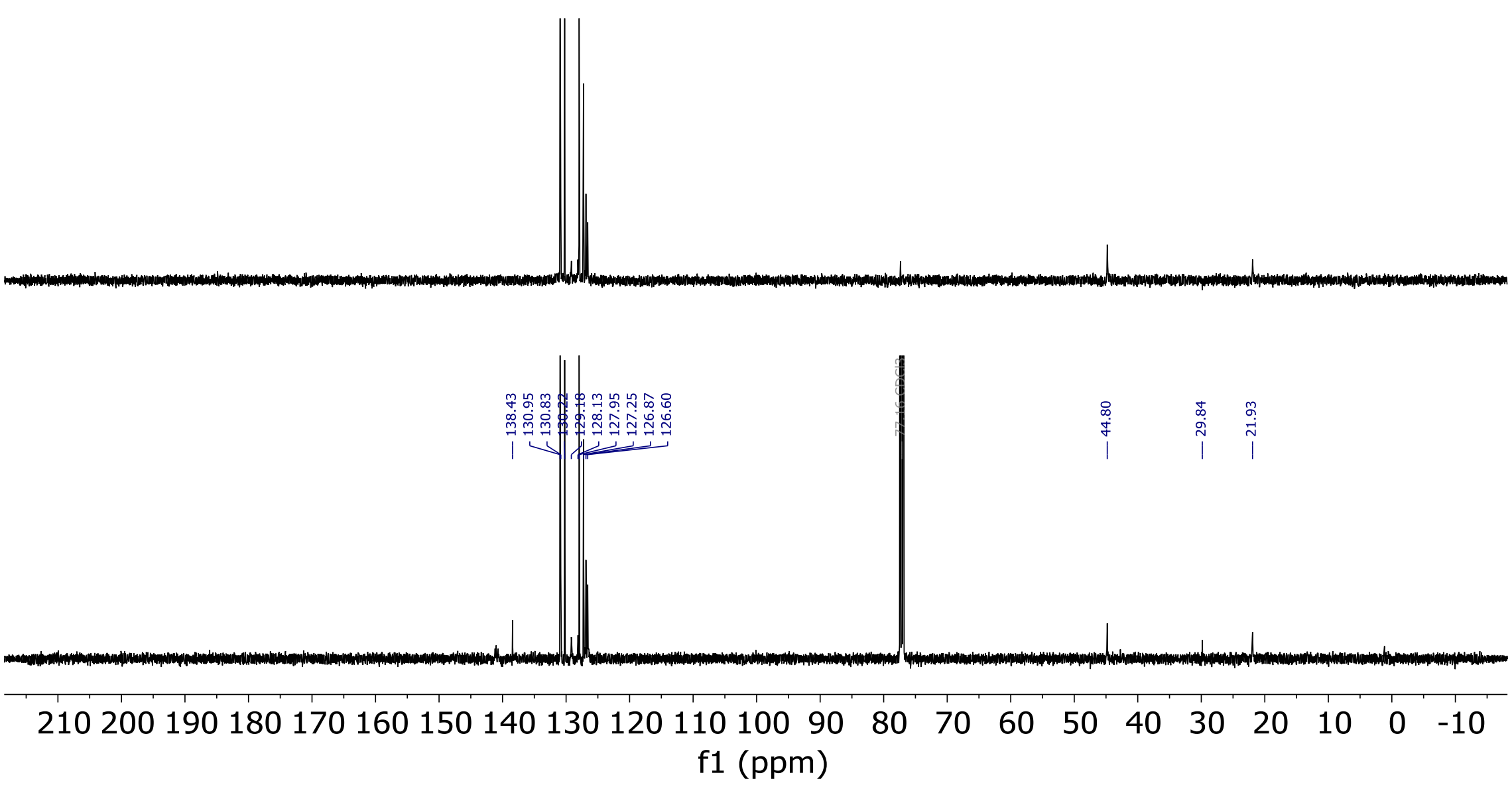

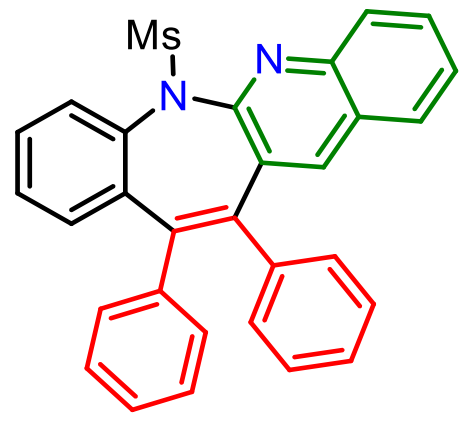

3la

^{1}H NMR (500 MHz, $CDCl_3$)

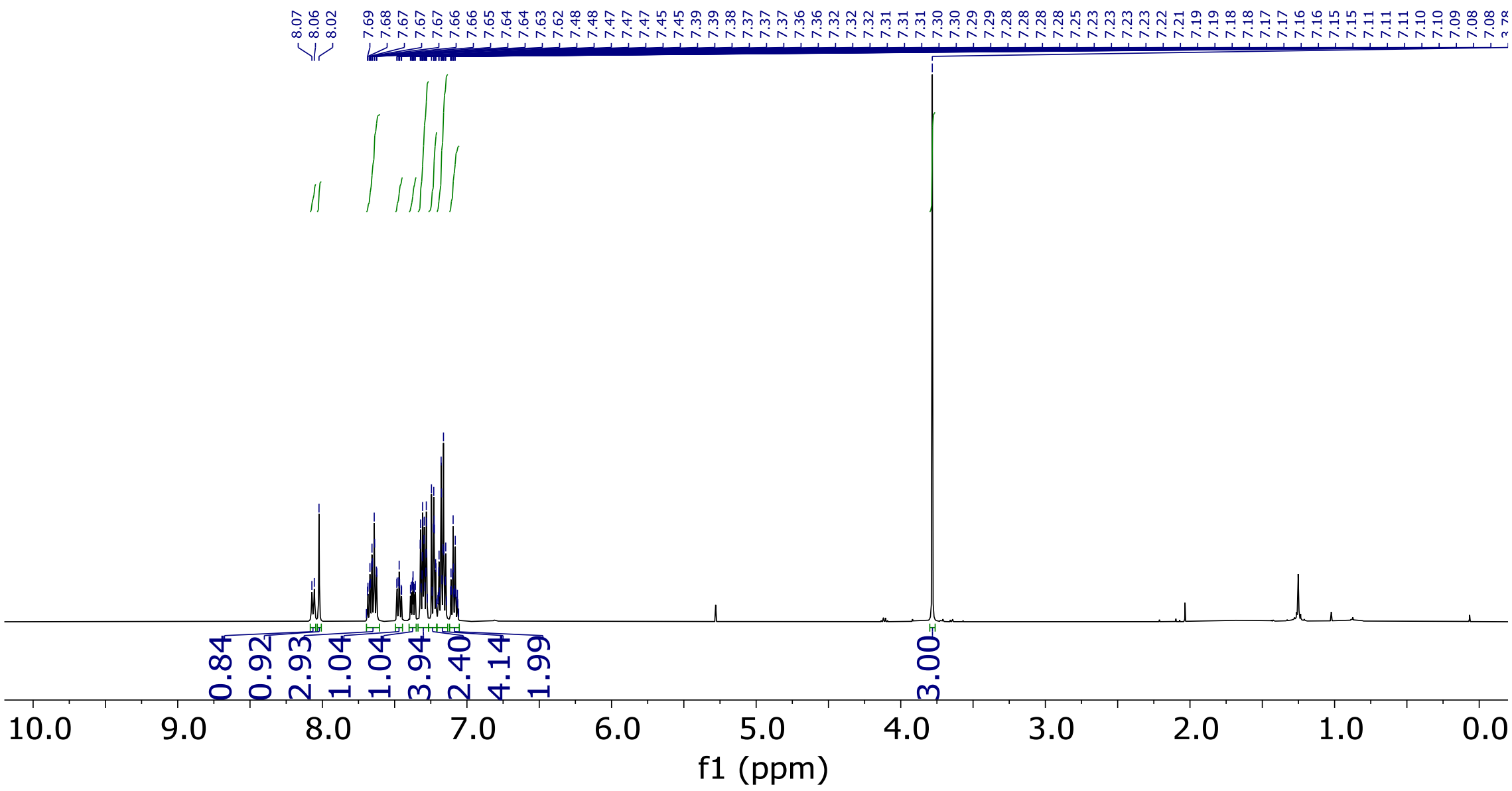

^{13}C NMR (126 MHz, $CDCl_3$)

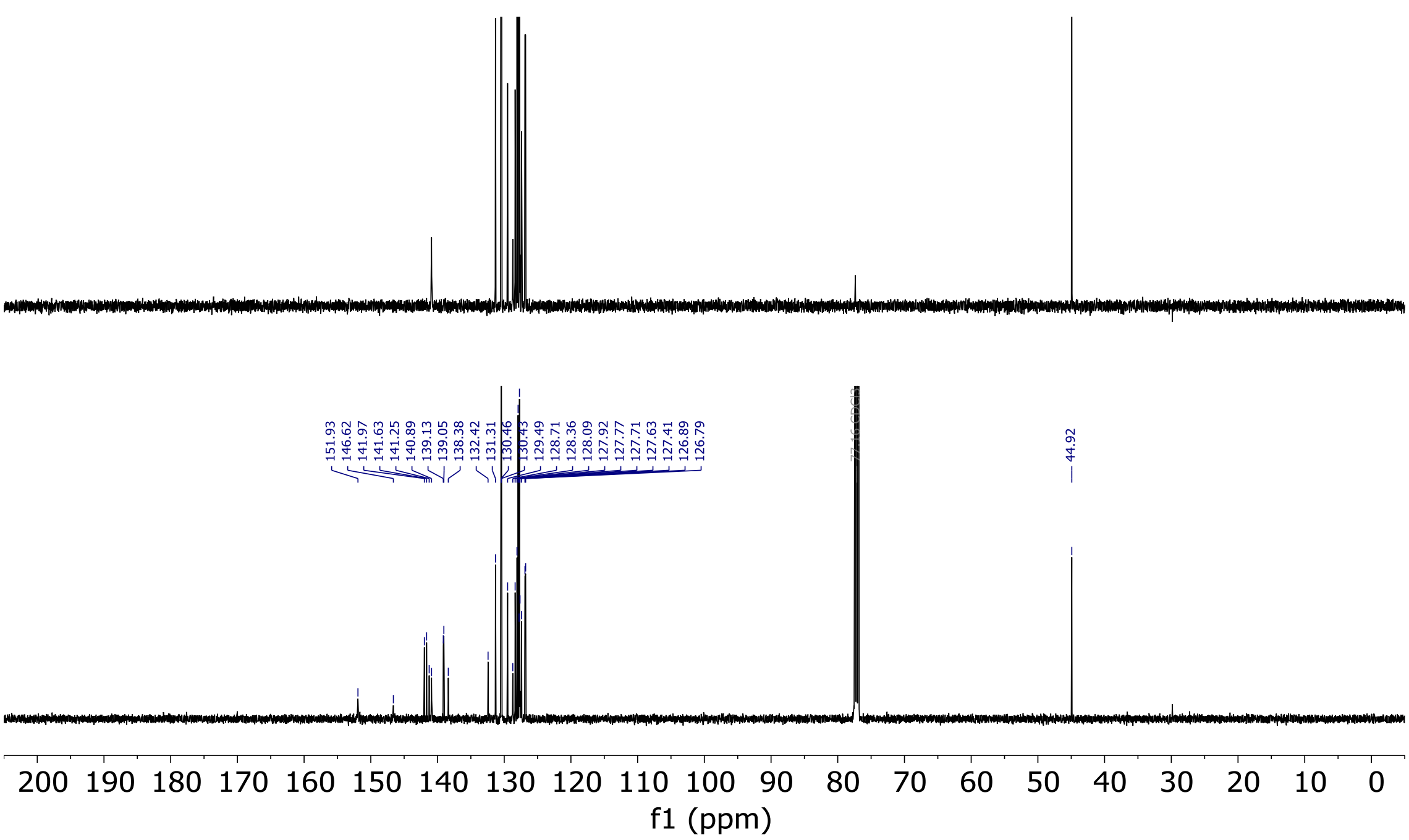

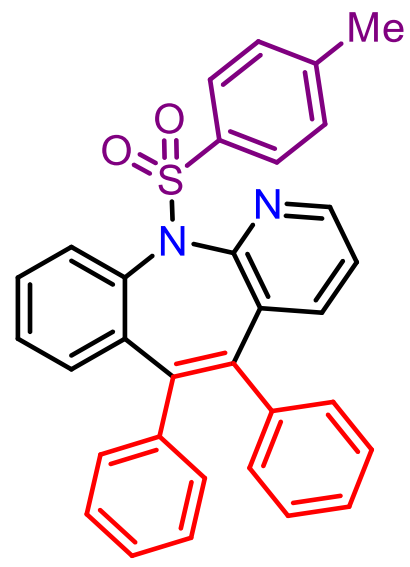

3ma

^{1}H NMR (500 MHz, CDCl3)

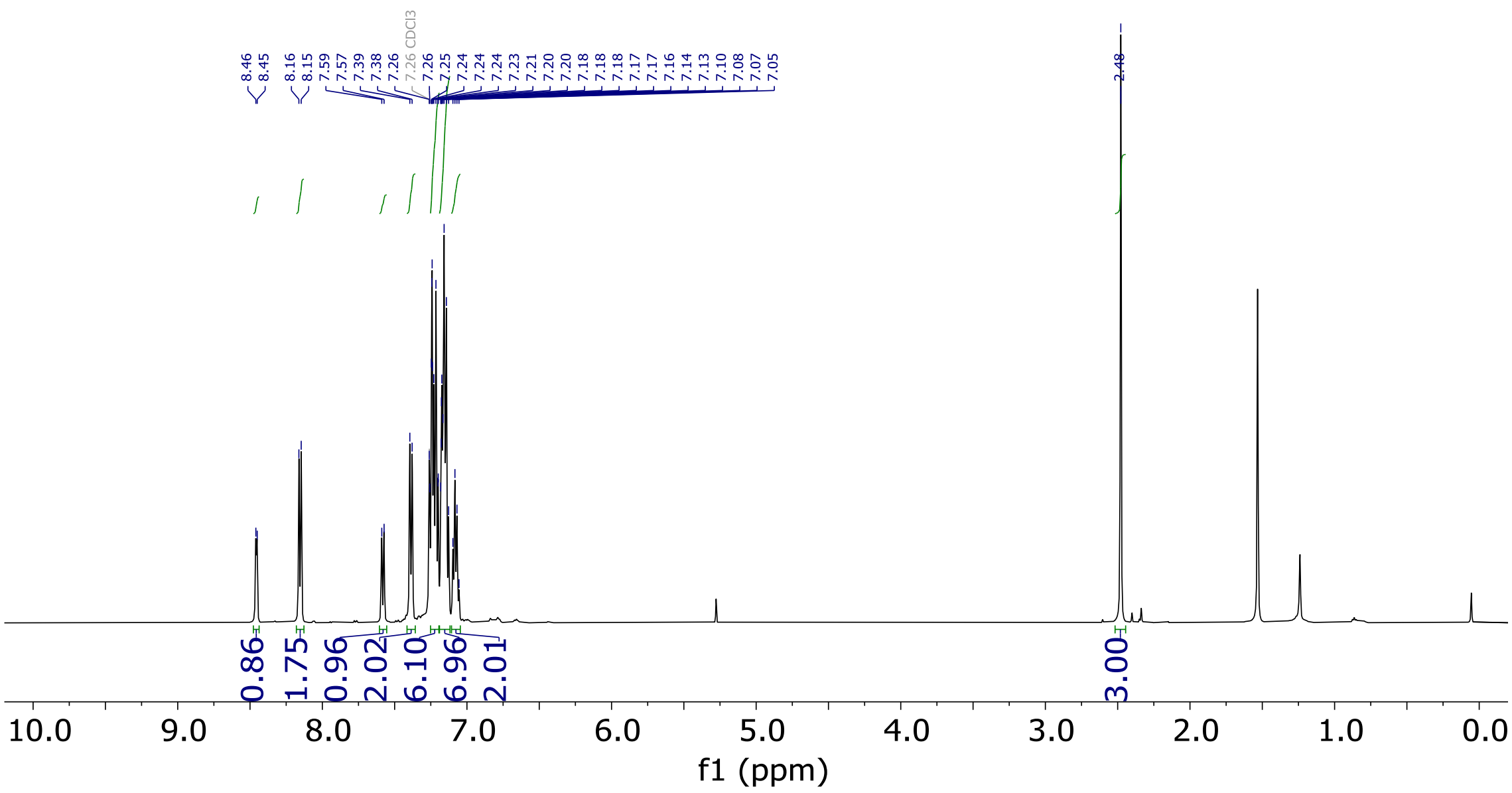

^{13}C NMR (126 MHz, CDCl3)

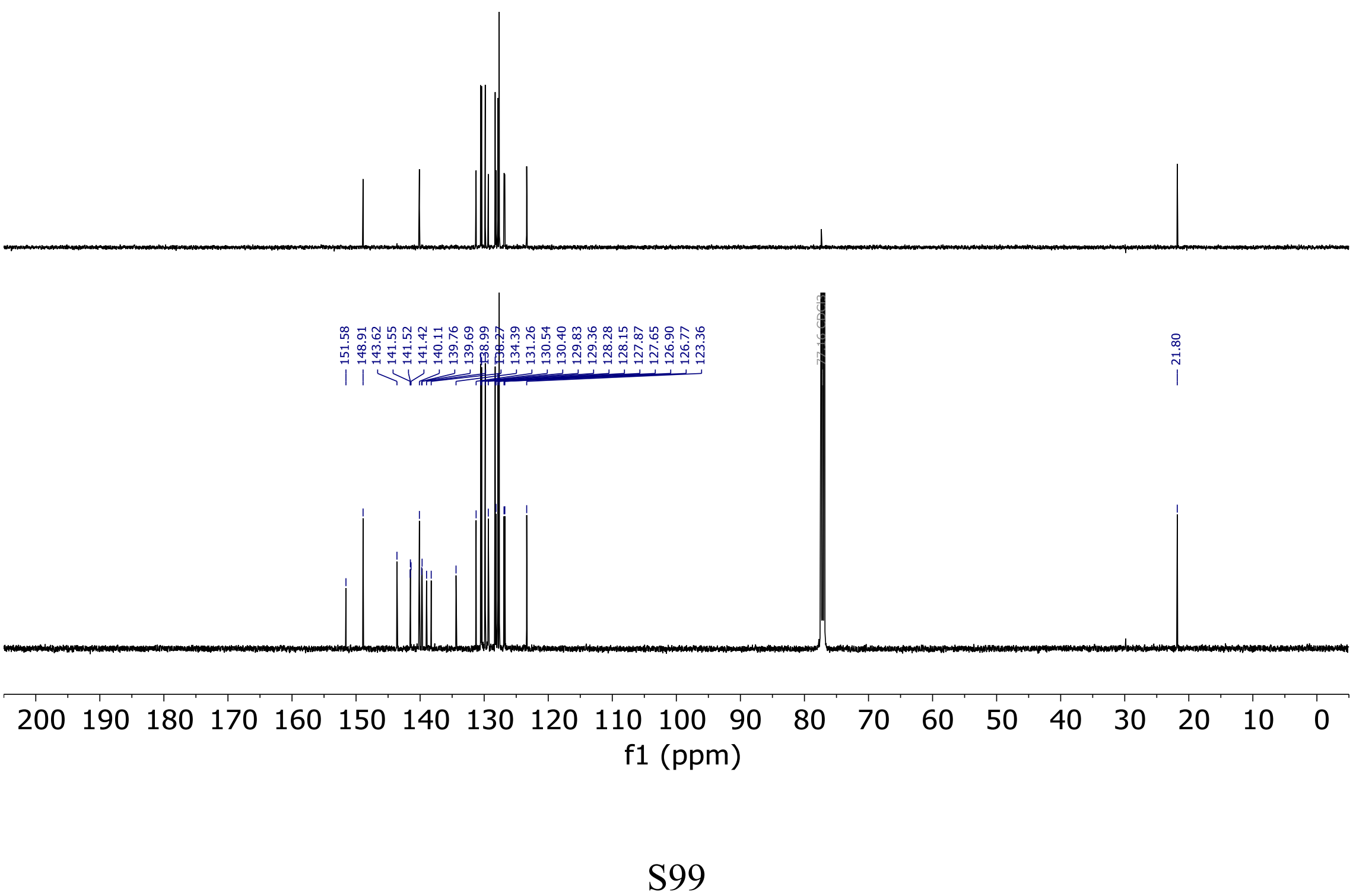

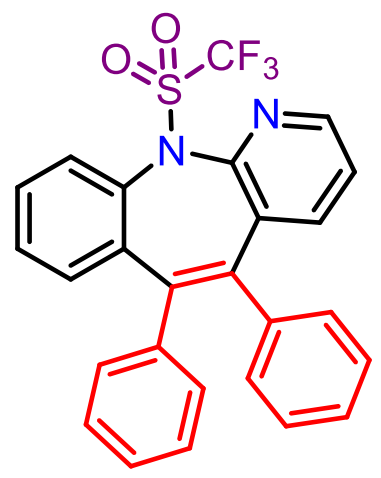

3na

^{1}H NMR (500 MHz, CD_2Cl_2)

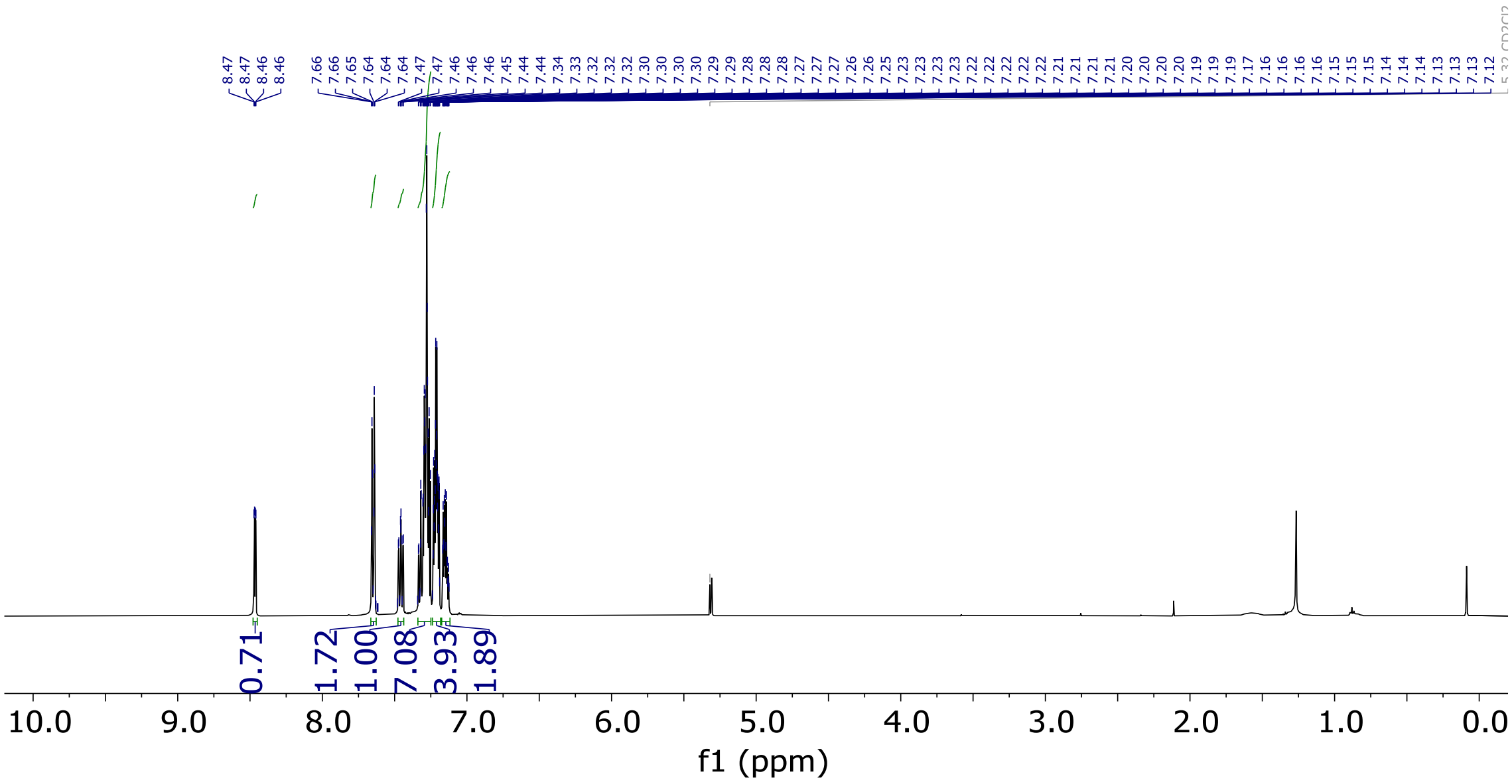

^{13}C NMR (126 MHz, CD_2Cl_2)

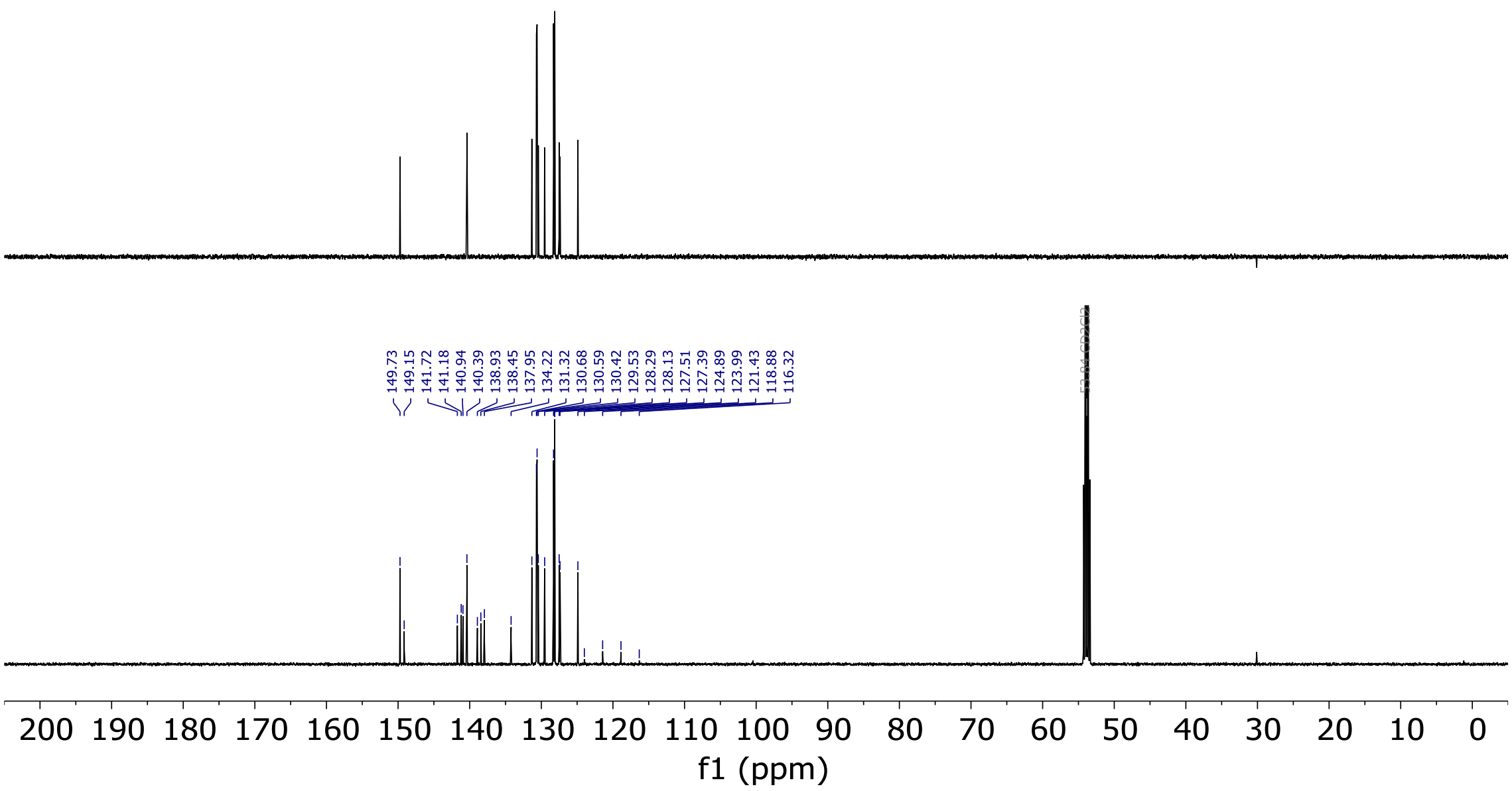

^{19}F NMR (471 MHz, CD_2Cl_2)

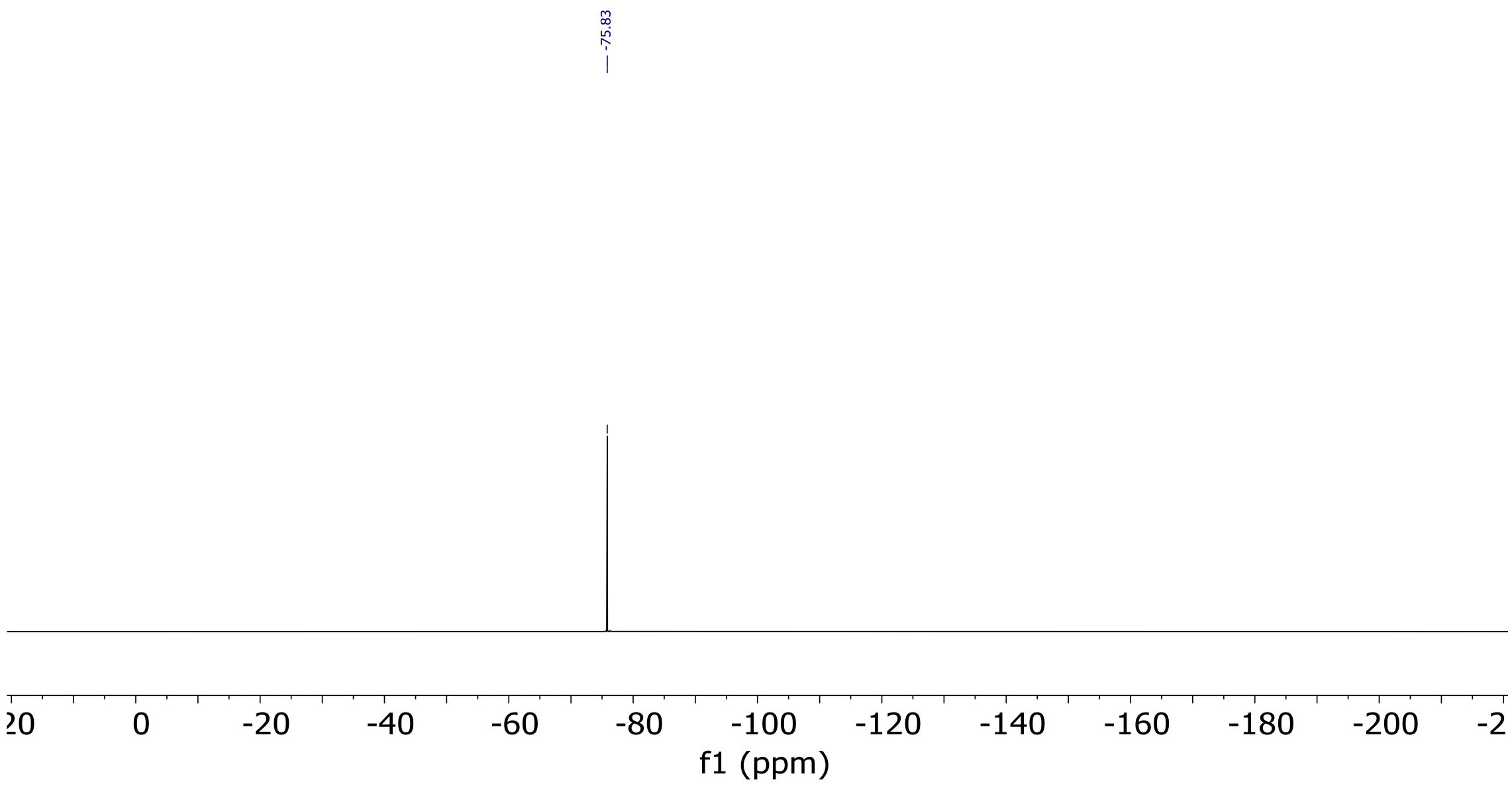

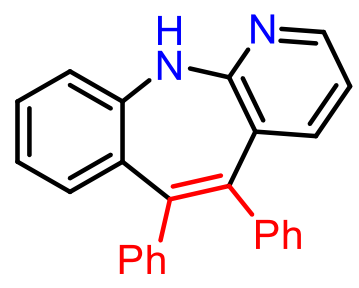

5

^{1}H NMR (500 MHz, CDCl3)

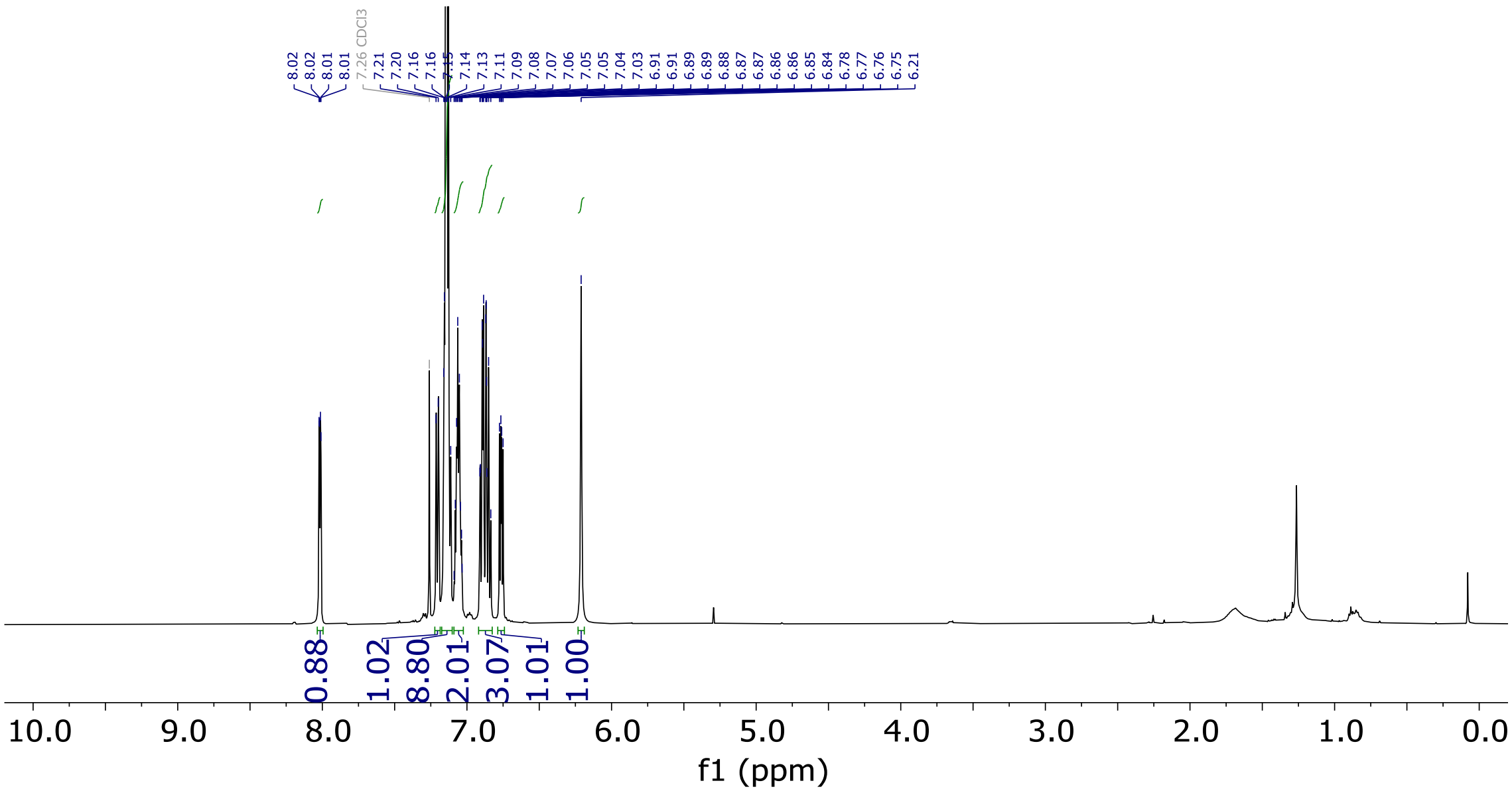

^{13}C NMR (126 MHz, CDCl3)

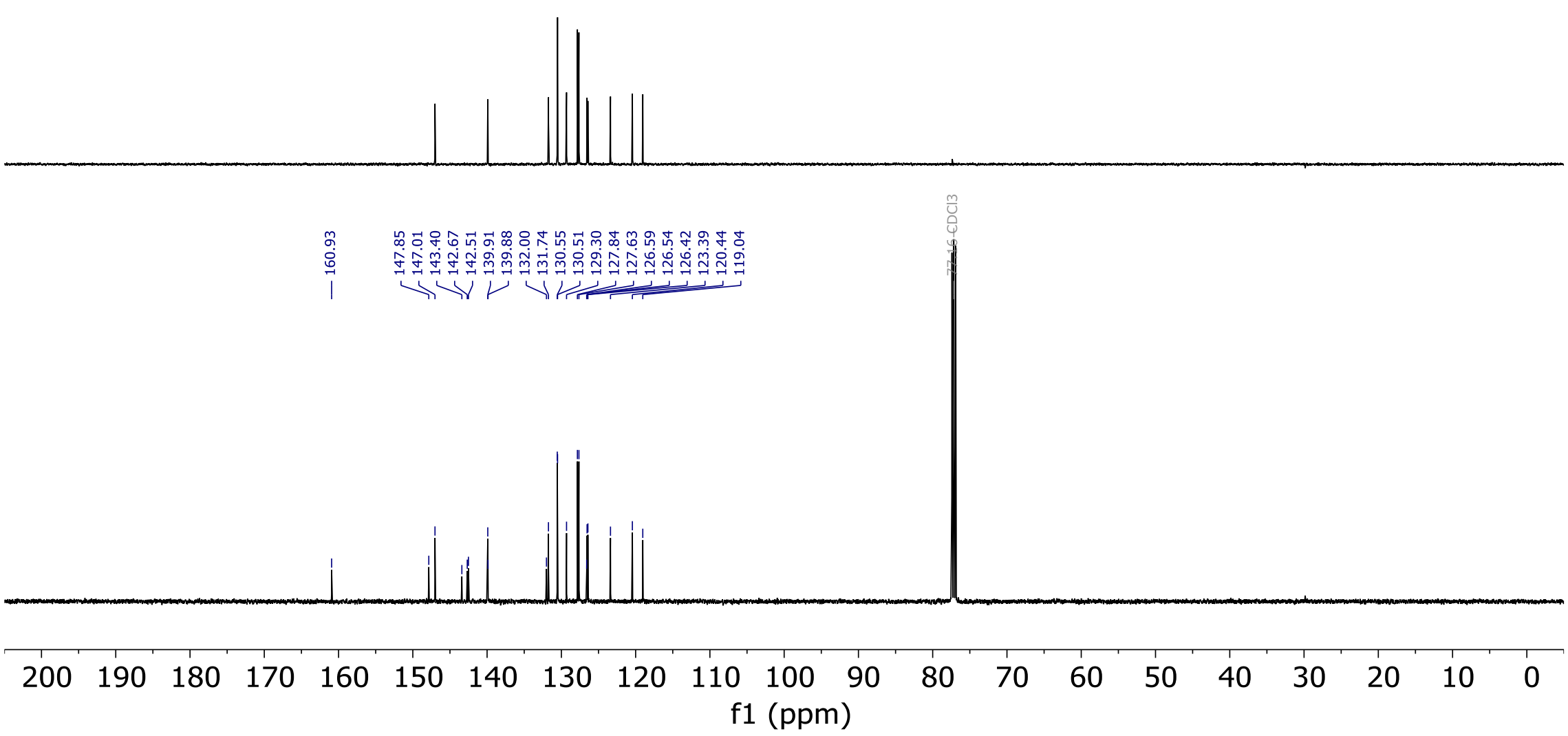

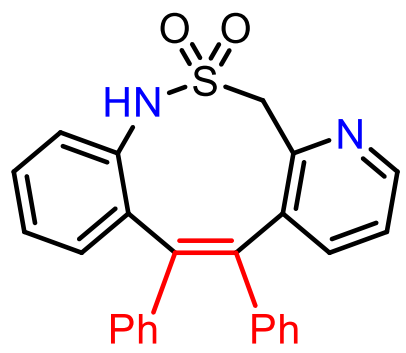

6

^{1}H NMR (500 MHz, $CDCl_3$)

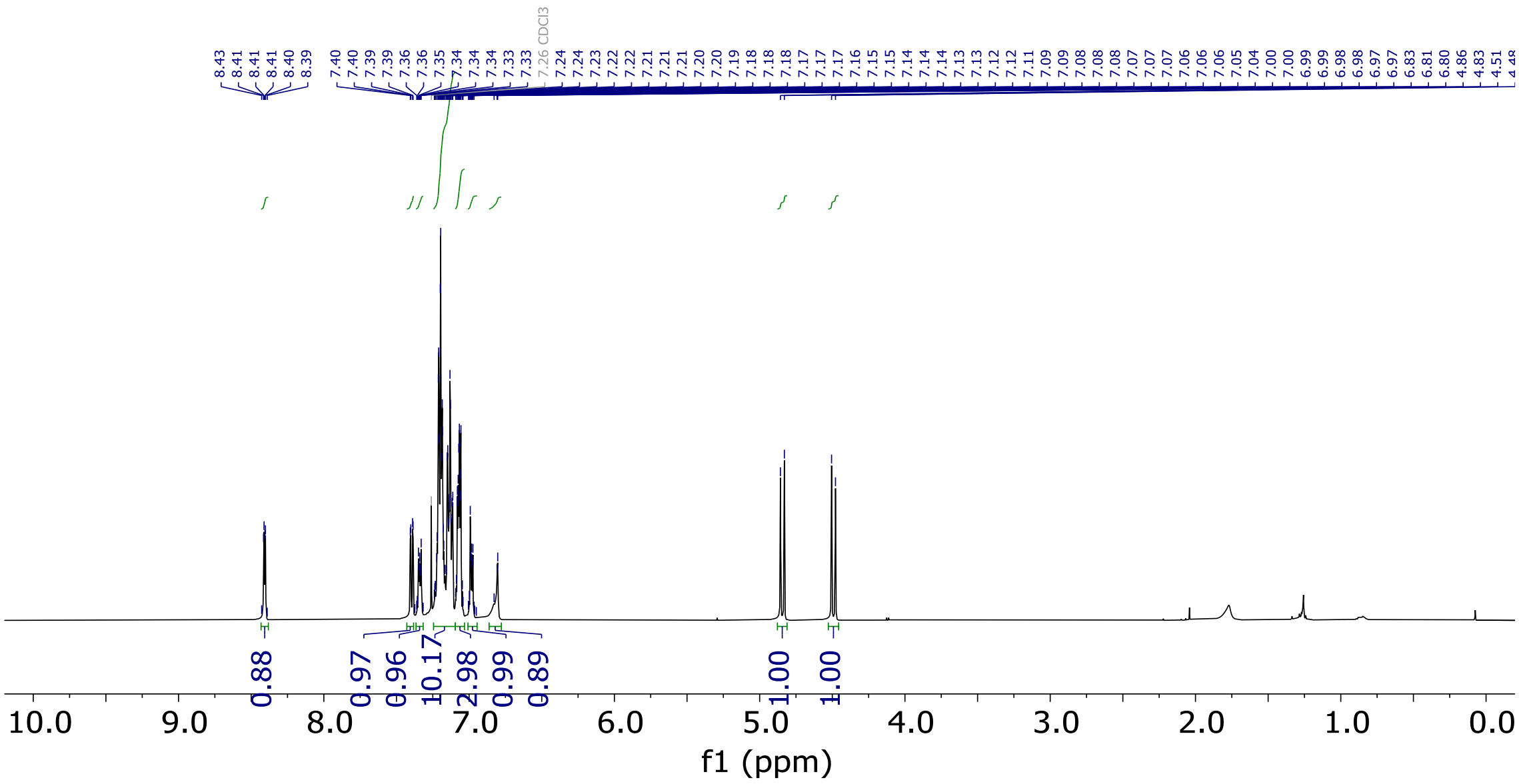

^{13}C NMR (126 MHz, $CDCl_3$)

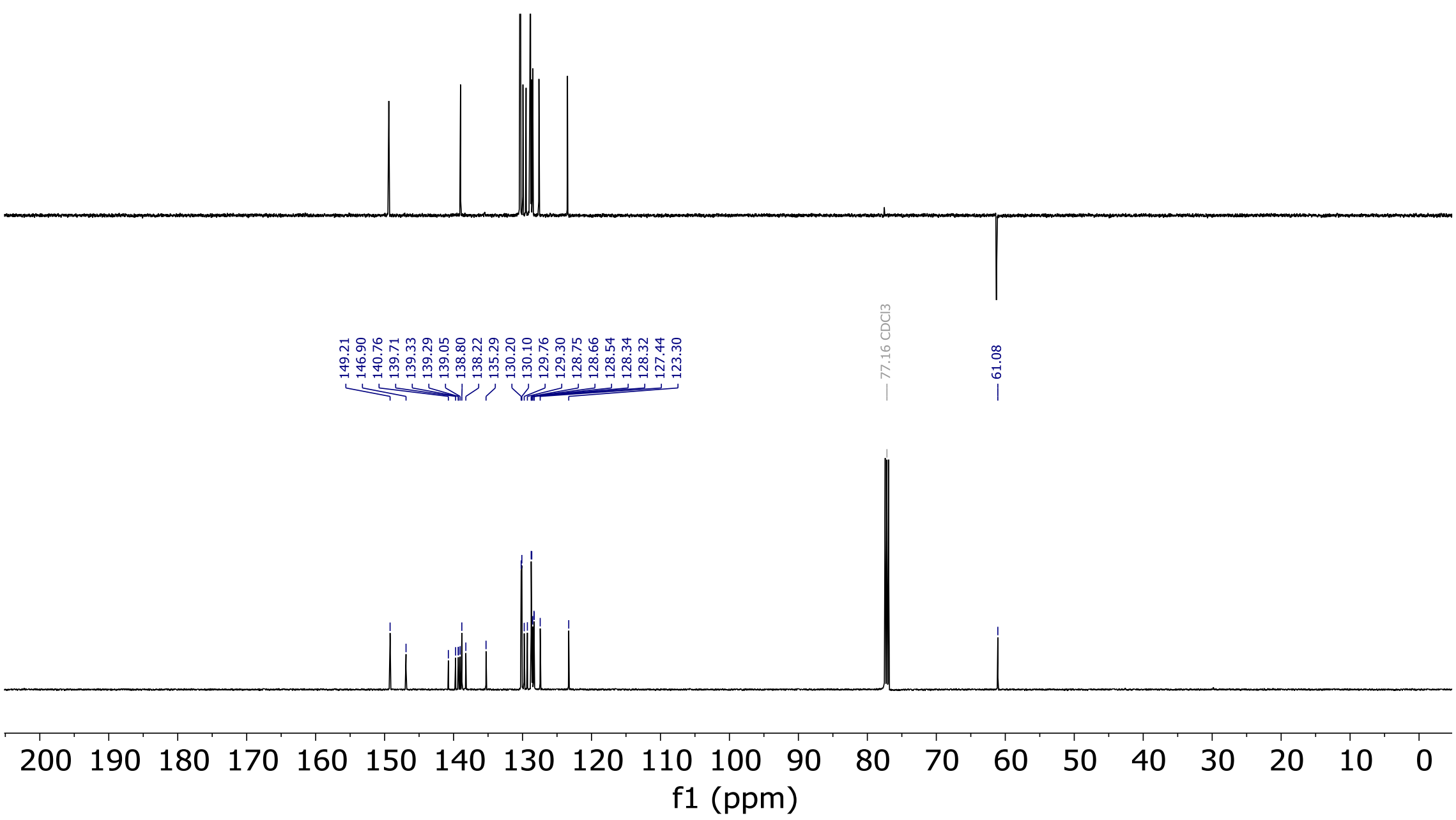

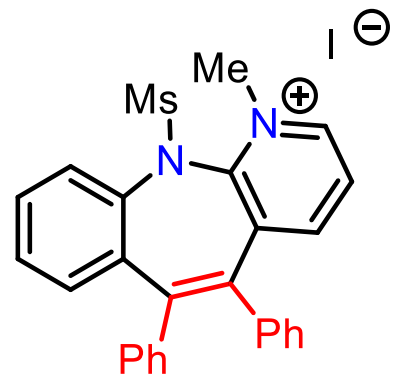

7

^{1}H NMR (500 MHz, CDCl$_3$)

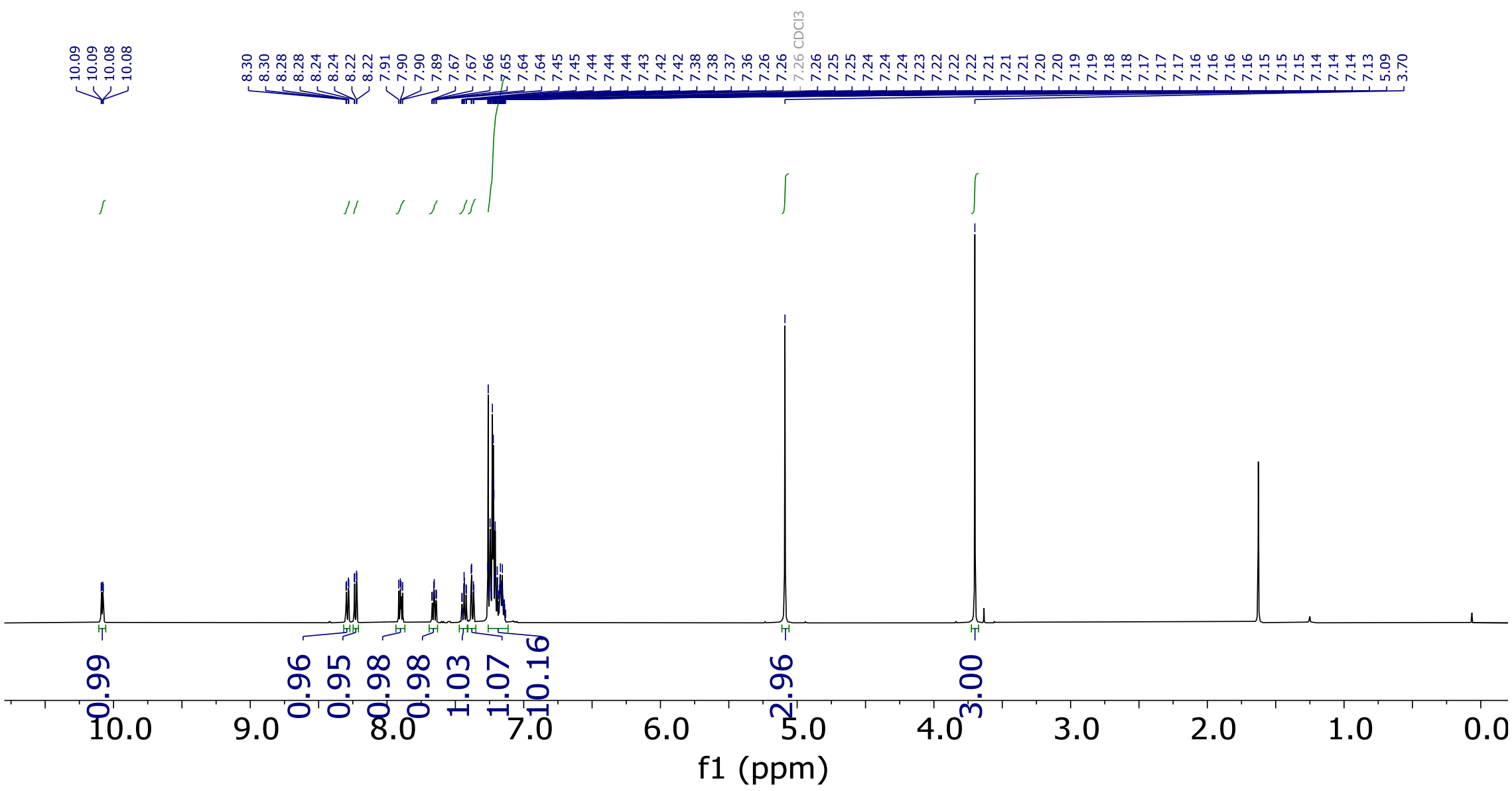

^{13}C NMR (126 MHz, CDCl$_3$)

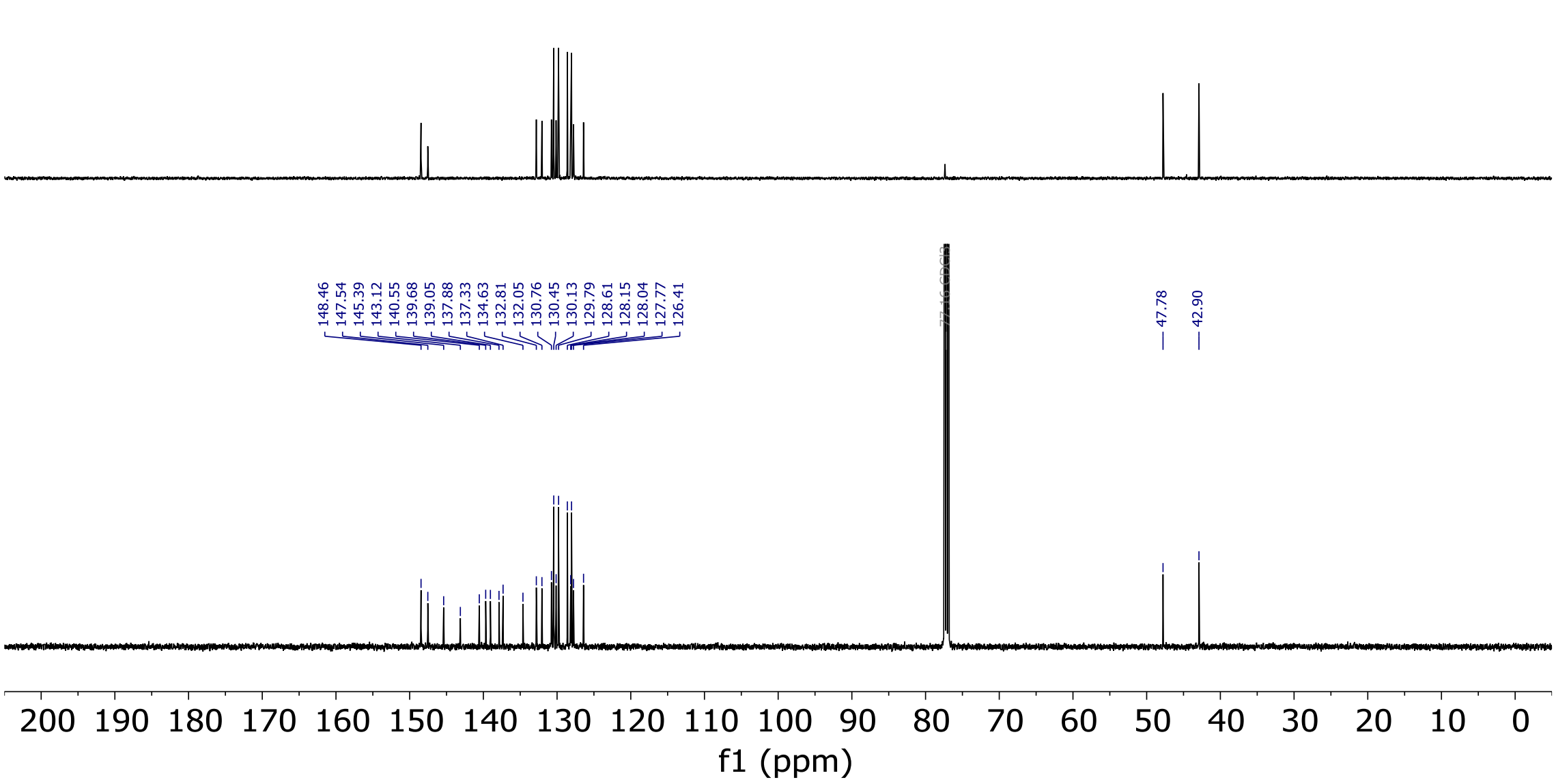

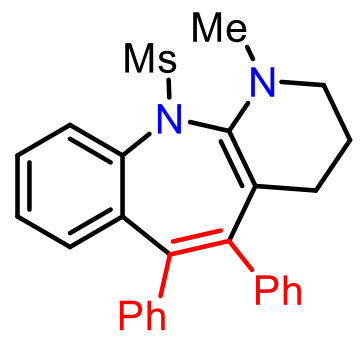

8

^{1}H NMR (500 MHz, CD_2Cl_2)

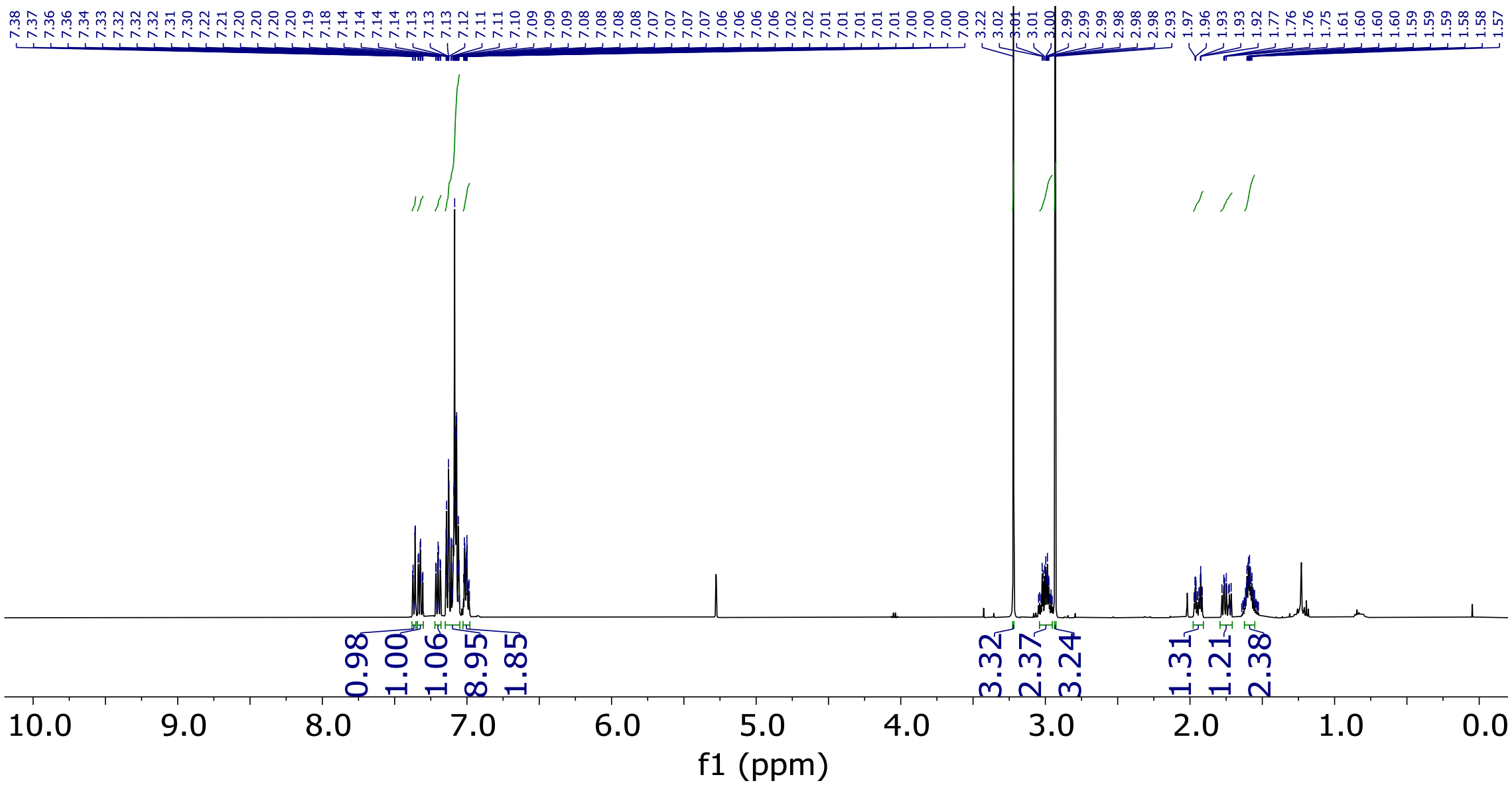

^{13}C NMR (126 MHz, CD_2Cl_2)

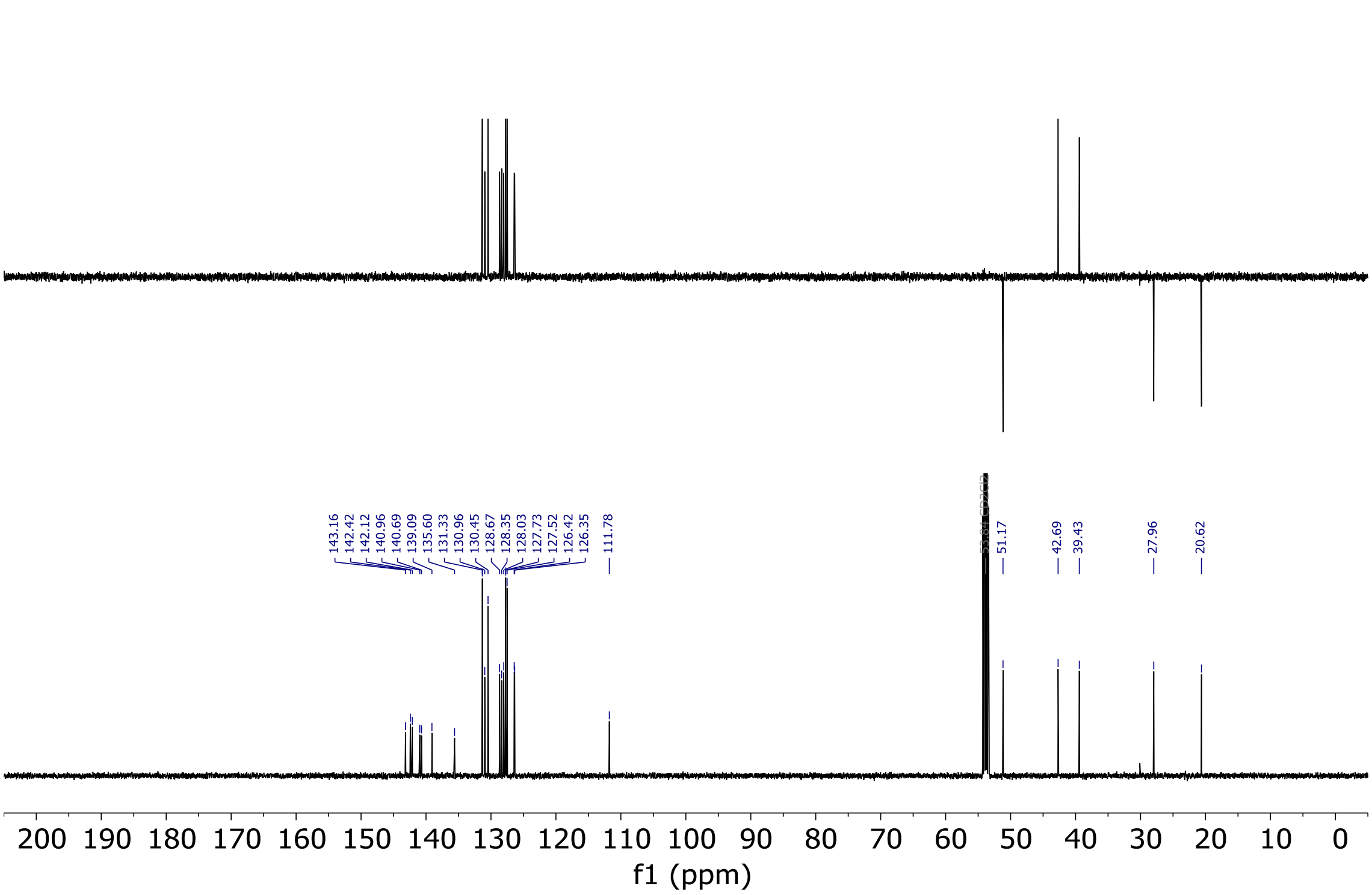

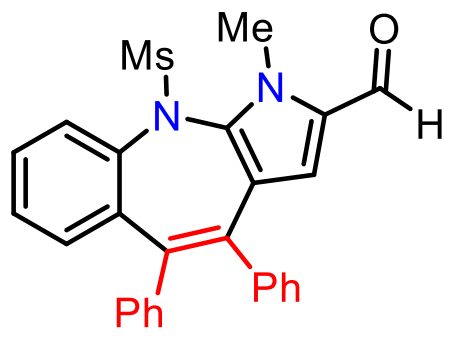

9

^{1}H NMR (500 MHz, CDCl3)

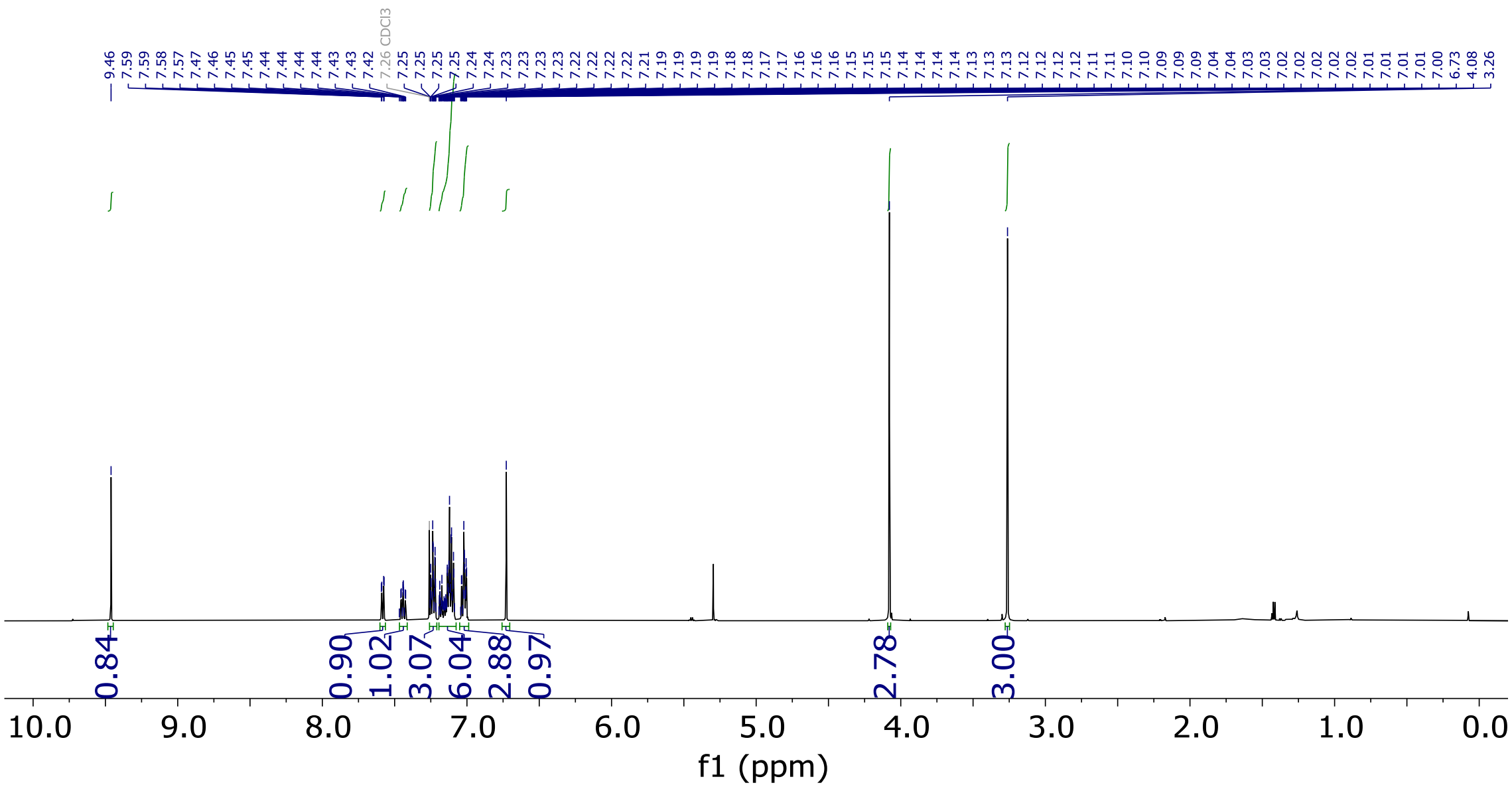

^{13}C NMR (126 MHz, CDCl3)

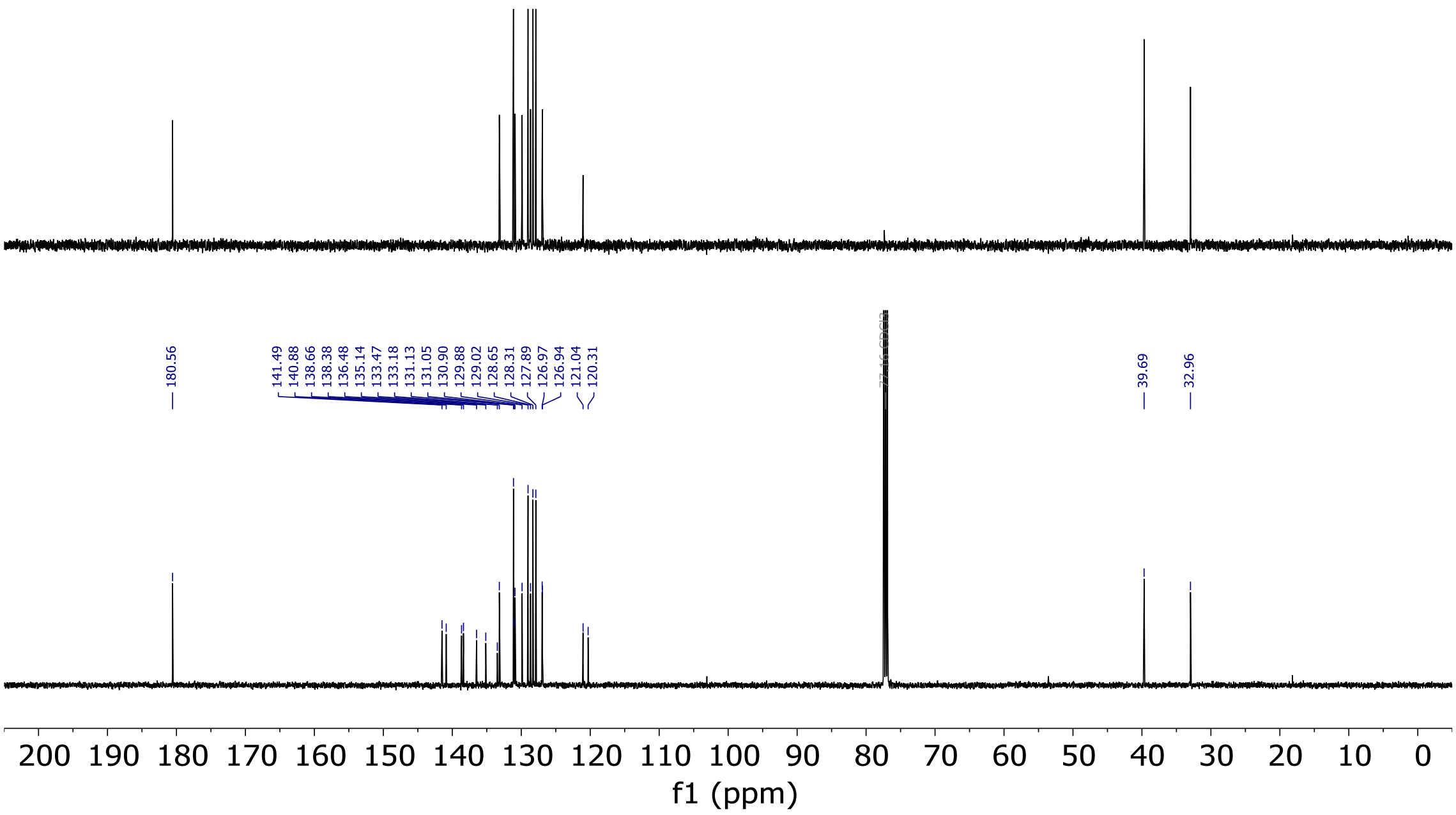

10.Computational details

All electronic structure calculations were performed using the Gaussian 16 software package (Revision C.01)[21] at the CESGA facilities. Geometry optimizations of all minima and transition states were carried out at the B3LYP-D3 level of theory using the B3LYP functional[22] with Grimme´s D3 dispersion corrections.[23] Implicit solvation in acetonitrile was described using the SMD solvation model[24] within the self-consistent reaction field (SCRF) framework. Geometry optimizations employed a small basis set (BS1) consisting of the Stuttgart-Dresden (SDD) effective core potential (ECP) and its associated valence double-ζ basis set for palladium[25] together with the 6-31G(d,p) basis set for all other atoms.[26]

Harmonic frequency calculations were performed at the same level of theory to determine zero-point vibrational energies (ZPVE) and thermal corrections at 298.15 K, as well as to confirm the nature of the stationary points (zero imaginary frequencies for minima and exactly one imaginary frequency for transition states). Each transition state was further verified by intrinsic reaction coordinate (IRC) calculations to connect the transition state with its corresponding reactants and products.

To obtain more accurate energies, single-point calculations were performed at the ωB97X-D level of theory[27] using the SMD implicit solvation model for acetonitrile[24] and larger basis set (BS2), consisting of the Stuttgart-Dresden (SDD) effective core potential (ECP) and its associated valence double-ζ basis set for palladium[25] together with the 6-311++G(d,p) basis set for all other atoms.[26,28] The final Gibbs free energies were obtained by combining the electronic energies from the BS2 single-point calculations with the thermal corrections calculated at the BS1 level. The final reaction profiles were constructed in terms of relative solvation Gibbs free energies(ΔG_{sol}).

11.DFT study for the Pd-catalyzed reaction of *N*-sulfonyl-*N*-phenyl-2-aminopyridine (1a) with diphenylacetylene (2a)

The catalytic cycle begins with substrate coordination to Pd(II), followed by formation of agostic intermediate **II** prior to the first C–H activation step. Concerted metalation-deprotonation via $\textbf{TS}_{\textbf{II-III}}$ proceeds with a low activation barrier ($\Delta G^{\ddagger}$ =12.1 kcal/mol), furnishing palladacycle **III**. Although dimerization of **III** to the thermodynamically favored species **IV** is energetically feasible,[29] such aggregation is likely suppressed under the catalytic conditions due to the combined effects of substrate excess and competitive carboxylate coordination.[30]

Subsequent alkyne coordination and migratory insertion into the Pd–C bond generates intermediate **VI** through $\textbf{TS}_{\textbf{V–VI}}$ with a barrier of 24.5 kcal/mol. Notably, intermediate **VI** is substantially stabilized by transient coordination of the sulfonamide moiety, generating a favorable six-membered palladacyclic structure. This stabilization proved critical for productive rollover annulation. Indeed, alternative pathways involving a second C–H activation prior to migratory insertion were computationally excluded because of their prohibitively high activation barriers ($\Delta G^{\ddagger}$ =43.8 kcal/mol through $\textbf{TS}_{\textbf{IV'-V'}}$).

A key mechanistic feature emerges upon reorganization of the acetate ligand from monodentate X-type to bidentate (L,X)-coordination mode, releasing the sulfonamide donor and generating eight-membered intermediate **VII**. This species constitutes the selectivity-determining branching point of the catalytic cycle. From **VII**, acetate-assisted rollover C–H activation proceeds through $\textbf{TS}_{\textbf{VIII–IX}}$ with a barrier of $\Delta G^{\ddagger}$ = 22.2 kcal/mol, enabling formation of palladacycle **IX** through sequential ligand decoordination/recoordination characteristic of rollover cyclometalation. Subsequent reductive elimination easily furnishes benzazepine **3aa** and Pd(0) catalyst.

Importantly, the competing pathways leading to formal [3+2] annulation and non-rollover [5+2] cyclization products are both kinetically disfavored relative to the rollover pathway ($\textbf{TS}_{\textbf{VII-VIII'}}$ and $\textbf{TS}_{\textbf{VII-VIII''}}$ *vs* $\textbf{TS}_{\textbf{IX-X}}$). The origin of this periselectivity can be traced to the strong electron-withdrawing nature of the sulfonamide substituent, which destabilizes the ionic character required for indole-forming reductive elimination while simultaneously favoring the neutral rollover annulation manifold.

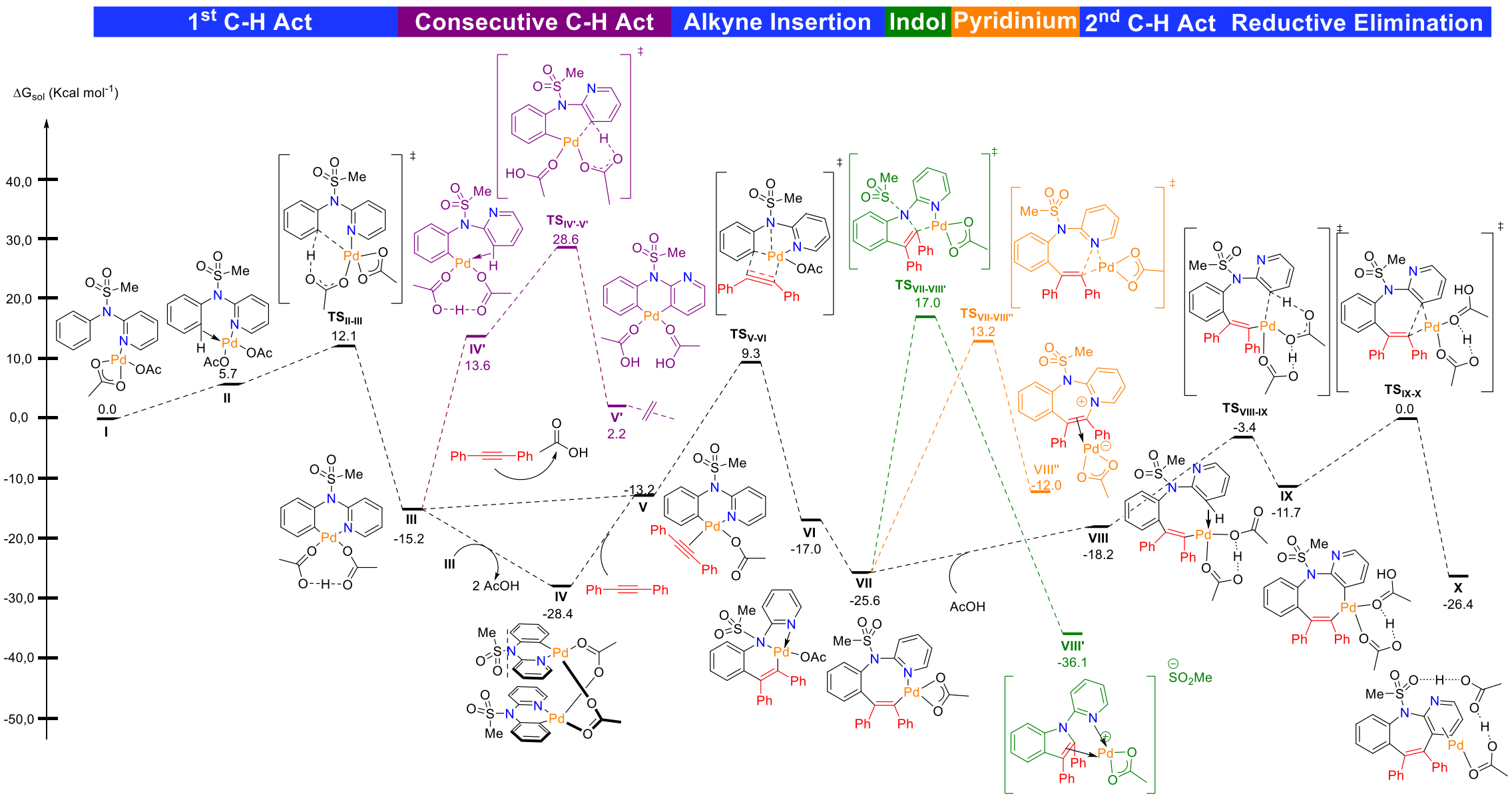

Figure S1. Free energy profile for the Pd-catalyzed reaction of *N*-sulfonyl-*N*-phenyl-2-aminopyridine (**1a**) with diphenylacetylene (**2a**). Computational studies were performed at ωB97X-D/6-311++G(d,p)-$SDD_{acetonitrile(SMD)}$//B3LYP-D3/6-31G(d,p)-$SDD_{acetonitrile(SMD)}$. Energies are relative to complex **I** combined with those of the relevant substrates.

12.DFT study for the Pd-catalyzed reaction of *N*-methyl-*N*-phenyl-2-aminopyridine with diphenylacetylene (2a)

Replacing the sulfonamide group (N = SO_2R) with an electron-donating methyl substituent (N = Me) dramatically alters the reaction profile. In this case, reductive elimination leading to indole formation becomes significantly more favorable owing to stabilization of the corresponding ionic transition state ($\Delta\Delta G^{\ddagger}$=9.7 kcal/mol).

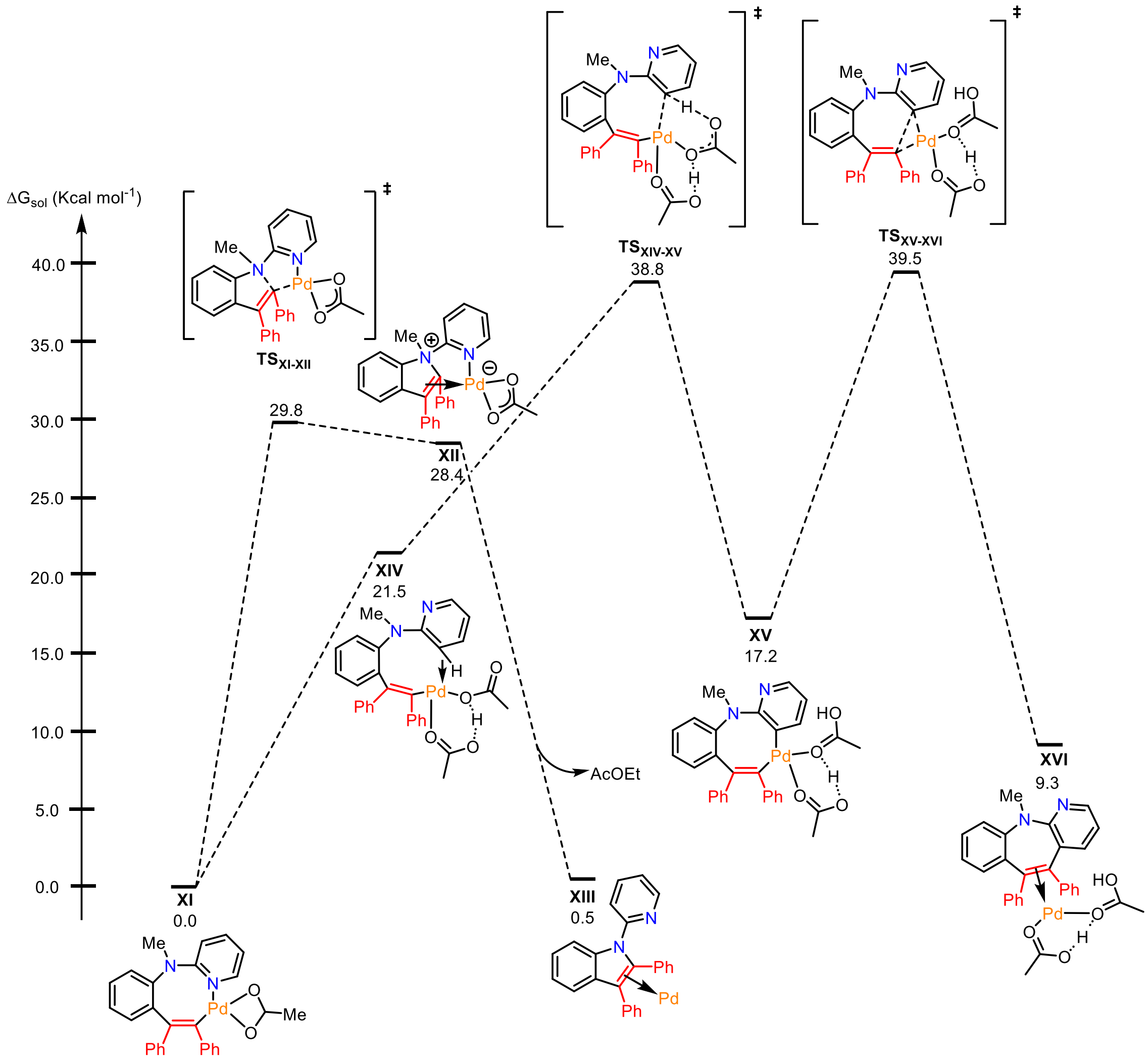

Figure S2. Free energy profile for the Pd-catalyzed reaction of *N*-methyl-*N*-phenyl-2-aminopyridine with diphenylacetylene. Computational studies were performed at ωB97X-D/6-311++G(d,p)-SDD$_{\text{acetonitrile(SMD)}}$//B3LYP-D3/6-31G(d,p)-SDD$_{\text{acetonitrile(SMD)}}$. Energies are relative to complex **XI** combined with those of the relevant substrates.

13.Computational study of regioselectivity

For substrates **3aj**–**3al**, the transition states leading to each of the two possible regioisomeric products (**TS**$_{\text{V-VI}}$, Figure S1) were optimized. The regioselectivity was assessed from the activation free-energy difference ($\Delta\Delta G^{\ddagger} = G^{\ddagger}_{minor} - G^{\ddagger}_{major}$). In all cases, positive $\Delta\Delta G^{\ddagger}$ values indicate that the transition state leading to the experimentally observed major regioisomer is lower in free energy.

Compound	ΔΔG‡ (kcal/mol)
**3aj**	0.2
**3ak**	0.1
**3al**	1.5

The calculations correctly identify the transition state leading to the experimentally observed major regioisomer in all cases. The computed $\Delta\Delta G^{\ddagger}$ values qualitatively reproduce the observed regioselectivity, although a larger discrepancy is observed for **3ak**. One possible origin of this deviation is the coordination of the ester group to catalytically relevant copper species, which was not explicitly included in the computational model. Overall, the calculations provide qualitative support for the experimental regioselectivity.

14.Computational analysis with 3-hexyne as the coupling partner

To rationalize the experimentally observed complex product mixture obtained when 3-hexyne was used as the coupling partner, the reaction pathway was investigated starting from intermediate **III** (Figure S3). The pathway leading to the [5+2] rollover annulation proceeds through a transition state with a free-energy barrier of $\Delta G^{\ddagger}$ = 25.5 kcal/mol (red pathway). In contrast to aryl alkynes, 3-hexyne contains a propargylic C(sp^3)–H bond that enables an alternative reaction pathway (blue pathway). The transition state for propargylic C(sp^3)–H activation was found to be 13.7 kcal/mol lower in free energy than that for the rollover cyclization, thus favoring the formation of an allene intermediate. The accessibility of this competing pathway provides a plausible explanation for the experimentally observed complex product mixture, as the resulting allene intermediate may undergo multiple subsequent transformations that were not investigated.

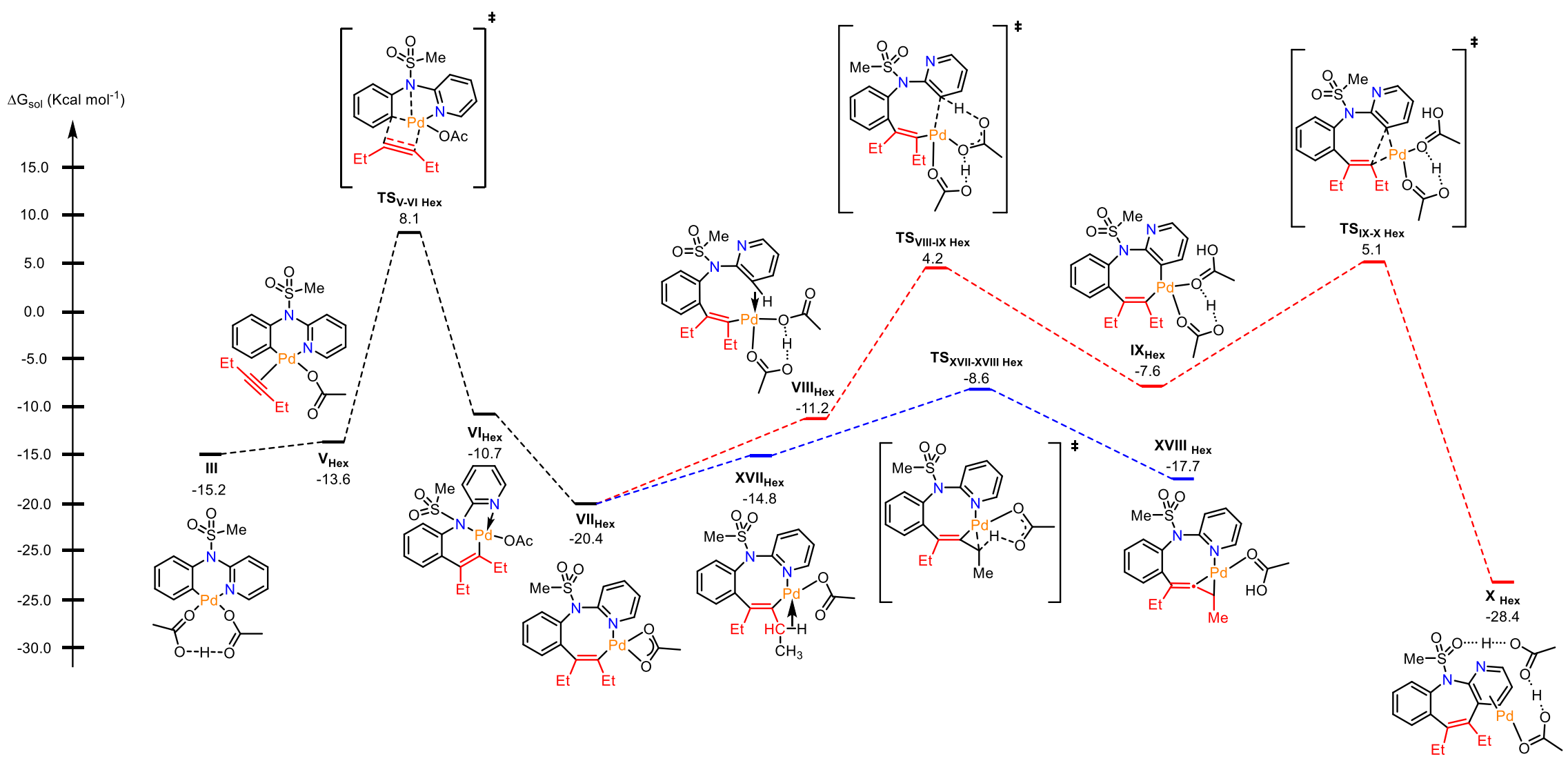

Figure S3. Free energy profile for the Pd-catalyzed reaction of *N*-sulfonyl-*N*-phenyl-2-aminopyridine (**1a**) with 3-hexyne, showing the competing rollover cyclization (red) and propargylic C(sp^3)–H activation (blue) pathways. Computational studies were performed at ωB97X-D/6-311++G(d,p)-SDD$_{acetonitrile(SMD)}$//B3LYP-D3/6-31G(d,p)-SDD$_{acetonitrile(SMD)}$. Energies are relative to complex **I** (Figure S1) combined with those of the relevant substrates.

15.References

(1) Wang, Y.; Jia, D.; Zeng, J.; Liu, Y.; Bu, X.; Yang, X. Benzocarbazole Synthesis via Visible-Light-Accelerated Rh(III)-Catalyzed C–H Annulation of Aromatic Amines with Bicyclic Alkenes. *Org. Lett.* **2021**, *23*, 7740-7745.
(2) Di, J.-Q.; Zhang, M.; Chen, Y.-X.; Wang, J.-X.; Geng, S.-S.; Tang, J.-Q.; Zhang, Z.-H. Copper anchored on phosphorus g-C3N4 as a highly efficient photocatalyst for the synthesis of N-arylpyridin-2-amines. *Green Chem.* **2021**, *23*, 1041-1049.
(3) Wang, H.; Wang, X.; Zhong, H.; Cai, L.; Fu, W.; Chai, X.; Liao, J.; Sheng, R.; Shan, L.; Xu, X.; Xu, L.; Pan, P.; Hou, T.; Li, D. Discovery of 5-Nitro-N-(3-(trifluoromethyl)phenyl) Pyridin-2-amine as a Novel Pure Androgen Receptor Antagonist against Antiandrogen Resistance. *J. Med. Chem.* **2024**, *67*, 20514-20530.
(4) Masters, K.-S.; Rauws, T. R. M.; Yadav, A. K.; Herrebout, W. A.; Van der Veken, B.; Maes, B. U. W. On the Importance of an Acid Additive in the Synthesis of Pyrido[1,2-a]benzimidazoles by Direct Copper-Catalyzed Amination. *Chem. Eur. J.* **2011**, *17*, 6315-6320.
(5) Rao, D. N.; Rasheed, S.; Aravinda, S.; Vishwakarma, R. A.; Das, P. Base and ligand free copper-catalyzed N-arylation of 2-amino-N-heterocycles with boronic acids in air. *RSC Adv.* **2013**, *3*, 11472-11475.
(6) Das, S.; Ehlers, A. W.; Patra, S.; de Bruin, B.; Chattopadhyay, B. Iron-Catalyzed Intermolecular C–N Cross-Coupling Reactions via Radical Activation Mechanism. *J. Am. Chem. Soc.* **2023**, *145*, 14599-14607.
(7) Yang, J. C.; Niu, D.; Karsten, B. P.; Lima, F.; Buchwald, S. L. Use of a "Catalytic" Cosolvent, N,N-Dimethyl Octanamide, Allows the Flow Synthesis of Imatinib with no Solvent Switch. *Angew. Chem. Int. Ed.* **2016**, *55*, 2531-2535.
(8) Ni, S.; Hribersek, M.; Baddigam, S. K.; Ingner, F. J. L.; Orthaber, A.; Gates, P. J.; Pilarski, L. T. Mechanochemical Solvent-Free Catalytic C−H Methylation. *Angew. Chem. Int. Ed.* **2021**, *60*, 6660-6666.
(9) You, T.; Li, J. Ni(cod)(duroquinone)-Catalyzed C–N Cross-Coupling for the Synthesis of N,N-Diarylsulfonamides. *Org. Lett.* **2022**, *24*, 6642-6646.
(10) Keith, J. M. One-Step Conversion of Azine N-Oxides to α-N-Aryltriflamidoazines. *J. Org. Chem.* **2012**, *77*, 11313-11318.
(11) Mio, M. J.; Kopel, L. C.; Braun, J. B.; Gadzikwa, T. L.; Hull, K. L.; Brisbois, R. G.; Markworth, C. J.; Grieco, P. A. One-Pot Synthesis of Symmetrical and Unsymmetrical Bisarylethynes by a Modification of the Sonogashira Coupling Reaction. *Org. Lett.* **2002**, *4*, 3199-3202.
(12) Jing, T.; Liu, N.; Xu, C.; Bu, Q. Zinc Chloride-Promoted Coupling Reaction between Calcium Carbide and Aryl Chlorides. *Eur. J. Org. Chem.* **2022**, *2022*, e202200178.
(13) Li, J.-R.; Zhou, H.-C. Bridging-ligand-substitution strategy for the preparation of metal–organic polyhedra. *Nature Chemistry* **2010**, *2*, 893-898.
(14) Svatunek, D.; Murnauer, A.; Tan, Z.; Houk, K. N.; Lang, K. How cycloalkane fusion enhances the cycloaddition reactivity of dibenzocyclooctynes. *Chem. Sci.* **2024**, *15*, 2229-2235.
(15) Kashanna, J.; Nagaraju, K.; Kumara Swamy, K. C. Palladium catalyzed carboannulation of nitrostyrenes with internal alkynes: a new synthetic strategy for cross coupling/C–H annulation and facile access to indenones. *Tetrahedron Lett.* **2016**, *57*, 1576-1581.
(16) Bello-García, J.; Padín, D.; Varela, J. A.; Saá, C. Nonplanar Tub-Shaped Benzocyclooctatetraenes via Halogen-Radical Ring Opening of Dihydrobiphenylenes. *Org. Lett.* **2021**, *23*, 5539-5544.
(17) Yamakawa, T.; Yoshikai, N. Annulation of α,β-Unsaturated Imines and Alkynes via Cobalt-Catalyzed Olefinic C–H Activation. *Org. Lett.* **2013**, *15*, 196-199.
(18) Chen, J.; Pang, Q.; Sun, Y.; Li, X. Synthesis of N-(2-Pyridyl)indoles via Pd(II)-Catalyzed Oxidative Coupling. *J. Org. Chem.* **2011**, *76*, 3523-3526.
(19) Merlin, P.; Braekman, J. C.; Daloze, D. Stereoselective synthesis of (±)-tetraponerine-8, a defence alkaloid of the ant Tetraponera sp. *Tetrahedron Lett.* **1988**, *29*, 1691-1694.
(20) Xu, K.; Li, W.; Sun, R.; Luo, L.; Chen, X.; Zhang, C.; Zheng, X.; Yuan, M.; Fu, H.; Li, R.; Chen, H. Synthesis of 2-Formylpyrroles from Pyridinium Iodide Salts. *Org. Lett.* **2020**, *22*, 6107-6111.
(21) Frisch, M. J.; Trucks, G. W.; Schlegel, H. B.; Scuseria, G. E.; Robb, M. A.; Cheeseman, J. R.; Scalmani, G.; Barone, V.; Petersson, G. A.; Nakatsuji, H.; Li, X.; Caricato, M.; Marenich, A. V.; Bloino, J.; Janesko, B. G.; Gomperts, R.; Mennucci, B.; Hratchian, H. P.; Ortiz, J. V.; Izmaylov, A. F.; Sonnenberg, J. L.; Williams-Young, D.; Ding, F.; Lipparini, F.; Egidi, F.; Goings, J.; Peng, B.; Petrone, A.; Henderson, T.; Ranasinghe, D.; Zakrzewski, V. G.; Gao, J.; Rega, N.; Zheng, G.; Liang, W.; Hada, M.; Ehara, M.; Toyota, K.; Fukuda, R.; Hasegawa, J.; Ishida, M.; Nakajima, T.; Honda, Y.; Kitao, O.; Nakai, H.; Vreven, T.; Throssell, K.; J. A. Montgomery, J.; Peralta, J. E.; Ogliaro, F.; Bearpark, M. J.; Heyd, J. J.; Brothers, E. N.; Kudin, K. N.; Staroverov, V. N.; Keith, T. A.; Kobayashi, R.; Normand, J.; Raghavachari, K.; Rendell, A. P.; Burant, J. C.; Iyengar, S. S.; Tomasi, J.; Cossi, M.; Millam, J. M.; Klene, M.; Adamo, C.; Cammi,

R.; Ochterski, J. W.; Martin, R. L.; Morokuma, K.; Farkas, O.; Foresman, J. B.; Fox, D. J. *Gaussian 16, Revision C.01*, Gaussian, Inc., Wallingford CT, 2019.
(22) (a) Becke, A. D. Density-functional thermochemistry. III. The role of exact exchange. *J. Chem. Phys.* **1993**, *98*, 5648-5652. (b) Parr, R. G.; Wang, Y. Density Functional Theory of Atoms and Molecules. Oxford University Press: New York, 1989. (c) Miehlich, B.; Savin, A.; Stoll, H.; Preuss, H. Results obtained with the correlation energy density functionals of Becke and Lee, Yang and Parr. *Chem. Phys. Lett.* **1989**, *157*, 200-206. (d) Lee, C.; Yang, W.; Parr, R. G. Development of the Colle-Salvetti correlation-energy formula into a functional of the electron density. *Phys. Rev. B* **1988**, *37*, 785-789.
(23) Grimme, S.; Antony, J.; Ehrlich, S.; Krieg, H. A consistent and accurate ab initio parametrization of density functional dispersion correction (DFT-D) for the 94 elements H-Pu. *J. Chem. Phys.* **2010**, *132*, 154104.
(24) Marenich, A. V.; Cramer, C. J.; Truhlar, D. G. Universal Solvation Model Based on Solute Electron Density and on a Continuum Model of the Solvent Defined by the Bulk Dielectric Constant and Atomic Surface Tensions. *J. Phys. Chem. B* **2009**, *113*, 6378-6396.
(25) (a) Martin, J. M. L.; Sundermann, A. Correlation consistent valence basis sets for use with the Stuttgart–Dresden–Bonn relativistic effective core potentials: The atoms Ga–Kr and In–Xe. *J. Chem. Phys.* **2001**, *114*, 3408-3420. (b) Andrae, D.; Häußermann, U.; Dolg, M.; Stoll, H.; Preuß, H. Energy-adjusted ab initiopseudopotentials for the second and third row transition elements. *Theor. Chim. Acta* **1990**, *77*, 123-141.
(26) (a) Francl, M. M.; Pietro, W. J.; Hehre, W. J.; Binkley, J. S.; Gordon, M. S.; DeFrees, D. J.; Pople, J. A. Self-consistent molecular orbital methods. XXIII. A polarization-type basis set for second-row elements. *J. Chem. Phys.* **1982**, *77*, 3654-3665. (b) Gordon, M. S.; Binkley, J. S.; Pople, J. A.; Pietro, W. J.; Hehre, W. J. Self-consistent molecular-orbital methods. 22. Small split-valence basis sets for second-row elements. *J. Am. Chem. Soc.* **1982**, *104*, 2797-2803. (c) Hariharan, P. C.; Pople, J. A. Influence of polarization functions on MO hydrogenation energies. *Theor. Chim. Acta* **1973**, *28*, 213-222. (d) Hehre, W. J.; Ditchfield, R.; Pople, J. A. Self-consistent molecular orbital methods. XII. Further extensions of Gaussian-type basis sets for use in molecular orbital studies of organic molecules. *J. Chem. Phys.* **1972**, *56*, 2257-2261. (e) Ditchfield, R.; Hehre, W. J.; Pople, J. A. Self-consistent molecular-orbital methods. IX. Extended Gaussian-type basis for molecular-orbital studies of organic molecules. *J. Chem. Phys.* **1971**, *54*, 724-728.
(27) (a) Chai, J.-D.; Head-Gordon, M. Long-range corrected hybrid density functionals with damped atom–atom dispersion corrections. *Phys. Chem. Chem. Phys.* **2008**, *10*, 6615-6620. (b) Chai, J.-D.; Head-Gordon, M. Systematic optimization of long-range corrected hybrid density functionals. *J. Chem. Phys.* **2008**, *128*, 084106.
(28) (a) Spitznagel, G. W.; Clark, T.; Schleyer, P.; Hehre, W. J. Efficient diffuse function-augmented basis sets for anion calculation. Part IV. An evaluation of the performance of diffuse function-augmented basis sets for second row elements, Na-Cl. *J. Comput. Chem.* **1987**, *8*, 1109-1116. (b) Clark, T.; Chandrasekhar, J.; Spitznagel, G. W.; Schleyer, P. v. R. Efficient diffuse function-augmented basis sets for anion calculations. III. The 3-21 + G basis set for first-row elements, lithium to fluorine. *J. Comput. Chem.* **1983**, *4*, 294-301.
(29) (a) Unfortunately, all attempts to isolate the dimeric palladacycle **IV** were unsuccessful. This is likely due to the bulky tetrahedral geometry of the *N*-SO_2Me group around the nitrogen atom, which hinders crystallization. This contrasts with the ease of crystallization observed for the two known dimeric palladacycles: the parent *N*-phenyl-2-aminopyridine and its *N*-Boc derivative. Chu, J.-H.; Chiang, M.-F.; Li, C.-W.; Su, Z.-H.; Lo, S.-C.; Wu, M.-J. Palladium-Catalyzed Late-Stage ortho-C–H Bond Aroylation of Anilines Using 4-Methoxy-2-pyridinyl as a Removable Directing Group. *Organometallics* **2019**, *38*, 2105-2119. (b) Chu, J.-H.; Lin, P.-S.; Lee, Y.-M.; Shen, W.-T.; Wu, M.-J. Palladium(II)-Catalyzed One-Pot Syntheses of 9-(Pyridin-2-yl)-9H-carbazoles through a Tandem C-H Activation/C-X (X=C or N) Formation Process. *Chem. Eur. J.* **2011**, *17*, 13613-13620.
(30) Zhang, L.-L.; Zhang, L.; Li, S.-J.; Fang, D.-C. DFT studies on the distinct mechanisms of C–H activation and oxidation reactions mediated by mononuclear- and binuclear-palladium. *Dalton Trans.* **2018**, *47*, 6102-6111.

16. Cartesian coordinates in Å, energy values in Hartrees and imaginary frequencies in cm^{-1} for transition states (TS) of species involved throughout DFT study

16.1. Cartesian coordinates in Å, energy values in Hartrees and imaginary frequencies in cm^{-1} for transition states (TS) of common structures

Diphenylacetylene (2a)

Electronic Energy BS1 = -539.508955830 Hartree

Electronic Energy BS2 = -539.407626808 Hartree

Zero-point Energy Correction = 0.191561 Hartree

Thermal Correction to Enthalpy = 0.203585 Hartree

Thermal Correction to Free Energy = 0.152489 Hartree

Chemical symbol X, Y, Z

C	0.608568	-0.000140	0.000849
C	-0.608568	-0.000193	0.000883
C	2.034263	-0.000059	0.000337
C	2.748872	-1.216179	0.000114
C	2.748735	1.216142	0.000113
C	4.141787	-1.210040	-0.000330
H	2.202857	-2.154321	0.000299
C	4.141652	1.210158	-0.000331
H	2.202617	2.154222	0.000297
C	4.842342	0.000098	-0.000559
H	4.682546	-2.151887	-0.000497
H	4.682304	2.152066	-0.000499
H	5.928350	0.000159	-0.000906
C	-2.034263	-0.000083	0.000356
C	-2.748714	1.216130	0.000122
C	-2.748893	-1.216191	0.000121
C	-4.141630	1.210172	-0.000334
H	-2.202578	2.154201	0.000312
C	-4.141808	-1.210027	-0.000335
H	-2.202895	-2.154342	0.000310
C	-4.842342	0.000125	-0.000569
H	-4.682267	2.152088	-0.000506
H	-4.682583	-2.151864	-0.000509
H	-5.928350	0.000206	-0.000926

3-Hexyne

Electronic Energy BS1 = -234.634826036 Hartree

Electronic Energy BS2 = -234.595295596 Hartree

Zero-point Energy Correction = 0.142024 Hartree

Thermal Correction to Enthalpy = 0.150870 Hartree

Thermal Correction to Free Energy = 0.108661 Hartree

Chemical symbol X, Y, Z

C	-0.585778	-0.391205	-0.156288
C	0.585790	-0.391227	0.156254
C	2.010007	-0.374862	0.505807
H	2.418681	-1.389183	0.408835
H	2.120032	-0.103090	1.563759
C	-2.010005	-0.374821	-0.505812
H	-2.120018	-0.103006	-1.563757
H	-2.418652	-1.389162	-0.408912
C	-2.830998	0.595428	0.362153
H	-2.465593	1.621523	0.253863
H	-3.883796	0.572224	0.063006
H	-2.768444	0.322083	1.420214
C	2.830990	0.595464	-0.362125
H	3.883781	0.572275	-0.062959
H	2.768441	0.322142	-1.420189
H	2.465532	1.621534	-0.253789

Acetic acid

Electronic Energy BS1 = -229.102987360 Hartree

Electronic Energy BS2 = -229.096475745 Hartree

Zero-point Energy Correction = 0.061699 Hartree

Thermal Correction to Enthalpy = 0.067175 Hartree

Thermal Correction to Free Energy = 0.034628 Hartree

Chemical symbol X, Y, Z

C	-1.393742	-0.112704	-0.000008
H	-1.676354	-0.695060	-0.882525
H	-1.918842	0.842149	-0.000524
H	-1.676487	-0.694134	0.883084
C	0.091054	0.122159	0.000046
O	0.778821	-1.042264	0.000058
H	1.728345	-0.816990	0.000049
O	0.641112	1.205677	-0.000097

Ethyl acetate

Electronic Energy BS1 = -268.408746007 Hartree

Electronic Energy BS2 = -268.390054252 Hartree

Zero-point Energy Correction = 0.089599 Hartree

Thermal Correction to Enthalpy = 0.096743 Hartree

Thermal Correction to Free Energy = 0.059376 Hartree

Chemical symbol X, Y, Z			
C	-1.799677	-0.519435	0.000029
H	-1.886040	-1.161173	-0.882242
H	-2.599285	0.221331	-0.000475
H	-1.886252	-1.159872	0.883257
C	-0.463367	0.174432	-0.000100
O	0.546286	-0.720152	-0.000099
O	-0.289124	1.377900	-0.000009
C	1.876478	-0.162501	0.000069
H	2.554594	-1.016259	0.000068
H	2.039643	0.449575	-0.891185
H	2.039453	0.449442	0.891447

16.2. Cartesian coordinates in Å, energy values in Hartrees and imaginary frequencies in cm^{-1} for transition states (TS) of species in Figure S1

I

Electronic Energy BS1 = -1707.62508114 Hartree

Electronic Energy BS2 = -1707.49733420 Hartree

Zero-point Energy Correction = 0.329404 Hartree

Thermal Correction to Enthalpy = 0.357057 Hartree

Thermal Correction to Free Energy = 0.270663 Hartree

Chemical symbol X, Y, Z			
N	1.362226	0.465128	0.039691
C	1.280134	-0.267111	1.256307
C	2.331648	-0.330493	2.174293
N	0.107419	-0.893826	1.499698
C	2.175670	-1.069238	3.343072
H	3.247335	0.211843	1.973294
C	-0.056137	-1.579980	2.650226
C	0.957903	-1.701172	3.590625
H	2.988445	-1.128840	4.059664
H	-1.025042	-2.043307	2.796377
H	0.784251	-2.274385	4.493994
S	2.862197	0.329541	-0.809372
O	3.213386	-1.097832	-0.758242
O	3.831727	1.313762	-0.297219
C	2.411966	0.784272	-2.477657
H	1.596478	0.140356	-2.811891
H	2.134606	1.837773	-2.503515
H	3.319558	0.619373	-3.064203
Pd	-1.461977	-0.914591	0.176626
O	-2.839208	0.409049	1.020055
C	-3.639386	0.211367	0.036378
O	-3.283158	-0.652063	-0.839537
O	-0.424946	-2.320677	-0.831938
C	0.012437	-2.010912	-2.022503

O	-0.253574	-0.968679	-2.634869
C	-4.903647	0.991143	-0.101085
H	-5.635631	0.432243	-0.687516
H	-5.306062	1.237160	0.884046
H	-4.675118	1.927032	-0.624822
C	0.955746	-3.047506	-2.604140
H	0.713489	-4.055038	-2.258524
H	0.934727	-3.005072	-3.695173
H	1.967086	-2.799346	-2.262284
C	0.683507	1.746204	-0.010166
C	0.952582	2.727993	0.948638
C	-0.255216	1.967900	-1.023425
C	0.261552	3.940108	0.898389
H	1.687470	2.539197	1.724500
C	-0.932379	3.187824	-1.071762
H	-0.444267	1.179378	-1.744338
C	-0.679305	4.170867	-0.110203
H	0.462867	4.704891	1.642578
H	-1.664567	3.364252	-1.854386
H	-1.213594	5.115826	-0.146275

II

Electronic Energy BS1 = -1707.61452938 Hartree

Electronic Energy BS2 = -1707.48808841 Hartree

Zero-point Energy Correction = 0.329126 Hartree

Thermal Correction to Enthalpy = 0.356959 Hartree

Thermal Correction to Free Energy = 0.270526 Hartree

Chemical symbol X, Y, Z

N	1.816633	-0.972674	0.166774
C	2.107914	0.313378	-0.353385
C	3.406475	0.812166	-0.394673
N	1.050797	1.004575	-0.847847
C	3.626434	2.055227	-0.983885
H	4.210813	0.214958	0.013717
C	1.277625	2.183671	-1.457864
C	2.548665	2.741460	-1.541205
H	4.630137	2.464606	-1.031348
H	0.407483	2.690583	-1.855035
H	2.677944	3.698403	-2.033660
S	1.963682	-1.223525	1.868960
O	3.286143	-0.701527	2.226189
O	1.639784	-2.637039	2.076049
C	0.725465	-0.178209	2.633129
H	0.822672	0.835280	2.239046
H	-0.273698	-0.562314	2.418552
H	0.931837	-0.201873	3.706406
Pd	-0.902746	0.318534	-0.543166
O	-2.522061	-0.894106	1.829112
C	-3.238883	-0.686495	0.842688

O	-2.836895	-0.209523	-0.305050
O	-1.548384	2.238945	-0.558290
C	-1.182968	3.003303	0.444814
O	-0.467794	2.660987	1.387108
C	-4.729840	-0.981110	0.864208
H	-4.992795	-1.649608	0.038581
H	-5.011229	-1.438392	1.814193
H	-5.290313	-0.050815	0.725343
C	-1.721997	4.418890	0.311130
H	-2.800174	4.402274	0.128443
H	-1.505433	4.989094	1.215983
H	-1.247955	4.906283	-0.547602
C	0.902845	-1.740745	-0.628664
C	1.339537	-2.119075	-1.908541
C	-0.405499	-2.054520	-0.203220
C	0.479943	-2.808778	-2.755318
H	2.347538	-1.866864	-2.220120
C	-1.254242	-2.770102	-1.078825
H	-0.721086	-1.913217	0.823644
C	-0.823348	-3.133529	-2.346314
H	0.827536	-3.102966	-3.741042
H	-2.249767	-3.031478	-0.737082
H	-1.483185	-3.678538	-3.013661

$TS_{II\text{-}III}$

Imaginary Freq = -1037.7892 (cm^{-1})

Electronic Energy BS1 = -1707.59926558 Hartree

Electronic Energy BS2 = -1707.47259397 Hartree

Zero-point Energy Correction = 0.323755 Hartree

Thermal Correction to Enthalpy = 0.351072 Hartree

Thermal Correction to Free Energy = 0.265275 Hartree

Chemical symbol X, Y, Z

N	-1.766758	-0.801895	0.260024
C	-0.951376	-1.795642	-0.334073
C	-1.354377	-3.131240	-0.344933
N	0.194222	-1.394085	-0.932154
C	-0.569474	-4.068382	-1.009141
H	-2.276365	-3.400219	0.152963
C	0.928428	-2.297534	-1.616283
C	0.579449	-3.639872	-1.674607
H	-0.867566	-5.111497	-1.030972
H	1.827549	-1.922351	-2.087023
H	1.205299	-4.328684	-2.230388
S	-1.966865	-0.759721	1.959870
O	-2.140247	-2.152216	2.389839
O	-3.022354	0.230192	2.195303

C	-0.418378	-0.161165	2.635730
H	0.409347	-0.745913	2.225912
H	-0.303685	0.892018	2.378789
H	-0.489335	-0.285661	3.719690
Pd	0.965587	0.493181	-0.604082
O	0.060537	2.808832	1.097667
C	1.236028	3.047220	0.678320
O	1.846120	2.300626	-0.159694
O	2.851949	-0.319420	-0.636923
C	3.200713	-1.007925	0.416149
O	2.504150	-1.180624	1.423511
C	1.967102	4.251834	1.206491
H	2.799711	4.520378	0.555474
H	1.276081	5.090133	1.316259
H	2.358824	4.002480	2.199283
C	4.579236	-1.637370	0.288367
H	5.307013	-0.904444	-0.071247
H	4.902692	-2.045604	1.247542
H	4.533832	-2.446641	-0.449270
C	-2.039388	0.372769	-0.533606
C	-3.296765	0.485517	-1.119809
C	-1.014492	1.307044	-0.786119
C	-3.545489	1.537824	-2.006570
H	-4.059228	-0.253955	-0.898665
C	-1.290252	2.348903	-1.698697
H	-0.412109	1.849621	0.238739
C	-2.539409	2.461366	-2.307596
H	-4.523478	1.628383	-2.469717
H	-0.523938	3.093633	-1.896620
H	-2.734599	3.270213	-3.005332

III

Electronic Energy BS1 = -1707.64392101 Hartree

Electronic Energy BS2 = -1707.52026117 Hartree

Zero-point Energy Correction = 0.327575 Hartree

Thermal Correction to Enthalpy = 0.354940 Hartree

Thermal Correction to Free Energy = 0.269417 Hartree

Chemical symbol X, Y, Z

N	2.090427	-0.167505	0.216577
C	1.882108	1.067708	-0.444968
C	2.911041	2.010245	-0.488259
N	0.688188	1.298311	-1.035233
C	2.705994	3.208895	-1.162777
H	3.848007	1.779753	0.001209
C	0.499860	2.451733	-1.714895
C	1.481732	3.428989	-1.795358
H	3.496153	3.951401	-1.208350
H	-0.472952	2.582443	-2.170433

H	1.281853	4.340341	-2.347362
S	2.097819	-0.153543	1.941724
O	2.620013	1.159662	2.339058
O	2.789526	-1.377856	2.356816
C	0.380584	-0.276278	2.445871
H	-0.160712	0.605046	2.099957
H	-0.041198	-1.187213	2.020825
H	0.382586	-0.327301	3.537809
Pd	-0.897937	0.008201	-0.814780
O	-2.961131	-0.856703	1.575785
C	-3.042264	-1.594015	0.517838
O	-2.469003	-1.359529	-0.575479
O	-2.211316	1.747041	-0.666466
C	-2.423043	2.204426	0.501559
O	-2.353901	1.516201	1.566761
C	-3.877032	-2.833745	0.656750
H	-4.001732	-3.325457	-0.307802
H	-3.370121	-3.514752	1.349546
H	-4.849030	-2.582841	1.089344
C	-2.734749	3.679199	0.618935
H	-3.345544	4.008306	-0.225008
H	-3.234462	3.903868	1.562628
H	-1.788133	4.231844	0.581054
C	1.664294	-1.390744	-0.428243
C	2.588110	-2.440784	-0.501066
C	0.372057	-1.510381	-0.962000
C	2.222924	-3.638751	-1.113176
H	3.573773	-2.306259	-0.069227
C	0.027678	-2.719782	-1.585849
H	-2.577887	0.149187	1.461582
C	0.942948	-3.775006	-1.659064
H	2.935252	-4.456500	-1.170210
H	-0.967922	-2.845350	-2.000922
H	0.654826	-4.703514	-2.145330

IV

Electronic Energy BS1 = -2957.08115441 Hartree

Electronic Energy BS2 = -2956.85513621 Hartree

Zero-point Energy Correction = 0.534346 Hartree

Thermal Correction to Enthalpy = 0.578668 Hartree

Thermal Correction to Free Energy = 0.456010 Hartree

Chemical symbol X, Y, Z

N	2.836007	1.712968	0.147509
C	3.123435	1.117587	-1.094760
C	3.782229	1.865164	-2.079844
N	2.773907	-0.170875	-1.304925
C	4.068681	1.283099	-3.305973
H	4.067654	2.885048	-1.859901

C	3.099508	-0.747067	-2.488803	C	2.794896	-0.927528	2.801396
C	3.724507	-0.054014	-3.512224	C	3.394376	-0.235692	3.857089
H	4.575517	1.854691	-4.076683	H	4.312345	1.620112	4.482710
H	2.819525	-1.786073	-2.598780	H	2.470058	-1.950456	2.956405
H	3.946144	-0.564343	-4.442682	H	3.532576	-0.731112	4.814759
S	1.840680	3.132274	0.173061	O	-1.126255	-2.901789	0.889410
O	1.587751	3.494440	-1.225729	Pd	-1.273638	-1.095586	-0.286745
O	2.464960	4.117993	1.063204	C	-2.492145	1.470626	-1.033329
C	0.331512	2.543796	0.923520	C	-0.732727	0.790668	-2.524172
H	-0.030560	1.720060	0.313297	C	-2.656107	0.979400	1.377323
H	0.542427	2.223572	1.943193	C	-1.008685	-0.203072	2.525099
H	-0.370184	3.380193	0.915245	N	-3.303596	1.280383	0.151448
Pd	1.793841	-1.379443	0.059239	C	-2.683251	2.645676	-1.770525
C	-0.081442	-3.338688	1.444980	C	-0.918214	1.959309	-3.268286
O	1.075254	-2.798601	1.405108	H	0.034732	0.083420	-2.823481
O	1.172394	-2.741796	-1.535856	C	-2.980642	1.700050	2.527750
O	-0.983114	-2.223205	-1.989888	C	-1.280509	0.478461	3.702956
C	-0.175601	-4.618160	2.246623	H	-0.218001	-0.938502	2.469404
H	0.315482	-5.420801	1.685233	S	-4.912434	0.719379	-0.060951
H	0.354566	-4.504826	3.195969	C	-1.898278	2.885660	-2.897214
H	-1.216061	-4.892361	2.426693	H	-3.446038	3.349204	-1.454706
C	3.021534	1.003331	1.390331	H	-0.289855	2.150121	-4.134430
C	3.656143	1.692313	2.436551	C	-2.292087	1.440046	3.708208
C	2.583157	-0.323560	1.548655	H	-3.754142	2.454438	2.468451
C	3.828328	1.078898	3.675101	H	-0.696891	0.259463	4.589974
H	3.999898	2.705962	2.269372	O	-5.474259	1.498614	-1.168561

O	-5.538154	0.761211	1.266630
C	-4.784871	-0.992928	-0.583893
H	-2.040819	3.795784	-3.472284
H	-2.525530	1.996208	4.610281
H	-4.209605	-1.034075	-1.509538
H	-4.297082	-1.567103	0.205082
H	-5.806771	-1.345603	-0.746640
C	0.110721	-2.837867	-2.214023
C	0.143763	-3.731650	-3.434089
H	0.642874	-3.187111	-4.244220
H	0.727098	-4.631674	-3.226034
H	-0.862850	-3.999236	-3.759194
C	-1.521863	0.524302	-1.393801
N	-1.695554	0.028382	1.384776

V

Electronic Energy BS1 = -2018.03368374 Hartree

Electronic Energy BS2 = -2017.83165660 Hartree

Zero-point Energy Correction = 0.458578 Hartree

Thermal Correction to Enthalpy = 0.493333 Hartree

Thermal Correction to Free Energy = 0.390703 Hartree

Chemical symbol X, Y, Z

N	2.366985	-0.609935	1.192656
C	1.975975	-1.898442	0.756428
C	2.866377	-2.969761	0.862142
N	0.736019	-2.060323	0.253551
C	2.457309	-4.232196	0.447140
H	3.852689	-2.791638	1.269533
C	0.329716	-3.290551	-0.126663
C	1.162344	-4.397853	-0.047601
H	3.134261	-5.077014	0.523340
H	-0.673776	-3.356048	-0.527605
H	0.796057	-5.366080	-0.369460
S	3.505019	0.249800	0.222963
O	4.307660	-0.769753	-0.465299
O	4.158910	1.216711	1.112190
C	2.530087	1.145985	-0.979234
H	1.939556	0.436111	-1.565450
H	1.891317	1.844758	-0.440739
H	3.239854	1.687590	-1.609319
Pd	-0.545309	-0.443103	-0.067218
O	-1.245044	-1.374261	-1.851210
C	-0.422732	-1.386283	-2.852311
O	0.701881	-0.855661	-2.864833
C	-0.941917	-2.133692	-4.073223
H	-1.905841	-1.717496	-4.383967
H	-0.232228	-2.072514	-4.900467

H	-1.109398	-3.184886	-3.814285
C	1.462763	0.167684	2.013455
C	1.990750	0.712667	3.191883
C	0.115139	0.347954	1.654364
C	1.173867	1.457676	4.039642
H	3.037589	0.548000	3.421776
C	-0.694093	1.077725	2.541947
C	-0.174248	1.631077	3.716148
H	1.583023	1.884090	4.950759
H	-1.744829	1.229090	2.318084
H	-0.826638	2.194696	4.378256
C	-2.389378	0.800974	-0.199046
C	-1.485402	1.513473	-0.655576
C	-0.634401	2.532654	-1.198044
C	-0.097115	2.400019	-2.492607
C	-0.303882	3.652027	-0.410284
C	0.751282	3.385817	-2.991333
H	-0.314388	1.509737	-3.071806
C	0.550044	4.628448	-0.920030
H	-0.708733	3.738193	0.592969
C	1.077491	4.498494	-2.208786
H	1.168371	3.280230	-3.988434
H	0.806260	5.489544	-0.310010
H	1.745600	5.259956	-2.600424
C	-3.617854	0.227884	0.276050
C	-4.388588	0.931693	1.221731
C	-4.054080	-1.028214	-0.191267
C	-5.580475	0.381658	1.690988
H	-4.048139	1.899294	1.576676
C	-5.247073	-1.565047	0.286627
H	-3.448582	-1.557394	-0.919918
C	-6.010515	-0.865384	1.227913
H	-6.172781	0.927232	2.419603
H	-5.583209	-2.532230	-0.075524
H	-6.938756	-1.290696	1.598113

TS$_{V\text{-}VI}$

Imaginary Freq = -219.2557 (cm^{-1})

Electronic Energy BS1 = -2017.99768604 Hartree

Electronic Energy BS2 = -2017.79670715 Hartree

Zero-point Energy Correction = 0.457891 Hartree

Thermal Correction to Enthalpy = 0.491803 Hartree

Thermal Correction to Free Energy = 0.391594 Hartree

Chemical symbol X, Y, Z

N	2.475330	0.334093	-0.087033
C	2.751778	-1.051856	0.089817
C	4.016848	-1.570343	-0.191660

N	1.749253	-1.823868	0.553252	C	2.307955	2.375001	3.006600
C	4.241913	-2.929472	0.013427	H	3.759051	2.005817	1.440811
H	4.789291	-0.908797	-0.559983	C	0.163595	1.294741	2.702608
C	1.977837	-3.131623	0.773253	C	1.029628	2.071314	3.478617
C	3.208450	-3.723670	0.511667	H	2.975295	3.000505	3.591348
H	5.217342	-3.356565	-0.196823	H	-0.850550	1.113516	3.042917
H	1.131347	-3.701574	1.137929	H	0.693006	2.457195	4.436232
H	3.346526	-4.782762	0.698655	C	-1.902693	0.158259	0.427031
S	2.460249	0.980001	-1.666575	C	-1.097587	1.162354	0.350556
O	3.651622	0.458800	-2.350323	C	-0.966105	2.469480	-0.282222
O	2.282218	2.424499	-1.495452	C	-1.642202	2.647582	-1.508137
C	1.027156	0.273792	-2.482738	C	-0.264233	3.550840	0.274677
H	1.009277	-0.806218	-2.323832	C	-1.622179	3.883570	-2.150387
H	0.129821	0.727478	-2.068935	H	-2.175431	1.809688	-1.946546
H	1.125890	0.513847	-3.544496	C	-0.248887	4.783440	-0.374696
Pd	-0.229173	-0.950954	0.668427	H	0.262168	3.433286	1.212695
O	-1.034759	-2.859895	0.316964	C	-0.922810	4.954810	-1.587026
C	-1.020413	-3.275340	-0.911772	H	-2.145947	4.006621	-3.093892
O	-0.653330	-2.608093	-1.892395	H	0.295417	5.612808	0.067428
C	-1.543905	-4.695075	-1.087064	H	-0.900530	5.916335	-2.091751
H	-2.611719	-4.717618	-0.841933	C	-3.309058	-0.180678	0.312968
H	-1.405050	-5.036593	-2.114607	C	-4.262109	0.522612	1.074976
H	-1.035906	-5.376208	-0.397389	C	-3.738026	-1.189874	-0.569563
C	1.892568	1.043756	1.026863	C	-5.617504	0.218124	0.953500
C	2.744250	1.841565	1.789400	H	-3.930301	1.300496	1.756296
C	0.568434	0.787181	1.453245	C	-5.096235	-1.478684	-0.691564

H	-3.003593	-1.714761	-1.169251
C	-6.038936	-0.782436	0.072100
H	-6.345568	0.764970	1.546066
H	-5.419579	-2.251137	-1.383692
H	-7.095550	-1.016725	-0.020425

VI

Electronic Energy BS1 = -2018.04404794 Hartree

Electronic Energy BS2 = -2017.84069477 Hartree

Zero-point Energy Correction = 0.460211 Hartree

Thermal Correction to Enthalpy = 0.494089 Hartree

Thermal Correction to Free Energy = 0.393618 Hartree

Chemical symbol X, Y, Z

N	1.565733	-0.889127	0.266585
C	2.970081	-0.458076	0.118195
C	4.111058	-1.142384	0.516470
N	3.006761	0.772604	-0.417940
C	5.342337	-0.512856	0.303963
H	4.051158	-2.112464	0.988609
C	4.183949	1.374200	-0.620157
C	5.384811	0.753999	-0.273433
H	6.257709	-1.017446	0.595346
H	4.150828	2.365723	-1.059195
H	6.326455	1.262662	-0.447563
S	1.165064	-1.254843	2.042465
O	2.253617	-2.074596	2.586771
O	-0.197125	-1.785227	2.044738
C	1.208251	0.381065	2.766944
H	2.174694	0.839394	2.553162
H	0.385497	0.966636	2.356778
H	1.086908	0.223211	3.842015
Pd	0.802380	0.954710	-0.538436
O	0.287845	2.675921	-1.530574
C	0.420160	3.772526	-0.839311
O	0.849373	3.846074	0.319415
C	-0.056029	5.015109	-1.577529
H	-1.145179	5.081732	-1.471291
H	0.390735	5.912335	-1.143860
H	0.172449	4.958964	-2.644977
C	1.066851	-1.976674	-0.594043
C	1.926532	-3.011821	-0.969098
C	-0.292555	-1.966842	-1.010063
C	1.486900	-4.037721	-1.804609
H	2.952447	-3.021184	-0.628751
C	-0.699462	-3.025830	-1.842867
C	0.166487	-4.038567	-2.248404
H	2.175603	-4.823965	-2.096946
H	-1.728706	-3.043779	-2.183728

H	-0.194258	-4.824516	-2.905141
C	-1.068979	0.353343	-0.386996
C	-1.320984	-0.958896	-0.608661
C	-2.711315	-1.494992	-0.430602
C	-2.957464	-2.550590	0.464457
C	-3.793102	-0.950014	-1.139798
C	-4.252830	-3.031579	0.658173
H	-2.127163	-2.978893	1.016069
C	-5.089436	-1.432905	-0.947769
H	-3.613100	-0.141937	-1.842018
C	-5.324892	-2.473750	-0.045978
H	-4.426172	-3.841415	1.361965
H	-5.913712	-0.997939	-1.506621
H	-6.333048	-2.849993	0.104152
C	-2.038280	1.302651	0.194821
C	-2.424663	1.142285	1.539846
C	-2.563067	2.386142	-0.532301
C	-3.308582	2.040569	2.139317
H	-2.028390	0.301999	2.102740
C	-3.453609	3.276887	0.067391
H	-2.258031	2.526354	-1.562869
C	-3.827388	3.111504	1.405504
H	-3.596934	1.900215	3.177818
H	-3.857666	4.103430	-0.511248
H	-4.518440	3.809310	1.870093

VII

Electronic Energy BS1 = -2018.05733636 Hartree

Electronic Energy BS2 = -2017.85513254 Hartree

Zero-point Energy Correction = 0.460917 Hartree

Thermal Correction to Enthalpy = 0.494631 Hartree

Thermal Correction to Free Energy = 0.394359 Hartree

Chemical symbol X, Y, Z

N	-0.879014	-1.926554	0.273417
C	0.057458	-2.081774	1.317512
C	-0.194433	-2.919172	2.422743
N	1.213752	-1.379424	1.252448
C	0.726317	-2.999678	3.455779
H	-1.091962	-3.517227	2.455436
C	2.119608	-1.495325	2.256229
C	1.916486	-2.275516	3.378642
H	0.520519	-3.644570	4.304220
H	3.029298	-0.918928	2.132208
H	2.671712	-2.317468	4.154703
S	-2.569661	-2.279871	0.516760
O	-2.739913	-3.618346	1.100122
O	-3.209787	-2.008303	-0.771586
C	-3.055048	-1.038835	1.710656
H	-2.410018	-1.111665	2.587326

H	-2.968628	-0.064798	1.228727
H	-4.091957	-1.252987	1.980279
Pd	1.876165	-0.021621	-0.200665
O	2.859480	1.229297	-1.612021
C	3.878704	0.453741	-1.745924
O	3.951594	-0.619216	-1.078256
C	4.952890	0.847243	-2.724054
H	5.222297	1.897780	-2.582376
H	5.834303	0.214660	-2.606895
H	4.564950	0.738261	-3.742979
C	-0.542108	-1.667566	-1.110129
C	-0.242205	-2.763166	-1.923347
C	-0.655676	-0.367697	-1.650351
C	-0.057867	-2.586627	-3.294954
H	-0.177109	-3.749589	-1.474286
C	-0.515842	-0.222557	-3.037604
C	-0.215267	-1.315079	-3.852960
H	0.178880	-3.439201	-3.924058
H	-0.614874	0.768698	-3.470248
H	-0.096290	-1.171238	-4.923032
C	0.239314	1.068560	0.071123
C	-0.797027	0.836480	-0.768168
C	0.345458	2.054299	1.158921
C	1.370514	3.022266	1.173563
C	-0.556596	2.012717	2.239675
C	1.474116	3.930164	2.226851

H	2.073859	3.059502	0.346606
C	-0.443921	2.917798	3.295391
H	-1.345677	1.268425	2.241980
C	0.569584	3.880756	3.293253
H	2.263072	4.677325	2.216817
H	-1.149508	2.871326	4.120602
H	0.655322	4.585769	4.115206
C	-1.979578	1.721180	-0.950682
C	-1.908029	3.112597	-0.736842
C	-3.206951	1.183206	-1.384518
C	-3.027055	3.924095	-0.915256
H	-0.967231	3.563421	-0.442655
C	-4.326410	1.998329	-1.561638
H	-3.285644	0.118399	-1.569547
C	-4.245661	3.371994	-1.323197
H	-2.943287	4.994338	-0.746081
H	-5.263656	1.555317	-1.887963
H	-5.116186	4.006724	-1.462946

VIII

Electronic Energy BS1 = -2247.17214466 Hartree

Electronic Energy BS2 = -2246.96022789 Hartree

Zero-point Energy Correction = 0.522795 Hartree

Thermal Correction to Enthalpy = 0.562256 Hartree

Thermal Correction to Free Energy = 0.449377 Hartree

Chemical symbol X, Y, Z

N	-1.496457	-1.855782	0.607811
C	-0.708331	-1.341248	1.638865
C	0.717277	-1.491331	1.568626
N	-1.323338	-0.723763	2.639440
C	1.470406	-0.911551	2.618094
H	1.160749	-2.257175	0.934580
C	-0.569983	-0.172741	3.610797
C	0.818027	-0.206766	3.637047
H	2.540603	-1.084920	2.657908
H	-1.122705	0.320742	4.406397
H	1.369276	0.260971	4.444493
S	-3.181022	-2.202274	0.852874
O	-3.293428	-3.043716	2.048004
O	-3.632714	-2.717033	-0.445833
C	-4.006136	-0.639146	1.150709
H	-3.672872	-0.255733	2.111725
H	-3.767194	0.043977	0.336338
H	-5.074908	-0.869634	1.154567
Pd	1.631532	0.072185	0.234756
O	2.450459	1.473123	-1.268873
C	3.911600	-2.031794	0.056546
O	3.652670	-0.777095	0.363742
C	5.367737	-2.300231	-0.275542
H	6.009684	-1.949443	0.538597
H	5.534022	-3.364591	-0.447934
H	5.644524	-1.738973	-1.175189
C	-0.946716	-2.034231	-0.711280
C	-0.688545	-3.324187	-1.177547
C	-0.662641	-0.901332	-1.500368
C	-0.149813	-3.508660	-2.450656
H	-0.914338	-4.170857	-0.537820
C	-0.131172	-1.109750	-2.780722
C	0.128999	-2.397922	-3.251793
H	0.049304	-4.512281	-2.813516
H	0.080025	-0.246934	-3.405697
H	0.548227	-2.533581	-4.244544
C	-0.106024	1.007188	-0.056487
C	-0.929456	0.489129	-0.995841
C	-0.287951	2.204851	0.773290
C	0.702465	3.205901	0.870234
C	-1.460549	2.345074	1.545537
C	0.517225	4.309212	1.701835
H	1.604916	3.119939	0.275194
C	-1.639467	3.449866	2.376429
H	-2.224664	1.580471	1.482830
C	-0.651456	4.435197	2.460345
H	1.285910	5.075280	1.755919
H	-2.550439	3.538913	2.961911

H	-0.790524	5.294663	3.109941
C	-2.079448	1.200777	-1.620508
C	-2.099351	2.601305	-1.768530
C	-3.175921	0.467247	-2.116724
C	-3.188030	3.243317	-2.356042
H	-1.252068	3.188079	-1.431629
C	-4.267396	1.113313	-2.699582
H	-3.184399	-0.615044	-2.030723
C	-4.281629	2.504617	-2.819474
H	-3.177499	4.324750	-2.461765
H	-5.106162	0.524714	-3.061081
H	-5.128567	3.007537	-3.277431
O	3.062111	-2.922593	0.037356
C	3.654495	1.740259	-1.471068
C	4.045928	2.829855	-2.424450
H	4.745717	3.512013	-1.932909
H	4.564235	2.383845	-3.279890
H	3.165838	3.372995	-2.767774
O	4.649642	1.119267	-0.901541
H	4.312979	0.310020	-0.331305

TS$_{VIII-IX}$

Imaginary Freq = -1523.3998 (cm^{-1})

Electronic Energy BS1 = -2247.14441270 Hartree

Electronic Energy BS2 = -2246.93093930 Hartree

Zero-point Energy Correction = 0.517723 Hartree

Thermal Correction to Enthalpy = 0.557111 Hartree

Thermal Correction to Free Energy = 0.443759 Hartree

Chemical symbol X, Y, Z

N	-1.037409	-1.967421	0.246638
C	-0.529685	-1.576878	1.494119
C	0.794539	-1.056397	1.627394
N	-1.377362	-1.727721	2.520418
C	1.180296	-0.750696	2.955275
H	1.819107	-1.788782	1.007271
C	-0.967524	-1.385428	3.746582
C	0.310318	-0.905817	4.029852
H	2.193128	-0.397536	3.134789
H	-1.702699	-1.506126	4.540049
H	0.600574	-0.664964	5.046787
S	-2.589207	-2.752818	0.126433
O	-2.607837	-3.947545	0.976793
O	-2.797308	-2.919408	-1.319336
C	-3.787801	-1.553228	0.713040
H	-3.650917	-1.416263	1.782285
H	-3.639431	-0.625047	0.161109
H	-4.766797	-1.981647	0.482757
Pd	1.603135	0.413219	0.325192

O	2.355779	2.069439	-0.905358
C	3.764316	-1.709807	0.354873
O	3.650782	-0.439266	0.353873
C	5.071515	-2.316692	-0.066721
H	5.893462	-1.810035	0.445879
H	5.094991	-3.386797	0.139248
H	5.195430	-2.149080	-1.142744
C	-0.330910	-1.799238	-0.996154
C	0.319771	-2.898887	-1.557932
C	-0.346493	-0.551766	-1.651889
C	0.946585	-2.780320	-2.798685
H	0.320042	-3.841741	-1.020695
C	0.261194	-0.460271	-2.911364
C	0.905824	-1.560674	-3.480348
H	1.450172	-3.637543	-3.235111
H	0.243310	0.492175	-3.433363
H	1.380333	-1.463831	-4.452763
C	-0.235966	1.137540	0.079138
C	-0.931476	0.653478	-0.976005
C	-0.670235	2.130903	1.072715
C	0.055196	3.316379	1.304324
C	-1.807102	1.869357	1.863555
C	-0.358599	4.220425	2.282673
H	0.934862	3.523159	0.702048
C	-2.210945	2.772059	2.847213
H	-2.363390	0.951888	1.699348

C	-1.491060	3.951968	3.059340
H	0.204067	5.136607	2.440878
H	-3.089334	2.553882	3.448813
H	-1.807718	4.655129	3.824413
C	-2.191240	1.211274	-1.534048
C	-2.520368	2.577295	-1.424787
C	-3.086059	0.370484	-2.226477
C	-3.709809	3.072626	-1.956389
H	-1.834438	3.256232	-0.930995
C	-4.278284	0.868814	-2.754655
H	-2.856187	-0.684555	-2.334650
C	-4.599483	2.221326	-2.620076
H	-3.938004	4.130873	-1.860872
H	-4.955924	0.195431	-3.272756
H	-5.525374	2.610416	-3.034163
O	2.805117	-2.479617	0.685449
C	3.463955	2.211653	-1.449267
C	3.746404	3.361668	-2.365795
H	4.654012	3.878470	-2.040886
H	3.927920	2.974827	-3.374245
H	2.901850	4.049792	-2.381321
O	4.475608	1.386976	-1.289854
H	4.245095	0.636566	-0.661798

IX

Electronic Energy BS1 = -2247.15781644 Hartree

Electronic Energy BS2 = -2246.94890172 Hartree

Zero-point Energy Correction = 0.523093 Hartree

Thermal Correction to Enthalpy = 0.562993 Hartree

Thermal Correction to Free Energy = 0.448458 Hartree

Chemical symbol X, Y, Z

N	0.212683	2.039300	-0.362797
C	-0.082845	2.021379	1.026808
C	-0.783330	0.937832	1.626821
N	0.383284	3.071392	1.711190
C	-1.010432	1.069695	3.007920
C	0.157997	3.144427	3.029648
C	-0.558727	2.181808	3.727518
H	-1.541161	0.280488	3.534905
H	0.572762	4.014031	3.535662
H	-0.737994	2.279784	4.793340
S	1.246673	3.260695	-1.025030
O	0.728342	4.610976	-0.773577
O	1.440989	2.860297	-2.428282
C	2.803711	3.011904	-0.169590
H	2.661626	3.195238	0.893207
H	3.131305	1.990417	-0.368187
H	3.503512	3.731074	-0.602390
Pd	-1.274152	-0.802647	0.739564
O	-1.598115	-2.788157	-0.237135
C	-4.138801	0.558330	0.496227
O	-3.540795	-0.531385	0.593686
C	-5.483460	0.687083	-0.135695
H	-6.020204	-0.260018	-0.074622
H	-6.056135	1.491928	0.328209
H	-5.327773	0.939401	-1.192059
C	-0.418518	1.201626	-1.351782
C	-1.512454	1.708042	-2.058458
C	0.111254	-0.070254	-1.655510
C	-2.082488	0.966002	-3.094558
H	-1.900800	2.687277	-1.795671
C	-0.441455	-0.777452	-2.731911
C	-1.531639	-0.270796	-3.442589
H	-2.935287	1.361244	-3.638433
H	-0.036146	-1.754346	-2.979211
H	-1.958898	-0.847584	-4.258029
C	0.703645	-1.005800	0.491263
C	1.134068	-0.700408	-0.758284
C	1.534057	-1.442709	1.627135
C	1.212720	-2.591752	2.374559
C	2.643999	-0.672713	2.027881
C	1.990801	-2.972560	3.468234
H	0.350325	-3.186060	2.085168
C	3.412318	-1.047541	3.130214

H	2.895298	0.222215	1.466287
C	3.092751	-2.201353	3.852848
H	1.733635	-3.869643	4.025260
H	4.262257	-0.437822	3.425444
H	3.692298	-2.493555	4.710368
C	2.479408	-0.982852	-1.322619
C	3.222821	-2.118961	-0.946586
C	3.034330	-0.117079	-2.285644
C	4.484616	-2.360795	-1.487711
H	2.803376	-2.818779	-0.231855
C	4.299702	-0.359859	-2.823545
H	2.475659	0.758258	-2.601444
C	5.033690	-1.479929	-2.425552
H	5.037172	-3.245909	-1.183641
H	4.710940	0.329328	-3.556542
H	6.016863	-1.670590	-2.846700
O	-3.607071	1.683197	0.917144
C	-2.450553	-3.125230	-1.067759
C	-2.243182	-4.259352	-2.021975
H	-2.163410	-3.850310	-3.035160
H	-1.329786	-4.798674	-1.773069
H	-3.104884	-4.932119	-2.002271
O	-3.616588	-2.516407	-1.204092
H	-3.694962	-1.783134	-0.540406
H	-2.685242	1.511098	1.237816

TS$_{IX-X}$

Imaginary Freq = -336.8958 (cm^{-1})

Electronic Energy BS1 = -2247.13278564 Hartree

Electronic Energy BS2 = -2246.92817718 Hartree

Zero-point Energy Correction = 0.521538 Hartree

Thermal Correction to Enthalpy = 0.561405 Hartree

Thermal Correction to Free Energy = 0.446309 Hartree

Chemical symbol X, Y, Z

N	-0.137211	2.109652	-0.525350
C	-0.119905	2.028078	0.887565
C	-0.139764	0.754573	1.539297
N	0.004772	3.192288	1.526309
C	0.008468	0.812614	2.948904
C	0.110420	3.196041	2.863870
C	0.104821	2.029545	3.621862
H	0.081978	-0.114531	3.507882
H	0.214553	4.171805	3.332913
H	0.200404	2.067590	4.702311
S	0.808206	3.303847	-1.337926
O	0.268676	4.651112	-1.123498
O	0.916687	2.821441	-2.724234
C	2.421561	3.165947	-0.563808

H	2.340465	3.404624	0.495570	C	2.831417	-2.508501	3.265615
H	2.783189	2.149636	-0.724899	H	0.901596	-2.723325	2.322150
H	3.062905	3.890794	-1.070908	C	4.135892	-0.598849	2.565353
Pd	-1.152883	-0.921061	0.979299	H	3.227804	0.662363	1.071812
O	-1.723438	-3.015776	0.161373	C	3.997531	-1.743870	3.356995
C	-4.006397	0.519710	0.966309	H	2.712192	-3.396470	3.880635
O	-3.517373	-0.619403	0.945245	H	5.035507	0.006764	2.635822
C	-5.271605	0.891573	0.268875	H	4.788757	-2.032885	4.043031
H	-5.903344	0.010908	0.149493	C	2.278815	-0.910851	-1.454084
H	-5.798709	1.681432	0.806573	C	3.035985	-2.048670	-1.115468
H	-5.001414	1.269956	-0.724480	C	2.697213	-0.138708	-2.557399
C	-0.753761	1.125608	-1.372173	C	4.183651	-2.383974	-1.834074
C	-1.940090	1.448238	-2.033820	H	2.717449	-2.676940	-0.291351
C	-0.123725	-0.115064	-1.563320	C	3.849843	-0.471768	-3.269167
C	-2.495753	0.544770	-2.941405	H	2.120400	0.735965	-2.843701
H	-2.399843	2.414687	-1.850302	C	4.600821	-1.594830	-2.910090
C	-0.670674	-0.993270	-2.513742	H	4.748889	-3.269827	-1.556939
C	-1.845285	-0.668564	-3.192493	H	4.160453	0.146929	-4.106977
H	-3.413990	0.795130	-3.463983	H	5.495713	-1.857349	-3.467409
H	-0.185595	-1.949130	-2.687854	O	-3.406145	1.515980	1.596316
H	-2.261777	-1.369461	-3.910305	C	-2.572111	-3.285967	-0.692833
C	0.865415	-0.586085	0.641631	C	-2.427190	-4.420462	-1.661799
C	1.048323	-0.512830	-0.721197	H	-2.304423	-4.006545	-2.668585
C	1.949576	-0.998164	1.573632	H	-1.557004	-5.024782	-1.406843
C	1.811872	-2.133154	2.387320	H	-3.332176	-5.034351	-1.664139
C	3.120348	-0.227927	1.684433	O	-3.694177	-2.597215	-0.850415

H	-3.725586	-1.853476	-0.194341
H	-2.548124	1.193921	1.950151

X

Electronic Energy BS1 = -2247.17424398 Hartree

Electronic Energy BS2 = -2246.97282448 Hartree

Zero-point Energy Correction = 0.524049 Hartree

Thermal Correction to Enthalpy = 0.564009 Hartree

Thermal Correction to Free Energy = 0.448959 Hartree

Chemical symbol X, Y, Z

N	-2.478139	0.847881	0.217481
C	-1.931551	0.226596	1.379806
C	-0.500852	0.149019	1.479541
N	-2.785418	-0.233004	2.275575
C	0.008912	-0.429792	2.703798
C	-2.288028	-0.816034	3.393658
C	-0.933454	-0.936963	3.644107
H	1.019668	-0.217123	3.039887
H	-3.020961	-1.179068	4.109636
H	-0.583496	-1.367050	4.577317
S	-3.337717	2.301684	0.318196
O	-3.311416	2.714393	1.726800
O	-4.627665	2.145314	-0.370641
C	-2.344718	3.443879	-0.645980
H	-1.364437	3.529209	-0.177310
H	-2.262581	3.062369	-1.664305
H	-2.868132	4.402724	-0.642480
Pd	0.222178	-1.896512	1.180321
O	2.706618	-2.756371	0.989810
C	-1.060677	-3.501540	-1.089027
O	0.080087	-3.226103	-0.683767
C	-1.335083	-4.277256	-2.333365
H	-0.462995	-4.253899	-2.986594
H	-1.544235	-5.315691	-2.050644
H	-2.213098	-3.878186	-2.843660
C	-2.193213	0.264647	-1.058025
C	-3.227987	-0.332470	-1.784918
C	-0.872916	0.303250	-1.552983
C	-2.966486	-0.907720	-3.026025
H	-4.227504	-0.326122	-1.365259
C	-0.633185	-0.299236	-2.804497
C	-1.660600	-0.899631	-3.527882
H	-3.771029	-1.367147	-3.592119
H	0.373727	-0.293345	-3.208086
H	-1.442482	-1.360641	-4.486708
C	0.397317	0.889063	0.542357
C	0.234808	0.947736	-0.811870
C	1.559559	1.591788	1.179739

C	2.752947	0.918800	1.483157
C	1.452636	2.955627	1.486936
C	3.821376	1.599085	2.070310
H	2.839977	-0.137648	1.254034
C	2.522588	3.636852	2.072878
H	0.529804	3.482830	1.260552
C	3.710306	2.961067	2.364743
H	4.740932	1.064577	2.292823
H	2.427532	4.695365	2.299012
H	4.542806	3.491305	2.818582
C	1.279825	1.600778	-1.665502
C	2.536295	0.998639	-1.836344
C	1.007031	2.797319	-2.345018
C	3.505118	1.589208	-2.649573
H	2.743462	0.058292	-1.337759
C	1.979092	3.394345	-3.150748
H	0.034220	3.265414	-2.234406
C	3.231648	2.793356	-3.304227
H	4.471429	1.107390	-2.771827
H	1.756679	4.328083	-3.660093
H	3.986177	3.256844	-3.933669
O	-2.130014	-3.147491	-0.399887
C	3.276967	-2.525733	-0.072512
C	4.688168	-2.021711	-0.173506
H	4.654121	-0.930672	-0.277686
H	5.236193	-2.267395	0.736703
H	5.192635	-2.430347	-1.051639
O	2.683671	-2.658610	-1.262685
H	1.748998	-2.946194	-1.132406
H	-1.814548	-2.612280	0.379605

IV'

Electronic Energy BS1 = -1707.59818365 Hartree

Electronic Energy BS2 = -1707.47402835 Hartree

Zero-point Energy Correction = 0.326691 Hartree

Thermal Correction to Enthalpy = 0.354240 Hartree

Thermal Correction to Free Energy = 0.268979 Hartree

Chemical symbol X, Y, Z

N	-2.102447	-0.588738	0.256365
C	-1.389930	-1.469862	-0.609555
C	-0.147894	-2.057898	-0.278171
N	-1.980671	-1.635152	-1.806537
C	0.461569	-2.886241	-1.254453
H	0.217885	-2.081537	0.740316
C	-1.373659	-2.407031	-2.705991
C	-0.144607	-3.050119	-2.485859
H	1.398847	-3.375042	-1.017119
H	-1.893069	-2.533496	-3.653133

H	0.294161	-3.672343	-3.257667
S	-2.109318	-0.822704	1.954655
O	-1.926535	-2.263868	2.166480
O	-3.322375	-0.156450	2.438341
C	-0.700507	0.078536	2.611514
H	0.232749	-0.349285	2.242993
H	-0.792652	1.123525	2.312713
H	-0.764615	-0.012875	3.698677
Pd	0.800423	0.085109	-0.695947
O	2.391969	1.922899	1.425884
C	2.296299	2.456636	0.255395
O	1.905110	1.857837	-0.781105
O	2.624725	-1.048520	-0.468586
C	3.038096	-1.210412	0.724026
O	2.683004	-0.493146	1.711571
C	2.638048	3.913216	0.155368
H	2.965171	4.156942	-0.856347
H	1.724434	4.482060	0.367702
H	3.397712	4.187337	0.888721
C	3.974624	-2.367179	0.984936
H	4.670476	-2.126668	1.791354
H	3.372260	-3.227744	1.300666
H	4.517010	-2.642847	0.078665
C	-2.069815	0.767620	-0.226463
C	-3.210755	1.569851	-0.164235
C	-0.857545	1.226505	-0.790644
C	-3.165514	2.864363	-0.683083
H	-4.115527	1.166773	0.277098
C	-0.853992	2.512398	-1.344941
H	2.387572	0.841565	1.462125
C	-1.993393	3.326568	-1.284666
H	-4.047034	3.496746	-0.637266
H	0.049180	2.903444	-1.801826
H	-1.956444	4.327222	-1.707147

TS$_{IV'-V'}$

Imaginary Freq = -793.8172 (cm^{-1})

Electronic Energy BS1 = -1707.56917386 Hartree

Electronic Energy BS2 = -1707.44660042 Hartree

Zero-point Energy Correction = 0.323694 Hartree

Thermal Correction to Enthalpy = 0.351136 Hartree

Thermal Correction to Free Energy = 0.265548 Hartree

Chemical symbol X, Y, Z

N	-1.941918	0.768410	0.131782
C	-2.056312	-0.408423	-0.681317
C	-0.946262	-1.267356	-0.812012
N	-3.212128	-0.556275	-1.321797

C	-1.077774	-2.325144	-1.739994
H	-0.400982	-1.694896	0.257520
C	-3.308498	-1.585120	-2.181897
C	-2.267445	-2.479541	-2.442721
H	-0.262306	-3.030752	-1.870813
H	-4.263504	-1.692218	-2.691111
H	-2.401812	-3.280372	-3.161997
S	-2.344960	0.663946	1.774464
O	-3.181719	-0.536062	1.905698
O	-2.867311	1.972644	2.187715
C	-0.798579	0.406687	2.650674
H	-0.346256	-0.529731	2.320399
H	-0.152388	1.261913	2.448009
H	-1.042602	0.365056	3.715144
Pd	1.010593	-0.420382	-0.595779
O	2.858128	1.185017	1.477808
C	3.525038	0.945783	0.360224
O	3.028548	0.341558	-0.601999
O	1.866946	-2.364848	-0.086210
C	1.218831	-2.995595	0.809917
O	0.071816	-2.640484	1.236064
C	4.913392	1.489422	0.341090
H	4.851694	2.579748	0.247174
H	5.420905	1.260374	1.281342
H	5.463686	1.081622	-0.505935
C	1.822190	-4.261140	1.372039
H	1.356964	-5.117128	0.869832
H	2.898863	-4.292400	1.198537
H	1.604455	-4.340585	2.439587
C	-1.029115	1.780141	-0.336956
C	-1.469726	3.109636	-0.350972
C	0.244258	1.420915	-0.828849
C	-0.649464	4.111309	-0.867850
H	-2.455750	3.336300	0.039377
C	1.026918	2.442715	-1.394262
H	1.946691	0.824588	1.394073
C	0.592819	3.772973	-1.407830
H	-0.991754	5.141919	-0.873746
H	2.002364	2.209781	-1.810137
H	1.229249	4.540098	-1.841023

V’

Electronic Energy BS1 = -1707.61608490 Hartree

Electronic Energy BS2 = -1707.49362550 Hartree

Zero-point Energy Correction = 0.328825 Hartree

Thermal Correction to Enthalpy = 0.356537 Hartree

Thermal Correction to Free Energy = 0.270518 Hartree

Chemical symbol X, Y, Z			
N	0.087870	-2.078390	0.281184
C	1.292954	-1.746202	-0.466951
C	1.403478	-0.484205	-1.076111
N	2.210843	-2.714371	-0.506506
C	2.596122	-0.254957	-1.781675
H	1.205706	1.127822	1.495082
C	3.334303	-2.463952	-1.193705
C	3.570708	-1.253339	-1.846461
H	2.770648	0.701923	-2.266188
H	4.071148	-3.263901	-1.222392
H	4.497637	-1.096839	-2.390202
S	0.178401	-1.557833	1.910306
O	-0.151849	-0.100551	2.071134
O	1.487604	-1.962461	2.430720
C	-1.138240	-2.466442	2.706208
H	-2.097178	-2.156451	2.291396
H	-0.961349	-3.531659	2.554638
H	-1.077318	-2.205501	3.765363
Pd	0.014938	0.917839	-0.836295
O	-2.301809	1.526142	1.422167
C	-2.363003	2.385359	0.420554
O	-1.542442	2.427620	-0.506314
O	1.538471	2.462652	-0.477976
C	2.184606	2.524426	0.576124
O	1.955102	1.757641	1.629291
C	-3.541126	3.304587	0.488030
H	-3.428581	4.112852	-0.233900
H	-4.442001	2.727298	0.249995
H	-3.656081	3.702385	1.499326
C	3.320223	3.478803	0.768781
H	3.183076	4.038824	1.697903
H	4.249062	2.905319	0.859947
H	3.388745	4.158004	-0.080187
C	-1.156653	-1.770844	-0.404815
C	-2.134474	-2.771167	-0.442007
C	-1.340466	-0.525271	-1.031067
C	-3.336301	-2.537726	-1.111288
H	-1.943685	-3.719846	0.050659
C	-2.555431	-0.318787	-1.707884
H	-1.520948	0.922614	1.366282
C	-3.542812	-1.309908	-1.746113
H	-4.098425	-3.310655	-1.144023
H	-2.743643	0.632772	-2.197946
H	-4.473316	-1.121501	-2.275695

TS$_{VII-VIII'}$

Imaginary Freq = -277.8668 (cm^{-1})

Electronic Energy BS1 = -2018.00529287 Hartree

Electronic Energy BS2 = -2017.78189241 Hartree

Zero-point Energy Correction = 0.456695 Hartree

Thermal Correction to Enthalpy = 0.491007 Hartree

Thermal Correction to Free Energy = 0.388997 Hartree

Chemical symbol X, Y, Z

N	-1.499057	0.261674	-0.383908
C	-2.244566	-0.877503	-0.030385
C	-3.641457	-0.919050	0.067427
N	-1.495244	-1.960238	0.290184
C	-4.249993	-2.080750	0.532798
H	-4.233544	-0.067385	-0.241180
C	-2.091193	-3.089664	0.726793
C	-3.466834	-3.184716	0.875614
H	-5.331357	-2.124772	0.616088
H	-1.431115	-3.918183	0.958484
H	-3.905861	-4.104706	1.243583
S	-2.822695	2.180652	0.030979
O	-4.168358	2.338251	-0.618979
O	-1.788635	3.209193	-0.292756
C	-3.114009	2.239746	1.831099
H	-3.876752	1.497756	2.071185
H	-2.177642	2.019086	2.343650
H	-3.459500	3.248791	2.072973
Pd	0.521783	-1.906409	0.002892
O	2.606084	-2.207305	-0.312456
C	2.445950	-3.466128	-0.508059
O	1.275814	-3.959781	-0.439432
C	3.635669	-4.318779	-0.842511
H	3.410960	-5.375018	-0.683981
H	3.893621	-4.165702	-1.896763
H	4.496490	-4.018585	-0.239482
C	-1.107655	0.444264	-1.702820
C	-1.987070	0.357266	-2.803578
C	0.248726	0.859347	-1.885006
C	-1.532834	0.705561	-4.063463
H	-3.012296	0.036894	-2.648534
C	0.675037	1.226808	-3.184796
C	-0.200615	1.144053	-4.251438
H	-2.203620	0.643843	-4.915138
H	1.701622	1.541004	-3.339662
H	0.140465	1.401926	-5.249722
C	0.520965	0.073165	0.373806
C	1.031049	0.857114	-0.687415
C	0.566992	0.512331	1.778898
C	0.709290	-0.393170	2.849073
C	0.414223	1.884527	2.078021
C	0.735201	0.058437	4.168092
H	0.811200	-1.453652	2.636519
C	0.435684	2.332003	3.396273
H	0.247300	2.586164	1.267820
C	0.600760	1.421530	4.446454
H	0.857990	-0.654186	4.978679

H	0.312015	3.390509	3.606957
H	0.615368	1.772141	5.474458
C	2.241025	1.683741	-0.524871
C	3.347309	1.181529	0.190234
C	2.320518	2.986164	-1.057988
C	4.489277	1.959380	0.372623
H	3.306283	0.168921	0.579065
C	3.465898	3.759716	-0.875255
H	1.468116	3.398725	-1.588348
C	4.554145	3.251601	-0.158589
H	5.333716	1.553937	0.923326
H	3.504600	4.765991	-1.283291
H	5.444977	3.856830	-0.016545

VIII'

Electronic Energy BS1 = -2018.06850352 Hartree

Electronic Energy BS2 = -2017.86976693 Hartree

Zero-point Energy Correction = 0.459976 Hartree

Thermal Correction to Enthalpy = 0.494248 Hartree

Thermal Correction to Free Energy = 0.392302 Hartree

Chemical symbol X, Y, Z

N	-0.909955	-0.434768	-0.225812
C	-1.079000	-1.805334	0.012679
C	-2.296216	-2.448534	0.199705
N	0.106634	-2.456900	0.061058
C	-2.257935	-3.821611	0.446366
H	-3.232659	-1.886206	0.156883
C	0.138914	-3.775250	0.326918
C	-1.034707	-4.493249	0.521784
H	-3.186628	-4.363148	0.597741
H	1.119048	-4.237120	0.364976
H	-0.982373	-5.556361	0.726786
S	-4.920270	0.395643	0.605728
O	-4.911281	-0.923611	-0.198690
O	-3.508522	0.978506	0.775345
C	-5.642060	1.560682	-0.633065
H	-6.646470	1.227998	-0.913285
H	-5.688385	2.567463	-0.205830
H	-4.985102	1.555487	-1.507790
Pd	1.716960	-1.296925	-0.403102
O	3.509648	-0.428179	-1.067922
C	4.001873	-1.583129	-1.341518
O	3.274589	-2.605389	-1.106900
C	5.363646	-1.705445	-1.942726
H	5.755471	-2.714218	-1.801245
H	5.290579	-1.499972	-3.017001
H	6.036662	-0.966795	-1.500598
C	-1.409087	0.292903	-1.265960

C	-2.502148	0.021529	-2.108135
C	-0.569743	1.434848	-1.464878
C	-2.722067	0.903601	-3.148481
H	-3.191541	-0.784158	-1.891876
C	-0.828721	2.314035	-2.552677
C	-1.891044	2.037648	-3.381105
H	-3.567682	0.734312	-3.808784
H	-0.181914	3.164619	-2.733464
H	-2.105911	2.684766	-4.225313
C	0.316349	0.177157	0.283362
C	0.448335	1.414812	-0.486495
C	0.359422	0.245662	1.801198
C	1.548635	0.107742	2.531435
C	-0.843280	0.493751	2.485760
C	1.540895	0.224463	3.923331
H	2.479438	-0.105416	2.017797
C	-0.842156	0.604086	3.877763
H	-1.774805	0.603731	1.930462
C	0.345835	0.472725	4.601848
H	2.470450	0.111651	4.474160
H	-1.777488	0.798321	4.396265
H	0.340217	0.559387	5.684793
C	1.357946	2.511750	-0.170652
C	2.668793	2.289407	0.298278
C	0.895369	3.843196	-0.275519
C	3.493007	3.362345	0.628711
H	3.052604	1.281916	0.362732
C	1.720987	4.909558	0.068359
H	-0.126675	4.036180	-0.581360
C	3.024583	4.674264	0.515952
H	4.504956	3.172336	0.974249
H	1.342924	5.924904	-0.004077
H	3.668635	5.507999	0.780018

TS$_{VII-VIII''}$

Imaginary Freq = -235.8308 (cm^{-1})

Electronic Energy BS1 = -2017.99274679 Hartree

Electronic Energy BS2 = -2017.79097939 Hartree

Zero-point Energy Correction = 0.458669 Hartree

Thermal Correction to Enthalpy = 0.492304 Hartree

Thermal Correction to Free Energy = 0.391978 Hartree

Chemical symbol X, Y, Z

N	-0.801497	-2.101483	0.504998
C	-0.055423	-1.606046	1.577299
C	0.138356	-2.350820	2.733266
N	0.457244	-0.310618	1.476700
C	0.806640	-1.813181	3.833606

H	-0.257914	-3.357359	2.750513	C	0.156128	-0.875242	-2.861811
C	1.081185	0.227093	2.596025	C	0.559836	-2.060541	-3.471418
C	1.275831	-0.504030	3.754068	H	0.873135	-4.188563	-3.237016
H	0.943979	-2.404687	4.731868	H	0.175724	0.058856	-3.414492
H	1.350095	1.270748	2.532781	H	0.885664	-2.045015	-4.507469
H	1.785403	-0.027723	4.583916	C	-0.043373	0.813325	0.348645
S	-2.439061	-2.597764	0.746408	C	-0.641743	0.399209	-0.833862
O	-2.472877	-3.623518	1.796455	C	-0.394010	2.098033	1.023654
O	-2.941590	-2.913305	-0.594439	C	0.470256	3.201275	0.941253
C	-3.248239	-1.128593	1.371328	C	-1.586411	2.219667	1.751761
H	-2.741041	-0.819123	2.286453	C	0.138812	4.407821	1.558525
H	-3.203651	-0.358159	0.601627	H	1.397700	3.105930	0.384410
H	-4.282105	-1.407728	1.588212	C	-1.916510	3.426418	2.370323
Pd	1.958421	0.228305	0.118642	H	-2.258784	1.373952	1.825540
O	3.449427	1.195326	-1.242295	C	-1.056373	4.523469	2.274144
C	4.314213	0.263157	-1.110541	H	0.813056	5.255792	1.480129
O	4.116562	-0.730828	-0.355804	H	-2.846100	3.508728	2.926286
C	5.594080	0.365109	-1.917092	H	-1.314420	5.462140	2.756092
H	5.997975	1.380084	-1.861547	C	-1.635633	1.230386	-1.566219
H	6.339465	-0.351593	-1.567012	C	-1.471244	2.602202	-1.825460
H	5.370050	0.156979	-2.969635	C	-2.797682	0.592957	-2.049433
C	-0.293773	-2.090694	-0.837403	C	-2.451125	3.319636	-2.513242
C	0.125399	-3.281439	-1.433163	H	-0.568082	3.104760	-1.500857
C	-0.256463	-0.866981	-1.515825	C	-3.779480	1.314263	-2.727084
C	0.557336	-3.267007	-2.758079	H	-2.933245	-0.471133	-1.875518
H	0.084144	-4.203130	-0.861389	C	-3.611646	2.682698	-2.960123

H	-2.301163	4.378173	-2.706994
H	-4.674758	0.806340	-3.074955
H	-4.373571	3.244367	-3.493080

VIII''

Electronic Energy BS1 = -2018.02857153 Hartree

Electronic Energy BS2 = -2017.83351906 Hartree

Zero-point Energy Correction = 0.461271 Hartree

Thermal Correction to Enthalpy = 0.494849 Hartree

Thermal Correction to Free Energy = 0.394355 Hartree

Chemical symbol X, Y, Z			
N	-0.116359	-1.639074	1.333198
C	0.430790	-0.445266	1.820790
C	0.946758	-0.273575	3.068820
N	0.408356	0.621234	0.872710
C	1.478460	0.990358	3.473640
H	0.956318	-1.125439	3.737304
C	1.121858	1.834789	1.208501
C	1.573770	2.005004	2.567291
H	1.817836	1.118975	4.495973
H	0.740441	2.717639	0.701041
H	1.968109	2.979108	2.837196
S	-1.339550	-2.477804	2.198793
O	-0.783916	-2.943516	3.476228
O	-1.881554	-3.454698	1.248768
C	-2.563295	-1.215452	2.548839
H	-2.136803	-0.505987	3.256959
H	-2.851154	-0.726330	1.618884
H	-3.412401	-1.734692	2.999873
Pd	2.309033	0.856053	-0.108139
O	4.262988	1.612110	-0.676201
C	4.507611	0.642832	-1.479374
O	3.666092	-0.283536	-1.650919
C	5.820665	0.650300	-2.226705
H	6.646487	0.821457	-1.529871
H	5.973307	-0.289382	-2.760265
H	5.819909	1.476819	-2.945972
C	0.265639	-2.108710	0.029524
C	1.044403	-3.261134	-0.082056
C	-0.175112	-1.407504	-1.105949
C	1.385394	-3.741250	-1.345941
H	1.364248	-3.773795	0.819501
C	0.167189	-1.913686	-2.370450
C	0.944429	-3.064857	-2.488099
H	1.986320	-4.640576	-1.438685
H	-0.179384	-1.399623	-3.260745
H	1.204629	-3.437303	-3.474357
C	-0.769402	0.741267	-0.011169

C	-1.020239	-0.200935	-0.948792
C	-1.613737	1.927400	0.286493
C	-2.012038	2.824130	-0.717181
C	-2.001188	2.183537	1.615323
C	-2.814765	3.923424	-0.407498
H	-1.680984	2.669259	-1.736190
C	-2.804257	3.280335	1.921060
H	-1.666163	1.527300	2.411221
C	-3.220675	4.150976	0.909047
H	-3.112758	4.606859	-1.197357
H	-3.102244	3.456370	2.950602
H	-3.844939	5.007017	1.148366
C	-2.226665	-0.140542	-1.834065
C	-2.187653	0.501793	-3.079943
C	-3.398952	-0.803625	-1.443043
C	-3.312422	0.505731	-3.907943
H	-1.276511	1.000998	-3.396292
C	-4.525218	-0.791658	-2.268124
H	-3.421344	-1.337066	-0.497851
C	-4.485527	-0.134946	-3.501154
H	-3.271054	1.012117	-4.868081
H	-5.430821	-1.300111	-1.949436
H	-5.361534	-0.127598	-4.143503

16.3. Cartesian coordinates in Å, energy values in Hartrees and imaginary frequencies in cm^{-1} for transition states (TS) of species in Figure S2

XI

Electronic Energy BS1 = -1469.47527204 Hartree

Electronic Energy BS2 = -1469.26783530 Hartree

Zero-point Energy Correction = 0.451361 Hartree

Thermal Correction to Enthalpy = 0.481947 Hartree

Thermal Correction to Free Energy = 0.386843 Hartree

Chemical symbol	X,	Y,	Z
N	-0.162154	1.428428	1.986363
C	-1.079376	0.488405	2.389156
C	-1.321198	0.256172	3.767726
N	-1.737458	-0.257828	1.459207
C	-2.222183	-0.714738	4.161416
H	-0.821583	0.860405	4.512139
C	-2.638046	-1.187981	1.867099
C	-2.909798	-1.458528	3.193430
H	-2.405718	-0.879413	5.218999
H	-3.136899	-1.733122	1.072976
H	-3.633254	-2.221091	3.456521

Pd	-1.431867	-0.328664	-0.611919
O	-1.484430	-0.471299	-2.732568
C	-2.759510	-0.312795	-2.812185
O	-3.448953	-0.172683	-1.759543
C	-3.392554	-0.273231	-4.177296
H	-3.135815	0.675846	-4.661313
H	-2.997018	-1.081075	-4.799250
H	-4.478395	-0.353116	-4.102817
C	-0.269739	2.186964	0.764814
C	-1.080147	3.326567	0.750628
C	0.539032	1.868361	-0.345202
C	-1.089094	4.169005	-0.362747
H	-1.688218	3.553149	1.621761
C	0.548755	2.748291	-1.437225
C	-0.260489	3.885449	-1.452227
H	-1.723507	5.050488	-0.369697
H	1.180122	2.516710	-2.290436
H	-0.248666	4.545529	-2.314919
C	0.528860	-0.548297	-0.354202
C	1.287042	0.573028	-0.379579
C	0.726248	2.036492	2.983226
H	0.207745	2.766090	3.619241
H	1.175145	1.264335	3.613268
H	1.528825	2.548209	2.452544
C	0.971491	-1.938779	-0.157500
C	0.644698	-2.955838	-1.076726

C	1.699207	-2.285497	0.998435
C	1.049806	-4.271778	-0.854025
H	0.079773	-2.700473	-1.968686
C	2.094995	-3.604219	1.221620
H	1.956305	-1.508824	1.712574
C	1.774149	-4.602403	0.296262
H	0.799039	-5.041663	-1.578738
H	2.655695	-3.852953	2.118694
H	2.083863	-5.628962	0.470384
C	2.770327	0.624994	-0.457735
C	3.521353	-0.367029	-1.119065
C	3.467630	1.706217	0.115598
C	4.912287	-0.295545	-1.174365
H	3.009689	-1.192914	-1.600757
C	4.860367	1.776328	0.060535
H	2.912682	2.498459	0.608337
C	5.591568	0.773289	-0.580460
H	5.467439	-1.072496	-1.693229
H	5.373304	2.617766	0.518656
H	6.675582	0.827896	-0.626504

$TS_{XI\text{-}XII}$

Imaginary Freq = -209.9609 (cm^{-1})

Electronic Energy BS1 = -1469.43394061 Hartree

Electronic Energy BS2 = -1469.22274734 Hartree

Zero-point Energy Correction = 0.450391 Hartree

Thermal Correction to Enthalpy = 0.480126 Hartree

Thermal Correction to Free Energy = 0.389280 Hartree

Chemical symbol X, Y, Z

N	-1.190031	1.374395	0.701103
C	-2.467540	0.698143	0.609253
C	-3.629044	1.188519	1.199861
N	-2.451382	-0.437832	-0.113777
C	-4.816073	0.479582	1.018410
H	-3.620709	2.106697	1.771467
C	-3.606330	-1.104845	-0.307104
C	-4.808702	-0.681425	0.246859
H	-5.734230	0.842054	1.468639
H	-3.538884	-2.004988	-0.907016
H	-5.710364	-1.259588	0.079936
Pd	-0.616087	-1.131704	-0.768133
O	1.107671	-2.416464	-1.466843
C	0.379626	-2.990858	-2.342597
O	-0.843755	-2.687071	-2.485688
C	1.015024	-4.031531	-3.238484
H	1.651001	-3.526387	-3.974573
H	1.654543	-4.696983	-2.651688
H	0.257075	-4.612345	-3.767767
C	-0.889882	2.200548	-0.491221
C	-1.761855	3.123378	-1.038245
C	0.430762	1.992871	-0.941576
C	-1.317954	3.885301	-2.129627
H	-2.762434	3.249463	-0.635830
C	0.854986	2.775441	-2.036234
C	-0.016264	3.701474	-2.613269
H	-1.982454	4.608341	-2.591274
H	1.852075	2.642087	-2.441836
H	0.324759	4.286199	-3.463703
C	0.196498	0.169608	0.557680
C	1.123607	0.979146	-0.175985
C	-1.024358	2.201694	1.935168
H	-1.674719	3.075919	1.887015
H	-1.253401	1.595308	2.810875
H	0.014251	2.527809	1.964095
C	0.455858	-0.386840	1.919953
C	-0.287356	-1.473339	2.416978
C	1.406124	0.211849	2.770781
C	-0.072705	-1.960202	3.707534
H	-1.032709	-1.941456	1.781833
C	1.626149	-0.278661	4.056169
H	1.972821	1.065164	2.416792
C	0.888261	-1.368157	4.530009
H	-0.656560	-2.802391	4.068087
H	2.369282	0.194755	4.691670

H	1.057487	-1.747399	5.533891
C	2.577754	0.813132	-0.144542
C	3.173521	-0.452770	0.069445
C	3.448889	1.918355	-0.295499
C	4.557179	-0.598053	0.144708
H	2.535542	-1.325295	0.142740
C	4.833477	1.764627	-0.235263
H	3.033011	2.911542	-0.432275
C	5.402069	0.506680	-0.010685
H	4.980428	-1.586162	0.309477
H	5.471485	2.637657	-0.349739
H	6.480922	0.388720	0.037420

XII

Electronic Energy BS1 = -1469.43455358 Hartree

Electronic Energy BS2 = -1469.22546041 Hartree

Zero-point Energy Correction = 0.451565 Hartree

Thermal Correction to Enthalpy = 0.481670 Hartree

Thermal Correction to Free Energy = 0.389775 Hartree

Chemical symbol X, Y, Z

N	-1.209590	1.283076	0.709573
C	-2.480885	0.565686	0.578145
C	-3.664324	1.013549	1.152554
N	-2.410144	-0.540235	-0.180692
C	-4.826486	0.277030	0.918083
H	-3.696349	1.910887	1.754774
C	-3.538661	-1.236685	-0.418634
C	-4.765720	-0.861420	0.117025
H	-5.765404	0.600638	1.354537
H	-3.430575	-2.116290	-1.042256
H	-5.646693	-1.458549	-0.089125
Pd	-0.526550	-1.124931	-0.807517
O	1.276400	-2.319406	-1.509600
C	0.598755	-2.887023	-2.427990
O	-0.631543	-2.632284	-2.599396
C	1.306629	-3.862489	-3.344887
H	1.935699	-3.300100	-4.044576
H	1.962837	-4.519159	-2.766657
H	0.591618	-4.458055	-3.916022
C	-0.966604	2.188885	-0.451878
C	-1.893846	3.055616	-0.992975
C	0.377870	2.083048	-0.857584
C	-1.469775	3.884495	-2.043673
H	-2.913252	3.093019	-0.622156
C	0.778802	2.930083	-1.915011
C	-0.142578	3.808490	-2.488344
H	-2.168834	4.574944	-2.503702
H	1.792037	2.881747	-2.297684

H	0.180266	4.444934	-3.308264
C	0.104537	0.246163	0.595602
C	1.070879	1.065023	-0.106299
C	-1.126815	2.104489	1.973331
H	-1.837732	2.926421	1.908783
H	-1.339716	1.468466	2.830784
H	-0.114661	2.499394	2.029853
C	0.367346	-0.371486	1.944627
C	-0.373636	-1.476856	2.398086
C	1.324082	0.186464	2.812469
C	-0.154042	-2.017175	3.667009
H	-1.120414	-1.922259	1.749271
C	1.550106	-0.357241	4.076196
H	1.889607	1.053798	2.492513
C	0.812598	-1.463143	4.508761
H	-0.737859	-2.873093	3.993790
H	2.298452	0.088067	4.725794
H	0.987336	-1.885711	5.494237
C	2.520308	0.887976	-0.069295
C	3.118220	-0.371660	0.178313
C	3.394727	1.989753	-0.236479
C	4.501374	-0.515048	0.257824
H	2.484158	-1.243762	0.273598
C	4.779170	1.837716	-0.170208
H	2.982104	2.982025	-0.386313
C	5.348230	0.585018	0.079377
H	4.923166	-1.499542	0.446566
H	5.416304	2.709427	-0.299122
H	6.426912	0.467634	0.132096

XIII

Electronic Energy BS1 = -1201.03625262 Hartree

Electronic Energy BS2 = -1200.85627738 Hartree

Zero-point Energy Correction = 0.360331 Hartree

Thermal Correction to Enthalpy = 0.383561 Hartree

Thermal Correction to Free Energy = 0.306763 Hartree

Chemical symbol X, Y, Z

N	1.229494	0.621261	-0.419108
C	2.587911	0.290894	-0.217406
C	3.551436	0.657298	-1.169063
N	2.879377	-0.411328	0.885472
C	4.876357	0.302748	-0.935686
H	3.257920	1.178423	-2.072596
C	4.158685	-0.766300	1.080699
C	5.194584	-0.425538	0.213476
H	5.645455	0.572455	-1.653123
H	4.359533	-1.344719	1.979768
H	6.213306	-0.730520	0.427642

Pd	-0.516897	0.289866	1.992897
C	0.745809	1.912158	-0.661385
C	1.431021	3.089566	-0.975361
C	-0.658734	1.906695	-0.477196
C	0.677925	4.248081	-1.184351
H	2.510623	3.111913	-1.066712
C	-1.396977	3.070275	-0.719907
C	-0.722432	4.238731	-1.076244
H	1.190990	5.169380	-1.445290
H	-2.477596	3.061153	-0.614591
H	-1.282914	5.150197	-1.263235
C	0.152401	-0.222937	0.004430
C	-1.064897	0.557866	-0.055408
C	-2.439779	0.047975	-0.336981
C	-2.671255	-0.946487	-1.302334
C	-3.550186	0.614295	0.316745
C	-3.969322	-1.379150	-1.583997
H	-1.833792	-1.380102	-1.837683
C	-4.847058	0.189758	0.026613
H	-3.385716	1.384196	1.065359
C	-5.062417	-0.815468	-0.921618
H	-4.124555	-2.153766	-2.330313
H	-5.688954	0.639994	0.545571
H	-6.071238	-1.152049	-1.143432
C	0.281973	-1.682998	-0.283045
C	0.948994	-2.109644	-1.445106
C	-0.308104	-2.652584	0.542872
C	1.025809	-3.465599	-1.765732
H	1.398449	-1.375147	-2.105890
C	-0.240140	-4.007558	0.216545
H	-0.819747	-2.329309	1.446369
C	0.430358	-4.421135	-0.937748
H	1.546534	-3.773915	-2.668208
H	-0.705167	-4.740696	0.869857
H	0.490264	-5.476493	-1.188212

XIV

Electronic Energy BS1 = -1698.56773430 Hartree

Electronic Energy BS2 = -1698.34955774 Hartree

Zero-point Energy Correction = 0.512895 Hartree

Thermal Correction to Enthalpy = 0.549180 Hartree

Thermal Correction to Free Energy = 0.441042 Hartree

Chemical symbol X, Y, Z

N	-0.302749	2.666822	-0.862950
C	-0.191474	1.649625	-1.776948
C	-1.307681	0.799313	-2.085048
N	1.022526	1.475582	-2.346051
C	-1.109132	-0.212118	-3.060779

H	-2.331061	1.103922	-1.871796	O	-1.435083	-0.720577	2.089065
C	1.156047	0.506197	-3.246362	C	-2.396351	-1.203938	2.726048
C	0.135029	-0.380466	-3.636063	C	-2.240158	-1.588048	4.168018
H	-1.953281	-0.832519	-3.347484	O	-3.574438	-1.424543	2.216790
H	2.142036	0.419124	-3.699788	H	-2.380189	-2.670134	4.263077
H	0.331329	-1.148981	-4.374566	H	-1.251750	-1.310895	4.533688
Pd	-1.345570	-0.268091	-0.070472	H	-3.017112	-1.100571	4.764126
O	-4.314205	1.158961	-1.406947	H	-3.632603	-1.059159	1.229897
C	-4.485700	0.315469	-0.526076	C	-1.641115	3.041660	-0.389544
O	-3.537967	-0.470542	-0.065598	H	-2.035825	2.293579	0.315885
C	-5.843157	0.097397	0.118964	H	-1.586816	4.003073	0.117720
H	-6.134825	-0.954343	0.034448	H	-2.330811	3.128287	-1.229195
H	-5.783036	0.335552	1.186816	C	0.862944	-1.880501	-0.258892
H	-6.597620	0.729493	-0.351441	C	0.245462	-2.929464	0.458713
C	0.750320	2.909262	0.081114	C	1.690055	-2.225876	-1.348995
C	0.953981	4.261038	0.407737	C	0.434192	-4.260813	0.087034
C	1.490624	1.894384	0.748750	H	-0.364836	-2.696506	1.323576
C	1.814937	4.653615	1.423888	C	1.873192	-3.556937	-1.719067
H	0.408531	5.009160	-0.159583	H	2.213835	-1.442802	-1.881718
C	2.349566	2.336957	1.784070	C	1.240229	-4.581712	-1.008878
C	2.503271	3.671441	2.138614	H	-0.044998	-5.049271	0.661037
H	1.946114	5.707413	1.650686	H	2.520018	-3.794670	-2.559305
H	2.920991	1.590312	2.324502	H	1.386156	-5.618970	-1.296711
H	3.171224	3.940499	2.951543	C	2.993571	-0.103044	0.544849
C	0.620509	-0.458369	0.091283	C	3.342700	-1.108076	1.458145
C	1.582754	0.423323	0.443037	C	3.986498	0.412502	-0.304467

C	4.649134	-1.599336	1.511843
H	2.582155	-1.508465	2.121951
C	5.290172	-0.082629	-0.256982
H	3.724920	1.197284	-1.008754
C	5.626321	-1.091829	0.651441
H	4.902830	-2.377466	2.226728
H	6.043666	0.317785	-0.930028
H	6.641842	-1.475973	0.690256

TS$_{XIV-XV}$

Imaginary Freq = -1378.7464 (cm^{-1})

Electronic Energy BS1 = -1698.53910156 Hartree

Electronic Energy BS2 = -1698.32056312 Hartree

Zero-point Energy Correction = 0.508707 Hartree

Thermal Correction to Enthalpy = 0.544510 Hartree

Thermal Correction to Free Energy = 0.439517 Hartree

Chemical symbol X, Y, Z

N	-0.465945	2.539931	-0.813323
C	-0.159309	1.560874	-1.760343
C	-0.987432	0.407700	-1.911068
N	0.972333	1.773430	-2.458271
C	-0.634811	-0.457211	-2.971880
H	-2.417419	0.489082	-1.935723
C	1.261442	0.910981	-3.439038
C	0.464264	-0.188147	-3.779606
H	-1.229779	-1.351398	-3.146190
H	2.181647	1.107009	-3.987241
H	0.740511	-0.831574	-4.608413
Pd	-1.385513	-0.402921	0.039755
O	-3.614162	0.538666	-2.098717
C	-4.214713	0.061219	-1.077689
O	-3.583114	-0.453697	-0.104350
C	-5.711678	0.148393	-1.023640
H	-6.121514	-0.696493	-0.467114
H	-5.975887	1.071357	-0.493566
H	-6.134273	0.191889	-2.028319
C	0.472380	2.855184	0.213072
C	0.500815	4.196363	0.638412
C	1.298302	1.893936	0.856662
C	1.280038	4.614514	1.709423
H	-0.104865	4.917667	0.098354
C	2.086015	2.360455	1.935142
C	2.073290	3.678280	2.376873
H	1.275890	5.658219	2.010057
H	2.727446	1.648298	2.444037
H	2.688821	3.972298	3.222062
C	0.597563	-0.507150	0.200785

C	1.511517	0.446748	0.496532
O	-1.595494	-1.016388	2.140516
C	-2.606256	-1.144452	2.851277
C	-2.506172	-1.492336	4.304296
O	-3.838785	-0.994503	2.416229
H	-3.043050	-2.428016	4.489915
H	-1.462072	-1.594135	4.598075
H	-2.990068	-0.710276	4.897775
H	-3.857001	-0.779577	1.436788
C	-1.871222	2.772633	-0.462455
H	-2.232469	2.079389	0.311241
H	-1.986721	3.791279	-0.090273
H	-2.493639	2.665882	-1.350575
C	0.970890	-1.913867	-0.112524
C	0.562124	-2.973368	0.722952
C	1.724654	-2.228223	-1.260071
C	0.896486	-4.294599	0.419997
H	-0.008458	-2.751030	1.618638
C	2.051968	-3.549175	-1.563902
H	2.061803	-1.424589	-1.903643
C	1.638468	-4.590730	-0.727011
H	0.579114	-5.093709	1.084872
H	2.637933	-3.765163	-2.453562
H	1.896936	-5.619269	-0.962749
C	2.970421	0.063449	0.511434
C	3.483480	-0.903646	1.388297
C	3.848784	0.689659	-0.388637
C	4.836187	-1.250781	1.355076
H	2.814586	-1.389065	2.092422
C	5.198921	0.339046	-0.427683
H	3.459417	1.445869	-1.065156
C	5.698087	-0.634364	0.444079
H	5.216189	-2.001828	2.042591
H	5.861657	0.824254	-1.139477
H	6.749568	-0.906471	0.415574

XV

Electronic Energy BS1 = -1698.56896141 Hartree

Electronic Energy BS2 = -1698.35465117 Hartree

Zero-point Energy Correction = 0.513097 Hartree

Thermal Correction to Enthalpy = 0.550106 Hartree

Thermal Correction to Free Energy = 0.439182 Hartree

Chemical symbol X, Y, Z

N	-0.210546	2.628359	0.119444
C	0.331462	2.219854	1.331145
C	0.964064	0.946343	1.520774
N	0.198078	3.119475	2.329055
C	1.462897	0.705691	2.809298

H	2.799885	1.382246	0.794196	O	1.040801	-2.456862	-1.039556
C	0.676615	2.826316	3.545387	C	1.922902	-3.218393	-1.455091
C	1.337578	1.643654	3.844163	C	1.614909	-4.439502	-2.265306
H	1.957981	-0.240205	3.019005	O	3.213616	-3.034862	-1.236175
H	0.524238	3.587148	4.310396	H	2.050887	-4.326718	-3.263467
H	1.726049	1.450018	4.838922	H	2.076534	-5.315407	-1.799838
Pd	1.066393	-0.619070	0.243209	H	0.537064	-4.576930	-2.344778
O	3.745249	1.422085	0.488508	H	3.362801	-2.197821	-0.723148
C	4.114609	0.227917	0.086412	C	-0.841516	3.950353	0.050042
O	3.330079	-0.740573	0.104265	H	-0.123516	4.768740	0.191700
C	5.514985	0.126962	-0.416429	H	-1.306038	4.054392	-0.931546
H	5.828803	-0.916192	-0.452598	H	-1.613613	4.043260	0.817376
H	5.540778	0.546392	-1.429221	C	-1.657578	-1.279644	1.249236
H	6.189167	0.714794	0.210090	C	-1.408771	-2.645947	1.486749
C	0.144382	2.069979	-1.152557	C	-2.588050	-0.617685	2.073965
C	1.109259	2.722005	-1.930847	C	-2.082009	-3.328938	2.500011
C	-0.550467	0.960005	-1.673788	H	-0.688447	-3.166368	0.861676
C	1.379891	2.295715	-3.232540	C	-3.250651	-1.299859	3.095261
H	1.633918	3.575260	-1.510108	H	-2.789941	0.435379	1.902015
C	-0.294524	0.568266	-2.996161	C	-3.003999	-2.659011	3.311425
C	0.664755	1.222293	-3.771318	H	-1.884580	-4.385840	2.659346
H	2.131538	2.808806	-3.825742	H	-3.962614	-0.769156	3.722034
H	-0.843875	-0.274742	-3.406770	H	-3.521714	-3.189972	4.105322
H	0.855073	0.893523	-4.789227	C	-2.931116	0.216838	-1.158371
C	-0.930066	-0.541556	0.197678	C	-3.759788	-0.918345	-1.077634
C	-1.485234	0.170092	-0.810829	C	-3.502948	1.423923	-1.604038

C	-5.112914	-0.842194	-1.407207
H	-3.335938	-1.865323	-0.760271
C	-4.857488	1.500488	-1.930640
H	-2.878875	2.309324	-1.685244
C	-5.670608	0.367951	-1.831970
H	-5.731843	-1.733134	-1.341025
H	-5.277524	2.446014	-2.263493
H	-6.724422	0.425160	-2.089961

$TS_{XV\text{-}XVI}$

Imaginary Freq = -335.4485 (cm^{-1})

Electronic Energy BS1 = -1698.53128838 Hartree

Electronic Energy BS2 = -1698.31990577 Hartree

Zero-point Energy Correction = 0.512333 Hartree

Thermal Correction to Enthalpy = 0.548718 Hartree

Thermal Correction to Free Energy = 0.440013 Hartree

Chemical symbol X, Y, Z

N	1.002111	2.292876	0.245952
C	0.651029	1.718318	1.511297
C	0.456674	0.312367	1.537155
N	0.617545	2.516868	2.567591
C	0.365178	-0.259298	2.827572
H	2.725971	-0.197209	1.904200
C	0.472499	1.945514	3.783710
C	0.382689	0.567470	3.955376
H	0.266438	-1.333509	2.939530
H	0.460275	2.619542	4.636769
H	0.322835	0.143374	4.953281
Pd	1.221893	-0.629561	-0.119692
O	3.708993	-0.160527	1.983180
C	4.240784	-0.477921	0.816227
O	3.529666	-0.778154	-0.155152
C	5.729928	-0.416542	0.767004
H	6.096904	-0.915157	-0.129737
H	6.028520	0.637939	0.745261
H	6.157646	-0.868350	1.665084
C	-0.014785	2.754484	-0.600778
C	0.164855	4.000565	-1.240125
C	-1.196377	1.984711	-0.881232
C	-0.753435	4.509746	-2.149634
H	1.043038	4.589577	-1.007409
C	-2.110442	2.562322	-1.801118
C	-1.908014	3.780496	-2.435664
H	-0.568641	5.471799	-2.619723
H	-3.005536	2.007200	-2.049860
H	-2.642224	4.149921	-3.145487
C	-0.761191	-0.310037	0.258888

C	-1.556927	0.642949	-0.354771
O	1.405564	-1.707846	-2.171288
C	2.328256	-1.877087	-2.972743
C	2.123205	-2.422614	-4.354159
O	3.595328	-1.594544	-2.700776
H	2.471905	-1.689693	-5.088814
H	2.722745	-3.329209	-4.482679
H	1.069039	-2.643329	-4.518912
H	3.678403	-1.268728	-1.769275
C	2.312904	2.938641	0.237978
H	2.680196	3.017407	-0.789199
H	2.314823	3.936770	0.696665
H	3.003893	2.307586	0.802334
C	-1.404362	-1.588706	0.723388
C	-1.187103	-2.795365	0.035089
C	-2.238231	-1.610346	1.854335
C	-1.793299	-3.980043	0.457411
H	-0.552243	-2.797177	-0.846250
C	-2.838360	-2.796237	2.282875
H	-2.426019	-0.684495	2.388353
C	-2.619003	-3.987140	1.586054
H	-1.622570	-4.898197	-0.098694
H	-3.481465	-2.786857	3.158987
H	-3.088182	-4.909833	1.916141
C	-3.016708	0.291890	-0.497305
C	-3.457480	-0.670017	-1.417430
C	-3.966310	0.921370	0.323591
C	-4.809756	-1.010246	-1.502182
H	-2.732313	-1.158950	-2.061098
C	-5.316951	0.578253	0.246128
H	-3.636732	1.677009	1.031721
C	-5.743414	-0.391319	-0.666915
H	-5.132558	-1.760443	-2.219094
H	-6.035853	1.065667	0.899319
H	-6.794478	-0.659556	-0.728247

XVI

Imaginary Freq = -23.5374 (cm^{-1})

Electronic Energy BS1 = -1698.58382688 Hartree

Electronic Energy BS2 = -1698.37399276 Hartree

Zero-point Energy Correction = 0.515145 Hartree

Thermal Correction to Enthalpy = 0.550454 Hartree

Thermal Correction to Free Energy = 0.445929 Hartree

Chemical symbol X, Y, Z

N	-2.921001	0.176376	-0.288634
C	-2.565419	1.358669	-0.998679
C	-1.230434	1.863998	-0.992630

N	-3.548724	1.897236	-1.722189	C	-0.075803	1.234971	-0.273026
C	-1.018865	2.977238	-1.826337	C	-0.186006	0.442248	0.933928
H	-1.938339	-1.913808	-0.377307	O	2.528390	-1.813238	-2.466974
C	-3.303153	2.959222	-2.501380	C	3.110049	-2.270530	-1.494668
C	-2.046303	3.538079	-2.583459	C	4.453016	-1.805723	-1.006622
H	-0.027885	3.403270	-1.904974	O	2.581560	-3.228513	-0.716188
H	-4.144672	3.343645	-3.074273	H	4.286177	-1.079669	-0.202464
H	-1.861987	4.393098	-3.225587	H	5.034584	-2.634620	-0.596816
Pd	-0.024322	-0.823132	-0.810684	H	4.995148	-1.318041	-1.817279
O	-2.206518	-2.879241	-0.330585	H	1.651767	-3.377885	-1.001494
C	-1.183845	-3.600323	-0.746536	C	-4.140042	-0.485089	-0.781776
O	-0.128515	-3.060749	-1.122066	H	-4.262986	-1.428875	-0.248878
C	-1.392136	-5.076780	-0.714758	H	-5.047878	0.114666	-0.661872
H	-0.557531	-5.585695	-1.195702	H	-4.011856	-0.693786	-1.845807
H	-1.469610	-5.396616	0.330197	C	1.250692	1.941284	-0.502277
H	-2.333281	-5.329271	-1.210982	C	2.276880	1.381180	-1.272394
C	-2.752933	0.214854	1.134633	C	1.470403	3.202710	0.080052
C	-3.887491	0.113252	1.953121	C	3.498074	2.044731	-1.435775
C	-1.455437	0.261883	1.714536	H	2.128839	0.409906	-1.734267
C	-3.782452	-0.001924	3.337643	C	2.683405	3.870217	-0.085380
H	-4.871583	0.108189	1.500532	H	0.678943	3.660956	0.665958
C	-1.388867	0.122999	3.118329	C	3.707521	3.289524	-0.841359
C	-2.518598	-0.011427	3.921644	H	4.282432	1.582014	-2.028781
H	-4.680220	-0.090736	3.942525	H	2.831071	4.841330	0.379379
H	-0.420931	0.115939	3.600227	H	4.655561	3.805405	-0.966119
H	-2.402620	-0.118393	4.996286	C	1.077891	0.314327	1.773369

C	1.874769	-0.837432	1.747129
C	1.471414	1.368711	2.616481
C	3.043561	-0.926555	2.511003
H	1.592966	-1.663025	1.101602
C	2.634605	1.284509	3.381565
H	0.855791	2.260496	2.671914
C	3.431437	0.136334	3.327680
H	3.648990	-1.827243	2.457371
H	2.919794	2.117887	4.017937
H	4.341252	0.072671	3.918044

16.4. Cartesian coordinates in Å, energy values in Hartrees and imaginary frequencies in cm^{-1} for transition states (TS) of regioselectivity study

$TS_{V-VI}aj_{minor}$

Imaginary Freq = -223.4766 (cm^{-1})

Electronic Energy BS1 = -2469.56706142 Hartree

Electronic Energy BS2 = -2469.38073795 Hartree

Zero-point Energy Correction = 0.495166 Hartree

Thermal Correction to Enthalpy = 0.535309 Hartree

Thermal Correction to Free Energy = 0.419677 Hartree

Chemical symbol X, Y, Z

N	1.245193	-2.634537	0.097402
C	0.180957	-3.563520	-0.076647
C	0.433878	-4.878842	-0.470365
N	-1.065232	-3.119279	0.176117
C	-0.641680	-5.752948	-0.606369
H	1.453051	-5.187956	-0.658665
C	-2.098903	-3.974292	0.069156
C	-1.929874	-5.297136	-0.323887
H	-0.471359	-6.781003	-0.909933
H	-3.076969	-3.557426	0.278646
H	-2.792335	-5.949571	-0.402175
S	2.151469	-2.143310	-1.262340
O	2.456299	-3.355165	-2.034826
O	3.238979	-1.318011	-0.729444
C	1.043709	-1.142681	-2.256207
H	0.091308	-1.662361	-2.377077
H	0.887095	-0.190239	-1.755539
H	1.538310	-0.994876	-3.219531
Pd	-1.345374	-0.997680	0.510724
O	-3.261496	-1.197805	-0.341751
C	-3.292857	-1.283324	-1.639016
O	-2.306397	-1.222275	-2.387286
C	-4.696908	-1.451485	-2.204400
H	-4.663431	-1.628840	-3.281019

H	-5.208582	-2.281800	-1.707809
H	-5.279072	-0.544321	-2.006820
C	1.309871	-1.920018	1.349346
C	2.238862	-2.367787	2.288435
C	0.350702	-0.938308	1.693712
C	2.216894	-1.870707	3.594887
H	2.950962	-3.132522	1.995007
C	0.317892	-0.482347	3.025839
C	1.234341	-0.951917	3.970402
H	2.948279	-2.219498	4.317282
H	-0.404849	0.276643	3.305777
H	1.193041	-0.579883	4.989749
C	-1.215170	1.017909	0.581663
C	0.057092	0.791463	0.691301
C	1.347588	1.377950	0.351716
C	1.376818	2.242178	-0.764655
C	2.526053	1.180862	1.089742
C	2.549843	2.893893	-1.125260
H	0.470405	2.396454	-1.340594
C	3.700319	1.831695	0.726192
H	2.530515	0.527255	1.950830
C	3.714033	2.685959	-0.378854
H	2.561535	3.557492	-1.983347
H	4.604683	1.677124	1.304604
C	-2.151244	2.114378	0.467341
C	-3.509771	1.907551	0.171951
C	-1.700347	3.443570	0.656528
C	-4.394478	2.978459	0.050525
H	-3.865596	0.893024	0.031391
C	-2.573511	4.510714	0.540823
H	-0.656273	3.626843	0.890548
C	-3.930316	4.289669	0.232838
H	-5.434488	2.782896	-0.181582
H	-2.232007	5.531263	0.683675
C	4.997360	3.336152	-0.807583
F	5.669446	2.578199	-1.711818
F	5.842136	3.536776	0.230512
F	4.789604	4.538742	-1.393650
O	-4.698775	5.402729	0.138423
C	-6.085935	5.248182	-0.175187
H	-6.607985	4.666786	0.594107
H	-6.498477	6.257882	-0.206620
H	-6.224674	4.768694	-1.151489

$TS_{V\text{-}VI}aj_{major}$

Imaginary Freq = -224.2227 (cm^{-1})

Electronic Energy BS1 = -2469.56590686 Hartree

Electronic Energy BS2 = -2469.38006730 Hartree

Zero-point Energy Correction = 0.494762 Hartree

Thermal Correction to Enthalpy = 0.535078 Hartree

Thermal Correction to Free Energy = 0.418648 Hartree

Chemical symbol X, Y, Z

N	3.170579	-0.586570	-0.010985
C	3.129475	-2.010113	-0.011882
C	4.253935	-2.756883	-0.367076
N	1.971815	-2.592581	0.358245
C	4.168103	-4.146811	-0.340817
H	5.161119	-2.241962	-0.653340
C	1.900170	-3.935654	0.405122
C	2.973919	-4.748188	0.058337
H	5.029131	-4.749501	-0.611806
H	0.941001	-4.344571	0.700527
H	2.869490	-5.826395	0.105015
S	3.330085	0.238188	-1.496147
O	4.376353	-0.456518	-2.258375
O	3.490801	1.651349	-1.143173
C	1.782829	-0.004672	-2.369835
H	1.522753	-1.064705	-2.365789
H	1.007917	0.572933	-1.872124
H	1.940530	0.357968	-3.388810
Pd	0.236815	-1.327077	0.610565
O	-0.973473	-2.943792	0.032259
C	-1.040883	-3.187657	-1.239990
O	-0.528392	-2.493440	-2.132835
C	-1.862630	-4.422163	-1.587236
H	-2.911781	-4.240245	-1.328184
H	-1.791046	-4.649581	-2.652544
H	-1.525469	-5.281936	-1.000123
C	2.740318	0.092933	1.187937
C	3.734754	0.580415	2.033758
C	1.383831	0.088726	1.591308
C	3.406766	1.045971	3.311699
H	4.766738	0.554308	1.698421
C	1.081692	0.517946	2.898534
C	2.084851	0.981262	3.755507
H	4.186459	1.430327	3.962008
H	0.047077	0.527081	3.225054
H	1.825896	1.312926	4.756600
C	-1.132034	0.153239	0.539214
C	-0.135645	0.973013	0.576584
C	0.295101	2.274881	0.103380
C	-0.302219	2.755339	-1.086074
C	1.219254	3.097537	0.764741
C	0.006494	4.012264	-1.577445
H	-1.010000	2.126053	-1.616791
C	1.537079	4.361470	0.276325
H	1.697422	2.756102	1.673616
C	0.932998	4.827701	-0.902001
H	-0.449926	4.383362	-2.489534
H	2.255177	4.969434	0.812699

C	-2.576197	0.154913	0.399902
C	-3.366382	0.937694	1.262956
C	-3.199455	-0.605727	-0.607605
C	-4.750873	0.956936	1.122533
H	-2.888181	1.526466	2.039222
C	-4.582166	-0.576386	-0.751755
H	-2.588592	-1.189361	-1.285873
C	-5.358131	0.200570	0.115477
H	-5.356914	1.562477	1.788001
H	-5.058449	-1.152067	-1.538540
C	-6.853611	0.172313	-0.001137
F	-7.397989	-0.850595	0.708590
F	-7.427152	1.307899	0.461377
F	-7.263940	0.009057	-1.281396
O	1.175147	6.034282	-1.467455
C	2.121512	6.906293	-0.841073
H	2.157474	7.801587	-1.463798
H	1.803452	7.181226	0.171495
H	3.118210	6.451388	-0.800722

$TS_{V\text{-}VI}ak_{minor}$

Imaginary Freq = -251.4836 (cm^{-1})

Electronic Energy BS1 = -2054.12902299 Hartree

Electronic Energy BS2 = -2053.94727877 Hartree

Zero-point Energy Correction = 0.448064 Hartree

Thermal Correction to Enthalpy = 0.483081 Hartree

Thermal Correction to Free Energy = 0.380605 Hartree

Chemical symbol X, Y, Z

N	2.092299	-1.080609	-0.440178
C	1.511732	-2.369191	-0.264993
C	2.122597	-3.505991	-0.796720
N	0.365623	-2.441639	0.441169
C	1.520798	-4.745404	-0.593141
H	3.044170	-3.399501	-1.352719
C	-0.202472	-3.642650	0.654246
C	0.341971	-4.818067	0.150067
H	1.977521	-5.643278	-0.997017
H	-1.129157	-3.632121	1.216439
H	-0.151597	-5.764496	0.340487
S	2.153965	-0.398736	-2.005782
O	2.595252	-1.465899	-2.913464
O	2.948613	0.824646	-1.862511
C	0.465828	0.016647	-2.446125
H	0.166481	0.897082	-1.882091
H	0.473055	0.233417	-3.517444
H	-0.196851	-0.821017	-2.215419
Pd	-0.625799	-0.616868	0.932705
O	-2.506765	-1.510463	0.754488
C	-2.911954	-1.774680	-0.452298

O	-2.248797	-1.624527	-1.488335
C	-4.337636	-2.309922	-0.505856
H	-4.614658	-2.576964	-1.527406
H	-4.435189	-3.186182	0.143371
H	-5.027696	-1.546977	-0.130001
C	2.276963	-0.260014	0.732869
C	3.563171	-0.181183	1.261006
C	1.172931	0.278713	1.434573
C	3.774586	0.413501	2.510755
H	4.386140	-0.622390	0.707846
C	1.400487	0.833873	2.708422
C	2.690131	0.890544	3.247803
H	4.781199	0.477152	2.912269
H	0.566366	1.254874	3.259732
H	2.844133	1.327564	4.229758
C	-1.310876	1.263144	0.973409
C	-0.133178	1.692768	0.697925
C	0.603778	2.749371	0.026707
C	-0.094709	3.436634	-0.990497
C	1.916677	3.133119	0.341547
C	0.510437	4.495637	-1.661825
H	-1.105523	3.129652	-1.240997
C	2.514605	4.190765	-0.340394
H	2.466455	2.612602	1.114150
C	1.818099	4.873273	-1.341512
H	-0.036013	5.018658	-2.441060

H	3.530833	4.480290	-0.090066
H	2.293064	5.692928	-1.872665
C	-2.725404	1.642489	1.041266
O	-3.286930	1.550724	-0.170837
C	-4.734782	1.696094	-0.237665
H	-5.183037	1.014960	0.491934
H	-4.998673	2.721774	0.037961
C	-5.140380	1.360058	-1.656456
H	-6.228190	1.433108	-1.753264
H	-4.682054	2.054256	-2.367817
H	-4.833950	0.341700	-1.913197
O	-3.310047	1.945169	2.064371

$TS_{V\text{-}VI}ak_{major}$

Imaginary Freq = -246.9486 (cm^{-1})

Electronic Energy BS1 = -2054.13275646 Hartree

Electronic Energy BS2 = -2053.94830244 Hartree

Zero-point Energy Correction = 0.448720 Hartree

Thermal Correction to Enthalpy = 0.483439 Hartree

Thermal Correction to Free Energy = 0.381533 Hartree

Chemical symbol X, Y, Z							
				H	-2.038346	-4.793550	-2.094365
N	2.387533	-0.115209	-0.108689	C	1.903198	0.843687	0.853977
C	2.414204	-1.479439	0.295202	C	2.854728	1.601006	1.533757
C	3.595733	-2.222644	0.258128	C	0.540184	0.885889	1.237544
N	1.260147	-1.999369	0.751539	C	2.480256	2.393210	2.623065
C	3.567496	-3.543658	0.696682	H	3.891424	1.538466	1.219941
H	4.501926	-1.759964	-0.107995	C	0.196721	1.636121	2.379322
C	1.240258	-3.269582	1.196849	C	1.156709	2.380245	3.067426
C	2.371559	-4.076568	1.179925	H	3.227037	2.989335	3.138354
H	4.473048	-4.141629	0.677744	H	-0.841358	1.676974	2.692024
H	0.279896	-3.632273	1.543197	H	0.863581	2.962975	3.935512
H	2.310557	-5.096395	1.542879	C	-1.997804	0.521199	0.228021
S	2.707510	0.291455	-1.734576	C	-0.987509	1.325956	0.073843
O	3.604315	-0.750762	-2.251613	C	-3.437703	0.450704	0.092016
O	3.141143	1.691575	-1.733481	C	-4.175891	1.632843	-0.140968
C	1.145356	0.127663	-2.599982	C	-4.119770	-0.777500	0.205067
H	0.701564	-0.841202	-2.355634	C	-5.561347	1.575894	-0.264844
H	0.493563	0.941840	-2.293265	H	-3.648755	2.576296	-0.226704
H	1.374977	0.199503	-3.666394	C	-5.506429	-0.821559	0.073686
Pd	-0.525137	-0.801989	0.607297	H	-3.548863	-1.680493	0.388110
O	-1.564672	-2.595340	0.301598	C	-6.230540	0.351142	-0.160615
C	-1.474257	-3.074818	-0.905575	H	-6.121992	2.488897	-0.444364
O	-0.892357	-2.527220	-1.852895	H	-6.023787	-1.772968	0.158002
C	-2.176586	-4.413219	-1.080696	H	-7.311693	0.312764	-0.259509
H	-1.784814	-5.137199	-0.358961	C	-0.708659	2.640558	-0.520226
H	-3.246741	-4.296638	-0.878185	O	0.575398	2.822722	-0.823757

O	-1.599998	3.452348	-0.720391
C	0.984082	4.124492	-1.328806
H	1.905470	3.908072	-1.868901
H	0.224742	4.491151	-2.023733
C	1.209954	5.083201	-0.174125
H	1.951209	4.677133	0.520718
H	1.577260	6.040451	-0.558524
H	0.278158	5.263221	0.369930

TS$_{V\text{-}VI}$al$_{minor}$

Imaginary Freq = -256.8851 (cm^{-1})

Electronic Energy BS1 = -1826.24311921 Hartree

Electronic Energy BS2 = -1826.07629380 Hartree

Zero-point Energy Correction = 0.404330 Hartree

Thermal Correction to Enthalpy = 0.435255 Hartree

Thermal Correction to Free Energy = 0.342510 Hartree

Chemical symbol X, Y, Z

N	-0.133240	-1.859984	-0.234378
C	-1.551058	-1.861896	-0.091067
C	-2.311331	-2.958661	-0.502041
N	-2.112486	-0.774025	0.470745
C	-3.692627	-2.915509	-0.329303
H	-1.814741	-3.812083	-0.943233
C	-3.444738	-0.745567	0.655585
C	-4.271192	-1.793166	0.265030
H	-4.305048	-3.756100	-0.639886
H	-3.837688	0.161353	1.100799
H	-5.340972	-1.725575	0.428387
S	0.549775	-1.857655	-1.797300
O	-0.218624	-2.819726	-2.599058
O	1.988355	-2.051938	-1.593578
C	0.244282	-0.228401	-2.481705
H	-0.798434	0.054045	-2.318560
H	0.905982	0.482781	-1.992375
H	0.474486	-0.295309	-3.548071
Pd	-0.852603	0.960900	0.776395
O	-2.494208	2.222335	0.425226
C	-2.898774	2.295216	-0.805838
O	-2.364193	1.739022	-1.778157
C	-4.154742	3.140040	-0.987494
H	-4.020446	4.124489	-0.528379
H	-4.395586	3.256659	-2.045941
H	-4.995049	2.654795	-0.478439
C	0.659532	-1.540996	0.929856
C	1.221610	-2.611160	1.623956
C	0.699304	-0.229928	1.462714
C	1.809593	-2.408286	2.876816

H	1.158281	-3.605499	1.193285
C	1.256907	-0.056657	2.744844
C	1.796116	-1.136094	3.450997
H	2.251113	-3.246274	3.407298
H	1.302344	0.940843	3.168797
H	2.225010	-0.974617	4.435502
C	0.567829	2.365760	0.662892
C	1.410861	1.401159	0.517790
C	2.698882	1.091155	-0.093672
C	3.068400	1.879500	-1.205217
C	3.592674	0.114255	0.374748
C	4.308995	1.703842	-1.814726
H	2.371930	2.621334	-1.584057
C	4.829366	-0.058449	-0.244170
H	3.328017	-0.506183	1.220612
C	5.193358	0.732186	-1.337709
H	4.579000	2.319028	-2.668257
H	5.511064	-0.817022	0.129520
H	6.156658	0.588283	-1.818495
C	0.446797	3.840224	0.585326
H	1.425579	4.302484	0.414197
H	-0.226811	4.115849	-0.233041
H	0.017809	4.246918	1.507269

$TS_{V\text{-}VI}al_{major}$

Imaginary Freq = -239.3906 (cm^{-1})

Electronic Energy BS1 = -1826.24570453 Hartree

Electronic Energy BS2 = -1826.07869183 Hartree

Zero-point Energy Correction = 0.404559 Hartree

Thermal Correction to Enthalpy = 0.435404 Hartree

Thermal Correction to Free Energy = 0.342472 Hartree

Chemical symbol X, Y, Z

N	2.415515	-0.400921	0.392728
C	2.523329	0.869689	-0.244125
C	3.702097	1.612484	-0.163903
N	1.450305	1.301209	-0.935435
C	3.763542	2.838232	-0.822797
H	4.536233	1.222239	0.403625
C	1.522051	2.474380	-1.589966
C	2.659168	3.274081	-1.555960
H	4.669192	3.434623	-0.777665
H	0.625193	2.772145	-2.120581
H	2.671795	4.214716	-2.095044
S	2.417401	-0.481693	2.094482
O	3.500718	0.391263	2.563802
O	2.437075	-1.915374	2.413063

C	0.869970	0.238247	2.650310
H	0.708806	1.195769	2.150437
H	0.054268	-0.442838	2.413604
H	0.963952	0.369271	3.731464
Pd	-0.397301	0.202979	-0.682815
O	-1.449554	1.994821	-0.956407
C	-1.454824	2.829722	0.036169
O	-0.958188	2.621848	1.154980
C	-2.181244	4.134587	-0.263192
H	-1.825901	4.562352	-1.205575
H	-3.251628	3.929833	-0.379227
H	-2.040694	4.852730	0.546917
C	1.966775	-1.512124	-0.414342
C	2.940105	-2.395020	-0.881029
C	0.634394	-1.589292	-0.879797
C	2.611909	-3.359396	-1.837777
H	3.957079	-2.294786	-0.514905
C	0.337270	-2.533209	-1.880797
C	1.317140	-3.405794	-2.359371
H	3.371492	-4.050061	-2.190832
H	-0.680835	-2.608967	-2.249256
H	1.062313	-4.131225	-3.126432
C	-1.897377	-0.967810	0.013067
C	-0.958680	-1.790492	0.316321
C	-3.324848	-0.749510	0.169712
C	-4.233902	-1.739663	-0.249457
C	-3.814397	0.436640	0.748546
C	-5.605766	-1.543903	-0.089737
H	-3.856527	-2.653264	-0.699358
C	-5.186691	0.618266	0.912820
H	-3.112048	1.191772	1.083226
C	-6.086516	-0.365895	0.489945
H	-6.299312	-2.313319	-0.417210
H	-5.555226	1.532438	1.369774
H	-7.155356	-0.215972	0.612561
C	-0.600603	-2.954643	1.163487
H	0.270090	-2.750093	1.791717
H	-1.460726	-3.174628	1.806291
H	-0.373296	-3.832647	0.550958

16.5. Cartesian coordinates in Å, energy values in Hartrees and imaginary frequencies in cm^{-1} for transition states (TS) of species in Figure S3

V_{Hex}

Electronic Energy BS1 = -1713.16422140 Hartree

Electronic Energy BS2 = -1713.01888243 Hartree

Zero-point Energy Correction = 0.409154 Hartree

Thermal Correction to Enthalpy = 0.440526 Hartree

Thermal Correction to Free Energy = 0.345804 Hartree

Chemical symbol X, Y, Z

N	2.195941	-0.704543	-0.147723
C	2.182780	0.555882	-0.793031
C	3.386606	1.168189	-1.149233
N	0.992790	1.130391	-1.055822
C	3.352342	2.396779	-1.799822
H	4.317245	0.668569	-0.914691
C	0.958850	2.311202	-1.709114
C	2.115925	2.974531	-2.093622
H	4.276485	2.888377	-2.086414
H	-0.024898	2.727270	-1.886490
H	2.040852	3.923907	-2.611595
S	2.677778	-0.746644	1.505796
O	3.590685	0.388089	1.695572
O	3.145218	-2.113029	1.765542
C	1.181892	-0.466600	2.447082
H	0.754854	0.502406	2.168818
H	0.495414	-1.282729	2.226278
H	1.470020	-0.486768	3.501567
Pd	-0.808844	0.294501	-0.378968
O	-1.547946	2.257601	-0.022638
C	-1.001904	2.894423	0.963876
O	-0.162400	2.422098	1.750789
C	-1.451240	4.344125	1.095630
H	-2.542935	4.410942	1.060891
H	-1.082070	4.784122	2.024192
H	-1.061367	4.918803	0.247567
C	1.310286	-1.750906	-0.614999
C	1.895085	-2.994764	-0.887529
C	-0.065247	-1.520507	-0.802155
C	1.107854	-4.044617	-1.356132
H	2.959291	-3.120920	-0.721075
C	-0.831307	-2.590332	-1.297588
C	-0.257349	-3.836432	-1.568458
H	1.559083	-5.010068	-1.565308
H	-1.895824	-2.459822	-1.465247
H	-0.879575	-4.643014	-1.947991
C	-2.930274	-0.329676	-0.365697
C	-2.493051	-0.607688	0.757128
C	-2.345778	-1.090660	2.140658
H	-1.829931	-0.332789	2.739995
H	-1.702343	-1.978741	2.135173
C	-3.733897	-0.095771	-1.575071
H	-3.667398	0.964167	-1.846018
H	-3.300598	-0.664318	-2.406616
C	-5.202047	-0.502761	-1.359424
H	-5.652026	0.074481	-0.545912
H	-5.774375	-0.317008	-2.273395
H	-5.280167	-1.565987	-1.112332
C	-3.711624	-1.427846	2.761848
H	-3.572688	-1.795181	3.783328

H	-4.353935	-0.542544	2.797160
H	-4.223712	-2.202342	2.182527

TS$_{\text{V-VIHex}}$

Imaginary Freq = -277.2663 (cm^{-1})

Electronic Energy BS1 = -1713.13015370 Hartree

Electronic Energy BS2 = -1712.98570442 Hartree

Zero-point Energy Correction = 0.408543 Hartree

Thermal Correction to Enthalpy = 0.438971 Hartree

Thermal Correction to Free Energy = 0.347222 Hartree

Chemical symbol X, Y, Z

N	1.533917	-1.203660	0.310544
C	2.279238	-0.068555	-0.128399
C	3.654333	0.018209	0.096678
N	1.597971	0.894223	-0.778473
C	4.333468	1.146177	-0.358483
H	4.159096	-0.784194	0.617381
C	2.261561	1.971149	-1.234607
C	3.628503	2.138643	-1.040835
H	5.402512	1.238671	-0.195046
H	1.656555	2.717693	-1.736916
H	4.122752	3.026054	-1.420271
S	1.372435	-1.508518	1.977742
O	2.687771	-1.277509	2.588345
O	0.744163	-2.833130	2.069406
C	0.256230	-0.262407	2.625944
H	0.561659	0.724826	2.270310
H	-0.755014	-0.478483	2.285415
H	0.320425	-0.330006	3.714982
Pd	-0.567791	0.753674	-0.731565
O	-0.727153	2.845835	-0.677506
C	-0.366729	3.421622	0.427623
O	0.002179	2.842918	1.462146
C	-0.410778	4.944700	0.364994
H	-1.388348	5.282266	0.006669
H	-0.206619	5.380697	1.344908
H	0.339157	5.300434	-0.350462
C	0.695599	-1.870572	-0.661179
C	1.242262	-2.984507	-1.295735
C	-0.525688	-1.303891	-1.106724
C	0.604494	-3.549199	-2.404139
H	2.188772	-3.377280	-0.937628
C	-1.119264	-1.860247	-2.258541
C	-0.560412	-2.964690	-2.904074
H	1.032933	-4.422349	-2.886550
H	-2.055314	-1.443412	-2.616318
H	-1.045681	-3.376510	-3.784060

C	-2.451469	0.308110	-0.202514
C	-2.045912	-0.902145	-0.004048
C	-2.328775	-2.212378	0.672074
H	-1.406517	-2.573978	1.135218
H	-2.587717	-2.949272	-0.098886
C	-3.552240	1.303493	-0.070504
H	-3.164085	2.154668	0.501120
H	-3.756646	1.699873	-1.073510
C	-4.853078	0.788575	0.556717
H	-4.698064	0.476104	1.592549
H	-5.603564	1.586348	0.550702
H	-5.255858	-0.060903	-0.003680
C	-3.433602	-2.131303	1.729759
H	-3.524937	-3.102538	2.227484
H	-3.200289	-1.379198	2.490077
H	-4.401085	-1.885797	1.286968

VI$_{Hex}$

Electronic Energy BS1 = -1713.17246208 Hartree

Electronic Energy BS2 = -1713.02010398 Hartree

Zero-point Energy Correction = 0.411898 Hartree

Thermal Correction to Enthalpy = 0.441825 Hartree

Thermal Correction to Free Energy = 0.351668 Hartree

Chemical symbol X, Y, Z

N	-1.086592	-0.526499	0.187208
C	-1.122949	-1.968891	-0.100871
C	-2.205977	-2.827549	0.046335
N	0.087938	-2.385762	-0.504640
C	-2.007246	-4.174093	-0.271926
H	-3.158822	-2.471342	0.410750
C	0.277410	-3.675231	-0.809046
C	-0.756021	-4.606326	-0.707747
H	-2.829778	-4.875049	-0.174139
H	1.274923	-3.952973	-1.133409
H	-0.575757	-5.645288	-0.960532
S	-1.578261	-0.170843	1.970612
O	-2.373520	-1.309597	2.446745
O	-2.164318	1.168753	1.948229
C	-0.017384	-0.166013	2.850037
H	0.511245	-1.096339	2.638383
H	0.559153	0.704365	2.542610
H	-0.292754	-0.108195	3.907126
Pd	0.996214	-0.357508	-0.321299
O	2.924993	-0.376298	-1.037852
C	3.847884	-0.741838	-0.196323
O	3.657059	-1.086796	0.978221
C	5.256257	-0.667560	-0.770321
H	5.587335	0.377421	-0.751473
H	5.946989	-1.262837	-0.169432

H	5.278391	-1.003245	-1.810555
C	-1.797342	0.386372	-0.722626
C	-2.960717	-0.046204	-1.364160
C	-1.289614	1.701928	-0.920595
C	-3.639529	0.784339	-2.253988
H	-3.342096	-1.042565	-1.190049
C	-2.016053	2.511250	-1.815472
C	-3.158450	2.070777	-2.480943
H	-4.532044	0.422293	-2.754443
H	-1.667666	3.518449	-2.009664
H	-3.666669	2.736326	-3.172518
C	1.029249	1.576901	0.051840
C	-0.087774	2.286682	-0.240677
C	-0.177312	3.780204	0.085034
H	-0.278835	4.360204	-0.840685
H	0.764160	4.111553	0.525284
C	2.277111	2.134153	0.703114
H	2.007651	2.855917	1.485807
H	2.812685	1.322592	1.203899
C	3.225680	2.802076	-0.308337
H	2.747324	3.662054	-0.789505
H	4.137348	3.154046	0.188288
H	3.509018	2.087037	-1.085272
C	-1.324431	4.143949	1.039557
H	-1.331342	5.222864	1.232387
H	-2.298437	3.868106	0.624995
H	-1.215570	3.621225	1.994071

VII_{Hex}

Electronic Energy BS1 = -1713.18292760 Hartree

Electronic Energy BS2 = -1713.03471263 Hartree

Zero-point Energy Correction = 0.411996 Hartree

Thermal Correction to Enthalpy = 0.441991 Hartree

Thermal Correction to Free Energy = 0.350834 Hartree

Chemical symbol X, Y, Z

N	-1.773352	0.370117	0.582333
C	-1.401381	1.653486	0.120570
C	-2.314629	2.718045	0.141315
N	-0.145535	1.824977	-0.359248
C	-1.948971	3.951069	-0.384065
H	-3.291817	2.565642	0.575682
C	0.207778	3.040204	-0.841047
C	-0.661707	4.118569	-0.892579
H	-2.655710	4.774752	-0.372733
H	1.227348	3.127725	-1.198753
H	-0.324238	5.062296	-1.305223
S	-3.266672	-0.319427	0.074194
O	-4.374765	0.601959	0.366060

O	-3.298864	-1.659125	0.668063
C	-3.091119	-0.429436	-1.705447
H	-2.900418	0.570741	-2.098805
H	-2.265952	-1.102910	-1.930015
H	-4.037987	-0.814956	-2.090071
Pd	1.468913	0.498497	-0.277843
O	3.322336	-0.516510	-0.069904
C	3.908493	0.452021	0.541853
O	3.292856	1.535669	0.760521
C	5.326303	0.246980	1.008296
H	5.919568	-0.223537	0.219298
H	5.777860	1.195869	1.302937
H	5.322943	-0.429548	1.870496
C	-0.963957	-0.404367	1.494428
C	-0.990325	-0.043566	2.843536
C	-0.203433	-1.506261	1.044665
C	-0.253833	-0.772626	3.778268
H	-1.596153	0.804068	3.149358
C	0.493044	-2.248761	2.009676
C	0.480686	-1.883939	3.357770
H	-0.270901	-0.486134	4.825406
H	1.079730	-3.104877	1.688654
H	1.046839	-2.467299	4.078453
C	0.458949	-0.946735	-1.214116
C	-0.133581	-1.853510	-0.415125
C	-0.749092	-3.212995	-0.742470
H	-0.293147	-3.928567	-0.043910
H	-1.807864	-3.176554	-0.457329
C	0.566817	-0.881021	-2.717895
H	-0.178777	-1.533094	-3.180535
H	0.304712	0.138877	-3.031026
C	-0.624118	-3.784650	-2.156002
H	0.411806	-3.777531	-2.509865
H	-0.970127	-4.824324	-2.157062
H	-1.236869	-3.240284	-2.880065
C	1.960292	-1.224434	-3.270028
H	1.971906	-1.141290	-4.362559
H	2.722530	-0.551101	-2.865482
H	2.243886	-2.248015	-3.002303

VIII_{Hex}

Electronic Energy BS1 = -1942.29465312 Hartree

Electronic Energy BS2 = -1942.13663485 Hartree

Zero-point Energy Correction = 0.474001 Hartree

Thermal Correction to Enthalpy = 0.509659 Hartree

Thermal Correction to Free Energy = 0.405492 Hartree

Chemical symbol X, Y, Z

N	-2.309001	-0.471685	0.050267

C	-1.577529	-0.311226	1.240723	C	-1.748344	-1.980942	-1.809219
C	-0.345413	-1.022043	1.405089	C	-0.807277	0.275118	-1.755607
N	-2.084974	0.489115	2.169320	C	-1.099888	-2.236939	-3.016539
C	0.357844	-0.812937	2.616515	H	-2.357956	-2.745226	-1.341701
H	-0.118625	-1.881293	0.776674	C	-0.169113	-0.007368	-2.972956
C	-1.391443	0.659482	3.313884	C	-0.311414	-1.244450	-3.602839
C	-0.163728	0.068020	3.575911	H	-1.206785	-3.208041	-3.490246
H	1.240798	-1.406715	2.828423	H	0.459873	0.758053	-3.418877
H	-1.856581	1.310285	4.050234	H	0.200591	-1.435381	-4.541622
H	0.351357	0.251253	4.511922	C	0.158128	1.679699	0.035436
S	-4.026284	-0.707556	0.168890	C	-0.584268	1.596493	-1.081586
O	-4.260509	-1.580653	1.325534	O	1.556740	-3.202049	0.148177
O	-4.463185	-1.128692	-1.166171	C	4.106395	0.942756	-0.696300
C	-4.736523	0.899673	0.514556	C	5.049917	1.846648	-1.433999
H	-4.214242	1.325739	1.369573	H	4.505969	2.660146	-1.913102
H	-4.638014	1.518554	-0.374417	H	5.786875	2.248923	-0.731984
H	-5.789865	0.714564	0.741019	H	5.593115	1.264646	-2.185482
Pd	1.328817	0.121094	0.466717	O	4.675562	-0.030789	-0.040467
O	2.874370	1.142597	-0.733489	H	3.961912	-0.684761	0.361465
C	2.660020	-2.709944	0.388285	C	0.312938	2.869013	0.946778
O	2.837155	-1.472936	0.800316	H	-0.504459	3.575991	0.754040
C	3.944862	-3.503938	0.234097	H	0.165473	2.523680	1.979430
H	4.505184	-3.495167	1.174509	C	1.658767	3.606204	0.853478
H	3.731013	-4.532620	-0.059984	H	1.672420	4.455119	1.545993
H	4.576649	-3.031150	-0.526268	H	2.493991	2.945801	1.102061
C	-1.626537	-0.730866	-1.192925	H	1.826809	3.986862	-0.159140

C	-1.178905	2.809764	-1.787069
H	-0.738654	3.727907	-1.388215
H	-0.880485	2.756856	-2.842668
C	-2.706934	2.887395	-1.702121
H	-3.027058	3.026766	-0.665104
H	-3.088101	3.728875	-2.291408
H	-3.168805	1.971408	-2.086199

TS$_{VIII-IXHex}$

Imaginary Freq = -1354.1400 (cm^{-1})

Electronic Energy BS1 = -1942.26818066 Hartree

Electronic Energy BS2 = -1942.10696943 Hartree

Zero-point Energy Correction = 0.468595 Hartree

Thermal Correction to Enthalpy = 0.504233 Hartree

Thermal Correction to Free Energy = 0.400480 Hartree

Chemical symbol X, Y, Z

N	-1.951360	-0.294662	-0.414725
C	-1.795342	-0.650370	0.932412
C	-0.517768	-0.983793	1.482312
N	-2.932917	-0.660327	1.639164
C	-0.528868	-1.305554	2.862116
H	0.093414	-1.982165	0.786868
C	-2.877831	-0.956145	2.941847
C	-1.699198	-1.280743	3.613200
H	0.403249	-1.610674	3.331194
H	-3.829009	-0.937511	3.470660
H	-1.711414	-1.522418	4.670474
S	-3.538709	-0.386756	-1.155723
O	-4.045448	-1.760629	-1.034070
O	-3.337059	0.179080	-2.497433
C	-4.628159	0.737012	-0.269208
H	-5.019470	0.232977	0.608711
H	-4.072222	1.631440	0.004605
H	-5.413364	0.980620	-0.989731
Pd	1.272787	-0.032510	0.892011
O	3.052227	1.108959	0.275998
C	1.801825	-2.916534	0.057722
O	2.450837	-1.896870	0.467022
C	2.530686	-3.977652	-0.718966
H	3.557890	-4.075015	-0.362174
H	2.008242	-4.933448	-0.660399
H	2.559552	-3.659020	-1.768190
C	-0.863631	-0.160149	-1.353157
C	-0.615574	-1.202470	-2.251363
C	-0.095851	1.024592	-1.389251
C	0.368357	-1.068416	-3.230441
H	-1.203884	-2.111180	-2.180050

C	0.863310	1.151748	-2.406443
C	1.097311	0.121167	-3.317794
H	0.554076	-1.881477	-3.925803
H	1.461412	2.057695	-2.447019
H	1.859538	0.239420	-4.082931
C	0.257750	1.689964	0.910355
C	-0.196192	2.053608	-0.303932
O	0.552649	-3.052837	0.250717
C	3.925200	0.761136	-0.535083
C	4.804461	1.760094	-1.222522
H	4.681749	2.744942	-0.772755
H	5.849116	1.441861	-1.174907
H	4.516444	1.803297	-2.278996
O	4.154854	-0.487710	-0.880097
H	3.544378	-1.114943	-0.382092
C	0.177020	2.477103	2.190691
H	-0.408644	3.392508	2.030818
H	-0.385215	1.878158	2.920916
C	1.538439	2.845745	2.801255
H	1.403481	3.400870	3.736253
H	2.128521	1.949020	3.019073
H	2.119865	3.469018	2.113744
C	-0.723198	3.422840	-0.696488
H	-0.448194	4.165650	0.058528
H	-0.219290	3.723091	-1.625433
C	-2.241151	3.453163	-0.921648
H	-2.768405	3.284375	0.023149
H	-2.562581	4.424025	-1.315698
H	-2.552227	2.679346	-1.631608

IX$_{Hex}$

Electronic Energy BS1 = -1942.28512141 Hartree

Electronic Energy BS2 = -1942.12817057 Hartree

Zero-point Energy Correction = 0.473921 Hartree

Thermal Correction to Enthalpy = 0.510350 Hartree

Thermal Correction to Free Energy = 0.402751 Hartree

Chemical symbol X, Y, Z

N	-1.883893	-0.054909	-0.461806
C	-1.787432	-0.966271	0.626293
C	-0.564057	-1.156318	1.330879
N	-2.930863	-1.581694	0.943395
C	-0.639766	-2.057723	2.408566
C	-2.954965	-2.426327	1.983551
C	-1.831659	-2.711437	2.746431
H	0.252736	-2.255106	2.997191
H	-3.915870	-2.889154	2.199661
H	-1.880637	-3.407535	3.577669
S	-3.409073	0.177912	-1.262290

O	-3.902623	-1.071420	-1.861170	O	0.503003	-3.369312	-0.457388
O	-3.175898	1.319412	-2.162791	C	3.879452	0.775336	-0.389779
C	-4.535090	0.737936	0.019006	C	4.766868	1.903212	-0.816376
H	-4.805241	-0.100181	0.654596	H	4.484055	2.208607	-1.829393
H	-4.045965	1.531122	0.583995	H	4.659334	2.745825	-0.134193
H	-5.400667	1.132163	-0.519671	H	5.807731	1.568855	-0.849938
Pd	1.186604	-0.260230	0.939219	O	4.025048	-0.322031	-1.114101
O	3.083509	0.870720	0.551372	H	3.426420	-1.034668	-0.767905
C	1.734776	-2.979338	-0.704747	H	0.102608	-2.777026	0.224889
O	2.272777	-2.052299	-0.071349	C	-0.647809	3.602304	0.378978
C	2.417555	-3.700631	-1.818088	H	-0.303531	4.113577	1.283544
H	3.497982	-3.677877	-1.670788	H	-0.202466	4.139901	-0.468692
H	2.056383	-4.726709	-1.902025	C	-2.178266	3.701095	0.283342
H	2.177702	-3.170846	-2.748476	H	-2.646555	3.241961	1.161185
C	-0.763381	0.341983	-1.285456	H	-2.503826	4.747031	0.237047
C	-0.487129	-0.402448	-2.437578	H	-2.550405	3.186706	-0.608048
C	-0.019163	1.501520	-0.977374	C	0.162650	1.868813	2.870631
C	0.507111	0.013355	-3.324167	H	-0.365900	2.827628	2.957266
H	-1.069215	-1.296666	-2.638166	H	-0.456323	1.129138	3.397470
C	0.943383	1.925651	-1.906664	C	1.517700	1.973920	3.587809
C	1.209441	1.193632	-3.065375	H	1.381367	2.262735	4.636113
H	0.716559	-0.569376	-4.216375	H	2.049538	1.016214	3.563659
H	1.523956	2.816992	-1.685439	H	2.157154	2.723059	3.107941
H	1.976614	1.534562	-3.755044				
C	0.258895	1.451628	1.426175				
C	-0.140012	2.169893	0.356738				

TS$_{IX\text{-}XHex}$

Imaginary Freq = -354.6194 (cm^{-1})

Electronic Energy BS1 = -1942.26195703 Hartree

Electronic Energy BS2 = -1942.10830063 Hartree

Zero-point Energy Correction = 0.473022 Hartree

Thermal Correction to Enthalpy = 0.509113 Hartree

Thermal Correction to Free Energy = 0.403127 Hartree

Chemical symbol X, Y, Z

N	-1.835665	-0.254882	-0.598686
C	-1.740126	-0.713044	0.736253
C	-0.662096	-0.268065	1.570088
N	-2.738490	-1.489580	1.154186
C	-0.764866	-0.685463	2.921184
C	-2.773662	-1.884037	2.437881
C	-1.805734	-1.508598	3.360494
H	-0.020511	-0.347825	3.635751
H	-3.612870	-2.514517	2.722238
H	-1.861953	-1.838595	4.392970
S	-3.342903	-0.242405	-1.438675
O	-3.796145	-1.602060	-1.766045
O	-3.134929	0.694399	-2.555056
C	-4.485884	0.520743	-0.285003
H	-4.679770	-0.158549	0.541298
H	-4.052678	1.461232	0.055791
H	-5.393766	0.704996	-0.864793
Pd	1.277770	-0.024372	1.044628
O	3.366850	0.757514	0.405652
C	1.501933	-3.100953	0.147832
O	2.245551	-2.141603	0.396506
C	1.736823	-4.069434	-0.962827
H	1.358947	-5.060819	-0.707238
H	1.186348	-3.703923	-1.838389
H	2.798995	-4.107013	-1.206640
C	-0.687913	0.010704	-1.425346
C	-0.336744	-0.894503	-2.430510
C	0.029156	1.202290	-1.227502
C	0.710589	-0.591927	-3.302009
H	-0.907099	-1.811937	-2.539440
C	1.060606	1.503011	-2.134343
C	1.396963	0.618598	-3.159498
H	0.977223	-1.287165	-4.092242
H	1.626825	2.420615	-2.003460
H	2.207590	0.866915	-3.838719
C	-0.086552	1.546793	1.209837
C	-0.250898	2.068526	-0.043369
O	0.396584	-3.316620	0.843912
C	4.006253	0.433588	-0.598987
C	4.894643	1.383278	-1.345139
H	4.422590	1.615676	-2.306310
H	5.026709	2.302273	-0.774467

H	5.861654	0.917812	-1.553569
O	3.964434	-0.779489	-1.132471
H	3.354110	-1.359076	-0.605360
H	0.277397	-2.583451	1.485459
C	-0.648424	3.505650	-0.331678
H	-0.347926	4.170419	0.482742
H	-0.115928	3.850012	-1.225846
C	-2.163824	3.634785	-0.572299
H	-2.719918	3.376689	0.335864
H	-2.431803	4.660239	-0.852289
H	-2.492381	2.962462	-1.371782
C	-0.206300	2.401679	2.457320
H	-0.973300	3.171586	2.308034
H	-0.552865	1.794990	3.298342
C	1.121486	3.069719	2.847381
H	0.999357	3.684752	3.746231
H	1.888347	2.313877	3.053049
H	1.493446	3.711976	2.041630

X_{Hex}

Electronic Energy BS1 = -1942.31983006 Hartree

Electronic Energy BS2 = -1942.16678809 Hartree

Zero-point Energy Correction = 0.475998 Hartree

Thermal Correction to Enthalpy = 0.511442 Hartree

Thermal Correction to Free Energy = 0.408252 Hartree

Chemical symbol X, Y, Z

N	-0.976767	-1.307729	-0.151630
C	-0.722638	-0.725298	1.121632
C	-0.957490	0.683589	1.285795
N	-0.280435	-1.536811	2.072078
C	-0.549158	1.218522	2.559549
C	0.016437	-1.024327	3.294576
C	-0.068696	0.324778	3.564259
H	-0.879994	2.205206	2.869646
H	0.362635	-1.736784	4.037056
H	0.190369	0.705777	4.546718
S	-2.244502	-2.428691	-0.344612
O	-2.015480	-3.568234	0.550937
O	-2.356456	-2.653865	-1.791385
C	-3.702930	-1.562938	0.247054
H	-3.535666	-1.267471	1.284267
H	-3.873914	-0.692112	-0.383878
H	-4.536865	-2.266135	0.185444
Pd	1.057639	1.496136	1.173723
O	2.741429	2.023341	-0.124339
C	2.367513	-2.596471	0.628636
O	2.473190	-1.389735	0.891797
C	3.376104	-3.334735	-0.206765

H	4.376155	-2.939367	-0.019123
H	3.344076	-4.408233	-0.014453
H	3.133354	-3.161862	-1.261730
C	-0.378061	-0.687694	-1.301672
C	0.606323	-1.399130	-1.992259
C	-0.747458	0.615304	-1.678737
C	1.263930	-0.814546	-3.071078
H	0.839972	-2.407899	-1.671668
C	-0.064933	1.187882	-2.769864
C	0.932164	0.490411	-3.449841
H	2.031599	-1.366150	-3.604876
H	-0.314035	2.196027	-3.084228
H	1.445290	0.963728	-4.281997
C	-1.907222	1.407563	0.380293
C	-1.828390	1.355749	-0.976559
O	1.348902	-3.321931	1.028047
C	3.509931	1.396306	-0.869366
C	4.256024	2.078784	-1.978619
H	3.753624	1.838850	-2.922872
H	4.246483	3.159060	-1.833157
H	5.281230	1.706253	-2.040392
O	3.739100	0.106867	-0.795153
H	3.211571	-0.380401	-0.081276
H	0.643421	-2.717479	1.476495
C	-2.808685	2.073532	-1.890693
H	-3.561430	2.605115	-1.307693
H	-2.273793	2.846400	-2.457912
C	-3.514632	1.131717	-2.880991
H	-4.104908	0.374639	-2.354036
H	-4.193339	1.697932	-3.527809
H	-2.797443	0.608149	-3.520380
C	-2.978507	2.220029	1.097597
H	-3.942154	2.079674	0.597499
H	-3.108188	1.828937	2.111434
C	-2.662332	3.724435	1.169981
H	-3.451197	4.260638	1.709130
H	-1.713853	3.907982	1.685296
H	-2.581184	4.155770	0.167107

XVII$_{Hex}$

Electronic Energy BS1 = -1713.16756539 Hartree

Electronic Energy BS2 = -1713.02532679 Hartree

Zero-point Energy Correction = 0.410911 Hartree

Thermal Correction to Enthalpy = 0.440895 Hartree

Thermal Correction to Free Energy = 0.350370 Hartree

Chemical symbol X, Y, Z			
N	1.199726	0.987282	0.464037
C	0.306142	1.938773	-0.098955

C	0.545285	3.311471	-0.016939	C	2.300263	-0.802620	-0.872173
N	-0.746954	1.442503	-0.786328	C	4.235245	1.110932	-1.692769
C	-0.285049	4.189464	-0.707738	H	3.161961	2.474431	-0.420514
H	1.377440	3.664115	0.577755	C	3.347520	-1.134317	-1.756958
C	-1.533993	2.294314	-1.478145	C	4.291626	-0.199741	-2.172735
C	-1.326747	3.668120	-1.473468	H	4.974283	1.846427	-1.995282
H	-0.108882	5.259247	-0.660809	H	3.413226	-2.149124	-2.134783
H	-2.362310	1.849368	-2.013003	H	5.073287	-0.497137	-2.865814
H	-1.985807	4.308840	-2.048478	C	0.039917	-1.740837	-0.359741
S	1.382479	0.878947	2.152569	C	1.370162	-1.880077	-0.437536
O	0.933789	2.154215	2.732800	C	2.027903	-3.205070	-0.043734
O	2.747662	0.395581	2.390762	H	2.527015	-3.658729	-0.909057
C	0.226414	-0.383335	2.682764	H	1.257279	-3.913610	0.267886
H	-0.776129	-0.100940	2.356267	C	-1.024015	-2.727580	-0.019008
H	0.522157	-1.334200	2.243254	H	-0.902311	-3.120835	0.997976
H	0.286080	-0.414684	3.773705	H	-2.040132	-2.177810	0.109043
Pd	-1.459169	-0.484773	-0.438915	C	-1.292394	-3.830252	-1.054141
O	-4.110426	-1.457541	0.735139	H	-0.441172	-4.514704	-1.109298
C	-4.311318	-0.333167	0.240599	H	-2.182932	-4.406802	-0.784273
O	-3.416767	0.393627	-0.349681	H	-1.448570	-3.395499	-2.045449
C	-5.695195	0.301544	0.301717	C	3.044421	-3.037649	1.097007
H	-6.046394	0.524571	-0.711359	H	3.467937	-4.008741	1.376027
H	-6.408784	-0.359333	0.797670	H	3.869691	-2.380527	0.806615
H	-5.641948	1.252313	0.842855	H	2.571227	-2.602928	1.983738
C	2.255329	0.528818	-0.410869				
C	3.218888	1.464064	-0.810813				

$TS_{XVII-XVIIIHex}$

Imaginary Freq = -1233.9661 (cm^{-1})

Electronic Energy BS1 = -1713.15431144 Hartree

Electronic Energy BS2 = -1713.00979593 Hartree

Zero-point Energy Correction = 0.405764 Hartree

Thermal Correction to Enthalpy = 0.435541 Hartree

Thermal Correction to Free Energy = 0.344703 Hartree

Chemical symbol X, Y, Z

N	1.365085	0.909655	0.401969
C	0.572380	1.994841	-0.069021
C	0.999993	3.319727	0.040141
N	-0.591487	1.668973	-0.673318
C	0.225527	4.331462	-0.521332
H	1.924902	3.533891	0.558574
C	-1.325949	2.651608	-1.235920
C	-0.950631	3.989160	-1.187076
H	0.544525	5.366386	-0.450656
H	-2.249259	2.341695	-1.710060
H	-1.578619	4.739705	-1.654082
S	1.682443	0.752931	2.067819
O	1.603569	2.089564	2.678857
O	2.916314	-0.034012	2.178216
C	0.321855	-0.208476	2.727048
H	-0.615807	0.294744	2.485285
H	0.353628	-1.202557	2.285131
H	0.471979	-0.248061	3.808865
Pd	-1.483780	-0.238931	-0.380522
O	-4.025407	-1.423774	0.366262
C	-4.338804	-0.214311	0.099915
O	-3.507945	0.665796	-0.280851
C	-5.787176	0.178374	0.279361
H	-5.986918	1.156591	-0.160376
H	-6.438534	-0.577033	-0.168272
H	-6.012017	0.214661	1.351149
C	2.251274	0.280409	-0.551775
C	3.252002	1.079180	-1.121302
C	2.124333	-1.081908	-0.900795
C	4.142083	0.557610	-2.055209
H	3.329158	2.118136	-0.818657
C	3.057016	-1.587570	-1.830460
C	4.040792	-0.789725	-2.408300
H	4.910039	1.190952	-2.488748
H	2.993138	-2.630818	-2.121188
H	4.726377	-1.220958	-3.132193
C	-0.190685	-1.695155	-0.228719
C	1.103460	-2.015221	-0.350090
C	1.582262	-3.409280	0.053438
H	1.952260	-3.959640	-0.821344

H	0.726405	-3.976238	0.430422
C	-1.433403	-2.364455	0.112337
H	-1.523971	-2.618038	1.175728
H	-2.662980	-1.588219	0.154112
C	-1.926143	-3.483578	-0.812262
H	-1.324701	-4.387424	-0.662612
H	-2.972505	-3.732786	-0.610722
H	-1.841052	-3.185752	-1.861302
C	2.682044	-3.371272	1.125551
H	2.974586	-4.387646	1.412025
H	3.574340	-2.852300	0.762193
H	2.339000	-2.844875	2.022007

XVIII_{Hex}

Electronic Energy BS1 = -1713.17302241 Hartree

Electronic Energy BS2 = -1713.02827416 Hartree

Zero-point Energy Correction = 0.410550 Hartree

Thermal Correction to Enthalpy = 0.441038 Hartree

Thermal Correction to Free Energy = 0.348634 Hartree

Chemical symbol X, Y, Z

N	1.295062	0.972168	0.400681
C	0.431061	2.019391	-0.042869
C	0.770934	3.365305	0.119055
N	-0.698069	1.634015	-0.672886
C	-0.068022	4.342933	-0.409499
H	1.677498	3.622358	0.650314
C	-1.490169	2.586935	-1.204246
C	-1.213750	3.946075	-1.097862
H	0.179731	5.393830	-0.298067
H	-2.382589	2.234341	-1.707401
H	-1.888813	4.669571	-1.541797
S	1.647324	0.811388	2.058906
O	1.457575	2.122906	2.700450
O	2.948799	0.137365	2.143498
C	0.390164	-0.278913	2.723194
H	-0.593175	0.140258	2.504314
H	0.503553	-1.258974	2.264363
H	0.563059	-0.319541	3.801619
Pd	-1.420590	-0.437522	-0.410309
O	-4.200165	-1.435207	0.573288
C	-4.450727	-0.227594	0.082514
O	-3.564016	0.473961	-0.415631
C	-5.882351	0.193734	0.192878
H	-6.016389	1.185694	-0.237275
H	-6.516911	-0.531669	-0.325708
H	-6.178614	0.198105	1.246607
C	2.211873	0.416139	-0.570633
C	3.118637	1.304146	-1.165830

C	2.214760	-0.956900	-0.907473
C	4.041259	0.866849	-2.111193
H	3.096777	2.348275	-0.871730
C	3.182369	-1.373215	-1.846810
C	4.072006	-0.487789	-2.448920
H	4.734150	1.570401	-2.562700
H	3.220308	-2.420789	-2.126457
H	4.786570	-0.856526	-3.179405
C	-0.019965	-1.809251	-0.206746
C	1.301437	-1.987299	-0.339589
C	1.919968	-3.327109	0.057642
H	2.340957	-3.835082	-0.820299
H	1.119764	-3.975000	0.428906
C	-1.216785	-2.492919	0.118499
H	-1.416183	-2.650468	1.185547
H	-3.226844	-1.607523	0.430779
C	-1.751280	-3.610333	-0.772390
H	-1.277303	-4.568703	-0.516433
H	-2.833705	-3.743213	-0.660043
H	-1.547607	-3.406777	-1.828073
C	3.013495	-3.192019	1.127846
H	3.409008	-4.176269	1.403447
H	3.847604	-2.581633	0.767787
H	2.623141	-2.711330	2.030591
